# Photocatalytic redox-neutral selective single C(sp^3^)–F bond activation of perfluoroalkyl iminosulfides with alkenes and water†

**DOI:** 10.1039/d3sc03771a

**Published:** 2023-10-07

**Authors:** Tao Wang, Yuan-Yuan Zong, Tao Huang, Xiao-Ling Jin, Li-Zhu Wu, Qiang Liu

**Affiliations:** a State Key Laboratory of Applied Organic Chemistry, College of Chemistry and Chemical Engineering, Lanzhou University Lanzhou 730000 China liuqiang@lzu.edu.cn; b Key Laboratory of Photochemical Conversion and Optoelectronic Materials, Technical Institute of Physics and Chemistry, The Chinese Academy of Sciences Beijing 100190 P. R. China

## Abstract

Visible-light-promoted site-selective and direct C–F bond functionalization of polyfluorinated iminosulfides was accomplished with alkenes and water under redox-neutral conditions, affording a diverse array of γ-lactams with a fluoro- and perfluoroalkyl-substituted carbon centre. A variety of perfluoroalkyl units, including C_2_F_5_, C_3_F_7_, C_4_F_9_, and C_5_F_11_ underwent site-selective defluorofunctionalization. This protocol allows high chemoselectivity control and shows excellent functional group tolerance. Mechanistic studies reveal that the remarkable changes of the electron geometries during the defluorination widen the redox window between the substrates and the products and ensure the chemoselectivity of single C(sp^3^)–F bond cleavage.

## Abstract 2

Site-selective and direct C–F bond functionalization of polyfluorinated iminosulfides was achieved to synthesize 3-fluoro-3-perfluoroalkyl-γ-lactams under photoredox-neutral conditions.

## Graphical abstract

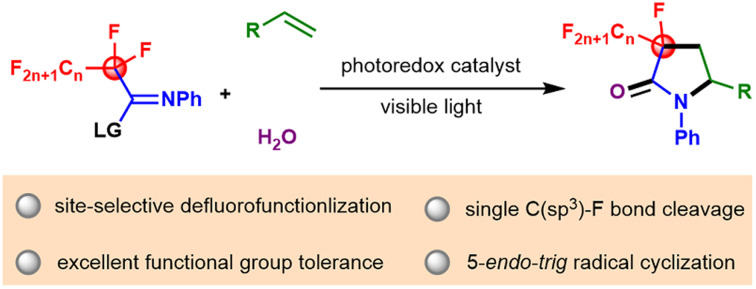

## Introduction

**Fig. 1:**
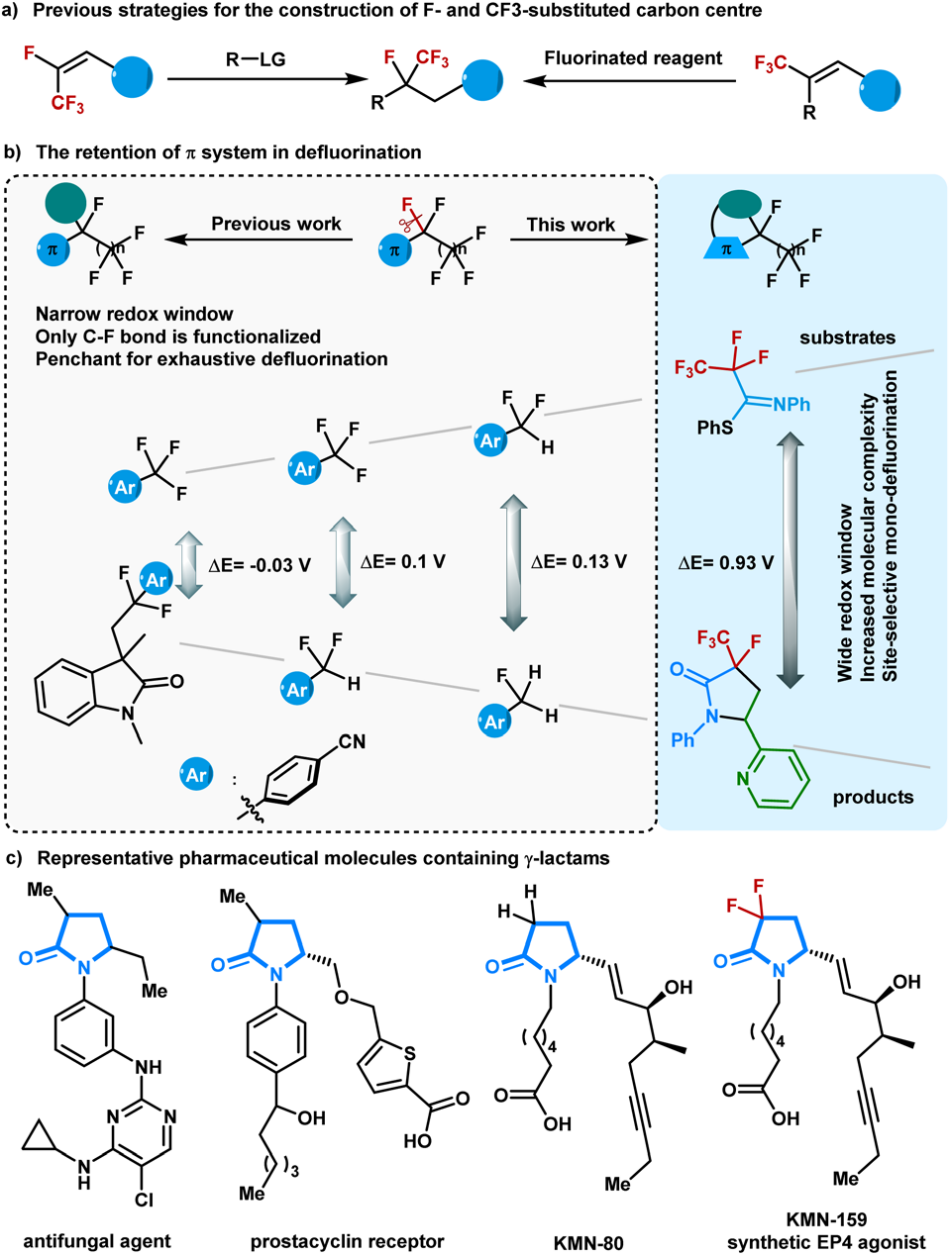
Selective defluorofunctionalization.

Polyfluorinated compounds have been exploited in pharmaceutical, agrochemical, and materials sciences owing to the unique physical and biological properties of fluorine atoms.^1–8^ In particular, the preparation of compounds containing a tetrasubstituted tertiary carbon center with a trifluoromethyl group and a fluorine atom has recently drawn increasing attention due to their unusual reactivity profiles to corresponding host compounds.^9–14^ The primary methods for synthesizing these molecules largely rely on specialized fluorinated functional groups and/or the stoichiometric use of fluorinating reagents.^15–23^ Although selective C–F bond functionalization of easily accessible multifluorinated compounds has shown massive potential for atom- and step-economic access to complex fluorine-containing molecules,^24–30^ the concise synthesis of compounds containing a tetrasubstituted tertiary carbon center with a trifluoromethyl group and a fluorine atom using defluoroalkylation strategies remains underdeveloped (Fig. 1a). This may be because of the high bond dissociation energy of the C(sp^3^)–F bond and the stepwise decreased C–F bond strength during the defluorination processes.^31–35^

Visible-light-driven photoredox catalysis avoids harsh reaction conditions and enables the generation of strong reducing intermediates for inert C–F bond activation.^36–38^ Several visible-light-absorbing photocatalysts with high reducing capability have been successfully explored to realize defluorinated functionalization of multifluorinated compounds containing aroyls or electron-withdrawing aryls.^39–41^ In another inventively-designed strategy, carbon dioxide radical anion, a powerful single electron reductant *in situ* generated in photoredox cycles, was elegantly designed for activating C(sp^3^)–F bonds in trifluoromethyl (hetero)arenes, trifluoroacetamides, and trifluoroacetates.^42–47^ So far, most of the π-systems situated next to fluoroalkyl groups have focused on arenes and carbonyl groups, and the commonly explored defluorinated products contain the starting π-systems (Fig. 1b). The electron geometries of the defluorinated products are highly similar to those of the starting polyfluorinated compounds, resulting in a narrow redox window between the substrates and the defluorination products. Although selective defluorination proceeds predominantly because electron-transfer events for the polyfluorinated substrates are slightly more exergonic than those for the corresponding mono-defluorinated products,^48^ the narrow redox window between the substrates and the defluorination products still gives rise to undesirable side-products. As such, an extension of reaction types for the visible-light-driven defluorination of C(sp^3^)–F bonds and ultimately toward the exclusive formation of mono-defluorinated products is deemed worthy of pursuit.

In seeking a solution to circumvent these challenges, we were drawn to readily accessible imines that have shown abundant chemical properties in photoredox catalysis. Conceptually, the installation of an imine next to the polyfluoroalkyl chain could facilitate the defluorinative spin-center shift (SCS) process.^49^ We anticipate that polyfluorinated imines will provide the defluorinated products with diverse molecular scaffolds,^50^ which can thoroughly exclude exhaustive defluorinations due to the distinct redox window. Herein, a photoredox-neutral single C(sp^3^)–F bond activation method was developed for the radical cycloaddition of perfluoroalkyl imines with alkenes and water. By tuning the perfluoroalkyl imines structure, we achieved γ-lactams with a fluoro- and perfluoroalkyl-substituted carbon centre *via* an imine-involved 5-*endo-trig* radical cyclization, which is generally considered to be kinetically unfavorable according to Baldwin's rules.

γ-Lactams are basic structural elements found in many complex natural products and pharmaceutical compounds (Fig. 1c).^51^ In particular, 3-alkyl-γ-lactams are core structural motifs in antifungal agents and prostacyclin receptors. The introduction of a CF_2_ group into γ-lactams with interesting biological profiles has been intensively studied.^52^ Nevertheless, the synthesis of γ-lactams containing C–F and C–CF_3_ bonds at one carbon remains challenging and has never been explored until now. With a wide range of polyfluorinated imines being easily prepared from polyfluorocarboxylic acids, the present approach allows access to a variety of multifluorinated γ-lactams with rapid modifications.

## Results and discussion

The success of the proposed strategy requires suitable polyfluorinated imine derivatives that can readily undergo single-electron reduction and subsequent fluoride elimination under mild reaction conditions. Through initial exploration, we found that the selective defluorination of phenyl 2,2,3,3,3-pentafluoro-propanimidothioate (PFIT) 1a could indeed be realized under photoredox conditions (Table S1†). After extensive optimization, the reaction among PFIT 1a, water, and 2-vinylpyridine 2a efficiently afforded 3-fluoro-3-trifluoromethyl-γ-lactam 3a (Table 1). The model reaction could be used to optimize the reaction conditions (Tables 1 and S2–S5†). The optimized reaction conditions include 1 mol% of Ir(ppy)_3_ as a photocatalyst, and CH_3_CN as the solvent, with 2 equivalents of cesium carbonate under blue light irradiation (6 W, 450 nm). Under the standard conditions, the corresponding 3-fluoro-3-trifluoromethyl-γ-lactam 3a was obtained in 83% yield with high diastereoselectivity (Table 1, entry 1). Other reducing photocatalysts, such as 4-CzIPN, [Ir(ppy)_2_(dtbbpy)]^+^ and [Ru(bpy)_3_]^2+^ were unable to promote the reaction (entries 2–4). The evaluation of the solvent revealed that this transformation proceeds best in acetonitrile (entries 1 and 5–8). A lower yield of 3a was isolated when K_2_HPO_4_, NaHCO_3_, and KOH were used as bases or in the absence of a base (entries 9–12). We conducted control experiments to confirm the essential role of light and photocatalyst in the success of this transformation (entries 13 and 14).

**Table 1:** Optimization of the reactions conditions^a^

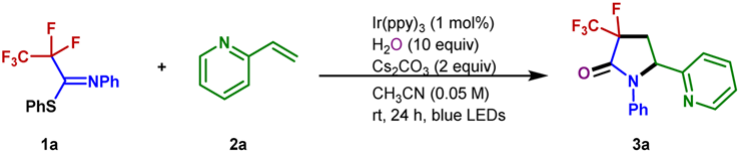			
Entry	Deviation	Yield^b^ (%)	dr^c^
1	None	83	12 : 1
2	4-CzIPN	N.D	
3	[Ir(ppy)_2_(dtbbpy)]PF_6_	N.D	
4	[Ru(bpy)_3_]Cl_2_	N.D	
5	DMF	72	11 : 1
6	DMSO	51	5 : 1
7	Acetone	72	9 : 1
8	THF	N.D	
9	K_2_HPO_4_	70	11 : 1
10	NaHCO_3_	58	10 : 1
11	KOH	69	10 : 1
12	No base	32	11 : 1
13	No catalyst	N.D	
14	No light	N.D	

a Reaction conditions: 1a (0.2 mmol), 2a (0.1 mmol), Cs_2_CO_3_ (0.2 mmol), H_2_O (1 mmol), and Ir(ppy)_3_ (0.001 mmol), CH_3_CN (2 mL), irradiation with 6 W blue LEDs at rt for 24 h.

b Isolated yield; N.D. = not detected.

c Detected by ^19^F NMR or ^1^H NMR.

**Fig. 2:**
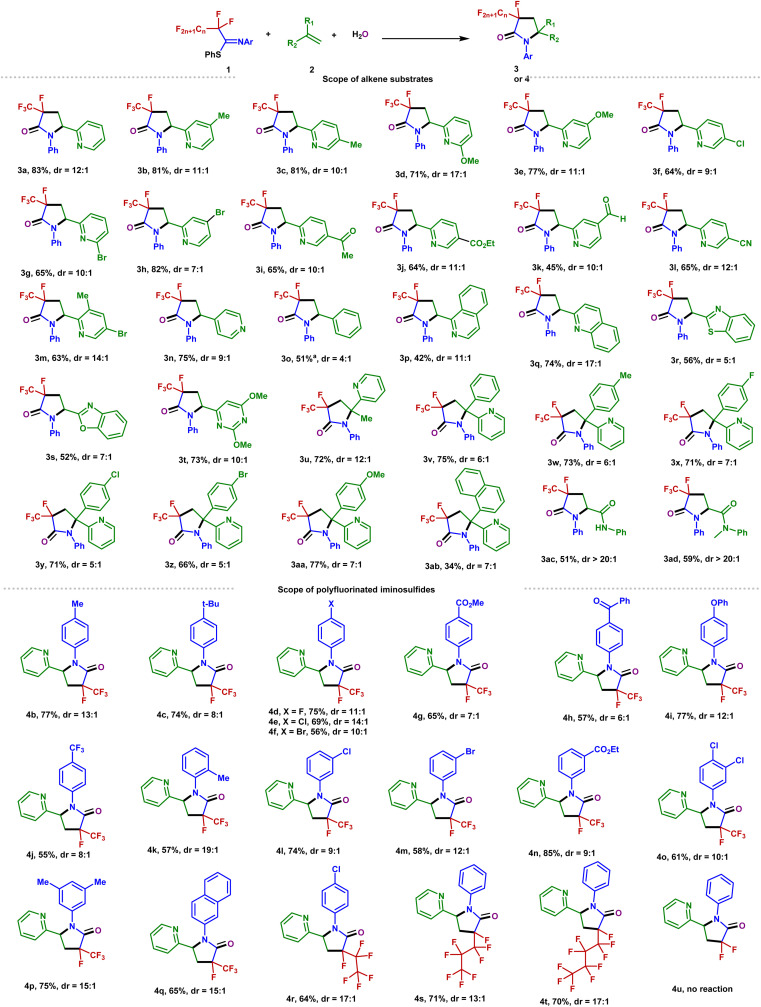
Substrate scope. Unless otherwise noted, all reactions were conducted using 1 (0.2 mmol), 2 (0.1 mmol), Cs_2_CO_3_ (0.2 mmol), H_2_O (1 mmol), and Ir(ppy)_3_ (0.001 mmol), CH_3_CN (2 mL), irradiation with 6 W blue LEDs at rt for 24 h, with isolated yields shown. ^*a*^Using 3 equiv. styrene.

With the optimized conditions in hand, the scope of the alkenes was explored with the representative examples shown in Fig. 2. Due to the ubiquitous role of pyridyl motifs as aromatic heterocycles in ligand scaffolds, natural products, and medically relevant molecules, a wide range of structurally diverse 2-vinylpyridines possess different kinds of functional groups were subjected to this protocol. It was found that a variety of substituted 2-vinylpyridines reacted well under the conditions, giving rise to the corresponding products in moderate to high yields with high to excellent diastereoselectivities. Various functional groups, including methyl, bromo, chloro, ketone, ester, aldehyde, methoxyl, and cyano were well tolerated (3a–3m). Although a pyridyl halide moiety has been known to be activated by strong reducing photocatalysts, we did not observe any decomposition in the defluorination.^53,54^ The benign compatibility of halogen substituents further emphasizes the potential synthetic applications. Subsequently, reactions of 4-vinylpyridine and styrene were examined, and the desired products (3n–3o) were produced as expected. Other heterocyclic substituted olefins such as quinoline, isoquinoline, benzothiazole, benzoxazole, and pyrimidine were also compatible under the standard conditions, and the corresponding 3-fluoro-3-trifluoromethyl-γ-lactams were generated efficiently (3p–3t). When a variety of 1,1-disubstituted vinylpyridines were employed, the desired products (3u–3ab) were obtained in 34–77% yields. Furthermore, the acrylamides were also accommodated and converted into the corresponding products with excellent diastereoselectivity (3ac–3ad). Unfortunately, inactive olefins and 1,2-disubstituted alkenes proved to be unsuitable for this transformation.

To further explore the potential of this efficient defluoroalkylation reaction, a variety of polyfluorinated iminosulfides were investigated. As shown in Fig. 2, this method exhibits good substrate compatibility and excellent selectivity. Polyfluorinated iminosulfides bearing various substituents at the aromatic ring could be converted to the desired products 4b–4p in moderate to high yields with high to excellent diastereoselectivities. Notably, the reaction was well-compatible with a range of functional groups (*e.g.*, F, Cl, Br, CF_3_, ether, ester, ketone) commonly encountered in organic synthesis. It should be noted that cleavage of the aryl C–F, C–Cl, C–Br, and even the benzylic C(sp^3^)–F bonds did not occur in these cases, indicating the excellent site-selectivity of the present C–F activation. In addition, *N*-naphthalen-2-yl-PFIT could also give the desired product 4q in 65% yield. To our delight, selective C–F bond functionalization of C_3_F_7_, C_4_F_9_, and C_5_F_11_ groups was also achievable to this method (4r–4t). Unfortunately, when phenyl 2,2,2-trifluoro-*N*-phenylethanimidothioate was used, the desired cyclized product could not be obtained (4u), presumably owing to the stabilization of radical *via* fluoride elimination from the corresponding radical anion.

The generation of 3-fluoro-3-trifluoromethyl-γ-lactam 3a could be scaled up to 4 mmol without a dramatic decrease in yield (72% yield). To demonstrate the synthetic utility of the transformation, the product 3a was further derivatized (Fig. 4). Treatment of 3a with 9-borabicyclo[3.3.1]nonane (9-BBN) in refluxing THF resulted in the formation of the 4-fluoro-4-trifluoromethyl-pyrrolidine 5a in 70% isolated yield. A reduction of 3a with LiAlH_4_ enabled the 3-fluoro-3-trifluoromethyl-pyrrolidin-2-ol 5b to be generated in 55% yield.

**Fig. 3:**
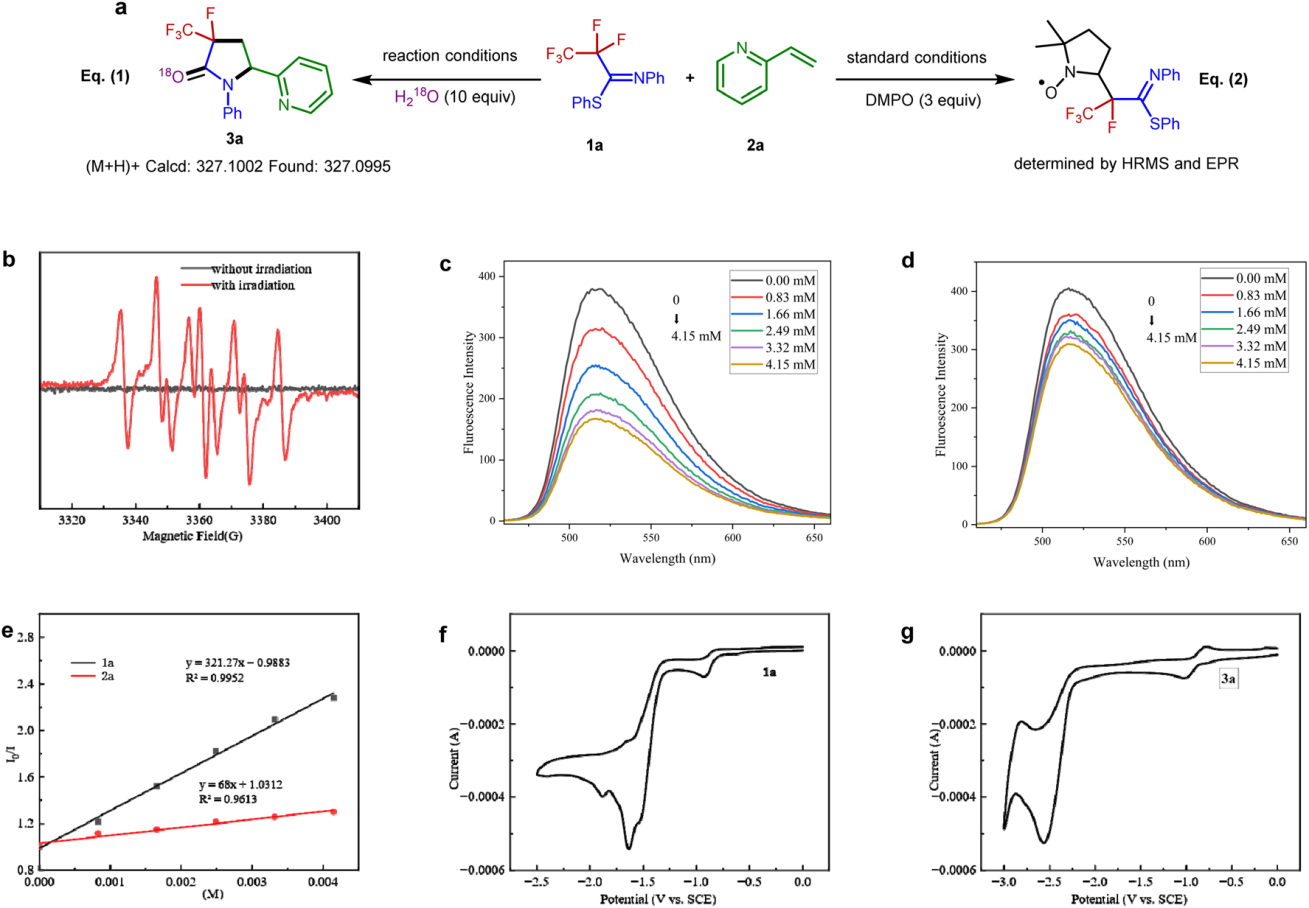
Mechanistic investigations. (a) ^18^O-Labeling experiment and EPR experiment. (b) EPR spectrum. (c) Fluorescence quenching experiments with various concentrations of 1a. (d) Fluorescence quenching experiments with various concentrations of 2a. (e) Stern–Volmer plots with different quenchers. (f) Cyclic voltammograms of 1a. (g) Cyclic voltammograms of 3a.

To further probe the mechanism, some control experiments were investigated. The ^18^O-labeling results proved that the oxygen atom in the amide group originates from water [Fig. 3a, eqn (1)]. In the presence of a radical scavenger 2,2,6,6-tetramethylpiperidin-1-yloxy (TEMPO), the reaction was completely shut down, suggesting the intermediacy of radical formation in this transformation. However, all efforts to the isolation of any TEMPO adduct remained unsuccessful.

**Fig. 4:**
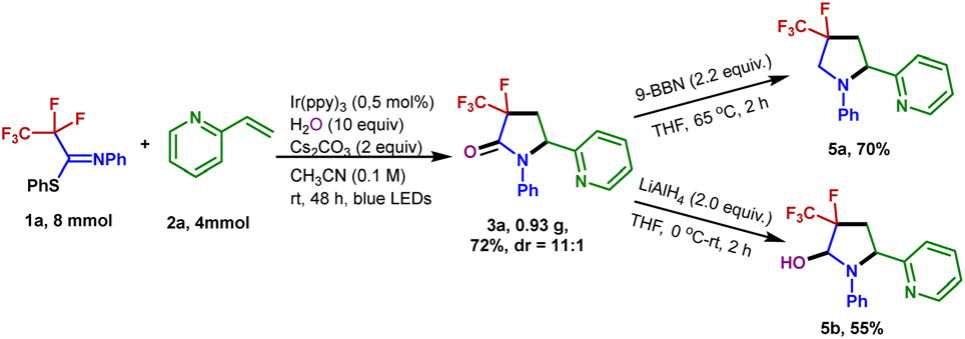
Gram-scale experiment and synthetic transformations of 3a.

Furthermore, the reaction was monitored by electron paramagnetic resonance spectroscopy with 5,5-dimethyl-1-pyrroline *N*-oxide as the radical trap [Fig. 3a, eqn (2)] (Fig. S1†). The result of this experiment is consistent with the radical nature of this defluorination process (Fig. 3a). Stern–Volmer fluorescence quenching experiments of the Ir(ppy)_3_ catalyst with reagents PFIT 1a and 2-vinylpyridine 2a were carried out. As shown in Fig. 3c and d, the results clearly show that only PFIT 1a could dramatically quench the excited state Ir(ppy)_3_ (Fig. 3e). Such findings demonstrate that the SET reduction of 1a is likely the first step in the photo-catalytic cycle *via* oxidative quenching [*Ir(iii)/Ir(iv)] pathway.

In order to get insight into the photoredox process, cyclic voltammetry experiments were further analyzed. The measured reduction potential of 1a is −1.63 V (peak potential *vs.* SCE, see Fig. 3f), which is much higher than the reduction potential of 3a (−2.56 V *vs.* SCE, see Fig. 3g). The wide redox window between the substrates and the defluorination products controls the chemoselectivity of the single C(sp^3^)–F bond cleavage.

**Fig. 5:**
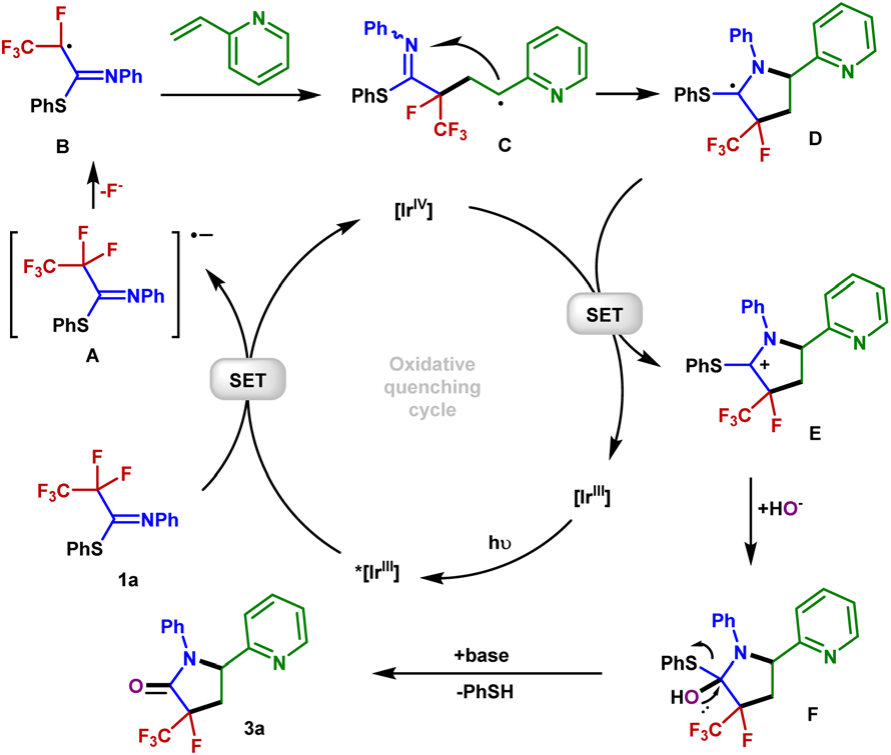
Proposed mechanism.

**Fig. 6:**
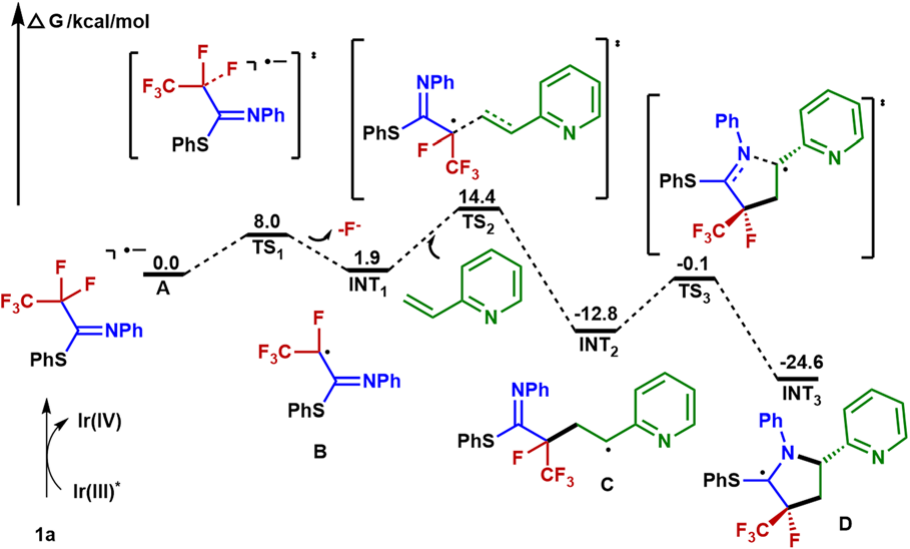
Free energy profile.

Based on our mechanistic investigations and precedent literature,^55,56^ we proposed a plausible reaction mechanism outlined in Fig. 5. Initially, irradiation of [Ir]^III^ gives rise to its excited state *[Ir]^III^. Then 1a is reduced by *[Ir]^III^*via* SET, affording the Ir(iv) species and radical anion A. Next, an SCS process occurs to generate the corresponding radical B with the cleavage of a C–F bond. The 2-vinylpyridine 2a captures B to form the alkyl radical intermediate C, which is intercepted by the C=N double bond of the imine group *via* a 5-*endo-trig* cyclization to give the C-centered radical intermediate D. Described by Baldwin's rules, 5-*endo-trig* cyclizations are generally considered to be kinetically unfavorable. However, in our case, density functional theory using the (U)M06-2X/6-311+G(d,p)/SMD(CH_3_CN) method revealed that such a 5-*endo-trig* cyclization of C requires an activation free energy (Δ*G*^‡^ = 12.7 kcal mol^−1^) and is exergonic by 11.8 kcal mol^−1^ (Fig. 6). Thus, this route is considered feasible.^57^ The photoredox cycle is then closed by SET between D and [Ir]^IV^ to afford the cationic intermediate E, which is trapped by the hydroxyl anion to afford the intermediate F. Finally, F undergoes elimination at the prompting of the base to give a polyfluorinated γ-lactam 3a and thiophenol. In addition, an alternative mechanism for styrene may be considered. The intermediate C is oxidized by Ir(iv) into the corresponding benzylic carbocation and subsequently undergoes a 5-*endo-trig* cyclization into intermediate E. This cyclization process is very kinetically favored, thus rendering the lower diastereoselectivities (see the ESI† for details).

In addition, DFT calculations (Fig. S7†) suggest that radical anion A delocalizes in a π orbital on the benzene ring and also in the σ* orbitals of two C–F bonds (C1–F1 and C1–F2), and these C–F bonds of A are lengthened by comparison with those of neutral 1a (from 1.353 to 1.400 Å). These distinctive chemical characteristics ensure exquisite chemoselectivity during defluorination.

In summary, a direct and site-selective C(sp^3^)–F bond alkylation in polyfluorinated iminosulfides with alkenes and water was accomplished *via* photoredox catalysis, affording a diverse array of 3-fluoro-3-perfluoroalkyl-γ-lactams with concomitant formation of one C(sp^3^)–C(sp^3^) bond, one C(sp^3^)–N bond and one C=O bond. This protocol allows for achieving more complex fluorine-containing molecules in a single step. The π-systems next to fluoroalkyl groups are functionalized simultaneously during defluorination, widening the redox window between the substrates and the defluorination products. These reactivity characteristics are crucial to control the single C(sp^3^)–F bond cleavage and avoid exhaustive defluorination. This novel methodology is anticipated to provide an efficient synthetic toolbox for further drug discovery and development. Further investigations on the development of photocatalytic C–F bond activation are currently ongoing in our laboratory.

## Data availability

The datasets supporting this article have been uploaded as part of the ESI.†

## Author contributions

Q. L. and T. W. conceived the idea and wrote the manuscript. T. W. and Y.-Y. Z. performed the experiments and analyzed the data. T. H. and X.-L. J. performed the computational studies. L.-Z. W. discussed the results and commented on the manuscript. All authors have given approval to the final version of the manuscript.

## Conflicts of interest

There are no conflicts to declare.

## Supplementary Material

SC-014-D3SC03771A-s001

SC-014-D3SC03771A-s002
