# Supplementary material for: Photocatalytic redox-neutral selective single C(sp3)–F bond activation of perfluoroalkyl iminosulfides with alkenes and water
Source: Chem Sci. 2023 Oct 7;14(41):11566–72. doi: 10.1039/d3sc03771a (PMC10599478; doi:10.1039/d3sc03771a)
Supplement: SC-014-D3SC03771A-s001 [file SC-014-D3SC03771A-s001.pdf]

Electronic Supplementary Material (ESI) for Chemical Science.
This journal is © The Royal Society of Chemistry 2023

# Supporting Information

## Photocatalytic Redox-Neutral Selective Single C(sp3)–F Bond Activation of Perfluoroalkyl Imines under Redox-Neutral Conditions

Tao Wang,[†] Yuan-Yuan Zong,[†] Tao Huang,[†] Xiao-Ling Jin,[†] Li-Zhu Wu,[‡] and Qiang Liu[*,†]

[a]State Key Laboratory of Applied Organic Chemistry, College of Chemistry and Chemical Engineering, Lanzhou University, Lanzhou 730000, P. R. China

[b]Key Laboratory of Photochemical Conversion and Optoelectronic Materials, Technical Institute of Physics and Chemistry, the Chinese Academy of Sciences, Beijing 100190, P. R. China

*E-mail: liuqiang@lzu.edu.cn

**I. General Information** .......... S2
**II. Detailed Reaction Optimizations** .......... S3
**III. Gram-Scale Synthesis of 3a** .......... S6
**IV. General Synthetic Procedures** .......... S6
**1. General Procedure for Single C(sp$^3$)-F Bond Cleavage** .......... S6
**2. Synthesis of Alkenylpyridines** .......... S6
**3. Synthesis of Perfluoroalkyl Group Substituted Iminosulfides** .......... S8
**4. Derivatization of Products** .......... S18
**V. Mechanistic Studies** .......... S19
**1. The Radical Trapping Experiments** .......... S19
**2. For the Labeling experiments:** .......... S20
**3. General procedure of the ESR experiments** .......... S20
**4. The Cyclic Voltammetry Experiments** .......... S22
**5. Quenching studies of Ir(ppy)$_3$ with 1a and 2a** .......... S23
**6. Theoretical Calculations** .......... S25
**VI. X-Ray Crystal Structure Determinations** .......... S51
**VII. Characterization Data of the Products** .......... S52
**VIII. References** .......... S74
**NMR Spectra of the Substrates** .......... S75
**NMR Spectra of the Products** .......... S126

# I. General Information

Unless otherwise stated, all reactions were set up in a clear Pyrex glass tube and were stirred with a Teflon-coated magnetic stir bar. Analytical grade solvents and commercially available reagents were used to conduct the reactions. All other reagents were purchased from TCI, Innochem, J&K, Adamas, and Energy chemicals, and were directly used without further purifications. TLC was performed on silica HSGF254 plates and visualized by quenching of UV fluorescence (λmax= 254 nm). Silica gel (200–300 mesh) was purchased from Qingdao Haiyang Chemical Co., China. All reaction glassware was flame- or oven-dried before use. Reactions were monitored by thin-layer chromatography (TLC), and the products were obtained by column chromatography on silica gel.

$^{1}$H NMR, $^{13}$C NMR and $^{19}$F NMR were recorded on a Bruker AM-400 MHz spectrometer (400MHz, 101MHz and 376MHz). $^{1}$H NMR spectra were reported in parts per million (ppm) downfield of tetramethylsilane (TMS) and internally referenced to TMS (0.00 ppm) or the residual protic-solvent peak in $CDCl_3$ (7.26 ppm). $^{13}$C NMR spectra were reported in ppm relative to residual $CHCl_3$ (77.0 ppm). In reporting spectral data the format (δ) chemical shift (multiplicity, *J* values in Hz, integration) was used with the following abbreviations: s = singlet, d = doublet, t = triplet, q = quartet, dd = doublet of doublets, m = multiplet. High-resolution mass spectra (HRMS) were measured on a TOF by ESI and performed on Bruker Daltonics APEXII 47e Specifications. The X-ray single-crystal determination was performed on a Bruker APEX II X-ray single crystal diffractometer. The melting point was measured by WRX® X-4B.

# II. Detailed Reaction Optimizations

### Table S1. Screening of substrates[a]

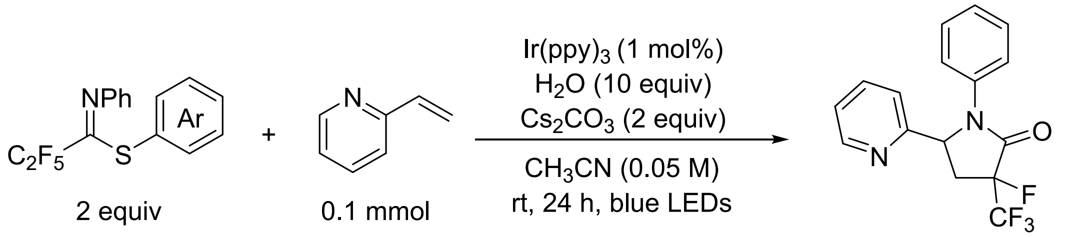


| Substrate | Yield (%)[b] | dr[c] |
|---|---|---|
| 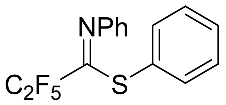 **1a** | 83 | 12:1 |
| 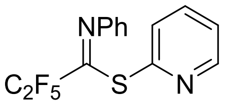 **1v** | 72 | 11:1 |
| 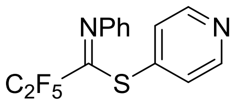 **1w** | 71 | 12:1 |
| 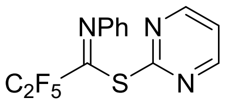 **1x** | 68 | 10:1 |
| 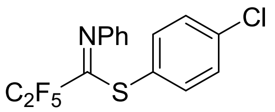 **1y** | 62 | 11:1 |
| 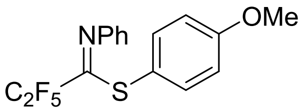 **1z** | 62 | 12:1 |
| 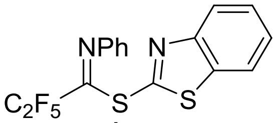 **1aa** | N.D. | |

[a] Reaction conditions: **1** (0.2 mmol), **2a** (0.1 mmol), $Cs_2CO_3$ (0.2 mmol), $H_2O$ (1 mmol), $Ir(ppy)_3$ (0.001 mmol) in 2.0 mL of dry $CH_3CN$ with 6 W blue LED irradiation at rt for 24 h, unless otherwise noted. [b] Isolated yield; N.D. = not detected. [c] Detected by $^{19}$F NMR.

**Table S2. Screening of catalyst screening and control experiments[a]**

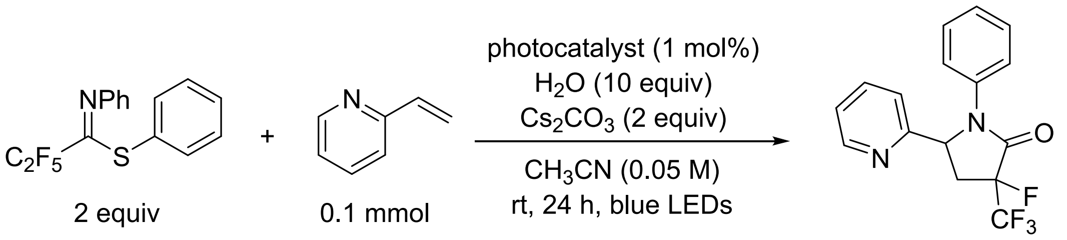


| Entry | PC | Yield (%)[b] | dr[c] |
|---|---|---|---|
| 1 | $Ir(ppy)_3$ | 83 | 12:1 |
| 2 | $[Ir(dtbbpy)(ppy)_2]PF_6$ | N.D | |
| 3 | $[Ir\{dF(CF_3)ppy\}_2(dtbbpy)]PF_6$ | N.D | |
| 4 | $[Ru(bpy)_3]Cl_2$ | N.D | |
| 5 | $Ir(dFppy)_3$ | 68 | 16:1 |
| 6 | $Ir(t\text{-}Buppy)_3$ | 65 | 8:1 |
| 7 | $Mes\text{-}Acr^+ClO_4^-$ | N.D. | |
| 8 | 4-CzIPN | N.D | |
| 9 | Eosin-Y | N.D | |
| 10 | no catalyst | N.D | |
| 11 | no light | N.D | |

[a] Reaction conditions: **1a** (0.2 mmol), **2a** (0.1 mmol), $Cs_2CO_3$ (0.2 mmol), $H_2O$ (1 mmol) and photocatalyst (0.001 mmol) in 2.0 mL of dry $CH_3CN$ with 6 W blue LED irradiation at rt for 24 h, unless otherwise noted. [b] Isolated yield; N.D. = not detected. [c] Detected by $^{19}F$ NMR.

**Table S3. Screening of solvents[a]**

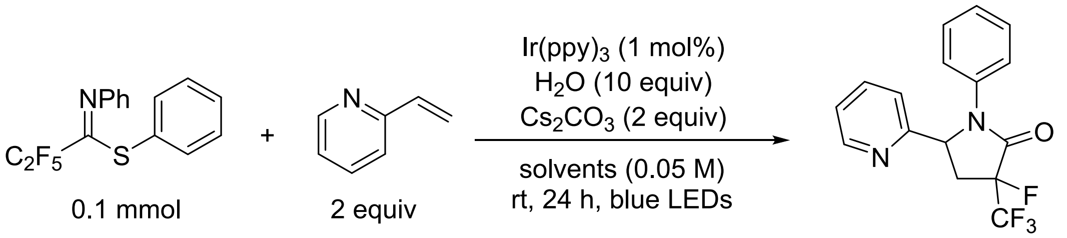


| Entry | solvents | Yield (%)[b] | dr[c] |
|---|---|---|---|
| 1 | $CH_3CN$ | 72 | 12:1 |
| 2 | AcOEt | N.D | |
| 3 | DMF | 67 | 11:1 |
| 4 | DMSO | 45 | 5:1 |
| 5 | TFE | N.D | |
| 6 | DCM | 60 | 17:1 |
| 7 | Acetone | 69 | 10:1 |
| 8 | Dioxane | N.D | |
| 9 | Toluene | N.D | |
| 10 | THF | N.D | |

[a] Reaction conditions: **1a** (0.2 mmol), **2a** (0.1 mmol), $Cs_2CO_3$ (0.2 mmol), $H_2O$ (1 mmol), $Ir(ppy)_3$ (0.001 mmol) in 2.0 mL of dry solvent with 6 W blue LED irradiation at rt for 24 h, unless otherwise noted. [b] Isolated yield; N.D. = not detected. [c] Detected by $^{19}F$ NMR.

## Table S4. Screening of bases[a]

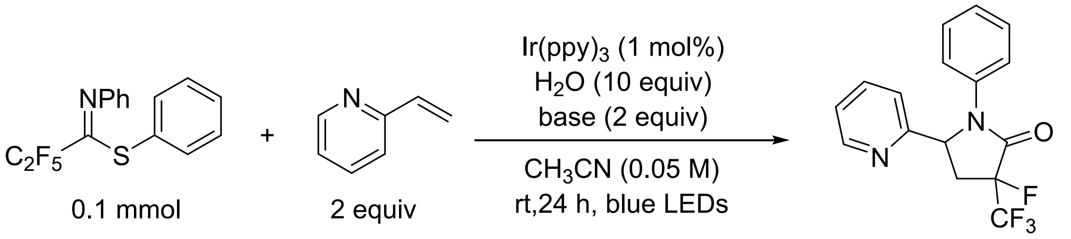


| Entry | bases | Yield (%)[b] | dr[c] |
|---|---|---|---|
| 1 | $Cs_2CO_3$ | 75 | 12:1 |
| 2 | $K_2HPO_4$ | 65 | 11:1 |
| 3 | $Na_2CO_3$ | 67 | 13:1 |
| 4 | $K_2CO_3$ | 67 | 9:1 |
| 5 | $NaHCO_3$ | 54 | 10:1 |
| 6 | $Li_2CO_3$ | 60 | 11:1 |
| 7 | $Na_2HPO_4$ | 63 | 10:1 |
| 8 | NaOH | 66 | 12:1 |
| 9 | KOH | 60 | 11:1 |

[a] Reaction conditions: **1a** (0.2 mmol), **2a** (0.1 mmol), bases (0.2 mmol), $H_2O$ (1 mmol), Ir(ppy)$_3$ (0.001 mmol) in 2.0 mL of dry $CH_3CN$ with 6 W blue LED irradiation at rt for 24 h, unless otherwise noted. [b] Isolated yield; N.D. = not detected. [c] Detected by $^{19}F$ NMR.

## Table S5. Screening of water[a]

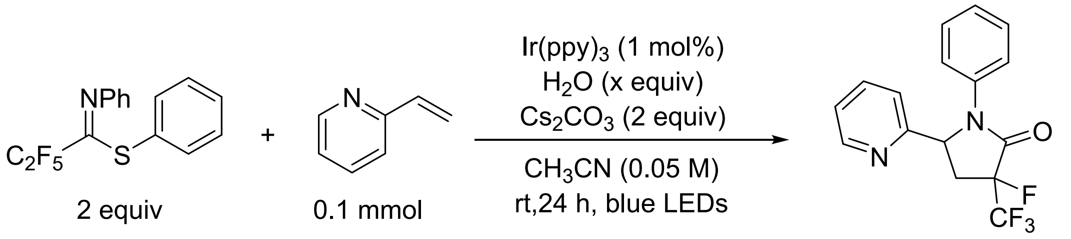


| Entry | $H_2O$ (equiv) | Yield (%)[b] | dr[c] |
|---|---|---|---|
| 1 | 5 | 78 | 9:1 |
| 2 | 10 | 82 | 12:1 |
| 3 | 25 | 69 | 10:1 |
| 4 | 50 | 55 | 9:1 |
| 5 | 100 | 25 | 11:1 |

[a] Reaction conditions: **1a** (0.2 mmol), **2a** (0.1 mmol), $Cs_2CO_3$ (0.2 mmol), $H_2O$ (x equiv), Ir(ppy)$_3$ (0.001 mmol) in 2.0 mL of dry $CH_3CN$ with 6 W blue LED irradiation at rt for 24 h, unless otherwise noted. [b] Isolated yield; N.D. = not detected. [c] Detected by $^{19}F$ NMR.

# III. Gram-Scale Synthesis of 3a

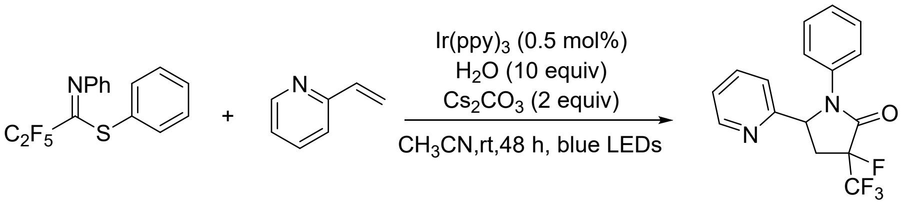


**1a**, 8.0 mmol **2a**, 4.0 mmol **3a**, 0.93g, 72%

To a 100 mL round-bottom flask equipped with a magnetic stir-bar were added phenyl 2,2,3,3,3-pentafluoro-N-phenylpropanimidothioate **1a** (8.0 mmol, 2.0 equiv), 2-vinylpyridine **2a** (4.0 mmol, 1.0 equiv), cesium carbonate (8.0 mmol, 2.0 equiv), and Ir(ppy)$_3$ (0.02 mmol, 0.5 mol%). Then, acetonitrile (40.0 mL) and water (0.72 ml, 10 equiv) were added. The vessel was bubbled with a stream of argon for 20 min via a syringe needle, and the tightly sealed tube was irradiated with a 36 W blue LED at about 20 ℃ for 48 h. The solvent was removed under reduced pressure by a rotary evaporator. Then, the residue was purified by silica gel column chromatography to give the desired product **3a** with a 72% yield.

# IV. General Synthetic Procedures

## 1. General Procedure for Single C(sp$^3$)-F Bond Cleavage

Polyfluorinated Iminosulfide **1** (0.2 mmol, 2.0 equiv), cesium carbonate (0.2 mmol, 2.0 equiv) and Ir(ppy)$_3$ (0.002 mmol, 1.4 mg, 2.0 mol%) were added into a 10 mL of Schlenk tube equipped with a rubber septum and magnetic stirring bar. Then anhydrous MeCN (2 ml, 0.05 M), distilled water (18 ul, 10 equiv) and the alkene (0.1 mmol, 1 equiv) were added sequentially by syringes. The vessel was bubbled with a stream of argon for 20 min via a syringe needle, and the tightly sealed tube was irradiated with 6 W blue LEDs at about 20 ℃. After 24 h, the solvent was removed, and the crude products were purified by column chromatography over silica gel as indicated.

## 2. Synthesis of Alkenylpyridines

**Procedure A:**

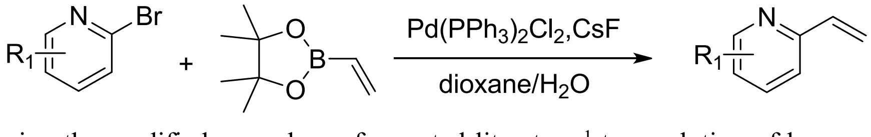


Following the modified procedure of reported literature,[1] to a solution of bromo-substituted azaarene (10.0 mmol, 1.0 equiv.), CsF (4.6 g, 30.0 mmol) and pinacol vinylboronate (2.3 g, 15.0

mmol) in dioxane/$H_2O$ (20 mL/10 mL) was added $Pd(PPh_3)_2Cl_2$ (0.7 g, 1.0 mmol). After stirring for 16 h at 80 °C under Ar, the mixture was concentrated in vacuo. The crude product was further purified by column chromatography (petroleum ether/ethyl acetate = 40/1 - 10/1) on silica gel to yield the corresponding alkenylpyridines.

**Procedure B:**

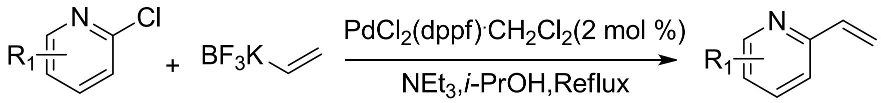


Following the modified procedure of reported literature,[2] a solution of chloro-substituted azaarene (8.0 mmol, 1.0 equiv.), potassium vinyltrifluoroborate (1.29 g, 9.6 mmol), $PdCl_2(dppf)\cdot CH_2Cl_2$ (131 mg, 0.16 mmol), and $Et_3N$ (1.12 mL, 8.0 mmol) in iPrOH (125 mL) was heated to reflux for 16 h. The mixture was cooled to room temperature and partitioned between $CH_2Cl_2$ (100 mL) and $H_2O$ (40 mL). The aqueous layer was separated and extracted with $CH_2Cl_2$ (2 x 50 mL) and the combined organic layers were washed with brine (100 mL), dried (MgSO4), filtered, and concentrated in vacuo. Purification of the residue by column chromatography (petroleum ether/ethyl acetate = 40/1 - 10/1) gave the vinylpyridines.

**Procedure C:**

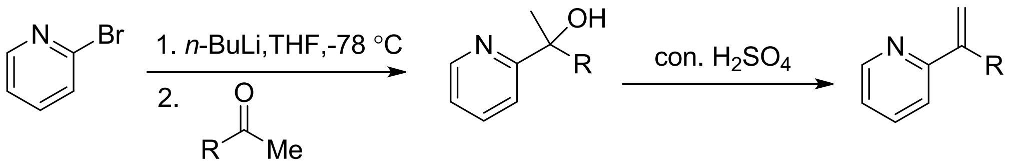


Following the modified procedure of reported literature,[3] a solution of 2-bromopyridine (2.37 g, 1.43 mL, 15.0 mmol, 1.0 equiv.) in THF (19 mL, 0.8 M) was cooled to -78 °C. n-Butyllithium (15.8 mmol, 1.1 equiv.) was then slowly added and the reaction mixture was stirred at -78 °C for 10 min. After that time, the corresponding ketone (15.0 mmol, 1.0 equiv.) was added to the above solution and the reaction mixture was stirred for 15 h while slowly warming up to 23 °C. $NH_4Cl$ (aq) (20 mL) and EtOAc (20 mL) was added, the layers were separated and the aqueous layer was extracted with EtOAc (3 × 20 mL). The organic extracts were combined, dried (MgSO4), filtered, and concentrated in vacuo. The residue was purified by chromatography on silica gel, eluting with hexanes/ EtOAc (9:1 to 4:2 (v/v)), to afford the title compound pyridine alcohol. Concentrated $H_2SO_4$ (5 mL) was added to the above alcohol and stirred at 100 °C for 1 h. Crashed ice was added and neutralized with NaOH solution to PH = 7 - 8. The reaction mixture was extracted with ethyl ether (20 mL×3) then washed with brine and dried over anhydrous sodium sulfate. The solvent was removed by rotary evaporation and purified by silica gel column chromatography to afford the pure products.

## 3. Synthesis of Perfluoroalkyl Group Substituted Iminosulfides

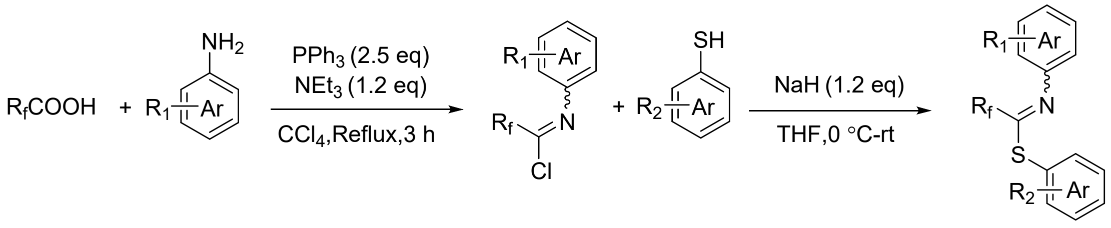


A 200 mL two-necked flask equipped with a septum cap, a condenser, and a Teflon-coated magnetic stir bar was charged with $PPh_3$ (34.5 g, 132 mmol), $Et_3N$ (7.3 mL, 53 mmol), $CCl_4$ (21.1 mL, 220 mmol), and TFA (3.4 mL, 44 mmol). After the solution was stirred for about 10 min (ice bath), an amine (53 mmol) dissolved in $CCl_4$ (21.1 mL, 220 mmol) was added. The mixture was then refluxed with stirring (3 h). After the reaction was completed, residual solid Ph3PO, PPh3, and $Et_3N$-HCl were washed with hexane several times. Then the hexane was filtered and concentrated under vacuum. The crude product was purified by column chromatography on silica gel or neutral alumina to afford the corresponding fluorinated imidoyl chlorides.

To a solution of thiophenol (2.37 g, 1.43 mL, 15.0 mmol, 1.0 equiv.) in THF (19 mL, 0.8 M) was cooled to 0 °C. Sodium hydride (15.8 mmol, 1.1 equiv.) was then slowly added and the reaction mixture was stirred at 0 °C for 10 min. After that time, the corresponding fluorinated imidoyl chlorides (15.0 mmol, 1.0 equiv.) was added to the above solution, and the reaction mixture was stirred for 3 h at room temperature, diluted with diethyl ether (60 mL), and washed with aqueous $NaHCO_3$ (2 × 30 mL) and brine (30 mL). The organic layer was dried over anhydrous $MgSO_4$, filtered, and concentrated under reduced pressure. The crude product was purified by column chromatography on silica gel to afford the corresponding product.

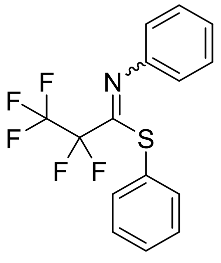


**1a**

Chemical Formula: $C_{15}H_{10}F_5NS$

**phenyl 2,2,3,3,3-pentafluoro-*N*-phenylpropanimidothioate (1a)**

**$^1$H NMR** (400 MHz, Chloroform-*d*) δ (ppm) = 7.30 – 7.25 (m, 2H), 7.24 – 7.12 (m, 5H), 7.07 – 7.00 (m, 1H), 6.81 – 6.74 (m, 2H).

**$^{13}$C NMR** (101 MHz, Chloroform-*d*) δ (ppm) = 151.7 (t, *J* = 29.6 Hz), 146.3, 134.7, 129.3, 129.0, 128.7, 126.9, 125.5, 119.0, 118.4 (qt, *J* = 287.2, 35.7 Hz), 110.2 (tq, *J* = 259.6, 36.3 Hz).

**$^{19}$F NMR** (376 MHz, Chloroform-*d*) δ (ppm) = -80.70 (s, 3F), -111.18 (s, 2F).

**HRMS (ESI) m/z:** $[M + H]^+$ Calcd for $C_{15}H_{11}F_5NS$ 332.0527; Found 332.0526.

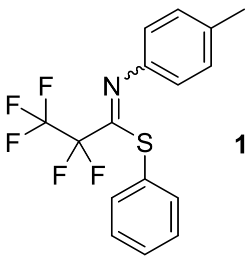

**1b**

Chemical Formula: $C_{16}H_{12}F_5NS$

**phenyl 2,2,3,3,3-pentafluoro-*N*-(p-tolyl)propanimidothioate (1b)**

**$^{1}$H NMR** (400 MHz, Chloroform-*d*) δ (ppm) = 7.31 – 7.27 (m, 2H), 7.26 – 7.21 (m, 1H), 7.20 – 7.14 (m, 2H), 7.02 (d, *J* = 8.2 Hz, 2H), 6.74 (d, *J* = 8.3 Hz, 2H), 2.28 (s, 3H).

**$^{13}$C NMR** (101 MHz, Chloroform-*d*) δ (ppm) = 150.9 (t, *J* = 29.6 Hz), 143.8, 135.5, 134.6, 129.2, 129.1, 128.9, 127.2, 119.4, 118.4 (qt, *J* = 287.4, 35.8 Hz), 110.3 (tq, *J* = 259.7, 36.3 Hz), 20.9.

**$^{19}$F NMR** (376 MHz, Chloroform-*d*) δ (ppm) = -80.67 (s, 3F), -110.62 (s, 2F).

**HRMS (ESI) m/z:** $[M + H]^+$ Calcd for $C_{16}H_{13}F_5NS$ 346.0683; Found 346.0682.

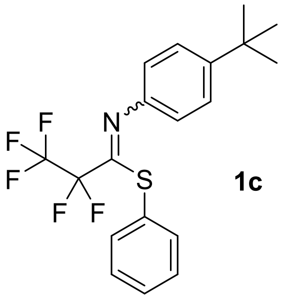

**1c**

Chemical Formula: $C_{19}H_{18}F_5NS$

**phenyl *N*-(4-(tert-butyl)phenyl)-2,2,3,3,3-pentafluoropropanimidothioate (1c)**

**$^{1}$H NMR** (400 MHz, Chloroform-*d*) δ (ppm) = 7.26 – 7.21 (m, 2H), 7.21 – 7.16 (m, 3H), 7.15 – 7.09 (m, 2H), 6.76 – 6.70 (m, 2H), 1.27 (s, 9H).

**$^{13}$C NMR** (101 MHz, Chloroform-*d*) δ (ppm) = 150.5 (t, *J* = 29.8 Hz), 148.7, 143.5, 134.5, 129.1, 128.9, 127.2, 125.4, 119.1, 118.5 (qt, *J* = 287.3, 36.3 Hz), 110.2 (tq, *J* = 259.6, 36.4 Hz), 34.4, 31.3.

**$^{19}$F NMR** (376 MHz, Chloroform-*d*) δ (ppm) = -80.70 (s, 3F), -111.29 (s, 2F).

**HRMS (ESI) m/z:** $[M + H]^+$ Calcd for $C_{19}H_{19}F_5NS$ 388.1153; Found 388.1151.

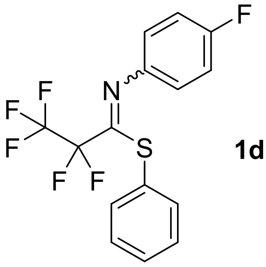

**1d**

Chemical Formula: $C_{15}H_9F_6NS$

**phenyl 2,2,3,3,3-pentafluoro-*N*-(4-fluorophenyl)propanimidothioate (1d)**

**$^{1}$H NMR** (400 MHz, Chloroform-*d*) δ (ppm) = 7.26 – 7.21 (m, 3H), 7.19 – 7.11 (m, 2H), 6.89 – 6.82 (m, 2H), 6.80 – 6.72 (m, 2H).

**$^{13}$C NMR** (101 MHz, Chloroform-*d*) δ (ppm) = 160.5 (d, *J* = 245.4 Hz), 152.0 (t, *J* = 29.5 Hz), 142.2 (d, *J* = 3.1 Hz), 134.4, 129.4, 129.1, 126.8, 121.0 (d, *J* = 8.3 Hz), 118.4 (qt, *J* = 287.3, 35.7 Hz), 115.5 (d, *J* = 22.8 Hz), 110.4 (tq, *J* = 259.6, 36.7 Hz).

**$^{19}$F NMR** (376 MHz, Chloroform-*d*) δ (ppm) = -80.74 (s, 3F), -111.57 (s, 2F), -117.11 (s, 1F).

**HRMS (ESI) m/z:** $[M + H]^+$ Calcd for $C_{15}H_{10}F_6NS$ 350.0433; Found 350.0432.

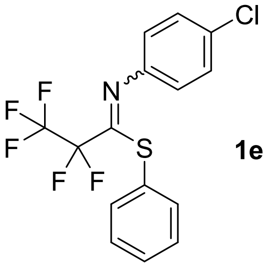


1e

Chemical Formula: $C_{15}H_9ClF_5NS$

**phenyl *N*-(4-chlorophenyl)-2,2,3,3,3-pentafluoropropanimidothioate (1e)**

**$^1$H NMR** (400 MHz, Chloroform-*d*) δ (ppm) = 7.26 – 7.21 (m, 3H), 7.19 – 7.09 (m, 4H), 6.70 – 6.66 (m, 2H).

**$^{13}$C NMR** (101 MHz, Chloroform-*d*) δ (ppm) = 152.8 (t, *J* = 29.2 Hz), 144.7, 134.7, 130.9, 129.5, 129.1, 128.7, 127.5, 120.4, 118.4 (qt, *J* = 287.4, 35.8 Hz), 110.0 (tq, *J* = 259.9, 36.7 Hz).

**$^{19}$F NMR** (376 MHz, Chloroform-*d*) δ (ppm) = -80.75 (s, 3F), -111.73 (s, 2F).

**HRMS (ESI) m/z:** $[M + H]^+$ Calcd for $C_{15}H_{10}ClF_5NS$ 366.0137; Found 366.0136.

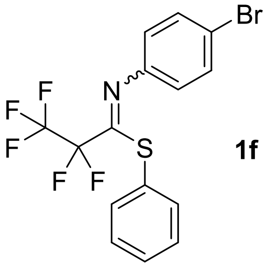


1f

Chemical Formula: $C_{15}H_9BrF_5NS$

**phenyl *N*-(4-bromophenyl)-2,2,3,3,3-pentafluoropropanimidothioate (1f)**

**$^1$H NMR** (400 MHz, Chloroform-*d*) δ (ppm) = 7.28 – 7.20 (m, 5H), 7.19 – 7.13 (m, 2H), 6.64 – 6.59 (m, 2H).

**$^{13}$C NMR** (101 MHz, Chloroform-*d*) δ (ppm) = 152.9 (t, *J* = 29.3 Hz), 145.2, 134.8, 131.7, 129.5, 129.1, 127.5, 120.7, 118.6, 118.3 (qt, *J* = 287.4, 35.7 Hz), 110.0 (tq, *J* = 260.0, 36.8 Hz).

**$^{19}$F NMR** (376 MHz, Chloroform-*d*) δ (ppm) = -80.7 (s, 3F), -111.8 (s, 2F).

**HRMS (ESI) m/z:** $[M + H]^+$ Calcd for $C_{15}H_{10}BrF_5NS$ 409.9632; Found 409.9631.

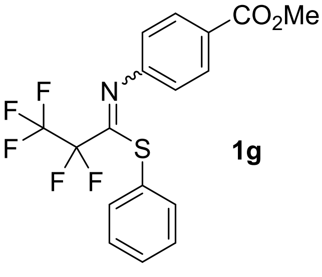


1g

Chemical Formula: $C_{17}H_{12}F_5NO_2S$

**methyl 4-((2,2,3,3,3-pentafluoro-1-(phenylthio)propylidene)amino)benzoate (1g)**

$^{1}$**H NMR** (400 MHz, Chloroform-*d*) δ (ppm) = 7.86 – 7.79 (m, 2H), 7.25 – 7.20 (m, 3H), 7.16 – 7.08 (m, 2H), 6.78 – 6.71 (m, 2H), 3.89 (s, 3H).

$^{13}$**C NMR** (101 MHz, Chloroform-*d*) δ (ppm) = 166.4, 153.4 (t, *J* = 29.3 Hz), 150.2, 135.0, 130.3, 129.7, 129.1, 126.6, 126.2, 118.4, 118.3 (qt, *J* = 287.6, 35.7 Hz), 109.9 (tq, *J* = 261.1, 37.0 Hz), 52.04.

$^{19}$**F NMR** (376 MHz, Chloroform-*d*) δ (ppm) = -80.83 (s, 3F), -112.09 (s, 2F).

**HRMS (ESI) m/z:** $[M + H]^+$ Calcd for $C_{17}H_{13}F_5NO_2S$ 390.0582; Found 390.0581.

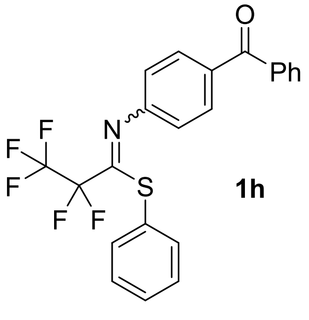


**1h**

Chemical Formula: $C_{22}H_{14}F_5NOS$

**phenyl *N*-(4-benzoylphenyl)-2,2,3,3,3-pentafluoropropanimidothioate (1h)**

$^{1}$**H NMR** (400 MHz, Chloroform-*d*) δ (ppm) = 7.75 – 7.69 (m, 2H), 7.64 – 7.55 (m, 3H), 7.52 – 7.44 (m, 2H), 7.29 – 7.21 (m, 3H), 7.19 – 7.11 (m, 2H), 6.81 – 6.75 (m, 2H).

$^{13}$**C NMR** (101 MHz, Chloroform-*d*) δ (ppm) = 195.4, 153.2 (t, *J* = 29.1 Hz), 149.7, 137.6, 134.9, 134.1, 132.3, 131.0, 129.8, 129.6, 129.2, 128.3, 126.3, 118.3, 118.3 (qt, *J* = 287.7, 35.7 Hz), 109.9 (tq, *J* = 261.2, 37.0 Hz).

$^{19}$**F NMR** (376 MHz, Chloroform-*d*) δ (ppm) = -80.80 (s, 3F), -112.24 (s, 2F).

**HRMS (ESI) m/z:** $[M + H]^+$ Calcd for $C_{22}H_{15}F_5NOS$ 436.0789; Found 436.0785.

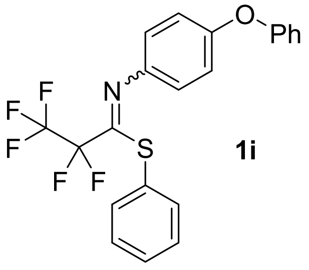


**1i**

Chemical Formula: $C_{21}H_{14}F_5NOS$

**phenyl 2,2,3,3,3-pentafluoro-*N*-(4-phenoxyphenyl)propanimidothioate (1i)**

$^{1}$**H NMR** (400 MHz, Chloroform-*d*) δ (ppm) = 7.37 – 7.30 (m, 2H), 7.29 – 7.16 (m, 5H), 7.10 (td, *J* = 7.5, 1.1 Hz, 1H), 6.95 – 6.89 (m, 2H), 6.87 – 6.79 (m, 4H).

$^{13}$**C NMR** (101 MHz, Chloroform-*d*) δ (ppm) = 157.2, 155.0, 150.5 (t, *J* = 29.3 Hz), 141.3, 134.2, 129.7, 129.1, 129.1, 127.2, 123.3, 121.3, 119.1, 118.6, 118.5 (qt, *J* = 287.4, 35.9 Hz), 110.2 (tq, *J* = 259.5, 36.5 Hz).

$^{19}$**F NMR** (376 MHz, Chloroform-*d*) δ (ppm) = -80.65 (s, 3F), -111.41 (s, 2F).

**HRMS (ESI) m/z:** $[M + H]^+$ Calcd for $C_{21}H_{15}F_5NOS$ 424.0789; Found 424.0788.

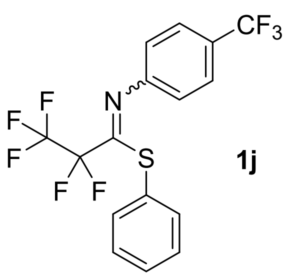


**1j**

Chemical Formula: $C_{16}H_9F_8NS$

**phenyl 2,2,3,3,3-pentafluoro-*N*-(4-(trifluoromethyl)phenyl)propanimidothioate (1j)**

**$^1$H NMR** (400 MHz, Chloroform-*d*) δ (ppm) = 7.35 (d, *J* = 8.3 Hz, 2H), 7.24 – 7.16 (m, 3H), 7.14 – 7.07 (m, 2H), 6.73 (d, *J* = 8.2 Hz, 2H).

**$^{13}$C NMR** (101 MHz, Chloroform-*d*) δ (ppm) = 153.9 (t, *J* = 29.2 Hz), 149.1, 135.0, 129.7, 129.2, 127.0 (q, *J* = 32.6 Hz), 126.2, 125.8 (q, *J* = 3.9 Hz), 124.0 (q, *J* = 271.7 Hz), 118.7, 118.4 (qt, *J* = 287.3, 35.6 Hz), 109.9 (tq, *J* = 260.2, 37.1 Hz).

**$^{19}$F NMR** (376 MHz, Chloroform-*d*) δ (ppm) = -62.39 (s, 3F), -80.87 (s, 3F), -112.57 (s, 2F).

**HRMS (ESI) m/z:** $[M + H]^+$ Calcd for $C_{16}H_{10}F_8NS$ 400.0401; Found 400.0399.

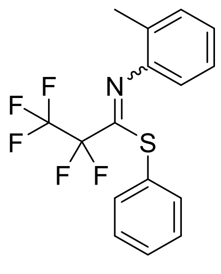


**1k**

Chemical Formula: $C_{16}H_{12}F_5NS$

**phenyl 2,2,3,3,3-pentafluoro-*N*-(o-tolyl)propanimidothioate (1k)**

**$^1$H NMR** (400 MHz, Chloroform-*d*) δ (ppm) = 7.27 – 7.18 (m, 3H), 7.16 – 7.12 (m, 2H), 7.06 – 7.02 (m, 1H), 6.98 – 6.91 (m, 2H), 6.74 (d, *J* = 7.8 Hz, 1H), 1.98 (s, 3H).

**$^{13}$C NMR** (101 MHz, Chloroform-*d*) δ (ppm) = 151.3 (t, *J* = 29.4 Hz), 145.1, 134.7, 130.2, 129.4, 128.9, 127.8, 126.7, 126.0, 125.7, 117.1, 118.5 (qt, *J* = 287.3, 35.3 Hz), 110.0 (tq, *J* = 259.6, 36.9 Hz).

**$^{19}$F NMR** (376 MHz, Chloroform-*d*) δ (ppm) = -80.7 (s, 3F), -111.5 (s, 2F).

**HRMS (ESI) m/z:** $[M + H]^+$ Calcd for $C_{16}H_{13}F_5NS$ 346.0683; Found 346.0684.

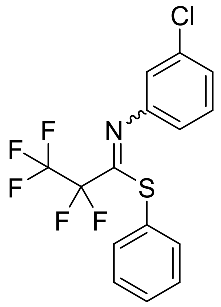


**1l**

Chemical Formula: $C_{15}H_9ClF_5NS$

**phenyl *N*-(3-chlorophenyl)-2,2,3,3,3-pentafluoropropanimidothioate (1l)**

$^{1}$**H NMR** (400 MHz, Chloroform-*d*) δ (ppm) = 7.26 – 7.20 (m, 3H), 7.18 – 7.13 (m, 2H), 7.07 (t, *J* = 8.0 Hz, 1H), 6.94 (ddd, *J* = 8.0, 2.0, 1.0 Hz, 1H), 6.68 (t, *J* = 2.0 Hz, 1H), 6.61 (ddd, *J* = 7.9, 2.0, 1.0 Hz, 1H).

$^{13}$**C NMR** (101 MHz, Chloroform-*d*) δ (ppm) = 153.3 (t, *J* = 29.0 Hz), 147.1, 134.8, 134.4, 129.7, 129.6, 129.1, 126.2, 125.2, 119.0, 118.4 (qt, *J* = 287.3, 35.6 Hz), 117.1, 110.0 (tq, *J* = 260.0, 36.8 Hz).

$^{19}$**F NMR** (376 MHz, Chloroform-*d*) δ (ppm) = -80.80 (s, 3F), -112.11 (s, 2F).

**HRMS (ESI) m/z:** $[M + H]^+$ Calcd for $C_{15}H_{10}ClF_5NS$ 366.0137; Found 366.0136.

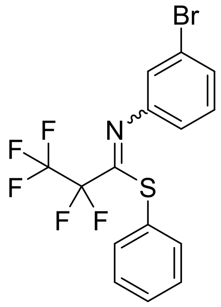


**1m**

Chemical Formula: $C_{15}H_9BrF_5NS$

**phenyl *N*-(3-bromophenyl)-2,2,3,3,3-pentafluoropropanimidothioate (1m)**

$^{1}$**H NMR** (400 MHz, Chloroform-*d*) δ (ppm) = 7.23 (dtd, *J* = 6.9, 3.2, 1.2 Hz, 3H), 7.18 – 7.13 (m, 2H), 7.09 (ddd, *J* = 8.0, 1.9, 1.1 Hz, 1H), 7.00 (t, *J* = 7.9 Hz, 1H), 6.83 (t, *J* = 1.9 Hz, 1H), 6.66 (ddd, *J* = 7.9, 2.0, 1.1 Hz, 1H).

$^{13}$**C NMR** (101 MHz, Chloroform-*d*) δ (ppm) = 153.3 (t, *J* = 29.2 Hz), 147.1, 134.8, 130.0, 129.6, 129.1, 128.0, 126.1, 122.4, 121.8, 118.3 (qt, *J* = 287.4, 35.7 Hz), 117.5, 110.4 (tq, *J* = 260.0, 36.9 Hz).

$^{19}$**F NMR** (376 MHz, Chloroform-*d*) δ (ppm) = -80.80 (s, 3F), -112.21 (s, 2F).

**HRMS (ESI) m/z:** $[M + H]^+$ Calcd for $C_{15}H_{10}BrF_5NS$ 409.9632; Found 409.9633.

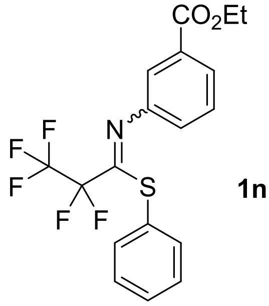


**1n**

Chemical Formula: $C_{18}H_{14}F_5NO_2S$

**ethyl 3-((2,2,3,3,3-pentafluoro-1-(phenylthio)propylidene)amino)benzoate (1n)**

$^{1}$**H NMR** (400 MHz, Chloroform-*d*) δ (ppm) = 7.68 (dt, *J* = 7.9, 1.3 Hz, 1H), 7.36 (t, *J* = 1.8 Hz, 1H), 7.27 – 7.21 (m, 3H), 7.20 – 7.09 (m, 3H), 6.93 (ddd, *J* = 7.9, 2.2, 1.1 Hz, 1H), 4.37 (q, *J* = 7.1 Hz, 2H), 1.41 (t, *J* = 7.2 Hz, 3H).

$^{13}$**C NMR** (101 MHz, Chloroform-*d*) δ (ppm) = 165.8, 153.0 (t, *J* = 29.3 Hz), 146.1, 134.7, 131.0, 129.4, 129.1, 128.7, 126.4, 126.3, 123.2, 112.0, 118.5 (qt, *J* = 288.6, 35.4 Hz), 110.4 (tq, *J* = 260.3, 36.3 Hz).

$^{19}$**F NMR** (376 MHz, Chloroform-*d*) δ (ppm) = -80.74 (s, 3F), -111.91 (s, 2F).

**HRMS (ESI) m/z:** $[M + H]^+$ Calcd for $C_{18}H_{15}F_5NO_2S$ 404.0738; Found 404.0735.

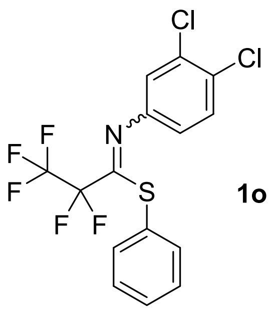

Chemical Formula: $C_{15}H_8Cl_2F_5NS$

**phenyl *N*-(3,4-dichlorophenyl)-2,2,3,3,3-pentafluoropropanimidothioate (1o)**

**$^{1}$H NMR** (400 MHz, Chloroform-*d*) δ (ppm) = 7.29 – 7.14 (m, 6H), 6.75 (d, *J* = 2.4 Hz, 1H), 6.57 (dd, *J* = 8.5, 2.4 Hz, 1H).

**$^{13}$C NMR** (101 MHz, Chloroform-*d*) δ (ppm) = 154.1 (t, *J* = 29.0 Hz), 145.2, 134.8, 132.5, 130.2, 129.7, 129.2, 128.7, 126.0, 120.8, 118.5, 118.3 (qt, *J* = 287.5, 35.6 Hz), 109.8 (tq, *J* = 261.2, 37.1 Hz).

**$^{19}$F NMR** (376 MHz, Chloroform-*d*) δ (ppm) = -80.85 (s, 3F), -112.48 (s, 2F).

**HRMS (ESI) m/z:** $[M + H]^+$ Calcd for $C_{15}H_9C_{12}F_5NS$ 399.9747; Found 399.9745.

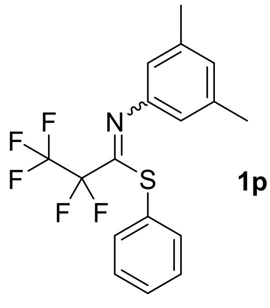

Chemical Formula: $C_{17}H_{14}F_5NS$

**phenyl *N*-(3,5-dimethylphenyl)-2,2,3,3,3-pentafluoropropanimidothioate (1p)**

**$^{1}$H NMR** (400 MHz, Chloroform-*d*) δ (ppm) = 7.29 – 7.24 (m, 2H), 7.22 – 7.12 (m, 3H), 6.64 (s, 1H), 6.37 (s, 2H), 2.20 (s, 6H).

**$^{13}$C NMR** (101 MHz, Chloroform-*d*) δ (ppm) = 150.9 (t, *J* = 29.6 Hz), 146.2, 138.4, 134.7, 129.1, 128.8, 127.1, 127.0, 118.5 (qt, *J* = 287.4, 35.9 Hz), 116.6, 110.2 (tq, *J* = 259.7, 36.4 Hz).

**$^{19}$F NMR** (376 MHz, Chloroform-*d*) δ (ppm) = -80.71 (s, 3F), -111.14 (s, 2F).

**HRMS (ESI) m/z:** $[M + H]^+$ Calcd for $C_{17}H_{15}F_5NS$ 360.0840; Found 360.0837.

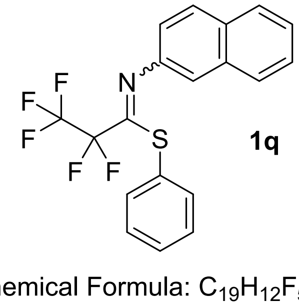

Chemical Formula: $C_{19}H_{12}F_5NS$

**phenyl 2,2,3,3,3-pentafluoro-*N*-(naphthalen-2-yl)propanimidothioate (1q)**

**$^{1}$H NMR** (400 MHz, Chloroform-*d*) δ (ppm) = 7.75 – 7.61 (m, 3H), 7.47 – 7.36 (m, 2H), 7.25 – 7.19 (m, 2H), 7.17 (d, *J* = 2.0 Hz, 1H), 7.09 – 6.94 (m, 4H).

**$^{13}$C NMR** (101 MHz, Chloroform-*d*) δ (ppm) = 152.0 (t, $J$ = 29.5 Hz), 143.8, 134.7, 133.2, 131.3, 129.2, 128.8, 128.7, 127.7, 127.6, 126.7, 126.5, 125.5, 119.2, 118.5 (qt, $J$ = 287.3, 35.7 Hz), 116.0, 110.2 (tq, $J$ = 259.8, 36.6 Hz).

**$^{19}$F NMR** (376 MHz, Chloroform-*d*) δ (ppm) = -80.64 (s, 3F), -111.14 (s, 2F).

**HRMS (ESI) m/z:** $[M + H]^+$ Calcd for $C_{19}H_{13}F_5NS$ 382.0683; Found 382.0682.

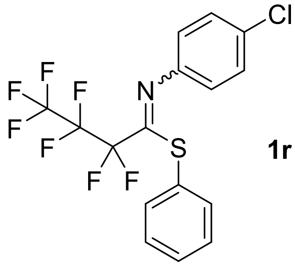


**1r**

Chemical Formula: $C_{16}H_9ClF_7NS$

**phenyl *N*-(4-chlorophenyl)-2,2,3,3,4,4,4-heptafluorobutanimidothioate (1r)**

**$^{1}$H NMR** (400 MHz, Chloroform-*d*) δ (ppm) = 7.24 – 7.19 (m, 1H), 7.18 – 7.09 (m, 4H), 7.08 – 7.01 (m, 2H), 6.66 – 6.58 (m, 2H).

**$^{13}$C NMR** (101 MHz, Chloroform-*d*) δ (ppm) = 152.4 (t, $J$ = 27.7 Hz), 144.6, 134.3, 130.7, 129.3, 129.1, 128.6, 126.7, 120.2. carbons corresponding to the $C_3F_7$ group cannot be identified due to C-F coupling.

**$^{19}$F NMR** (376 MHz, Chloroform-*d*) δ (ppm) = -79.87 (t, $J$ = 9.3 Hz, 3F), -109.77 (q, $J$ = 9.4 Hz, 2F), -123.50 – -124.37 (m, 2F).

**HRMS (ESI) m/z:** $[M + H]^+$ Calcd for $C_{16}H_{10}ClF_7NS$ 416.0105; Found 416.0104.

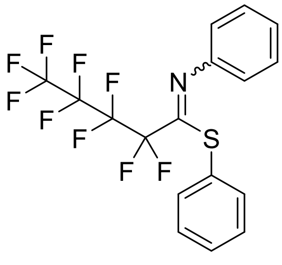


**1s**

Chemical Formula: $C_{17}H_{10}F_9NS$

**phenyl 2,2,3,3,4,4,5,5,5-nonafluoro-*N*-phenylpentanimidothioate (1s)**

**$^{1}$H NMR** (400 MHz, Chloroform-*d*) δ (ppm) = 7.21 – 7.07 (m, 7H), 7.01 – 6.95 (m, 1H), 6.75 – 6.69 (m, 2H).

**$^{13}$C NMR** (101 MHz, Chloroform-*d*) δ (ppm) = 151.4 (t, $J$ = 28.0 Hz), 146.1, 134.3, 129.1, 129.0, 128.6, 127.1, 125.4, 118.9. carbons corresponding to the $C_4F_9$ group cannot be identified due to C-F coupling.

**$^{19}$F NMR** (376 MHz, Chloroform-*d*) δ (ppm) = -80.94 (t, $J$ = 9.8 Hz, 3F), -108.53 (t, $J$ = 12.8 Hz, 2F), -120.66 (dddt, $J$ = 13.3, 9.9, 6.5, 3.5 Hz, 2F), -124.77 (tt, $J$ = 12.9, 4.1 Hz, 2F).

**HRMS (ESI) m/z:** $[M + H]^+$ Calcd for $C_{17}H_{11}F_9NS$ 432.0463; Found 432.0460.

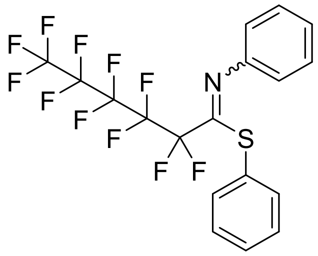


**1t**

Chemical Formula: $C_{18}H_{10}F_{11}NS$

**phenyl 2,2,3,3,4,4,5,5,6,6,6-undecafluoro-*N*-phenylhexanimidothioate (1t)**

**$^{1}$H NMR** (400 MHz, Chloroform-*d*) δ (ppm) = 7.20 – 7.05 (m, 7H), 7.00 – 6.94 (m, 1H), 6.71 (d, *J* = 7.7 Hz, 2H).

**$^{13}$C NMR** (101 MHz, Chloroform-*d*) δ (ppm) = 151.4 (t, *J* = 27.9 Hz), 146.2, 134.2, 129.1, 129.0, 128.6, 127.1, 125.3, 118.9. carbons corresponding to the $C_5F_{11}$ group cannot be identified due to C-F coupling.

**$^{19}$F NMR** (376 MHz, Chloroform-*d*) δ (ppm) = -80.76 (t, *J* = 10.3 Hz, 3F), -108.38 (t, *J* = 13.9 Hz, 2F), -119.99 (p, *J* = 8.4 Hz, 2F), -121.49 – -121.65 (m, 2F), -126.19 (tt, *J* = 13.7, 3.9 Hz, 2F).

**HRMS (ESI) m/z:** $[M + H]^+$ Calcd for $C_{18}H_{10}F_{11}NS$ 482.0431; Found 482.0428.

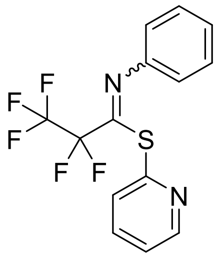


**1v**

Chemical Formula: $C_{14}H_9F_5N_2S$

**pyridin-2-yl 2,2,3,3,3-pentafluoro-*N*-phenylpropanimidothioate (1v)**

**$^{1}$H NMR** (400 MHz, Chloroform-*d*) δ (ppm) = 8.36 (ddd, *J* = 4.9, 2.0, 0.9 Hz, 1H), 7.41 (td, *J* = 7.7, 1.9 Hz, 1H), 7.22 – 7.16 (m, 2H), 7.13 (dt, *J* = 7.9, 1.0 Hz, 1H), 7.07 – 7.00 (m, 2H), 6.91 – 6.85 (m, 2H).

**$^{13}$C NMR** (101 MHz, Chloroform-*d*) δ (ppm) = 152.1, 150.2, 148.6 (t, *J* = 29.9 Hz), 136.8, 128.6, 126.9, 126.0, 122.3, 119.3, 118.5 (qt, *J* = 287.5, 36.0 Hz), 109.7 (tq, *J* = 259.5, 36.8 Hz).

**$^{19}$F NMR** (376 MHz, Chloroform-*d*) δ (ppm) = -80.91 (s, 3F), -112.59 (s, 2F).

**HRMS (ESI) m/z:** $[M + H]^+$ Calcd for $C_{14}H_{10}F_5N_2S$ 333.0479; Found 333.0478.

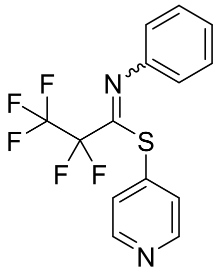


**1w**

Chemical Formula: $C_{14}H_9F_5N_2S$

**pyridin-4-yl 2,2,3,3,3-pentafluoro-*N*-phenylpropanimidothioate (1w)**

**$^{1}$H NMR** (400 MHz, Chloroform-*d*) δ (ppm) = 8.38 – 8.31 (m, 2H), 7.23 – 7.16 (m, 2H), 7.10 – 7.03 (m, 3H), 6.86 – 6.80 (m, 2H).

**¹³C NMR** (101 MHz, Chloroform-*d*) δ (ppm) = 149.9, 148.1 (t, *J* = 30.0 Hz), 145.7, 138.6, 128.8, 126.9, 126.4, 119.3, 118.3 (qt, *J* = 287.4, 35.8 Hz), 109.4 (tq, *J* = 259.3, 37 Hz).
**¹⁹F NMR** (376 MHz, Chloroform-*d*) δ (ppm) = -80.74 (s, 3F), -112.04 (s, 2F).
**HRMS (ESI) m/z:** $[M + H]^+$ Calcd for $C_{14}H_{10}F_5N_2S$ 333.0479; Found 333.0478.

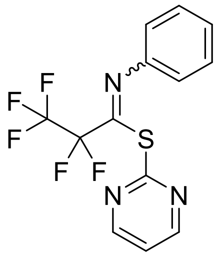


**1x**

Chemical Formula: $C_{13}H_8F_5N_3S$

**pyrimidin-2-yl 2,2,3,3,3-pentafluoro-*N*-phenylpropanimidothioate (1x)**
**¹H NMR** (400 MHz, Chloroform-*d*) δ (ppm) = 8.45 (d, *J* = 4.9 Hz, 2H), 7.30 – 7.25 (m, 2H), 7.16 – 7.11 (m, 1H), 7.03 – 6.96 (m, 3H).
**¹³C NMR** (101 MHz, Chloroform-*d*) δ (ppm) = 167.8, 157.8, 146.6, 145.9 (t, *J* = 30.2 Hz), 128.7, 126.6, 119.5, 118.6 (qt, *J* = 287.6, 36.3 Hz), 118.3, 109.3 (tq, *J* = 259.1, 36.8 Hz).
**¹⁹F NMR** (376 MHz, Chloroform-*d*) δ (ppm) = -81.06 (s, 3F), -113.32 (d, *J* = 2.0 Hz, 2F).
**HRMS (ESI) m/z:** $[M + H]^+$ Calcd for $C_{13}H_9F_5N_3S$ 334.0432; Found 334.0430.

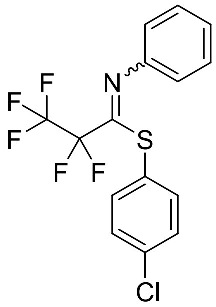


**1y**

Chemical Formula: $C_{15}H_9ClF_5NS$

**4-chlorophenyl 2,2,3,3,3-pentafluoro-*N*-phenylpropanimidothioate (1y)**
**¹H NMR** (400 MHz, Chloroform-*d*) δ (ppm) = 7.26 – 7.16 (m, 4H), 7.15 – 7.05 (m, 3H), 6.78 – 6.73 (m, 2H).
**¹³C NMR** (101 MHz, Chloroform-*d*) δ (ppm) = 151.2 (t, *J* = 29.7 Hz), 146.2, 136.0, 135.9, 129.2, 128.8, 125.6, 125.2, 118.9, 118.3 (qt, *J* = 287.2, 35.6 Hz), 110.1 (tq, *J* = 259.7, 36.7 Hz).
**¹⁹F NMR** (376 MHz, Chloroform-*d*) δ (ppm) = -80.70 (s, 3F), -111.21 (s, 2F).
**HRMS (ESI) m/z:** $[M + H]^+$ Calcd for $C_{15}H_{10}ClF_5NS$ 336.0137; Found 336.0135.

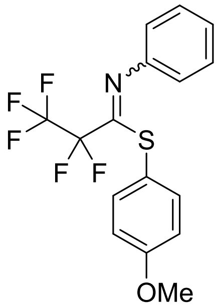


**1z**

Chemical Formula: $C_{16}H_{12}F_5NOS$

**4-methoxyphenyl 2,2,3,3,3-pentafluoro-*N*-phenylpropanimidothioate (1z)**

**$^1$H NMR** (400 MHz, Chloroform-*d*) δ (ppm) = 7.26 – 7.19 (m, 4H), 7.09 – 7.03 (m, 1H), 6.81 – 6.76 (m, 2H), 6.73 – 6.67 (m, 2H), 3.75 (s, 3H).

**$^{13}$C NMR** (101 MHz, Chloroform-*d*) δ (ppm) = 159.7, 151.9 (t, *J* = 29.6 Hz), 145.4, 135.9, 127.7, 124.3, 117.9, 117.4 (qt, *J* = 287.4, 35.6 Hz), 109.4 (tq, *J* = 260.0, 36.4 Hz), 115.9, 113.6, 54.3.

**$^{19}$F NMR** (376 MHz, Chloroform-*d*) δ (ppm) = -80.67 (s, 3F), -110.84 (s, 2F).

**HRMS (ESI) m/z:** $[M + H]^+$ Calcd for $C_{16}H_{13}F_5NOS$ 362.0633; Found 362.0631.

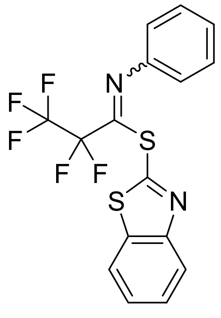


**1aa**

Chemical Formula: $C_{16}H_9F_5N_2S_2$

**benzo[d]thiazol-2-yl 2,2,3,3,3-pentafluoro-*N*-phenylpropanimidothioate (1aa)**

**$^1$H NMR** (400 MHz, Chloroform-*d*) δ (ppm) = 7.91 (dd, *J* = 8.2, 0.9 Hz, 1H), 7.75 – 7.70 (m, 1H), 7.45 (ddd, *J* = 8.3, 7.2, 1.3 Hz, 1H), 7.37 (ddd, *J* = 8.3, 7.2, 1.2 Hz, 1H), 7.20 – 7.14 (m, 2H), 7.04 – 6.98 (m, 1H), 6.97 – 6.91 (m, 2H).

**$^{13}$C NMR** (101 MHz, Chloroform-*d*) δ (ppm) = 155.3, 152.8, 145.8 (t, *J* = 30.7 Hz), 145.8, 137.4, 128.7, 126.5, 125.9, 123.3, 121.1, 119.2, 118.3 (qt, *J* = 287.6, 35.8 Hz), 109.6 (tq, *J* = 260.2, 37.3 Hz).

**$^{19}$F NMR** (376 MHz, Chloroform-*d*) δ (ppm) = -80.84 (s, 3F), -112.01 (s, 2F).

**HRMS (ESI) m/z:** $[M + H]^+$ Calcd for $C_{16}H_{10}F_5N_2S_2$ 389.0200; Found 389.0199.

## 4. Derivatization of Products

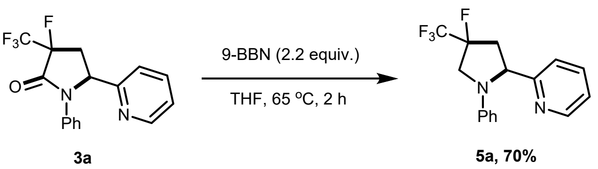


To a reaction vial was added **3a** (33.0 mg, 0.1 mmol, 1.0 equiv.) under $N_2$. Subsequently, 9-BBN (0.5 M in THF, 0.5 mL, 2.2 equiv.) was added via syringe, and the reaction mixture was stirred at 65 °C for 2 hours. After completion, 1 M HCl and EtOAc were added. The two-phase mixture was separated, and then the aqueous phase was basified to pH > 10 with $Na_2CO_3$ and extracted with EtOAc. The resulting organic layer was concentrated under reduced pressure. The residue was purified by column chromatography on silica gel to afford the desired product **5a** (21.6 mg, 70%).

**$^1$H NMR** (400 MHz, Chloroform-*d*) δ (ppm) = 8.64 – 8.58 (m, 1H), 7.59 (td, *J* = 7.7, 1.8 Hz, 1H), 7.30 – 7.14 (m, 4H), 6.83 – 6.75 (m, 1H), 6.51 (dd, *J* = 8.8, 1.1 Hz, 2H), 5.13 (dd, *J* = 9.7, 1.8 Hz, 1H), 4.16 – 4.03 (m, 1H), 4.02 – 3.88 (m, 1H), 3.08 – 2.88 (m, 1H), 2.75 – 2.60 (m, 1H).

$^{13}$**C NMR** (101 MHz, Chloroform-*d*) δ (ppm) = 161.5, 149.6, 145.6, 137.0, 129.4, 122.3, 120.3, 118.0, 112.6, 63.9, 54.6 (d, $J$ = 24.2 Hz), 38.7 (d, $J$ = 21.0 Hz).
$^{19}$**F NMR** (376 MHz, Chloroform-*d*) δ (ppm) = -80.34 (d, $J$ = 6.2 Hz, 3F), -162.44 (q, $J$ = 6.4 Hz, 1F).
**HRMS (ESI) m/z:** $[M + H]^+$ Calcd for $C_{16}H_{15}F_4N_2$ 311.1166; Found 311.1164.

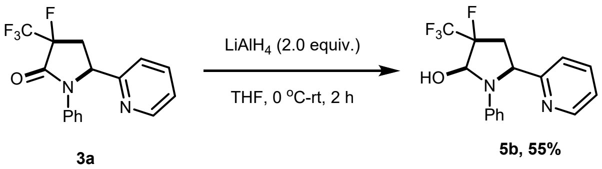


To a solution of **3a** (33.0 mg, 0.1 mmol, 1.0 equiv.) in THF (1.0 mL, 0.1 M) was slowly added lithium aluminum hydride (8.0 mg, 0.2 mmol, 2.0 equiv.), and the reaction mixture was stirred at r.t. for 2 hours. When the reaction was completed, the reaction was quenched with 1 M NaOH, $H_2O$ and extracted with EtOAc. The separated organic layer was washed twice with water and once with brine, then dried over anhydrous $Na_2SO_4$, the resulting organic layer was concentrated under reduced pressure, and the crude product was purified by chromatography on silica gel to afford the desired product **5b** (18.0 mg, 55%).
$^{1}$**H NMR** (400 MHz, Chloroform-*d*) δ (ppm) = 8.49 (ddd, $J$ = 4.9, 1.8, 0.9 Hz, 1H), 7.72 (td, $J$ = 7.6, 1.8 Hz, 1H), 7.43 (d, $J$ = 7.7 Hz, 1H), 7.24 – 7.11 (m, 3H), 6.84 (d, $J$ = 7.7 Hz, 2H), 6.80 – 6.74 (m, 1H), 6.55 (d, $J$ = 9.9 Hz, 1H), 5.75 (d, $J$ = 9.9 Hz, 1H), 5.17 (dt, $J$ = 8.9, 3.2 Hz, 1H), 3.07 (td, $J$ = 14.7, 8.9 Hz, 1H), 2.51 – 2.36 (m, 1H).
$^{13}$**C NMR** (101 MHz, Chloroform-*d*) δ (ppm) = 159.8, 149.9, 142.8, 137.6, 129.2, 123.0, 122.5, 119.3, 114.2, 80.9 (d, $J$ = 20.3 Hz), 61.0, 37.0 (d, $J$ = 22.7 Hz).
$^{19}$**F NMR** (376 MHz, Chloroform-*d*) δ (ppm) = -80.30 (d, $J$ = 9.5 Hz, 3F), -172.66 (q, $J$ = 9.2 Hz, 1F).
**HRMS (ESI) m/z:** $[M + H]^+$ Calcd for $C_{16}H_{15}F_4N_2O$ 327.1115; Found 327.1113.

# V. Mechanistic Studies

## 1. The Radical Trapping Experiments

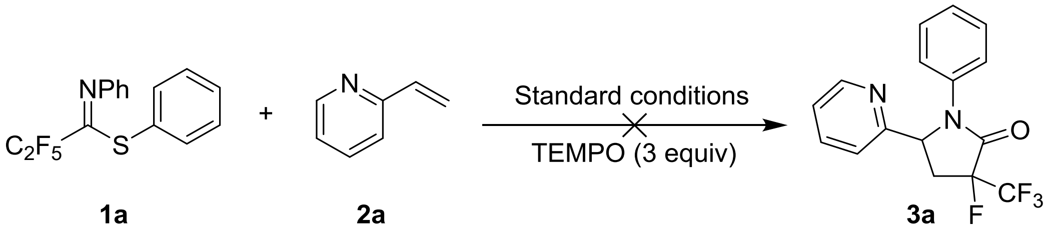


To a 10 mL pyrex glass tube which equipped with a magnetic stirring bar were added **1a** (0.2 mmol), **2a** (0.1 mmol), $Cs_2CO_3$ (0.2 mmol), $H_2O$ (10 equiv), $Ir(ppy)_3$ (0.001 mmol) followed by TEMPO (3.0 equiv) in dry $CH_3CN$ (2 mL). The reaction mixture was stirred at rt and irradiated by

6W blue LEDs for 24 hours. The reaction was monitored by TLC. No desired product **3a** was observed.

## 2. For the Labeling experiments:

Reaction conditions: **1a** (0.2 mmol), **2a** (0.1 mmol), $Cs_2CO_3$ (0.2 mmol), $H_2{}^{18}O$ (1 mmol), $Ir(ppy)_3$ (0.001 mmol), 6 W blue LEDs, rt, 24 h. Isolated yields.
The $^{18}O$ was determined by HRMS.

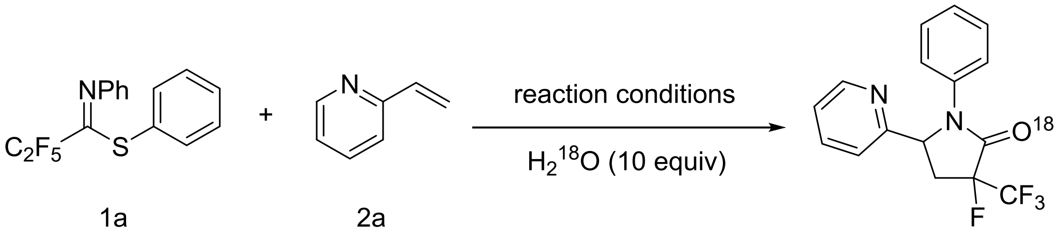


(M+H)$^+$ Calcd: 327.1002
Found: 327.0995

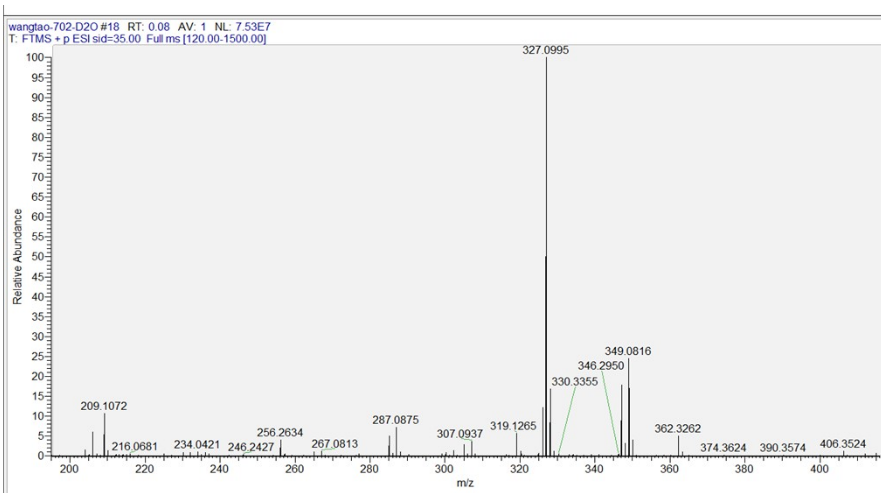


## 3. General procedure of the ESR experiments

EPR spectra were recorded at 300K using a Bruker A300 EPR spectrometer at 9.4447 GHz. The EPR parameters were set as the following: sweep width 120 G, center field 3362 G, time constant 81.92 ms, sweep time 60.00 s, microwave power 23.49 mW, modulation amplitude 1.00 G, modulation frequency 100 kHz, and receiver gain $1.00\times10^4$.

To gain more insight into the possible radical intermediates, EPR trapping experiments were conducted in the presence of a spin trapping agent, 5,5-dimethyl-1-pyrroline-N-oxide (DMPO) (33.9 mg). As a result, the formation of the DMPO spin adduct of radical was observed ($\alpha_N$ = 13.61 G, $\alpha_H$ = 24.18 G, and g = 2.0069) by EPR and HRMS (ESI+) (Figure S1).

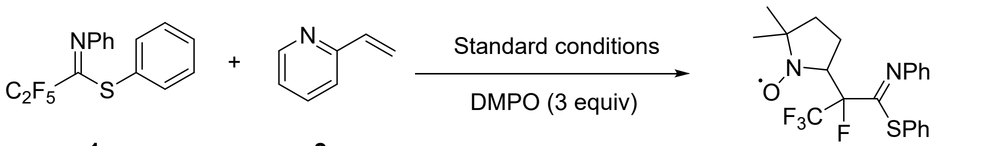


**1a** **2a**

determined by HRMS and EPR
calcd m/z for $[C_{21}H_{21}F_4N_2OS]^+$ 425.1305
Found: 425.1309

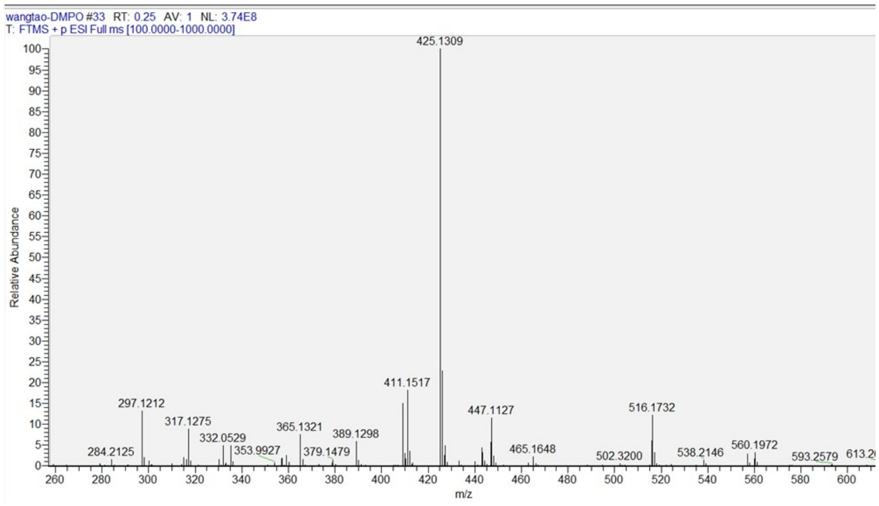


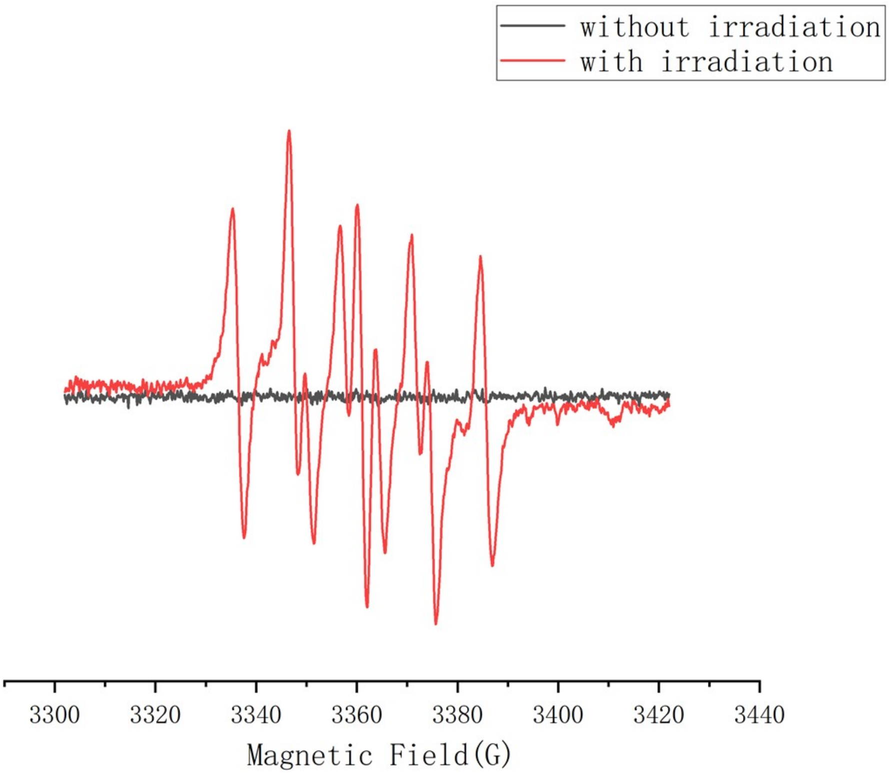


**Figure S1**. EPR spectra in the absence and presence of irradiation.

## 4. The Cyclic Voltammetry Experiments

Cyclovoltammetry experiments were carried out on a CH electrochemical workstation using a glassy carbon working electrode, Hg/$HgCl_2$ reference electrode, and Pt wire counter electrode. The measurements were taken at room temperature in dry $CH_3CN$ containing 0.1 M $Bu_4NPF_6$ as electrolyte. Scan Rate (V/s) = 0.05.

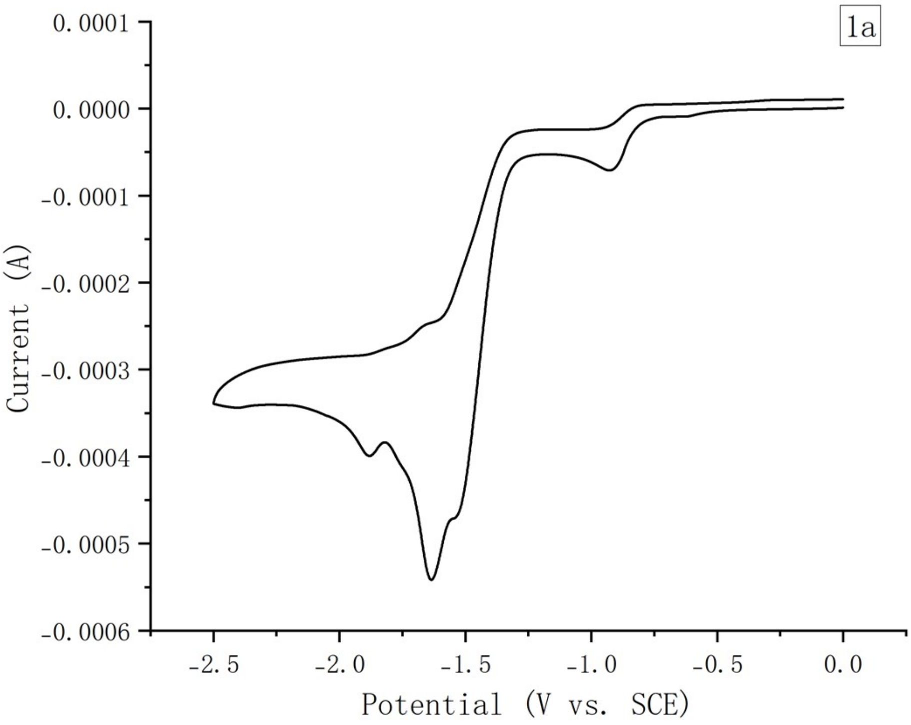


**Figure S2**. CV of **1a**

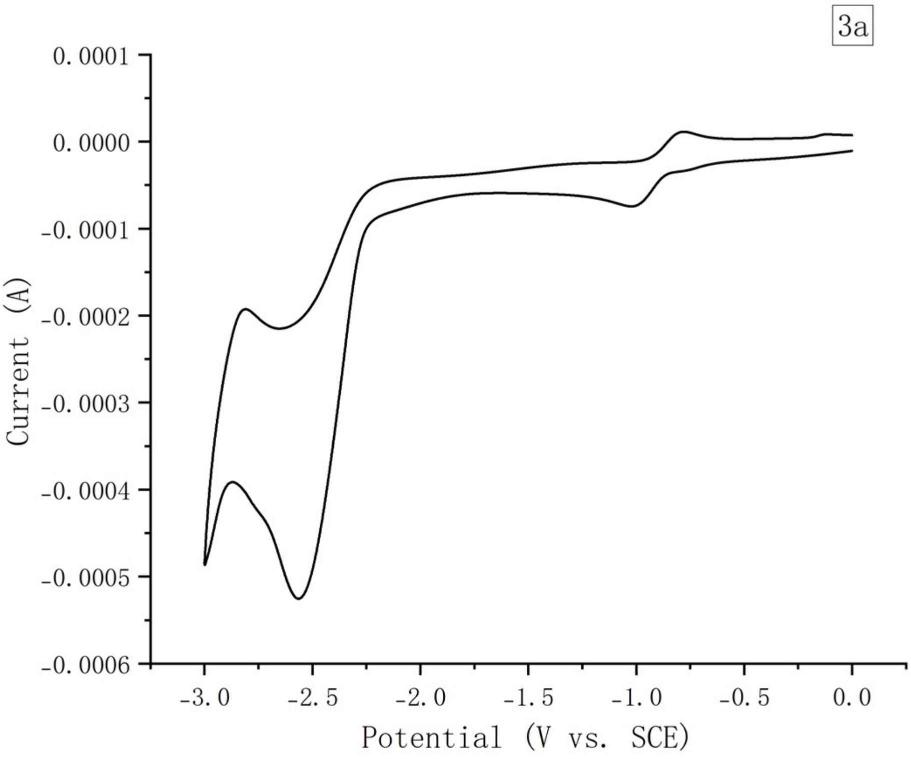


**Figure S3**. CV of **3a**

# 5. Quenching studies of Ir(ppy)$_3$ with 1a and 2a

Fluorescence quenching experiments were performed for a combination of Ir(ppy)$_3$ with **1a** or **2a** involved in the model reaction, and the results are depicted in respectively Figure S4 and Figure S5.

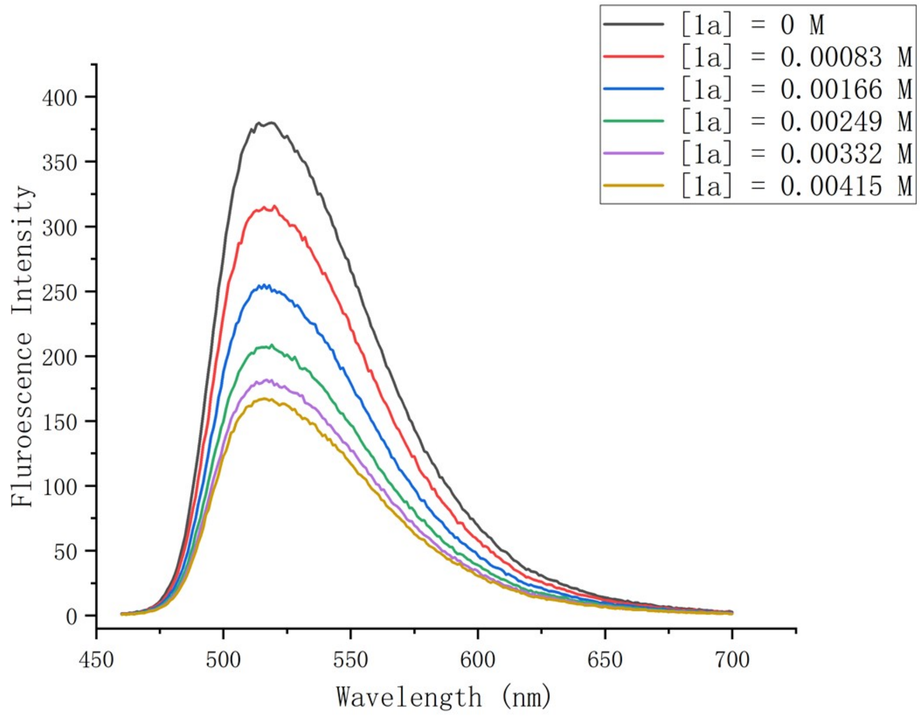


**Figure S4**. Fluorescence emission spectra of $Ir(ppy)_3$ in acetonitrile in the absence and presence of **1a**.

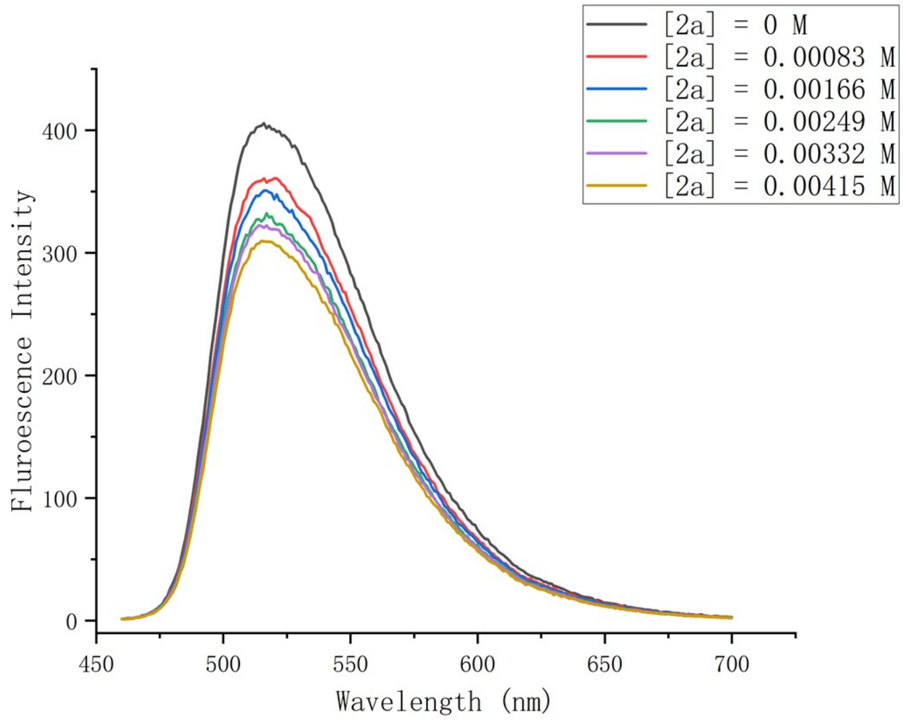


**Figure S5**. Fluorescence emission spectra of $Ir(ppy)_3$ in acetonitrile in the absence and presence of **2a**.

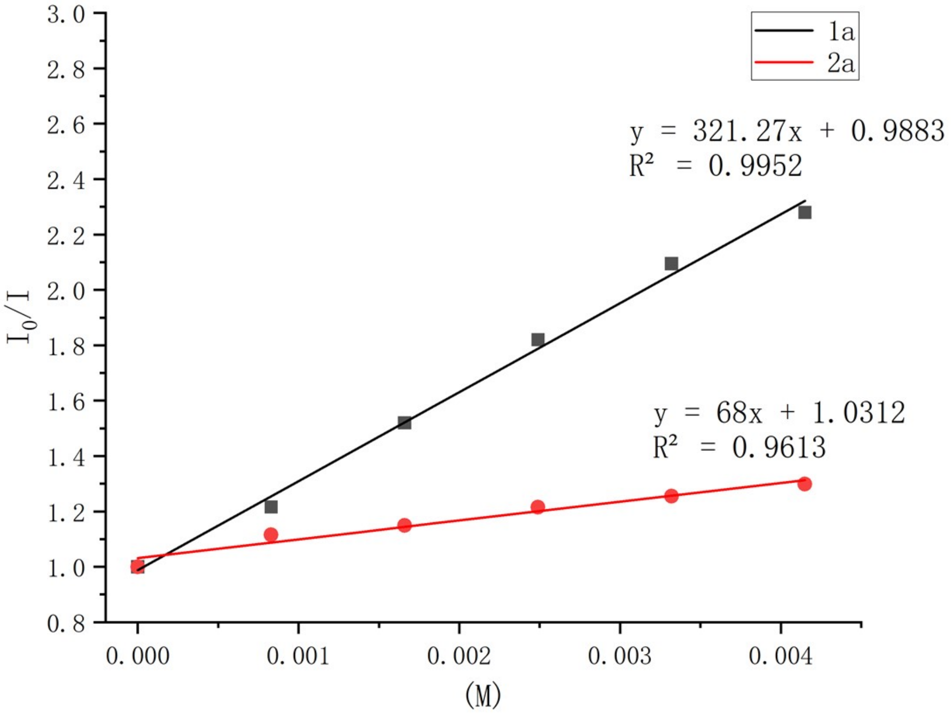


**Figure S6**. Stern-Volmer plot for the fluorescence quenching

# 6. Theoretical Calculations

Computational Details All calculations were performed using density functional theory as implemented within the Gaussian 16 (revision A.03) suite of programs[4]. Geometry optimizations were performed at the (U)M06-2X[5-6] level of theory with 6-311+ G(d,p) basis set, and vibrational frequencies of all the intermediates and transition states were performed at the (U)M06-2X/6-31+G(d,p). For modeling reactions conducted in solvent, solvation effects were incorporated using the SMD[7] model in geometry optimization, and frequency analysis (Acetonitrile, Eps= 35.688). All stationary points were confirmed to have no imaginary frequencies. Reported Gibbs free energies and enthalpies include thermal corrections computed at 298.15 K and 1 atm, and are reported in kcal/mol using the conversion of 1 Hartree = 627.5095kcal/mol. Molecular visualizations were created using CYLview[8].

**(A)**

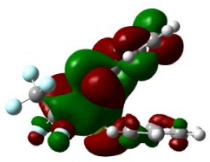

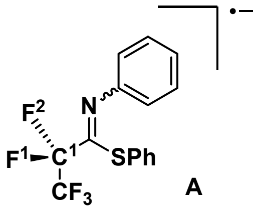


A

**(B)**

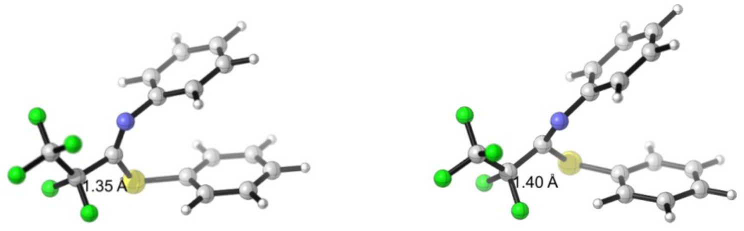


**Figure S7**. (A) SOMO of radical anion **A**. (B) The bond length of **1a** and radical anion **A**.

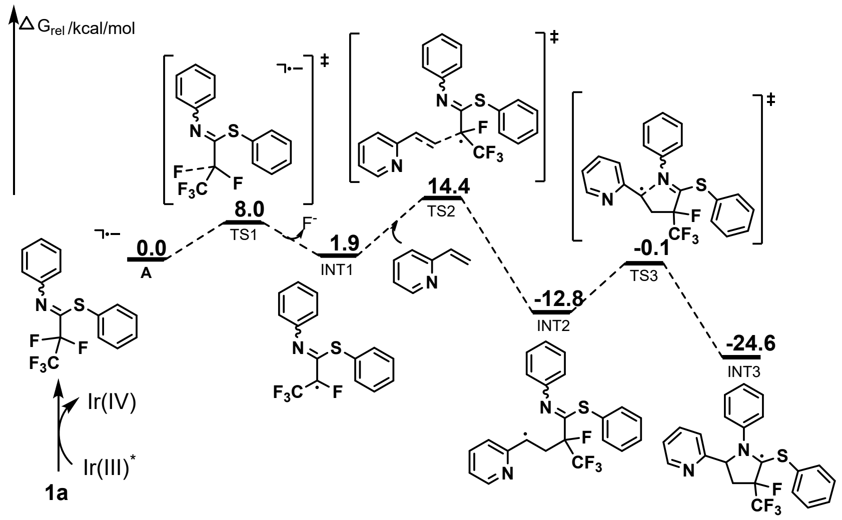


**Figure S8**. DFT calculated reaction energy profiles. Calculations were performed at the (U)M06-2X/6-31+G(d,p)/SMD($CH_3CN$) level of theory. Free energies are in kcal·mol$^{-1}$.

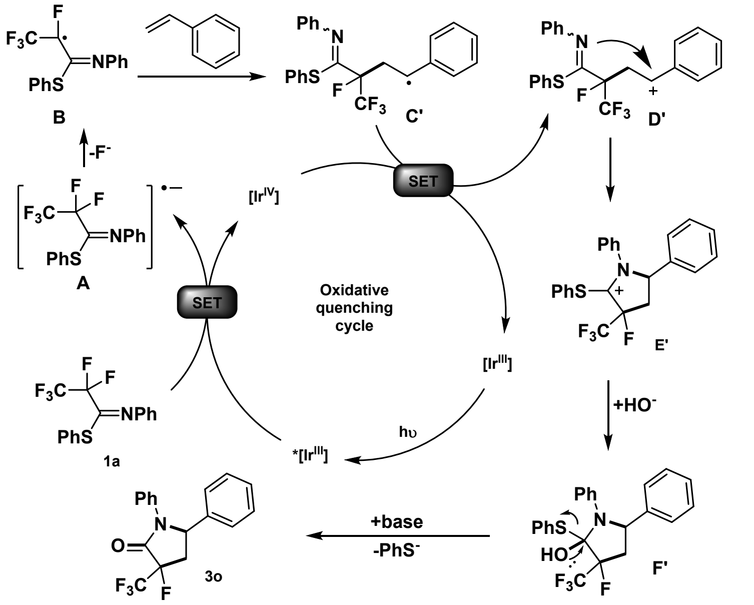


**Figure S9**. An alternative mechanism for styrene**.**

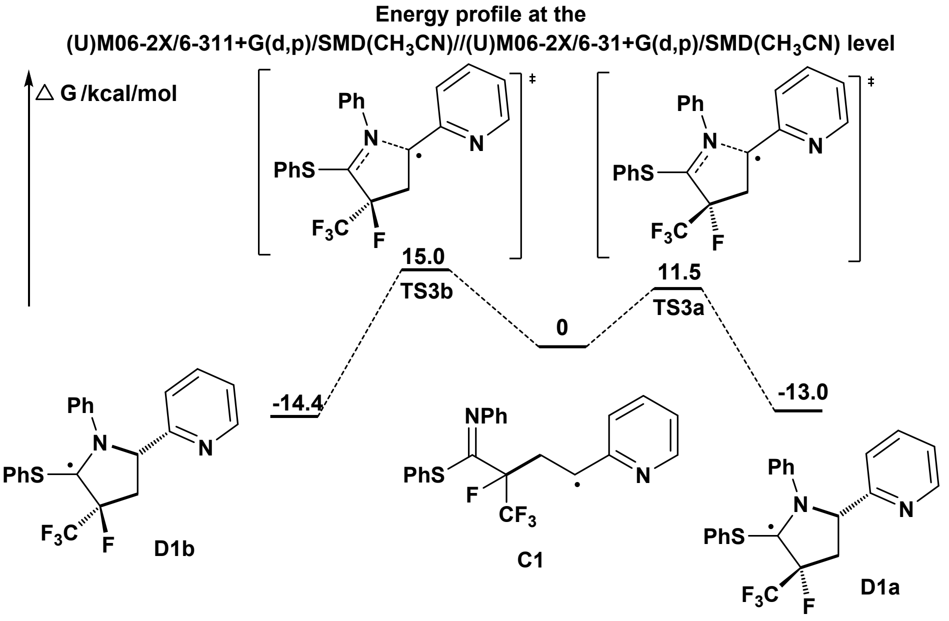


**Figure S10**. DFT-computed free energy changes of vinylpyridine to form **D1a** and **D1b.**

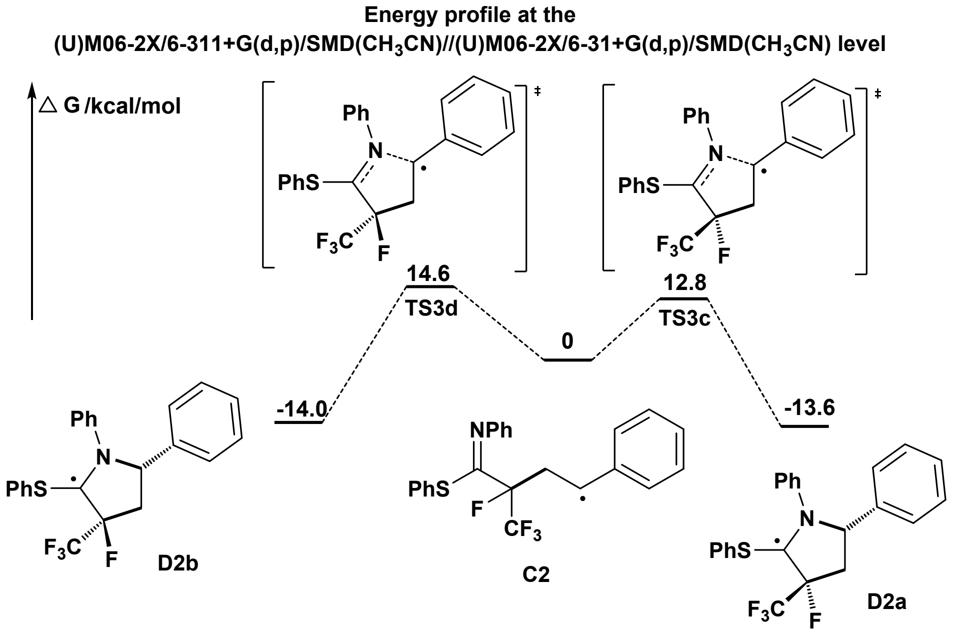


**Figure S11**. DFT-computed free energy changes of styrene to form **D2a** and **D2b**.

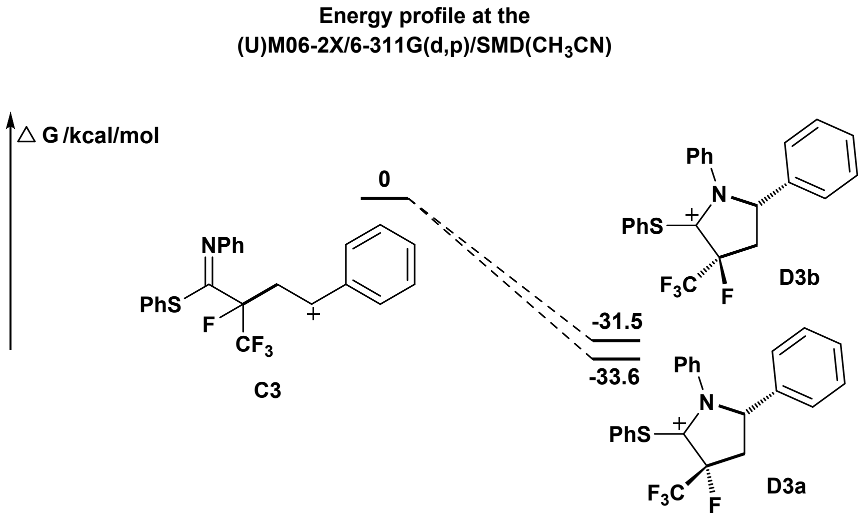


**Figure S12**. DFT-computed free energy changes of to form **D3a** and **D3b**.

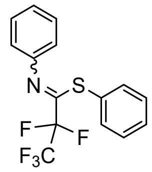


| | | | |
|---|---|---|---|
| C | -0.53302800 | 3.75714000 | 0.51900300 |
| C | -0.23133900 | 2.41160300 | 0.34143800 |
| C | 1.06195100 | 2.00529700 | -0.06806300 |
| C | 2.02580300 | 3.02334100 | -0.26325900 |
| C | 1.70957600 | 4.36199200 | -0.09263600 |
| C | 0.42349100 | 4.74713400 | 0.29680400 |
| N | 1.46635200 | 0.70378200 | -0.22599300 |
| C | 0.60733700 | -0.30356000 | -0.46534800 |
| C | 1.20198800 | -1.65233800 | -0.41557400 |
| S | -0.81401300 | -0.20266500 | -1.54297900 |
| C | -2.23542500 | -0.42532600 | -0.48032800 |
| C | -2.14102400 | -0.74265600 | 0.87432700 |
| C | -3.29919000 | -0.90194600 | 1.63209300 |
| C | -4.55448500 | -0.75270000 | 1.04933000 |
| C | -4.64569400 | -0.43916400 | -0.30575200 |
| C | -3.49508400 | -0.27531700 | -1.06906200 |
| F | 0.26207400 | -2.62158800 | -0.19850500 |
| F | 1.83159100 | -2.06414800 | -1.59304800 |
| C | 2.28122200 | -1.86073600 | 0.67041000 |
| F | 3.43405800 | -1.26828200 | 0.37173900 |
| F | 2.53904600 | -3.17181300 | 0.79922000 |
| F | 1.87025800 | -1.42073300 | 1.86035200 |
| H | -1.53095600 | 4.03503700 | 0.84319200 |
| H | -0.98610800 | 1.66511900 | 0.55397600 |
| H | 3.02664700 | 2.72885400 | -0.56190000 |

| | | | |
|---|---|---|---|
| H | 2.47282100 | 5.11486100 | -0.26037500 |
| H | 0.17745900 | 5.79364900 | 0.43284600 |
| H | -1.16536100 | -0.85928200 | 1.33369900 |
| H | -3.21444200 | -1.14610400 | 2.68538500 |
| H | -5.45233900 | -0.87851800 | 1.64303800 |
| H | -5.61667000 | -0.31776900 | -0.77301100 |
| H | -3.57503700 | -0.02776200 | -2.12292600 |

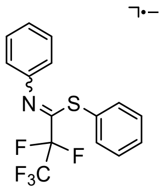


| | | | |
|---|---|---|---|
| C | -4.31002500 | -1.67081100 | -0.77659500 |
| C | -3.13812600 | -0.93978500 | -0.98764200 |
| C | -2.66027900 | -0.08344000 | 0.01898800 |
| C | -3.38191100 | 0.05826900 | 1.21763400 |
| C | -4.53259500 | -0.70023500 | 1.42950600 |
| C | -5.00244700 | -1.56558100 | 0.43340500 |
| N | -1.52382800 | 0.72566900 | -0.15017100 |
| C | -0.37885900 | 0.34816400 | -0.55009500 |
| C | 0.61057100 | 1.50859000 | -0.77370500 |
| S | 0.08382900 | -1.33493400 | -1.01916100 |
| C | 1.67119100 | -1.60782400 | -0.20858100 |
| C | 1.78252400 | -1.53809000 | 1.18650000 |
| C | 3.00370400 | -1.84130400 | 1.79487800 |
| C | 4.09878500 | -2.23752700 | 1.02003000 |
| C | 3.97434500 | -2.32560400 | -0.37024500 |
| C | 2.76338400 | -2.00545900 | -0.99059100 |
| F | 1.84520200 | 1.07366600 | -1.13754700 |
| F | 0.14968100 | 2.28886300 | -1.80472200 |
| C | 0.81767200 | 2.46207300 | 0.44584600 |
| F | -0.26686200 | 3.21191000 | 0.68788900 |
| F | 1.84812100 | 3.29497000 | 0.19731300 |
| F | 1.10900200 | 1.75672700 | 1.55380100 |
| H | -4.67956900 | -2.32313000 | -1.56261700 |
| H | -2.62412800 | -1.00121800 | -1.94147900 |
| H | -3.02200800 | 0.74828500 | 1.97483100 |
| H | -5.07199600 | -0.60393800 | 2.36737000 |
| H | -5.90903000 | -2.14141200 | 0.59482900 |
| H | 0.92830900 | -1.25303400 | 1.79249400 |
| H | 3.09260000 | -1.77794400 | 2.87564400 |
| H | 5.04340200 | -2.48016800 | 1.49819100 |
| H | 4.82084000 | -2.63443200 | -0.97683700 |

| | | | |
|---|---|---|---|
| H | 2.67321500 | -2.05760000 | -2.07126900 |

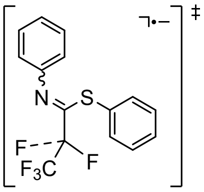

Ts1

| | | | |
|---|---|---|---|
| C | -0.38594200 | -0.71496600 | 0.18278000 |
| C | 0.70794700 | 0.14537100 | 0.20031800 |
| C | 1.29153900 | 0.58820600 | -1.00114200 |
| C | 0.71381700 | 0.18104400 | -2.21944100 |
| C | -0.39091400 | -0.66706500 | -2.22740200 |
| C | -0.94129900 | -1.12994800 | -1.03032700 |
| N | 2.35504300 | 1.47990200 | -0.92533800 |
| C | 3.41589800 | 1.40695100 | -1.70704700 |
| C | 4.29419800 | 2.50698700 | -1.74226400 |
| S | 3.85338400 | 0.00223300 | -2.75590300 |
| C | 5.30821200 | -0.58501100 | -1.89604200 |
| C | 5.39790100 | -0.56400700 | -0.50039200 |
| C | 6.53255700 | -1.07556600 | 0.12684800 |
| C | 7.57022400 | -1.62683100 | -0.62738400 |
| C | 7.47019300 | -1.65782600 | -2.01793700 |
| C | 6.34625800 | -1.13134900 | -2.65569900 |
| F | 5.53204000 | 2.32491400 | -2.22297400 |
| F | 3.79243900 | 3.72548600 | -3.03481500 |
| C | 4.29905500 | 3.53879700 | -0.63006200 |
| F | 3.14632000 | 4.19446300 | -0.48799600 |
| F | 5.26355600 | 4.44812900 | -0.83577300 |
| F | 4.57216300 | 2.95908700 | 0.56147400 |
| H | -0.81114800 | -1.05829800 | 1.12163300 |
| H | 1.13729000 | 0.47849600 | 1.14119300 |
| H | 1.11180400 | 0.56229800 | -3.15573200 |
| H | -0.82752600 | -0.96337100 | -3.17708200 |
| H | -1.80018400 | -1.79384100 | -1.04235400 |
| H | 4.58744000 | -0.14923800 | 0.09362300 |
| H | 6.59941400 | -1.05033400 | 1.21055700 |
| H | 8.44997000 | -2.02818200 | -0.13391600 |
| H | 8.27329900 | -2.08032600 | -2.61452000 |
| H | 6.28212600 | -1.13840300 | -3.74031300 |

$F^-$

| | | | |
|---|---|---|---|
| F | 0.00000000 | 0.00000000 | 0.00000000 |

1

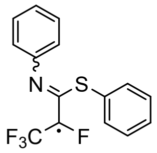


INT1

| | | | |
|---|---|---|---|
| C | -3.99886 | -1.99792 | -0.81941 |
| C | -2.84358 | -1.22559 | -0.923 |
| C | -2.5443 | -0.25987 | 0.06918 |
| C | -3.45964 | -0.06999 | 1.13383 |
| C | -4.59281 | -0.86867 | 1.24224 |
| C | -4.8679 | -1.83793 | 0.26698 |
| N | -1.45451 | 0.58688 | 0.04109 |
| C | -0.25752 | 0.33006 | -0.44096 |
| C | 0.61711 | 1.42624 | -0.51708 |
| S | 0.33554 | -1.30042 | -0.99206 |
| C | 1.93374 | -1.42906 | -0.16988 |
| C | 2.05012 | -1.26236 | 1.21749 |
| C | 3.29158 | -1.44478 | 1.83197 |
| C | 4.40707 | -1.81878 | 1.0743 |
| C | 4.28089 | -2.00185 | -0.30597 |
| C | 3.04784 | -1.79956 | -0.93408 |
| F | 1.79856 | 1.32543 | -1.13793 |
| C | 0.33899 | 2.81147 | 0.01498 |
| F | -0.67954 | 3.4313 | -0.63584 |
| F | 1.43053 | 3.5948 | -0.12558 |
| F | 0.01498 | 2.80473 | 1.32982 |
| H | -4.22584 | -2.72332 | -1.59527 |
| H | -2.21232 | -1.32779 | -1.79834 |
| H | -3.24415 | 0.69593 | 1.87254 |
| H | -5.27216 | -0.72866 | 2.07797 |
| H | -5.76225 | -2.44931 | 0.34266 |
| H | 1.1832 | -0.99437 | 1.81383 |
| H | 3.38144 | -1.30662 | 2.90574 |
| H | 5.36794 | -1.96846 | 1.55816 |
| H | 5.1428 | -2.29075 | -0.90068 |
| H | 2.95715 | -1.92302 | -2.0091 |

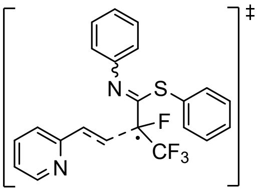


Ts2

| | | | |
|---|---|---|---|
| C | 2.99522500 | 2.75969000 | 0.26928600 |
| C | 2.10219400 | 1.71194400 | 0.07102700 |
| C | 1.38053100 | 1.19340000 | 1.15244700 |

| | | | |
|---|---|---|---|
| C | 1.58956000 | 1.72106000 | 2.43429700 |
| C | 2.46030100 | 2.78602000 | 2.61836700 |
| C | 3.16871600 | 3.30939600 | 1.53633200 |
| N | 0.51279800 | 0.10388600 | 1.04835500 |
| C | -0.35460000 | -0.06855300 | 0.12009000 |
| C | -1.10452200 | -1.30601900 | 0.18542300 |
| S | -0.70231500 | 1.03363200 | -1.26029400 |
| C | -2.46457000 | 1.20984800 | -1.02768700 |
| C | -3.01015500 | 1.39346600 | 0.24429400 |
| C | -4.38498700 | 1.55343700 | 0.38678300 |
| C | -5.21256800 | 1.56080900 | -0.73351300 |
| C | -4.66139800 | 1.39468200 | -2.00136600 |
| C | -3.29053200 | 1.20720300 | -2.15157600 |
| C | 0.32879500 | -2.94815300 | -0.57006600 |
| F | -2.06314500 | -1.52315200 | -0.71317000 |
| C | 1.55543300 | -2.59555100 | -0.11469300 |
| C | 2.43447900 | -1.68592600 | -0.84839200 |
| C | 3.68949000 | -1.33394200 | -0.33102300 |
| C | 4.51633400 | -0.49981400 | -1.07102300 |
| C | 4.07036800 | -0.03630300 | -2.30468700 |
| C | 2.79680700 | -0.42057700 | -2.72844000 |
| N | 1.99204700 | -1.21471600 | -2.02953900 |
| C | -1.45168300 | -1.92393200 | 1.52327000 |
| F | -2.23513600 | -2.99174500 | 1.33062400 |
| F | -2.13129000 | -1.05742300 | 2.29283900 |
| F | -0.38423400 | -2.31910300 | 2.21315300 |
| H | 3.55924500 | 3.14365400 | -0.57565300 |
| H | 1.99535700 | 1.26272600 | -0.91047000 |
| H | 1.04730200 | 1.28374000 | 3.26638900 |
| H | 2.59949900 | 3.20076700 | 3.61193200 |
| H | 3.86148000 | 4.13169000 | 1.68468200 |
| H | -2.36309900 | 1.40743600 | 1.11676400 |
| H | -4.80745100 | 1.68439600 | 1.37812600 |
| H | -6.28346800 | 1.69417900 | -0.61843700 |
| H | -5.30133400 | 1.39394000 | -2.87830700 |
| H | -2.86045700 | 1.04666900 | -3.13544300 |
| H | 0.02917600 | -2.66923100 | -1.57553300 |
| H | -0.25962900 | -3.70276600 | -0.05771600 |
| H | 1.90327800 | -2.93893800 | 0.85561800 |
| H | 3.99748700 | -1.71355700 | 0.63808700 |
| H | 5.49119900 | -0.21268100 | -0.68928400 |
| H | 4.68188900 | 0.61215200 | -2.92257100 |
| H | 2.40298700 | -0.06363500 | -3.67810200 |

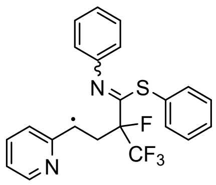

INT2

| | | | |
|---|---|---|---|
| C | 2.74278400 | 3.02663400 | 0.08812100 |
| C | 2.03322600 | 1.83113500 | 0.00639700 |
| C | 1.14180700 | 1.48490000 | 1.02558400 |
| C | 0.98357100 | 2.32504000 | 2.13275100 |
| C | 1.67463700 | 3.53080500 | 2.19068300 |
| C | 2.55964100 | 3.88434900 | 1.17120500 |
| N | 0.44906800 | 0.26080800 | 1.03274800 |
| C | -0.29780400 | -0.16022700 | 0.10495600 |
| C | -0.82730900 | -1.58647900 | 0.26725400 |
| S | -0.66829100 | 0.68421500 | -1.44292000 |
| C | -2.42778500 | 0.91177300 | -1.21168400 |
| C | -2.92944700 | 1.44591700 | -0.02346600 |
| C | -4.30054100 | 1.64724800 | 0.11665600 |
| C | -5.16615500 | 1.34464900 | -0.93322900 |
| C | -4.65764400 | 0.82825400 | -2.12411400 |
| C | -3.29057100 | 0.60006900 | -2.26325100 |
| C | 0.19994500 | -2.62169700 | -0.24669300 |
| F | -1.98084300 | -1.76084400 | -0.46680200 |
| C | 1.59123900 | -2.41662100 | 0.24750900 |
| C | 2.56602700 | -1.71482300 | -0.51964800 |
| C | 3.85122900 | -1.44584900 | 0.01148200 |
| C | 4.76849900 | -0.75075400 | -0.75651700 |
| C | 4.40274900 | -0.33896300 | -2.04056700 |
| C | 3.11291400 | -0.64237400 | -2.48597100 |
| N | 2.21223200 | -1.29678400 | -1.76212600 |
| C | -1.25209800 | -1.88152300 | 1.71864000 |
| F | -1.95816900 | -3.02263700 | 1.75256600 |
| F | -2.02908500 | -0.91338300 | 2.21651900 |
| F | -0.20679700 | -2.04468600 | 2.53677800 |
| H | 3.43831300 | 3.28729600 | -0.70377900 |
| H | 2.17777700 | 1.15741800 | -0.83189400 |
| H | 0.31347400 | 2.02002600 | 2.93047700 |
| H | 1.53334200 | 4.18950700 | 3.04172100 |
| H | 3.10927400 | 4.81837500 | 1.22637000 |
| H | -2.25084400 | 1.70031100 | 0.78617400 |
| H | -4.68981900 | 2.05028100 | 1.04624700 |
| H | -6.23315800 | 1.50924300 | -0.82322700 |
| H | -5.32736100 | 0.58617000 | -2.94331200 |
| H | -2.89275800 | 0.16745300 | -3.17586800 |

| | | | |
|---|---|---|---|
| H | 0.17924400 | -2.52397700 | -1.33557400 |
| H | -0.18148200 | -3.61097700 | 0.02868700 |
| H | 1.86082900 | -2.75146100 | 1.24252800 |
| H | 4.09433000 | -1.77732000 | 1.01598300 |
| H | 5.75541900 | -0.52673900 | -0.36396900 |
| H | 5.08976300 | 0.20356500 | -2.68008900 |
| H | 2.78776900 | -0.32959900 | -3.47624200 |

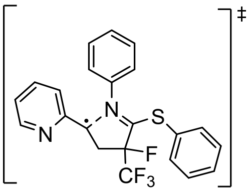


Ts3

| | | | |
|---|---|---|---|
| C | 2.56957600 | 3.21190100 | -0.34346200 |
| C | 1.91829800 | 1.98030500 | -0.45792000 |
| C | 1.27000000 | 1.42805600 | 0.65740200 |
| C | 1.29918600 | 2.10332100 | 1.88767400 |
| C | 1.93997100 | 3.33970500 | 1.99007000 |
| C | 2.57789500 | 3.89696300 | 0.87566900 |
| N | 0.62960500 | 0.16499600 | 0.61716200 |
| C | -0.44222100 | -0.13918800 | -0.07293900 |
| C | -0.74540200 | -1.61782100 | 0.09419900 |
| S | -1.12262400 | 0.82703900 | -1.42519900 |
| C | -2.88064000 | 0.90596600 | -1.02634700 |
| C | -3.32326600 | 1.58301700 | 0.11900100 |
| C | -4.69305300 | 1.70325800 | 0.36555200 |
| C | -5.62153800 | 1.16895800 | -0.53547800 |
| C | -5.17653400 | 0.50719700 | -1.68365000 |
| C | -3.80624800 | 0.36896300 | -1.93010000 |
| C | 0.62642300 | -2.34765100 | 0.01467800 |
| F | -1.58692200 | -2.09804200 | -0.91525200 |
| C | 1.72349200 | -1.50564000 | 0.59941600 |
| C | 2.94567600 | -1.26591300 | -0.14875500 |
| C | 4.12520400 | -0.87888900 | 0.53061100 |
| C | 5.28369700 | -0.63326700 | -0.19676300 |
| C | 5.25046500 | -0.77077100 | -1.58947900 |
| C | 4.04267900 | -1.15188200 | -2.18461100 |
| N | 2.91877700 | -1.39418000 | -1.50255500 |
| C | -1.51406900 | -1.95084900 | 1.39513600 |
| F | -1.77860300 | -3.27835700 | 1.47515800 |
| F | -2.70296200 | -1.31244700 | 1.46322300 |
| F | -0.80999500 | -1.61706400 | 2.49817600 |
| H | 3.06981800 | 3.63427700 | -1.21041200 |
| H | 1.92280600 | 1.44570900 | -1.40182000 |

| | | | |
|---|---|---|---|
| H | 0.81055500 | 1.65755200 | 2.74902200 |
| H | 1.94618000 | 3.86364300 | 2.94165200 |
| H | 3.08289000 | 4.85502200 | 0.95923200 |
| H | -2.60487400 | 2.01131700 | 0.81134000 |
| H | -5.03339800 | 2.22358400 | 1.25650900 |
| H | -6.68591800 | 1.27090200 | -0.34371700 |
| H | -5.89196100 | 0.08994800 | -2.38667100 |
| H | -3.46164800 | -0.15590800 | -2.81589400 |
| H | 0.80615400 | -2.51027200 | -1.05054600 |
| H | 0.56718900 | -3.32167500 | 0.50908300 |
| H | 1.85986500 | -1.56555500 | 1.67414500 |
| H | 4.11473000 | -0.78623700 | 1.61253700 |
| H | 6.19917500 | -0.34206100 | 0.31046200 |
| H | 6.12958700 | -0.59144000 | -2.19995500 |
| H | 3.98094000 | -1.26837900 | -3.26521400 |

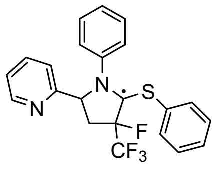

INT3

| | | | |
|---|---|---|---|
| C | 0.21936600 | -3.62049900 | 0.76244200 |
| C | 0.59272100 | -2.29621300 | 0.99513800 |
| C | 0.25455500 | -1.31729900 | 0.06674200 |
| C | -0.44109200 | -1.64864100 | -1.09799700 |
| C | -0.80921700 | -2.97029500 | -1.32676700 |
| C | -0.48060900 | -3.95661200 | -0.39447600 |
| N | 0.62646100 | 0.04260900 | 0.31514800 |
| C | -0.32592600 | 0.99687100 | 0.65031300 |
| C | 0.23665200 | 2.36024700 | 0.40189700 |
| S | -1.63076700 | 0.68629600 | 1.76273400 |
| C | -2.71491400 | -0.33710000 | 0.75587000 |
| C | -3.03036300 | -1.62134300 | 1.19756700 |
| C | -3.86290500 | -2.43347300 | 0.42769400 |
| C | -4.37135700 | -1.96719500 | -0.78264900 |
| C | -4.05790900 | -0.67811100 | -1.21748600 |
| C | -3.23834700 | 0.14250900 | -0.44692900 |
| C | 1.67655600 | 2.09907200 | -0.05118000 |
| F | 0.21263700 | 3.16661700 | 1.53543700 |
| C | 1.67538000 | 0.64446200 | -0.52465200 |
| C | 2.98665600 | -0.09088500 | -0.34144700 |
| C | 3.42717400 | -0.99435800 | -1.30884800 |
| C | 4.60939200 | -1.69404100 | -1.08320500 |
| C | 5.30498500 | -1.46316800 | 0.09811200 |

| | | | |
|---|---|---|---|
| C | 4.78172500 | -0.53881200 | 1.00294100 |
| N | 3.65017000 | 0.13367300 | 0.79607300 |
| C | -0.55857500 | 3.14998100 | -0.64134800 |
| F | -0.02682900 | 4.36283300 | -0.85045200 |
| F | -1.83634700 | 3.32261400 | -0.29160800 |
| F | -0.54161800 | 2.49891300 | -1.82300800 |
| H | 0.47458400 | -4.38737900 | 1.48692300 |
| H | 1.13300200 | -2.00375600 | 1.89018800 |
| H | -0.71807600 | -0.86199200 | -1.79576000 |
| H | -1.36825300 | -3.22782400 | -2.22086100 |
| H | -0.77552100 | -4.98649900 | -0.56995000 |
| H | -2.59974600 | -1.99044100 | 2.12377600 |
| H | -4.09640000 | -3.43788900 | 0.76754000 |
| H | -5.01210200 | -2.60339700 | -1.38535600 |
| H | -4.46098400 | -0.30643700 | -2.15475500 |
| H | -2.99239100 | 1.14768000 | -0.77619400 |
| H | 2.30942000 | 2.18385400 | 0.83557900 |
| H | 2.02346100 | 2.79520800 | -0.81665800 |
| H | 1.37576500 | 0.58227700 | -1.58214600 |
| H | 2.84389100 | -1.15038400 | -2.21122700 |
| H | 4.97676200 | -2.40669400 | -1.81485900 |
| H | 6.23091500 | -1.98181800 | 0.32086700 |
| H | 5.29725100 | -0.32858900 | 1.93688200 |

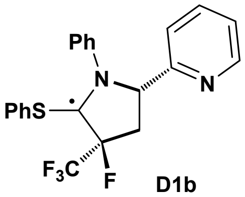


D1b

| | | | |
|---|---|---|---|
| C | 0.27893600 | -0.52107700 | 0.07950200 |
| C | -0.30137700 | -1.77159200 | -0.12110200 |
| C | -1.68819600 | -1.92477500 | 0.01128000 |
| C | -2.48166200 | -0.82148200 | 0.35251500 |
| C | -1.89023700 | 0.42447400 | 0.53996000 |
| C | -0.50965700 | 0.58262100 | 0.40536000 |
| N | -2.26968400 | -3.19591000 | -0.16188400 |
| C | -3.48129400 | -3.45805600 | -0.78034300 |
| C | -3.66502900 | -4.93990200 | -0.83339400 |
| S | -4.14094100 | -2.40509100 | -2.01319400 |
| C | -5.63169800 | -1.73754500 | -1.27617100 |
| C | -5.97459000 | -1.90261500 | 0.06685700 |
| C | -7.15628400 | -1.33990900 | 0.55158200 |
| C | -7.99352600 | -0.61105200 | -0.29208500 |
| C | -7.64277200 | -0.44646600 | -1.63336700 |
| C | -6.46853600 | -1.00894800 | -2.12890300 |

| | | | |
|---|---|---|---|
| C | -2.65490500 | -5.49032000 | 0.16178000 |
| F | -3.38338800 | -5.43618600 | -2.12504300 |
| C | -1.54162200 | -4.43124400 | 0.18464000 |
| C | -5.08526400 | -5.43773300 | -0.56832800 |
| F | -5.13618000 | -6.77956600 | -0.63503300 |
| F | -5.96581700 | -4.96400800 | -1.45967300 |
| F | -5.51185000 | -5.08304500 | 0.65221100 |
| H | 1.35395300 | -0.41202700 | -0.02704100 |
| H | 0.32078500 | -2.62066600 | -0.38729700 |
| H | -3.55070400 | -0.94358600 | 0.49192800 |
| H | -2.51346200 | 1.27280500 | 0.80629200 |
| H | -0.05282500 | 1.55509100 | 0.55946800 |
| H | -5.32645400 | -2.46652900 | 0.73215300 |
| H | -7.41739800 | -1.47422200 | 1.59726200 |
| H | -8.91120400 | -0.17557500 | 0.09058600 |
| H | -8.28650500 | 0.11794800 | -2.30146500 |
| H | -6.20673800 | -0.88365500 | -3.17677200 |
| H | -2.29643600 | -6.47520900 | -0.13940100 |
| H | -3.11868300 | -5.56180900 | 1.15003600 |
| H | -0.79101500 | -4.64501600 | -0.58551000 |
| C | -0.84659900 | -4.34310900 | 1.52660800 |
| C | -1.50271500 | -3.80821100 | 2.63820400 |
| C | -0.82854600 | -3.76997400 | 3.85288100 |
| H | -2.51660300 | -3.42751400 | 2.54244300 |
| C | 1.03508500 | -4.79251900 | 2.75470000 |
| C | 0.47010100 | -4.27359600 | 3.91569300 |
| H | -1.30616000 | -3.35482400 | 4.73504100 |
| H | 2.04484400 | -5.19456600 | 2.76445600 |
| H | 1.03635400 | -4.26404900 | 4.84068300 |
| N | 0.39783800 | -4.82493700 | 1.57667100 |

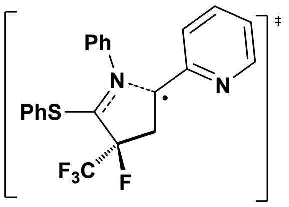


**TS3b**

| | | | |
|---|---|---|---|
| C | 0.38771100 | -1.01424400 | 0.27809200 |
| C | -0.50893900 | -2.06192400 | 0.07585300 |
| C | -1.33236900 | -2.47844800 | 1.12698200 |
| C | -1.23930900 | -1.86945600 | 2.38155500 |
| C | -0.35172800 | -0.81192500 | 2.57034900 |
| C | 0.46352600 | -0.38355400 | 1.52096800 |

| | | | |
|---|---|---|---|
| N | -2.19904900 | -3.58653800 | 0.99409700 |
| C | -3.16717800 | -3.68906900 | 0.14012700 |
| C | -3.73150300 | -5.09537100 | 0.17428200 |
| S | -3.50107100 | -2.57321100 | -1.20754400 |
| C | -5.28066300 | -2.41112400 | -1.06530100 |
| C | -5.82021200 | -1.66457600 | -0.01436000 |
| C | -7.20152200 | -1.50800400 | 0.07916800 |
| C | -8.03768900 | -2.08252300 | -0.88019200 |
| C | -7.49218200 | -2.81408500 | -1.93518000 |
| C | -6.11026700 | -2.98275600 | -2.03073900 |
| C | -2.53005000 | -6.08559600 | 0.36753900 |
| F | -4.41052400 | -5.39854100 | -0.99126800 |
| C | -1.27084100 | -5.37198000 | 0.75920000 |
| C | -4.78545500 | -5.28605700 | 1.27274400 |
| F | -5.21817100 | -6.55881400 | 1.30007800 |
| F | -5.86196600 | -4.51531500 | 1.07118800 |
| F | -4.29550800 | -4.99764000 | 2.48373900 |
| H | 1.02997600 | -0.69399700 | -0.53664800 |
| H | -0.55451000 | -2.57283700 | -0.88239200 |
| H | -1.86649700 | -2.22915500 | 3.19243000 |
| H | -0.28841500 | -0.33076500 | 3.54152700 |
| H | 1.16311300 | 0.43239800 | 1.67391900 |
| H | -5.16341300 | -1.21766300 | 0.72711900 |
| H | -7.62331900 | -0.93351700 | 0.89828400 |
| H | -9.11363400 | -1.95617900 | -0.80689500 |
| H | -8.14038200 | -3.25996500 | -2.68340200 |
| H | -5.68084500 | -3.56147400 | -2.84296900 |
| H | -2.40423100 | -6.56454600 | -0.60709900 |
| H | -2.80169200 | -6.86543400 | 1.08225000 |
| H | -0.62372800 | -5.05208300 | -0.05370400 |
| C | -0.57880300 | -5.56311100 | 2.01865500 |
| C | -1.20456400 | -6.08812400 | 3.16563700 |
| C | -0.49218300 | -6.14960200 | 4.35592700 |
| H | -2.22975100 | -6.43966400 | 3.12228400 |
| C | 1.36387200 | -5.17674500 | 3.20459300 |
| C | 0.82279700 | -5.68623300 | 4.38580900 |
| H | -0.95779400 | -6.55234400 | 5.25053400 |
| H | 2.38608900 | -4.80525500 | 3.18748400 |
| H | 1.41641300 | -5.71483500 | 5.29290600 |
| N | 0.69640800 | -5.10789700 | 2.05293000 |

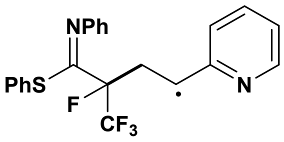

**C1**

| | | | |
|---|---|---|---|
| C | 0.59417000 | -1.81708700 | 0.02316700 |
| C | 0.84995400 | -2.67086500 | 1.09502400 |
| C | 0.83553700 | -2.16503000 | 2.39858800 |
| C | 0.59267200 | -0.80718500 | 2.62763500 |
| C | 0.31667100 | 0.03226200 | 1.55080600 |
| C | 0.31885200 | -0.46777600 | 0.24691700 |
| N | 1.14364600 | -2.98682700 | 3.50330400 |
| C | 0.46485000 | -4.00140600 | 3.83159000 |
| C | 1.04994300 | -4.85378800 | 4.96681100 |
| S | -0.99596800 | -4.59877900 | 2.96529700 |
| C | -2.17293800 | -4.68347800 | 4.31227500 |
| C | -2.42319600 | -3.55644900 | 5.09804700 |
| C | -3.37757600 | -3.62623400 | 6.11246100 |
| C | -4.09664700 | -4.80371000 | 6.32022200 |
| C | -3.85663900 | -5.91815200 | 5.51509100 |
| C | -2.88855400 | -5.86554100 | 4.51324600 |
| C | 2.26923600 | -5.63228200 | 4.44283800 |
| F | 0.11453800 | -5.75960400 | 5.42741000 |
| C | 2.83651900 | -6.58668300 | 5.43816000 |
| C | 4.13002700 | -6.50403500 | 6.02658700 |
| C | 5.63944600 | -7.49481700 | 7.47444600 |
| C | 6.60116100 | -6.50116800 | 7.26016100 |
| C | 6.28626500 | -5.46526300 | 6.38078700 |
| C | 1.38258000 | -3.98028900 | 6.19430800 |
| F | 1.54963900 | -4.74456400 | 7.28342500 |
| F | 0.39143300 | -3.11863300 | 6.46941600 |
| F | 2.50399600 | -3.27343400 | 6.02361800 |
| H | 0.60873700 | -2.21092600 | -0.98858200 |
| H | 1.07919500 | -3.71990900 | 0.92632000 |
| H | 0.60953500 | -0.42737200 | 3.64500300 |
| H | 0.10933200 | 1.08269100 | 1.73135000 |
| H | 0.11540300 | 0.19276900 | -0.59011400 |
| H | -1.87700200 | -2.63343600 | 4.92104400 |
| H | -3.56540100 | -2.75423200 | 6.73156400 |
| H | -4.84433000 | -4.85221800 | 7.10595000 |
| H | -4.41461900 | -6.83613700 | 5.67261900 |
| H | -2.68302300 | -6.73902500 | 3.90144100 |
| H | 2.99861300 | -4.90593900 | 4.07975900 |
| H | 1.90109500 | -6.19043400 | 3.57116700 |

| | | | |
|---|---|---|---|
| H | 2.21937000 | -7.42565300 | 5.74367700 |
| H | 5.85089800 | -8.31683000 | 8.15505600 |
| H | 7.55725300 | -6.54607100 | 7.76960400 |
| H | 6.99879500 | -4.66978000 | 6.18464400 |
| C | 5.04793700 | -5.45863600 | 5.75734900 |
| H | 4.78831000 | -4.65709000 | 5.07498900 |
| N | 4.44704100 | -7.51194200 | 6.89096300 |

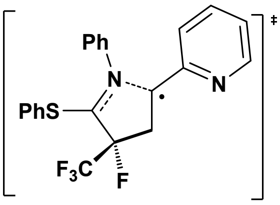


**TS3a**

| | | | |
|---|---|---|---|
| C | 2.96383900 | 2.77963100 | -0.28158600 |
| C | 2.07770000 | 1.70982300 | -0.39642000 |
| C | 1.46547700 | 1.19162100 | 0.75042900 |
| C | 1.76352100 | 1.72555000 | 2.00793200 |
| C | 2.64010200 | 2.80317100 | 2.11331200 |
| C | 3.24215900 | 3.33159400 | 0.96958400 |
| N | 0.61729300 | 0.06086400 | 0.71031600 |
| C | -0.41834600 | -0.08959100 | -0.05903300 |
| C | -0.93283400 | -1.50686400 | 0.06357800 |
| S | -0.93972600 | 0.97292400 | -1.38674400 |
| C | -2.68695400 | 1.10830600 | -1.00487000 |
| C | -3.09482400 | 1.82572000 | 0.12288000 |
| C | -4.45419200 | 1.96347800 | 0.39576700 |
| C | -5.40140600 | 1.40047900 | -0.46193300 |
| C | -4.98858800 | 0.69662600 | -1.59318500 |
| C | -3.62865400 | 0.54508700 | -1.86716600 |
| C | 0.32050800 | -2.42383900 | 0.01473100 |
| F | -1.80493400 | -1.83555000 | -0.95504300 |
| C | 1.50963600 | -1.73313000 | 0.60930700 |
| C | 2.67565900 | -1.44843300 | -0.20030000 |
| C | 3.90067000 | -1.11674400 | 0.41743000 |
| C | 4.97650600 | -0.74298300 | -0.37184300 |
| C | 4.81543100 | -0.70540800 | -1.75993000 |
| C | 3.57165100 | -1.05367200 | -2.28671400 |
| N | 2.52294100 | -1.41252500 | -1.54398300 |
| C | -1.74500600 | -1.74243400 | 1.34391200 |
| F | -2.22628000 | -2.99672800 | 1.37255500 |
| F | -2.79716300 | -0.91751900 | 1.43142200 |
| F | -0.99761900 | -1.57311200 | 2.44156900 |
| H | 3.44175900 | 3.17619000 | -1.17234800 |

| | | | |
|---|---|---|---|
| H | 1.88721800 | 1.25934600 | -1.36614900 |
| H | 1.30065700 | 1.29056400 | 2.88917600 |
| H | 2.85999100 | 3.22391900 | 3.08970000 |
| H | 3.93344800 | 4.16447300 | 1.05442000 |
| H | -2.35280600 | 2.26455000 | 0.78477400 |
| H | -4.77252600 | 2.51529800 | 1.27504000 |
| H | -6.46022300 | 1.51258500 | -0.24898100 |
| H | -5.72272900 | 0.25764100 | -2.26188900 |
| H | -3.30148200 | -0.01452300 | -2.73839700 |
| H | 0.48952900 | -2.62208000 | -1.04630700 |
| H | 0.11131700 | -3.36907200 | 0.52320300 |
| H | 1.69349600 | -1.87240800 | 1.67029500 |
| H | 3.98089400 | -1.15403800 | 1.50001700 |
| H | 5.92754000 | -0.48203900 | 0.08302900 |
| H | 5.62773200 | -0.41924900 | -2.41900400 |
| H | 3.41179400 | -1.03805800 | -3.36281400 |

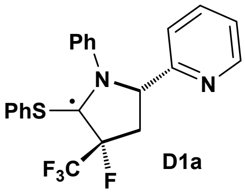

D1a

| | | | |
|---|---|---|---|
| C | 0.01726100 | -3.54869000 | 1.16097500 |
| C | 0.39553800 | -2.21018300 | 1.27566800 |
| C | 0.25939100 | -1.36245000 | 0.17808100 |
| C | -0.23096500 | -1.84273100 | -1.03860400 |
| C | -0.60149800 | -3.18141500 | -1.14841400 |
| C | -0.48261300 | -4.03371300 | -0.04772600 |
| N | 0.60906300 | 0.01673000 | 0.31632600 |
| C | -0.31357800 | 0.97775300 | 0.70221700 |
| C | 0.26251500 | 2.33156600 | 0.47782500 |
| S | -1.66360500 | 0.64562000 | 1.74815600 |
| C | -2.72208700 | -0.34769300 | 0.68008300 |
| C | -3.17609600 | -1.57877400 | 1.15518600 |
| C | -3.99696800 | -2.37062300 | 0.34933800 |
| C | -4.35485500 | -1.93776200 | -0.92691100 |
| C | -3.90102700 | -0.70178000 | -1.39415100 |
| C | -3.09103800 | 0.09883300 | -0.59132000 |
| C | 1.66178300 | 2.06527900 | -0.08438700 |
| F | 0.34195200 | 3.08355000 | 1.66789800 |
| C | 1.61869500 | 0.61216800 | -0.57359200 |
| C | 2.93854200 | -0.12021500 | -0.46409700 |
| C | 3.51150400 | -0.72030500 | -1.58360700 |
| C | 4.72256300 | -1.39509600 | -1.43488000 |
| C | 5.30714000 | -1.44810800 | -0.17448300 |

| | | | |
|---|---|---|---|
| C | 4.65334100 | -0.81911400 | 0.88633700 |
| N | 3.49650000 | -0.16718700 | 0.75203100 |
| C | -0.57532900 | 3.22492300 | -0.43905100 |
| F | -0.01841300 | 4.44004900 | -0.58130100 |
| F | -1.81923100 | 3.41597300 | 0.02192600 |
| F | -0.67283900 | 2.68581900 | -1.66555800 |
| H | 0.11484100 | -4.21062700 | 2.01603600 |
| H | 0.78665600 | -1.81361600 | 2.20805500 |
| H | -0.33889000 | -1.16505400 | -1.88172100 |
| H | -0.99233100 | -3.55639500 | -2.08966700 |
| H | -0.77886100 | -5.07481600 | -0.13408100 |
| H | -2.87384400 | -1.92660600 | 2.13935700 |
| H | -4.34118100 | -3.33285900 | 0.71703200 |
| H | -4.98409400 | -2.55952300 | -1.55651600 |
| H | -4.18170200 | -0.35692600 | -2.38503500 |
| H | -2.73626500 | 1.05839800 | -0.95636400 |
| H | 2.35759300 | 2.14674500 | 0.75524700 |
| H | 1.95824600 | 2.76295100 | -0.86898500 |
| H | 1.26832300 | 0.56783300 | -1.61348300 |
| H | 3.01272600 | -0.65992500 | -2.54608800 |
| H | 5.19506200 | -1.87301500 | -2.28753300 |
| H | 6.24669400 | -1.96335400 | -0.00610700 |
| H | 5.08061600 | -0.84106200 | 1.88593200 |

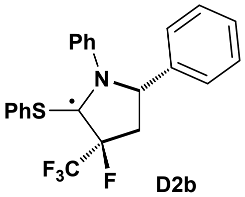


D2b

| | | | |
|---|---|---|---|
| C | -0.16790500 | -1.03882200 | 0.00106800 |
| C | 0.14856900 | 0.31622500 | 0.06682900 |
| C | -0.76635100 | 1.21859000 | 0.62505600 |
| C | -1.99734700 | 0.75468800 | 1.10676500 |
| C | -2.29749400 | -0.60318400 | 1.04455100 |
| C | -1.38735300 | -1.50629800 | 0.49219900 |
| N | -0.45427800 | 2.59121700 | 0.66897000 |
| C | -0.72839600 | 3.44903700 | 1.72063100 |
| C | -0.14026300 | 4.78349100 | 1.40043100 |
| S | -0.88835600 | 2.88124000 | 3.36783100 |
| C | 0.14094400 | 4.73256700 | -0.09342200 |
| F | 1.06486100 | 4.98876300 | 2.10943100 |
| C | 0.34893500 | 3.23860300 | -0.39056500 |
| C | -0.99159500 | 5.98835700 | 1.79746600 |
| F | -0.38537900 | 7.13524900 | 1.44363800 |
| F | -1.20876700 | 6.04782200 | 3.11810500 |

| | | | |
|---|---|---|---|
| F | -2.18686200 | 5.96581300 | 1.18985700 |
| H | 0.54812100 | -1.73111700 | -0.43132600 |
| H | 1.10547800 | 0.66976200 | -0.30433100 |
| H | -2.72139200 | 1.45649200 | 1.50770200 |
| H | -3.25499800 | -0.95300700 | 1.41852700 |
| H | -1.62902500 | -2.56321300 | 0.43999300 |
| H | 1.00859500 | 5.34007600 | -0.35380000 |
| H | -0.73167800 | 5.10038800 | -0.64115800 |
| C | -2.61210000 | 3.16227700 | 3.77037400 |
| C | -3.57159600 | 3.55817700 | 2.83703200 |
| C | -2.97686900 | 2.95486100 | 5.10543300 |
| C | -4.89320700 | 3.74807100 | 3.24354200 |
| H | -3.29219300 | 3.71928500 | 1.79951800 |
| C | -4.29998900 | 3.14188500 | 5.49950800 |
| H | -2.23060100 | 2.65105400 | 5.83557200 |
| C | -5.26380400 | 3.54131900 | 4.57164200 |
| H | -5.63352400 | 4.05816400 | 2.51178400 |
| H | -4.57511800 | 2.97855000 | 6.53726900 |
| H | -6.29322900 | 3.69018500 | 4.88213600 |
| C | -0.09570500 | 2.84588700 | -1.78141700 |
| C | 0.84775900 | 2.49753800 | -2.74769500 |
| C | -1.45448500 | 2.85163200 | -2.11532200 |
| C | 0.44102900 | 2.16321400 | -4.04132700 |
| H | 1.90301000 | 2.48154300 | -2.48532200 |
| C | -1.86225600 | 2.51084400 | -3.40245400 |
| H | -2.19322200 | 3.11706900 | -1.36062700 |
| C | -0.91360700 | 2.16680600 | -4.36946200 |
| H | 1.18257900 | 1.89322600 | -4.78723200 |
| H | -2.91900700 | 2.51173800 | -3.65242400 |
| H | -1.23193400 | 1.90004700 | -5.37278900 |
| H | 1.40404800 | 2.97049100 | -0.25082000 |

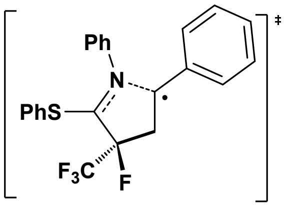


TS3d

| | | | |
|---|---|---|---|
| C | 0.59976400 | -0.48845900 | 0.25085800 |
| C | -0.29847500 | -1.53711500 | 0.06004800 |
| C | -1.11329000 | -1.95228400 | 1.11939500 |
| C | -1.00686600 | -1.33855900 | 2.37120600 |
| C | -0.11868400 | -0.27951100 | 2.54875000 |
| C | 0.68650000 | 0.14666400 | 1.49083500 |

| | | | |
|---|---|---|---|
| N | -1.97520100 | -3.06359800 | 1.00063200 |
| C | -2.93491400 | -3.18609700 | 0.13865300 |
| C | -3.47858100 | -4.59925800 | 0.18005700 |
| S | -3.27587400 | -2.09445300 | -1.22880200 |
| C | -5.05365800 | -1.92806400 | -1.06750000 |
| C | -5.58236900 | -1.18753200 | -0.00666200 |
| C | -6.96245200 | -1.03046000 | 0.10215300 |
| C | -7.80922200 | -1.59847600 | -0.85191700 |
| C | -7.27507800 | -2.32458000 | -1.91633900 |
| C | -5.89420700 | -2.49410900 | -2.02673400 |
| C | -2.26232800 | -5.57535600 | 0.35738900 |
| F | -4.16877500 | -4.91446300 | -0.97665400 |
| C | -1.01306500 | -4.85703000 | 0.77352900 |
| C | -4.51767800 | -4.80316700 | 1.28994100 |
| F | -4.93699100 | -6.08083000 | 1.32037700 |
| F | -5.60530800 | -4.04406800 | 1.09978700 |
| F | -4.02049200 | -4.51122000 | 2.49675800 |
| H | 1.23458600 | -0.17059400 | -0.57070200 |
| H | -0.35180600 | -2.05060500 | -0.89649200 |
| H | -1.62506500 | -1.69705000 | 3.18950400 |
| H | -0.04630600 | 0.20388800 | 3.51821300 |
| H | 1.38694400 | 0.96350700 | 1.63491300 |
| H | -4.91777100 | -0.74554200 | 0.73090900 |
| H | -7.37523500 | -0.46052000 | 0.92904200 |
| H | -8.88425400 | -1.47177100 | -0.76668600 |
| H | -7.93127800 | -2.76621200 | -2.66012800 |
| H | -5.47381600 | -3.06995700 | -2.84579000 |
| H | -2.12703000 | -6.02812100 | -0.62864200 |
| H | -2.52828400 | -6.37566200 | 1.05133500 |
| H | -0.37772200 | -4.51010400 | -0.03875400 |
| C | -0.33146200 | -5.06276500 | 2.03433900 |
| C | -0.95426200 | -5.63884600 | 3.16073500 |
| C | -0.27709200 | -5.73687800 | 4.37283300 |
| H | -1.97055800 | -6.01455700 | 3.08893600 |
| C | 1.65791900 | -4.67616900 | 3.39261700 |
| C | 1.02991900 | -5.25817000 | 4.49750400 |
| H | -0.77258200 | -6.18840400 | 5.22753300 |
| H | 2.67302900 | -4.29951400 | 3.47877700 |
| H | 1.55311900 | -5.33802000 | 5.44548200 |
| C | 0.98614500 | -4.57751600 | 2.17907100 |
| H | 1.47622500 | -4.12261100 | 1.32091200 |

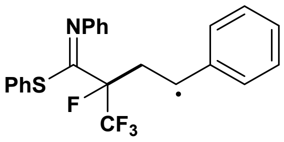

**C2**

| | | | |
|---|---|---|---|
| C | 0.30217800 | -2.11639400 | 0.03090500 |
| C | 0.54632600 | -2.97908300 | 1.09831900 |
| C | 0.52886500 | -2.48200600 | 2.40526400 |
| C | 0.29445800 | -1.12399100 | 2.64206700 |
| C | 0.03016000 | -0.27537100 | 1.56947900 |
| C | 0.03547700 | -0.76663300 | 0.26226900 |
| N | 0.82587000 | -3.31371800 | 3.50543400 |
| C | 0.13823900 | -4.32572400 | 3.82354900 |
| C | 0.71363300 | -5.19300900 | 4.95197100 |
| S | -1.32570600 | -4.90401900 | 2.94883200 |
| C | -2.50630300 | -4.98008100 | 4.29320500 |
| C | -2.73827000 | -3.85600100 | 5.08886800 |
| C | -3.69445400 | -3.91850100 | 6.10203100 |
| C | -4.43368900 | -5.08530100 | 6.29894200 |
| C | -4.21211700 | -6.19631200 | 5.48387800 |
| C | -3.24226900 | -6.15141900 | 4.48331500 |
| C | 1.92290400 | -5.98284100 | 4.42098400 |
| F | -0.23317600 | -6.09104300 | 5.40658900 |
| C | 2.50003300 | -6.93420400 | 5.41567100 |
| C | 3.78925000 | -6.83469200 | 6.00663100 |
| C | 4.20308000 | -7.83834900 | 6.92527200 |
| C | 5.44989300 | -7.79190500 | 7.52889800 |
| C | 6.33507400 | -6.74472100 | 7.24368900 |
| C | 5.94911200 | -5.74499000 | 6.34432600 |
| C | 1.05804000 | -4.33219500 | 6.18526500 |
| F | 1.19524400 | -5.10189800 | 7.27454200 |
| F | 0.08897600 | -3.44358300 | 6.45595600 |
| F | 2.19982500 | -3.65581500 | 6.02483500 |
| H | 0.31919200 | -2.50359900 | -0.98336600 |
| H | 0.76913900 | -4.02857400 | 0.92385800 |
| H | 0.30882400 | -0.75106000 | 3.66201500 |
| H | -0.17046900 | 0.77532000 | 1.75601000 |
| H | -0.15875500 | -0.09898100 | -0.57130400 |
| H | -2.17666600 | -2.94065800 | 4.92055900 |
| H | -3.86776400 | -3.04890200 | 6.72871200 |
| H | -5.18268600 | -5.12802700 | 7.08375400 |
| H | -4.78593000 | -7.10595900 | 5.63268400 |
| H | -3.05138400 | -7.02293000 | 3.86398800 |
| H | 2.65248600 | -5.26126100 | 4.04822400 |

| | | | |
|---|---|---|---|
| H | 1.54115400 | -6.53879400 | 3.55380900 |
| H | 1.87870800 | -7.77526900 | 5.71030200 |
| H | 3.51921200 | -8.65318400 | 7.14928300 |
| H | 5.73923000 | -8.57286800 | 8.22611500 |
| H | 7.31145800 | -6.70845100 | 7.71655600 |
| H | 6.62896900 | -4.92825400 | 6.11945000 |
| C | 4.70233500 | -5.78102200 | 5.73402400 |
| H | 4.43090900 | -4.98767000 | 5.04491800 |

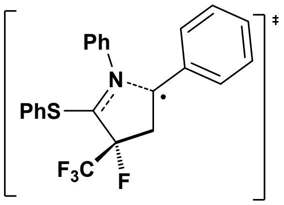


**TS3c**

| | | | |
|---|---|---|---|
| C | -0.78891500 | -1.04609400 | 0.04456400 |
| C | -1.69879400 | -2.09717000 | -0.05917200 |
| C | -2.27155900 | -2.63706900 | 1.09886700 |
| C | -1.90793400 | -2.13890300 | 2.35488400 |
| C | -1.00899900 | -1.07932700 | 2.45047600 |
| C | -0.44651600 | -0.53152100 | 1.29564600 |
| N | -3.13077700 | -3.75653500 | 1.07694300 |
| C | -4.17023600 | -3.92063400 | 0.31326300 |
| C | -4.67758700 | -5.33707400 | 0.46240100 |
| S | -4.70154600 | -2.89260400 | -1.03961300 |
| C | -6.44278600 | -2.73482000 | -0.63985900 |
| C | -6.83245300 | -2.01067600 | 0.49013600 |
| C | -8.18756200 | -1.85710800 | 0.77537800 |
| C | -9.14936500 | -2.41098700 | -0.07215700 |
| C | -8.75493500 | -3.12200100 | -1.20537400 |
| C | -7.39923900 | -3.28965700 | -1.49130100 |
| C | -3.42620900 | -6.26265100 | 0.40636300 |
| F | -5.56767200 | -5.68269200 | -0.53581200 |
| C | -2.21518000 | -5.57019800 | 0.95583900 |
| C | -1.04655600 | -5.29076600 | 0.15117400 |
| C | 0.16223800 | -4.94749400 | 0.79931300 |
| C | 1.28171200 | -4.56293000 | 0.07278100 |
| C | 1.22930500 | -4.50662500 | -1.32466800 |
| C | 0.04309300 | -4.84269200 | -1.98160200 |
| C | -5.46811000 | -5.55774300 | 1.75931400 |
| F | -5.95323600 | -6.81045600 | 1.80809500 |
| F | -6.51662000 | -4.72900000 | 1.85606200 |
| F | -4.70344700 | -5.38127600 | 2.84366400 |
| H | -0.34176400 | -0.63551700 | -0.85583900 |

| | | | |
|---|---|---|---|
| H | -1.93472600 | -2.51735500 | -1.03153600 |
| H | -2.33862700 | -2.59026400 | 3.24427800 |
| H | -0.73936500 | -0.68889800 | 3.42700800 |
| H | 0.26333600 | 0.28654600 | 1.37137200 |
| H | -6.07947000 | -1.57887400 | 1.14427500 |
| H | -8.49136200 | -1.29978600 | 1.65636200 |
| H | -10.20478900 | -2.28635200 | 0.15043100 |
| H | -9.50010900 | -3.55448900 | -1.86610200 |
| H | -7.08660800 | -3.85527700 | -2.36407400 |
| H | -3.30986900 | -6.51829800 | -0.64918600 |
| H | -3.62799000 | -7.18878000 | 0.95226900 |
| H | -2.03111500 | -5.69442200 | 2.01944800 |
| H | 0.20341800 | -4.98289600 | 1.88560300 |
| H | 2.19900400 | -4.30370900 | 0.59375800 |
| H | 2.10380400 | -4.20608800 | -1.89360600 |
| H | -0.00704700 | -4.79916000 | -3.06586700 |
| C | -1.08297900 | -5.22769400 | -1.25917000 |
| H | -1.99680200 | -5.46485200 | -1.79662700 |

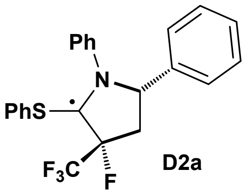


D2a

| | | | |
|---|---|---|---|
| C | 0.08578700 | -0.92396000 | 0.15227000 |
| C | 0.60105800 | 0.36913000 | 0.06583400 |
| C | 0.14322000 | 1.22792700 | -0.93505800 |
| C | -0.82995000 | 0.80278100 | -1.84295300 |
| C | -1.34722000 | -0.48757600 | -1.74506800 |
| C | -0.88977300 | -1.35249300 | -0.74889000 |
| N | 0.67440000 | 2.55269500 | -1.02568400 |
| C | -0.15722200 | 3.65099400 | -1.13697500 |
| C | 0.60528100 | 4.82135300 | -1.65643700 |
| S | -1.68197600 | 3.75649600 | -0.29996200 |
| C | 2.03064600 | 4.29726300 | -1.84760700 |
| F | 0.59479100 | 5.90871700 | -0.76842700 |
| C | 1.91267700 | 2.76663600 | -1.81193000 |
| C | 3.10410400 | 2.05846700 | -1.20973300 |
| C | 3.78054200 | 1.08230200 | -1.94170300 |
| C | 4.87120100 | 0.40912200 | -1.38507300 |
| C | 5.28495700 | 0.70622600 | -0.08829400 |
| C | 4.60830800 | 1.68119000 | 0.65108900 |
| C | 0.00189100 | 5.40581000 | -2.93461600 |
| F | 0.68237700 | 6.48262500 | -3.35887000 |
| F | -1.27290200 | 5.78686600 | -2.75770100 |

| | | | |
|---|---|---|---|
| F | 0.02071400 | 4.50147100 | -3.92658100 |
| H | 0.43929300 | -1.59098900 | 0.93263300 |
| H | 1.34187800 | 0.72205200 | 0.77747700 |
| H | -1.17563200 | 1.48751100 | -2.61351000 |
| H | -2.10595400 | -0.81800000 | -2.44801000 |
| H | -1.29500300 | -2.35702500 | -0.67390100 |
| H | 2.60657900 | 4.65814400 | -0.99131800 |
| H | 2.50904000 | 4.64923000 | -2.76350300 |
| H | 1.74148600 | 2.37943700 | -2.82595700 |
| H | 3.44983100 | 0.84435500 | -2.94988400 |
| H | 5.39051900 | -0.34890600 | -1.96380600 |
| H | 6.12966100 | 0.18189000 | 0.34849000 |
| H | 4.92488600 | 1.91357400 | 1.66360000 |
| C | 3.52540700 | 2.35396100 | 0.09235100 |
| H | 2.99382500 | 3.10374100 | 0.67521600 |
| C | -1.24391900 | 3.10167100 | 1.31545700 |
| C | -2.09754100 | 2.16703500 | 1.90400300 |
| C | -0.06986000 | 3.49553900 | 1.96341500 |
| C | -1.77752500 | 1.62995000 | 3.15201400 |
| H | -2.99704400 | 1.84801100 | 1.38394200 |
| C | 0.25274400 | 2.93863800 | 3.19904700 |
| H | 0.59038700 | 4.22327800 | 1.49801200 |
| C | -0.59973900 | 2.00661000 | 3.79679400 |
| H | -2.44267500 | 0.90338700 | 3.60907300 |
| H | 1.16975100 | 3.23746500 | 3.69868100 |
| H | -0.34443700 | 1.57609500 | 4.76023800 |

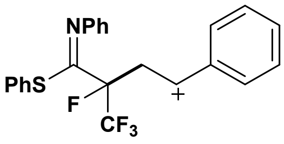


C3

| | | | |
|---|---|---|---|
| C | 0.91567500 | -0.44080800 | -0.02094400 |
| C | 1.17314700 | -1.27948500 | 1.05838100 |
| C | 1.16224800 | -0.75776900 | 2.35242300 |
| C | 0.91863900 | 0.59986700 | 2.56446300 |
| C | 0.63933200 | 1.42322000 | 1.48084300 |
| C | 0.63915700 | 0.90725100 | 0.18661500 |
| N | 1.46278300 | -1.55998700 | 3.47267000 |
| C | 0.80519000 | -2.58440300 | 3.79572300 |
| C | 1.36787700 | -3.38018400 | 4.98267700 |
| S | -0.60336400 | -3.25412100 | 2.90324100 |
| C | -1.80007500 | -3.40110800 | 4.22864400 |
| C | -2.15007500 | -2.27916200 | 4.97601900 |
| C | -3.11594800 | -2.39636100 | 5.97069900 |

| | | | |
|---|---|---|---|
| C | -3.74749700 | -3.61656800 | 6.19224400 |
| C | -3.40730400 | -4.72733900 | 5.42407300 |
| C | -2.42571100 | -4.62720400 | 4.44427700 |
| C | 2.70812200 | -4.01654000 | 4.58778100 |
| F | 0.50434300 | -4.37429200 | 5.36968100 |
| C | 3.21000200 | -4.95936800 | 5.60589100 |
| C | 4.51122000 | -5.10148100 | 6.04734800 |
| C | 4.77067900 | -6.13944800 | 6.99499100 |
| C | 6.04237600 | -6.32558300 | 7.48171100 |
| C | 7.06930100 | -5.48520500 | 7.03868300 |
| C | 6.84170200 | -4.45660700 | 6.11098100 |
| C | 1.54042800 | -2.49524000 | 6.22917200 |
| F | 1.84116000 | -3.27376000 | 7.27839000 |
| F | 0.42260300 | -1.83584300 | 6.52160300 |
| F | 2.52622600 | -1.61521700 | 6.08965000 |
| H | 0.92906300 | -0.84583100 | -1.02597800 |
| H | 1.40566200 | -2.32728200 | 0.90166500 |
| H | 0.94033800 | 0.98913000 | 3.57573600 |
| H | 0.43131500 | 2.47356800 | 1.64783500 |
| H | 0.43295800 | 1.55551500 | -0.65661900 |
| H | -1.66920300 | -1.32597500 | 4.78520500 |
| H | -3.38190300 | -1.52831200 | 6.56231900 |
| H | -4.50597800 | -3.70218500 | 6.96143800 |
| H | -3.89832600 | -5.67832200 | 5.59392400 |
| H | -2.14082000 | -5.49419500 | 3.85982900 |
| H | 3.41438000 | -3.24214400 | 4.29784200 |
| H | 2.50296600 | -4.64156100 | 3.70256800 |
| H | 2.47356000 | -5.64341800 | 6.02423000 |
| H | 3.94782200 | -6.76828400 | 7.31614400 |
| H | 6.25140100 | -7.10805200 | 8.19894800 |
| H | 8.07231300 | -5.62972900 | 7.42470000 |
| H | 7.66212800 | -3.82541300 | 5.79595500 |
| C | 5.57864200 | -4.25695700 | 5.61381000 |
| H | 5.39252900 | -3.46233800 | 4.90303000 |

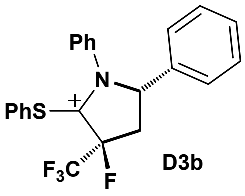


D3b

| | | | |
|---|---|---|---|
| C | 0.96610500 | -1.20501900 | 0.07379900 |
| C | 1.05524700 | 0.14342700 | 0.40177000 |
| C | 0.05698100 | 0.99579500 | -0.05041400 |
| C | -1.01347500 | 0.55829800 | -0.82087700 |
| C | -1.08467600 | -0.79246400 | -1.13998100 |

| | | | |
|---|---|---|---|
| C | -0.09880300 | -1.66894200 | -0.69355800 |
| N | 0.15249800 | 2.39888200 | 0.26658900 |
| C | -0.50308700 | 2.97330400 | 1.22069100 |
| C | -0.14812600 | 4.46772700 | 1.28020700 |
| S | -1.49488300 | 2.03506800 | 2.27133700 |
| C | 0.92563200 | 4.63093500 | 0.19620400 |
| F | 0.36650500 | 4.73873100 | 2.52025600 |
| C | 1.07013100 | 3.27952200 | -0.51594200 |
| C | -1.39067700 | 5.39108600 | 1.09209600 |
| F | -1.11408200 | 6.43776800 | 0.31768300 |
| F | -1.82257500 | 5.86558700 | 2.25201900 |
| F | -2.38840800 | 4.71383200 | 0.50995000 |
| H | 1.73040800 | -1.89050000 | 0.41844700 |
| H | 1.87377500 | 0.53096700 | 0.99742800 |
| H | -1.76348200 | 1.26479700 | -1.15865000 |
| H | -1.90870900 | -1.15734100 | -1.74054500 |
| H | -0.16030500 | -2.72053500 | -0.94689700 |
| H | 1.85019100 | 4.89063300 | 0.71064800 |
| H | 0.68367700 | 5.42447700 | -0.50410900 |
| C | -2.23444500 | 3.23950100 | 3.36740800 |
| C | -3.56171100 | 3.59868300 | 3.14469600 |
| C | -1.52507800 | 3.70047100 | 4.47394100 |
| C | -4.17561600 | 4.47522000 | 4.03191400 |
| H | -4.10061500 | 3.21111500 | 2.28844100 |
| C | -2.14966800 | 4.58360600 | 5.34568700 |
| H | -0.50335400 | 3.38438800 | 4.64329900 |
| C | -3.46836800 | 4.97197300 | 5.12257800 |
| H | -5.20492500 | 4.76960300 | 3.86742100 |
| H | -1.60711600 | 4.96160000 | 6.20364200 |
| H | -3.94987400 | 5.65848100 | 5.80896800 |
| C | 0.69000800 | 3.22940700 | -1.97778500 |
| C | 1.49802400 | 2.52807900 | -2.86899300 |
| C | -0.49087900 | 3.82021200 | -2.43069600 |
| C | 1.13469200 | 2.42353400 | -4.20924000 |
| H | 2.41068800 | 2.06276800 | -2.51158600 |
| C | -0.85473700 | 3.71221600 | -3.76704100 |
| H | -1.13098400 | 4.36356900 | -1.74313000 |
| C | -0.04158400 | 3.01369100 | -4.65789800 |
| H | 1.76952600 | 1.87987400 | -4.89881200 |
| H | -1.77195100 | 4.17289200 | -4.11426900 |
| H | -0.32673100 | 2.93166800 | -5.70026200 |
| H | 2.06822900 | 2.85924700 | -0.38350200 |

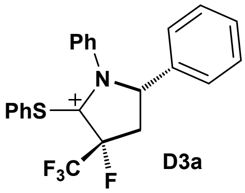

D3a

| | | | |
|---|---|---|---|
| C | -0.17366000 | -0.25066000 | 0.06915800 |
| C | 0.11493600 | 1.05971300 | 0.43294900 |
| C | -0.39014300 | 2.08587400 | -0.35556000 |
| C | -1.16189200 | 1.85240500 | -1.48673500 |
| C | -1.43728300 | 0.53535200 | -1.83660700 |
| C | -0.94419500 | -0.51039000 | -1.06122000 |
| N | -0.09471000 | 3.45580700 | -0.01556000 |
| C | -0.77313900 | 4.15687300 | 0.83651100 |
| C | -0.30898400 | 5.61399200 | 0.75575100 |
| S | -2.03884400 | 3.44744900 | 1.75841100 |
| C | 1.05646000 | 5.50405900 | 0.08783800 |
| F | -0.25707800 | 6.22844600 | 1.96887100 |
| C | 0.98836300 | 4.21357300 | -0.73331700 |
| C | 2.24932700 | 3.39212300 | -0.79807700 |
| C | 2.71264500 | 2.94728800 | -2.03369000 |
| C | 3.85968600 | 2.16162500 | -2.10773400 |
| C | 4.53735700 | 1.81078900 | -0.94520200 |
| C | 4.06924800 | 2.24550300 | 0.29376400 |
| C | -1.29892600 | 6.42070600 | -0.10157400 |
| F | -0.85951700 | 7.66449500 | -0.25264600 |
| F | -2.50705200 | 6.47354200 | 0.45148400 |
| F | -1.41653200 | 5.86028600 | -1.30839500 |
| H | 0.20830600 | -1.06756200 | 0.66884600 |
| H | 0.71376500 | 1.28727700 | 1.30710100 |
| H | -1.53499100 | 2.68652700 | -2.07073600 |
| H | -2.03691200 | 0.32926300 | -2.71459700 |
| H | -1.16153400 | -1.53467900 | -1.33935200 |
| H | 1.79129300 | 5.41684100 | 0.88923900 |
| H | 1.29185200 | 6.37082400 | -0.52655000 |
| H | 0.61372000 | 4.39878000 | -1.74188200 |
| H | 2.17410000 | 3.21719400 | -2.93604900 |
| H | 4.21929000 | 1.82230500 | -3.07185900 |
| H | 5.42862800 | 1.19674200 | -1.00107100 |
| H | 4.59307000 | 1.96797000 | 1.20078800 |
| C | 2.92900900 | 3.03515900 | 0.36846600 |
| H | 2.56060100 | 3.36144800 | 1.33658600 |
| C | -2.47466000 | 4.69200600 | 2.97312700 |
| C | -3.68225300 | 5.36843500 | 2.83294700 |
| C | -1.67685600 | 4.84218300 | 4.10533400 |

| | | | |
|---|---|---|---|
| C | -4.07938600 | 6.24405100 | 3.83826000 |
| H | -4.29757300 | 5.22115300 | 1.95399300 |
| C | -2.08176500 | 5.72776000 | 5.09560100 |
| H | -0.75330600 | 4.28455700 | 4.20480300 |
| C | -3.27942400 | 6.42668500 | 4.96159300 |
| H | -5.01489400 | 6.78094400 | 3.73967100 |
| H | -1.46519000 | 5.86426200 | 5.97563200 |
| H | -3.59340300 | 7.11074200 | 5.74105100 |

# VI. X-Ray Crystal Structure Determinations

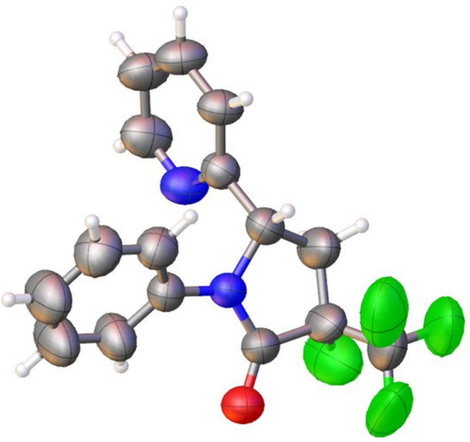

Single crystal of **3a** (CCDC 2209201) was obtained by recrystallization from a mixed solvent of acetone/ petroleum ether at room temperature (evaporation in air). The X-ray single-crystal determination was performed on a Bruker APEX II X-ray single crystal diffractometer.

```
Bond precision:   C-C = 0.0059 A          Wavelength=1.54184

Cell:             a=15.5988(5)     b=8.2297(4)         c=12.1249(6)
                  alpha=90         beta=101.812(4)     gamma=90
Temperature:      295 K

                 Calculated                   Reported
Volume           1523.56(12)                  1523.56(12)
Space group      P 21/c                       P 1 21/c 1
Hall group       -P 2ybc                      -P 2ybc
Moiety formula   C16 H12 F4 N2 O              C16 H12 F4 N2 O
Sum formula      C16 H12 F4 N2 O              C16 H12 F4 N2 O
Mr               324.28                       324.28
Dx,g cm-3        1.414                        1.414
Z                4                            4
Mu (mm-1)        1.073                        1.073
F000             664.0                        664.0
F000'            666.66
h,k,lmax         19,10,15                     19,10,15
Nref             3216                         3031
Tmin,Tmax        0.975,0.979                  0.809,1.000
Tmin'            0.968

Correction method= # Reported T Limits: Tmin=0.809 Tmax=1.000
AbsCorr = MULTI-SCAN

Data completeness= 0.942           Theta(max)= 76.552

                                                   wR2(reflections)=
R(reflections)= 0.0722( 1541)                      0.2354( 3031)

S = 1.307                 Npar= 208
```

# VII. Characterization Data of the Products

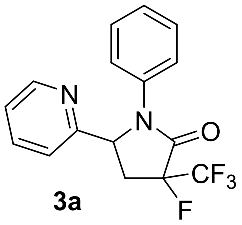


**3a**

Chemical Formula: $C_{16}H_{12}F_4N_2O$

**3-fluoro-1-phenyl-5-(pyridin-2-yl)-3-(trifluoromethyl)pyrrolidin-2-one (3a)**: yellow solid (mp = 118.3-118.8 ℃), 26.8 mg, (Yield: 83%).

**$^1$H NMR** (400 MHz, Chloroform-*d*) δ (ppm) = 8.60 – 8.54 (m, 1H), 7.60 (td, *J* = 7.7, 1.8 Hz, 1H), 7.46 – 7.40 (m, 2H), 7.32 – 7.26 (m, 2H), 7.22 – 7.13 (m, 3H), 5.40 (ddd, *J* = 7.7, 5.6, 3.8 Hz, 1H), 3.22 (td, *J* = 14.8, 7.7 Hz, 1H), 2.77 – 2.62 (m, 1H).

**$^{13}$C NMR** (101 MHz, Chloroform-*d*) δ (ppm) = 163.1 (d, *J* = 22.3 Hz), 157.5, 150.1, 137.3, 136.3, 129.0, 126.7, 123.5, 122.9, 121.4, 121.1 (qd, *J* = 282.0, 31.8 Hz), 92.3 (dq, *J* = 199.8, 34.4 Hz), 60.6, 34.1 (d, *J* = 20.5 Hz).
**$^{19}$F NMR** (376 MHz, Chloroform-*d*) δ (ppm) = -79.88 (d, *J* = 10.5 Hz, 3F), -167.16 (q, *J* = 10.6 Hz, 1F).
**HRMS (ESI) m/z:** $[M + H]^+$ Calcd for $C_{16}H_{13}F_4N_2O$ 325.0959; Found 325.0958.

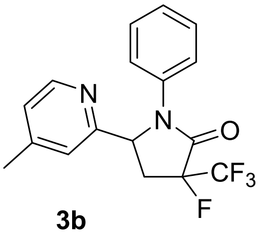


**3b**

Chemical Formula: $C_{17}H_{14}F_4N_2O$

**3-fluoro-5-(4-methylpyridin-2-yl)-1-phenyl-3-(trifluoromethyl)pyrrolidin-2-one (3b)**: yellow solid (mp = 125.6-126.6 °C), 27.6 mg, (Yield: 81%).
**$^{1}$H NMR** (400 MHz, Chloroform-*d*) δ (ppm) = 8.41 (d, *J* = 5.0 Hz, 1H), 7.48 – 7.40 (m, 2H), 7.32 – 7.25 (m, 2H), 7.20 – 7.11 (m, 1H), 7.04 – 6.95 (m, 2H), 5.35 (ddd, *J* = 7.8, 5.5, 3.9 Hz, 1H), 3.19 (td, *J* = 14.9, 7.8 Hz, 1H), 2.66 (dddd, *J* = 25.7, 15.0, 5.5, 1.2 Hz, 1H), 2.26 (s, 3H).
**$^{13}$C NMR** (101 MHz, Chloroform-*d*) δ (ppm) = 163.1 (d, *J* = 22.1 Hz), 157.4, 149.7, 136.4, 129.0, 126.6, 124.5, 122.7, 122.0 (qd, *J* = 283.3, 32.3 Hz), 92.4 (dq, *J* = 201.0, 34.3 Hz), 60.6, 34.1 (d, *J* = 20.3 Hz), 21.0.
**$^{19}$F NMR** (376 MHz, Chloroform-*d*) δ (ppm) = -79.91 (d, *J* = 10.4 Hz, 3F), -167.10 (q, *J* = 10.7 Hz, 1F).
**HRMS (ESI) m/z:** $[M + H]^+$ Calcd for $C_{17}H_{15}F_4N_2O$ 339.1115; Found 339.1112.

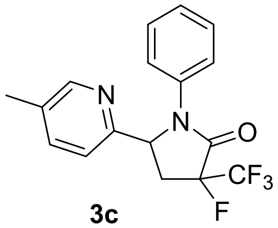


**3c**

Chemical Formula: $C_{17}H_{14}F_4N_2O$

**3-fluoro-5-(5-methylpyridin-2-yl)-1-phenyl-3-(trifluoromethyl)pyrrolidin-2-one (3c)**: white solid (mp = 105.6-127.3 °C), 27.6 mg, (Yield: 81%).
**$^{1}$H NMR** (400 MHz, Chloroform-*d*) δ (ppm) = 8.42 – 8.35 (m, 1H), 7.45 – 7.37 (m, 3H), 7.32 – 7.23 (m, 2H), 7.19 – 7.11 (m, 1H), 7.05 (d, *J* = 7.9 Hz, 1H), 5.36 (ddd, *J* = 7.7, 5.7, 3.9 Hz, 1H), 3.19 (td, *J* = 14.6, 7.7 Hz, 1H), 2.67 (dddd, *J* = 25.6, 15.0, 5.7, 1.3 Hz, 1H), 2.28 (s, 3H).
**$^{13}$C NMR** (101 MHz, Chloroform-*d*) δ (ppm) = 163.1 (d, *J* = 22.3 Hz), 154.6, 150.5, 137.7, 136.4, 133.2, 129.0, 126.7, 122.9, 121.9 (qd, *J* = 284.7, 31.8 Hz), 120.8, 92.4 (dq, *J* = 200.0, 34.2 Hz), 60.4, 34.2 (d, *J* = 20.2 Hz), 18.1.
**$^{19}$F NMR** (376 MHz, Chloroform-*d*) δ (ppm) = -79.89 (d, *J* = 10.7 Hz, 3F), -167.21 (q, *J* = 10.9 Hz, 1F).
**HRMS (ESI) m/z:** $[M + H]^+$ Calcd for $C_{17}H_{15}F_4N_2O$ 339.1115; Found 339.1114.

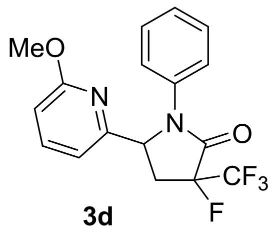

**3d**

Chemical Formula: $C_{17}H_{14}F_4N_2O_2$

**3-fluoro-5-(6-methoxypyridin-2-yl)-1-phenyl-3-(trifluoromethyl)pyrrolidin-2-one (3d)**: yellow dense oil, 25.3 mg, (Yield: 71%).

**$^{1}$H NMR** (400 MHz, Chloroform-*d*) δ (ppm) = 7.44 (dd, *J* = 8.4, 7.2 Hz, 1H), 7.39 – 7.34 (m, 2H), 7.32 – 7.24 (m, 2H), 7.19 – 7.12 (m, 1H), 6.72 (dd, *J* = 7.2, 0.7 Hz, 1H), 6.61 (dd, *J* = 8.3, 0.7 Hz, 1H), 5.22 (ddd, *J* = 7.8, 5.2, 4.2 Hz, 1H), 3.89 (s, 3H), 3.13 (td, *J* = 14.8, 7.7 Hz, 1H), 2.72 (dddd, *J* = 26.2, 15.0, 5.3, 1.2 Hz, 1H).

**$^{13}$C NMR** (101 MHz, Chloroform-*d*) δ (ppm) = 164.4, 163.2 (d, *J* = 22.5 Hz), 154.4, 139.2, 136.5, 128.9, 126.8, 123.4, 122.0 (qd, *J* = 283.5, 32.0 Hz), 114.8, 111.1, 92.2 (dq, *J* = 200.1, 34.0 Hz), 60.3, 53.5, 33.5 (d, *J* = 20.7 Hz).

**$^{19}$F NMR** (376 MHz, Chloroform-*d*) δ (ppm) = -80.01 (d, *J* = 10.7 Hz, 3F), -167.27 (q, *J* = 10.6 Hz, 1F).

**HRMS (ESI) m/z:** $[M + H]^+$ Calcd for $C_{17}H_{15}F_4N_2O_2$ 355.1064; Found 355.1063.

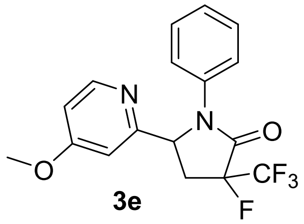

**3e**

Chemical Formula: $C_{17}H_{14}F_4N_2O_2$

**3-fluoro-5-(4-methoxypyridin-2-yl)-1-phenyl-3-(trifluoromethyl)pyrrolidin-2-one (3e)**: yellow solid (mp = 104.2-124.7 °C), 27.5 mg, (Yield: 77%).

**$^{1}$H NMR** (400 MHz, Chloroform-*d*) δ (ppm) = 8.38 (d, *J* = 5.6 Hz, 1H), 7.45 (d, *J* = 8.0 Hz, 2H), 7.30 (t, *J* = 7.8 Hz, 2H), 7.16 (t, *J* = 7.4 Hz, 1H), 6.74 – 6.63 (m, 2H), 5.35 (dt, *J* = 8.4, 4.7 Hz, 1H), 3.74 (s, 3H), 3.20 (td, *J* = 15.1, 7.8 Hz, 1H), 2.66 (ddd, *J* = 25.7, 15.1, 5.5 Hz, 1H).

**$^{13}$C NMR** (101 MHz, Chloroform-*d*) δ (ppm) = 166.6, 163.1 (d, *J* = 22.3 Hz), 159.3, 151.3, 136.4, 129.0, 126.7, 122.7, 121.8 (qd, *J* = 283.4, 31.8 Hz), 109.2, 107.7 (d, *J* = 1.3 Hz), 92.3 (dq, *J* = 199.8, 34.3 Hz), 60.5, 55.2, 34.0 (d, *J* = 20.4 Hz).

**$^{19}$F NMR** (376 MHz, Chloroform-*d*) δ (ppm) = -79.89 (d, *J* = 10.8 Hz, 3F), -166.93 (q, *J* = 10.9 Hz, 1F).

**HRMS (ESI) m/z:** $[M + H]^+$ Calcd for $C_{17}H_{15}F_4N_2O_2$ 355.1064; Found 355.1063.

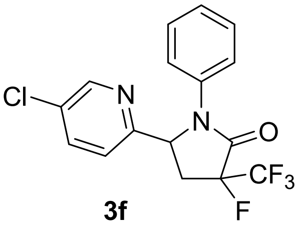

**3f**

Chemical Formula: $C_{16}H_{11}ClF_4N_2O$

**5-(5-chloropyridin-2-yl)-3-fluoro-1-phenyl-3-(trifluoromethyl)pyrrolidin-2-one (3f)**: white solid (mp = 143.9-144.7 °C), 22.9 mg, (Yield: 64%).

**$^1$H NMR** (400 MHz, Chloroform-*d*) δ (ppm) = 8.53 (d, *J* = 2.4 Hz, 1H), 7.58 (dd, *J* = 8.4, 2.4 Hz, 1H), 7.43 – 7.37 (m, 2H), 7.34 – 7.27 (m, 2H), 7.22 – 7.14 (m, 1H), 7.12 (d, *J* = 8.4 Hz, 1H), 5.39 (ddd, *J* = 7.8, 5.5, 3.8 Hz, 1H), 3.21 (td, *J* = 15.0, 7.7 Hz, 1H), 2.66 (dddd, *J* = 25.5, 15.1, 5.5, 1.2 Hz, 1H).

**$^{13}$C NMR** (101 MHz, Chloroform-*d*) δ (ppm) = 163.0 (d, *J* = 22.3 Hz), 155.6, 149.1, 137.0, 136.1, 131.9, 129.2, 126.9, 122.9, 122.2, 121.8 (qd, *J* = 283.4, 31.8 Hz), 92.2 (dq, *J* = 200.2, 34.2 Hz), 60.0, 34.0 (d, *J* = 20.5 Hz).

**$^{19}$F NMR** (376 MHz, Chloroform-*d*) δ (ppm) = -79.86 (d, *J* = 10.6 Hz, 3F), -167.02 (q, *J* = 10.8 Hz, 1F).

**HRMS (ESI) m/z:** $[M + H]^+$ Calcd for $C_{16}H_{12}ClF_4N_2O$ 359.0569; Found 359.0566.

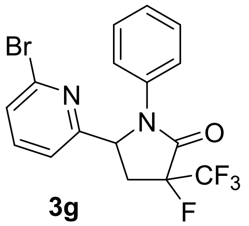


**3g**

Chemical Formula: $C_{16}H_{11}BrF_4N_2O$

**5-(6-bromopyridin-2-yl)-3-fluoro-1-phenyl-3-(trifluoromethyl)pyrrolidin-2-one (3g)**: dense yellow oil, 26.3 mg, (Yield: 65%).

**$^1$H NMR** (400 MHz, Chloroform-*d*) δ (ppm) = 7.50 – 7.42 (m, 3H), 7.41 – 7.36 (m, 1H), 7.32 (dd, *J* = 8.7, 7.2 Hz, 2H), 7.22 – 7.15 (m, 1H), 7.11 (d, *J* = 7.5 Hz, 1H), 5.40 (td, *J* = 4.9, 2.5 Hz, 1H), 3.22 (ddd, *J* = 17.0, 15.1, 7.9 Hz, 1H), 2.72 – 2.57 (m, 1H).

**$^{13}$C NMR** (101 MHz, Chloroform-*d*) δ (ppm) = 163.0 (d, *J* = 22.5 Hz), 159.2, 142.3, 139.6, 136.1, 129.2, 128.0, 126.9, 122.6, 121.7 (qd, *J* = 283.3, 31.5 Hz), 119.8, 92.3 (dq, *J* = 199.9, 34.3 Hz), 60.0, 34.0 (d, *J* = 20.6 Hz).

**$^{19}$F NMR** (376 MHz, Chloroform-*d*) δ (ppm) = -79.79 (d, *J* = 10.3 Hz, 3F), -166.94 (q, *J* = 10.7 Hz, 1F).

**HRMS (ESI) m/z:** $[M + H]^+$ Calcd for $C_{16}H_{12}BrF_4N_2O$ 403.0064; Found 403.0062.

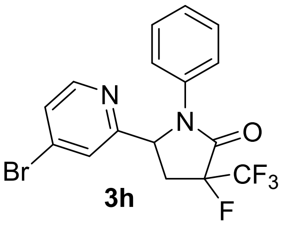


**3h**

Chemical Formula: $C_{16}H_{11}BrF_4N_2O$

**5-(4-bromopyridin-2-yl)-3-fluoro-1-phenyl-3-(trifluoromethyl)pyrrolidin-2-one (3h)**: yellow solid (mp = 82.1-82.7 °C), 33.2 mg, (Yield: 82%).

**$^1$H NMR** (400 MHz, Chloroform-*d*) δ (ppm) = 8.38 (dd, *J* = 5.1, 1.4 Hz, 1H), 7.44 – 7.27 (m, 6H), 7.22 – 7.12 (m, 1H), 5.36 (ddd, *J* = 7.7, 5.4, 3.8 Hz, 1H), 3.20 (td, *J* = 15.0, 7.8 Hz, 1H), 2.67 (dddd, *J* = 25.4, 15.1, 5.5, 1.3 Hz, 1H).

**$^{13}$C NMR** (101 MHz, Chloroform-*d*) δ (ppm) = 163.0 (d, *J* = 22.2 Hz), 159.0, 150.8, 136.0, 133.9, 129.2, 127.0, 126.9, 125.0, 122.9, 121.8 (qd, *J* = 283.4, 31.8 Hz), 92.1 (dq, *J* = 200.3, 34.2 Hz), 60.1, 33.8 (d, *J* = 20.7 Hz).
**$^{19}$F NMR** (376 MHz, Chloroform-*d*) δ (ppm) = -79.88 (d, *J* = 10.7 Hz, 3F), -166.98 (dt, *J* = 13.2, 10.6 Hz, 1F).
**HRMS (ESI) m/z:** $[M + H]^+$ Calcd for $C_{16}H_{12}BrF_4N_2O$ 403.0064; Found 403.0062.

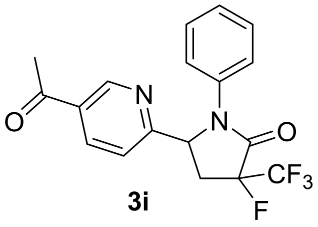

**3i**

Chemical Formula: $C_{18}H_{14}F_4N_2O_2$

**5-(5-acetylpyridin-2-yl)-3-fluoro-1-phenyl-3-(trifluoromethyl)pyrrolidin-2-one (3i)**: yellow solid (mp = 96.9-98.7 °C), 23.9 mg, (Yield: 65%).
**$^1$H NMR** (400 MHz, Chloroform-*d*) δ (ppm) = 9.11 (d, *J* = 2.2 Hz, 1H), 8.14 (dd, *J* = 8.2, 2.2 Hz, 1H), 7.47 – 7.39 (m, 2H), 7.34 – 7.25 (m, 3H), 7.22 – 7.13 (m, 1H), 5.49 (ddd, *J* = 8.5, 5.3, 3.7 Hz, 1H), 3.24 (td, *J* = 15.4, 7.9 Hz, 1H), 2.74 – 2.55 (m, 4H).
**$^{13}$C NMR** (101 MHz, Chloroform-*d*) δ (ppm) = 195.9, 163.0 (d, *J* = 22.2 Hz), 161.8, 150.2, 136.9, 136.1, 129.2, 126.9, 122.6, 121.7 (qd, *J* = 283.4, 31.6 Hz), 121.2, 92.2 (dq, *J* = 200.1, 34.3 Hz), 60.3, 33.8 (d, *J* = 20.7 Hz), 26.7.
**$^{19}$F NMR** (376 MHz, Chloroform-*d*) δ (ppm) = -79.84 (d, *J* = 10.7 Hz, 3F), -166.85 (q, *J* = 10.8 Hz, 1F).
**HRMS (ESI) m/z:** $[M + H]^+$ Calcd for $C_{18}H_{15}F_4N_2O_2$ 367.1064; Found 367.1062.

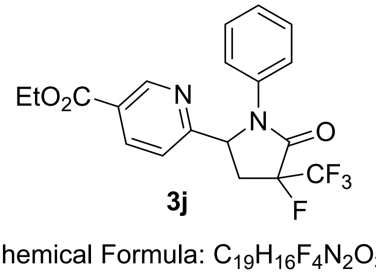

**3j**

Chemical Formula: $C_{19}H_{16}F_4N_2O_3$

**ethyl 6-(4-fluoro-5-oxo-1-phenyl-4-(trifluoromethyl)pyrrolidin-2-yl)nicotinate (3j)**: dense yellow oil, 25.9 mg, (Yield: 64%).
**$^1$H NMR** (400 MHz, Chloroform-*d*) δ (ppm) = 9.17 (d, *J* = 2.1 Hz, 1H), 8.21 (dd, *J* = 8.2, 2.2 Hz, 1H), 7.47 – 7.40 (m, 2H), 7.33 – 7.22 (m, 3H), 7.20 – 7.13 (m, 1H), 5.52 – 5.44 (m, 1H), 4.39 (q, *J* = 7.1 Hz, 2H), 3.24 (td, *J* = 15.3, 7.8 Hz, 1H), 2.67 (ddd, *J* = 25.3, 15.1, 5.4 Hz, 1H), 1.38 (t, *J* = 7.1 Hz, 3H).
**$^{13}$C NMR** (101 MHz, Chloroform-*d*) δ (ppm) = 164.5, 163.0 (d, *J* = 22.2 Hz), 161.7, 151.2, 138.4, 136.1, 129.2, 126.9, 126.1, 122.6, 121.8 (qd, *J* = 283.3, 31.7 Hz), 120.9, 92.2 (dq, *J* = 200.2, 34.2 Hz), 61.6, 60.4, 33.9 (d, *J* = 20.7 Hz), 14.2.
**$^{19}$F NMR** (376 MHz, Chloroform-*d*) δ (ppm) = -79.84 (d, *J* = 10.6 Hz, 3F), -166.91 (q, *J* = 10.8 Hz, 1F).
**HRMS (ESI) m/z:** $[M + H]^+$ Calcd for $C_{19}H_{17}F_4N_2O_3$ 397.1170; Found 397.1168.

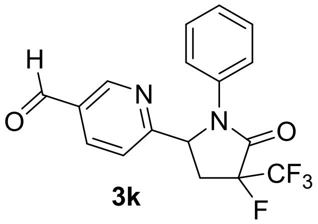

**3k**

Chemical Formula: $C_{17}H_{12}F_4N_2O_2$

**6-(4-fluoro-5-oxo-1-phenyl-4-(trifluoromethyl)pyrrolidin-2-yl)nicotinaldehyde (3k)**: dense brown oil, 15.9 mg, (Yield: 45%).

**$^1$H NMR** (400 MHz, Chloroform-*d*) δ (ppm) = 10.06 (s, 1H), 9.04 (dd, *J* = 2.2, 0.8 Hz, 1H), 8.09 (dd, *J* = 8.1, 2.2 Hz, 1H), 7.47 – 7.40 (m, 2H), 7.36 (d, *J* = 8.1 Hz, 1H), 7.33 – 7.25 (m, 2H), 7.21 – 7.13 (m, 1H), 5.51 (ddd, *J* = 8.0, 5.3, 3.6 Hz, 1H), 3.26 (ddd, *J* = 16.1, 15.1, 7.9 Hz, 1H), 2.75 – 2.61 (m, 1H).

**$^{13}$C NMR** (101 MHz, Chloroform-*d*) δ (ppm) = 189.7, 163.3, 163.0 (d, *J* = 22.5 Hz), 152.1, 137.4, 136.1, 131.0, 129.2, 127.0, 122.6, 121.7, 121.7 (qd, *J* = 284.4, 31.8 Hz), 92.2 (dq, *J* = 200.0, 34.3 Hz), 60.4, 33.8 (d, *J* = 20.8 Hz).

**$^{19}$F NMR** (376 MHz, Chloroform-*d*) δ (ppm) = -79.81 (d, *J* = 10.5 Hz, 3F), -166.82 (q, *J* = 10.4 Hz, 1F).

**HRMS (ESI) m/z:** $[M + H]^+$ Calcd for $C_{17}H_{13}F_4N_2O_2$ 353.0908; Found 353.0907.

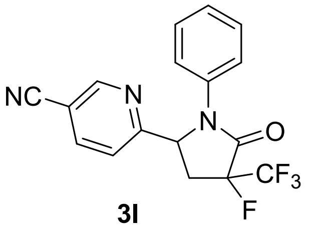

**3l**

Chemical Formula: $C_{17}H_{11}F_4N_3O$

**6-(4-fluoro-5-oxo-1-phenyl-4-(trifluoromethyl)pyrrolidin-2-yl)nicotinonitrile (3l)**: yellow solid (mp = 157.1-157.9 °C), 22.9 mg, (Yield: 65%).

**$^1$H NMR** (400 MHz, Chloroform-*d*) δ (ppm) = 8.85 (d, *J* = 2.0 Hz, 1H), 7.89 (dd, *J* = 8.2, 2.1 Hz, 1H), 7.45 – 7.38 (m, 2H), 7.36 – 7.28 (m, 3H), 7.23 – 7.16 (m, 1H), 5.49 (ddd, *J* = 8.3, 5.1, 3.6 Hz, 1H), 3.25 (ddd, *J* = 16.8, 15.1, 8.0 Hz, 1H), 2.72 – 2.57 (m, 1H).

**$^{13}$C NMR** (101 MHz, Chloroform-*d*) δ (ppm) = 162.9 (d, *J* = 22.0 Hz), 161.9, 152.7, 140.6, 135.9, 129.3, 127.1, 122.5, 121.6 (qd, *J* = 283.2, 31.5 Hz), 121.3, 115.9, 109.8, 92.1 (dq, *J* = 200.1, 34.5 Hz), 60.2, 33.7 (d, *J* = 20.7 Hz).

**$^{19}$F NMR** (376 MHz, Chloroform-*d*) δ (ppm) = -79.79 (d, *J* = 10.4 Hz, 3F), -166.60 (q, *J* = 10.6 Hz, 1F).

**HRMS (ESI) m/z:** $[M + H]^+$ Calcd for $C_{17}H_{12}F_4N_3O$ 350.0911; Found 350.0908.

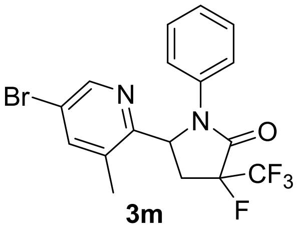

**3m**

Chemical Formula: $C_{17}H_{13}BrF_4N_2O$

**5-(5-bromo-3-methylpyridin-2-yl)-3-fluoro-1-phenyl-3-(trifluoromethyl)pyrrolidin-2-one (3m)**: yellow solid (mp = 110.6-111.1 °C), 26.8 mg, (Yield: 63%).

**$^1$H NMR** (400 MHz, Chloroform-*d*) δ (ppm) = 8.47 (d, *J* = 2.2 Hz, 1H), 7.53 (d, *J* = 2.2 Hz, 1H), 7.30 – 7.14 (m, 5H), 5.46 (td, *J* = 6.9, 4.2 Hz, 1H), 3.12 (ddd, *J* = 14.7, 9.3, 7.3 Hz, 1H), 2.84 – 2.69 (m, 1H), 2.26 (s, 3H).

**$^{13}$C NMR** (101 MHz, Chloroform-*d*) δ (ppm) = 163.0 (d, *J* = 22.6 Hz), 152.8, 149.0, 140.9, 136.3, 133.1, 129.0, 127.3, 124.1, 122.1 (qd, *J* = 283.9, 32.3 Hz), 120.2, 91.9 (dq, *J* = 201.5, 33.8 Hz), 56.5, 32.8 (d, *J* = 20.8 Hz), 17.7.

**$^{19}$F NMR** (376 MHz, Chloroform-*d*) δ (ppm) = -80.06 (d, *J* = 10.9 Hz, 3F), -168.42 (q, *J* = 10.8 Hz, 1F).

**HRMS (ESI) m/z:** $[M + H]^+$ Calcd for $C_{17}H_{14}BrF_4N_2O$ 417.0220; Found 417.0219.

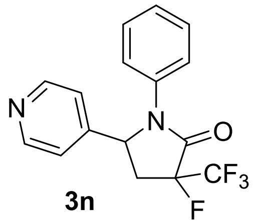


**3n**

Chemical Formula: $C_{16}H_{12}F_4N_2O$

**3-fluoro-1-phenyl-5-(pyridin-4-yl)-3-(trifluoromethyl)pyrrolidin-2-one (3n)**: yellow solid (mp = 156.9-157.7 °C), 24.4 mg, (Yield: 75%).

**$^1$H NMR** (400 MHz, Chloroform-*d*) δ (ppm) = 8.60 – 8.55 (m, 2H), 7.42 – 7.35 (m, 2H), 7.35 – 7.27 (m, 2H), 7.21 – 7.14 (m, 3H), 5.31 – 5.24 (m, 1H), 3.26 (td, *J* = 15.3, 7.9 Hz, 1H), 2.50 – 2.36 (m, 1H).

**$^{13}$C NMR** (101 MHz, Chloroform-*d*) δ (ppm) = 162.9 (d, *J* = 22.2 Hz), 150.8, 147.6, 135.7, 129.2, 127.1, 122.7, 121.6 (qd, *J* = 283.5, 31.6 Hz), 121.2, 92.0 (dq, *J* = 200.1, 34.4 Hz), 58.3, 35.3 (d, *J* = 20.5 Hz).

**$^{19}$F NMR** (376 MHz, Chloroform-*d*) δ (ppm) = -79.78 (d, *J* = 10.4 Hz, 3F), -165.85 (q, *J* = 10.5 Hz, 1F).

**HRMS (ESI) m/z:** $[M + H]^+$ Calcd for $C_{16}H_{13}F_4N_2O$ 325.0959; Found 325.0956.

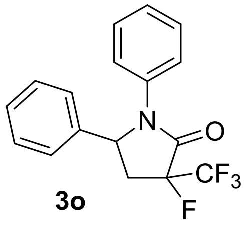


**3o**

Chemical Formula: $C_{17}H_{13}F_4NO$

**3-fluoro-1,5-diphenyl-3-(trifluoromethyl)pyrrolidin-2-one (3o)**: white solid (mp = 106.9-107.6 °C), 16.5 mg, (Yield: 51%).

**$^1$H NMR** (400 MHz, Chloroform-*d*) δ (ppm) = 7.39 – 7.34 (m, 2H), 7.34 – 7.21 (m, 7H), 7.16 – 7.11 (m, 1H), 5.23 (ddd, *J* = 7.6, 6.1, 4.1 Hz, 1H), 3.23 (ddd, *J* = 15.2, 12.8, 7.6 Hz, 1H), 2.47 (dddd, *J* = 26.0, 15.2, 6.1, 1.3 Hz, 1H).

**$^{13}$C NMR** (101 MHz, Chloroform-*d*) δ (ppm) = 163.1 (d, *J* = 22.3 Hz), 138.6, 136.2, 129.2, 128.9, 128.7, 126.7, 126.5, 123.3, 121.9 (qd, *J* = 283.6, 31.9 Hz), 92.2 (dq, *J* = 199.8, 34.2 Hz), 59.5 (d, *J* = 2.4 Hz), 36.2 (d, *J* = 20.0 Hz).

**$^{19}$F NMR** (376 MHz, Chloroform-*d*) δ (ppm) = -79.85 (d, *J* = 10.7 Hz, 3F), -166.37 (q, *J* = 10.9 Hz, 1F).
**HRMS (ESI) m/z:** $[M + H]^+$ Calcd for $C_{17}H_{14}F_4NO$ 324.1006; Found 324.1005.

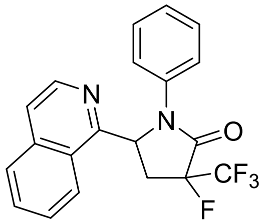

**3p**

Chemical Formula: $C_{20}H_{14}F_4N_2O$

**3-fluoro-5-(isoquinolin-1-yl)-1-phenyl-3-(trifluoromethyl)pyrrolidin-2-one (3p)**: white solid (mp = 101.4-102.6 °C), 15.9 mg, (Yield: 42%).
**$^1$H NMR** (400 MHz, Chloroform-*d*) δ (ppm) = 8.47 (d, *J* = 5.6 Hz, 1H), 8.14 (d, *J* = 8.4 Hz, 1H), 7.90 – 7.82 (m, 1H), 7.69 (dddd, *J* = 21.8, 8.3, 6.9, 1.3 Hz, 2H), 7.59 (d, *J* = 5.6 Hz, 1H), 7.37 (d, *J* = 8.1 Hz, 2H), 7.17 (dd, *J* = 8.6, 7.2 Hz, 2H), 7.09 – 7.01 (m, 1H), 6.20 (q, *J* = 6.6, 5.9 Hz, 1H), 3.32 (ddd, *J* = 14.9, 11.3, 7.6 Hz, 1H), 2.96 – 2.80 (m, 1H).
**$^{13}$C NMR** (101 MHz, Chloroform-*d*) δ (ppm) = 163.1 (d, *J* = 22.6 Hz), 155.2, 141.9, 136.7, 136.6, 130.5, 128.8, 128.3, 128.2, 126.7, 126.0, 123.3, 122.5, 122.1 (qd, *J* = 283.9, 30.9 Hz), 121.5, 92.1 (dq, *J* = 200.5, 34.0 Hz), 58.4, 33.7 (d, *J* = 20.9 Hz).
**$^{19}$F NMR** (376 MHz, Chloroform-*d*) δ (ppm) = -79.92 (d, *J* = 10.7 Hz, 3F), -167.98 (s, 1F).
**HRMS (ESI) m/z:** $[M + H]^+$ Calcd for $C_{20}H_{15}F_4N_2O$ 375.1115; Found 375.1114.

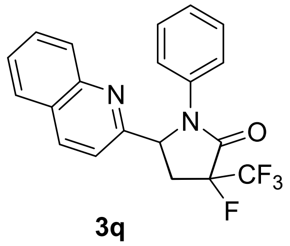

**3q**

Chemical Formula: $C_{20}H_{14}F_4N_2O$

**3-fluoro-1-phenyl-5-(quinolin-2-yl)-3-(trifluoromethyl)pyrrolidin-2-one (3q)**: brown solid (mp = 151.3-152.1 °C), 27.9 mg, (Yield: 74%).
**$^1$H NMR** (400 MHz, Chloroform-*d*) δ (ppm) = 8.13 – 8.02 (m, 2H), 7.80 – 7.69 (m, 2H), 7.58 – 7.48 (m, 3H), 7.31 – 7.21 (m, 3H), 7.14 – 7.06 (m, 1H), 5.62 (ddd, *J* = 7.8, 5.8, 3.8 Hz, 1H), 3.34 (td, *J* = 14.8, 7.8 Hz, 1H), 2.69 (dddd, *J* = 25.7, 15.2, 5.9, 1.3 Hz, 1H).
**$^{13}$C NMR** (101 MHz, Chloroform-*d*) δ (ppm) = 163.2 (d, *J* = 22.1 Hz), 158.1, 147.7, 138.0, 136.4, 130.3, 129.1, 127.7, 127.5, 127.2, 126.7, 122.6, 121.9 (qd, *J* = 283.6, 31.7 Hz), 117.6, 117.6, 92.3 (dq, *J* = 199.9, 34.3 Hz), 61.2, 34.2 (d, *J* = 20.3 Hz).
**$^{19}$F NMR** (376 MHz, Chloroform-*d*) δ (ppm) = -79.77 (d, *J* = 10.8 Hz, 3F), -166.55 (q, *J* = 10.7 Hz, 1F).
**HRMS (ESI) m/z:** $[M + H]^+$ Calcd for $C_{20}H_{15}F_4N_2O$ 375.1115; Found 375.1111.

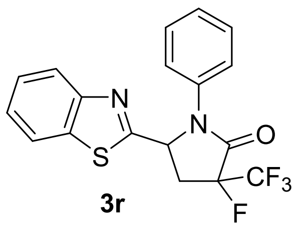

Chemical Formula: $C_{18}H_{12}F_4N_2OS$

**5-(benzo[d]thiazol-2-yl)-3-fluoro-1-phenyl-3-(trifluoromethyl)pyrrolidin-2-one (3r)**: white solid (mp = 140.3-141.2 °C), 21.3 mg, (Yield: 56%).

**$^1$H NMR** (400 MHz, Chloroform-*d*) δ (ppm) = 7.99 (dd, *J* = 8.1, 0.9 Hz, 1H), 7.84 – 7.78 (m, 1H), 7.57 – 7.45 (m, 3H), 7.43 – 7.30 (m, 3H), 7.24 – 7.17 (m, 1H), 5.81 (ddd, *J* = 8.0, 5.4, 3.6 Hz, 1H), 3.38 (td, *J* = 15.2, 7.9 Hz, 1H), 2.85 (dddd, *J* = 24.9, 15.3, 5.4, 1.1 Hz, 1H).

**$^{13}$C NMR** (101 MHz, Chloroform-*d*) δ (ppm) = 169.0, 162.6 (d, *J* = 22.1 Hz), 152.4, 135.8, 135.0, 129.3, 127.3, 126.6, 126.0, 123.5, 123.0, 122.0, 121.7 (qd, *J* = 283.5, 31.6 Hz), 91.7 (dq, *J* = 201.9, 34.4 Hz), 57.8, 34.2 (d, *J* = 21.1 Hz).

**$^{19}$F NMR** (376 MHz, Chloroform-*d*) δ (ppm) = -79.80 (d, *J* = 10.6 Hz, 3F), -166.09 (q, *J* = 10.8 Hz, 1F).

**HRMS (ESI) m/z:** $[M + H]^+$ Calcd for $C_{18}H_{13}F_4N_2OS$ 381.0679; Found 381.0678.

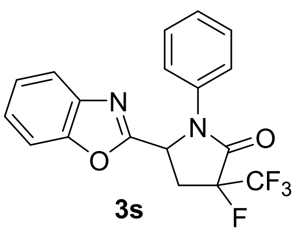

Chemical Formula:
$C_{18}H_{12}F_4N_2O_2$

**5-(benzo[d]oxazol-2-yl)-3-fluoro-1-phenyl-3-(trifluoromethyl)pyrrolidin-2-one (3s)**: white solid (mp = 154.7-155.4 °C), 19.0 mg, (Yield: 52%).

**$^1$H NMR** (400 MHz, Chloroform-*d*) δ (ppm) = 7.71 – 7.65 (m, 1H), 7.51 – 7.44 (m, 3H), 7.38 – 7.30 (m, 4H), 7.24 – 7.17 (m, 1H), 5.59 (ddd, *J* = 7.9, 5.8, 3.5 Hz, 1H), 3.29 (ddd, *J* = 15.1, 13.3, 7.9 Hz, 1H), 3.02 – 2.86 (m, 1H).

**$^{13}$C NMR** (101 MHz, Chloroform-*d*) δ (ppm) = 162.4 (d, *J* = 22.2 Hz), 161.3, 150.9, 140.4, 135.7, 129.3, 127.6, 126.2, 125.0, 123.2, 121.7 (qd, *J* = 283.7, 31.8 Hz), 120.6, 111.1, 91.3 (dq, *J* = 202.2, 34.5 Hz), 53.7, 31.8 (d, *J* = 21.7 Hz).

**$^{19}$F NMR** (376 MHz, Chloroform-*d*) δ (ppm) = -79.92 (d, *J* = 10.7 Hz, 3F), -168.06 (q, *J* = 10.8 Hz, 1F).

**HRMS (ESI) m/z:** $[M + H]^+$ Calcd for $C_{18}H_{13}F_4N_2O_2$ 365.0908; Found 365.0907.

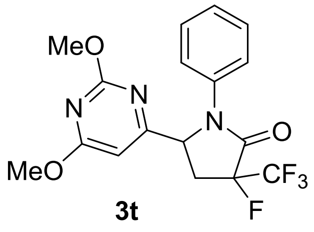

Chemical Formula: $C_{17}H_{15}F_4N_3O_3$

**5-(2,6-dimethoxypyrimidin-4-yl)-3-fluoro-1-phenyl-3-(trifluoromethyl)pyrrolidin-2-one (3t)**: dense yellow oil, 28.2 mg, (Yield: 73%).

**$^1$H NMR** (400 MHz, Chloroform-*d*) δ (ppm) = 7.50 – 7.43 (m, 2H), 7.34 (t, *J* = 8.0 Hz, 2H), 7.24 – 7.16 (m, 1H), 6.23 (s, 1H), 5.18 (dt, *J* = 8.3, 4.3 Hz, 1H), 3.97 (s, 3H), 3.91 (s, 3H), 3.14 (ddd, *J* = 17.7, 15.2, 8.1 Hz, 1H), 2.71 – 2.54 (m, 1H).

**$^{13}$C NMR** (101 MHz, Chloroform-*d*) δ (ppm) = 172.4, 167.9, 165.9, 163.0 (d, *J* = 22.1 Hz), 136.3, 129.2, 126.9, 122.5, 121.7 (qd, *J* = 283.3, 31.7 Hz), 99.5, 92.0 (dq, *J* = 200.4, 34.3 Hz), 59.3, 55.1, 54.1, 33.0 (d, *J* = 21.0 Hz).

**$^{19}$F NMR** (376 MHz, Chloroform-*d*) δ (ppm) = -79.94 (d, *J* = 10.3 Hz, 3F), -166.74 (q, *J* = 10.5 Hz, 1F).

**HRMS (ESI) m/z:** $[M + H]^+$ Calcd for $C_{17}H_{16}F_4N_3O_3$ 386.1122; Found 386.1121.

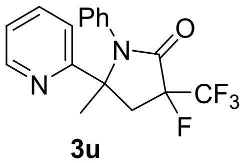


**3u**

Chemical Formula: $C_{17}H_{14}F_4N_2O$

**3-fluoro-5-methyl-1-phenyl-5-(pyridin-2-yl)-3-(trifluoromethyl)pyrrolidin-2-one (3u)**: yellow solid (mp = 138.4-139.7 ℃), 24.4 mg, (Yield: 72%).

**$^1$H NMR** (400 MHz, Chloroform-*d*) δ (ppm) = 8.69 (ddd, *J* = 4.8, 1.9, 1.0 Hz, 1H), 7.68 (td, *J* = 7.7, 1.8 Hz, 1H), 7.34 (dd, *J* = 8.0, 0.8 Hz, 1H), 7.30 – 7.22 (m, 4H), 6.92 – 6.83 (m, 2H), 3.08 (ddd, *J* = 27.3, 15.8, 1.0 Hz, 1H), 2.73 (dd, *J* = 23.0, 15.7 Hz, 1H), 1.81 (s, 3H).

**$^{13}$C NMR** (101 MHz, Chloroform-*d*) δ (ppm) = 163.9 (d, *J* = 22.0 Hz), 161.3, 149.7, 136.8, 134.9, 129.0, 128.1, 127.2, 123.0, 122.0 (qd, *J* = 284.0, 31.7 Hz), 120.4, 92.0 (dq, *J* = 198.4, 33.8 Hz), 65.5, 42.0 (d, *J* = 20.8 Hz), 24.7 (d, *J* = 2.7 Hz).

**$^{19}$F NMR** (376 MHz, Chloroform-*d*) δ (ppm) = -79.91 (d, *J* = 10.1 Hz, 3F), -166.69 (q, *J* = 9.7 Hz, 1F).

**HRMS (ESI) m/z:** $[M + H]^+$ Calcd for $C_{17}H_{15}F_4N_2O$ 339.1115; Found 339.1114.

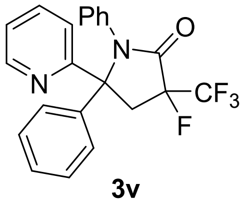


**3v**

Chemical Formula: $C_{22}H_{16}F_4N_2O$

**3-fluoro-1,5-diphenyl-5-(pyridin-2-yl)-3-(trifluoromethyl)pyrrolidin-2-one (3v)**: dense yellow oil, 30.1 mg, (Yield: 75%).

**$^1$H NMR** (400 MHz, Chloroform-*d*) δ (ppm) = 8.75 (ddd, *J* = 4.9, 1.8, 0.9 Hz, 1H), 7.59 (td, *J* = 7.8, 1.8 Hz, 1H), 7.32 – 7.24 (m, 6H), 7.23 – 7.18 (m, 1H), 7.14 – 7.03 (m, 5H), 3.91 (dd, *J* = 26.3, 15.5 Hz, 1H), 3.17 (dd, *J* = 25.7, 15.5 Hz, 1H).

**$^{13}$C NMR** (101 MHz, Chloroform-*d*) δ (ppm) = 164.4 (d, *J* = 21.9 Hz), 159.5, 149.2, 140.2, 136.3, 136.0, 128.5, 128.4, 128.3, 128.2, 127.1, 126.6, 123.9, 123.0, 121.8 (qd, *J* = 284.8, 31.6 Hz), 92.0 (dq, *J* = 198.1, 34.3 Hz), 73.6, 44.5 (d, *J* = 20.8 Hz).

**$^{19}$F NMR** (376 MHz, Chloroform-*d*) δ (ppm) = -79.24 (d, *J* = 10.1 Hz, 3F), -167.89 (q, *J* = 10.1 Hz, 1F).

**HRMS (ESI) m/z:** $[M + H]^+$ Calcd for $C_{22}H_{17}F_4N_2O$ 401.1272; Found 401.1269.

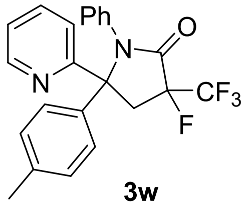


**3w**

Chemical Formula: $C_{23}H_{18}F_4N_2O$

**3-fluoro-1-phenyl-5-(pyridin-2-yl)-5-(p-tolyl)-3-(trifluoromethyl)pyrrolidin-2-one (3w)**: white solid (mp = 102.0-103.1 ℃), 30.3 mg, (Yield: 73%).

**$^1$H NMR** (400 MHz, Chloroform-*d*) δ (ppm) = 8.76 – 8.70 (m, 1H), 7.58 (td, *J* = 7.8, 1.9 Hz, 1H), 7.27 – 7.23 (m, 1H), 7.20 – 7.03 (m, 10H), 3.87 (dd, *J* = 26.4, 15.5 Hz, 1H), 3.15 (dd, *J* = 25.7, 15.5 Hz, 1H), 2.30 (s, 3H).

**$^{13}$C NMR** (101 MHz, Chloroform-*d*) δ (ppm) = 164.4 (d, *J* = 22.3 Hz), 159.7, 149.1, 138.2, 137.2, 136.2, 136.0, 129.1, 128.4, 128.1, 127.1, 126.7, 123.9, 122.9, 121.8 (qd, *J* = 282.8, 31.6 Hz), 92.0 (dq, *J* = 197.2, 34.2 Hz), 73.5, 44.4 (d, *J* = 20.8 Hz), 20.9.

**$^{19}$F NMR** (376 MHz, Chloroform-*d*) δ (ppm) = -79.24 (d, *J* = 10.0 Hz, 3F), -167.86 (q, *J* = 9.9 Hz, 1F).

**HRMS (ESI) m/z:** $[M + H]^+$ Calcd for $C_{23}H_{19}F_4N_2O$ 425.1428; Found 425.1427.

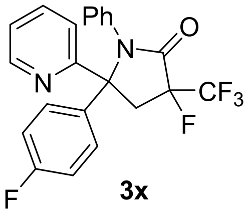


**3x**

Chemical Formula: $C_{22}H_{15}F_5N_2O$

**3-fluoro-5-(4-fluorophenyl)-1-phenyl-5-(pyridin-2-yl)-3-(trifluoromethyl)pyrrolidin-2-one (3x)**: dense yellow oil, 29.8 mg, (Yield: 71%).

**$^1$H NMR** (400 MHz, Chloroform-*d*) δ (ppm) = 8.75 (ddd, *J* = 4.8, 1.9, 0.9 Hz, 1H), 7.64 (td, *J* = 7.8, 1.9 Hz, 1H), 7.33 – 7.25 (m, 4H), 7.15 – 7.09 (m, 3H), 7.07 – 7.02 (m, 2H), 7.00 – 6.94 (m, 2H), 3.82 (dd, *J* = 25.3, 15.5 Hz, 1H), 3.14 (dd, *J* = 26.6, 15.5 Hz, 1H).

**$^{13}$C NMR** (101 MHz, Chloroform-*d*) δ (ppm) = 164.2 (d, *J* = 21.9 Hz), 162.2 (d, *J* = 249.1 Hz), 159.5, 149.3, 136.6, 135.7, 130.2 (d, *J* = 8.2 Hz), 128.5, 127.1, 126.3, 123.6, 123.1, 121.8 (qd, *J* = 282.6, 31.5 Hz), 115.4 (d, *J* = 21.7 Hz), 91.9 (dq, *J* = 197.8, 34.2 Hz), 73.0, 44.9 (d, *J* = 20.6 Hz).

**$^{19}$F NMR** (376 MHz, Chloroform-*d*) δ (ppm) = -79.28 (d, *J* = 9.7 Hz, 3F), -113.08 (s, 1F), -168.53 (q, *J* = 9.9 Hz, 1F).

**HRMS (ESI) m/z:** $[M + H]^+$ Calcd for $C_{22}H_{16}F_5N_2O$ 419.1177; Found 419.1175.

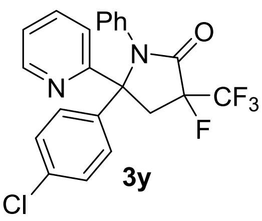

**3y**

Chemical Formula: $C_{22}H_{15}ClF_4N_2O$

**5-(4-chlorophenyl)-3-fluoro-1-phenyl-5-(pyridin-2-yl)-3-(trifluoromethyl)pyrrolidin-2-one (3y)**: dense colorless oil, 30.9 mg, (Yield: 71%).

**$^1$H NMR** (400 MHz, Chloroform-*d*) δ (ppm) = 8.75 (ddd, *J* = 4.8, 1.8, 0.9 Hz, 1H), 7.62 (td, *J* = 7.8, 1.8 Hz, 1H), 7.31 – 7.25 (m, 5H), 7.21 (d, *J* = 8.0 Hz, 1H), 7.16 – 7.10 (m, 3H), 7.07 – 7.01 (m, 2H), 3.88 (dd, *J* = 25.8, 15.5 Hz, 1H), 3.09 (dd, *J* = 25.6, 15.5 Hz, 1H).

**$^{13}$C NMR** (101 MHz, Chloroform-*d*) δ (ppm) = 164.2 (d, *J* = 21.9 Hz), 159.1, 149.3, 138.7, 136.6, 135.7, 134.3, 129.6, 128.6, 128.6, 127.2, 126.3, 123.7, 123.2, 121.7 (qd, *J* = 282.6, 31.4 Hz), 91.8 (dq, *J* = 197.8, 34.3 Hz), 73.0, 44.7 (d, *J* = 20.8 Hz).

**$^{19}$F NMR** (376 MHz, Chloroform-*d*) δ (ppm) = -79.25 (d, *J* = 10.1 Hz, 3F), -168.13 (q, *J* = 10.1 Hz, 1F).

**HRMS (ESI) m/z:** $[M + H]^+$ Calcd for $C_{22}H_{16}ClF_4N_2O$ 435.0882; Found 435.0878.

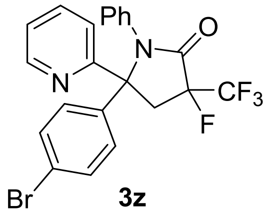

**3z**

Chemical Formula: $C_{22}H_{15}BrF_4N_2O$

**5-(4-bromophenyl)-3-fluoro-1-phenyl-5-(pyridin-2-yl)-3-(trifluoromethyl)pyrrolidin-2-one (3z)**: white solid (mp = 107.6-108.5 °C), 31.8 mg, (Yield: 66%).

**$^1$H NMR** (400 MHz, Chloroform-*d*) δ (ppm) = 8.75 (ddd, *J* = 4.8, 1.8, 0.9 Hz, 1H), 7.61 (td, *J* = 7.8, 1.9 Hz, 1H), 7.46 – 7.40 (m, 2H), 7.29 (ddd, *J* = 7.6, 4.8, 1.0 Hz, 1H), 7.23 – 7.17 (m, 3H), 7.16 – 7.10 (m, 3H), 7.07 – 7.00 (m, 2H), 3.89 (dd, *J* = 26.0, 15.5 Hz, 1H), 3.08 (dd, *J* = 25.3, 15.5 Hz, 1H).

**$^{13}$C NMR** (101 MHz, Chloroform-*d*) δ (ppm) = 164.3 (d, *J* = 22.1 Hz), 159.0, 149.3, 139.4, 136.5, 135.7, 131.6, 129.9, 128.6, 127.2, 126.3, 123.8, 123.2, 122.5, 121.7 (qd, *J* = 282.7, 31.4 Hz), 91.8 (dq, *J* = 197.8, 34.3 Hz), 73.1, 44.6 (d, *J* = 20.9 Hz).

**$^{19}$F NMR** (376 MHz, Chloroform-*d*) δ (ppm) = -79.25 (d, *J* = 9.8 Hz, 3F), -168.01 (q, *J* = 10.1 Hz, 1F).

**HRMS (ESI) m/z:** $[M + H]^+$ Calcd for $C_{22}H_{16}BrF_4N_2O$ 479.0377; Found 479.0375.

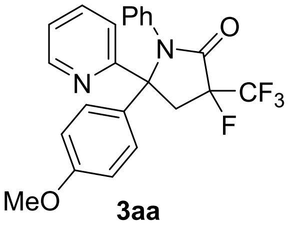

**3aa**

Chemical Formula: $C_{23}H_{18}F_4N_2O_2$

**3-fluoro-5-(4-methoxyphenyl)-1-phenyl-5-(pyridin-2-yl)-3-(trifluoromethyl)pyrrolidin-2-one (3aa)**: dense brown oil, 33.3 mg, (Yield: 77%).

**$^1$H NMR** (400 MHz, Chloroform-*d*) δ (ppm) = 8.73 (ddd, *J* = 4.8, 1.9, 0.9 Hz, 1H), 7.61 (td, *J* = 7.8, 1.8 Hz, 1H), 7.29 – 7.22 (m, 2H), 7.19 – 7.04 (m, 7H), 6.83 – 6.77 (m, 2H), 3.84 – 3.73 (m, 4H), 3.17 (dd, *J* = 26.9, 15.5 Hz, 1H).

**$^{13}$C NMR** (101 MHz, Chloroform-*d*) δ (ppm) = 164.2 (d, *J* = 21.7 Hz), 160.0, 159.3, 149.1, 136.4, 136.0, 131.7, 131.7, 129.6, 128.4, 127.0, 126.6, 123.6, 122.9, 121.8 (qd, *J* = 284.0, 31.2 Hz), 113.7, 92.0 (dq, *J* = 197.4, 34.1 Hz), 55.2, 44.5 (d, *J* = 20.6 Hz).

**$^{19}$F NMR** (376 MHz, Chloroform-*d*) δ (ppm) = -79.25 (d, *J* = 9.9 Hz, 3F), -168.47 (q, *J* = 10.0 Hz, 1F).

**HRMS (ESI) m/z:** $[M + H]^+$ Calcd for $C_{23}H_{19}F_4N_2O_2$ 434.1377; Found 434.1374.

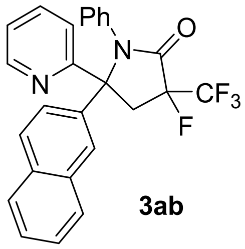


**3ab**

Chemical Formula: $C_{26}H_{18}F_4N_2O$

**3-fluoro-5-(naphthalen-2-yl)-1-phenyl-5-(pyridin-2-yl)-3-(trifluoromethyl)pyrrolidin-2-one (3ab)**: brown solid (mp = 248.5-249.3 °C), 15.6 mg, (Yield: 34%).

**$^1$H NMR** (400 MHz, Chloroform-*d*) δ (ppm) = 8.77 – 8.68 (m, 1H), 8.06 (d, *J* = 7.3 Hz, 1H), 7.96 – 7.87 (m, 2H), 7.57 (t, *J* = 7.8 Hz, 1H), 7.46 – 7.39 (m, 1H), 7.25 – 7.19 (m, 1H), 7.17 – 7.00 (m, 8H), 6.49 (d, *J* = 7.5 Hz, 1H), 4.85 (dd, *J* = 34.4, 15.1 Hz, 1H), 3.32 (dd, *J* = 15.0, 9.5 Hz, 1H).

**$^{13}$C NMR** (101 MHz, Chloroform-*d*) δ (ppm) = 165.6 (d, *J* = 22.7 Hz), 157.8, 149.3, 137.5, 136.5, 135.5, 134.9, 129.9, 129.6, 129.6, 128.5, 127.8, 127.6, 126.5, 126.0, 125.9, 125.6, 124.4, 123.1, 121.5 (qd, *J* = 284.3, 32.8 Hz), 92.1 (dq, *J* = 194.9, 33.5 Hz), 74.4 (d, *J* = 3.0 Hz), 41.4 (d, *J* = 22.0 Hz).

**$^{19}$F NMR** (376 MHz, Chloroform-*d*) δ (ppm) = -78.82 (d, *J* = 11.5 Hz, 3F), -161.79 (s, 1F).

**HRMS (ESI) m/z:** $[M + H]^+$ Calcd for $C_{26}H_{19}F_4N_2O$ 451.1428; Found 451.1425.

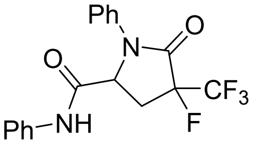


**3ac**

Chemical Formula: $C_{18}H_{14}F_4N_2O_2$

**4-fluoro-5-oxo-*N*,1-diphenyl-4-(trifluoromethyl)pyrrolidine-2-carboxamide (3ac)**: dense yellow oil, 18.7 mg, (Yield: 51%).

**$^1$H NMR** (400 MHz, Acetone-$d_6$) δ (ppm) = 9.76 (s, 1H), 7.67 – 7.60 (m, 2H), 7.61 – 7.54 (m, 2H), 7.48 – 7.38 (m, 2H), 7.32 – 7.24 (m, 3H), 7.14 – 7.05 (m, 1H), 5.20 (dt, *J* = 8.2, 4.1 Hz, 1H), 3.33 (ddd, *J* = 19.8, 15.4, 8.2 Hz, 1H), 2.88 – 2.74 (m, 1H).

**$^{13}$C NMR** (101 MHz, Acetone-$d_6$) δ (ppm) = 167.8, 163.5 (d, *J* = 22.4 Hz), 139.1, 138.0, 129.9, 129.6, 127.7, 125.1, 124.6, 124.3, 123.5, 121.8, 121.5, 120.4, 94.2, 93.8, 92.2, 91.8, 59.6, 31.2 (d, *J* = 21.5 Hz).

**$^{19}$F NMR** (376 MHz, Acetone-$d_6$) δ (ppm) = -80.67 (d, $J$ = 10.1 Hz, 3F), -167.55 (q, $J$ = 10.1 Hz, 1F).

**HRMS (ESI) m/z:** $[M + H]^+$ Calcd for $C_{18}H_{15}F_4N_2O_2$ 367.1064; Found 367.1061.

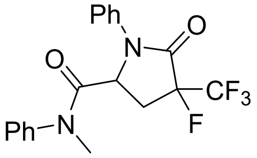


**3ad**

Chemical Formula: $C_{19}H_{16}F_4N_2O_2$

**4-fluoro-*N*-methyl-5-oxo-*N*,1-diphenyl-4-(trifluoromethyl)pyrrolidine-2-carboxamide (3ad)**: dense yellow oil, 22.4 mg, (Yield: 59%).

**$^1$H NMR** (400 MHz, Chloroform-$d$) δ (ppm) = 7.55 – 7.47 (m, 3H), 7.46 – 7.39 (m, 2H), 7.35 – 7.27 (m, 3H), 7.06 (dd, $J$ = 8.0, 1.6 Hz, 2H), 4.62 (ddd, $J$ = 7.9, 5.3, 4.1 Hz, 1H), 3.21 (s, 3H), 2.76 – 2.54 (m, 2H).

**$^{13}$C NMR** (101 MHz, Chloroform-$d$) δ (ppm) = 168.0, 162.7 (d, $J$ = 22.4 Hz), 141.7, 136.2, 130.5, 129.2, 129.2, 127.6, 126.9, 123.6, 123.3, 122.9, 120.4, 120.1, 92.5, 92.2, 90.5, 90.2, 54.8, 38.0, 30.6 (d, $J$ = 21.8 Hz).

**$^{19}$F NMR** (376 MHz, Chloroform-$d$) δ (ppm) = -80.25 (d, $J$ = 10.8 Hz, 3F), -167.30 (q, $J$ = 10.8 Hz, 1F).

**HRMS (ESI) m/z:** $[M + H]^+$ Calcd for $C_{19}H_{17}F_4N_2O_2$ 381.1221; Found 381.1220.

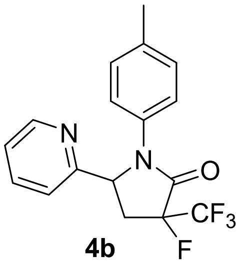


**4b**

Chemical Formula: $C_{17}H_{14}F_4N_2O$

**3-fluoro-5-(pyridin-2-yl)-1-(p-tolyl)-3-(trifluoromethyl)pyrrolidin-2-one (4b)**: yellow solid (mp = 85.6-86.8 ℃), 26.2 mg, (Yield: 77%).

**$^1$H NMR** (400 MHz, Chloroform-$d$) δ (ppm) = 8.62 – 8.54 (m, 1H), 7.60 (td, $J$ = 7.7, 1.8 Hz, 1H), 7.29 (d, $J$ = 8.5 Hz, 2H), 7.22 – 7.12 (m, 2H), 7.07 (d, $J$ = 8.2 Hz, 2H), 5.35 (ddd, $J$ = 7.8, 5.6, 4.0 Hz, 1H), 3.20 (td, $J$ = 14.9, 7.7 Hz, 1H), 2.68 (dddd, $J$ = 25.8, 15.1, 5.7, 1.3 Hz, 1H), 2.25 (s, 3H).

**$^{13}$C NMR** (101 MHz, Chloroform-$d$) δ (ppm) = 163.1 (d, $J$ = 22.0 Hz), 157.7, 150.0, 137.2, 136.7, 133.8, 129.6, 123.4, 122.8, 121.9 (qd, $J$ = 284.5, 32.1 Hz), 121.4, 92.3 (dq, $J$ = 199.6, 34.2 Hz), 60.7, 34.0 (d, $J$ = 20.5 Hz), 20.9.

**$^{19}$F NMR** (376 MHz, Chloroform-$d$) δ (ppm) = -79.90 (d, $J$ = 10.8 Hz, 3F), -167.06 (q, $J$ = 10.8 Hz, 1F).

**HRMS (ESI) m/z:** $[M + H]^+$ Calcd for $C_{17}H_{15}F_4N_2O$ 339.1115; Found 339.1112.

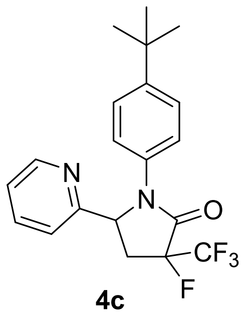

**4c**

Chemical Formula: $C_{20}H_{20}F_4N_2O$

**1-(4-(tert-butyl)phenyl)-3-fluoro-5-(pyridin-2-yl)-3-(trifluoromethyl)pyrrolidin-2-one (4c)**: white solid (mp = 121.9-123.4 ℃), 28.3 mg, (Yield: 74%).

**$^{1}$H NMR** (400 MHz, Chloroform-*d*) δ (ppm) = 8.59 (ddd, *J* = 4.8, 1.7, 0.9 Hz, 1H), 7.62 (td, *J* = 7.7, 1.8 Hz, 1H), 7.39 – 7.33 (m, 2H), 7.33 – 7.27 (m, 2H), 7.23 – 7.16 (m, 2H), 5.39 (ddd, *J* = 7.9, 5.2, 3.8 Hz, 1H), 3.20 (ddd, *J* = 16.0, 15.1, 7.8 Hz, 1H), 2.66 (dddd, *J* = 25.4, 15.1, 5.2, 1.2 Hz, 1H), 1.24 (s, 9H).

**$^{13}$C NMR** (101 MHz, Chloroform-*d*) δ (ppm) = 163.1 (d, *J* = 22.1 Hz), 157.9, 150.0, 149.7, 137.3, 133.7, 125.9, 123.4, 122.2, 121.9 (qd, *J* = 284.8, 31.9 Hz), 121.2, 92.4 (dq, *J* = 199.5, 34.1 Hz), 60.6, 34.4, 34.1 (d, *J* = 20.5 Hz), 31.1.

**$^{19}$F NMR** (376 MHz, Chloroform-*d*) δ (ppm) = -79.89 (d, *J* = 10.6 Hz, 3F), -166.98 (q, *J* = 10.9 Hz, 1F).

**HRMS (ESI) m/z:** $[M + H]^+$ Calcd for $C_{20}H_{21}F_4N_2O$ 351.1585; Found 351.1581.

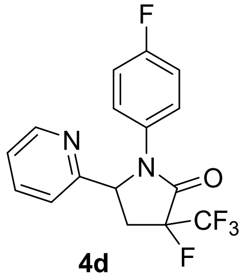

**4d**

Chemical Formula: $C_{16}H_{11}F_5N_2O$

**3-fluoro-1-(4-fluorophenyl)-5-(pyridin-2-yl)-3-(trifluoromethyl)pyrrolidin-2-one (4d)**: white solid (mp = 88.4-89.1 ℃), 25.8 mg, (Yield: 75%).

**$^{1}$H NMR** (400 MHz, Chloroform-*d*) δ (ppm) = 8.62 – 8.54 (m, 1H), 7.62 (td, *J* = 7.7, 1.8 Hz, 1H), 7.40 – 7.33 (m, 2H), 7.22 (ddd, *J* = 7.6, 4.8, 1.1 Hz, 1H), 7.16 (d, *J* = 7.8 Hz, 1H), 7.00 – 6.93 (m, 2H), 5.32 (ddd, *J* = 7.6, 6.0, 3.9 Hz, 1H), 3.22 (ddd, *J* = 15.0, 12.9, 7.6 Hz, 1H), 2.74 (dddd, *J* = 25.9, 15.1, 5.9, 1.2 Hz, 1H).

**$^{13}$C NMR** (101 MHz, Chloroform-*d*) δ (ppm) = 163.2 (d, *J* = 22.4 Hz), 160.7 (d, *J* = 247.5 Hz), 156.9, 150.2, 137.3, 132.2 (d, *J* = 3.1 Hz), 125.1 (d, *J* = 8.4 Hz), 123.7, 121.9 (qd, *J* = 283.6, 31.9 Hz), 121.7, 115.9 (d, *J* = 22.8 Hz), 92.2 (dq, *J* = 200.3, 34.2 Hz), 60.8, 34.0 (d, *J* = 20.5 Hz).

**$^{19}$F NMR** (376 MHz, Chloroform-*d*) δ (ppm) = -79.92 (d, *J* = 10.7 Hz, 3F), -114.00 (s, 1F), -167.48 (q, *J* = 10.7 Hz, 1F).

**HRMS (ESI) m/z:** $[M + H]^+$ Calcd for $C_{16}H_{12}F_5N_2O$ 343.0864; Found 343.0863.

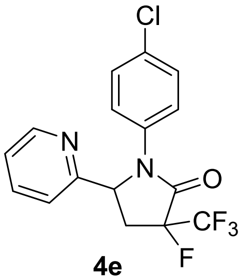

**4e**

Chemical Formula: $C_{16}H_{11}ClF_4N_2O$

**1-(4-chlorophenyl)-3-fluoro-5-(pyridin-2-yl)-3-(trifluoromethyl)pyrrolidin-2-one (4e)**: white solid (mp = 112.4-113.2 ℃), 24.9 mg, (Yield: 69%).

**$^{1}$H NMR** (400 MHz, Chloroform-*d*) δ (ppm) = 8.61 – 8.55 (m, 1H), 7.63 (td, *J* = 7.7, 1.8 Hz, 1H), 7.41 – 7.35 (m, 2H), 7.27 – 7.19 (m, 3H), 7.15 (d, *J* = 7.8 Hz, 1H), 5.35 (ddd, *J* = 7.7, 5.7, 3.8 Hz, 1H), 3.22 (td, *J* = 14.6, 7.7 Hz, 1H), 2.70 (dddd, *J* = 25.7, 15.0, 5.7, 1.2 Hz, 1H).

**$^{13}$C NMR** (101 MHz, Chloroform-*d*) δ (ppm) = 163.1 (d, *J* = 22.4 Hz), 157.1, 150.2, 137.3, 134.9, 132.2, 129.2, 124.1, 123.7, 121.8 (qd, *J* = 283.5, 31.8 Hz), 121.4, 92.2 (dq, *J* = 200.3, 34.3 Hz), 60.6, 34.0 (d, *J* = 20.5 Hz).

**$^{19}$F NMR** (376 MHz, Chloroform-*d*) δ (ppm) = -79.88 (d, *J* = 10.4 Hz, 3F), -167.19 (q, *J* = 10.7 Hz, 1F).

**HRMS (ESI) m/z:** $[M + H]^+$ Calcd for $C_{16}H_{12}ClF_4N_2O$ 359.0569; Found 359.0568.

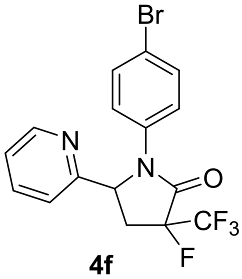

**4f**

Chemical Formula: $C_{16}H_{11}BrF_4N_2O$

**1-(4-bromophenyl)-3-fluoro-5-(pyridin-2-yl)-3-(trifluoromethyl)pyrrolidin-2-one (4f)**: white solid (mp = 115.6-116.8 ℃), 22.7 mg, (Yield: 56%).

**$^{1}$H NMR** (400 MHz, Chloroform-*d*) δ (ppm) = 8.58 (ddd, *J* = 4.8, 1.8, 0.9 Hz, 1H), 7.63 (td, *J* = 7.7, 1.7 Hz, 1H), 7.43 – 7.37 (m, 2H), 7.36 – 7.30 (m, 2H), 7.22 (ddd, *J* = 7.6, 4.8, 1.1 Hz, 1H), 7.16 (d, *J* = 7.8 Hz, 1H), 5.35 (ddd, *J* = 7.8, 5.7, 3.8 Hz, 1H), 3.22 (ddd, *J* = 15.1, 14.2, 7.7 Hz, 1H), 2.69 (dddd, *J* = 25.6, 15.1, 5.7, 1.3 Hz, 1H).

**$^{13}$C NMR** (101 MHz, Chloroform-*d*) δ (ppm) = 163.1 (d, *J* = 22.4 Hz), 157.0, 150.2, 137.4, 135.4, 132.1, 124.3, 123.7, 121.8 (qd, *J* = 283.6, 31.8 Hz), 121.4, 120.0, 92.2 (dq, *J* = 200.4, 34.3 Hz), 60.5, 34.0 (d, *J* = 20.5 Hz).

**$^{19}$F NMR** (376 MHz, Chloroform-*d*) δ (ppm) = -79.86 (d, *J* = 10.8 Hz, 3F), -167.17 (q, *J* = 10.8 Hz, 1F).

**HRMS (ESI) m/z:** $[M + H]^+$ Calcd for $C_{16}H_{12}BrF_4N_2O$ 403.0064; Found 403.0062.

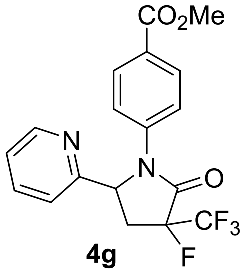

Chemical Formula: $C_{18}H_{14}F_4N_2O_3$

**methyl 4-(3-fluoro-2-oxo-5-(pyridin-2-yl)-3-(trifluoromethyl)pyrrolidin-1-yl)benzoate (4g)**: white solid (mp = 149.9-150.6 °C), 25.1 mg, (Yield: 65%).

**$^1$H NMR** (400 MHz, Chloroform-*d*) δ (ppm) = 8.60 – 8.54 (m, 1H), 8.00 – 7.92 (m, 2H), 7.67 – 7.55 (m, 3H), 7.21 (ddd, *J* = 7.6, 4.8, 1.1 Hz, 1H), 7.17 (d, *J* = 7.9 Hz, 1H), 5.46 (ddd, *J* = 7.8, 5.4, 3.5 Hz, 1H), 3.87 (s, 3H), 3.24 (td, *J* = 15.3, 7.8 Hz, 1H), 2.76 – 2.61 (m, 1H).

**$^{13}$C NMR** (101 MHz, Chloroform-*d*) δ (ppm) = 166.1, 163.3 (d, *J* = 22.6 Hz), 157.2, 150.2, 140.3, 137.4, 130.5, 127.9, 123.6, 121.8, 121.7 (qd, *J* = 284.4, 31.6 Hz), 121.1, 92.3 (dq, *J* = 201.0, 34.3 Hz), 60.4, 52.2, 34.1 (d, *J* = 20.4 Hz).

**$^{19}$F NMR** (376 MHz, Chloroform-*d*) δ (ppm) = -79.83 (d, *J* = 10.5 Hz, 3F), -166.95 (q, *J* = 10.9 Hz, 1F).

**HRMS (ESI) m/z:** $[M + H]^+$ Calcd for $C_{18}H_{15}F_4N_2O_3$ 383.1013; Found 383.1011.

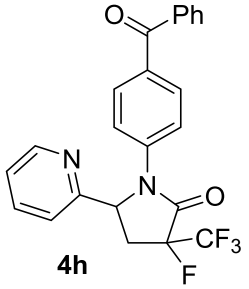

Chemical Formula: $C_{23}H_{16}F_4N_2O_2$

**1-(4-benzoylphenyl)-3-fluoro-5-(pyridin-2-yl)-3-(trifluoromethyl)pyrrolidin-2-one (4h)**: brown solid (mp = 129.5-130.3 °C), 24.6 mg, (Yield: 57%).

**$^1$H NMR** (400 MHz, Chloroform-*d*) δ (ppm) = 8.59 (ddd, *J* = 4.8, 1.8, 0.9 Hz, 1H), 7.78 – 7.70 (m, 4H), 7.65 – 7.54 (m, 4H), 7.49 – 7.43 (m, 2H), 7.25 – 7.17 (m, 2H), 5.48 (ddd, *J* = 7.8, 5.6, 3.5 Hz, 1H), 3.26 (td, *J* = 15.1, 7.8 Hz, 1H), 2.70 (dddd, *J* = 25.5, 15.3, 5.6, 1.3 Hz, 1H).

**$^{13}$C NMR** (101 MHz, Chloroform-*d*) δ (ppm) = 195.4, 163.4 (d, *J* = 22.1 Hz), 157.2, 150.3, 139.9, 137.5, 137.2, 135.2, 132.6, 131.0, 129.9, 128.3, 123.7, 121.8, 121.1, 118.9 (qd, *J* = 283.8, 32.1 Hz), 92.3 (dq, *J* = 201.3, 34.4 Hz), 60.4, 34.1 (d, *J* = 20.4 Hz).

**$^{19}$F NMR** (376 MHz, Chloroform-*d*) δ (ppm) = -79.80 (d, *J* = 10.5 Hz, 3F), -167.01 (q, *J* = 10.7 Hz, 1F).

**HRMS (ESI) m/z:** $[M + H]^+$ Calcd for $C_{23}H_{17}F_4N_2O_2$ 429.1221; Found 429.1214.

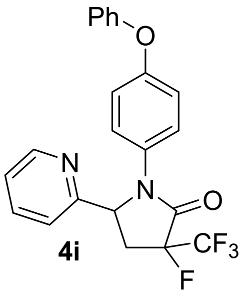


**4i**

Chemical Formula: $C_{22}H_{16}F_4N_2O_2$

**3-fluoro-1-(4-phenoxyphenyl)-5-(pyridin-2-yl)-3-(trifluoromethyl)pyrrolidin-2-one (4i)**: yellow solid (mp = 109.4-110.2 ℃), 32.3 mg, (Yield: 77%).

**$^1$H NMR** (400 MHz, Chloroform-*d*) δ (ppm) = 8.64 – 8.54 (m, 1H), 7.62 (td, *J* = 7.7, 1.8 Hz, 1H), 7.36 – 7.27 (m, 4H), 7.23 – 7.15 (m, 2H), 7.10 (t, *J* = 7.4 Hz, 1H), 6.93 (d, *J* = 8.0 Hz, 2H), 6.88 (d, *J* = 9.0 Hz, 2H), 5.32 (ddd, *J* = 7.7, 5.7, 4.0 Hz, 1H), 3.20 (td, *J* = 14.4, 7.6 Hz, 1H), 2.72 (ddd, *J* = 25.8, 15.0, 5.8 Hz, 1H).

**$^{13}$C NMR** (101 MHz, Chloroform-*d*) δ (ppm) = 163.1 (d, *J* = 22.3 Hz), 157.3, 156.4, 155.8, 150.1, 137.2, 131.2, 129.8, 124.7, 123.8, 123.6, 121.9 (qd, *J* = 284.6, 31.8 Hz), 121.6, 119.2, 118.7, 92.2 (dq, *J* = 200.1, 34.3 Hz), 60.9, 34.0 (d, *J* = 20.4 Hz).

**$^{19}$F NMR** (376 MHz, Chloroform-*d*) δ (ppm) = -79.88 (d, *J* = 10.8 Hz, 3F), -167.23 (q, *J* = 10.9 Hz, 1F).

**HRMS (ESI) m/z:** $[M + H]^+$ Calcd for $C_{22}H_{17}F_4N_2O_2$ 417.1221; Found 417.1216.

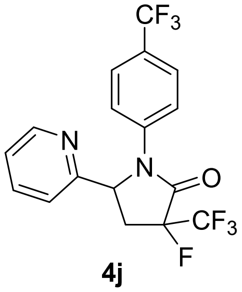


**4j**

Chemical Formula: $C_{17}H_{11}F_7N_2O$

**3-fluoro-5-(pyridin-2-yl)-3-(trifluoromethyl)-1-(4-(trifluoromethyl)phenyl)pyrrolidin-2-one (4j)**: white solid (mp = 100.5-101.6 ℃), 21.7 mg, (Yield: 55%).

**$^1$H NMR** (400 MHz, Chloroform-*d*) δ (ppm) = 8.59 (dt, *J* = 4.9, 1.4 Hz, 1H), 7.68 – 7.60 (m, 3H), 7.55 (d, *J* = 8.9 Hz, 2H), 7.23 (ddd, *J* = 7.6, 4.8, 1.1 Hz, 1H), 7.18 (d, *J* = 7.9 Hz, 1H), 5.44 (ddd, *J* = 7.9, 5.6, 3.6 Hz, 1H), 3.25 (td, *J* = 15.1, 7.8 Hz, 1H), 2.69 (dddd, *J* = 25.3, 15.1, 5.6, 1.3 Hz, 1H).

**$^{13}$C NMR** (101 MHz, Chloroform-*d*) δ (ppm) = 163.4 (d, *J* = 22.2 Hz), 157.0, 150.3, 139.4, 137.5, 128.3 (q, *J* = 33.1 Hz), 126.2 (q, *J* = 3.7 Hz), 123.6 (q, *J* = 272.0 Hz), 123.7, 122.3, 121.7 (qd, *J* = 283.4, 31.5 Hz), 121.1, 92.2 (dq, *J* = 200.5, 34.3 Hz), 60.4, 34.1 (d, *J* = 20.5 Hz).

**$^{19}$F NMR** (376 MHz, Chloroform-*d*) δ (ppm) = -62.62 (s, 3F), -79.85 (d, *J* = 10.2 Hz, 3F), -167.14 (q, *J* = 10.3 Hz, 1F).

**HRMS (ESI) m/z:** $[M + H]^+$ Calcd for $C_{17}H_{12}F_7N_2O$ 393.0832; Found 393.0827.

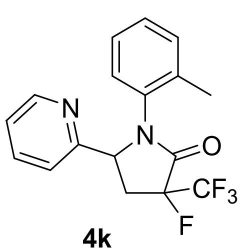

**4k**

Chemical Formula: $C_{17}H_{14}F_4N_2O$

**3-fluoro-5-(pyridin-2-yl)-1-(o-tolyl)-3-(trifluoromethyl)pyrrolidin-2-one (4k)**: yellow solid (mp = 105.2-106.0 ℃), 19.6 mg, (Yield: 57%).

**$^1$H NMR** (400 MHz, Chloroform-*d*) δ (ppm) = 8.65 – 8.56 (m, 1H), 7.52 (td, *J* = 7.6, 1.8 Hz, 1H), 7.19 (ddd, *J* = 7.7, 4.8, 1.1 Hz, 1H), 7.15 – 7.08 (m, 2H), 7.03 (d, *J* = 7.7 Hz, 2H), 6.85 (d, *J* = 8.0 Hz, 1H), 5.09 (td, *J* = 6.9, 4.4 Hz, 1H), 3.27 – 3.11 (m, 2H), 2.17 (s, 3H).

**$^{13}$C NMR** (101 MHz, Chloroform-*d*) δ (ppm) = 162.8 (d, *J* = 22.3 Hz), 155.3, 150.1, 136.7, 136.1, 134.3, 131.2, 128.6, 126.4, 123.9, 123.7, 122.3 (qd, *J* = 284.9, 32.2 Hz), 91.8 (dq, *J* = 200.4, 34.0 Hz), 61.4, 33.2 (d, *J* = 20.9 Hz), 17.8.

**$^{19}$F NMR** (376 MHz, Chloroform-*d*) δ (ppm) = -79.97 (d, *J* = 11.2 Hz, 3F), -168.19 (q, *J* = 11.0 Hz, 1F).

**HRMS (ESI) m/z:** $[M + H]^+$ Calcd for $C_{17}H_{15}F_4N_2O_2$ 339.1115; Found 339.1116.

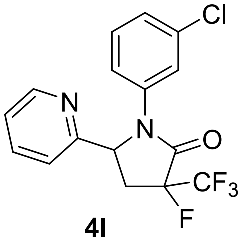

**4l**

Chemical Formula: $C_{16}H_{11}ClF_4N_2O$

**1-(3-chlorophenyl)-3-fluoro-5-(pyridin-2-yl)-3-(trifluoromethyl)pyrrolidin-2-one (4l)**: white solid (mp = 101.4-102.2 ℃), 26.8 mg, (Yield: 74%).

**$^1$H NMR** (400 MHz, Chloroform-*d*) δ (ppm) = 8.62 – 8.55 (m, 1H), 7.64 (td, *J* = 7.7, 1.8 Hz, 1H), 7.57 (t, *J* = 2.0 Hz, 1H), 7.29 – 7.11 (m, 5H), 5.37 (ddd, *J* = 7.8, 5.7, 3.7 Hz, 1H), 3.22 (td, *J* = 14.8, 7.7 Hz, 1H), 2.70 (dddd, *J* = 25.4, 15.0, 5.6, 1.3 Hz, 1H).

**$^{13}$C NMR** (101 MHz, Chloroform-*d*) δ (ppm) = 163.2 (d, *J* = 22.5 Hz), 157.0, 150.3, 137.42, 137.37, 134.7, 130.0, 126.8, 123.7, 123.0, 121.8 (qd, *J* = 284.5, 31.8 Hz), 121.3, 120.7, 92.2 (dq, *J* = 200.3, 34.3 Hz), 60.5, 34.0 (d, *J* = 20.4 Hz).

**$^{19}$F NMR** (376 MHz,Chloroform-*d*) δ (ppm) = -79.87 (d, *J* = 10.4 Hz, 3F), -167.24 (q, *J* = 10.8 Hz, 1F).

**HRMS (ESI) m/z:** $[M + H]^+$ Calcd for $C_{16}H_{12}ClF_4N_2O$ 359.0569; Found 359.0562.

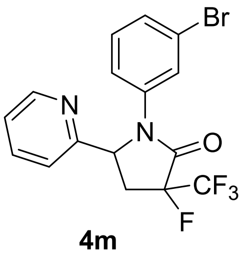


**4m**

Chemical Formula: $C_{16}H_{11}BrF_4N_2O$

**1-(3-bromophenyl)-3-fluoro-5-(pyridin-2-yl)-3-(trifluoromethyl)pyrrolidin-2-one (4m)**: yellow solid (mp = 80.5-81.6 °C), 23.7 mg, (Yield: 58%).

**$^1$H NMR** (400 MHz, Chloroform-*d*) δ (ppm) = 8.62 – 8.56 (m, 1H), 7.72 (t, *J* = 2.0 Hz, 1H), 7.64 (td, *J* = 7.8, 1.8 Hz, 1H), 7.33 – 7.27 (m, 2H), 7.22 (ddd, *J* = 7.7, 4.8, 1.1 Hz, 1H), 7.19 – 7.11 (m, 2H), 5.36 (ddd, *J* = 7.8, 5.6, 3.7 Hz, 1H), 3.21 (td, *J* = 14.8, 7.7 Hz, 1H), 2.70 (dddd, *J* = 25.6, 15.1, 5.6, 1.2 Hz, 1H).

**$^{13}$C NMR** (101 MHz, Chloroform-*d*) δ (ppm) = 163.2 (d, *J* = 22.4 Hz), 157.0, 150.3, 137.6, 137.4, 130.2, 129.8, 125.9, 123.7, 122.6, 121.8 (qd, *J* = 283.5, 31.7 Hz), 121.3, 121.2, 92.2 (dq, *J* = 200.5, 34.3 Hz), 60.5, 34.0 (d, *J* = 20.5 Hz).

**$^{19}$F NMR** (376 MHz, Chloroform-*d*) δ (ppm) = -79.87 (d, *J* = 10.8 Hz, 3F), -167.20 (q, *J* = 10.7 Hz, 1F).

**HRMS (ESI) m/z:** $[M + H]^+$ Calcd for $C_{16}H_{12}BrF_4N_2O$ 403.0064; Found 403.0060.

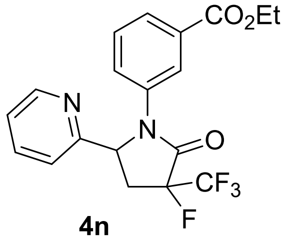


**4n**

Chemical Formula: $C_{19}H_{16}F_4N_2O_3$

**ethyl 3-(3-fluoro-2-oxo-5-(pyridin-2-yl)-3-(trifluoromethyl)pyrrolidin-1-yl)benzoate (4n)**: brown solid (mp = 81.6-82.3 °C), 33.9 mg, (Yield: 85%).

**$^1$H NMR** (400 MHz, Chloroform-*d*) δ (ppm) = 8.58 (dd, *J* = 5.3, 1.9 Hz, 1H), 8.03 (s, 1H), 7.84 (d, *J* = 8.1 Hz, 1H), 7.69 (dd, *J* = 8.1, 2.3 Hz, 1H), 7.61 (td, *J* = 7.7, 1.8 Hz, 1H), 7.37 (t, *J* = 8.0 Hz, 1H), 7.24 – 7.16 (m, 2H), 5.43 (ddd, *J* = 7.7, 5.8, 3.8 Hz, 1H), 4.33 (q, *J* = 7.1 Hz, 2H), 3.24 (ddd, *J* = 15.0, 13.5, 7.6 Hz, 1H), 2.75 (ddd, *J* = 25.8, 15.0, 5.9 Hz, 1H), 1.36 (t, *J* = 7.1 Hz, 3H).

**$^{13}$C NMR** (101 MHz, Chloroform-*d*) δ (ppm) = 165.5, 163.2 (d, *J* = 22.6 Hz), 156.9, 150.3, 137.3, 136.5, 131.4, 129.1, 127.7, 127.4, 123.7, 123.6, 121.8 (qd, *J* = 283.5, 31.7 Hz), 121.7, 92.2 (dq, *J* = 200.4, 34.2 Hz), 61.3, 60.5, 34.0 (d, *J* = 20.5 Hz), 14.2.

**$^{19}$F NMR** (376 MHz, Chloroform-*d*) δ (ppm) = -79.88 (d, *J* = 10.8 Hz, 3F), -167.33 (q, *J* = 11.0 Hz, 1F).

**HRMS (ESI) m/z:** $[M + H]^+$ Calcd for $C_{19}H_{17}F_4N_2O_3$ 397.1170; Found 397.1167.

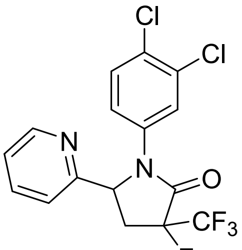

**4o**

Chemical Formula: $C_{16}H_{10}Cl_2F_4N_2O$

**1-(3,4-dichlorophenyl)-3-fluoro-5-(pyridin-2-yl)-3-(trifluoromethyl)pyrrolidin-2-one (4o)**: white solid (mp = 127.7-128.5 °C), 24.3 mg, (Yield: 61%).

**$^{1}$H NMR** (400 MHz, Chloroform-*d*) δ (ppm) = 8.63 – 8.56 (m, 1H), 7.72 – 7.63 (m, 2H), 7.33 (d, *J* = 8.8 Hz, 1H), 7.27 – 7.22 (m, 2H), 7.17 (d, *J* = 7.8 Hz, 1H), 5.34 (ddd, *J* = 7.9, 5.7, 3.6 Hz, 1H), 3.22 (td, *J* = 14.8, 7.7 Hz, 1H), 2.69 (dddd, *J* = 25.4, 15.2, 5.6, 1.3 Hz, 1H).

**$^{13}$C NMR** (101 MHz, Chloroform-*d*) δ (ppm) = 163.2 (d, *J* = 22.6 Hz), 156.7, 150.4, 137.5, 135.7, 133.1, 130.6, 130.5, 124.6, 123.8, 121.8, 121.7 (qd, *J* = 283.6, 31.7 Hz), 121.3, 92.1 (dq, *J* = 200.6, 34.3 Hz), 60.4, 34.0 (d, *J* = 20.5 Hz).

**$^{19}$F NMR** (376 MHz, Chloroform-*d*) δ (ppm) = -79.86 (d, *J* = 10.7 Hz, 3F), -167.17 (q, *J* = 10.8 Hz, 1F).

**HRMS (ESI) m/z:** $[M + H]^+$ Calcd for $C_{16}H_{11}Cl_2F_4N_2O$ 393.0179; Found 393.0178.

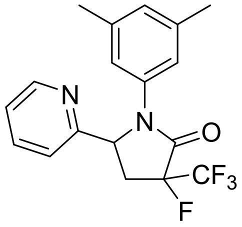

**4p**

Chemical Formula: $C_{18}H_{16}F_4N_2O$

**1-(3,5-dimethylphenyl)-3-fluoro-5-(pyridin-2-yl)-3-(trifluoromethyl)pyrrolidin-2-one (4p)**: white solid (mp = 138.4-139.0 °C), 26.6 mg, (Yield: 75%).

**$^{1}$H NMR** (400 MHz, Chloroform-*d*) δ (ppm) = 8.61 – 8.55 (m, 1H), 7.61 (td, *J* = 7.8, 1.8 Hz, 1H), 7.23 – 7.13 (m, 2H), 7.03 (s, 2H), 6.79 (s, 1H), 5.36 (ddd, *J* = 7.8, 5.5, 3.9 Hz, 1H), 3.19 (td, *J* = 15.0, 7.7 Hz, 1H), 2.67 (dddd, *J* = 25.6, 15.0, 5.6, 1.3 Hz, 1H), 2.22 (s, 6H).

**$^{13}$C NMR** (101 MHz, Chloroform-*d*) δ (ppm) = 163.1 (d, *J* = 22.2 Hz), 157.7, 149.9, 138.3, 137.2, 136.1, 128.6, 123.4, 121.3, 120.7, 119.2 (qd, *J* = 284.8, 32.3 Hz), 92.3 (dq, *J* = 199.6, 34.1 Hz), 60.7, 34.0 (d, *J* = 20.5 Hz), 21.2.

**$^{19}$F NMR** (376 MHz, Chloroform-*d*) δ (ppm) = -79.90 (d, *J* = 10.6 Hz, 3F), -167.08 (q, *J* = 10.7 Hz, 1F).

**HRMS (ESI) m/z:** $[M + H]^+$ Calcd for $C_{18}H_{17}F_4N_2O$ 353.1272; Found 353.1270.

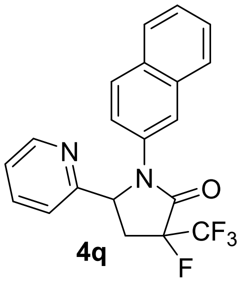

4q

Chemical Formula: $C_{20}H_{14}F_4N_2O$

**3-fluoro-1-(naphthalen-2-yl)-5-(pyridin-2-yl)-3-(trifluoromethyl)pyrrolidin-2-one (4q)**: white solid (mp = 157.9-158.3 °C), 24.4 mg, (Yield: 65%).

**$^1$H NMR** (400 MHz, Chloroform-*d*) δ (ppm) = 8.56 (d, *J* = 4.6 Hz, 1H), 7.91 (s, 1H), 7.77 – 7.70 (m, 3H), 7.59 – 7.50 (m, 2H), 7.47 – 7.39 (m, 2H), 7.22 – 7.11 (m, 2H), 5.57 – 5.47 (m, 1H), 3.27 (tdd, *J* = 14.8, 7.7, 1.4 Hz, 1H), 2.74 (ddd, *J* = 25.7, 15.0, 5.7 Hz, 1H).

**$^{13}$C NMR** (101 MHz, Chloroform-*d*) δ (ppm) = 163.3 (d, *J* = 22.3 Hz), 157.4, 150.1, 137.3, 133.8, 133.0, 131.6, 129.0, 127.9, 127.5, 126.6, 126.3, 123.5, 121.9 (qd, *J* = 284.6, 31.9 Hz), 121.5, 121.4, 121.0, 92.4 (dq, *J* = 199.9, 34.1 Hz), 60.7, 34.1 (d, *J* = 20.3 Hz).

**$^{19}$F NMR** (376 MHz,Chloroform-*d*) δ (ppm) = -79.79 (d, *J* = 11.0 Hz, 3F), -166.94 (q, *J* = 11.2 Hz, 1F).

**HRMS (ESI) m/z:** $[M + H]^+$ Calcd for $C_{20}H_{15}F_4N_2O$ 375.1115; Found 375.1113.

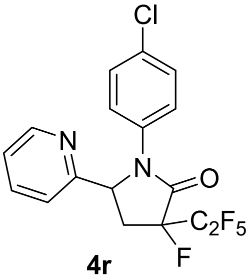

4r

Chemical Formula: $C_{17}H_{11}ClF_6N_2O$

**1-(4-chlorophenyl)-3-fluoro-3-(perfluoroethyl)-5-(pyridin-2-yl)pyrrolidin-2-one (4r)**: yellow solid (mp = 97.3-98.6 °C), 26.2 mg, (Yield: 64%).

**$^1$H NMR** (400 MHz, Chloroform-*d*) δ (ppm) = 8.59 – 8.57 (m, 1H), 7.62 (td, *J* = 7.8, 1.8 Hz, 1H), 7.41 – 7.37 (m, 2H), 7.27 – 7.20 (m, 3H), 7.15 (d, *J* = 7.8 Hz, 1H), 5.35 (ddd, *J* = 7.6, 5.8, 3.6 Hz, 1H), 3.33 (td, *J* = 14.9, 7.5 Hz, 1H), 2.73 (ddd, *J* = 25.8, 15.1, 5.9 Hz, 1H).

**$^{13}$C NMR** (101 MHz, Chloroform-*d*) δ (ppm) = 163.0 (d, *J* = 23.2 Hz), 157.0, 150.2, 137.3, 134.9, 132.2, 129.2, 124.1, 123.7, 121.5, 92.9 (dt, *J* = 201.3, 28.9 Hz), 60.7, 34.5 (d, *J* = 20.9 Hz). Carbons corresponding to the $C_2F_5$ group cannot be identified due to C-F coupling.

**$^{19}$F NMR** (376 MHz, Chloroform-*d*) δ (ppm) = -79.82 (d, *J* = 10.4 Hz, 3F), -123.91 – -127.22 (m, 2F), -165.66 (h, *J* = 9.7 Hz, 1F).

**HRMS (ESI) m/z:** $[M + H]^+$ Calcd for $C_{17}H_{12}ClF_6N_2O$ 409.0537; Found 409.0535.

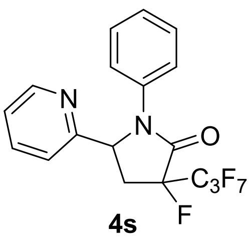

**4s**

Chemical Formula: $C_{18}H_{12}F_8N_2O$

**3-fluoro-3-(perfluoropropyl)-1-phenyl-5-(pyridin-2-yl)pyrrolidin-2-one (4s)**: white solid (mp = 93.6-94.2 °C), 30.3 mg, (Yield: 71%).

**$^1$H NMR** (400 MHz, Chloroform-*d*) δ (ppm) = 8.60 – 8.55 (m, 1H), 7.60 (td, *J* = 7.7, 1.8 Hz, 1H), 7.47 – 7.42 (m, 2H), 7.32 – 7.25 (m, 2H), 7.21 – 7.13 (m, 3H), 5.41 (ddd, *J* = 7.7, 5.4, 3.7 Hz, 1H), 3.34 (dddd, *J* = 16.4, 15.2, 7.7, 1.2 Hz, 1H), 2.75 (ddd, *J* = 25.6, 15.1, 5.5 Hz, 1H).

**$^{13}$C NMR** (101 MHz, Chloroform-*d*) δ (ppm) = 163.0 (d, *J* = 21.8 Hz), 157.5, 150.0, 137.2, 136.3, 129.0, 126.7, 123.5, 122.8, 121.3, 93.5 (dt, *J* = 197.1, 28.3 Hz), 60.7, 34.5 (d, *J* = 20.8 Hz). Carbons corresponding to the $C_3F_7$ group cannot be identified due to C-F coupling.

**$^{19}$F NMR** (376 MHz, Chloroform-*d*) δ (ppm) = -80.83 (td, *J* = 10.2, 3.9 Hz, 3F), -121.18 – -123.71 (m, 2F), -124.41 (dt, *J* = 10.3, 6.2 Hz, 2F), -164.45 (ttt, *J* = 13.2, 6.7, 3.9 Hz, 1F).

**HRMS (ESI) m/z:** $[M + H]^+$ Calcd for $C_{18}H_{13}F_8N_2O$ 425.0895; Found 425.0891.

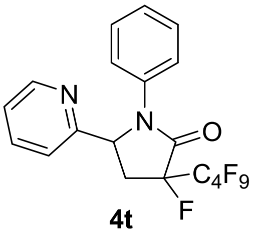

**4t**

Chemical Formula: $C_{19}H_{12}F_{10}N_2O$

**3-fluoro-3-(perfluorobutyl)-1-phenyl-5-(pyridin-2-yl)pyrrolidin-2-one (4t)**: white solid (mp = 90.3-91.6 °C), 33.4 mg, (Yield: 70%).

**$^1$H NMR** (400 MHz, Chloroform-*d*) δ (ppm) = 8.58 (dd, *J* = 4.7, 1.4 Hz, 1H), 7.60 (td, *J* = 7.7, 1.8 Hz, 1H), 7.48 – 7.41 (m, 2H), 7.32 – 7.25 (m, 2H), 7.22 – 7.13 (m, 3H), 5.41 (ddd, *J* = 7.7, 5.5, 3.7 Hz, 1H), 3.35 (td, *J* = 15.9, 8.1 Hz, 1H), 2.75 (ddd, *J* = 25.6, 15.1, 5.5 Hz, 1H).

**$^{13}$C NMR** (101 MHz, Chloroform-*d*) δ (ppm) = 163.0 (d, *J* = 22.6 Hz), 157.5, 150.1, 137.2, 136.4, 129.0, 126.7, 123.5, 122.8, 121.4, 93.7 (dt, *J* = 203.1, 28.8 Hz), 60.7, 34.6 (d, *J* = 20.6 Hz). Carbons corresponding to the $C_4F_9$ group cannot be identified due to C-F coupling.

**$^{19}$F NMR** (376 MHz, Chloroform-*d*) δ (ppm) = -80.77 (tt, *J* = 11.3, 2.8 Hz, 3F), -120.40 – -122.99 (m, 4F), -126.06 – -126.27 (m, 2F), -164.27 (tdq, *J* = 14.5, 10.4, 5.2 Hz, 1F).

**HRMS (ESI) m/z:** $[M + H]^+$ Calcd for $C_{19}H_{13}F_{10}N_2O$ 475.0863; Found 475.0860.

# VIII. References


(1) Y. Peng, C. Qin, X. Chen, J. Li, H. Li, W. Wang, *Asian J. Org. Chem*. **2017**, *6*, 694-697.

(2) A. Saxena, B. Choi, & H. W. Lam, *J. Am. Chem. Soc*. **134**, 8428-8431.

(3) (a) K. N. Lee, Z. Lei, & M.-Y. Ngai, *J. Am. Chem. Soc*. **139**, 5003-5006. (b) B. Zhou, Y. Hu, & C. Wang, *Angew. Chem. Int. Ed*. **54**, 13659-13663.

(4) Gaussian 16, Revision A.03, M. J. Frisch, G. W. Trucks, H. B. Schlegel, G. E. Scuseria, M. A. Robb, J. R. Cheeseman, G. Scalmani, V. Barone, G. A. Petersson, H. Nakatsuji, X. Li, M. Caricato, A. V. Marenich, J. Bloino, B. G. Janesko, R. Gomperts, B. Mennucci, H. P. Hratchian, J. V. Ortiz, A. F. Izmaylov, J. L. Sonnenberg, D. Williams-Young, F. Ding, F. Lipparini, F. Egidi, J. Goings, B. Peng, A. Petrone, T. Henderson, D. Ranasinghe, V. G. Zakrzewski, J. Gao, N. Rega, G. Zheng, W. Liang, M. Hada, M. Ehara, K. Toyota, R. Fukuda, J. Hasegawa, M. Ishida, T. Nakajima, Y. Honda, O. Kitao, H. Nakai, T. Vreven, K. Throssell, J. A. Montgomery, Jr., J. E. Peralta, F. Ogliaro, M. J. Bearpark, J. J. Heyd, E. N. Brothers, K. N. Kudin, V. N. Staroverov, T. A. Keith, R. Kobayashi, J. Normand, K. Raghavachari, A. P. Rendell, J. C. Burant, S. S. Iyengar, J. Tomasi, M. Cossi, J. M. Millam, M. Klene, C. Adamo, R. Cammi, J. W. Ochterski, R. L. Martin, K. Morokuma, O. Farkas, J. B. Foresman, and D. J. Fox, Gaussian, Inc., Wallingford CT, 2016.

(5) R. S. Paton, J. L. Mackey, W. H. Kim, J. H. Lee, S. J. Danishefsky, K. N. Houk, *J. Am. Chem. Soc*. **2010**, *132 (27)*, 9335−9340.

(6) J. G. Harrison, O. Gutierrez, N. Jana, T. G. Driver, D. J. Tantillo, *J. Am. Chem. Soc*. **2016**, *138*, 487−490

(7) A. V. Marenich, C. J. Cramer, D. G. Truhlar, *J. Phys. Chem. B* **2009,** *113*, 6378−6396.

(8) Legault, C. Y. CYLview, 1.0b; Université de Sherbrooke, 2009; http://www.cylview.org.

# NMR Spectra of the Substrates

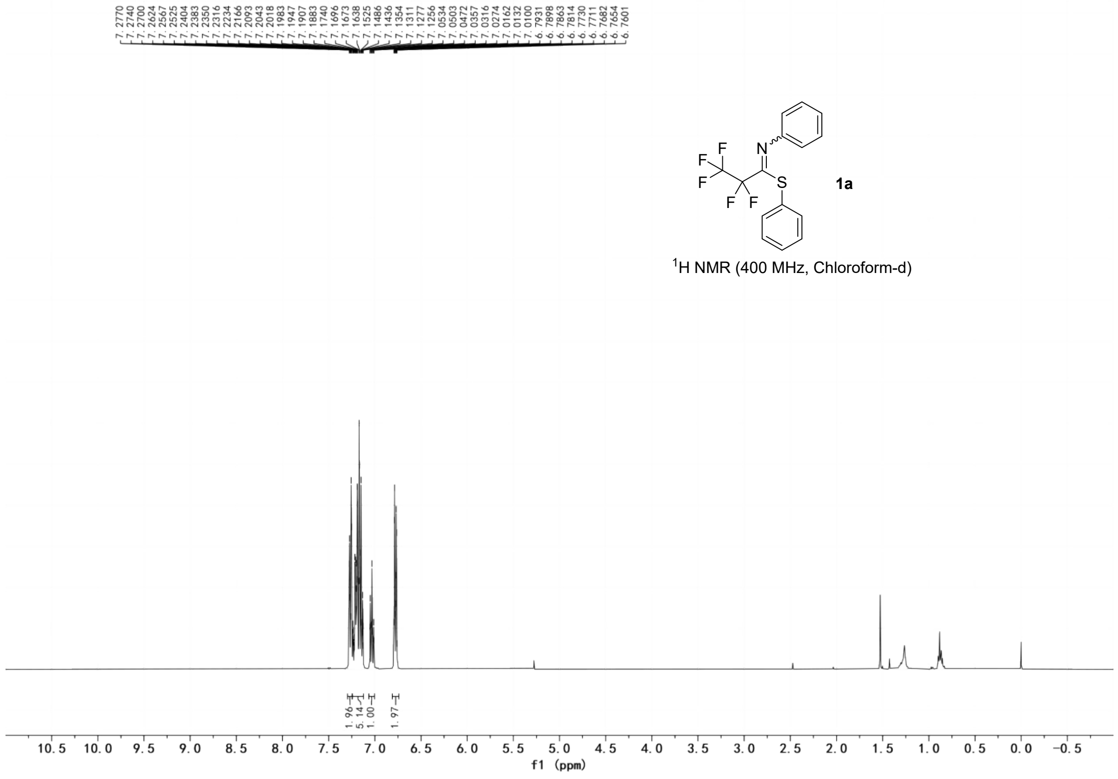

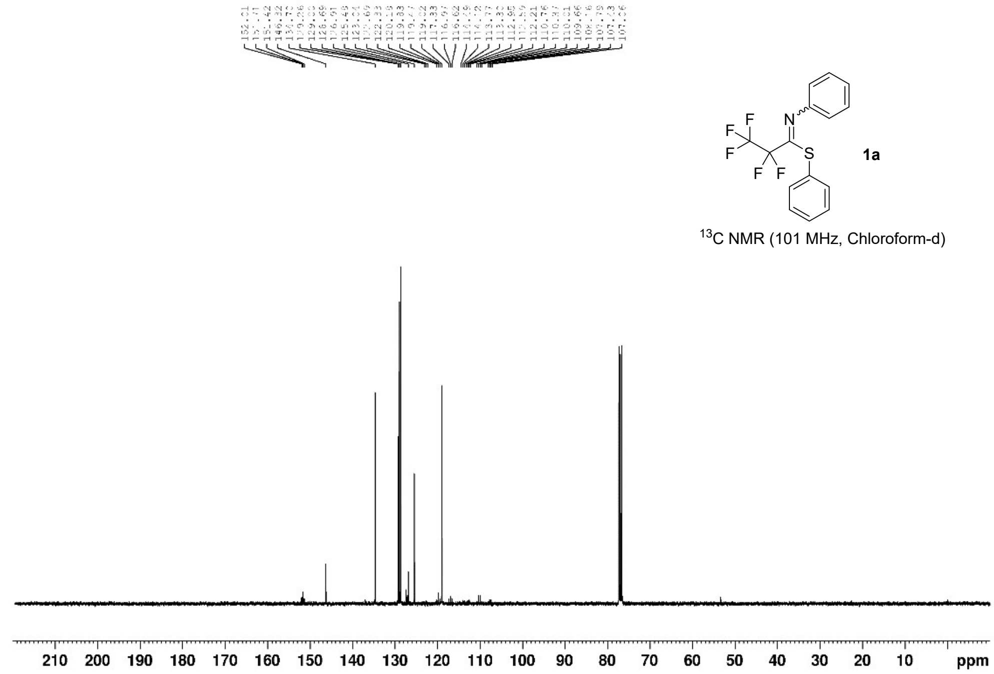
1a
$^{13}$C NMR (101 MHz, Chloroform-d)
210 200 190 180 170 160 150 140 130 120 110 100 90 80 70 60 50 40 30 20 10 ppm

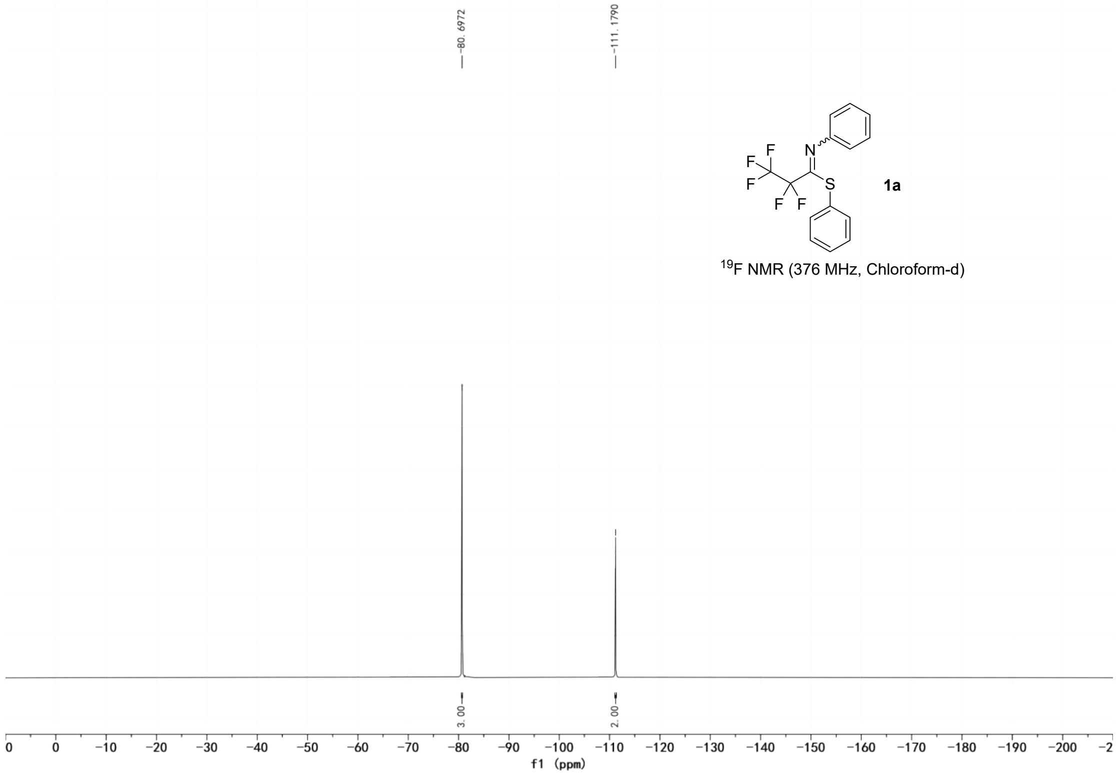
-80.6972
-111.1790
1a
$^{19}$F NMR (376 MHz, Chloroform-d)
3.00
2.00
0 0 −10 −20 −30 −40 −50 −60 −70 −80 −90 −100 −110 −120 −130 −140 −150 −160 −170 −180 −190 −200 −2
f1 (ppm)

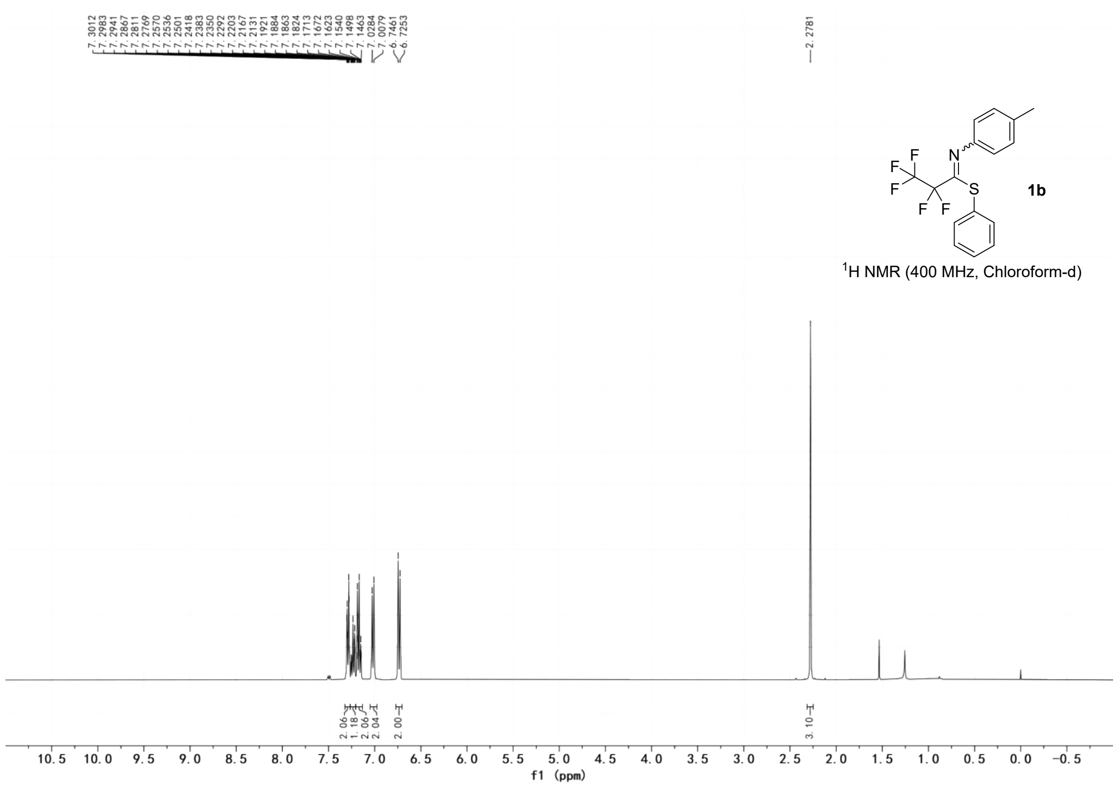
1b
$^1$H NMR (400 MHz, Chloroform-d)
f1 (ppm)

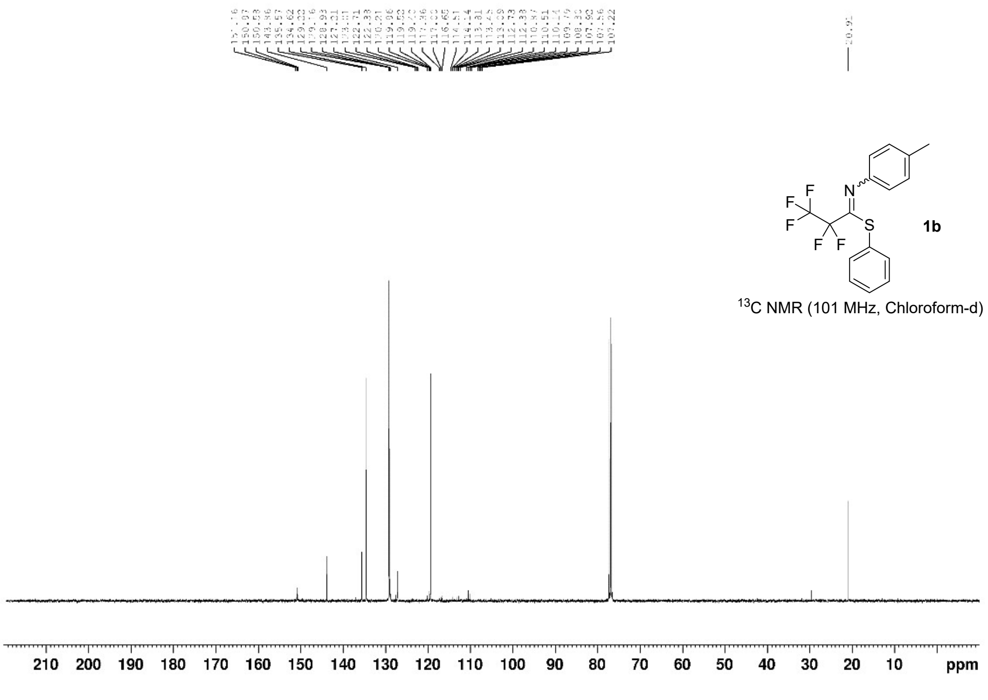
1b
$^{13}$C NMR (101 MHz, Chloroform-d)
ppm

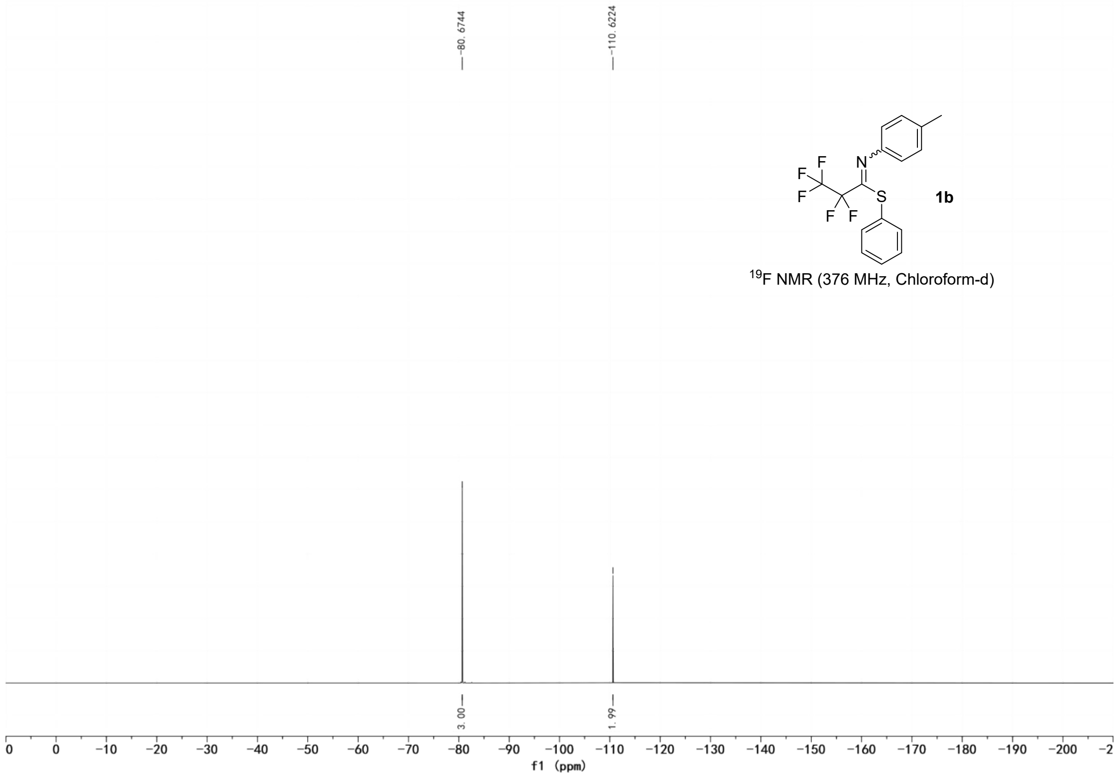

-80.6744
-110.6224
1b
19F NMR (376 MHz, Chloroform-d)
3.00
1.99
f1 (ppm)

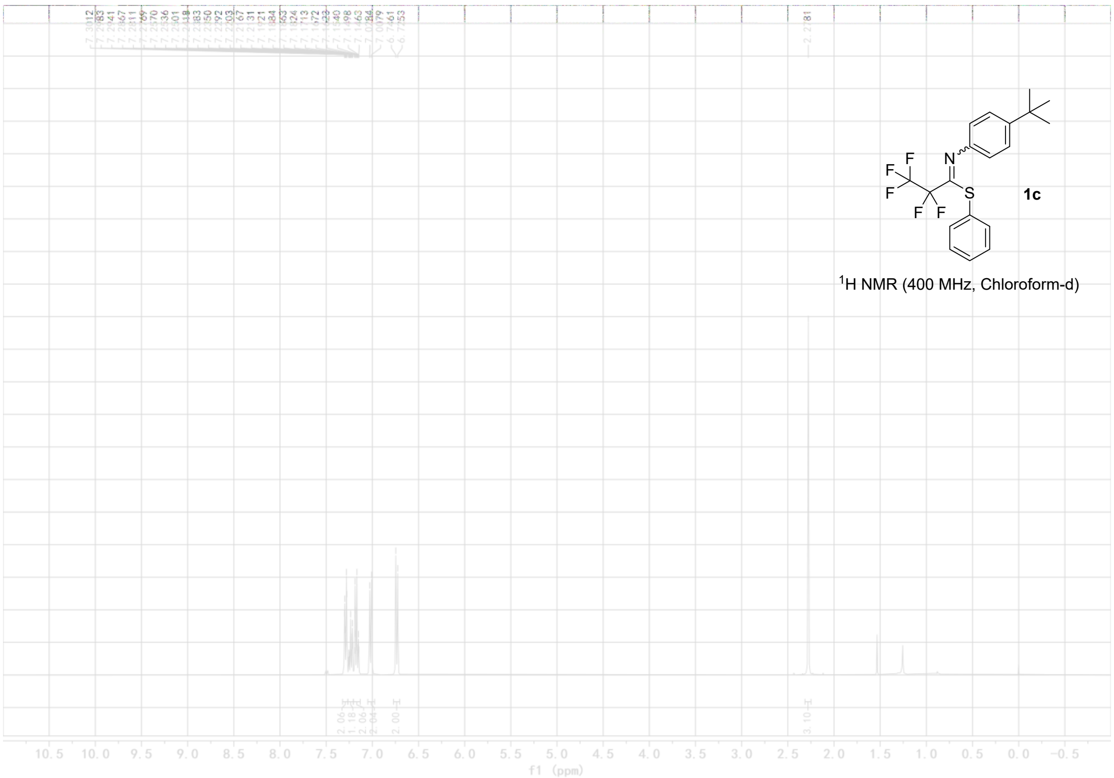
1c
1H NMR (400 MHz, Chloroform-d)
f1 (ppm)

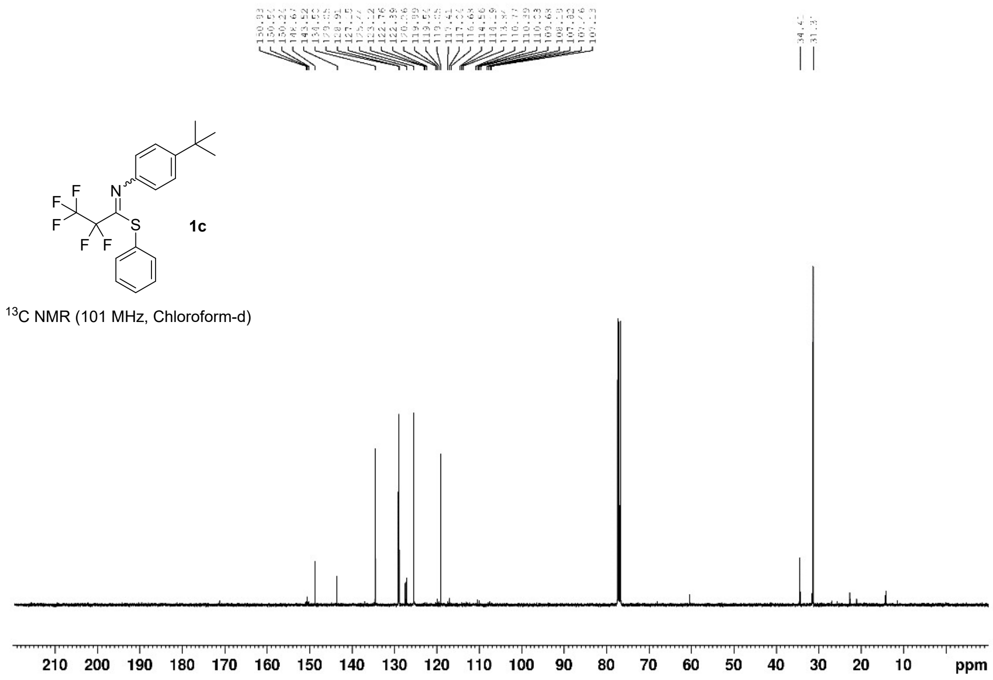
1c
13C NMR (101 MHz, Chloroform-d)
ppm

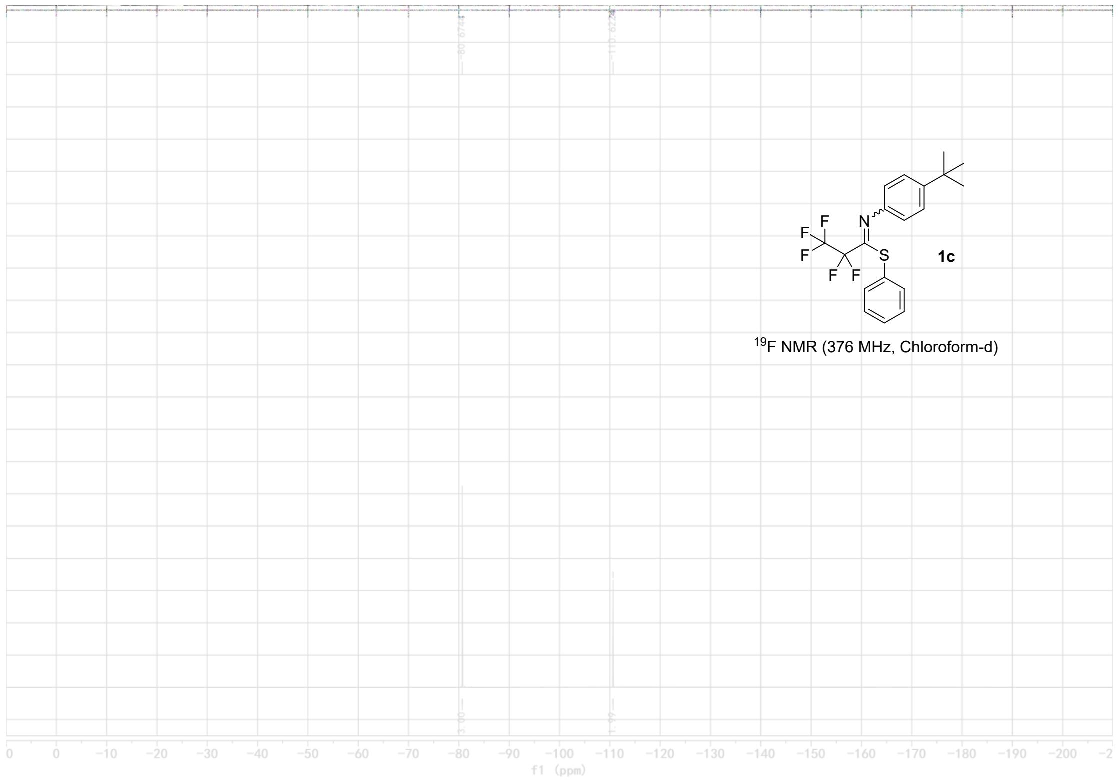
1c
19F NMR (376 MHz, Chloroform-d)

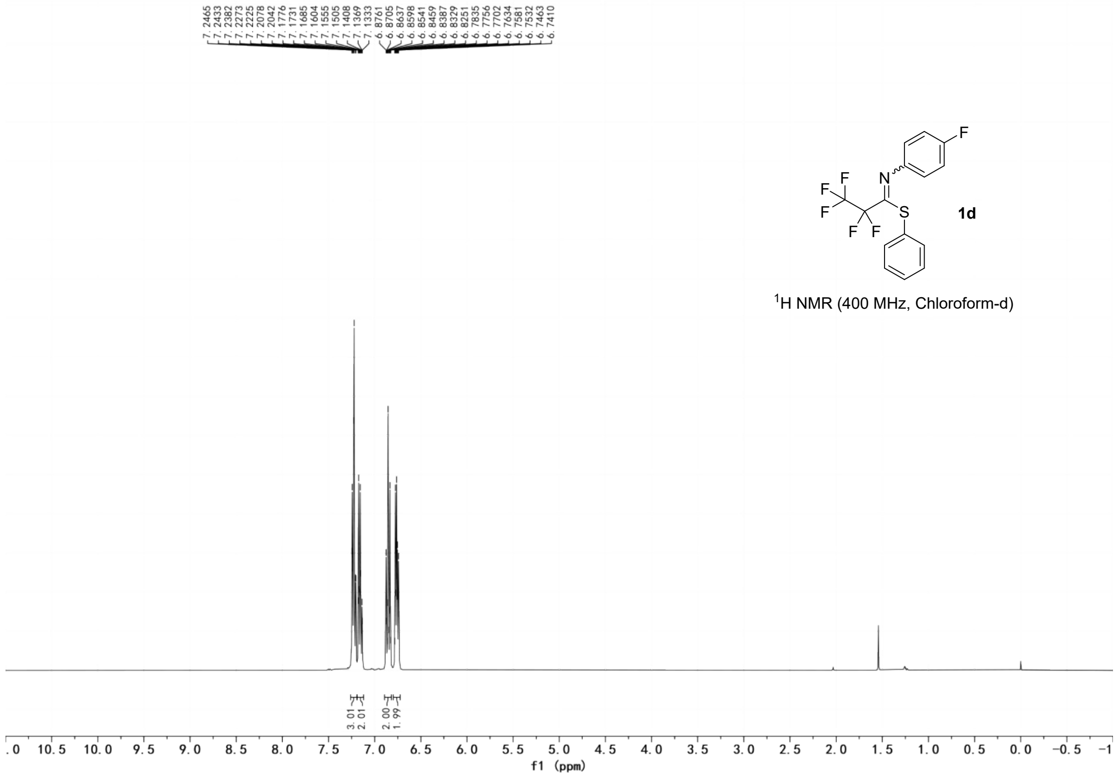
1d
1H NMR (400 MHz, Chloroform-d)
f1 (ppm)

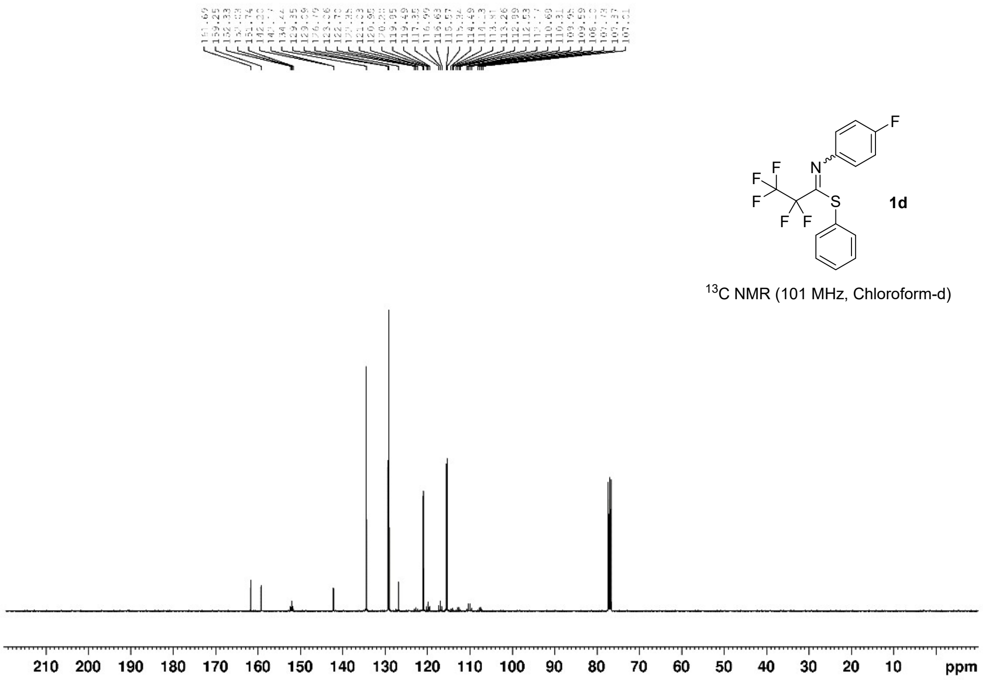
1d
13C NMR (101 MHz, Chloroform-d)
ppm

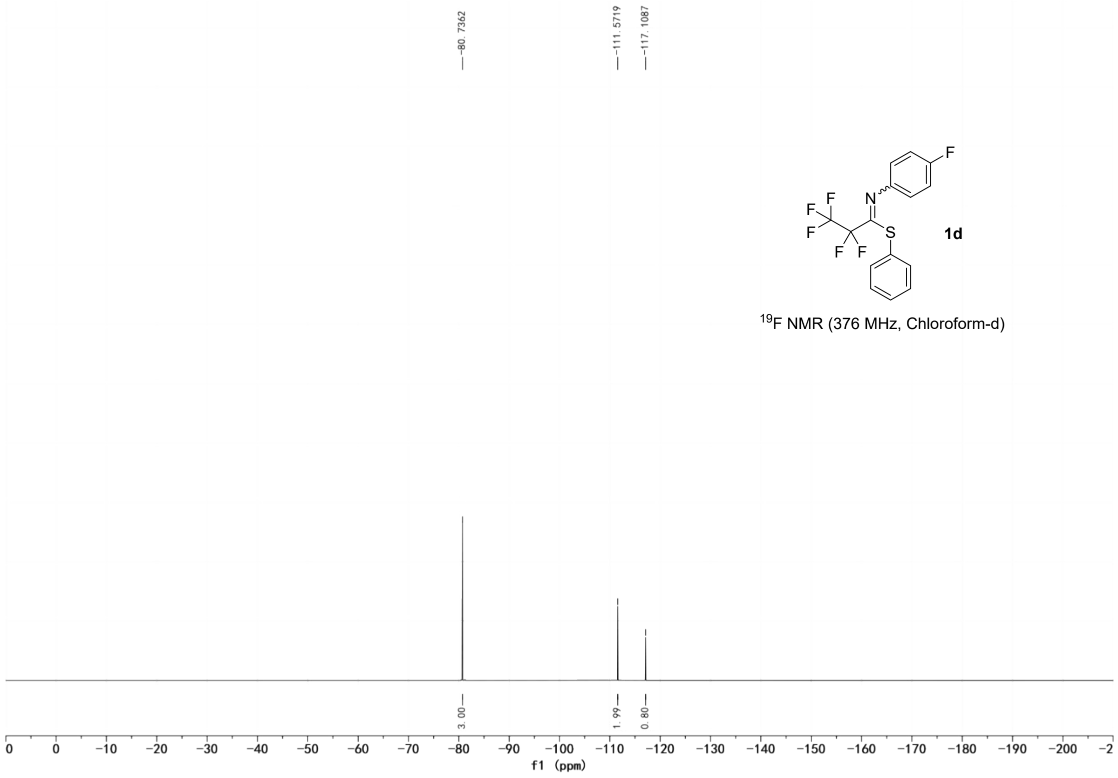


1d

$^{19}F$ NMR (376 MHz, Chloroform-d)

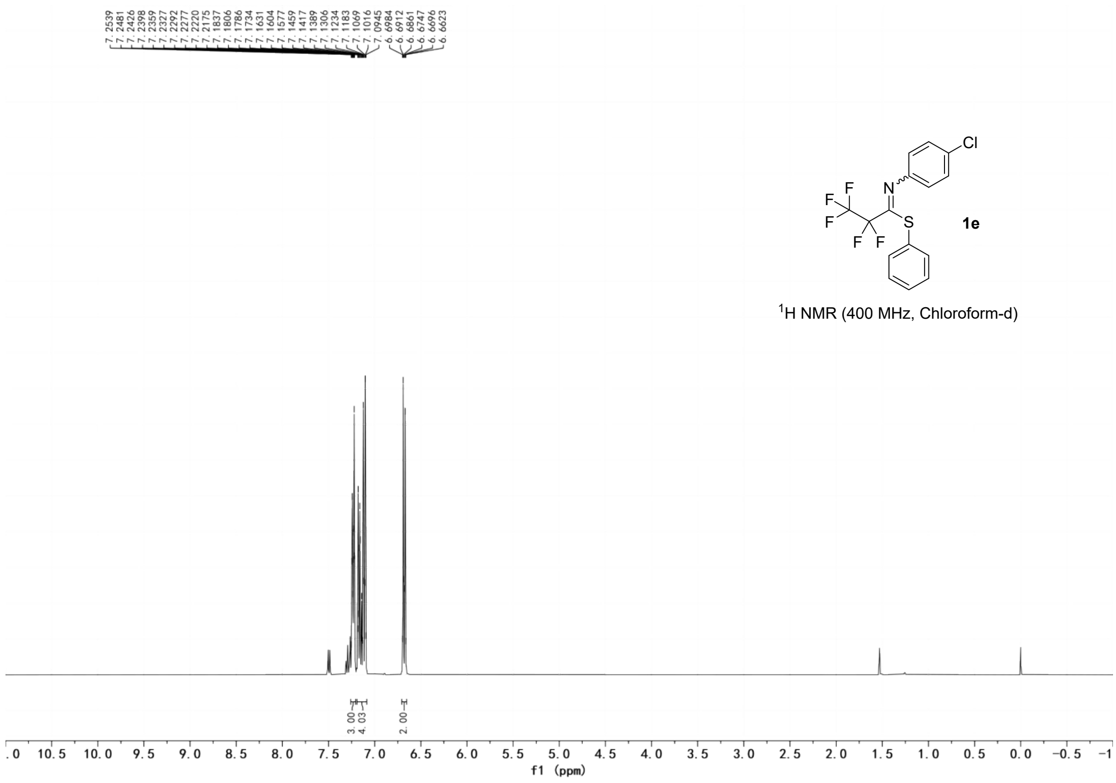
Cl
N
F
F
F
F
F
S
1e
$^{1}$H NMR (400 MHz, Chloroform-d)
3.00
4.03
2.00
f1 (ppm)

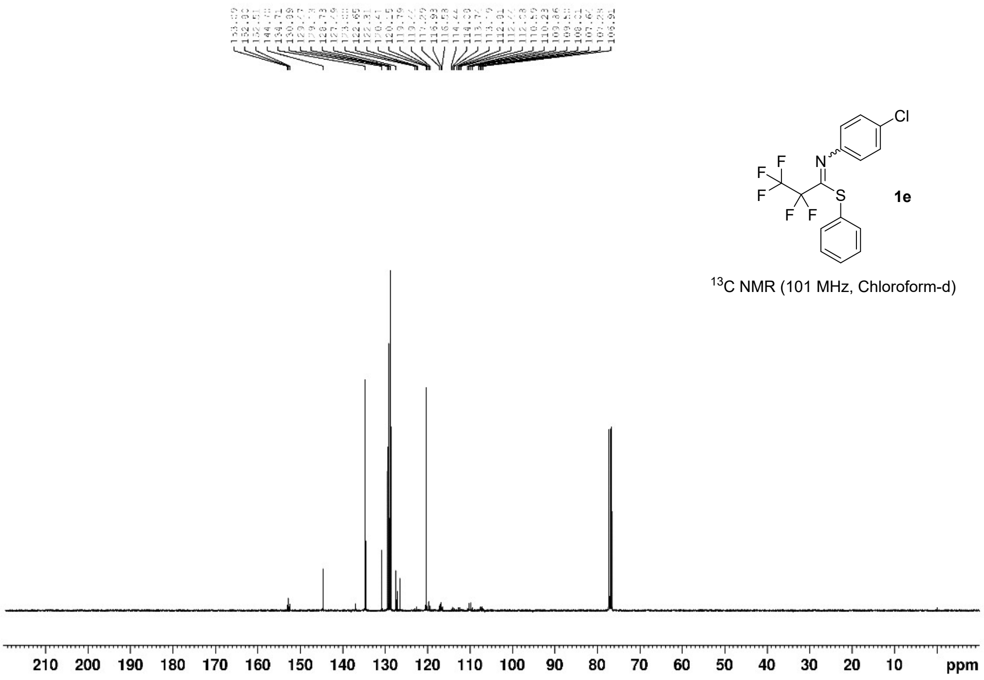
Cl
N
F
F
F
F
F
S
1e
$^{13}$C NMR (101 MHz, Chloroform-d)
ppm

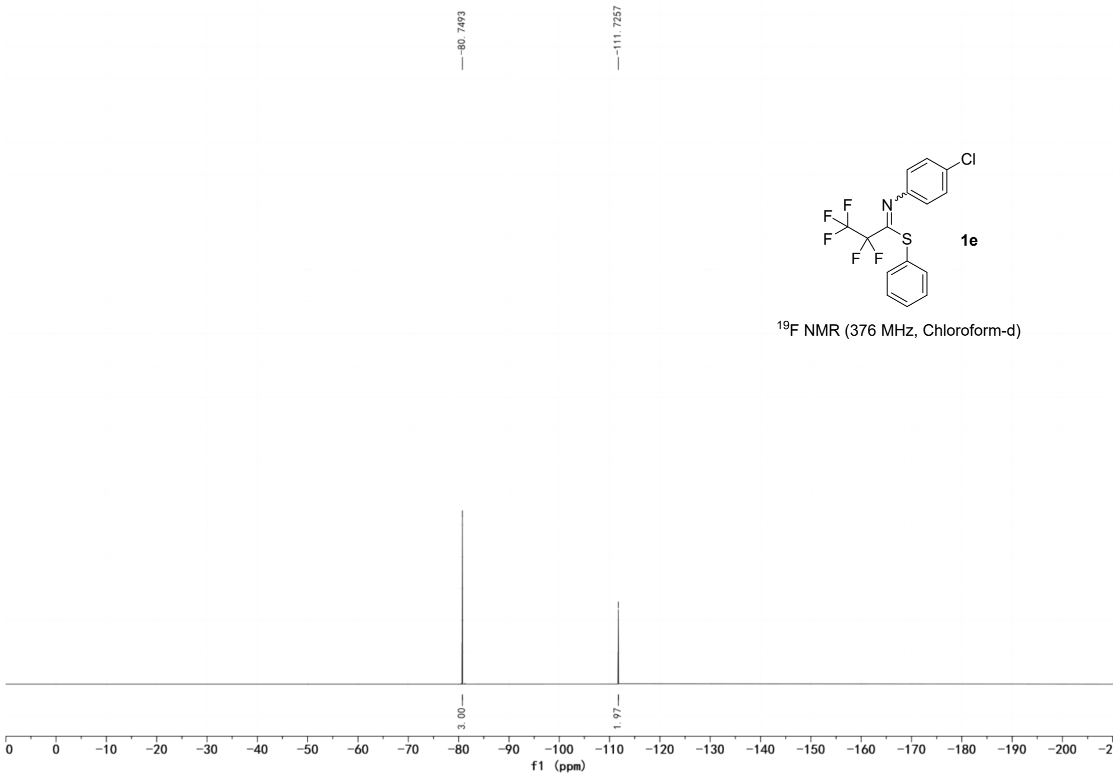
-80.7493
-111.7257
Cl
N
F
F
F
S
F
F
1e
19F NMR (376 MHz, Chloroform-d)
3.00
1.97
f1 (ppm)

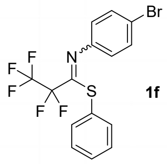

**1f**

$^{1}$H NMR (400 MHz, Chloroform-d)

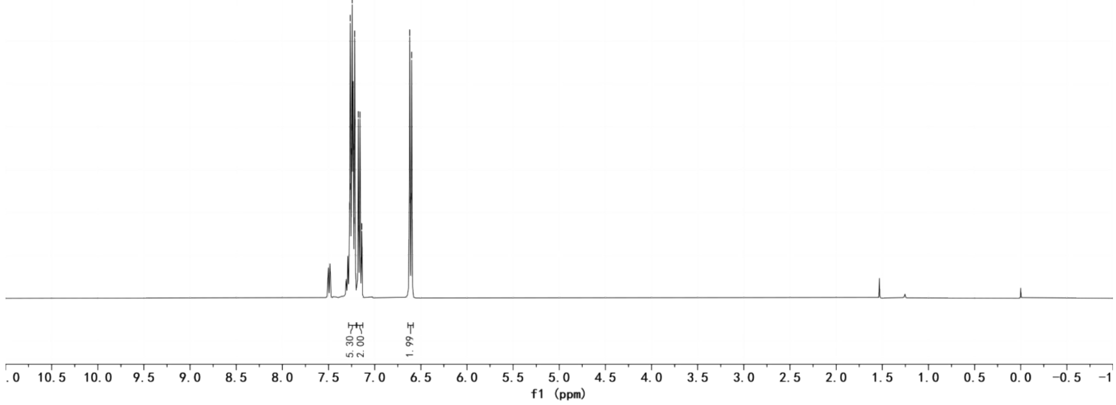


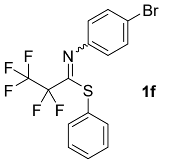

**1f**

$^{13}$C NMR (101 MHz, Chloroform-d)

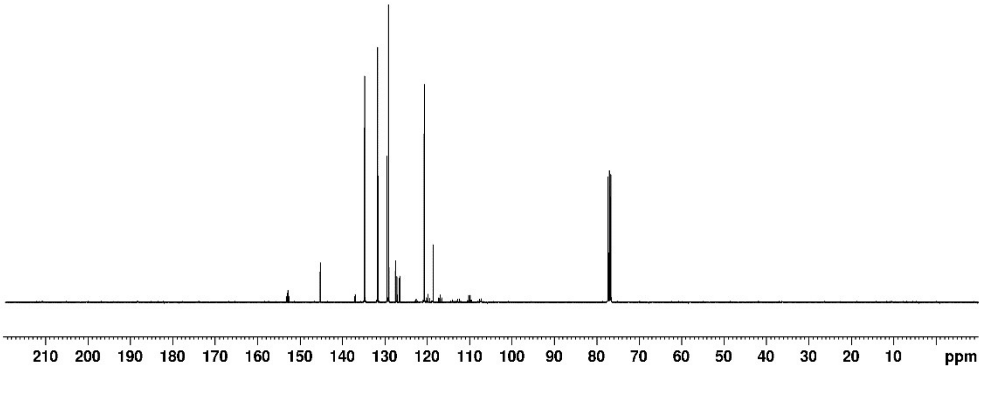

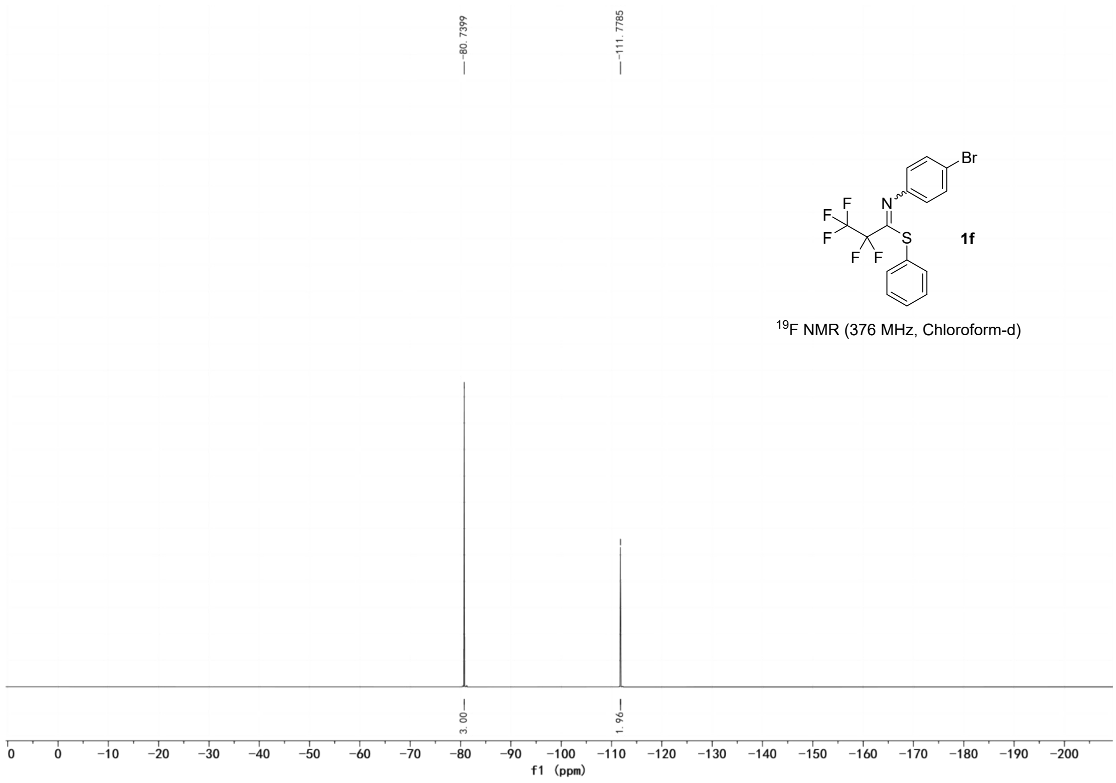
-80.7399
-111.7785
Br
N
F
F
F
S
F
F
1f
19F NMR (376 MHz, Chloroform-d)
3.00
1.96
0
0
-10
-20
-30
-40
-50
-60
-70
-80
-90
-100
-110
-120
-130
-140
-150
-160
-170
-180
-190
-200
f1 (ppm)

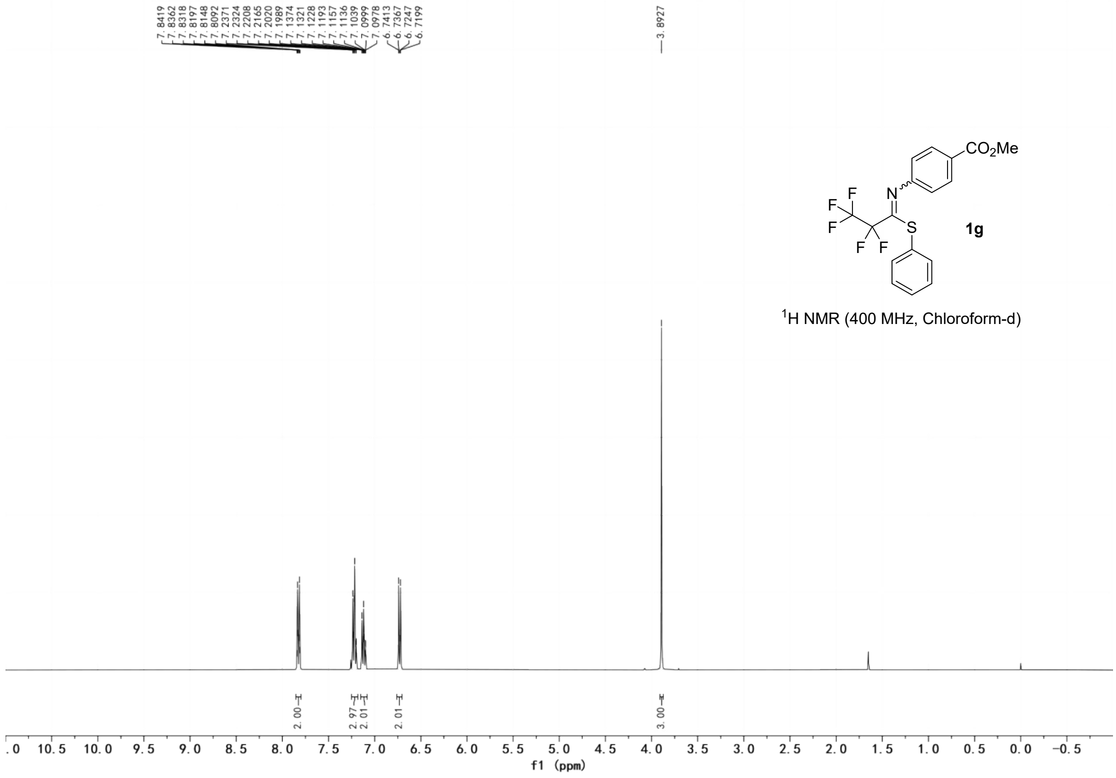


$^{1}H$ NMR (400 MHz, Chloroform-d)

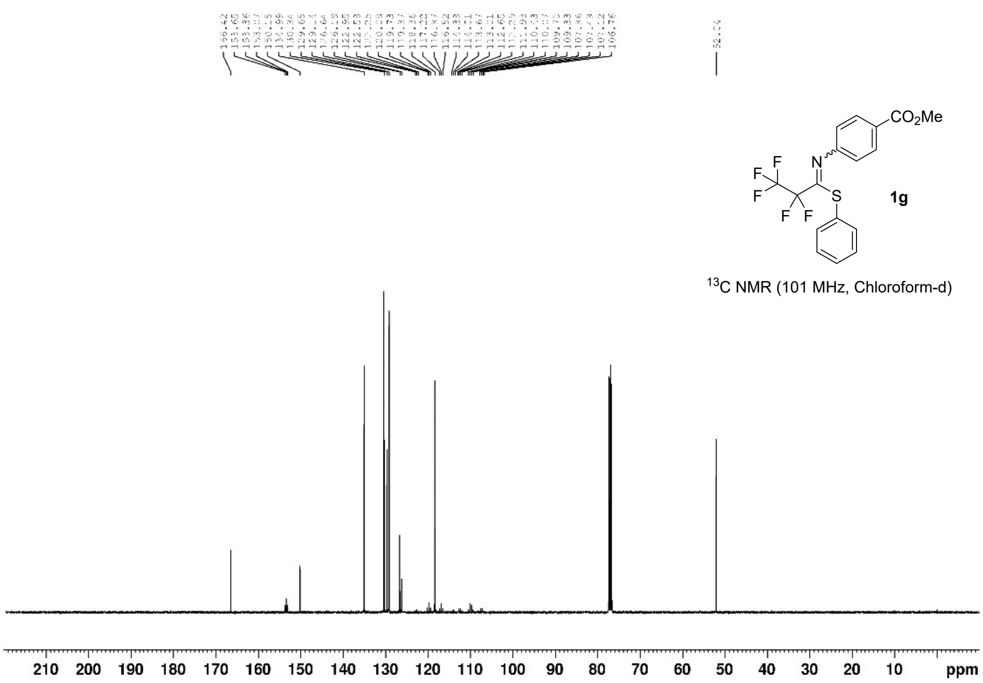


$^{13}C$ NMR (101 MHz, Chloroform-d)

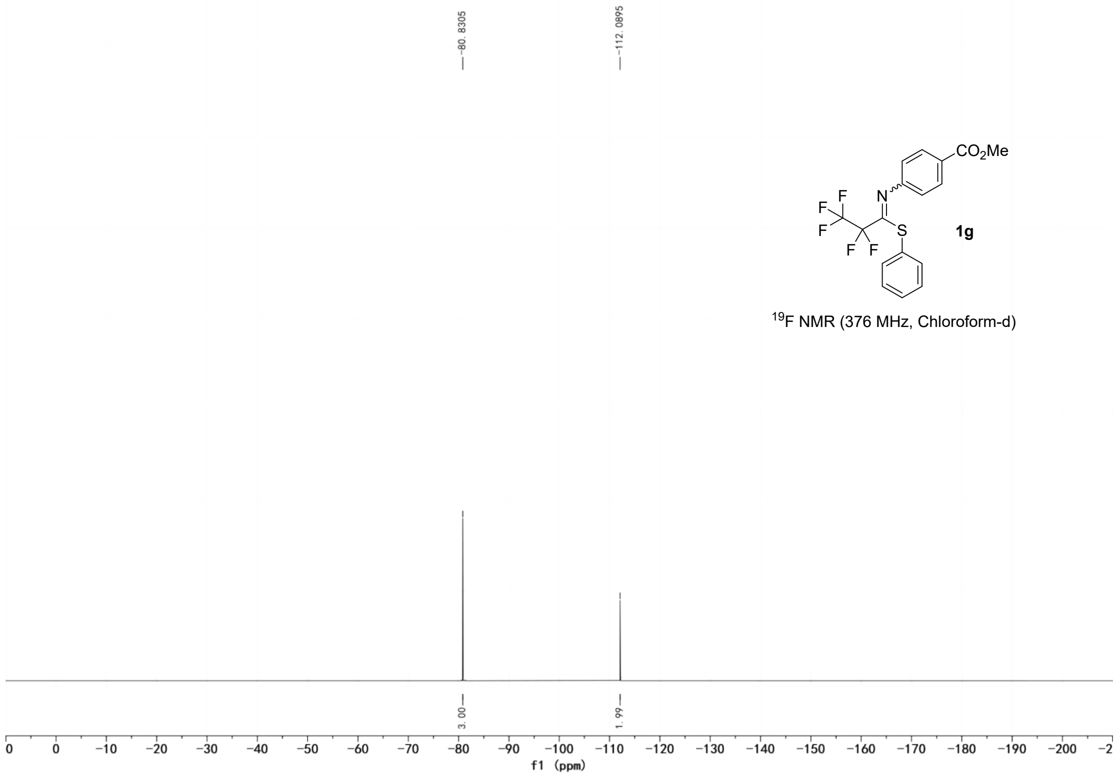

-80.8305
-112.0895
$CO_2Me$
N
F
S
1g
$^{19}$F NMR (376 MHz, Chloroform-d)
3.00
1.99
f1 (ppm)

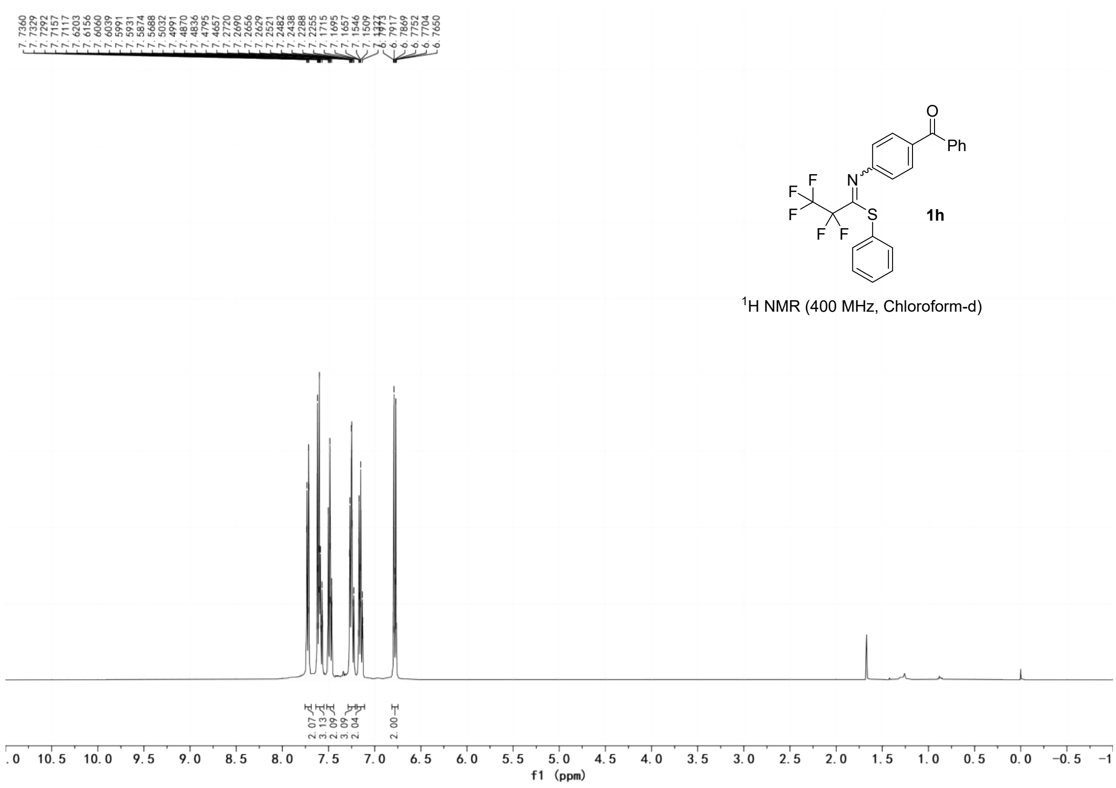
O
Ph
N
F
F
F
S
F
F
1h
$^{1}$H NMR (400 MHz, Chloroform-d)
2.07
3.13
2.09
3.09
2.04
2.00
f1 (ppm)

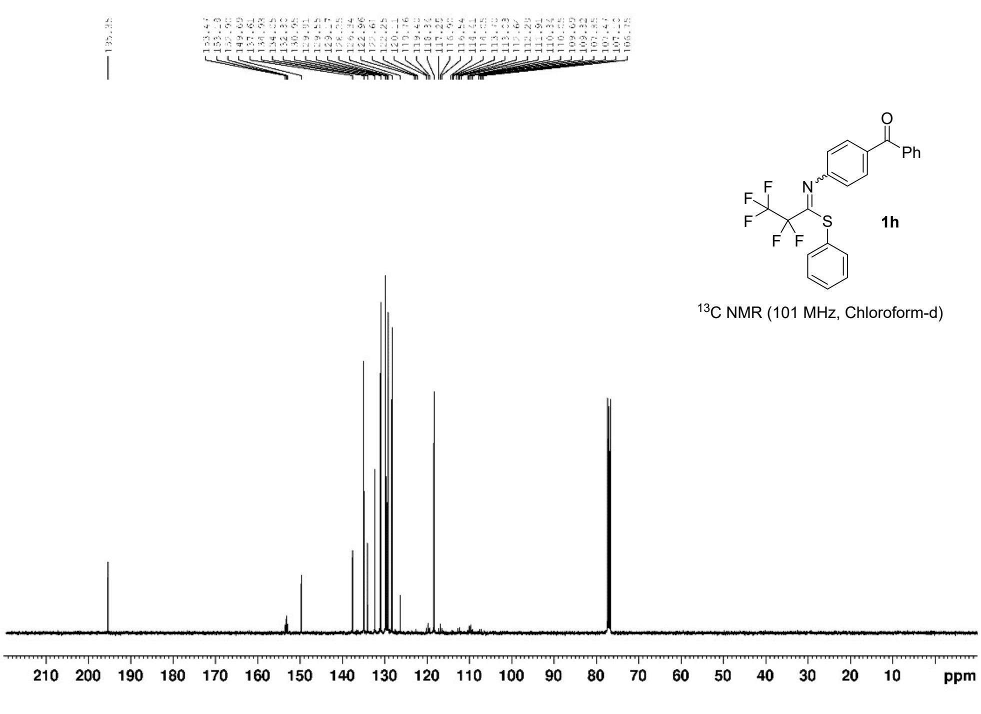
O
Ph
N
F
F
F
S
F
F
1h
$^{13}$C NMR (101 MHz, Chloroform-d)
ppm

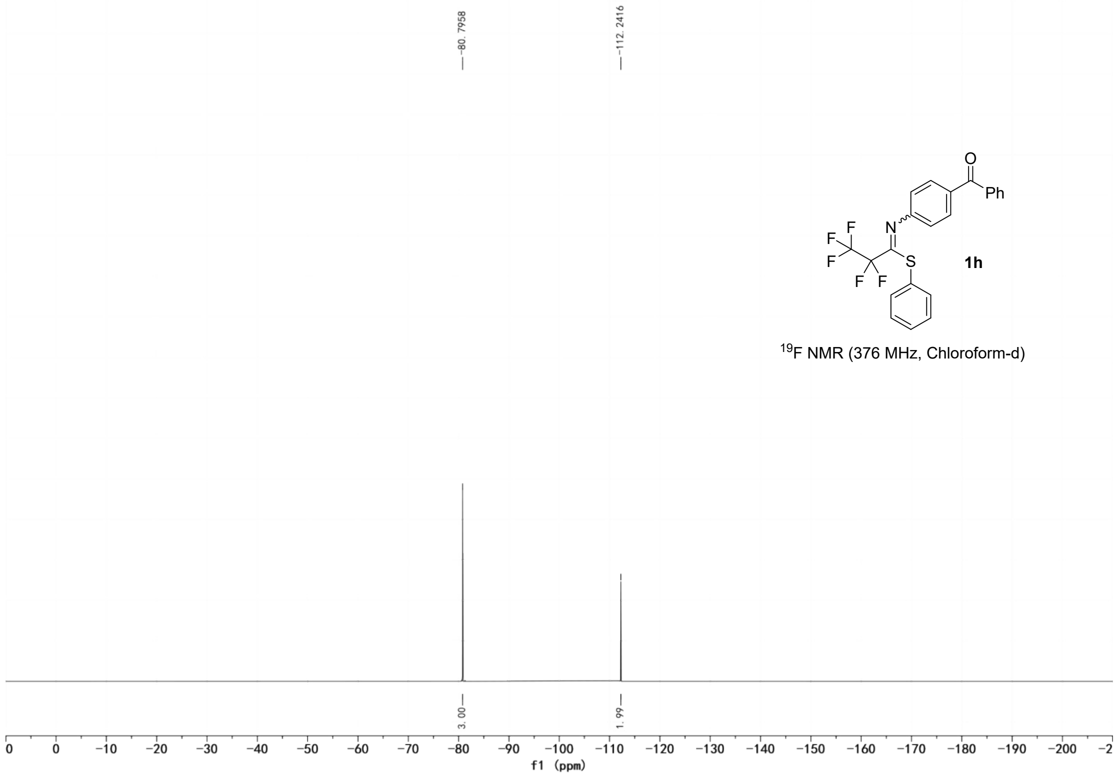


**1h**

$^{19}$F NMR (376 MHz, Chloroform-d)

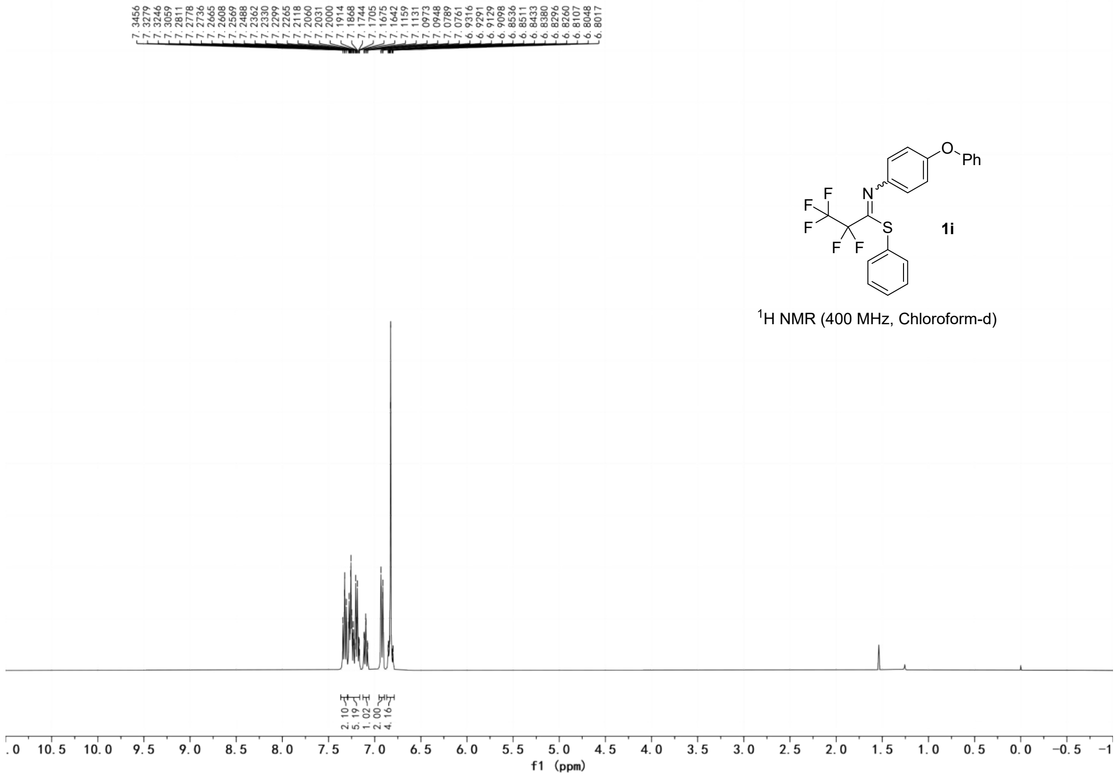
1i
$^1$H NMR (400 MHz, Chloroform-d)
f1 (ppm)

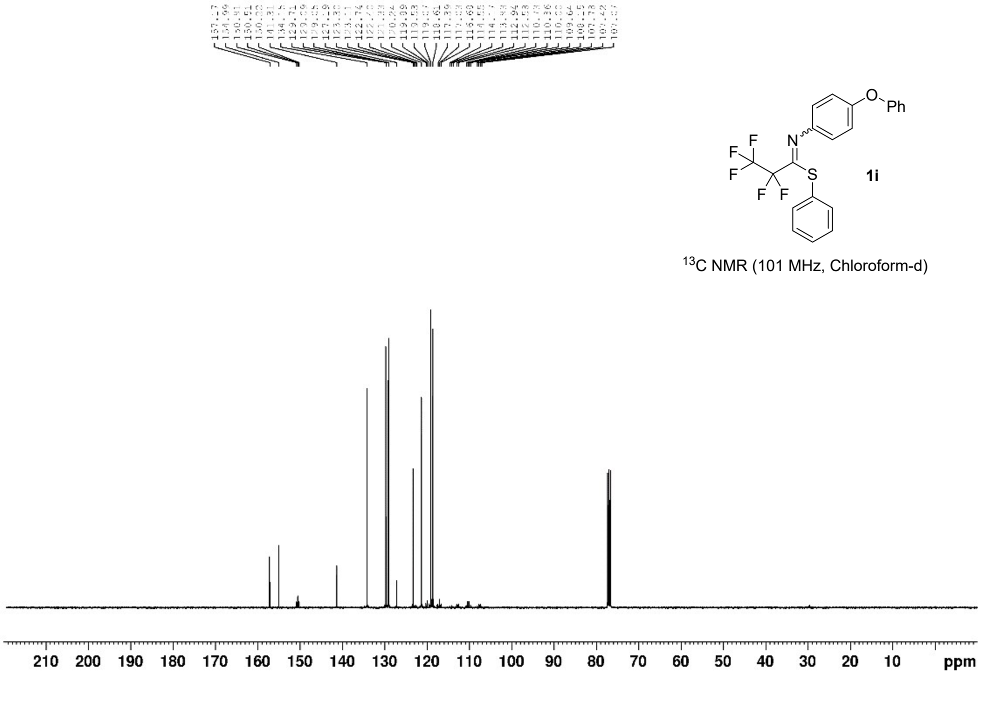
1i
$^{13}$C NMR (101 MHz, Chloroform-d)
ppm

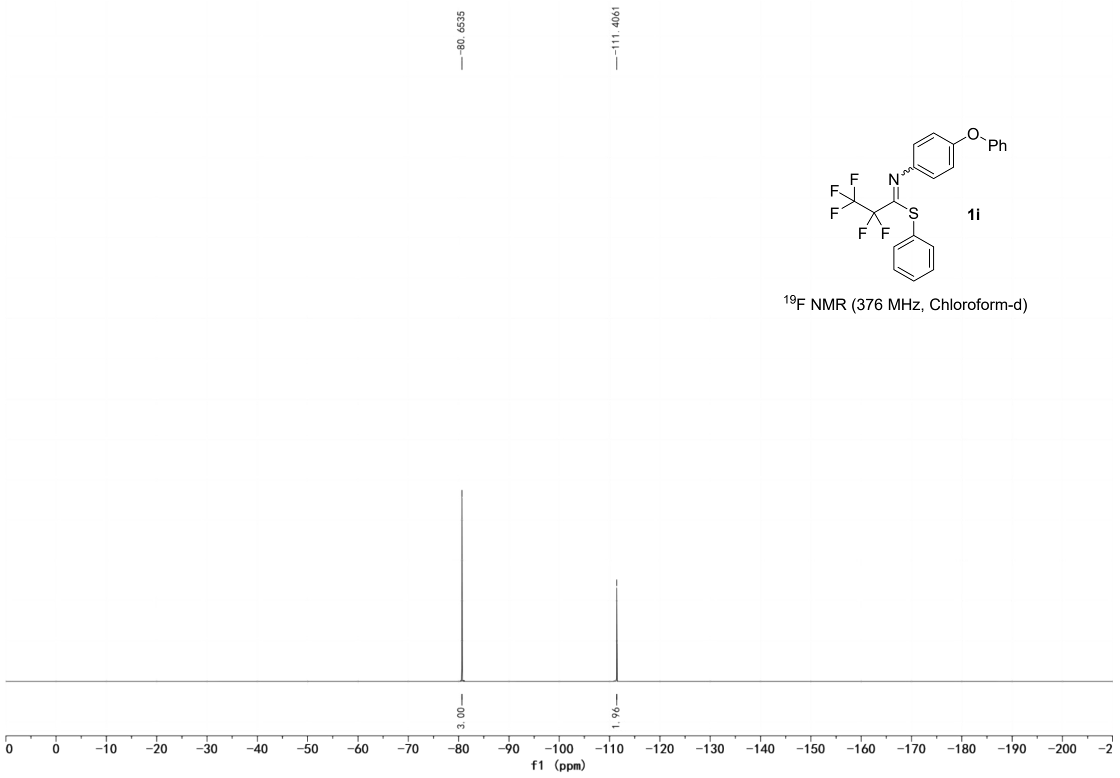

-80.6535
-111.4061
O
Ph
N
F
F
F
S
F
F
1i
19F NMR (376 MHz, Chloroform-d)
3.00
1.96
f1 (ppm)

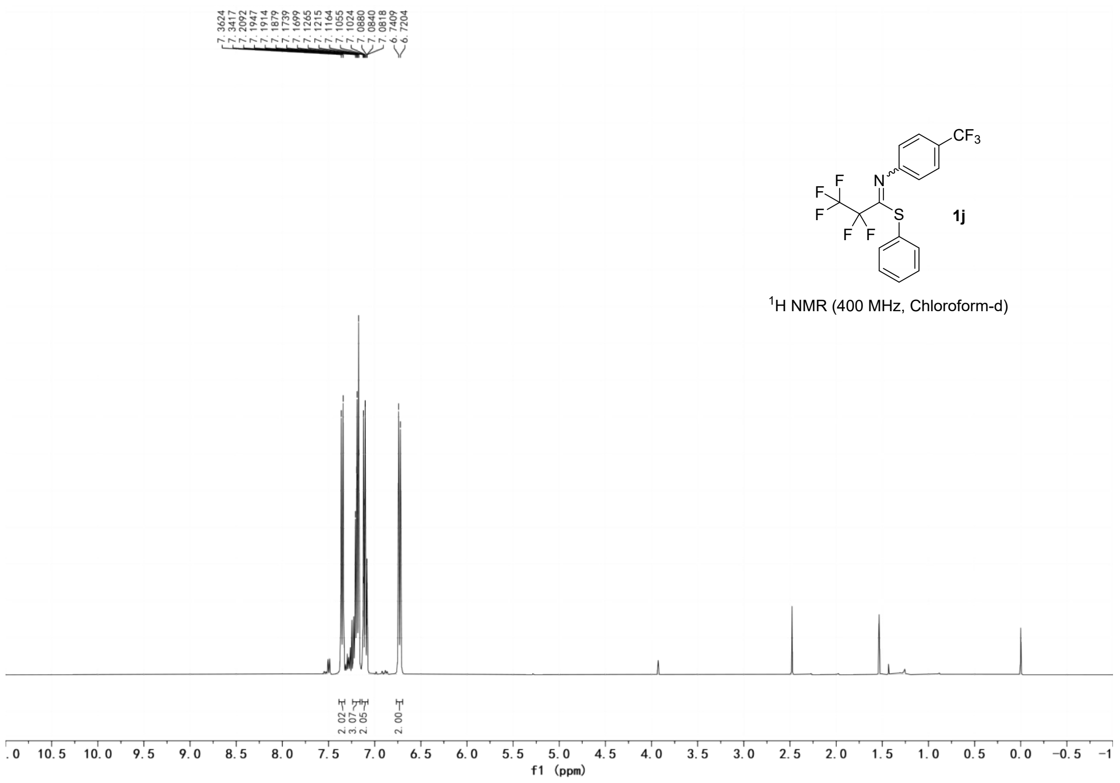

1j
$^1$H NMR (400 MHz, Chloroform-d)
f1 (ppm)


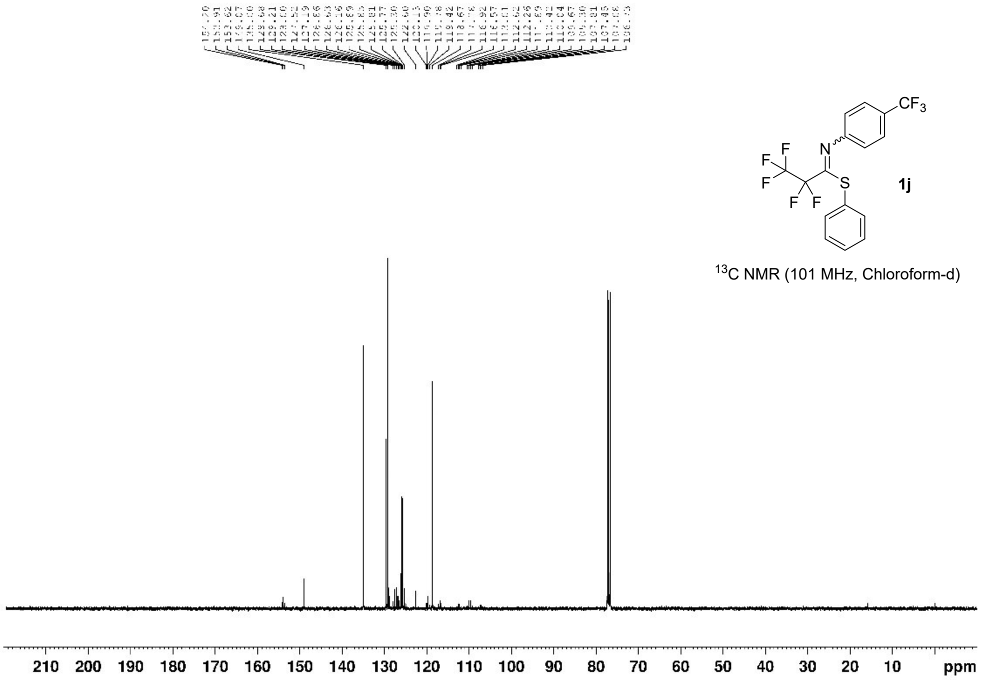

1j
$^{13}$C NMR (101 MHz, Chloroform-d)
ppm

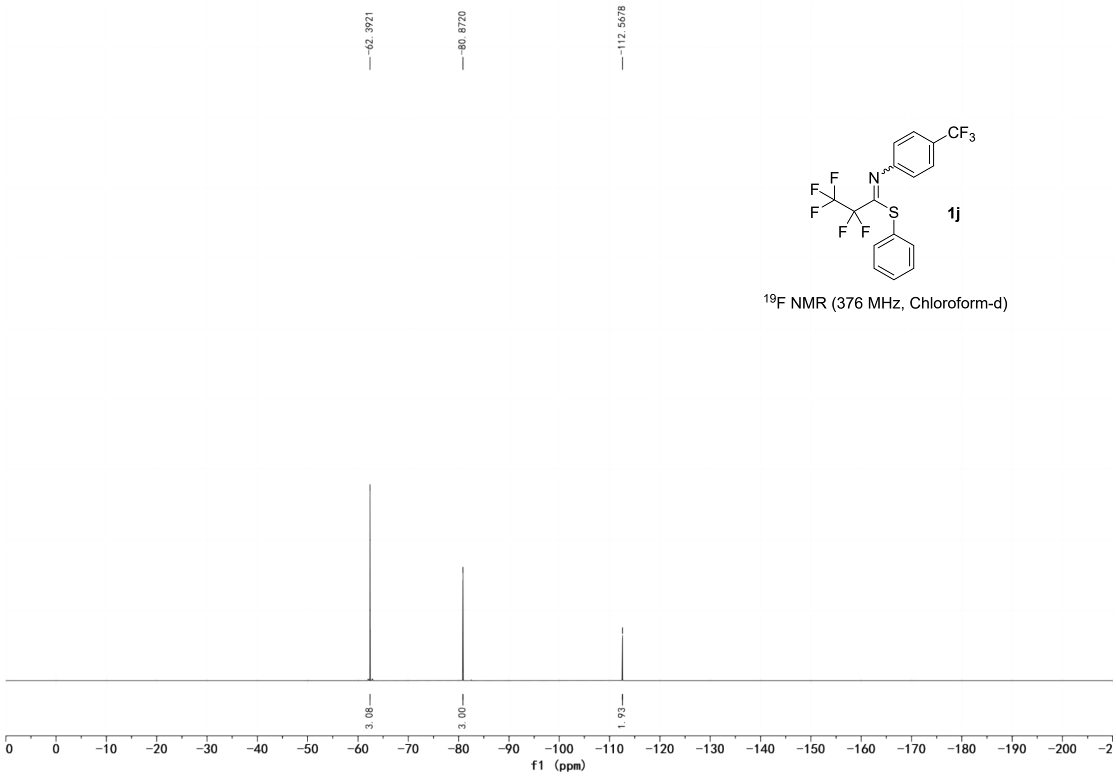


**1j**

$^{19}F$ NMR (376 MHz, Chloroform-d)

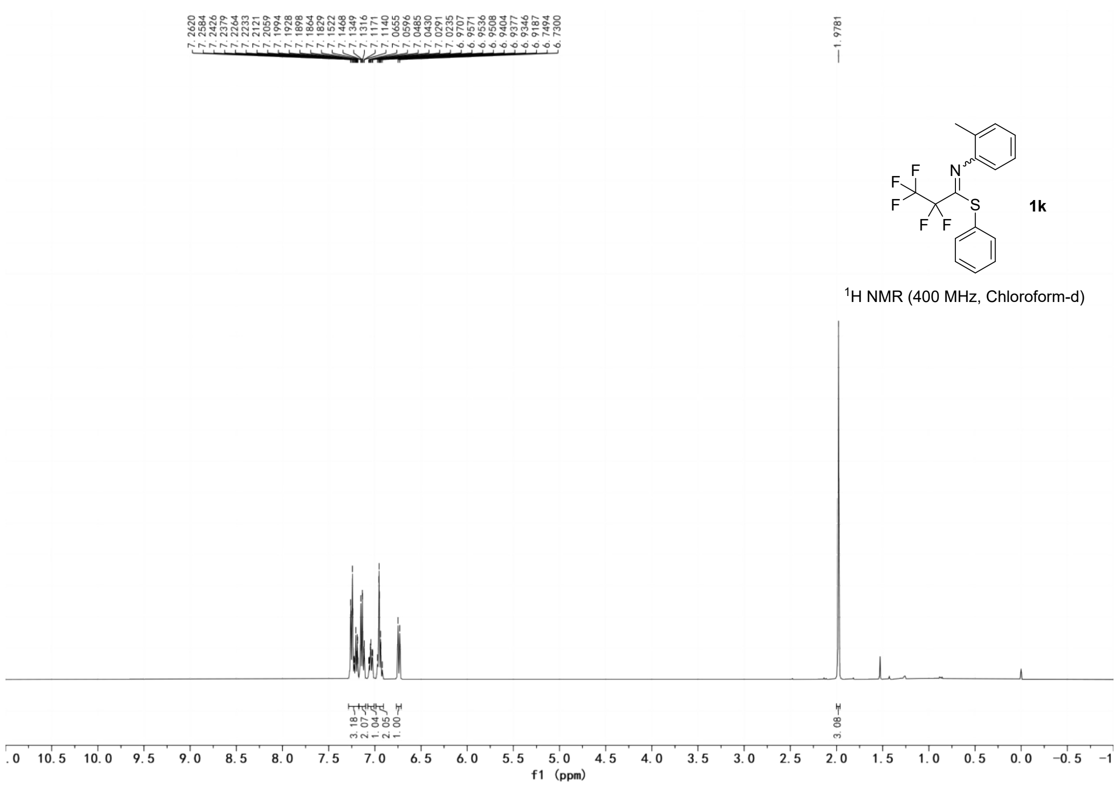
1k
1H NMR (400 MHz, Chloroform-d)
f1 (ppm)

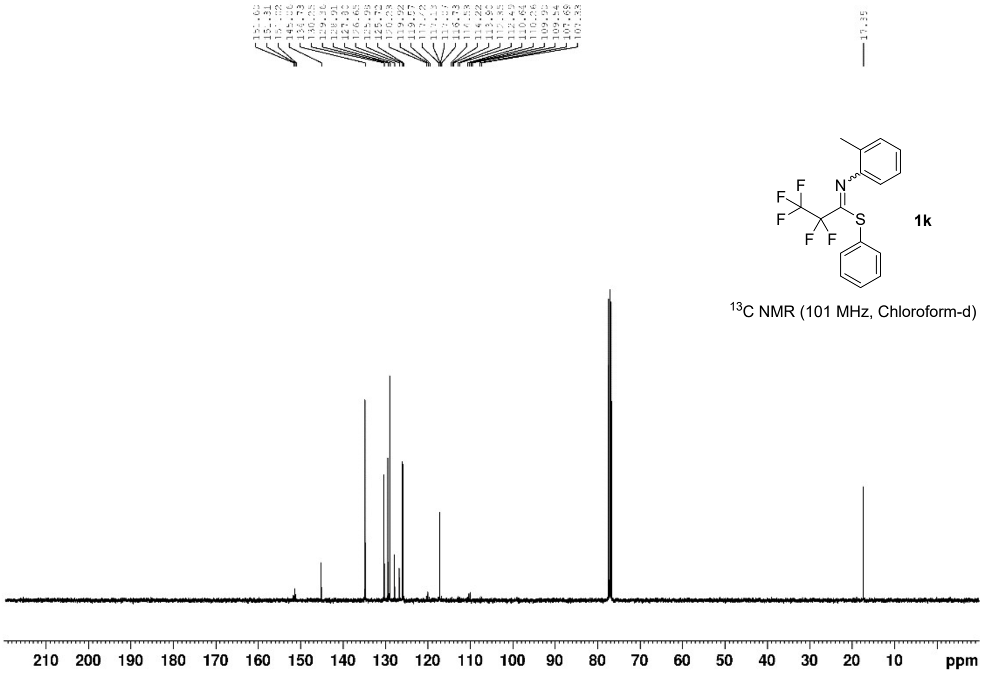
1k
13C NMR (101 MHz, Chloroform-d)
ppm

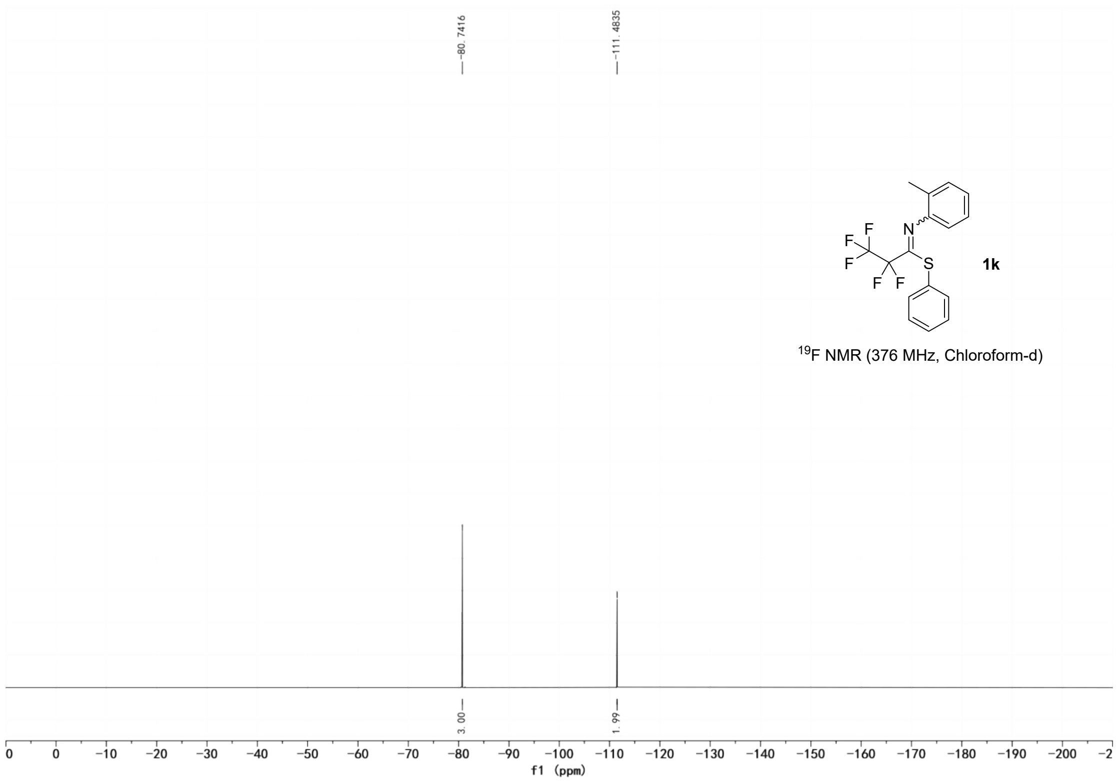

-80.7416
-111.4835
1k
19F NMR (376 MHz, Chloroform-d)
3.00
1.99
f1 (ppm)

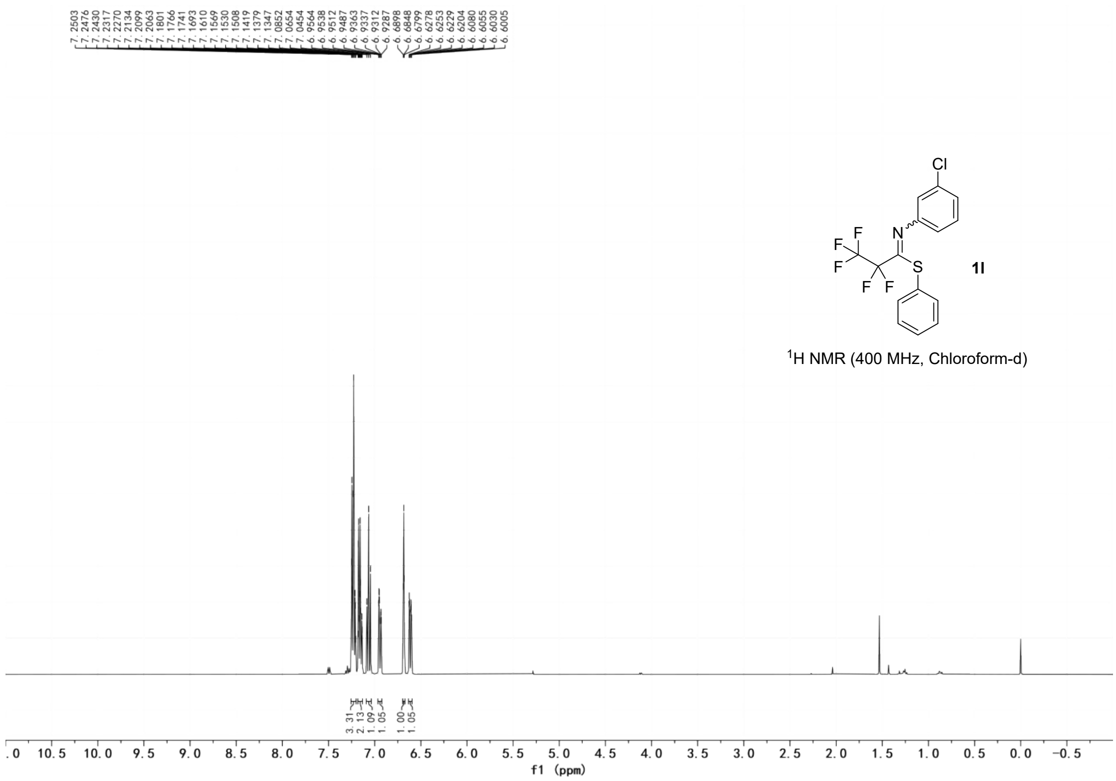

1l

$^{1}$H NMR (400 MHz, Chloroform-d)

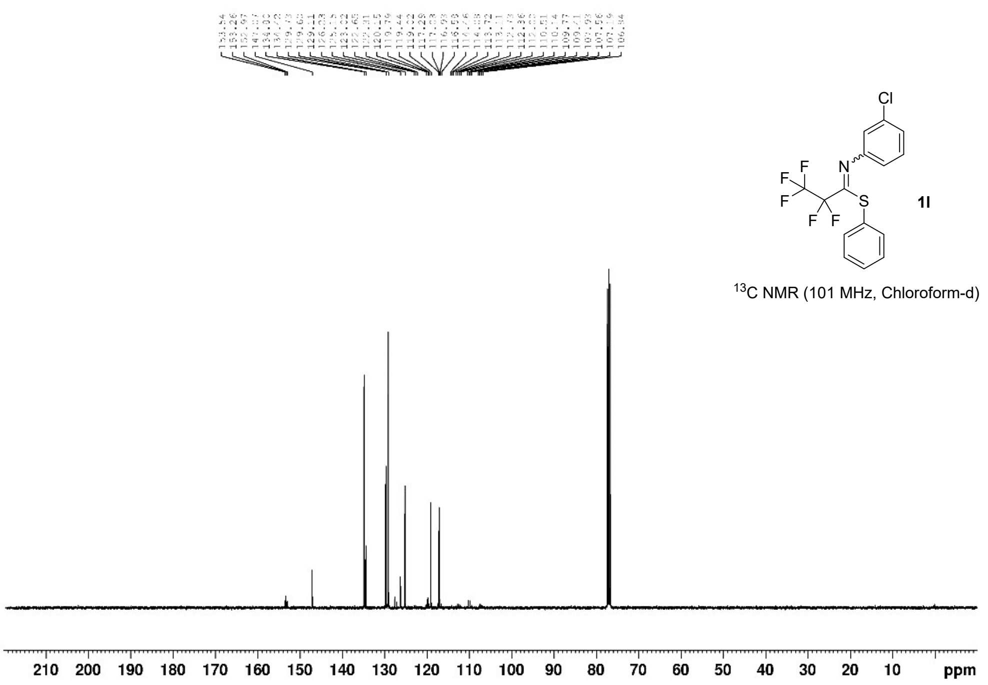

1l

$^{13}$C NMR (101 MHz, Chloroform-d)

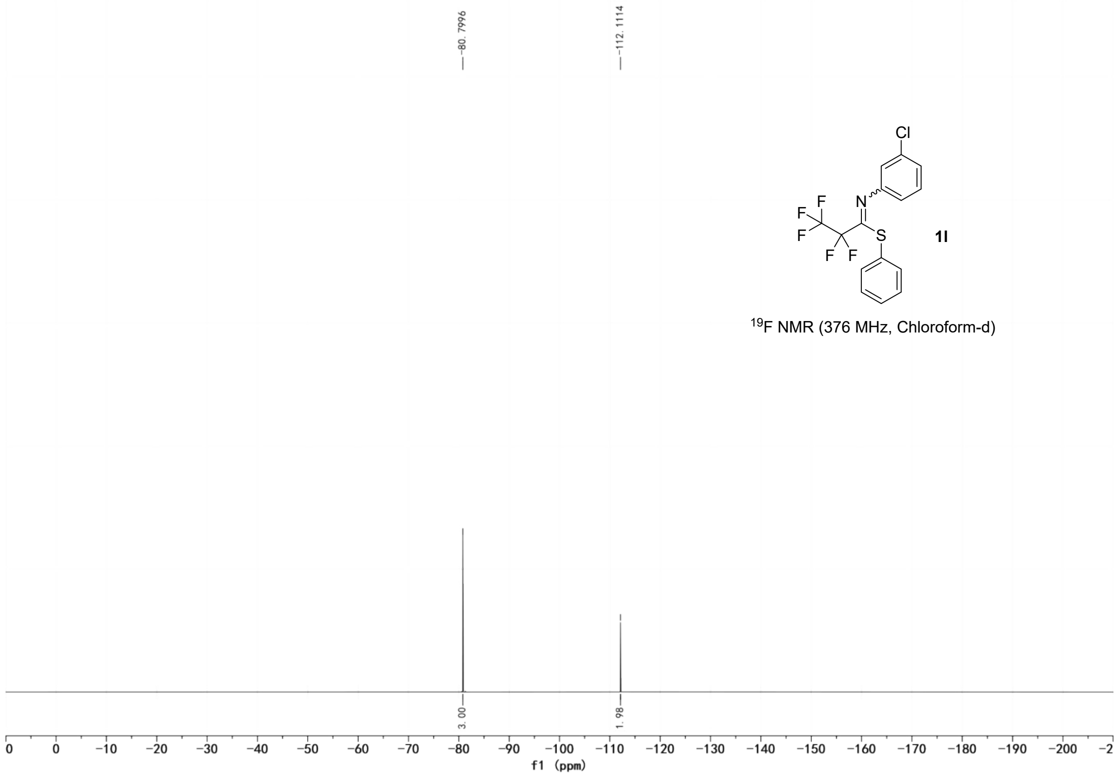
-80.7996
-112.1114
Cl
N
F
F
F
F
F
S
1l
$^{19}$F NMR (376 MHz, Chloroform-d)
3.00
1.98
f1 (ppm)

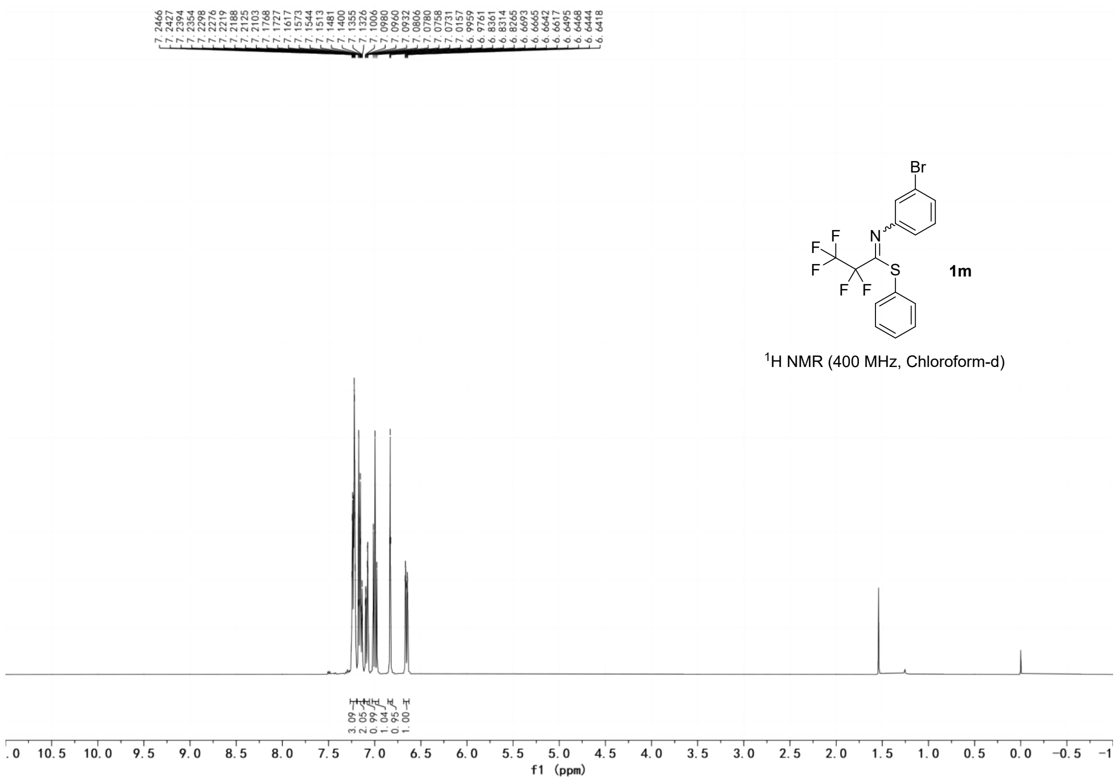
Br
1m
$^{1}$H NMR (400 MHz, Chloroform-d)
f1 (ppm)

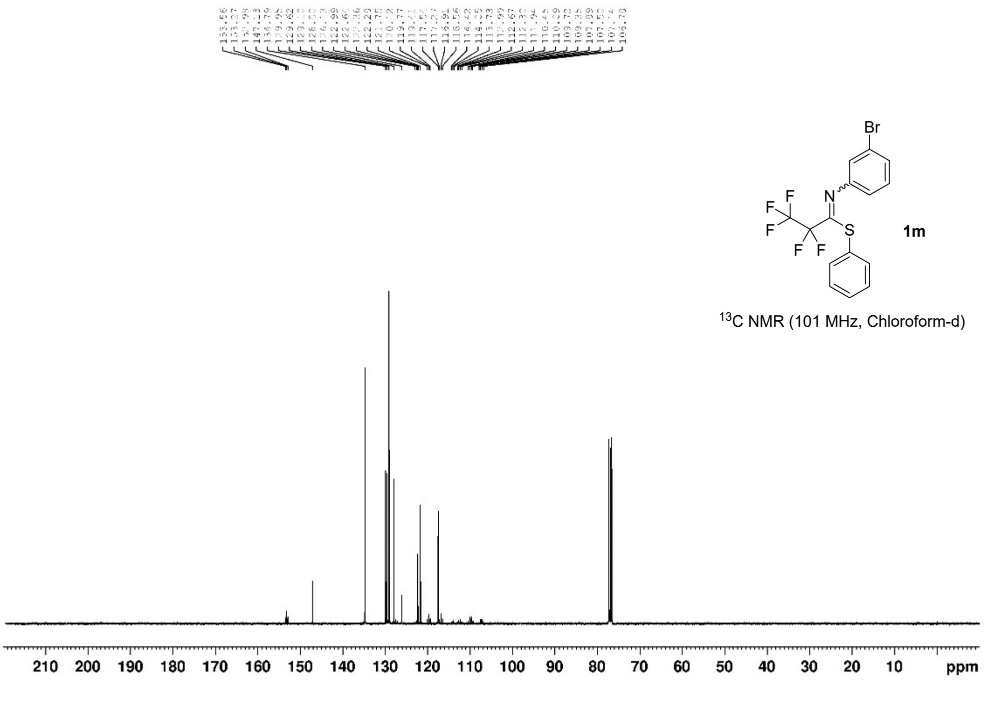
Br
1m
$^{13}$C NMR (101 MHz, Chloroform-d)
ppm

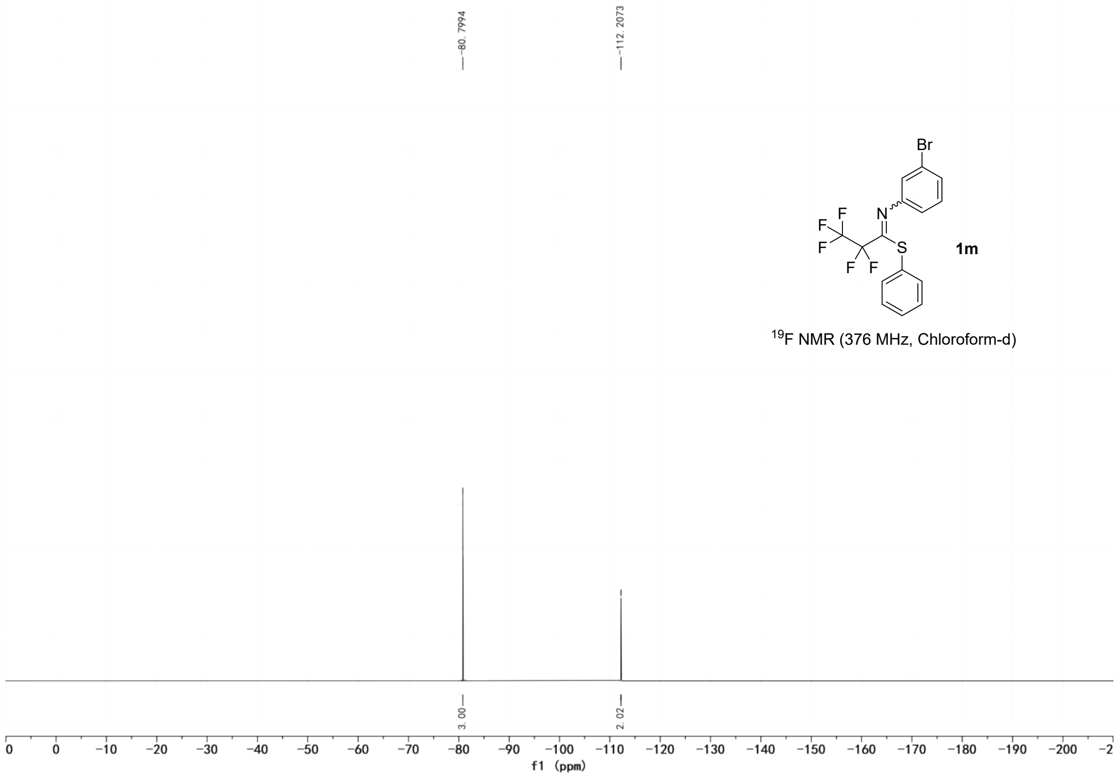

-80.7994
-112.2073
Br
N
F
F
F
F
F
S
1m
19F NMR (376 MHz, Chloroform-d)
3.00
2.02
f1 (ppm)

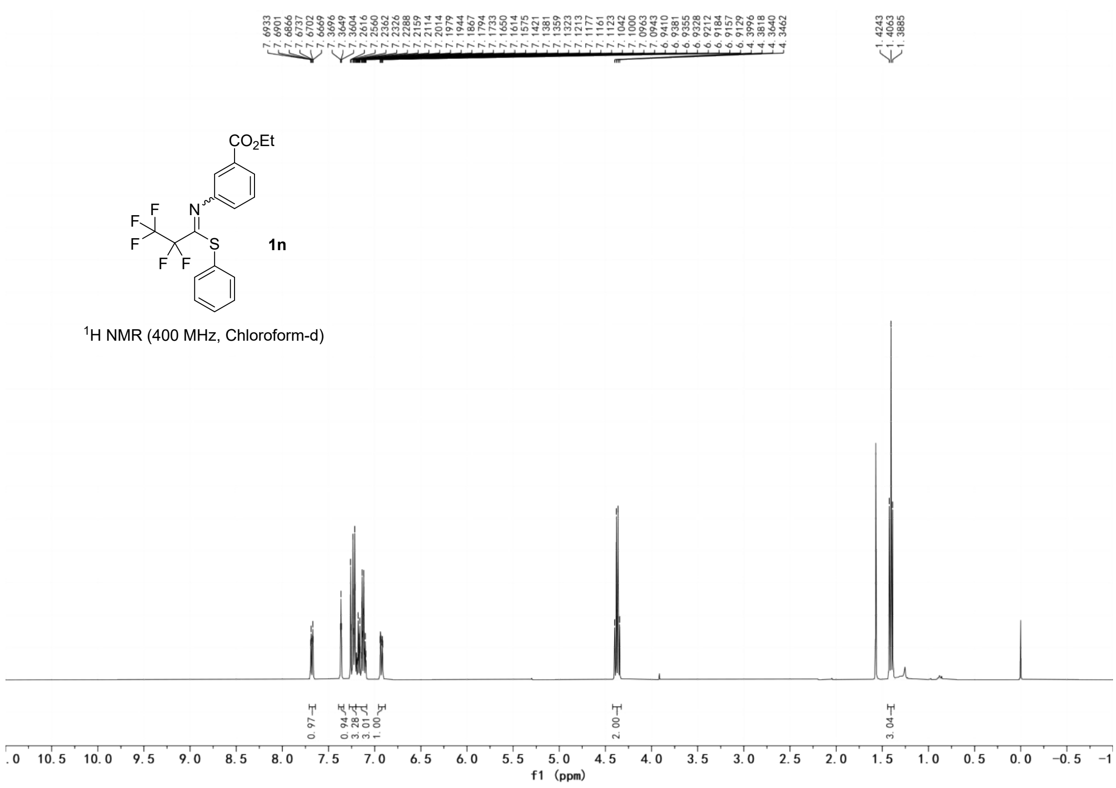
CO2Et
N
F F
F
S
F F
1n
1H NMR (400 MHz, Chloroform-d)
f1 (ppm)

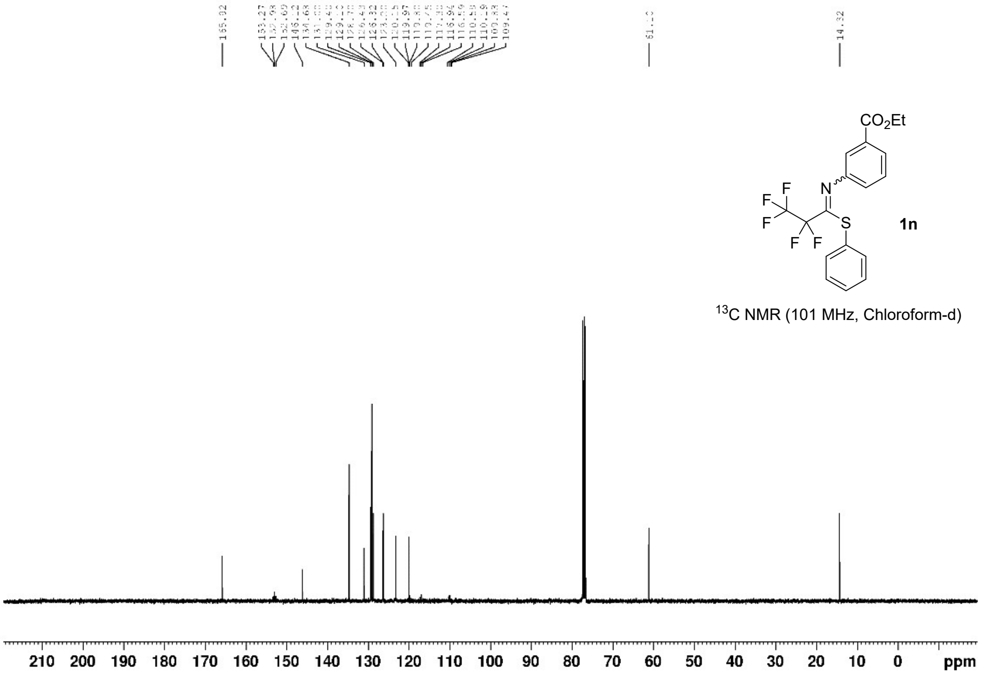
CO2Et
N
F F
F
S
F F
1n
13C NMR (101 MHz, Chloroform-d)
ppm

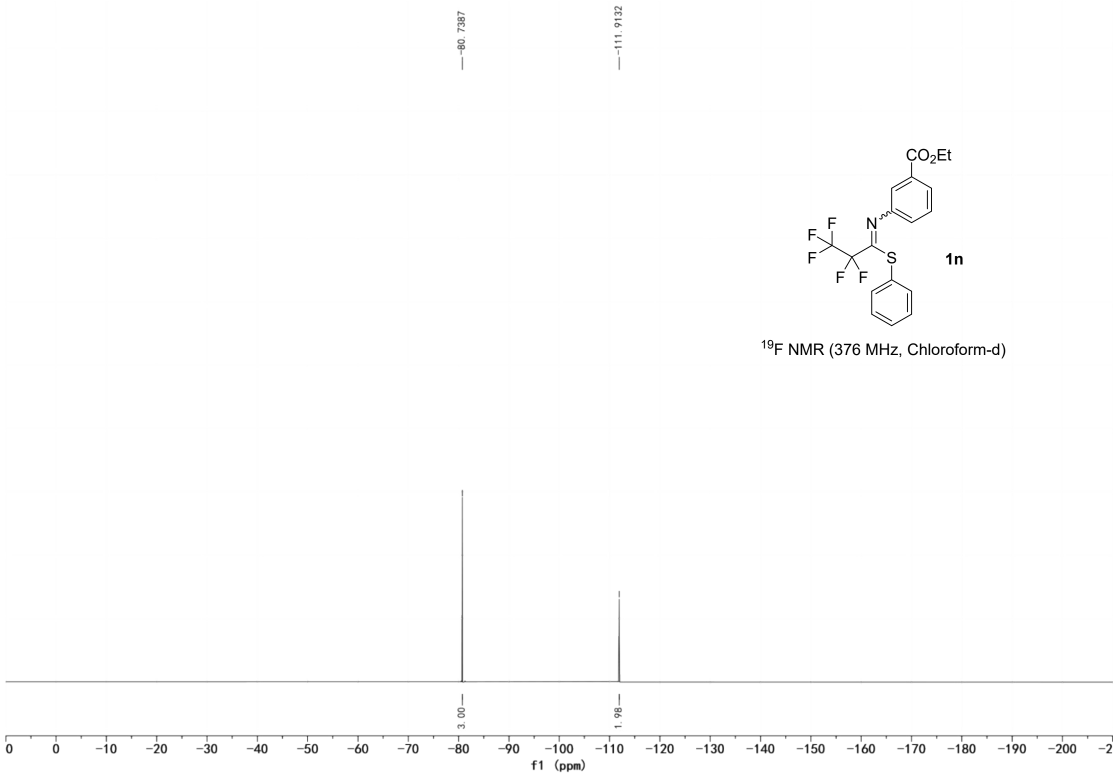

-80.7387
-111.9132
CO2Et
N
F
F
F
F
F
S
1n
19F NMR (376 MHz, Chloroform-d)
3.00
1.98
0 0 −10 −20 −30 −40 −50 −60 −70 −80 −90 −100 −110 −120 −130 −140 −150 −160 −170 −180 −190 −200 −2
f1 (ppm)

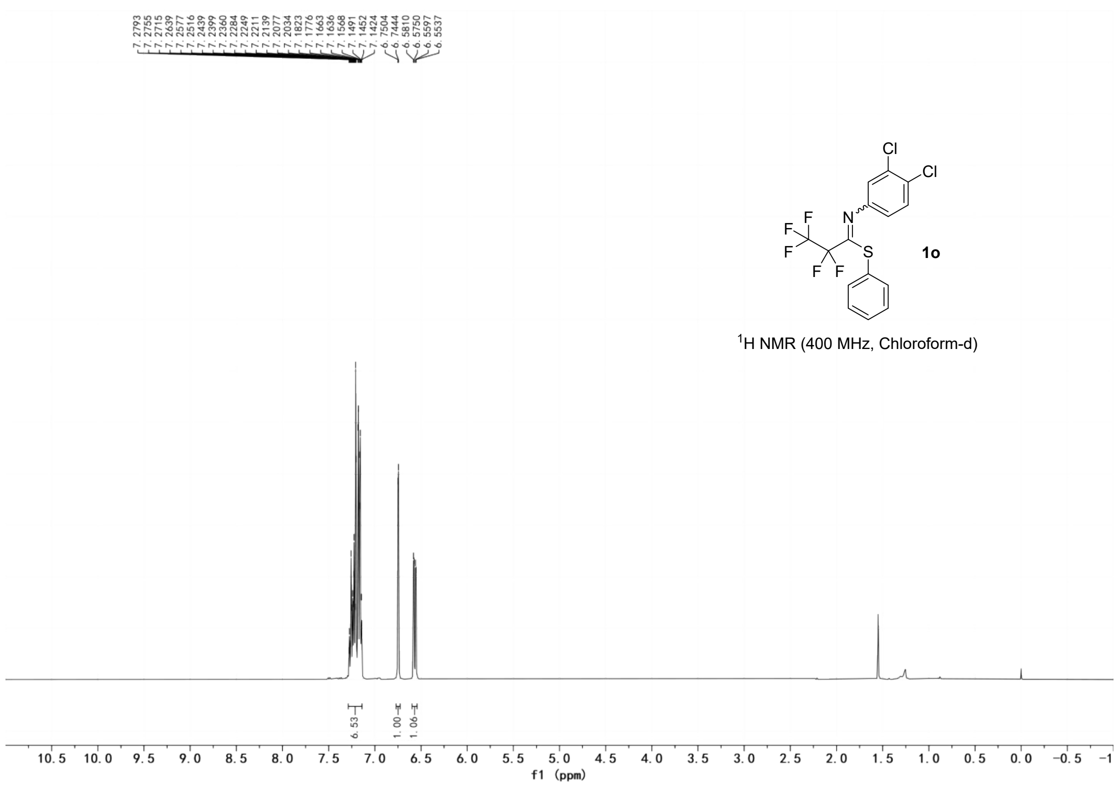
7.2793
7.2755
7.2715
7.2639
7.2577
7.2516
7.2439
7.2399
7.2360
7.2284
7.2249
7.2211
7.2139
7.2077
7.2034
7.1823
7.1776
7.1663
7.1636
7.1568
7.1491
7.1452
7.1424
6.7504
6.7444
6.5810
6.5750
6.5597
6.5537
Cl
Cl
N
F
F
F
S
F
F
1o
1H NMR (400 MHz, Chloroform-d)
6.53
1.00
1.06
10.5 10.0 9.5 9.0 8.5 8.0 7.5 7.0 6.5 6.0 5.5 5.0 4.5 4.0 3.5 3.0 2.5 2.0 1.5 1.0 0.5 0.0 -0.5 -1
f1 (ppm)

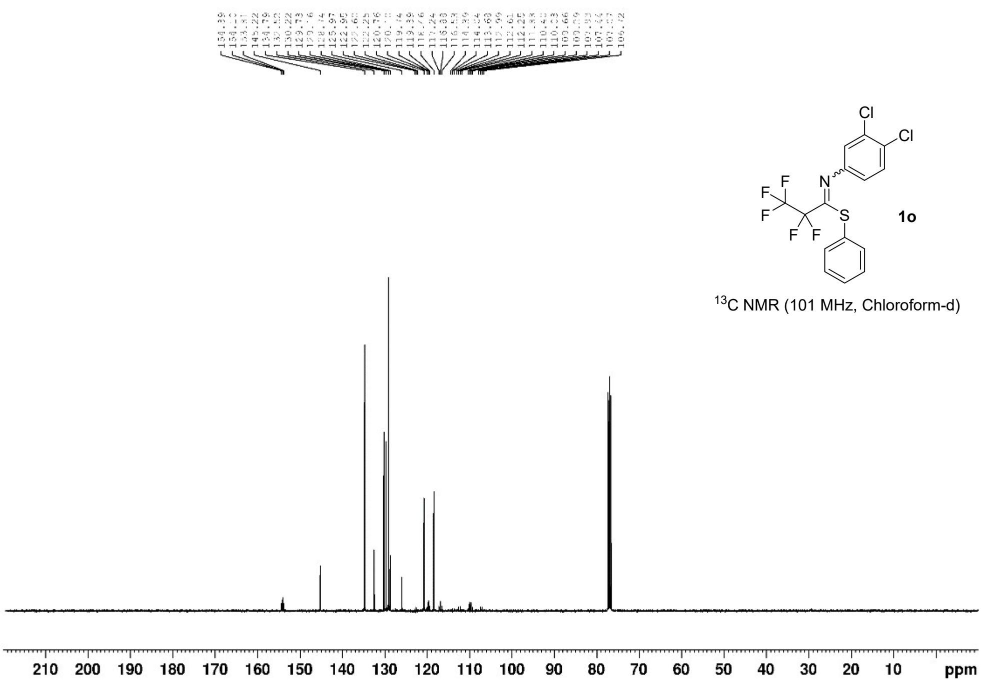
Cl
Cl
N
F
F
F
S
F
F
1o
13C NMR (101 MHz, Chloroform-d)
210 200 190 180 170 160 150 140 130 120 110 100 90 80 70 60 50 40 30 20 10 ppm

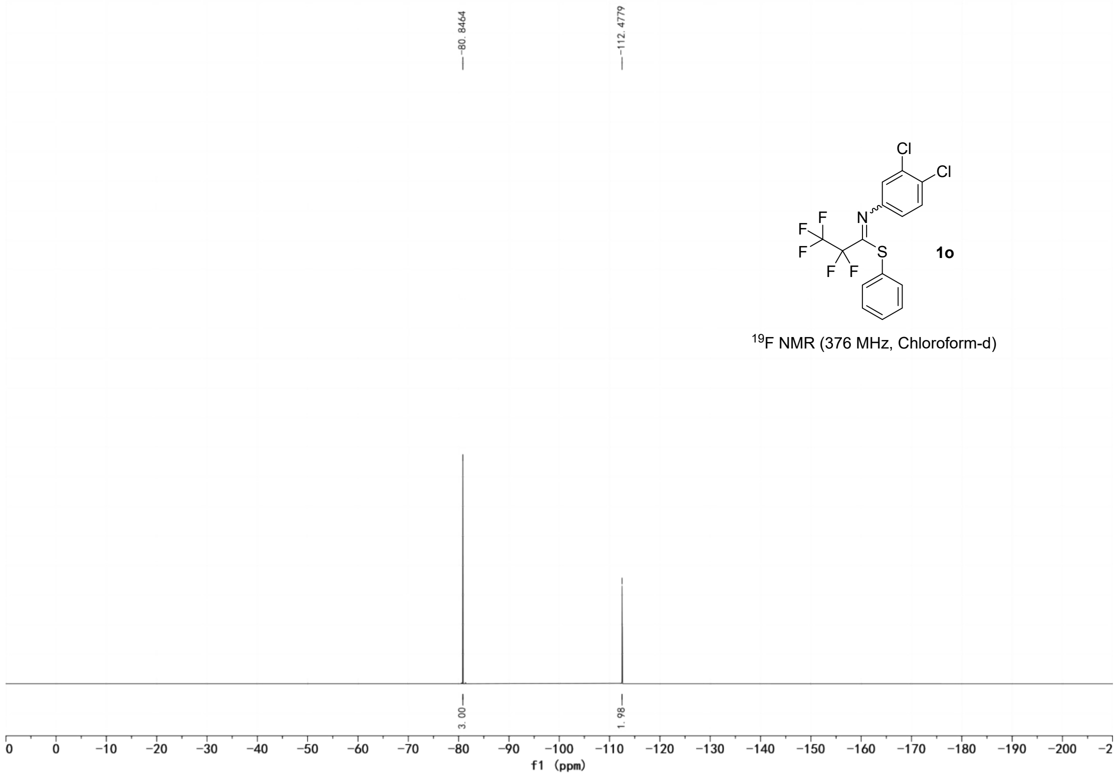
-80.8464
-112.4779
Cl
Cl
N
F
F
F
S
F
F
1o
19F NMR (376 MHz, Chloroform-d)
3.00
1.98
0
0
−10
−20
−30
−40
−50
−60
−70
−80
−90
−100
−110
−120
−130
−140
−150
−160
−170
−180
−190
−200
f1 (ppm)

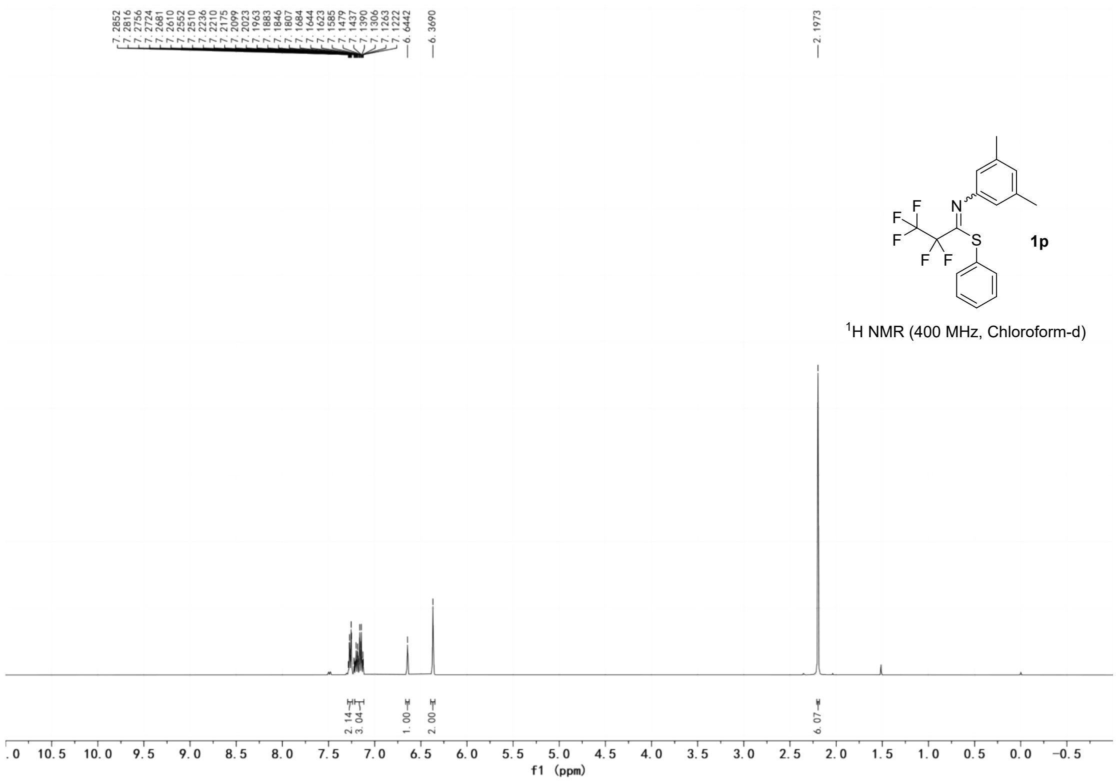

1p
$^{1}$H NMR (400 MHz, Chloroform-d)
f1 (ppm)


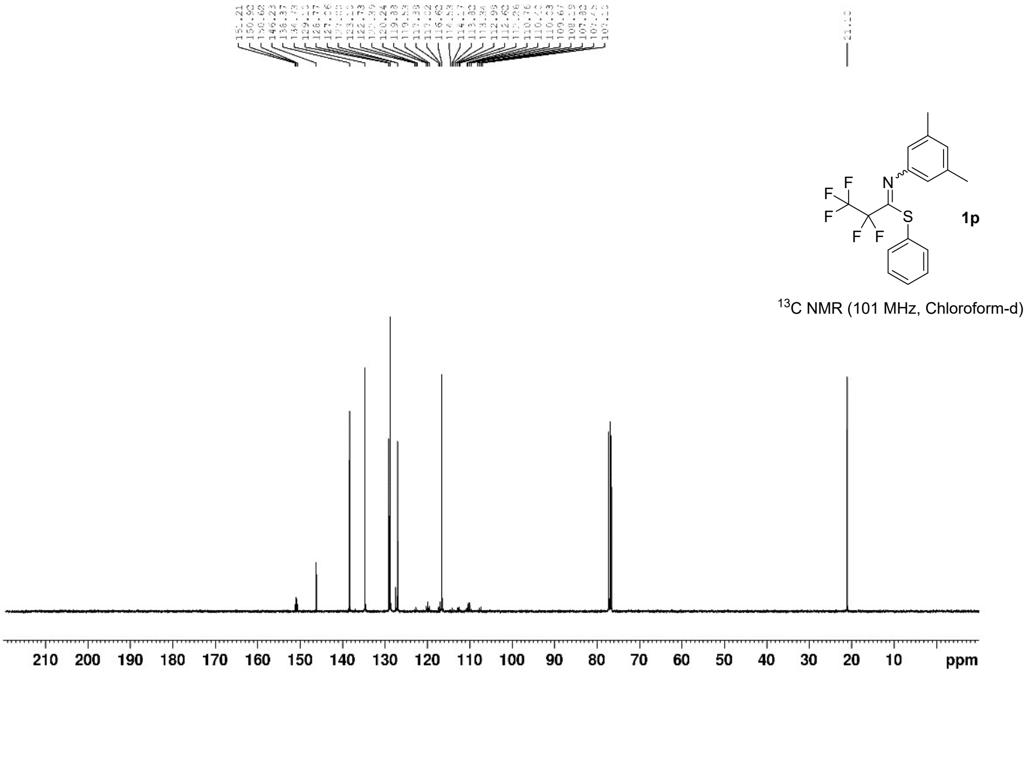

1p
$^{13}$C NMR (101 MHz, Chloroform-d)
ppm

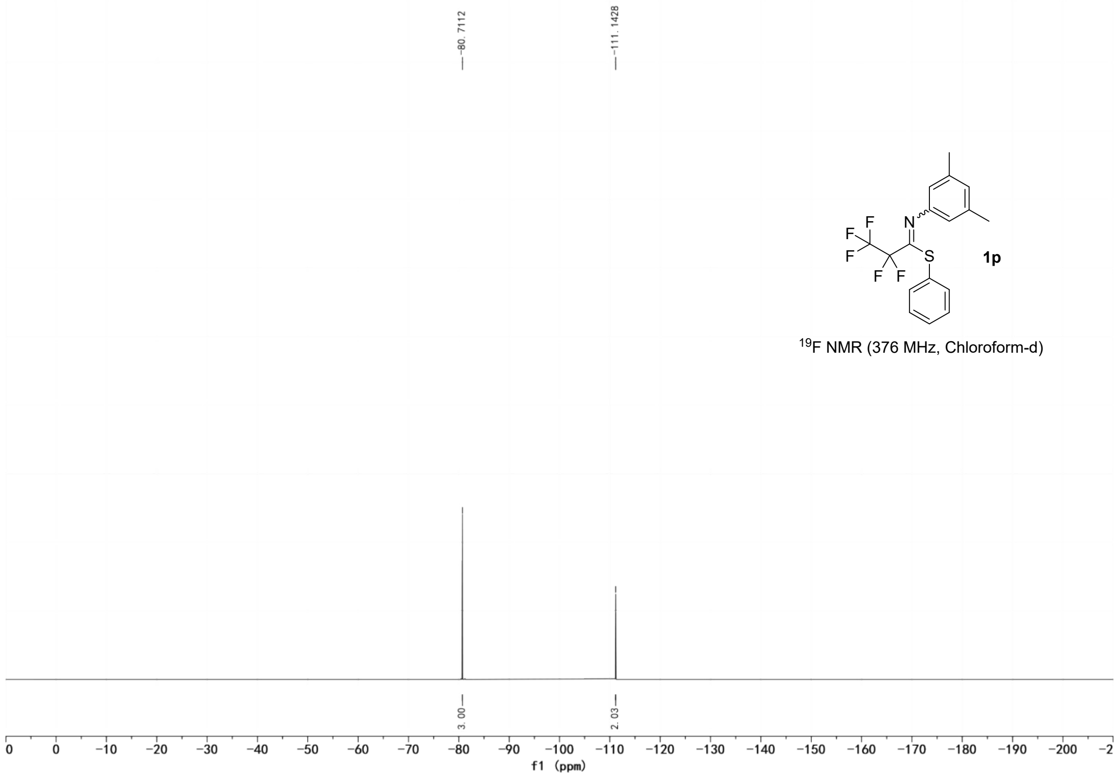
-80.7112
-111.1428
1p
$^{19}$F NMR (376 MHz, Chloroform-d)
3.00
2.03
0 −10 −20 −30 −40 −50 −60 −70 −80 −90 −100 −110 −120 −130 −140 −150 −160 −170 −180 −190 −200
f1 (ppm)

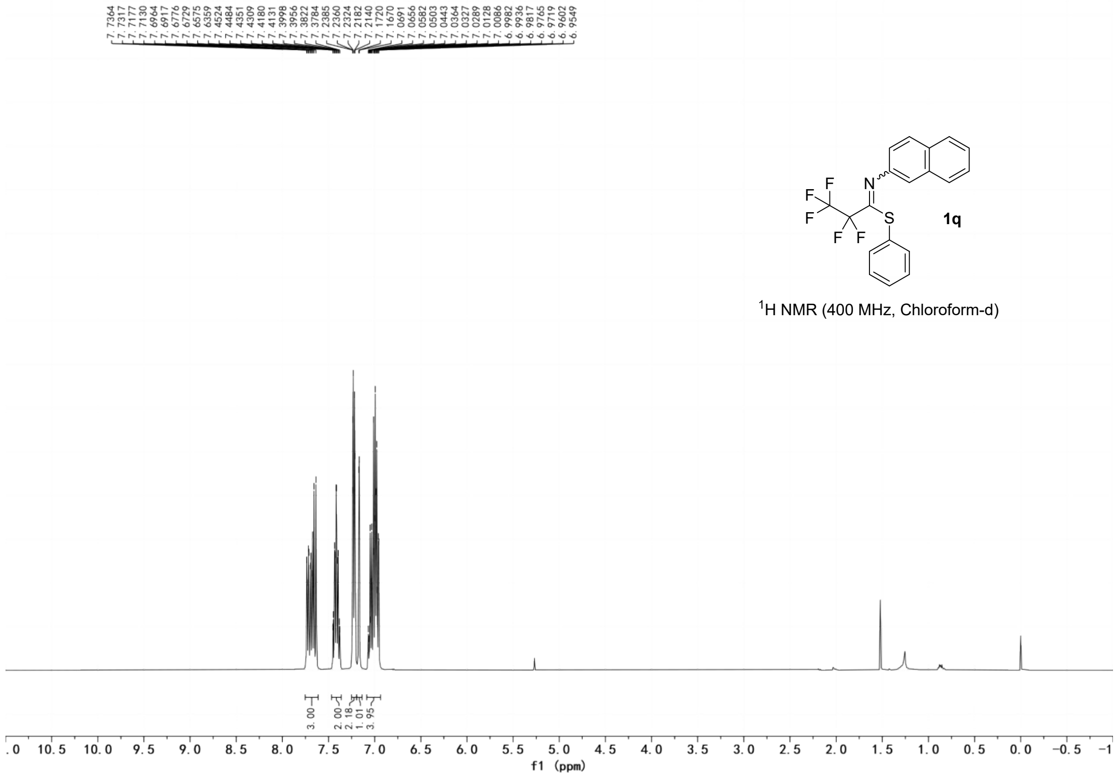
1q
$^1$H NMR (400 MHz, Chloroform-d)
f1 (ppm)

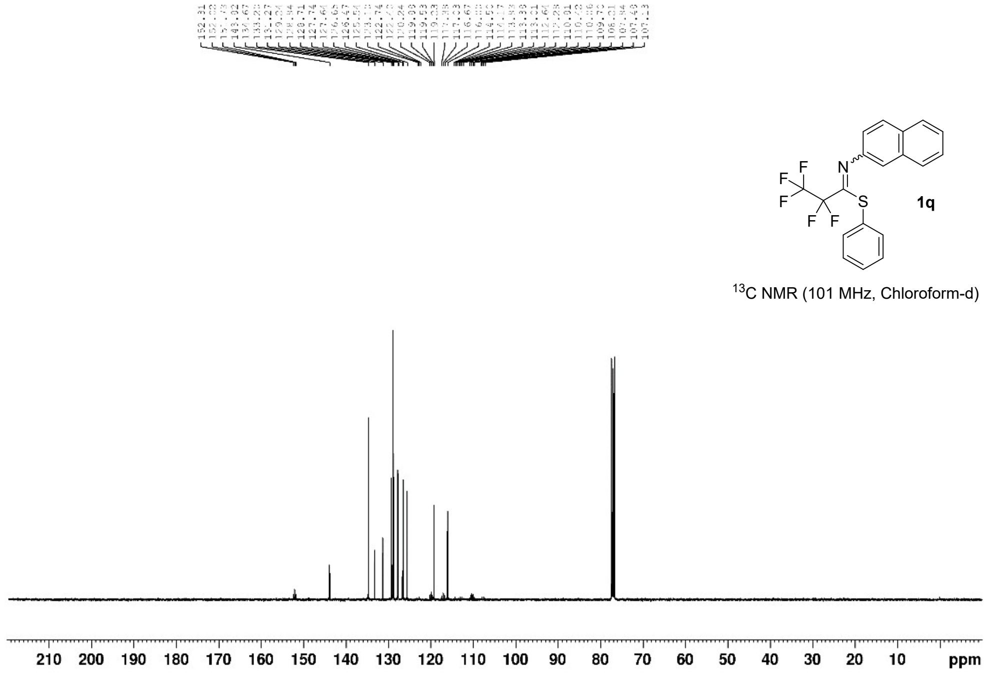
1q
$^{13}$C NMR (101 MHz, Chloroform-d)
ppm

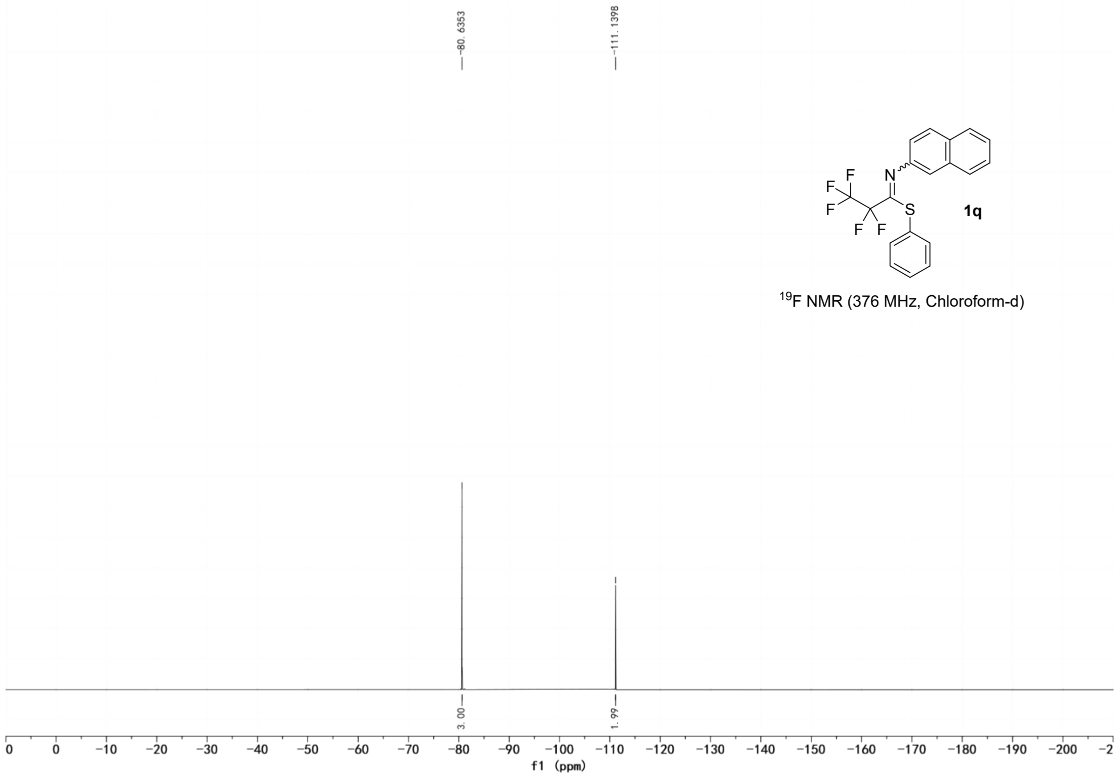

-80.6353
-111.1398
F
F
F
F
F
N
S
1q
$^{19}$F NMR (376 MHz, Chloroform-d)
3.00
1.99
0
0
−10
−20
−30
−40
−50
−60
−70
−80
−90
−100
−110
−120
−130
−140
−150
−160
−170
−180
−190
−200
−2
f1 (ppm)

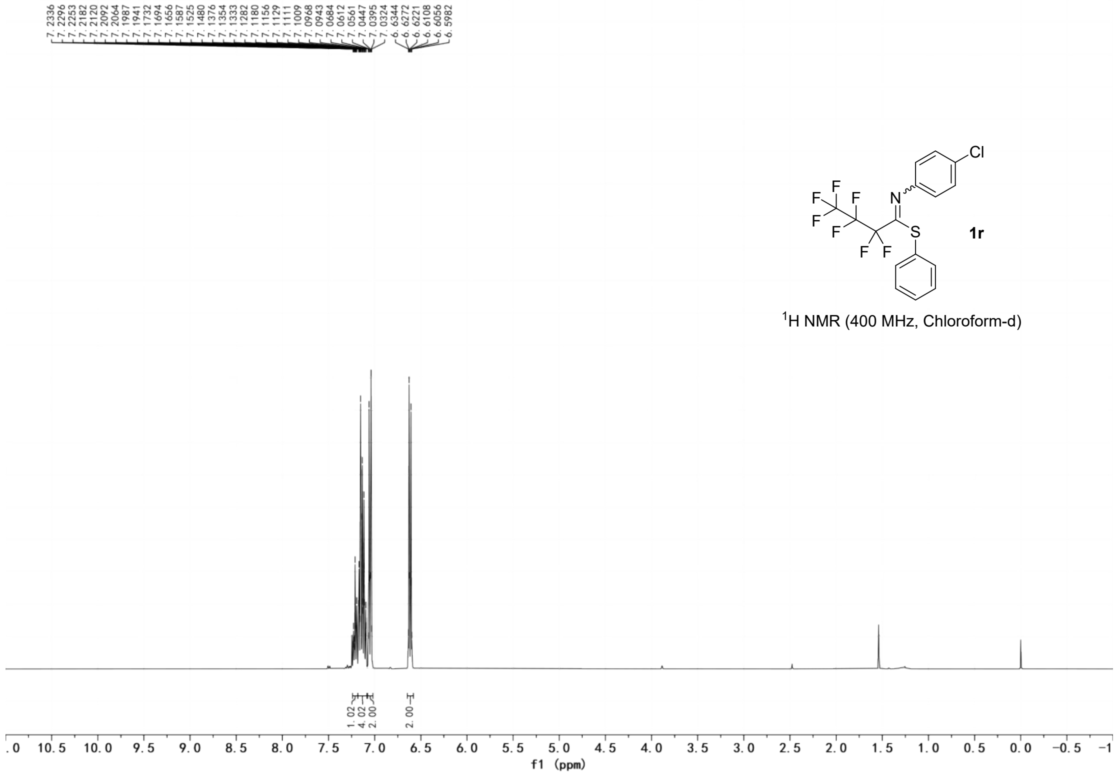
Cl
N
S
1r
1H NMR (400 MHz, Chloroform-d)
f1 (ppm)

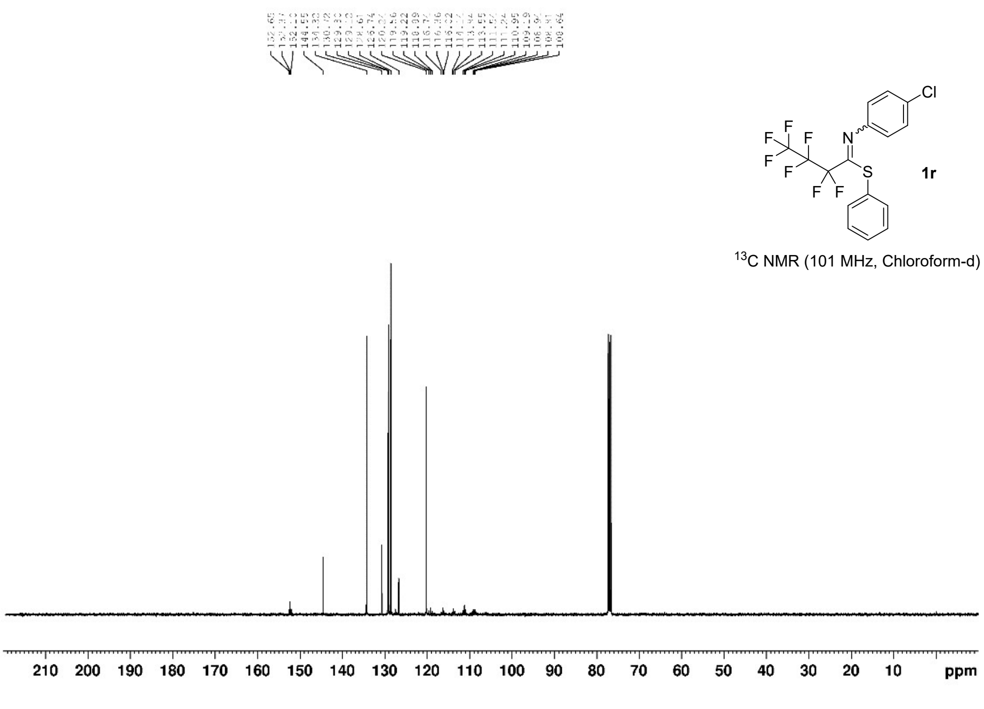
Cl
N
S
1r
13C NMR (101 MHz, Chloroform-d)
ppm

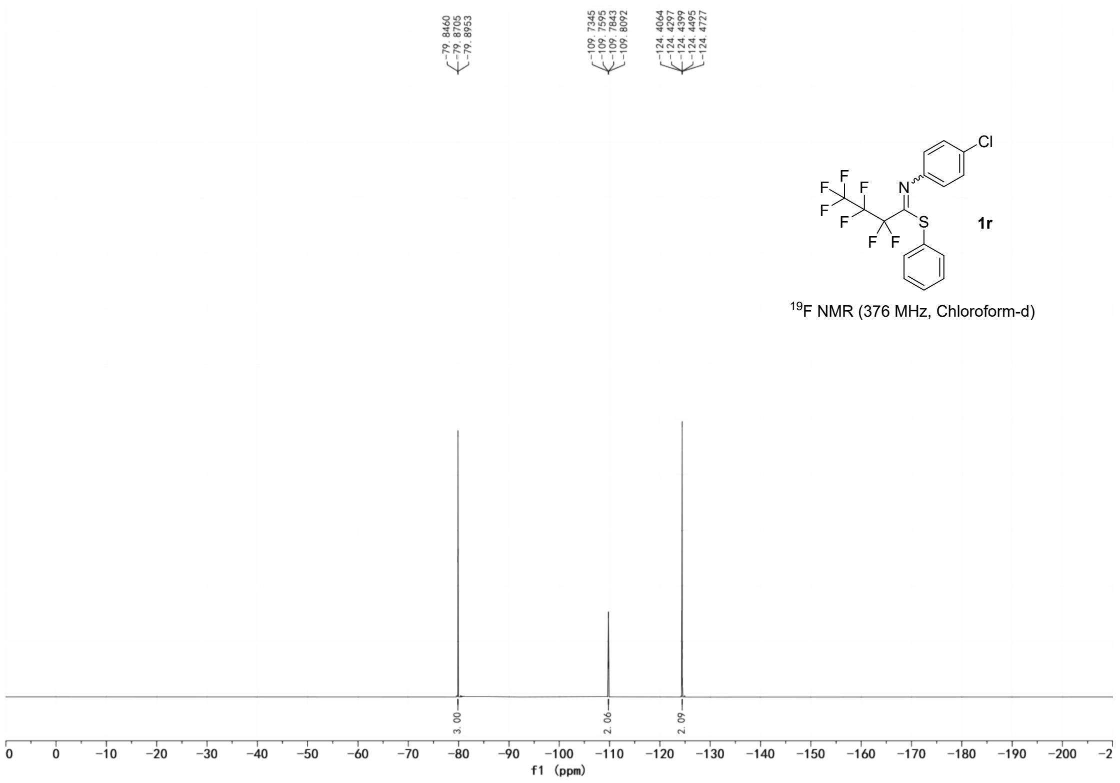
-79.8460
-79.8705
-79.8953
-109.7345
-109.7595
-109.7843
-109.8092
-124.4064
-124.4297
-124.4399
-124.4495
-124.4727
Cl
N
F
S
1r
$^{19}$F NMR (376 MHz, Chloroform-d)
3.00
2.06
2.09
f1 (ppm)

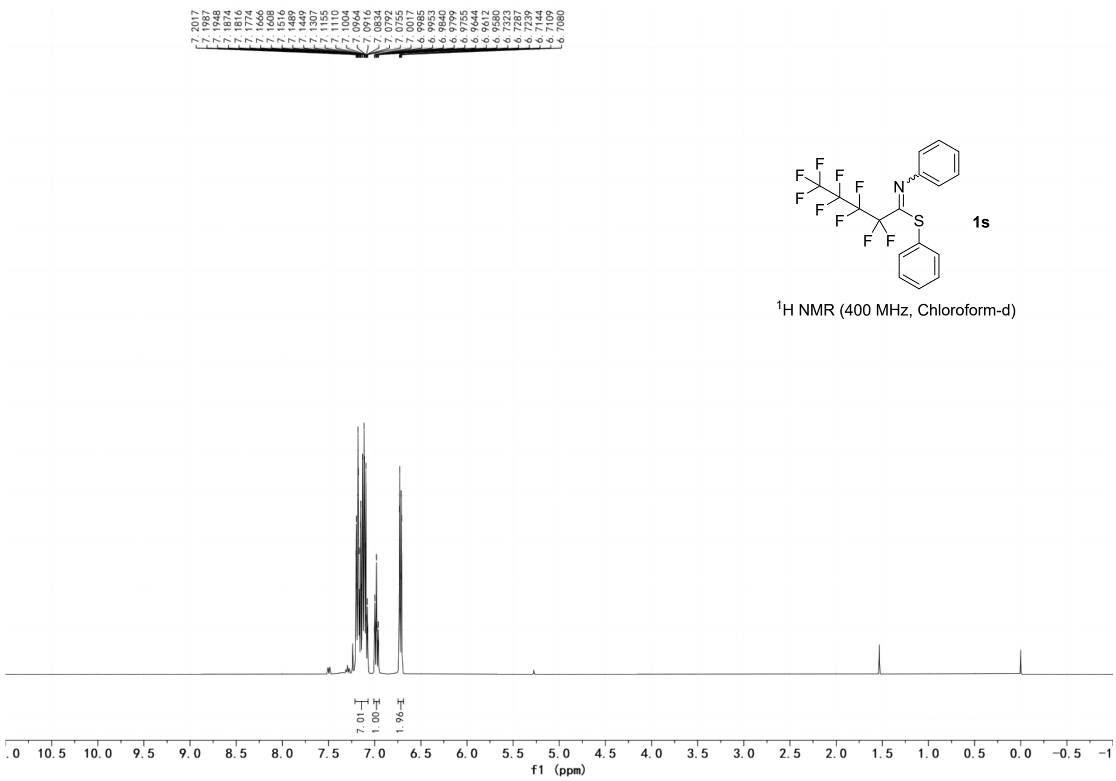
7.2017
7.1987
7.1948
7.1874
7.1816
7.1774
7.1666
7.1608
7.1516
7.1489
7.1449
7.1307
7.1155
7.1110
7.1004
7.0964
7.0916
7.0834
7.0792
7.0755
7.0017
6.9985
6.9953
6.9840
6.9799
6.9755
6.9644
6.9612
6.9580
6.7323
6.7287
6.7239
6.7144
6.7109
6.7080
1s
1H NMR (400 MHz, Chloroform-d)
7.01
1.00
1.96
f1 (ppm)

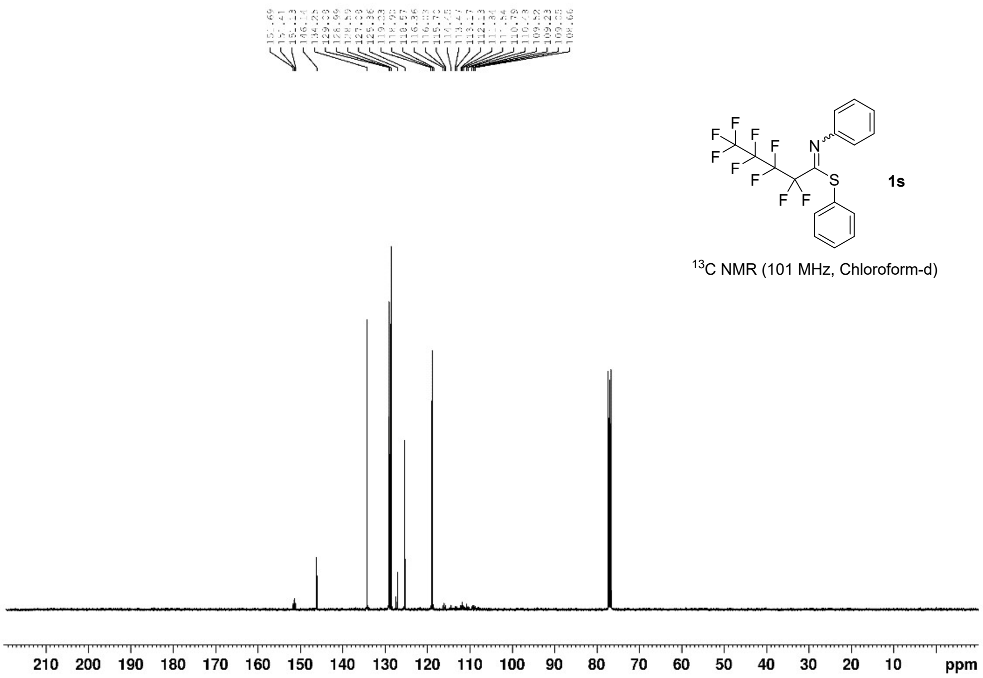
1s
13C NMR (101 MHz, Chloroform-d)
ppm

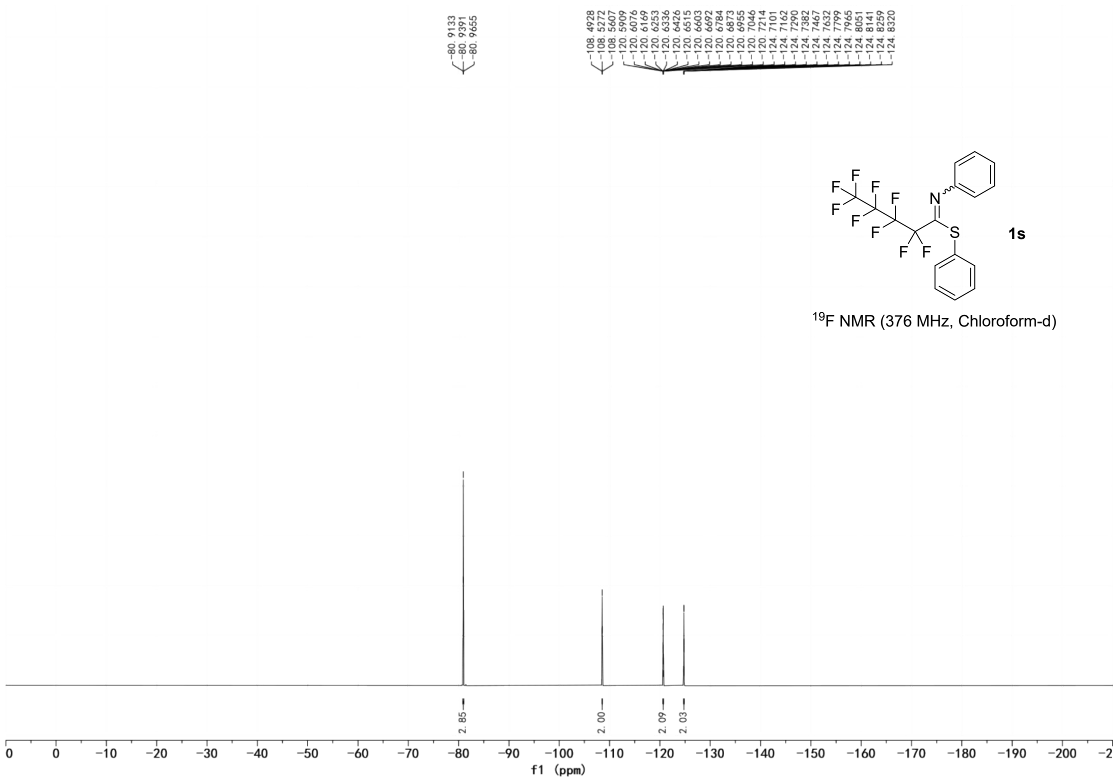
80.9133
80.9391
80.9655
108.4928
108.5272
108.5607
120.5909
120.6076
120.6169
120.6253
120.6336
120.6426
120.6515
120.6603
120.6692
120.6784
120.6873
120.6955
120.7046
120.7214
124.7101
124.7162
124.7290
124.7382
124.7467
124.7632
124.7799
124.7965
124.8051
124.8141
124.8259
124.8320
1s
19F NMR (376 MHz, Chloroform-d)
2.85
2.00
2.09
2.03
f1 (ppm)

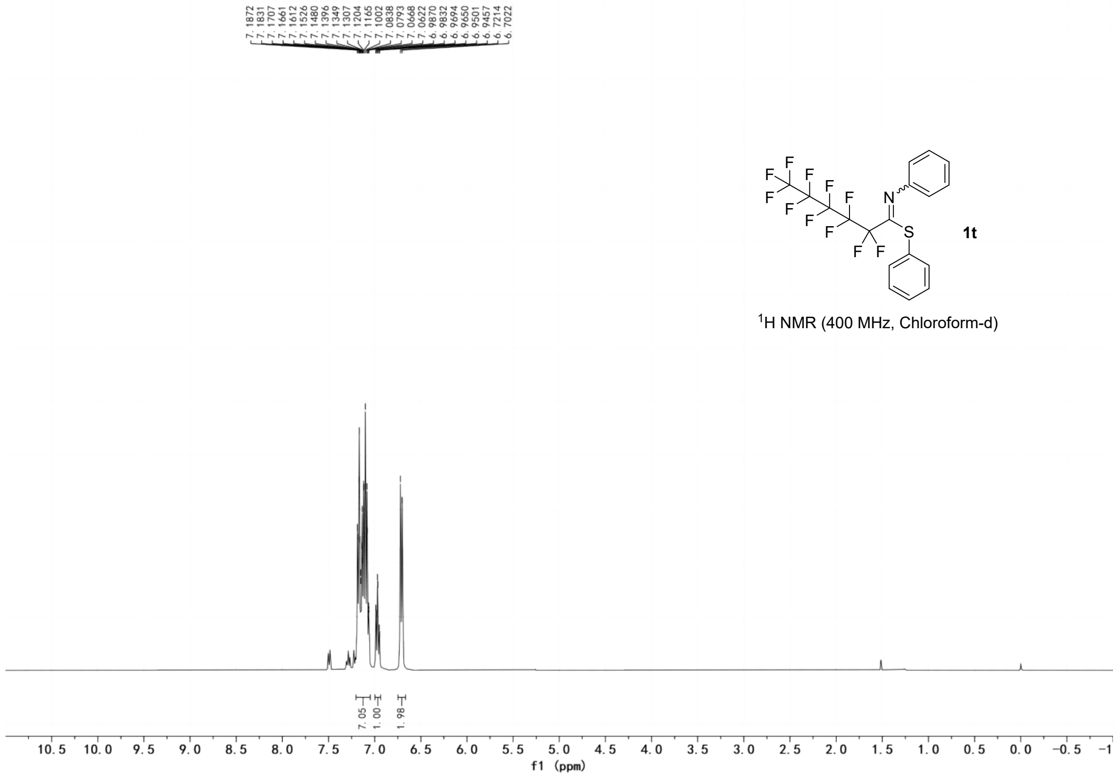
1t
$^1$H NMR (400 MHz, Chloroform-d)
f1 (ppm)

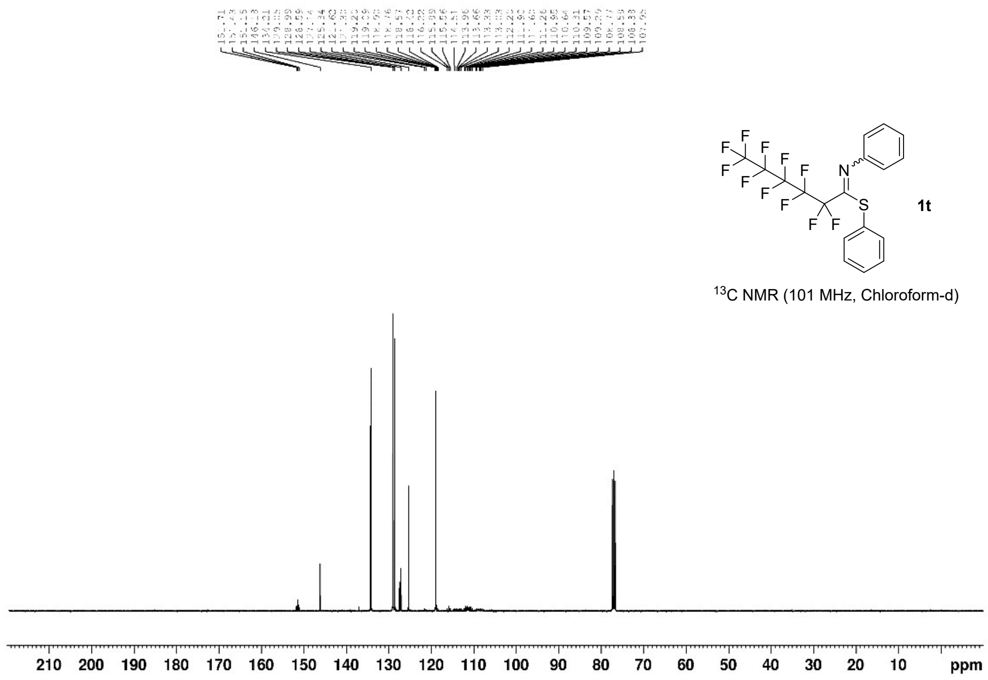
1t
$^{13}$C NMR (101 MHz, Chloroform-d)
ppm

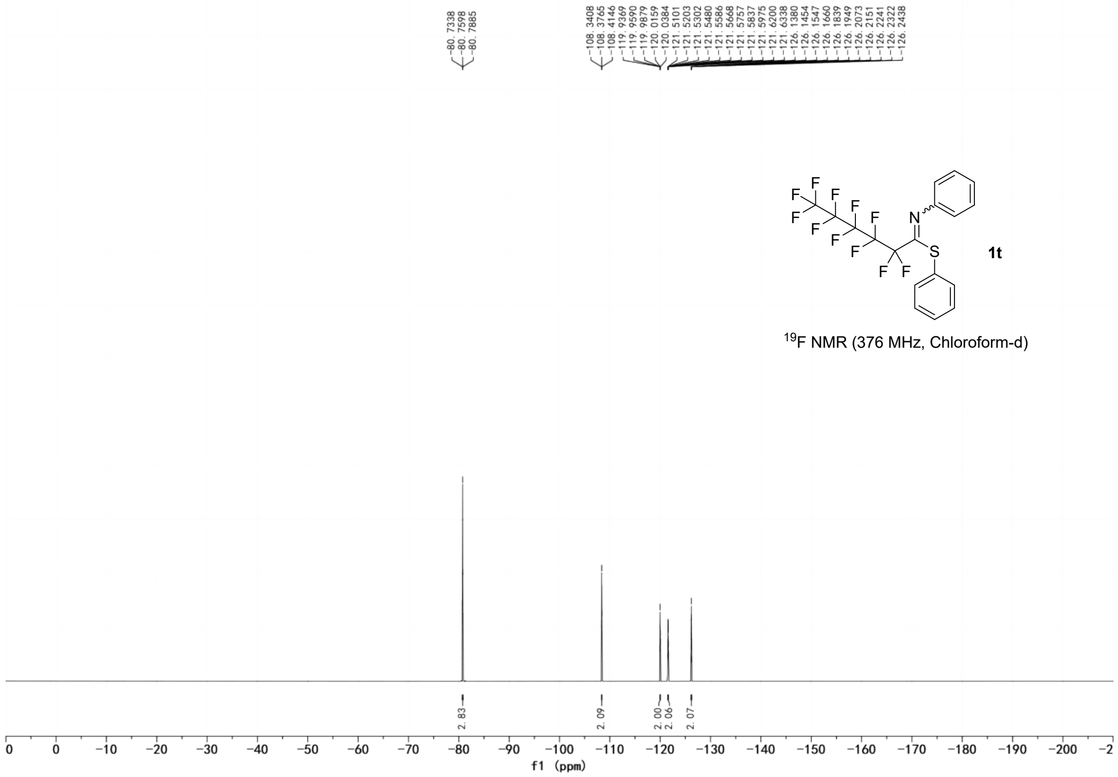

1t
19F NMR (376 MHz, Chloroform-d)
f1 (ppm)

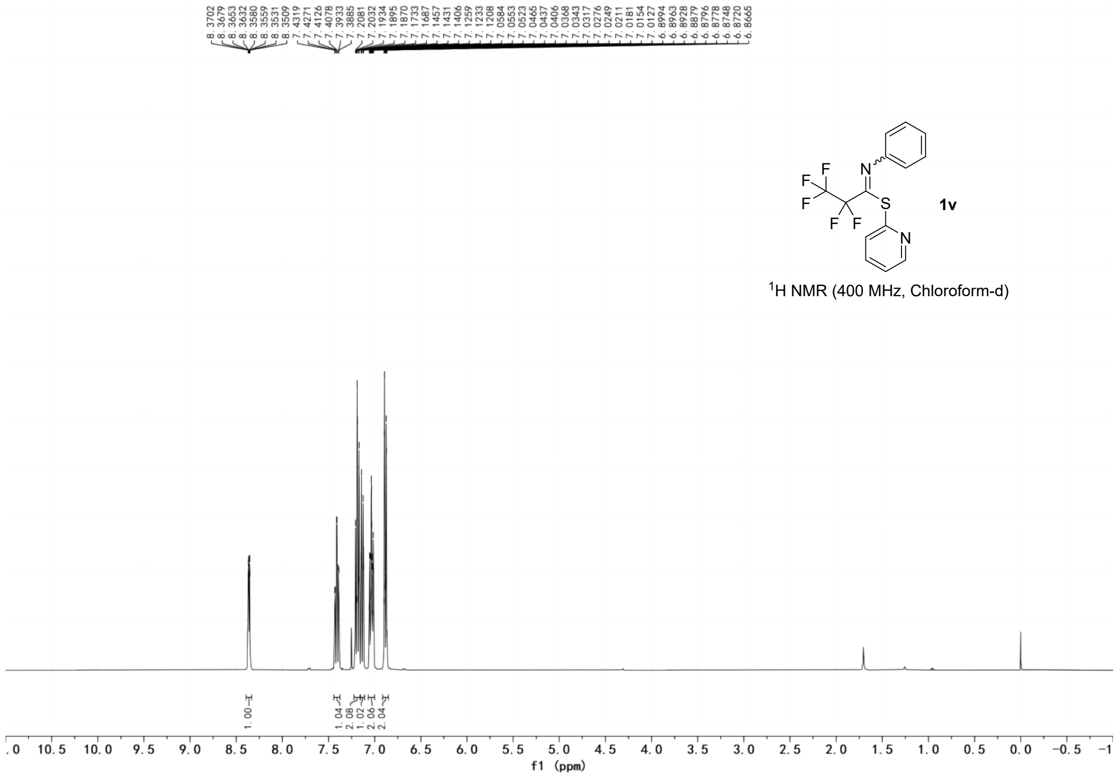
8.3702
8.3679
8.3653
8.3632
8.3580
8.3559
8.3531
8.3509
7.4319
7.4271
7.4126
7.4078
7.3933
7.3885
7.2081
7.2032
7.1934
7.1895
7.1870
7.1733
7.1687
7.1457
7.1431
7.1406
7.1259
7.1233
7.1208
7.0584
7.0553
7.0523
7.0465
7.0437
7.0406
7.0368
7.0343
7.0317
7.0276
7.0249
7.0211
7.0181
7.0154
7.0127
6.8994
6.8963
6.8928
6.8879
6.8796
6.8778
6.8748
6.8720
6.8665
1v
¹H NMR (400 MHz, Chloroform-d)
1.00
1.04
2.08
1.02
2.06
2.04
f1 (ppm)

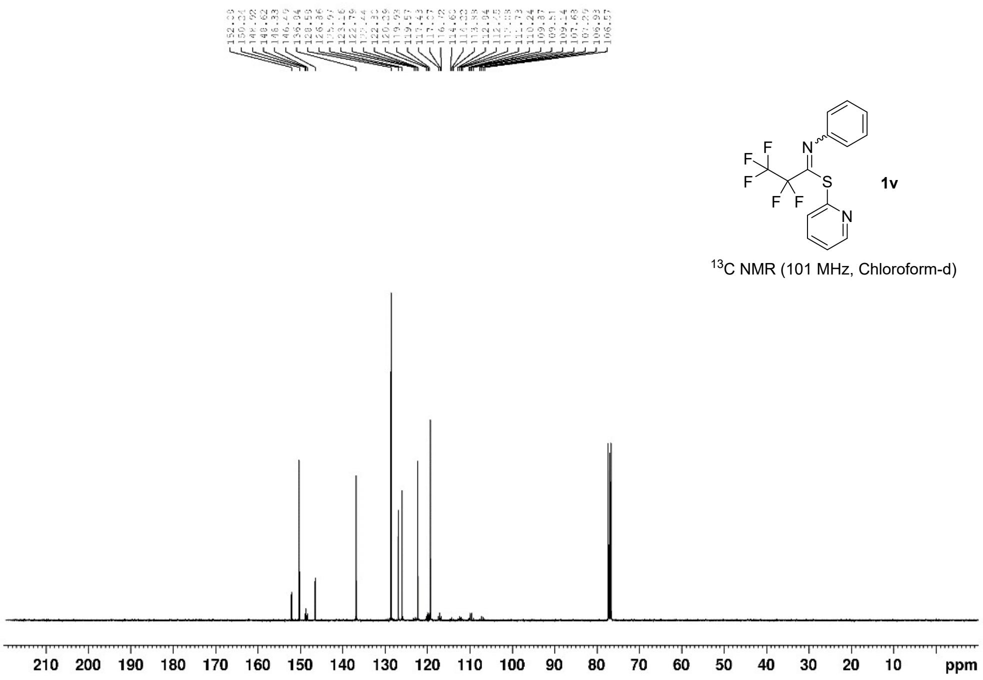
1v
¹³C NMR (101 MHz, Chloroform-d)
ppm

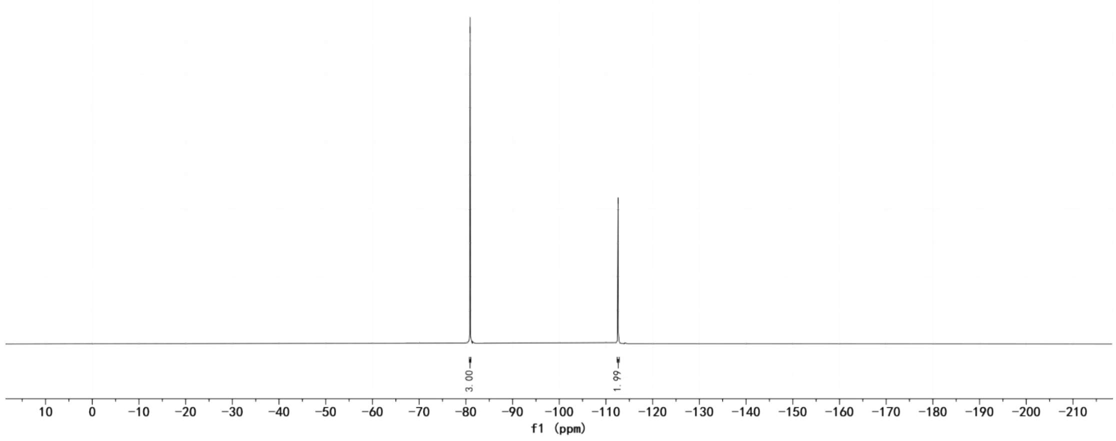


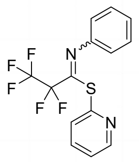


**1v**

$^{19}$F NMR (376 MHz, Chloroform-d)

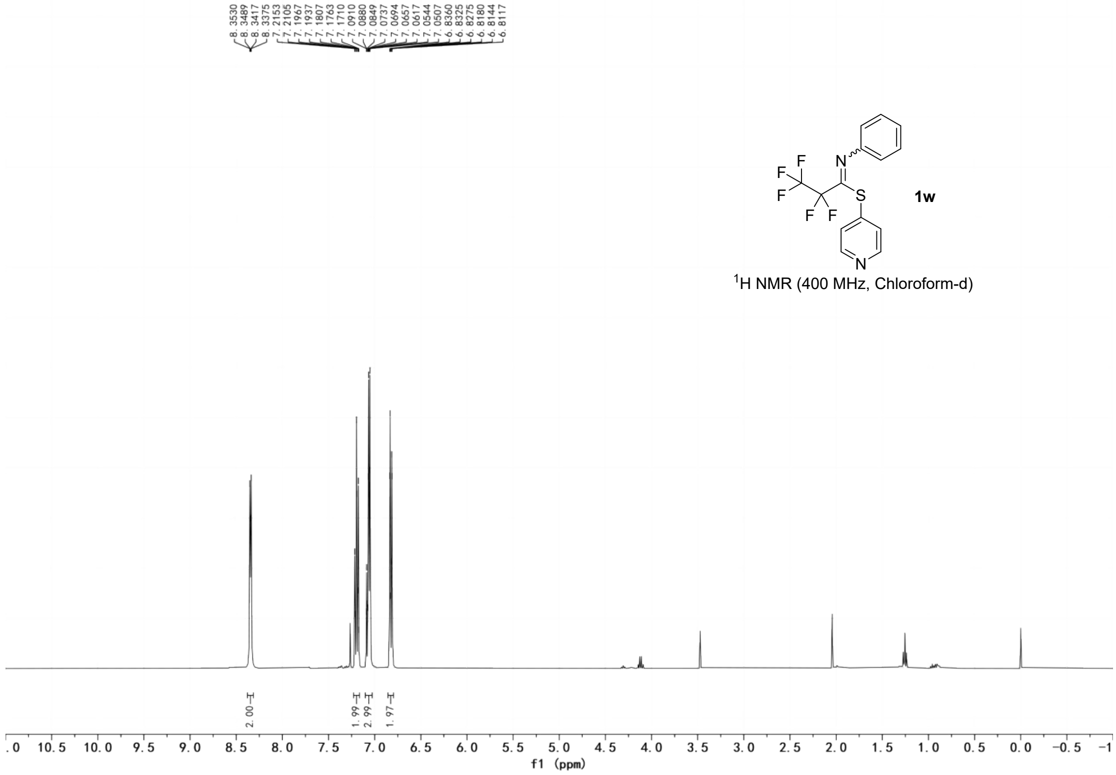
8.3530
8.3489
8.3417
8.3375
7.2153
7.2105
7.1967
7.1937
7.1807
7.1763
7.1710
7.0910
7.0880
7.0849
7.0737
7.0694
7.0657
7.0617
7.0544
7.0507
6.8360
6.8325
6.8275
6.8180
6.8144
6.8117
1w
1H NMR (400 MHz, Chloroform-d)
2.00
1.99
2.99
1.97
f1 (ppm)

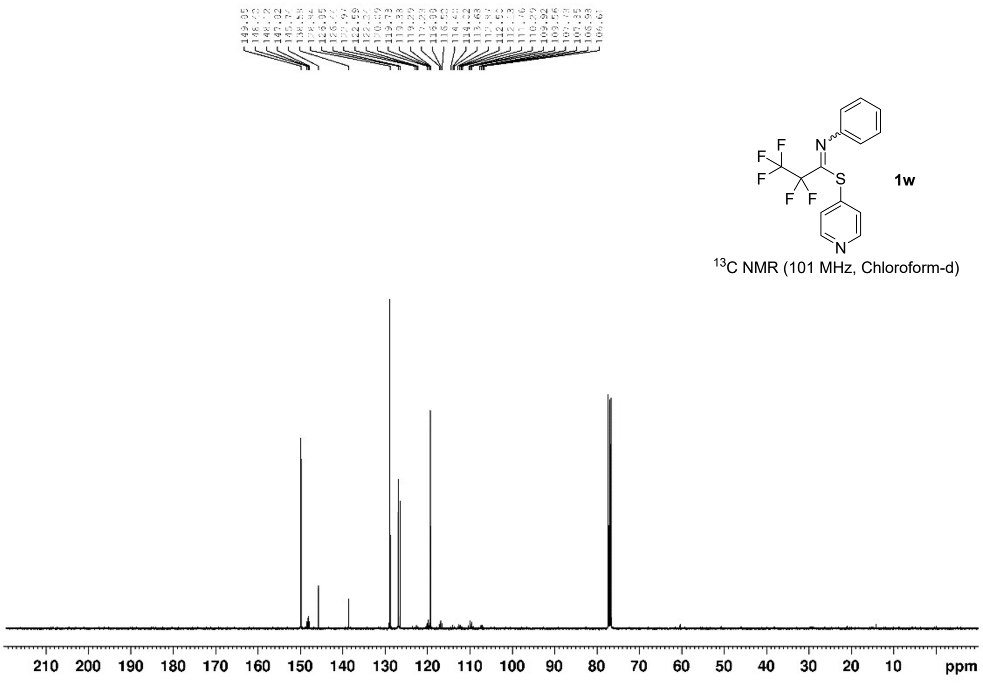
1w
13C NMR (101 MHz, Chloroform-d)
ppm

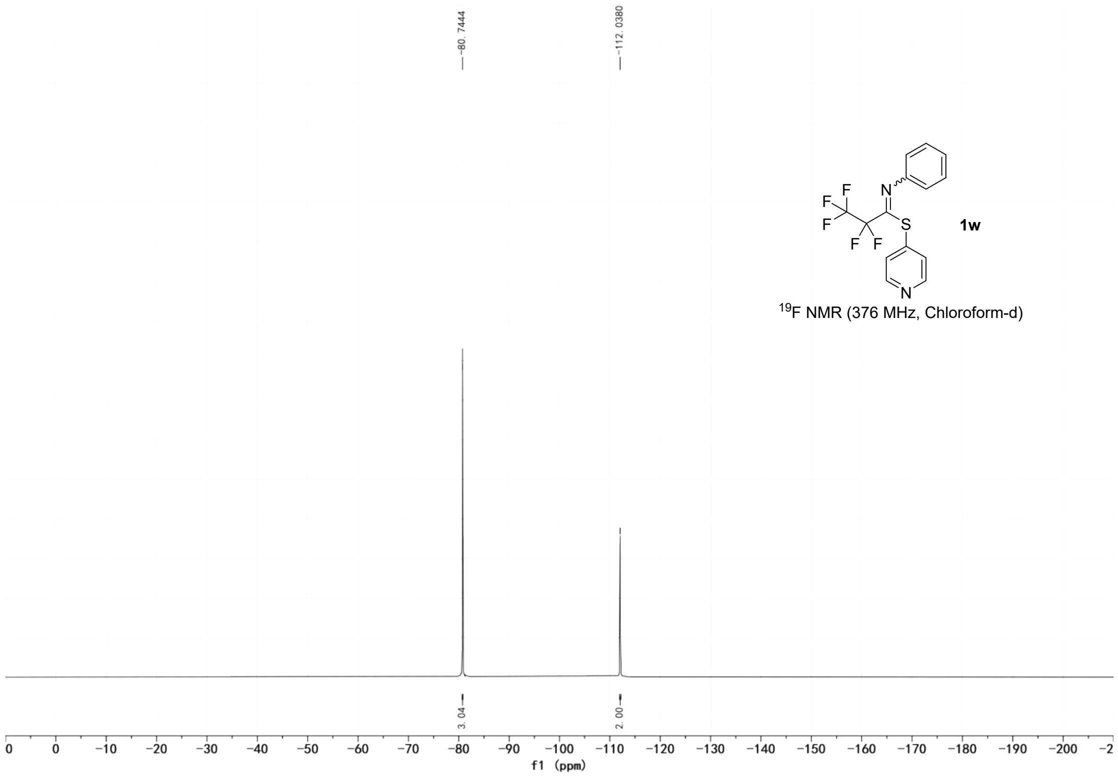
-80.7444
-112.0380
1w
$^{19}$F NMR (376 MHz, Chloroform-d)
3.04
2.00
f1 (ppm)

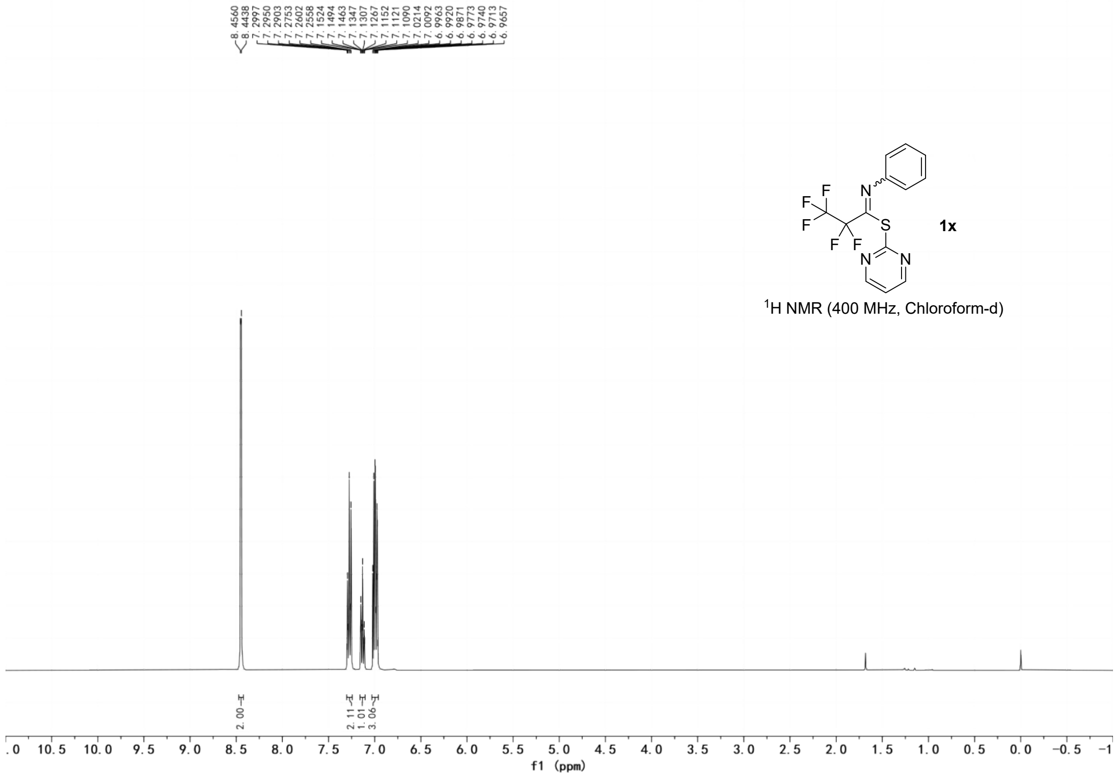
1x
1H NMR (400 MHz, Chloroform-d)

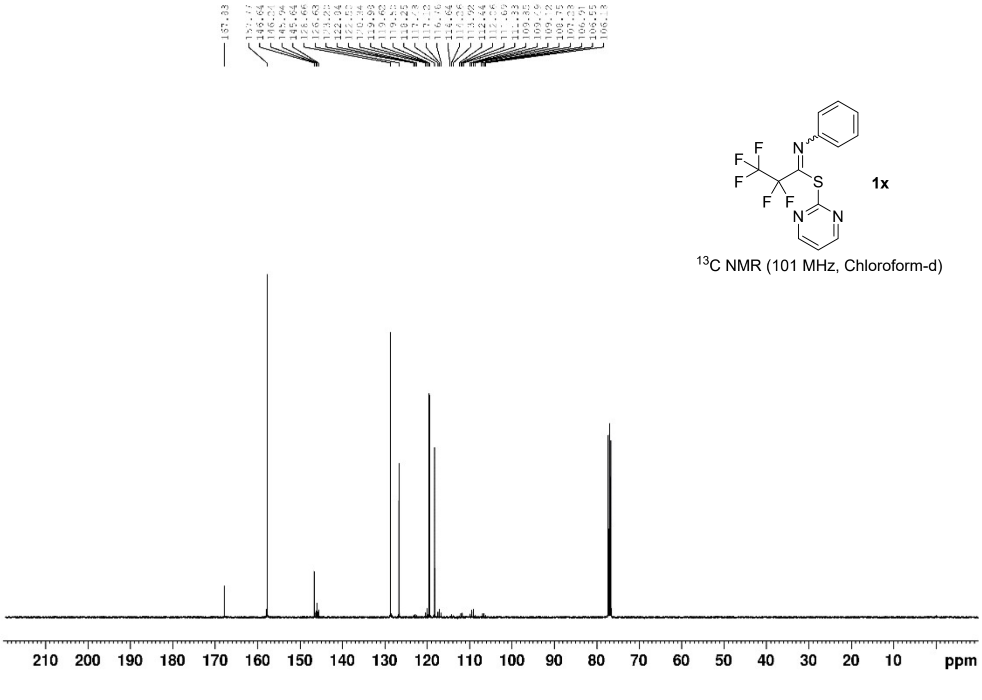
1x
13C NMR (101 MHz, Chloroform-d)

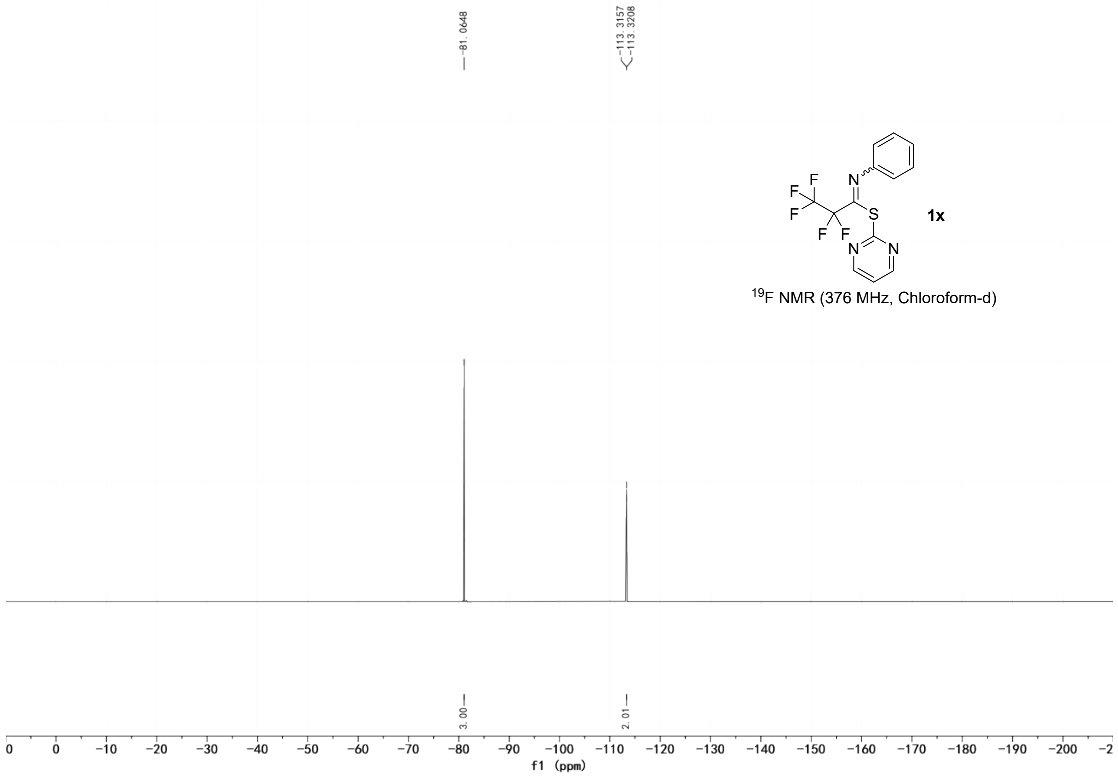

—81.0648
-113.3157
-113.3208
F
F
F
F
F
N
S
N
N
1x
19F NMR (376 MHz, Chloroform-d)
3.00
2.01
0
0
−10
−20
−30
−40
−50
−60
−70
−80
−90
−100
−110
−120
−130
−140
−150
−160
−170
−180
−190
−200
f1 (ppm)

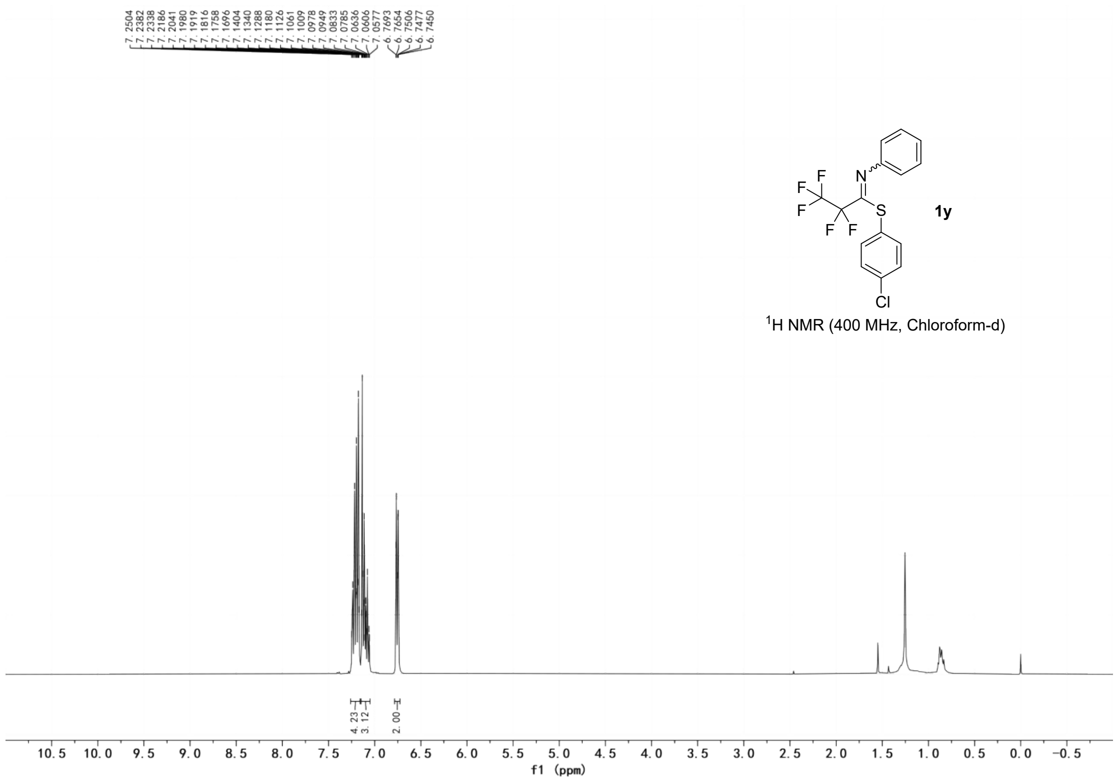


1y

$^{1}H$ NMR (400 MHz, Chloroform-d)

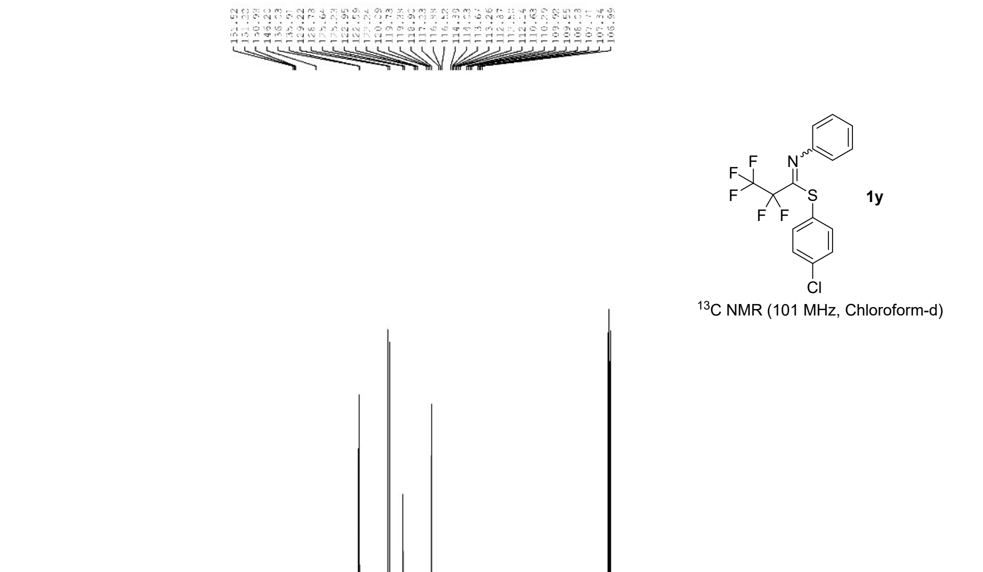


1y

$^{13}C$ NMR (101 MHz, Chloroform-d)

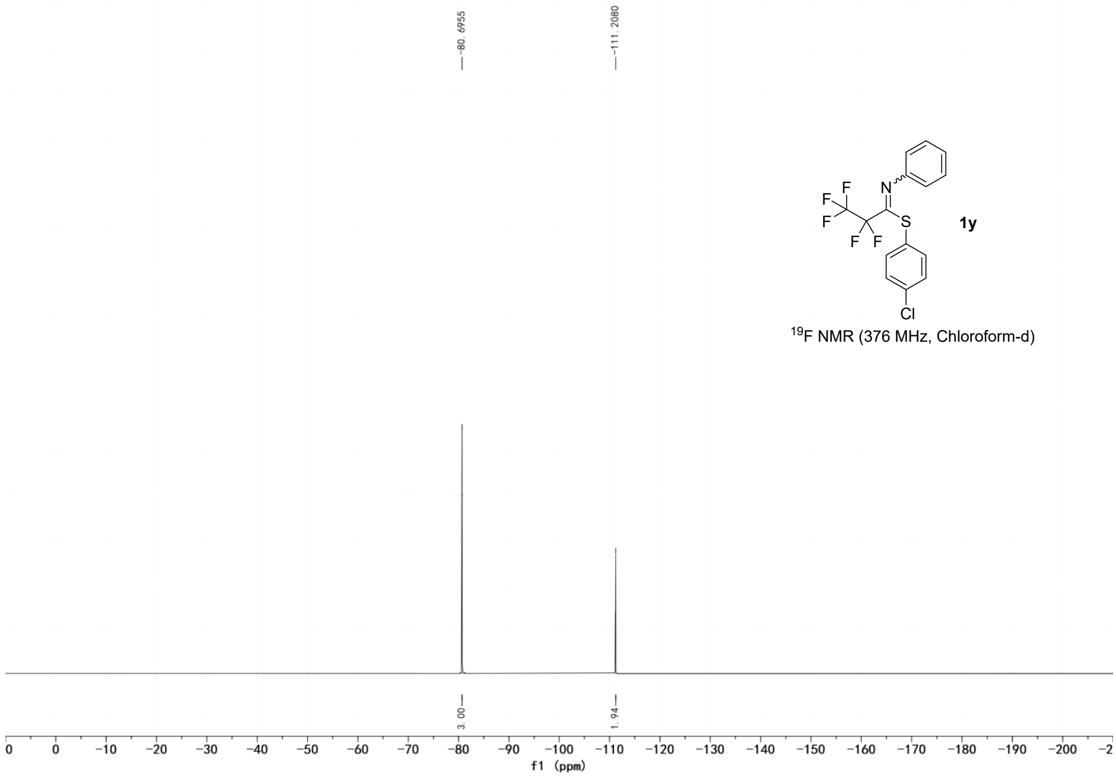

—80.6955
—111.2080
1y
$^{19}$F NMR (376 MHz, Chloroform-d)
3.00
1.94
f1 (ppm)

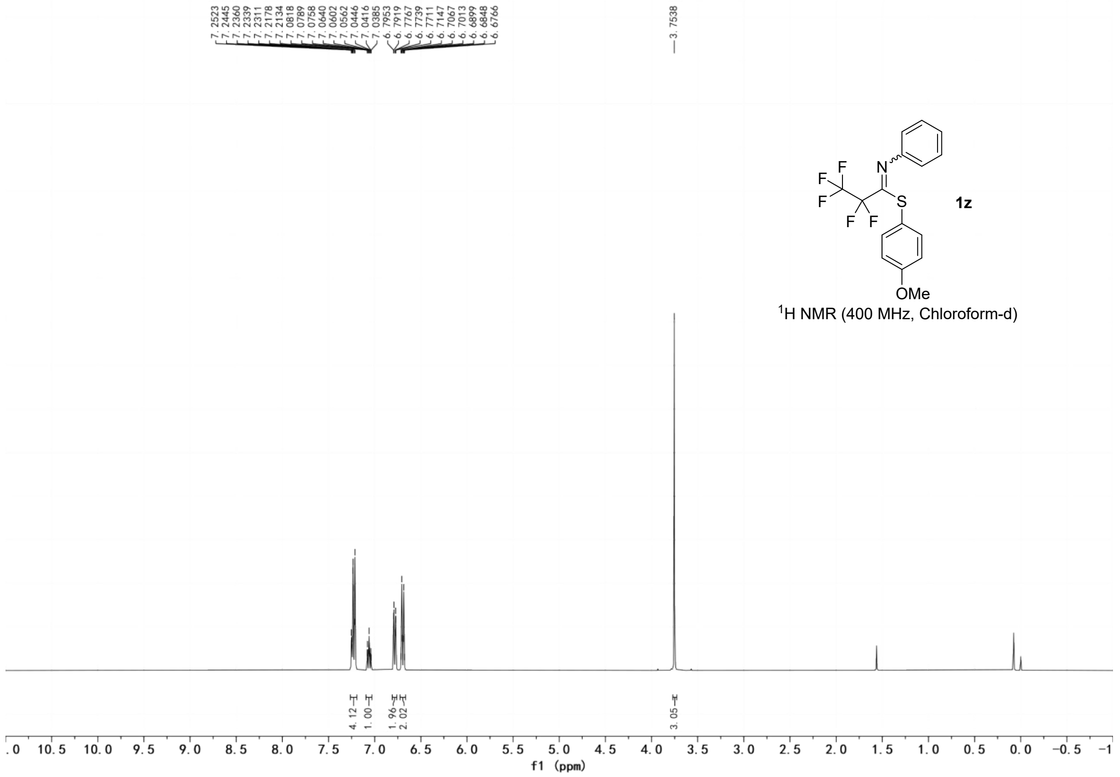
1z
$^1$H NMR (400 MHz, Chloroform-d)

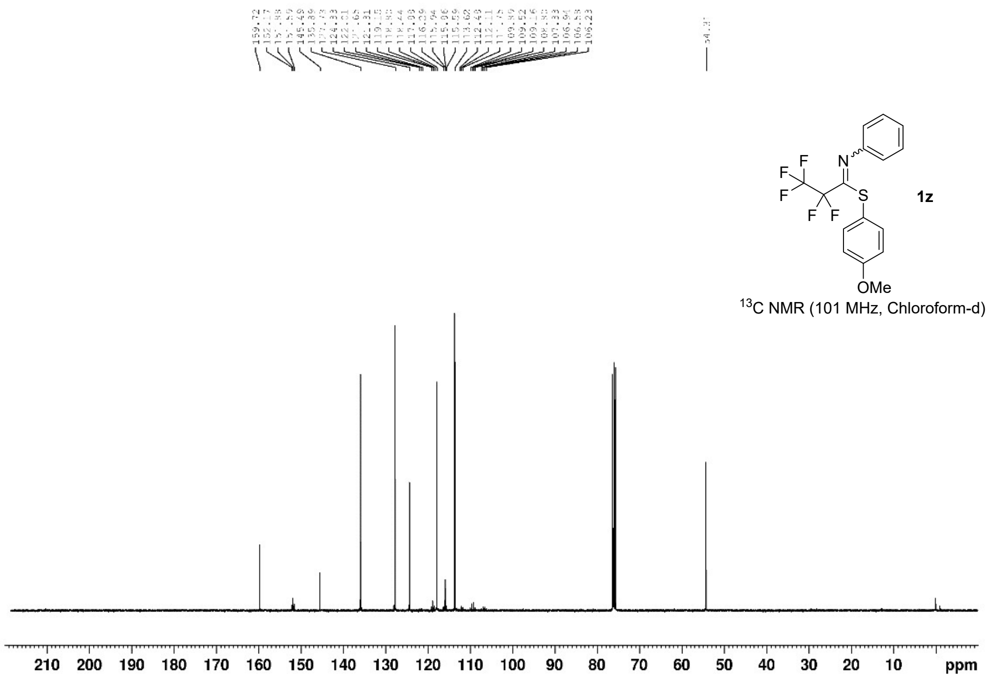
1z
$^{13}$C NMR (101 MHz, Chloroform-d)

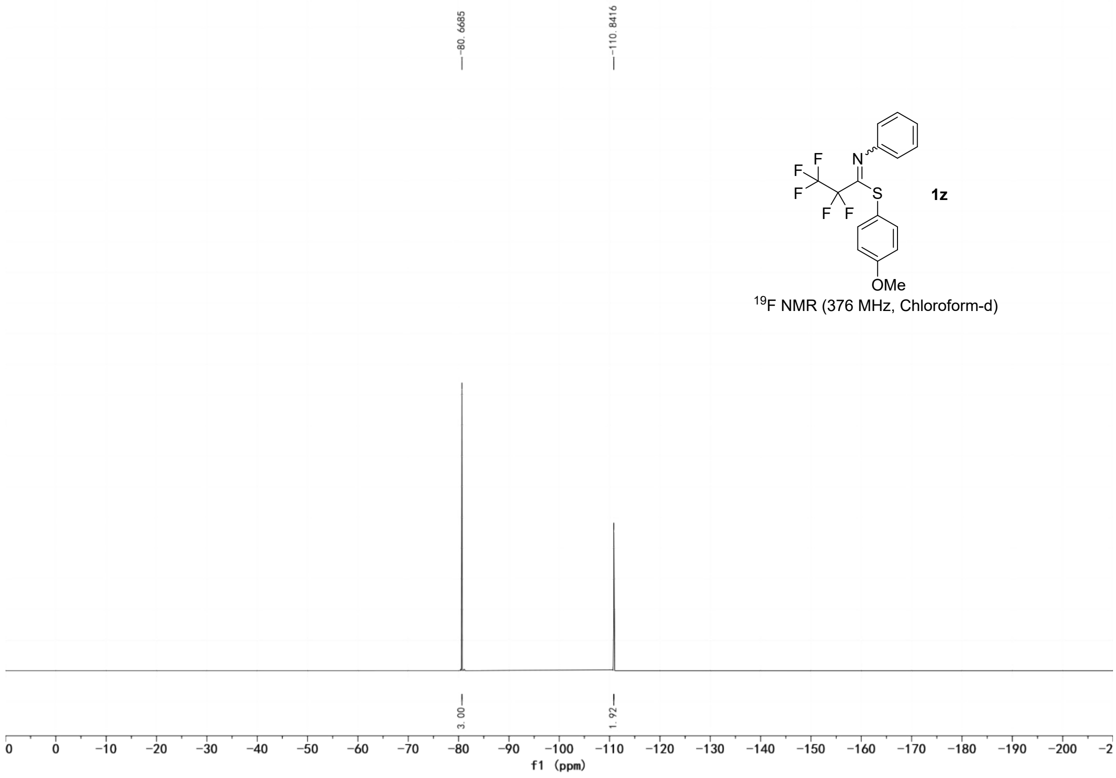

$^{19}$F NMR (376 MHz, Chloroform-d)

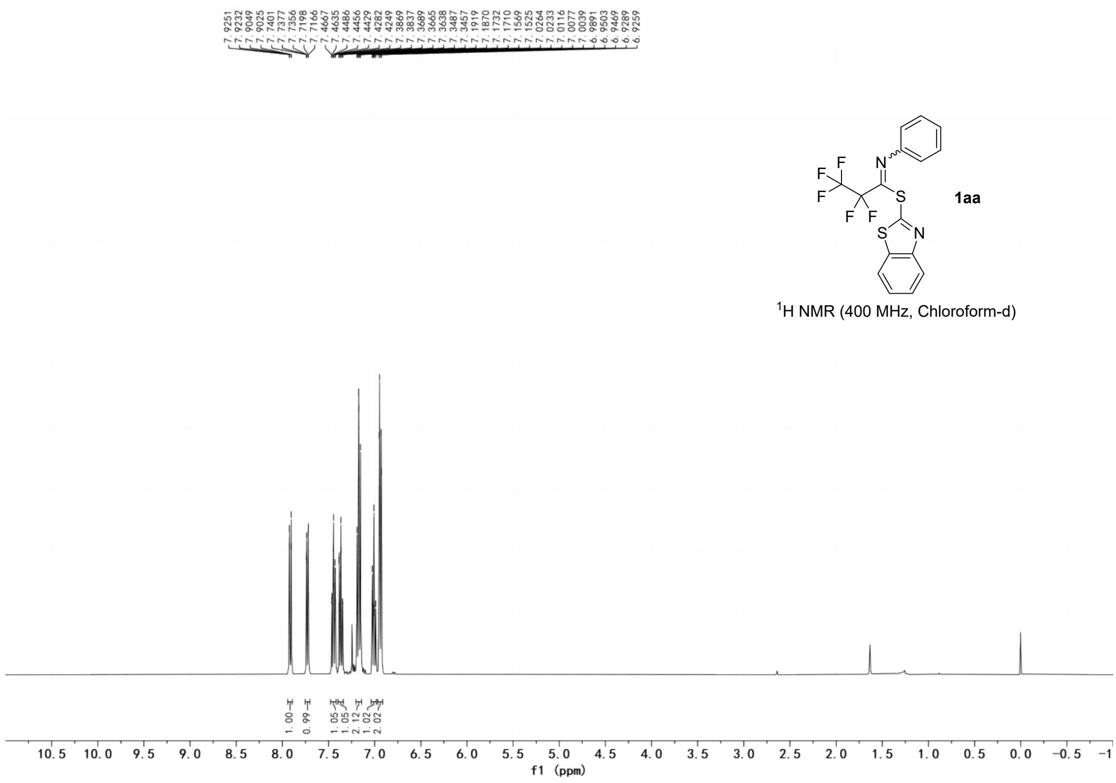
1aa
$^{1}$H NMR (400 MHz, Chloroform-d)
f1 (ppm)

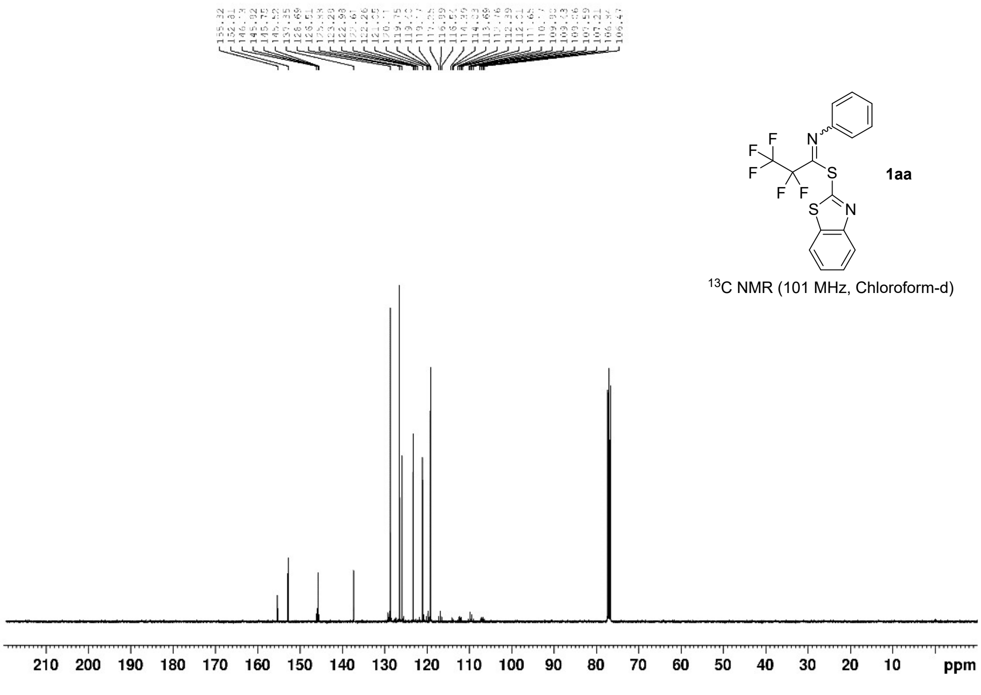
1aa
$^{13}$C NMR (101 MHz, Chloroform-d)
ppm

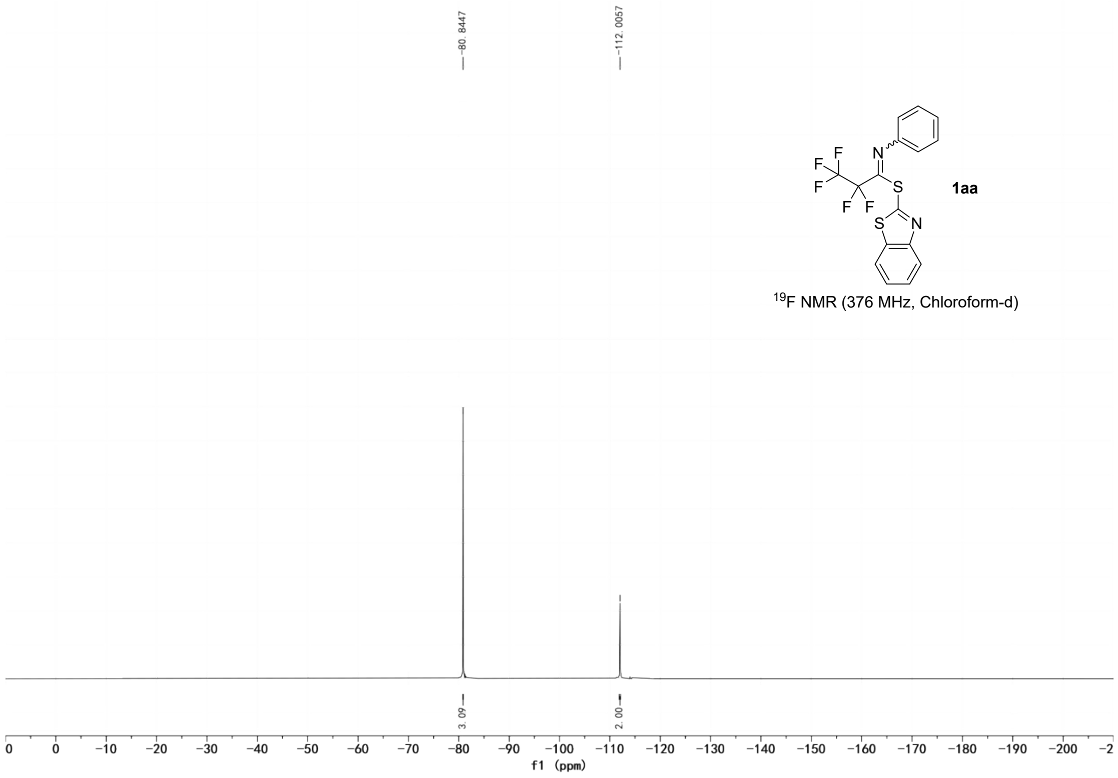


# NMR Spectra of the Products

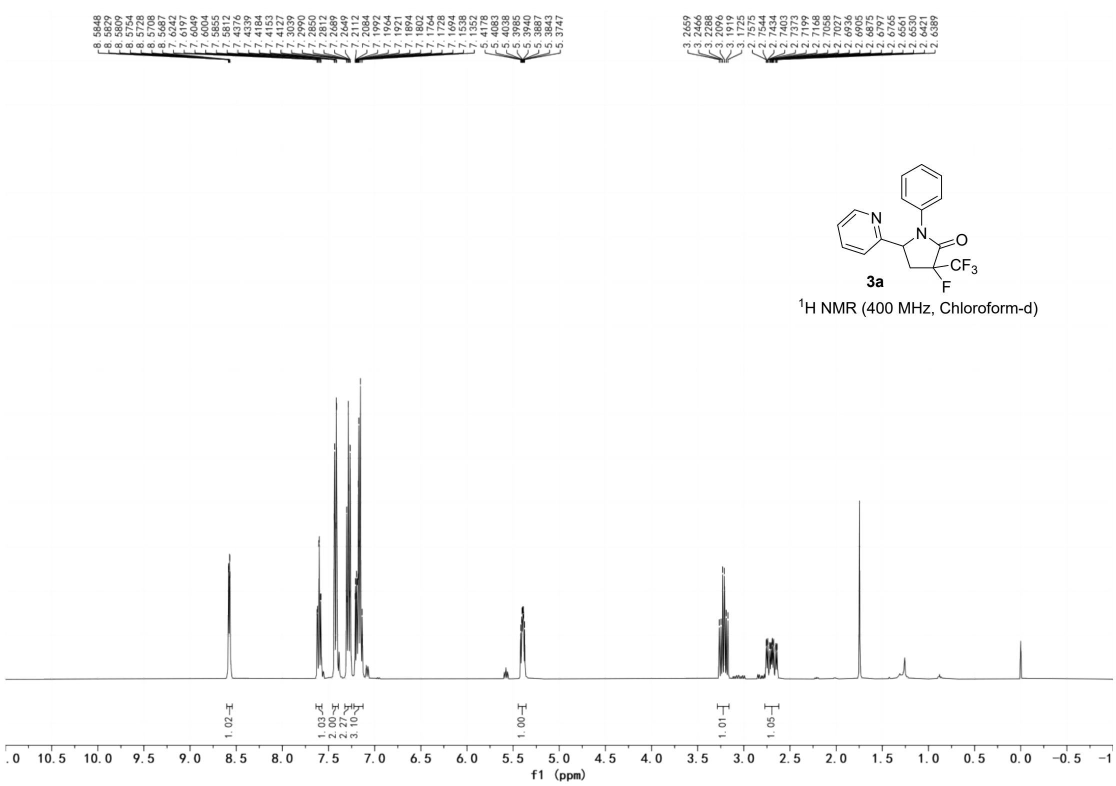

3a
$^1$H NMR (400 MHz, Chloroform-d)
f1 (ppm)


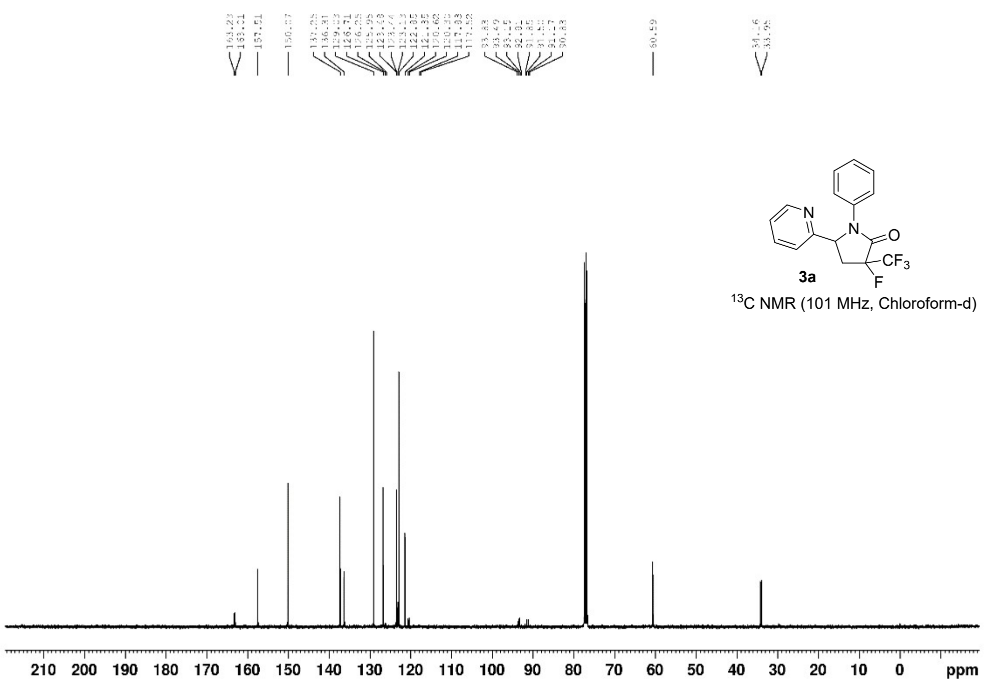

3a
$^{13}$C NMR (101 MHz, Chloroform-d)
ppm

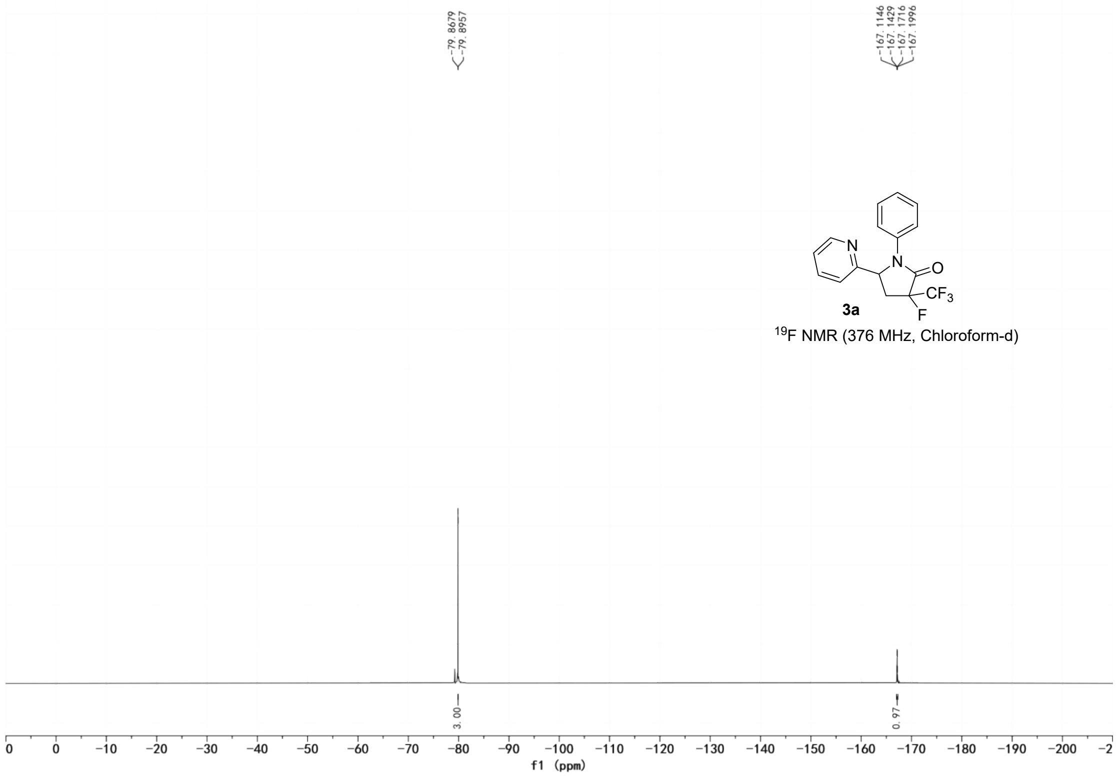

-79.8679
-79.8957
-167.1146
-167.1429
-167.1716
-167.1996
N
N
O
CF3
F
3a
19F NMR (376 MHz, Chloroform-d)
3.00
0.97
f1 (ppm)

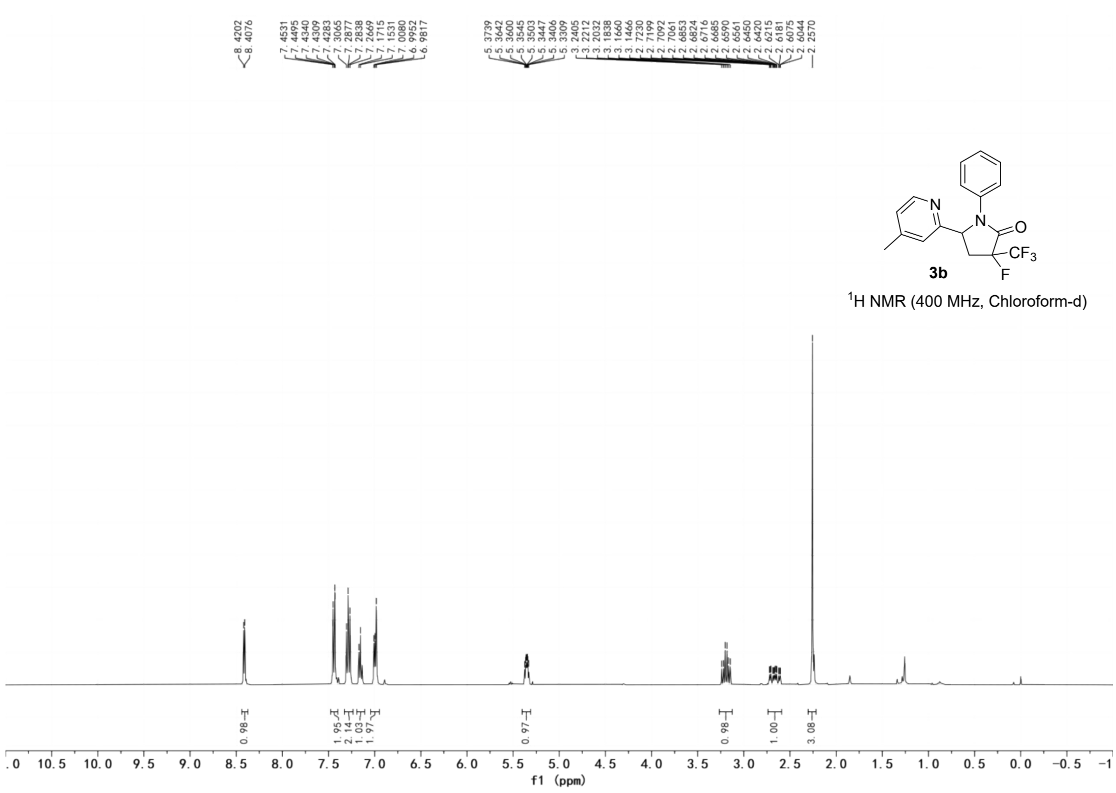


**3b**

$^1$H NMR (400 MHz, Chloroform-d)

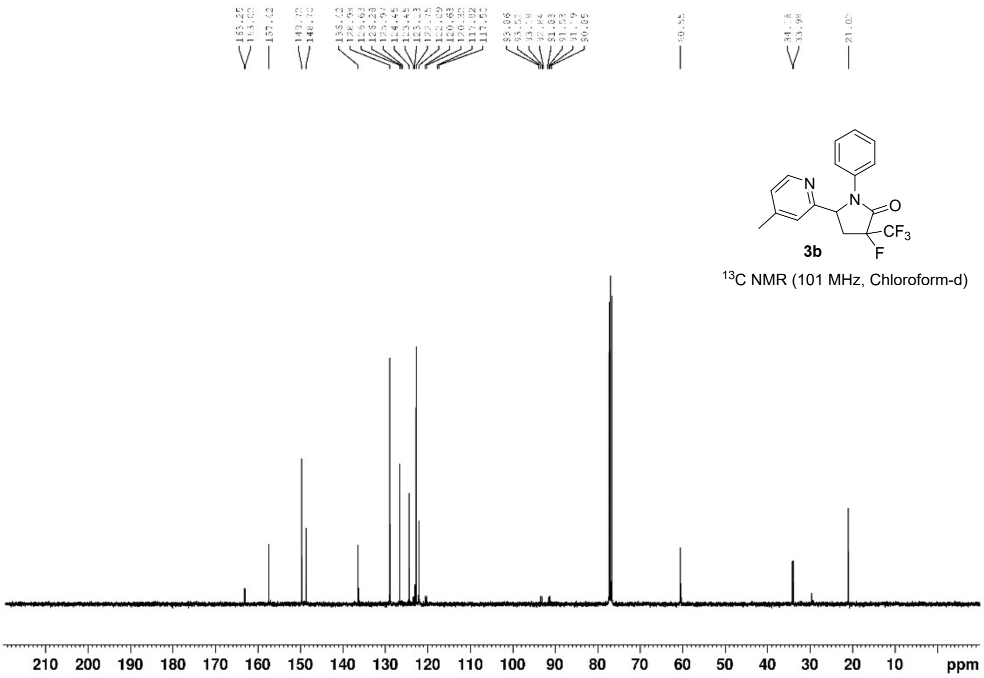


**3b**

$^{13}$C NMR (101 MHz, Chloroform-d)

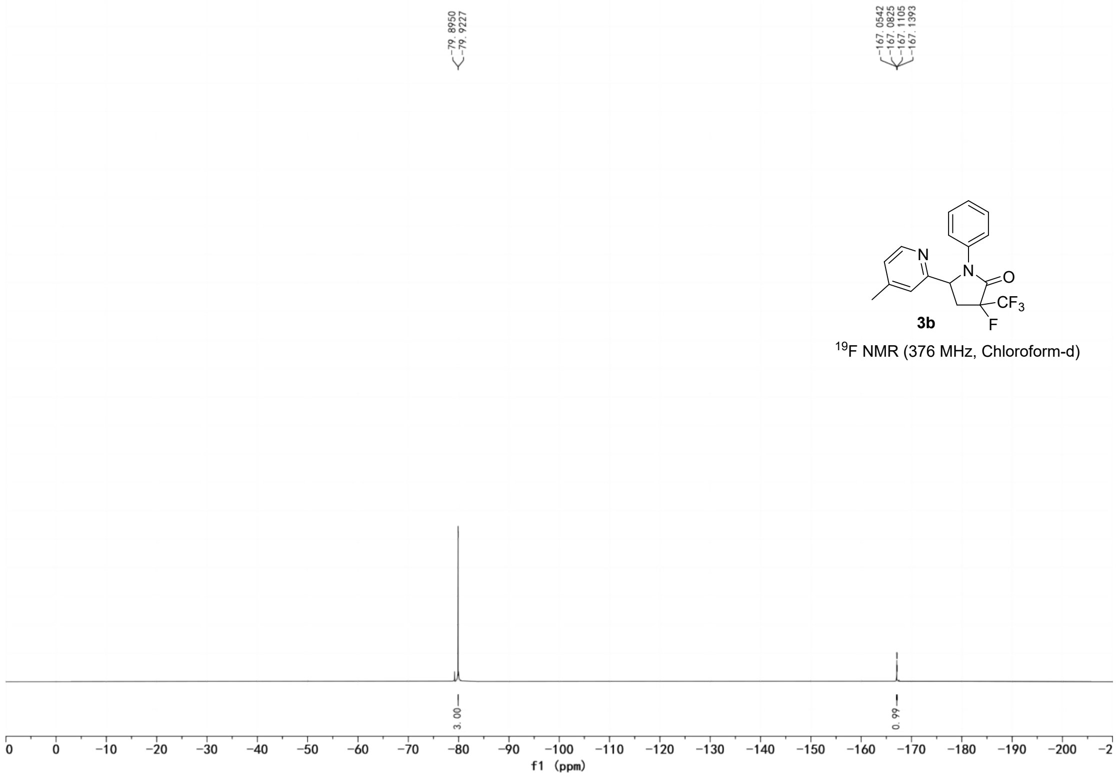
3b
$^{19}$F NMR (376 MHz, Chloroform-d)
f1 (ppm)

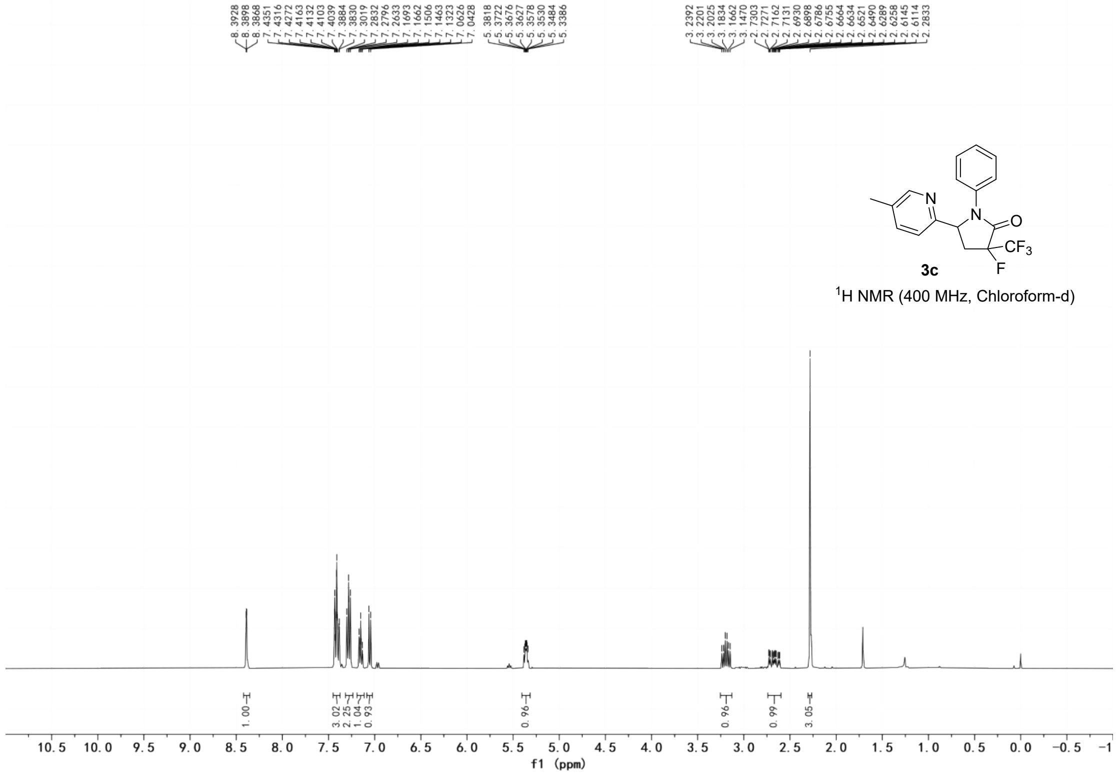
3c
$^{1}$H NMR (400 MHz, Chloroform-d)
f1 (ppm)

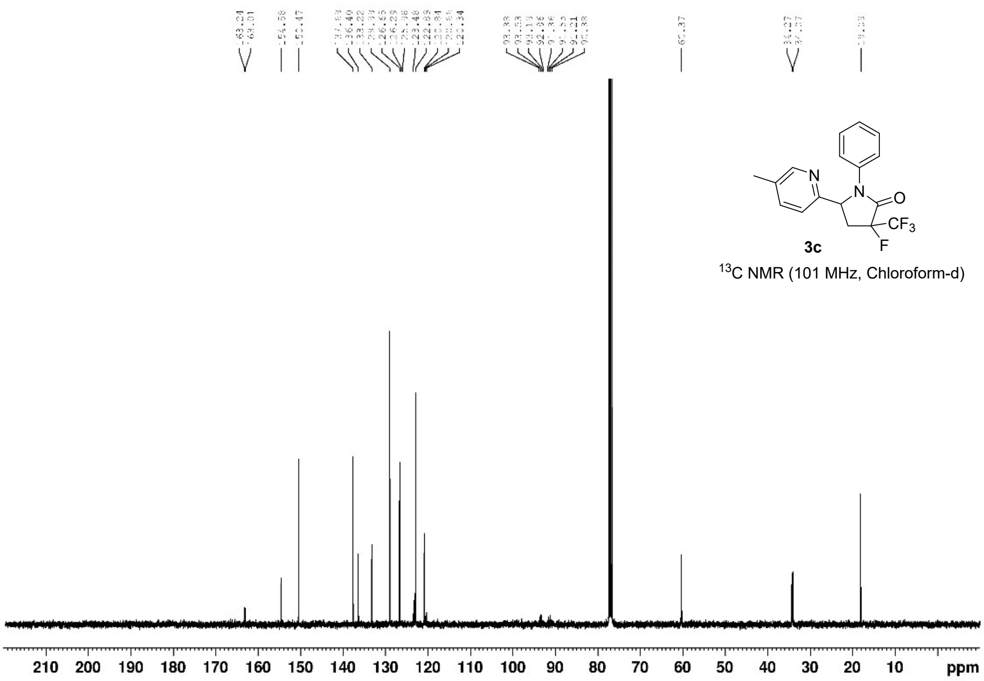
N
O
$CF_3$
F
3c
$^{13}C$ NMR (101 MHz, Chloroform-d)
210 200 190 180 170 160 150 140 130 120 110 100 90 80 70 60 50 40 30 20 10 ppm

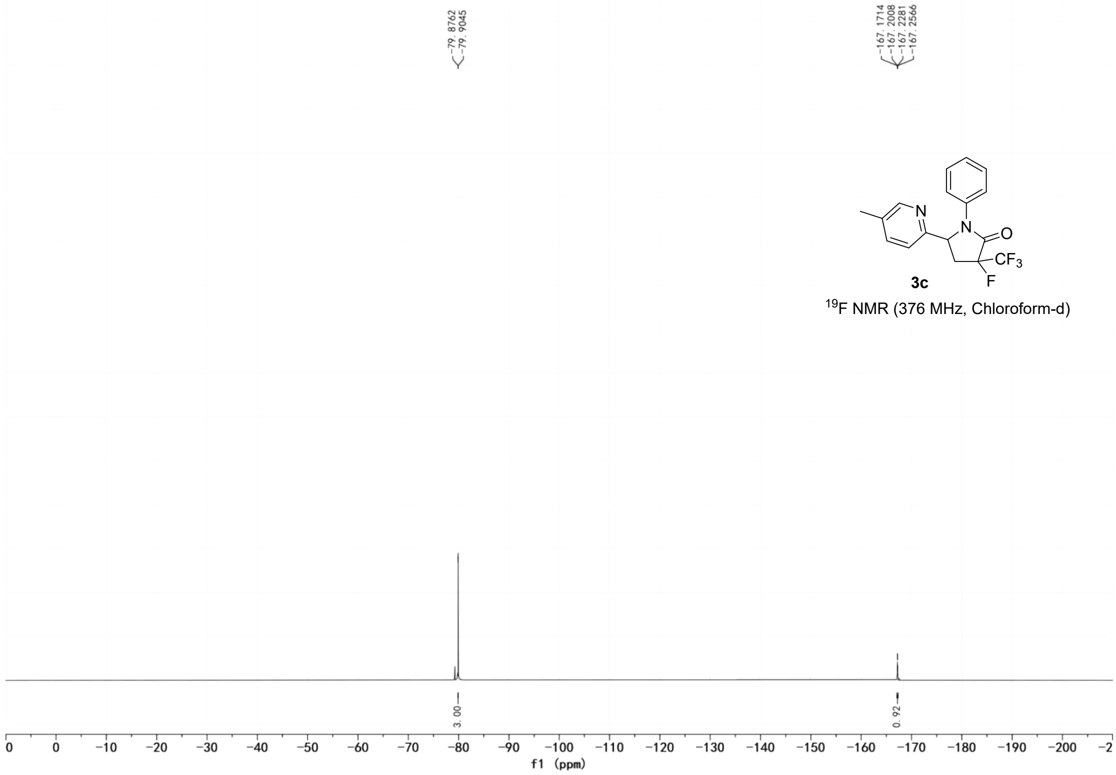
-79.8762
-79.9045
-167.1714
-167.2008
-167.2281
-167.2566
N
O
$CF_3$
F
3c
$^{19}F$ NMR (376 MHz, Chloroform-d)
3.00
0.92
f1 (ppm)

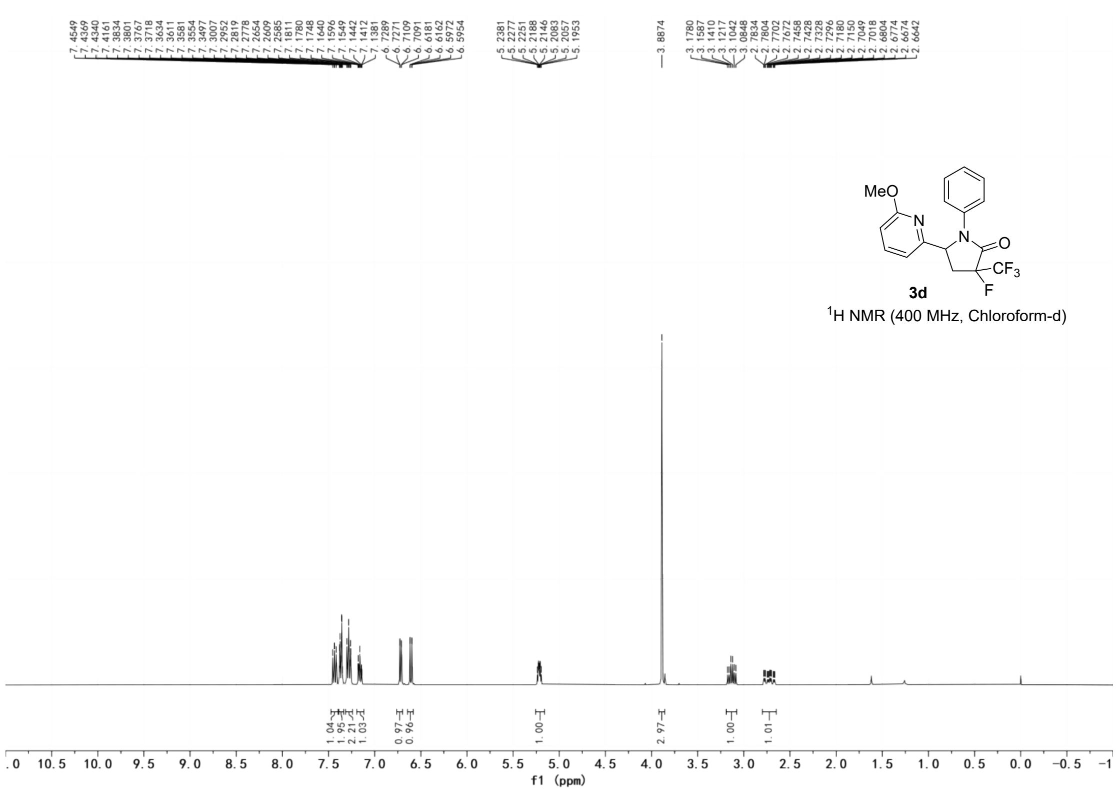


**3d**

$^{1}$H NMR (400 MHz, Chloroform-d)

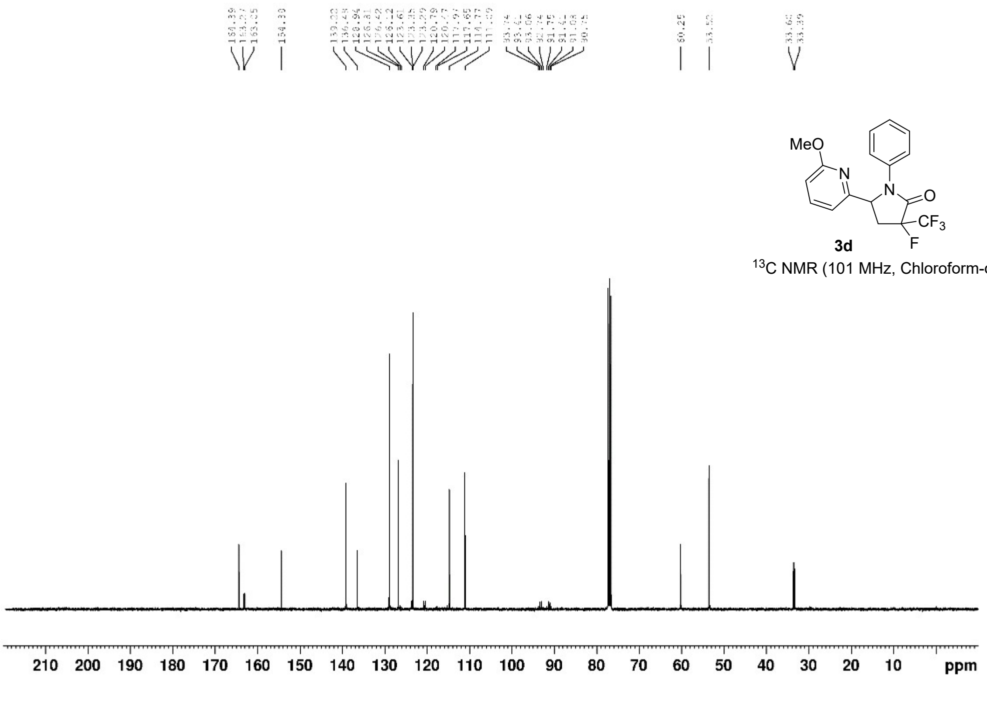


**3d**

$^{13}$C NMR (101 MHz, Chloroform-d)

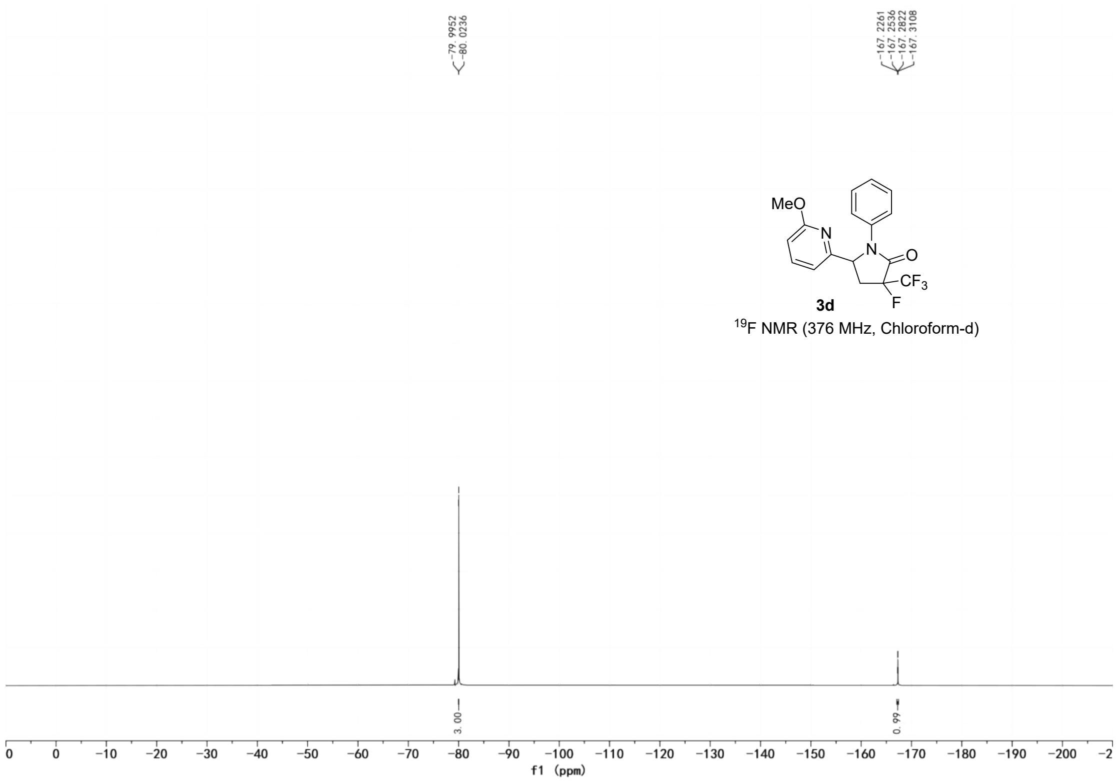
-79.9952
-80.0236
-167.2261
-167.2536
-167.2822
-167.3108
MeO
N
N
O
CF3
F
3d
19F NMR (376 MHz, Chloroform-d)
3.00
0.99
0 0 −10 −20 −30 −40 −50 −60 −70 −80 −90 −100 −110 −120 −130 −140 −150 −160 −170 −180 −190 −200 −2
f1 (ppm)

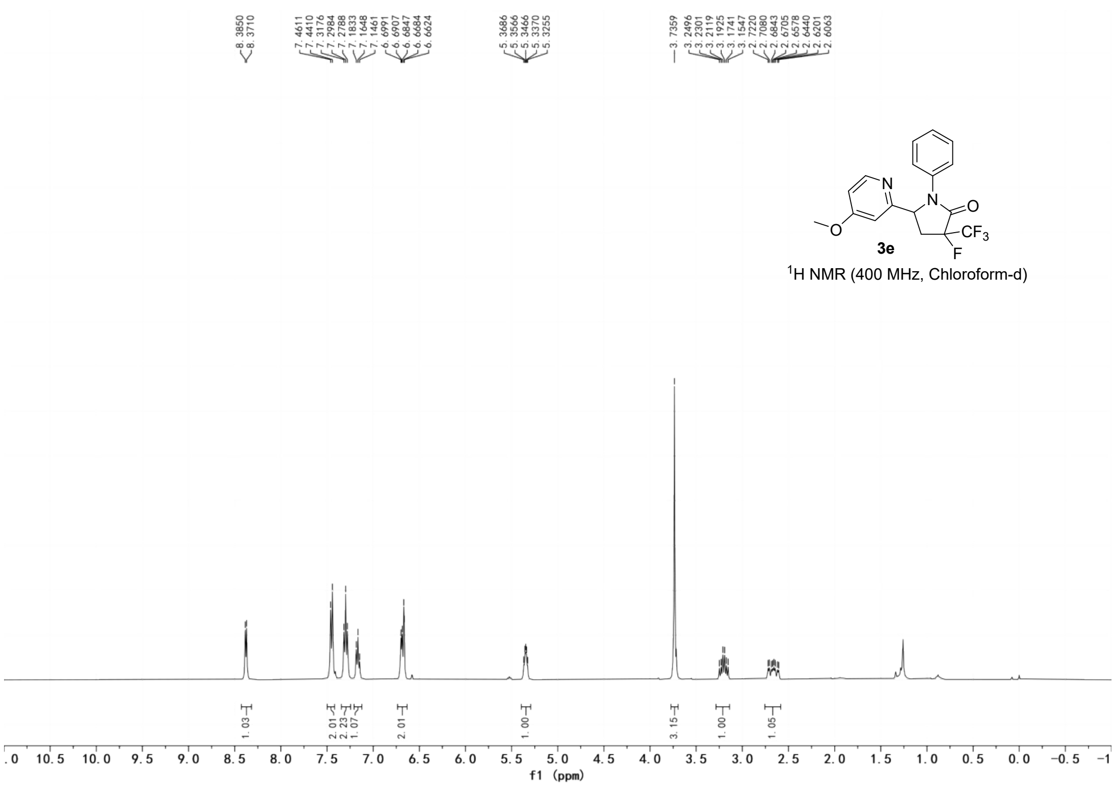
3e
$^{1}$H NMR (400 MHz, Chloroform-d)
f1 (ppm)

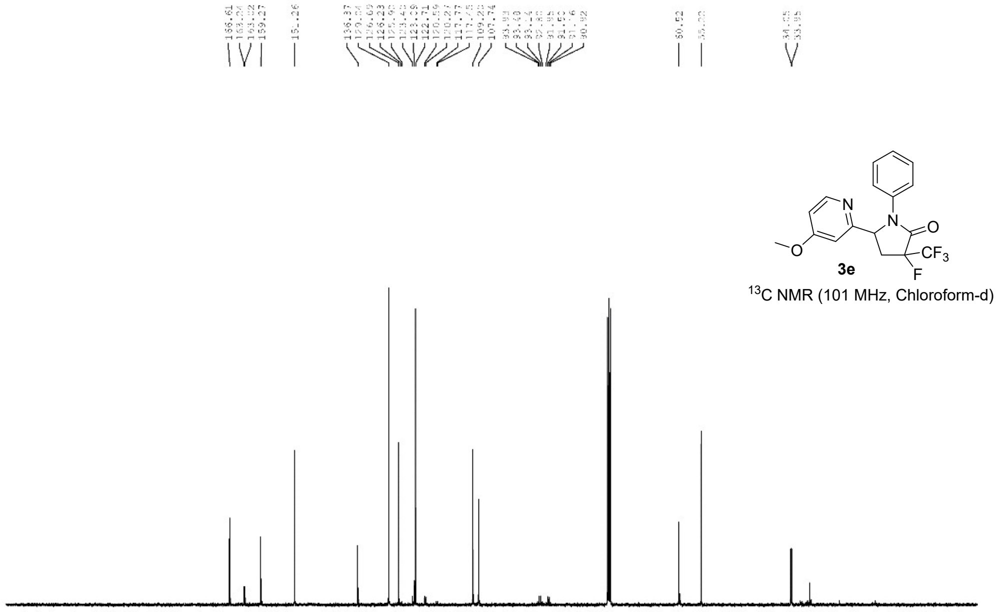
3e
$^{13}$C NMR (101 MHz, Chloroform-d)
ppm

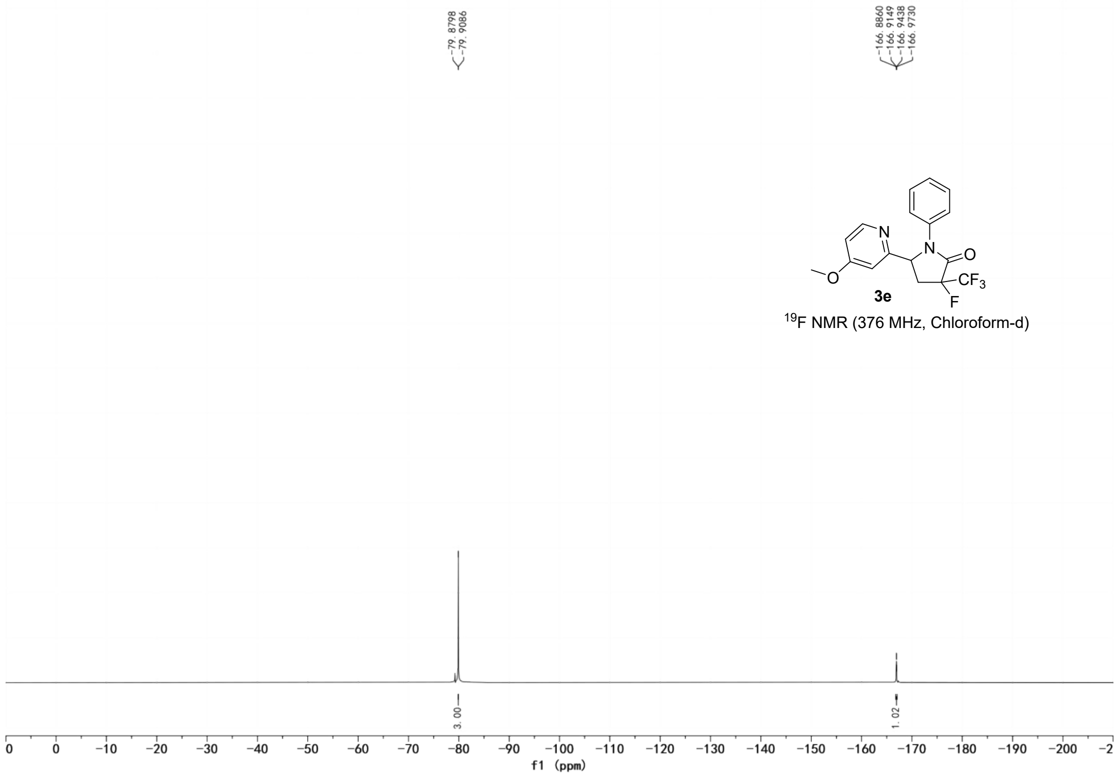


$^{19}F$ NMR (376 MHz, Chloroform-d)

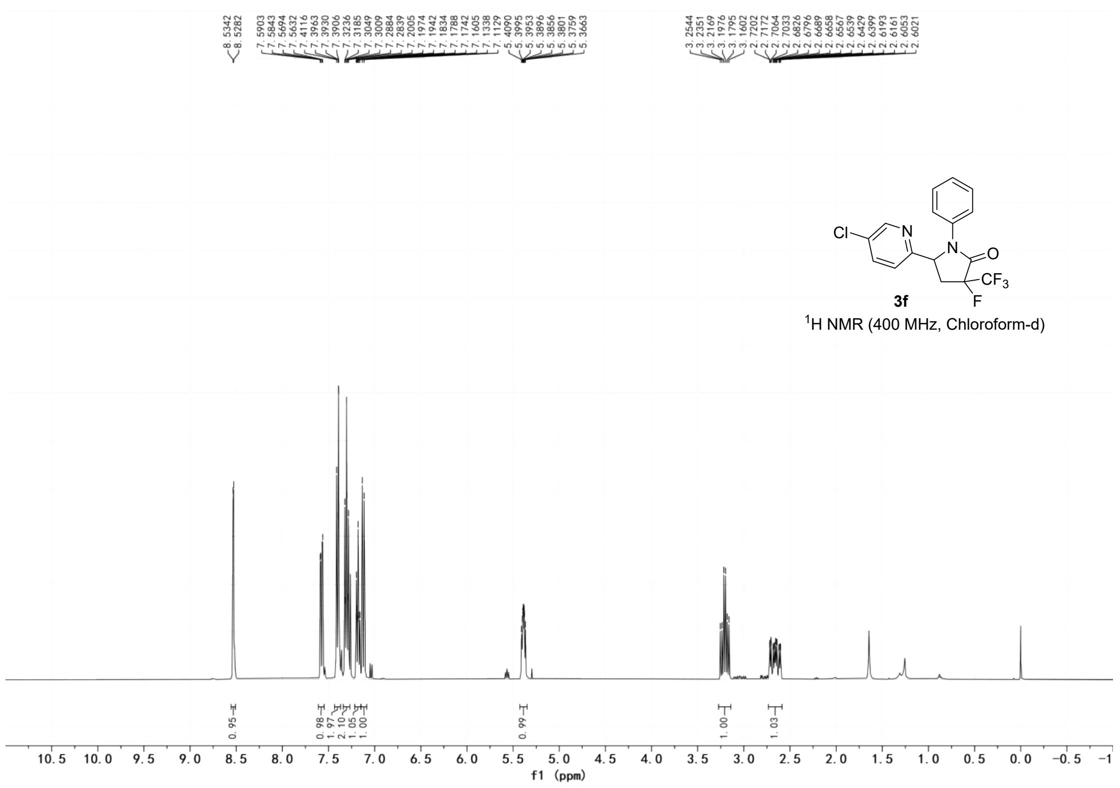
Cl
N
O
CF3
F
3f
1H NMR (400 MHz, Chloroform-d)
f1 (ppm)

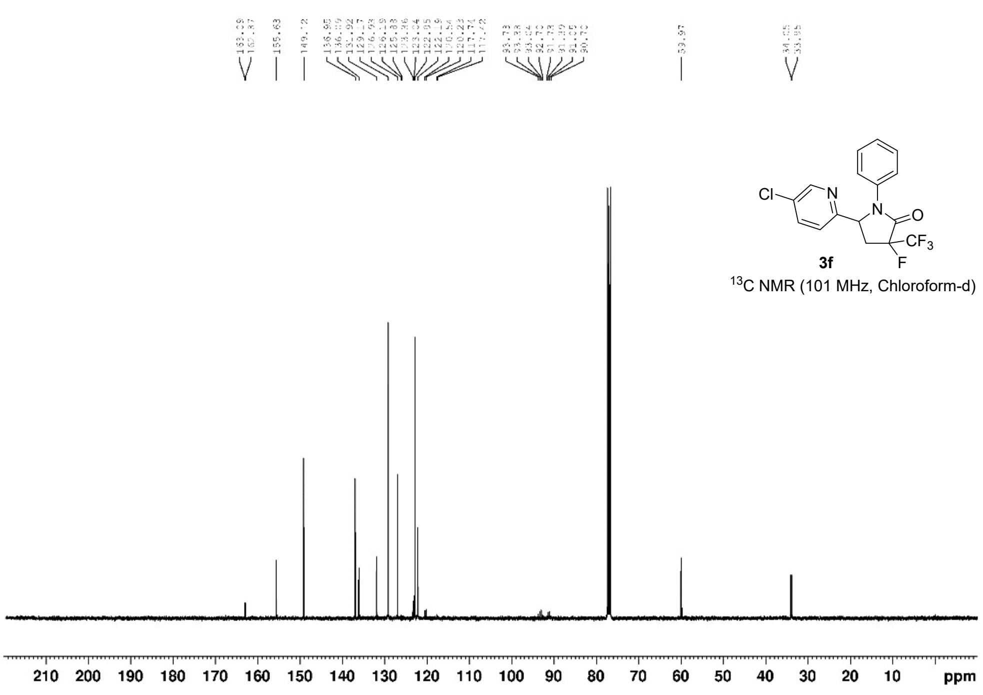
Cl
N
O
CF3
F
3f
13C NMR (101 MHz, Chloroform-d)
ppm

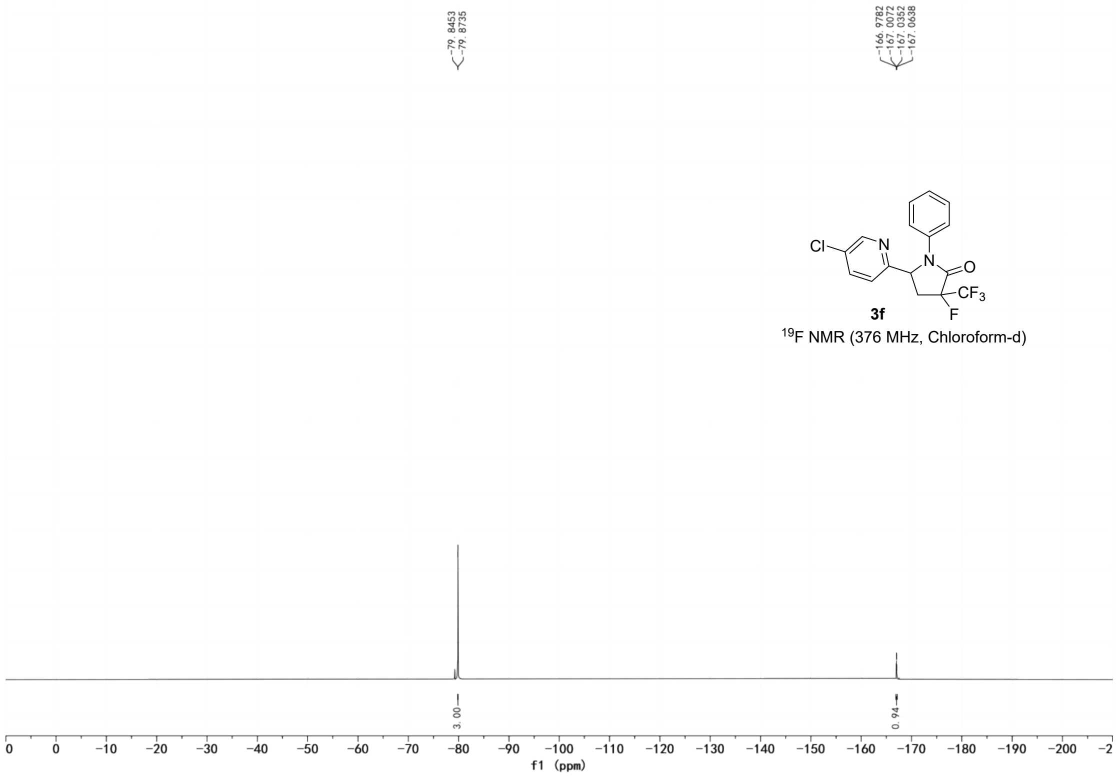

-79.8453
-79.8735
-166.9782
-167.0072
-167.0352
-167.0638
Cl
N
N
O
CF3
F
3f
19F NMR (376 MHz, Chloroform-d)
3.00
0.94
f1 (ppm)

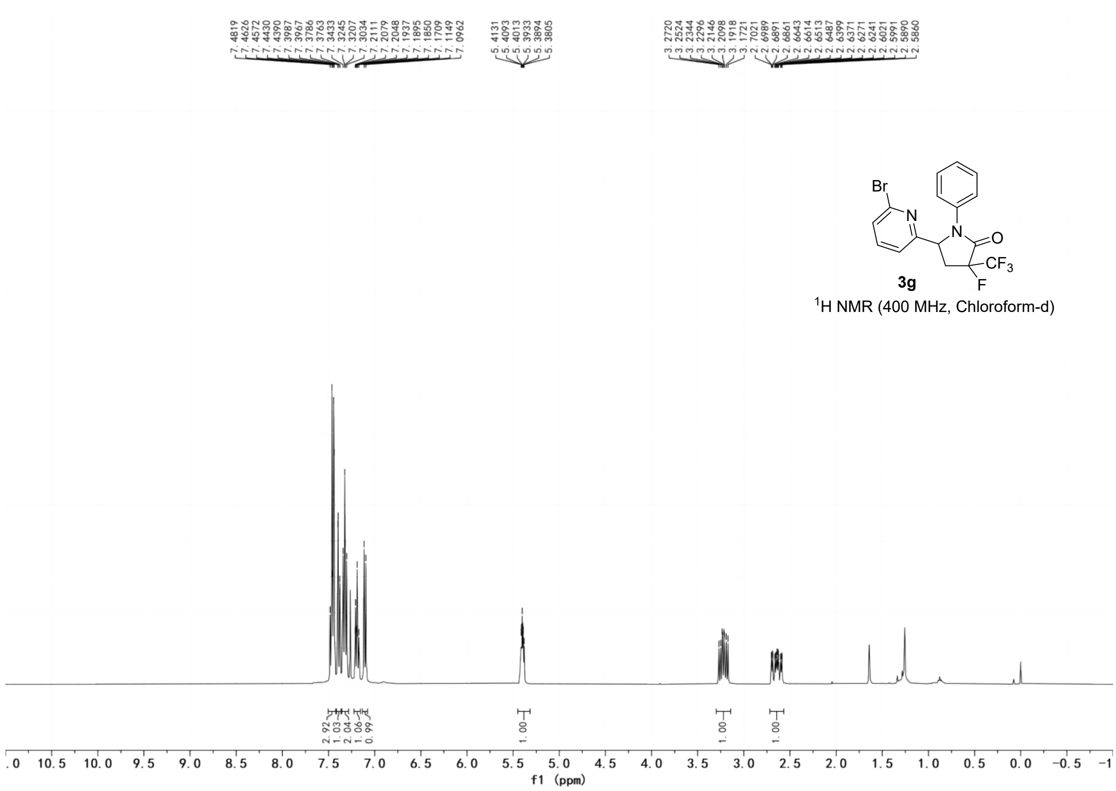
Br
N
N
O
CF3
F
3g
1H NMR (400 MHz, Chloroform-d)
f1 (ppm)

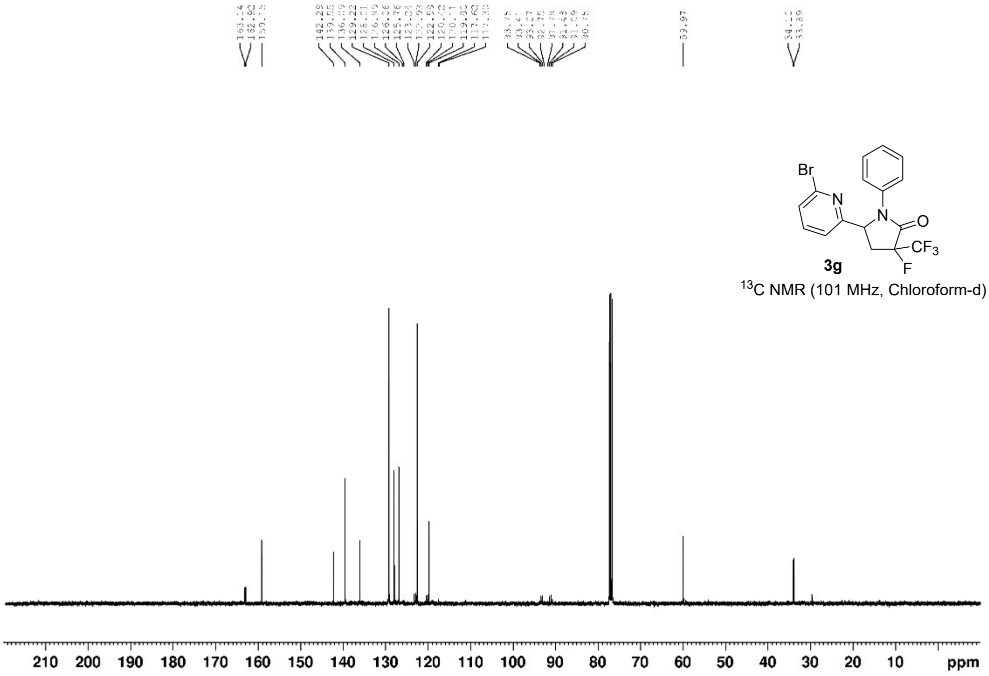
Br
N
N
O
CF3
F
3g
13C NMR (101 MHz, Chloroform-d)
ppm

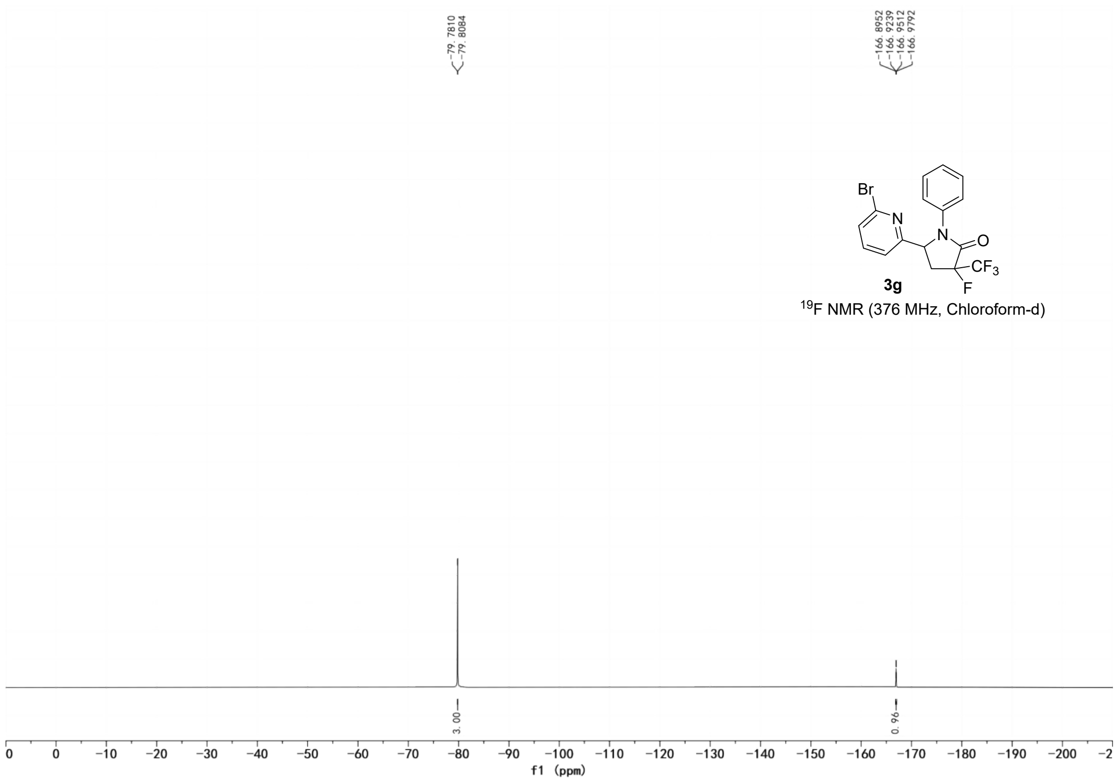


**3g**

$^{19}$F NMR (376 MHz, Chloroform-d)

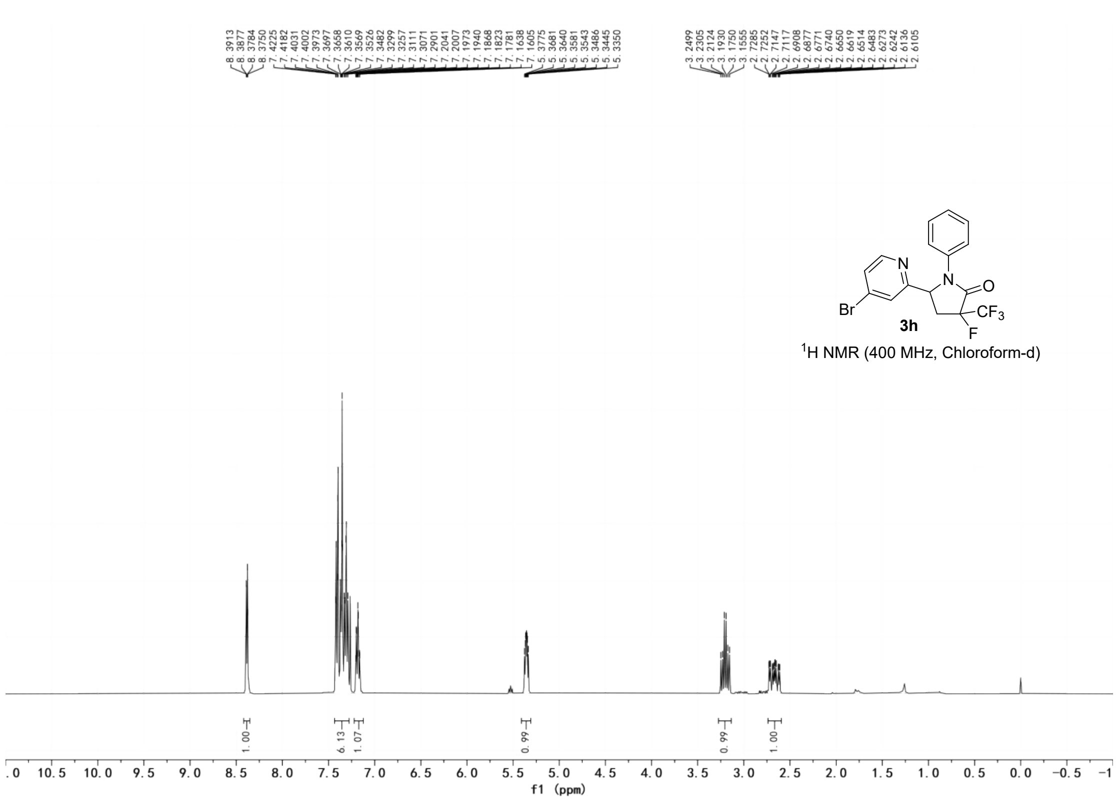
3h
1H NMR (400 MHz, Chloroform-d)
f1 (ppm)

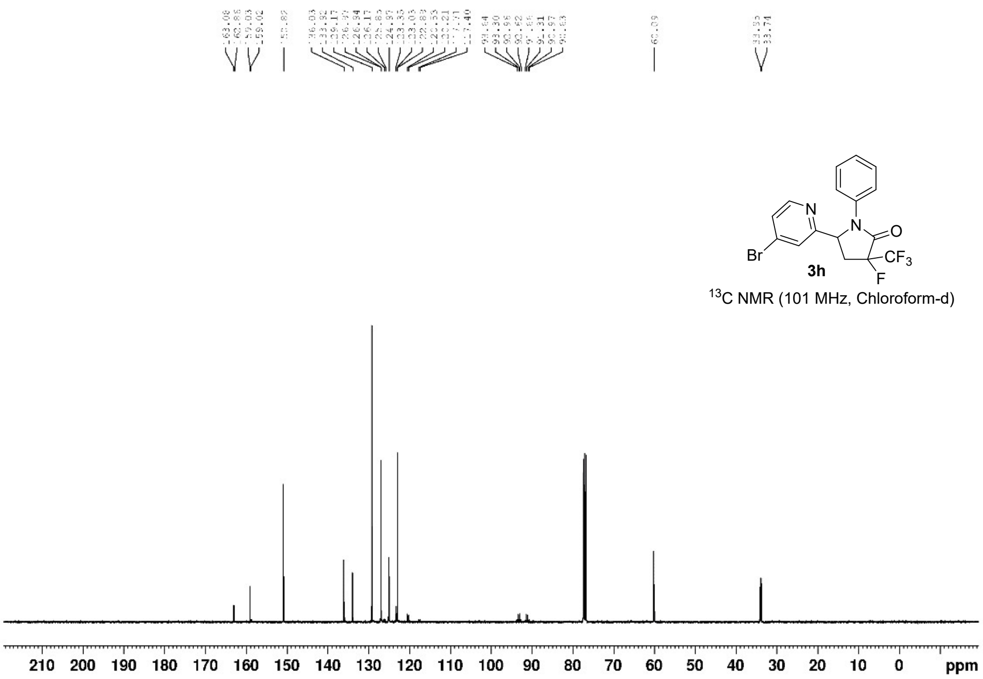
3h
13C NMR (101 MHz, Chloroform-d)
ppm

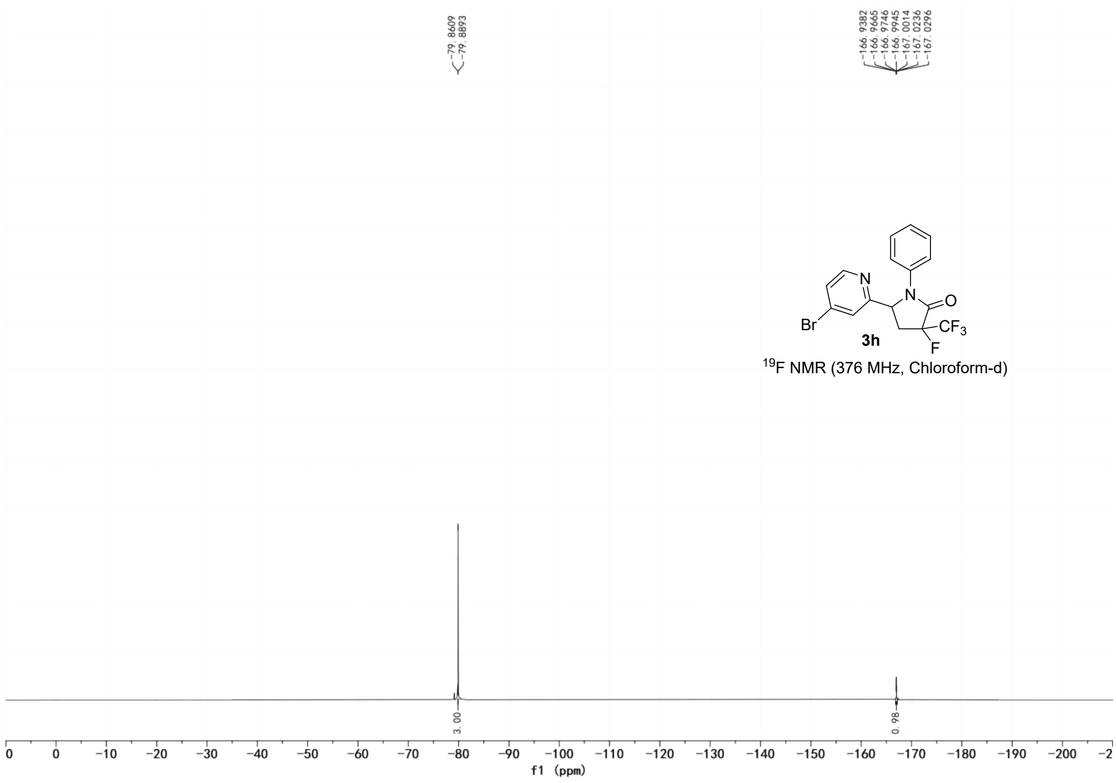
-79.8609
-79.8893
-166.9382
-166.9665
-166.9746
-166.9945
-167.0014
-167.0236
-167.0296
Br
N
N
O
CF3
F
3h
19F NMR (376 MHz, Chloroform-d)
3.00
0.98
f1 (ppm)

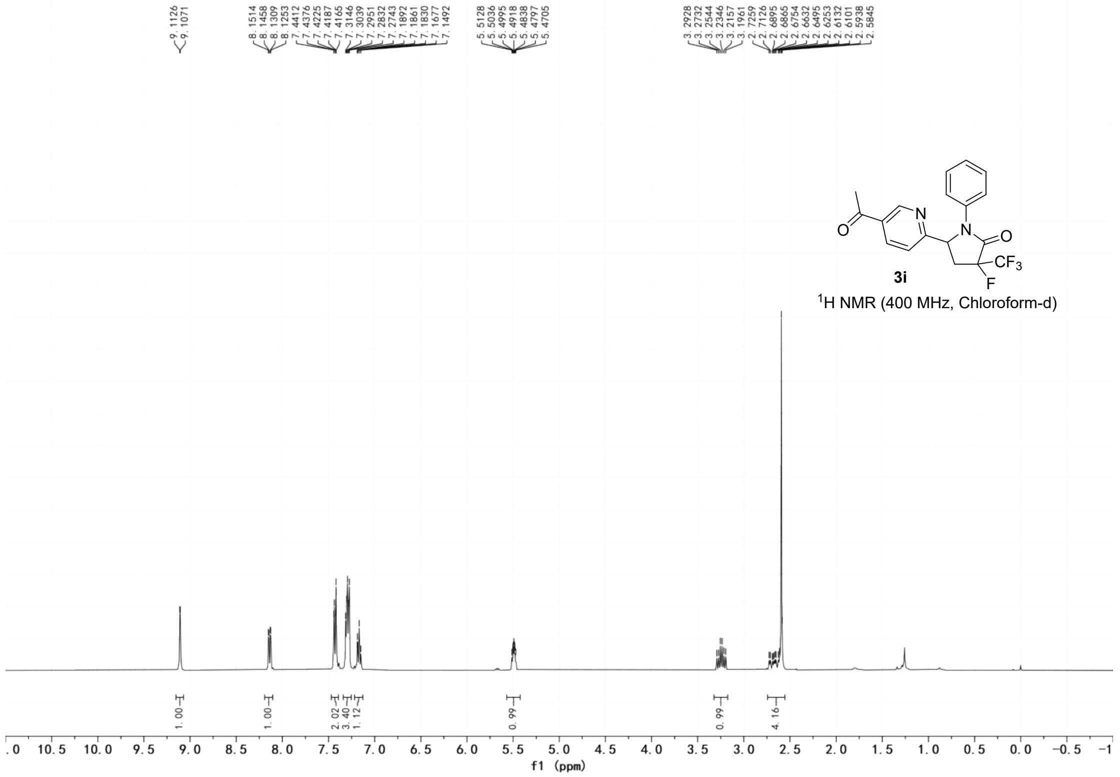

3i
$^1$H NMR (400 MHz, Chloroform-d)
f1 (ppm)


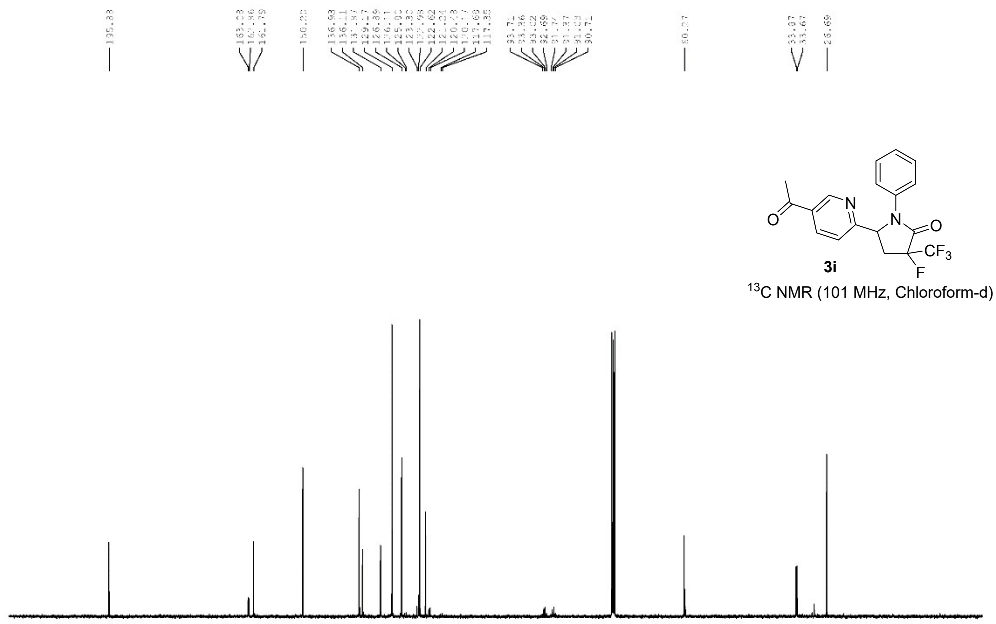

3i
$^{13}$C NMR (101 MHz, Chloroform-d)
ppm

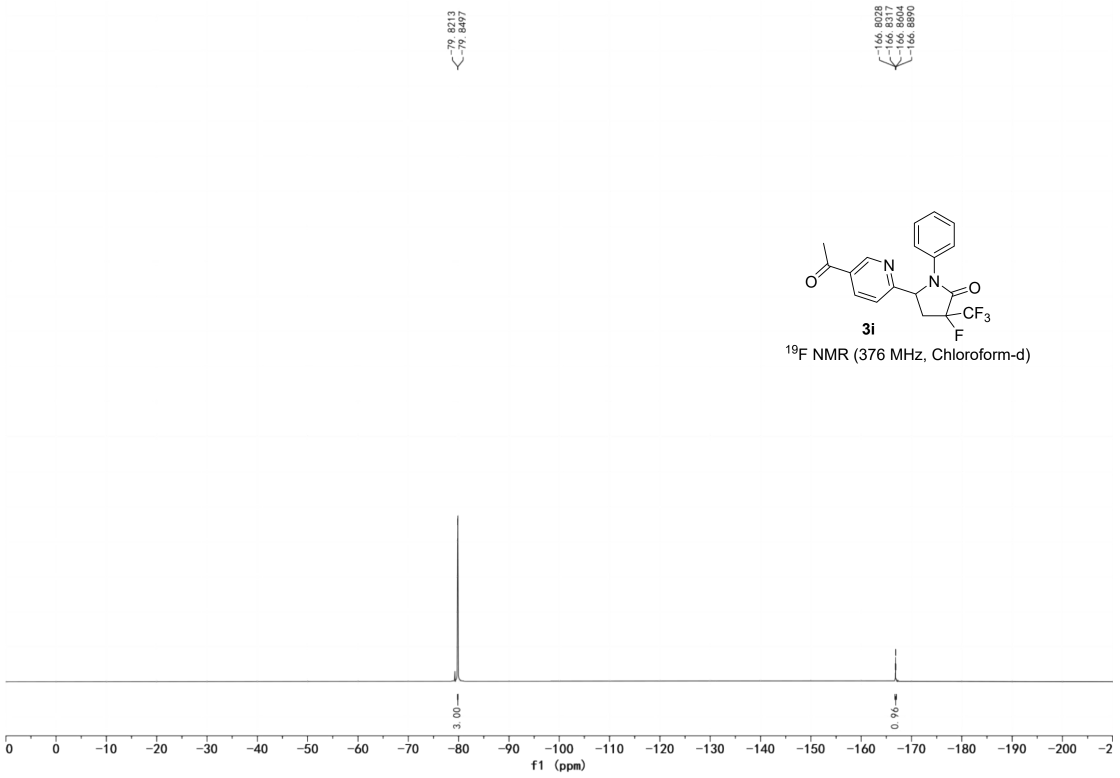
-79.8213
-79.8497
-166.8028
-166.8317
-166.8604
-166.8890
N
O
O
N
CF3
F
3i
19F NMR (376 MHz, Chloroform-d)
3.00
0.96
f1 (ppm)

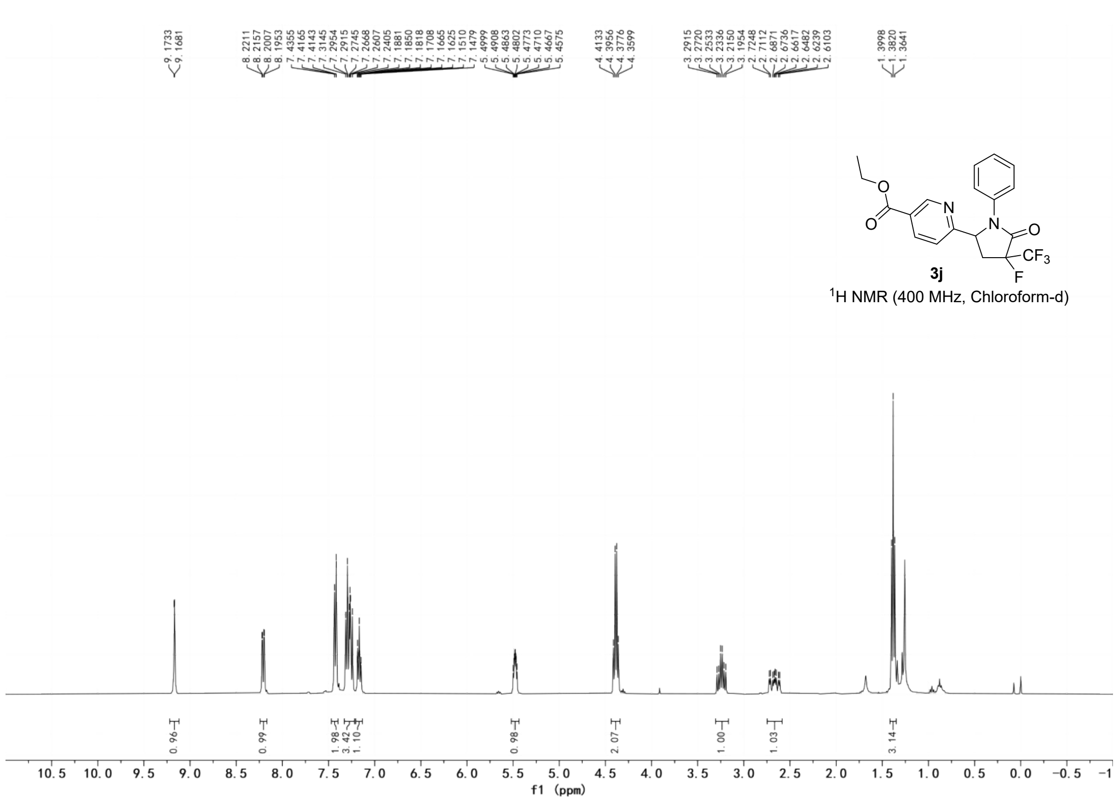


**3j**

$^{1}H$ NMR (400 MHz, Chloroform-d)

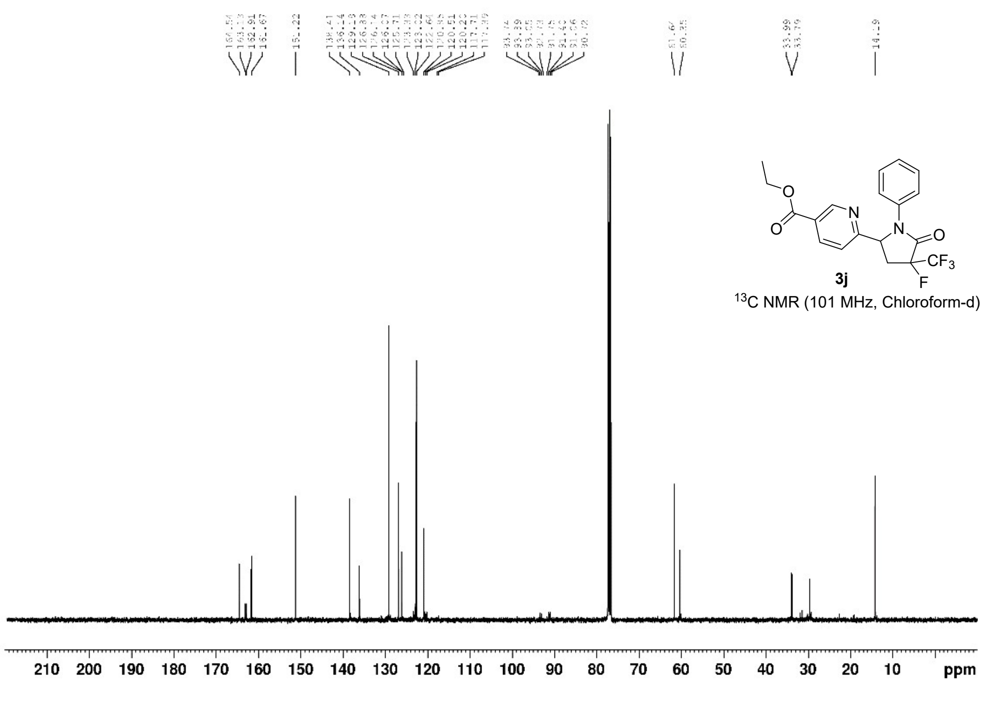


**3j**

$^{13}C$ NMR (101 MHz, Chloroform-d)

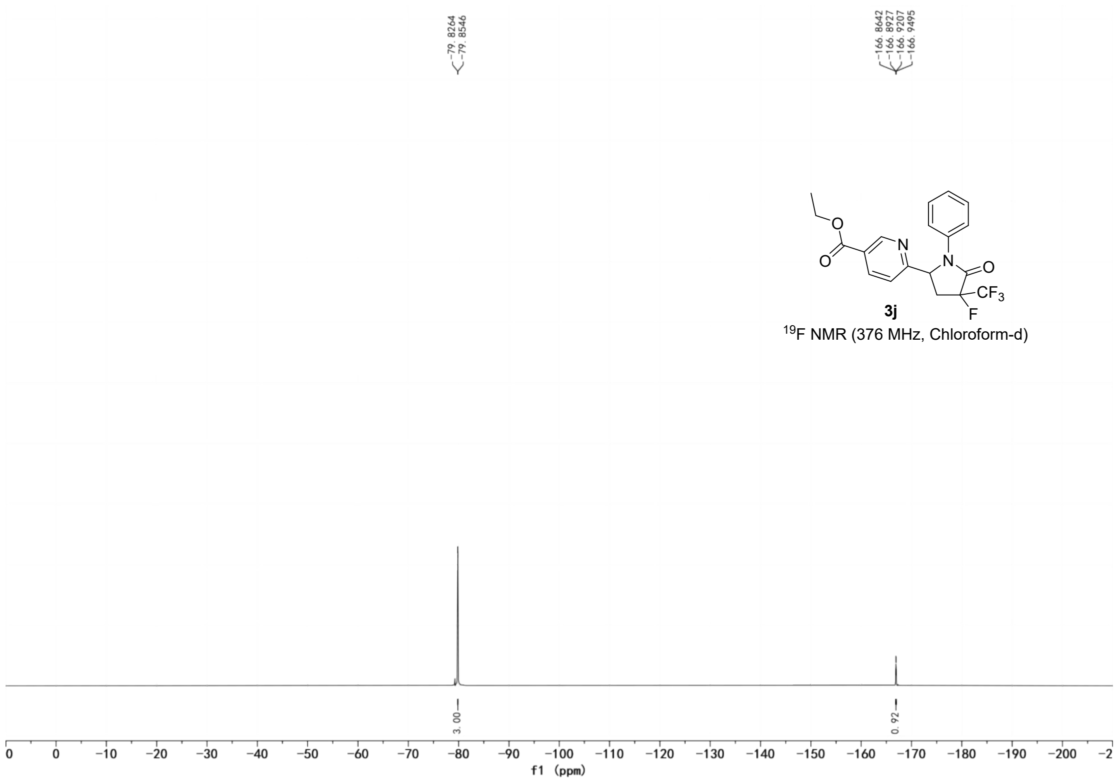

-79.8264
-79.8546
-166.8642
-166.8927
-166.9207
-166.9495
3j
$^{19}$F NMR (376 MHz, Chloroform-d)
3.00
0.92
f1 (ppm)

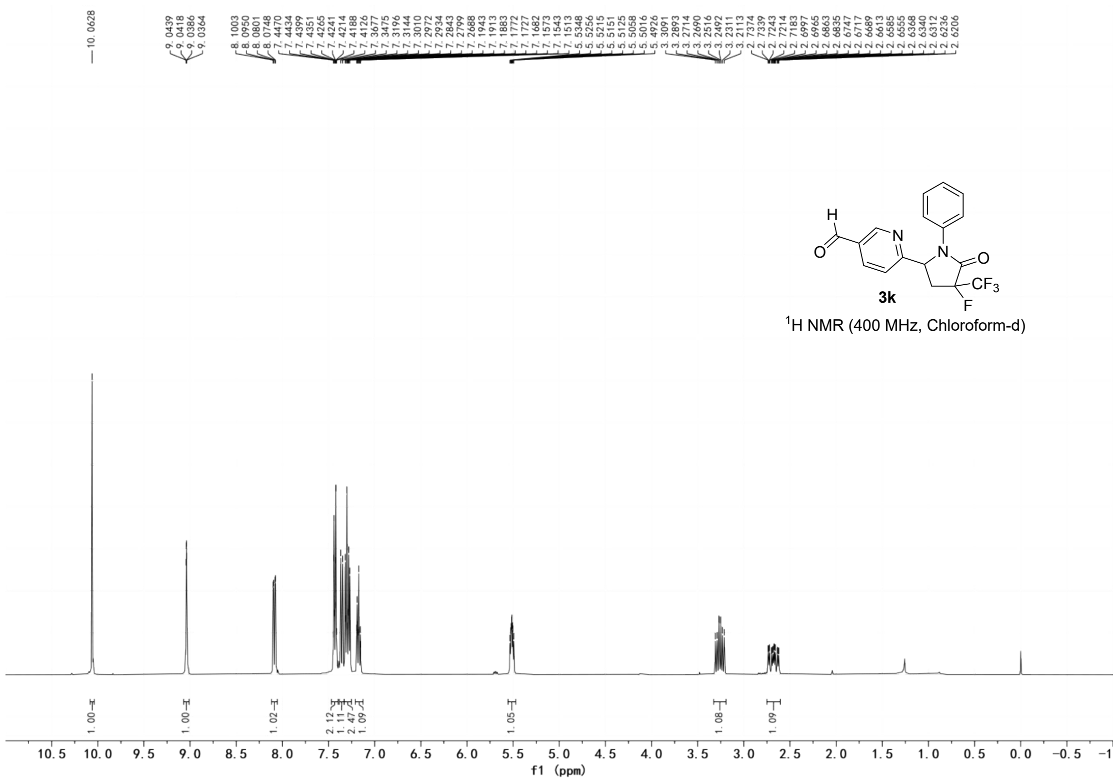
3k
$^{1}$H NMR (400 MHz, Chloroform-d)
f1 (ppm)

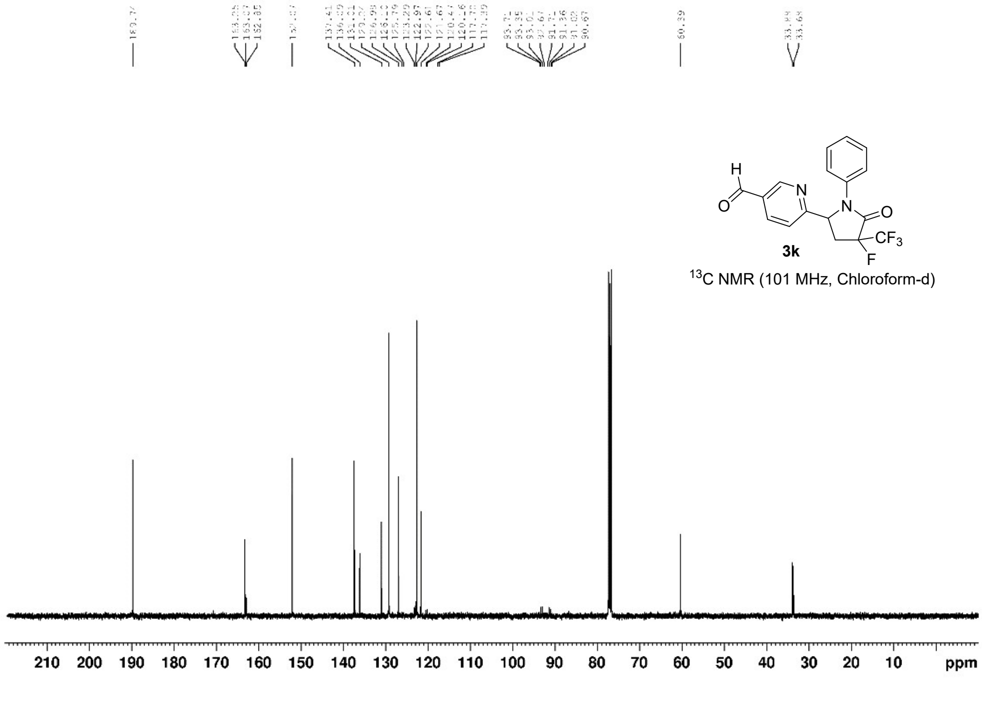
3k
$^{13}$C NMR (101 MHz, Chloroform-d)
ppm

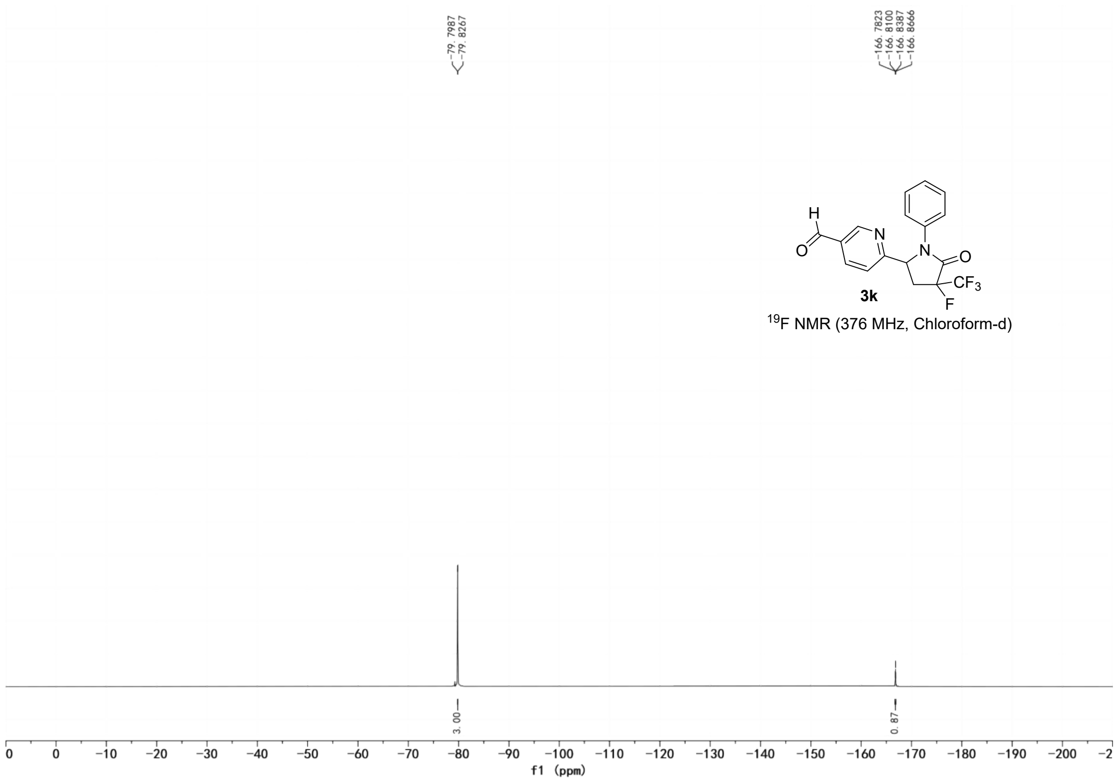


**3k**

$^{19}$F NMR (376 MHz, Chloroform-d)

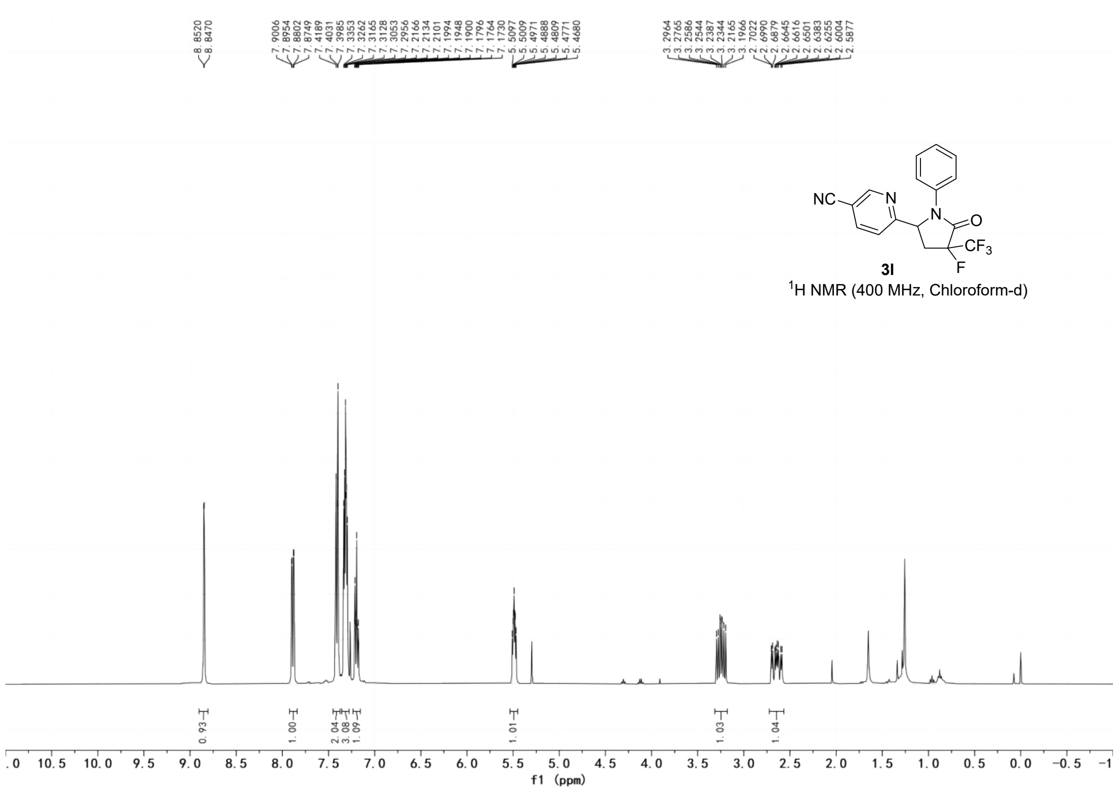


**3l**
$^{1}$H NMR (400 MHz, Chloroform-d)

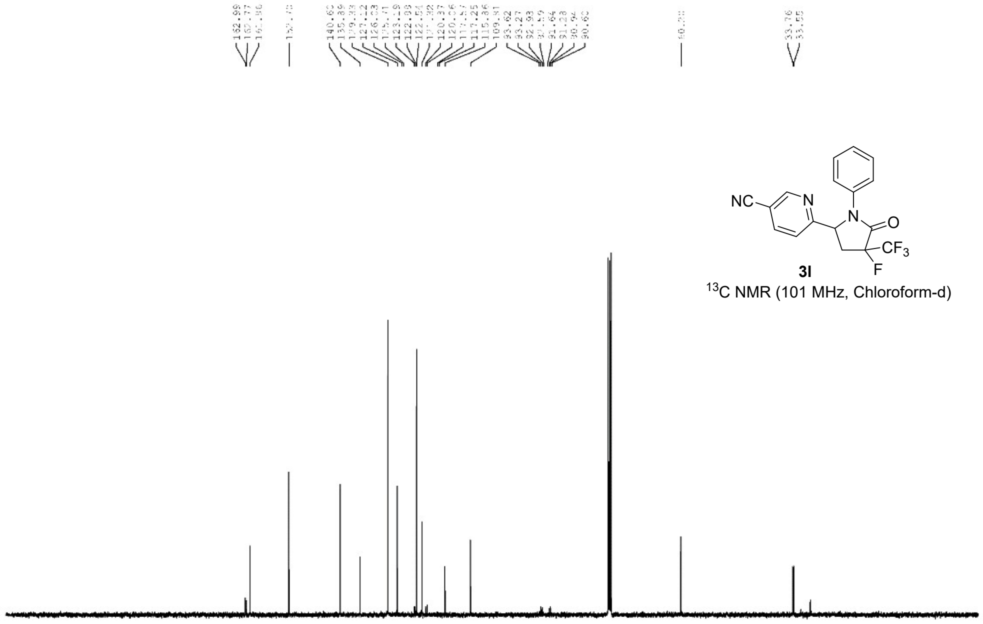


**3l**
$^{13}$C NMR (101 MHz, Chloroform-d)

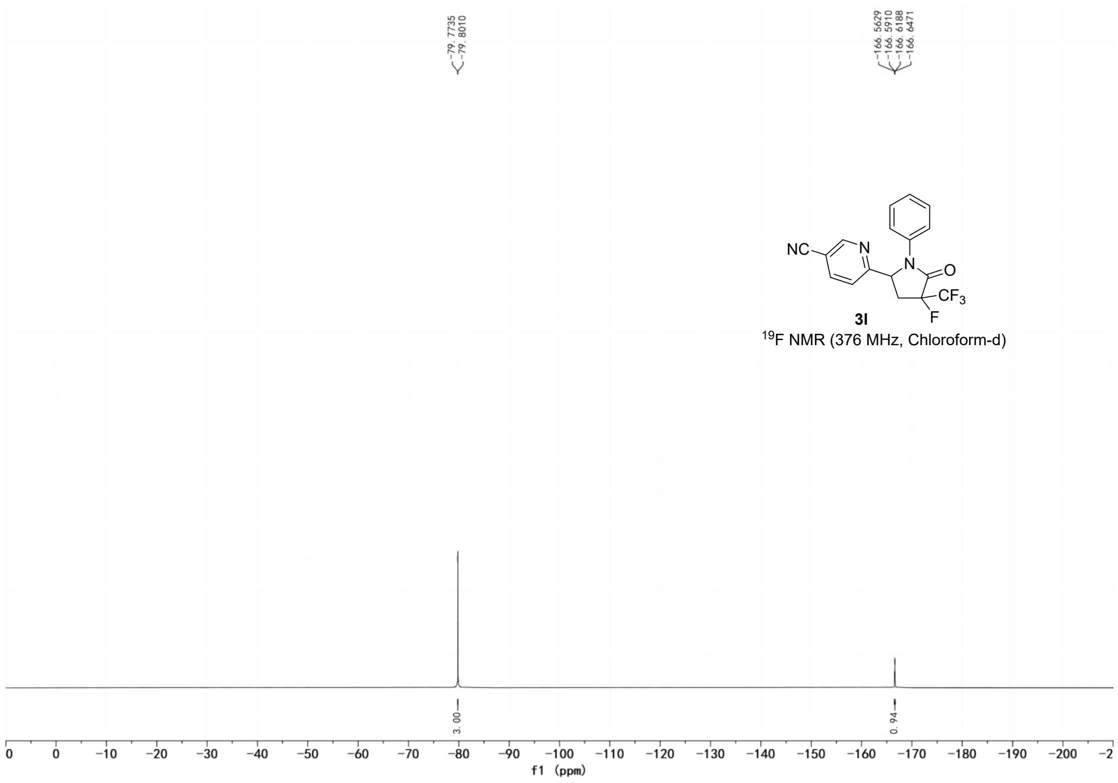
-79.7735
-79.8010
-166.5629
-166.5910
-166.6188
-166.6471
NC
N
N
O
CF3
F
3l
19F NMR (376 MHz, Chloroform-d)
3.00
0.94
f1 (ppm)

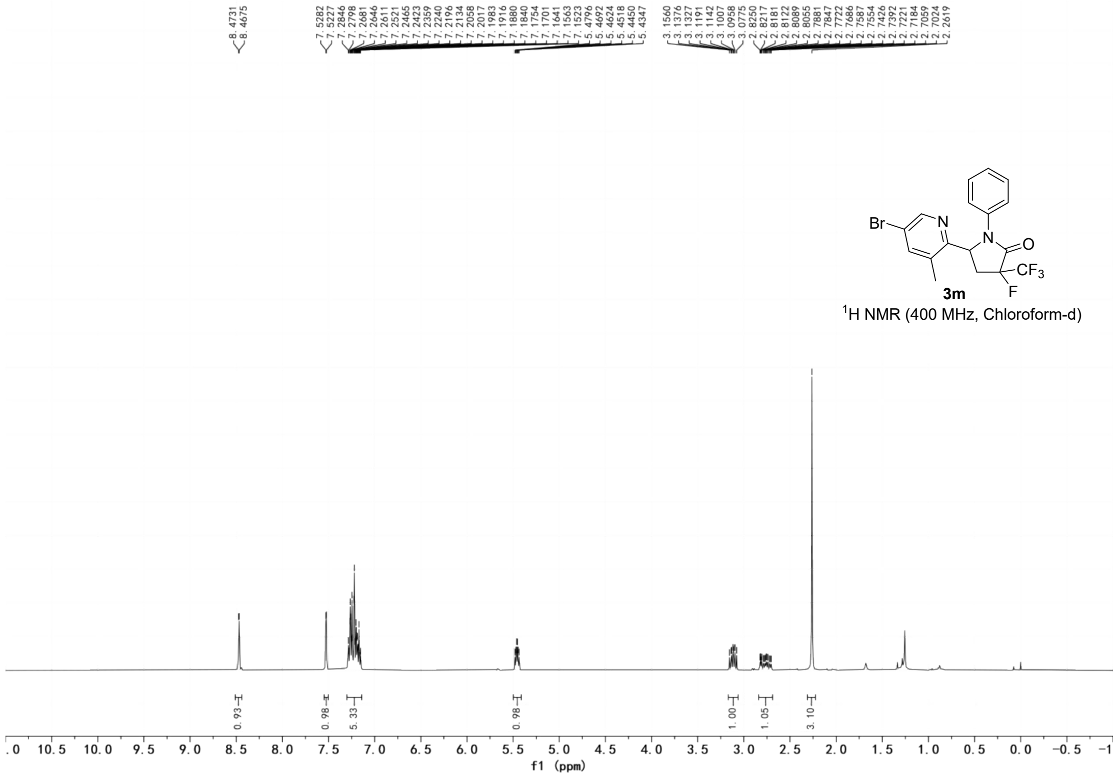
Br
N
O
CF3
F
3m
1H NMR (400 MHz, Chloroform-d)
f1 (ppm)

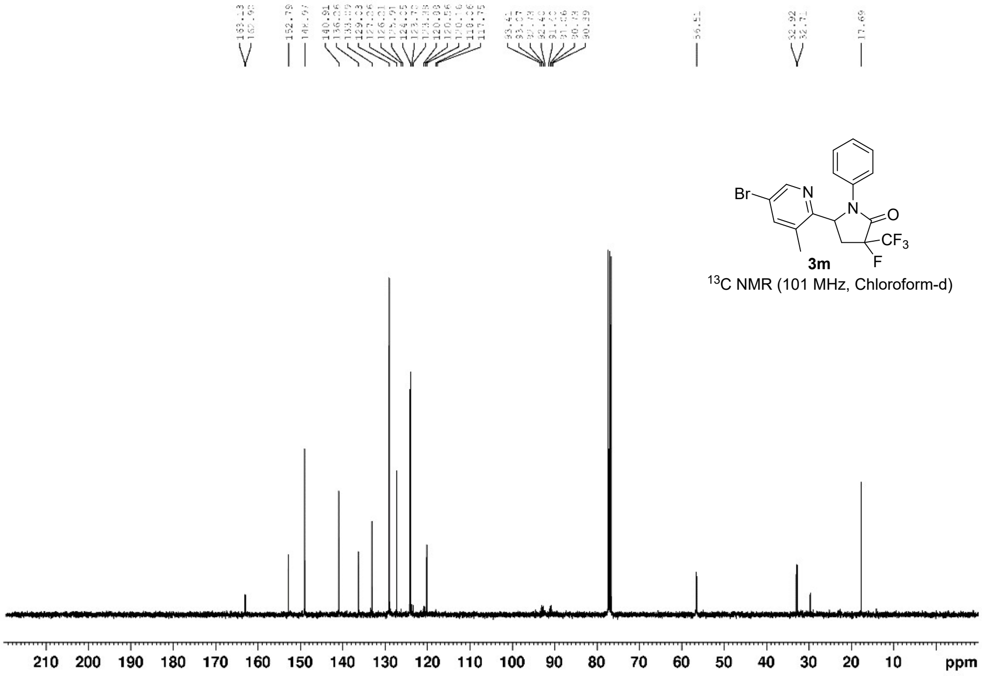
Br
N
O
CF3
F
3m
13C NMR (101 MHz, Chloroform-d)
ppm

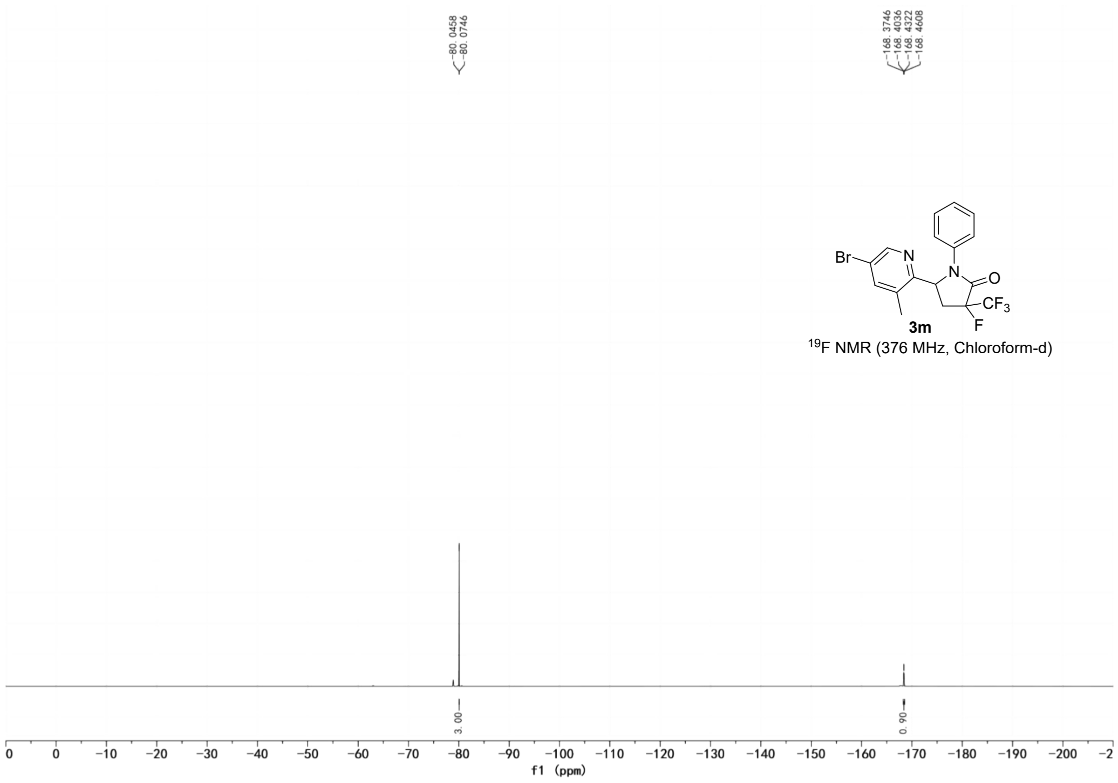


**3m**

$^{19}$F NMR (376 MHz, Chloroform-d)

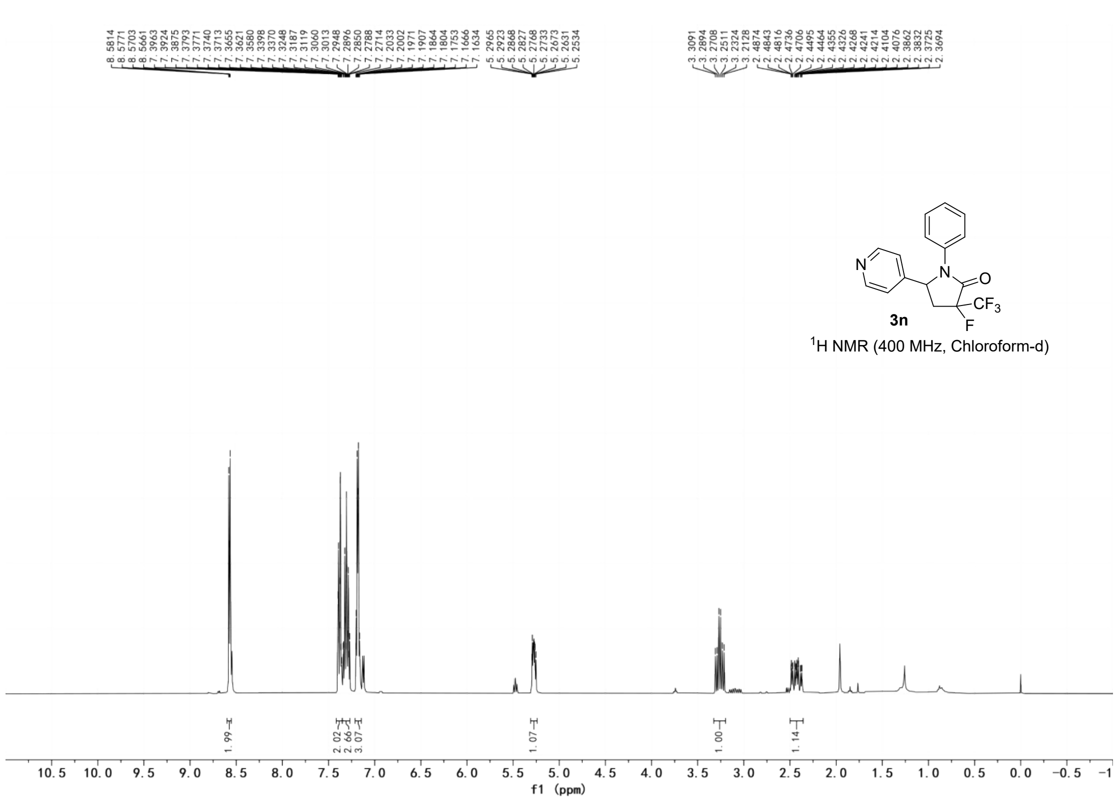
3n
$^{1}$H NMR (400 MHz, Chloroform-d)
f1 (ppm)

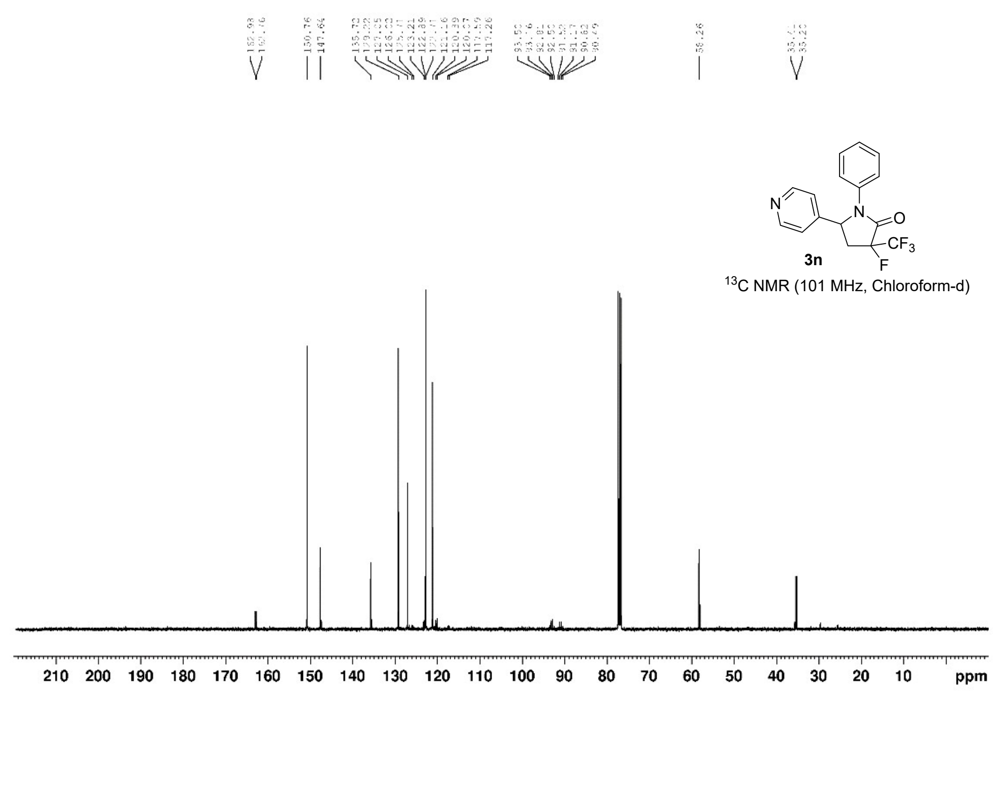
3n
$^{13}$C NMR (101 MHz, Chloroform-d)
ppm

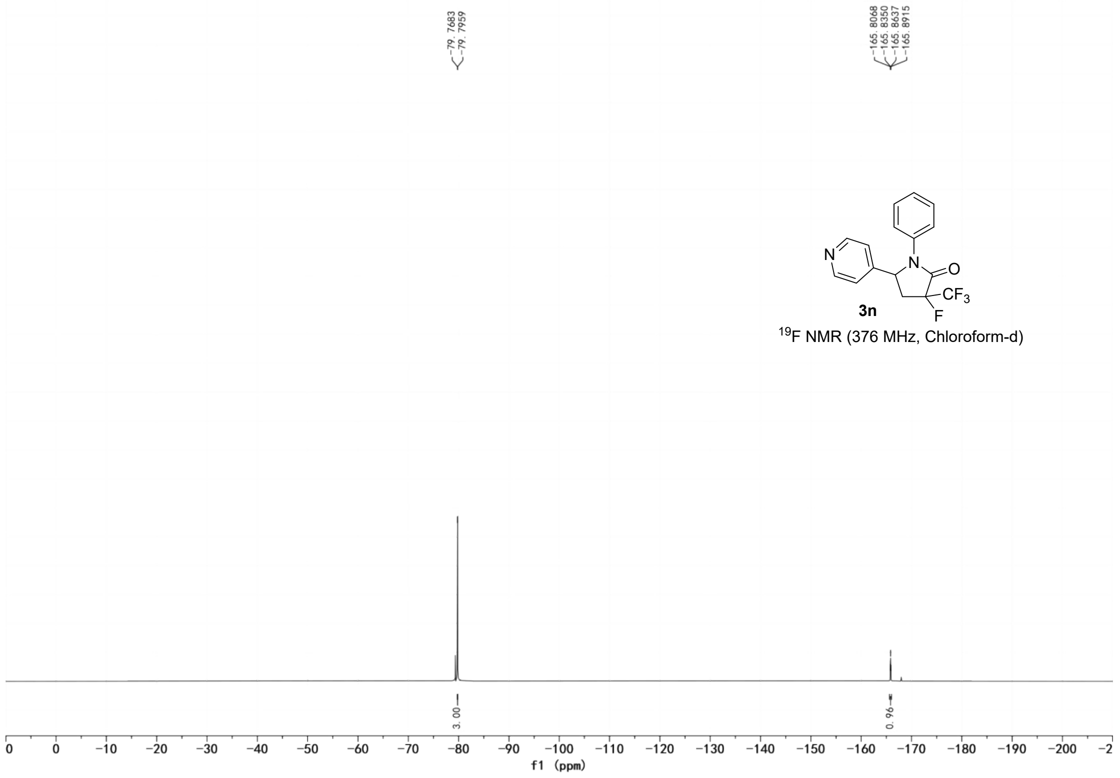

-79.7683
-79.7959
-165.8068
-165.8350
-165.8637
-165.8915
N
N
O
CF3
F
3n
19F NMR (376 MHz, Chloroform-d)
3.00
0.96
f1 (ppm)

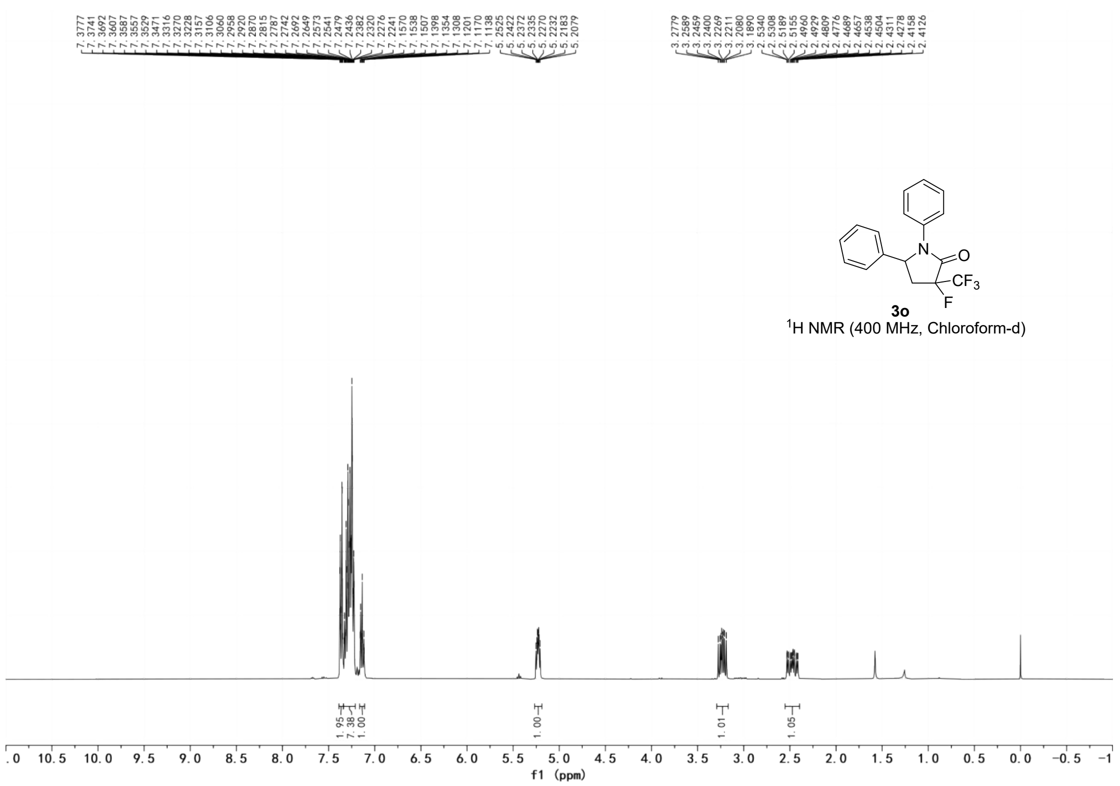
3o
1H NMR (400 MHz, Chloroform-d)
1.95
7.38
1.00
1.00
1.01
1.05
f1 (ppm)

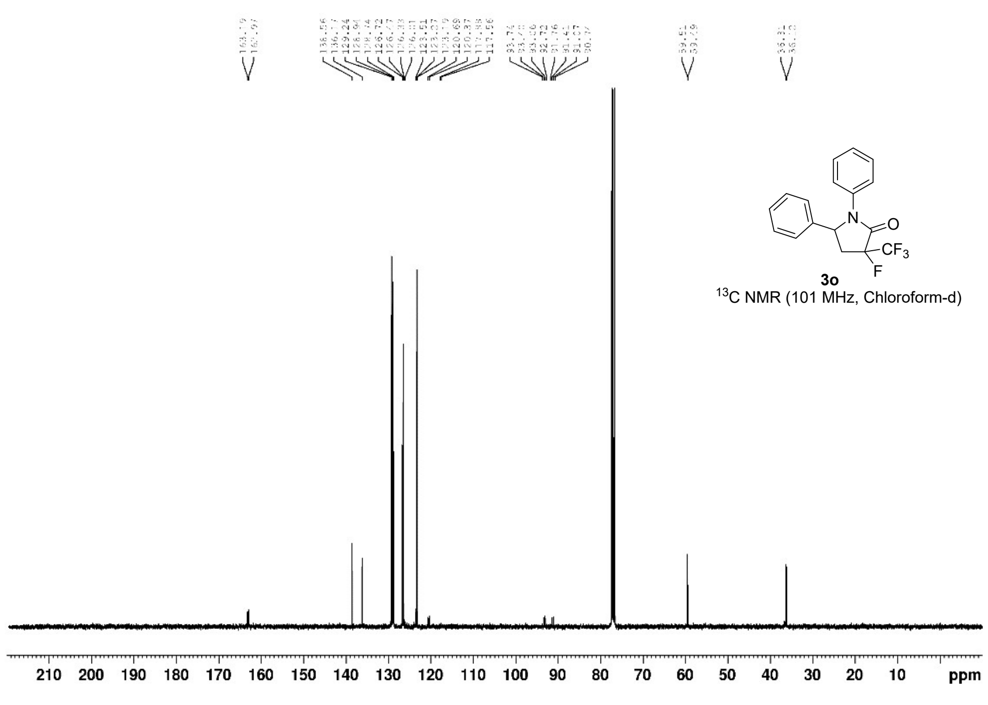
3o
13C NMR (101 MHz, Chloroform-d)
ppm

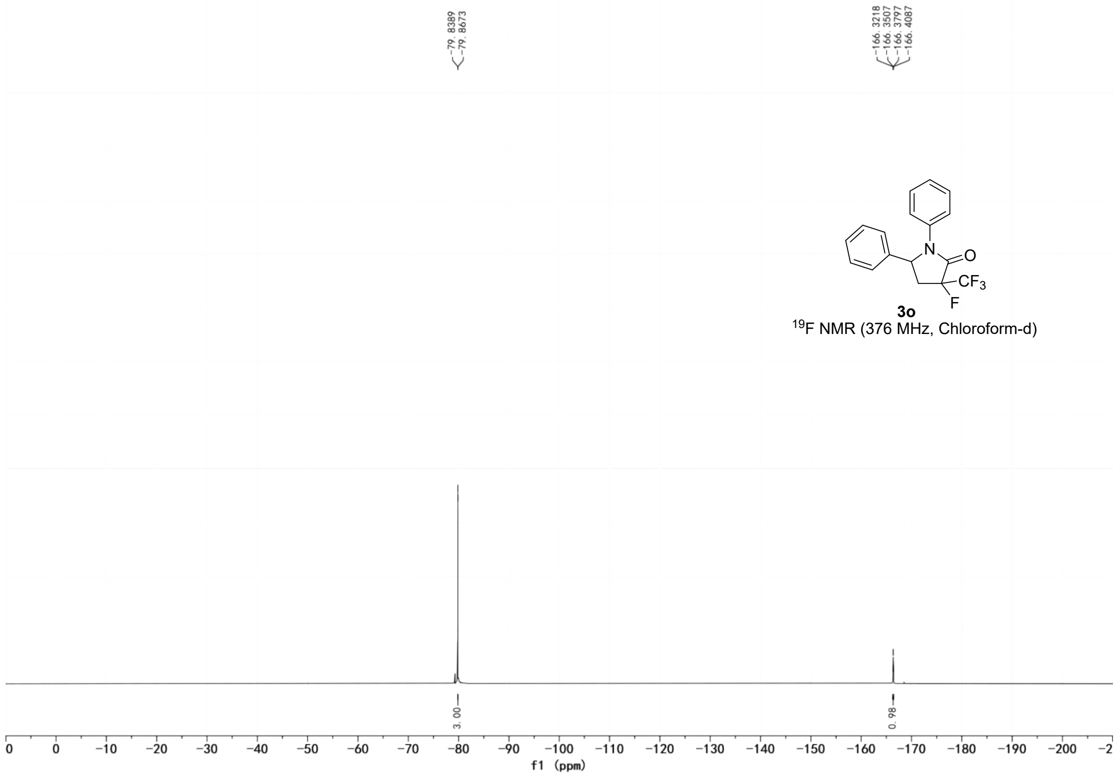
-79.8389
-79.8673
-166.3218
-166.3507
-166.3797
-166.4087
3o
$^{19}$F NMR (376 MHz, Chloroform-d)
3.00
0.98
f1 (ppm)

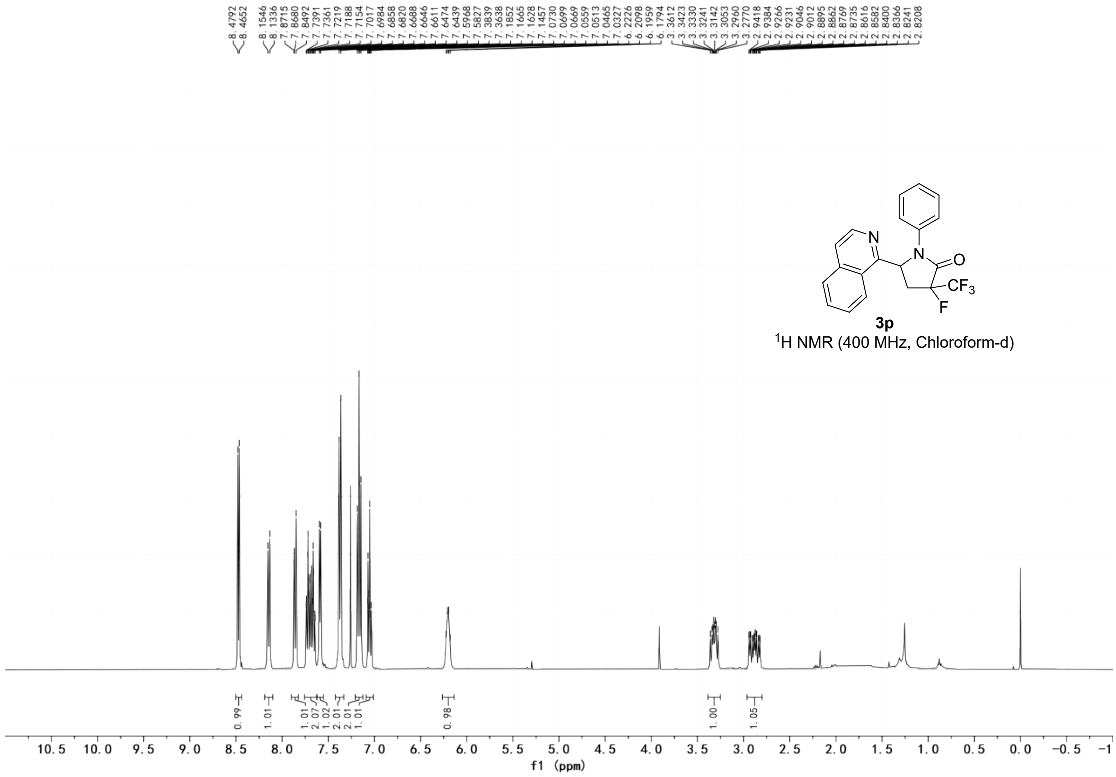
3p
$^1$H NMR (400 MHz, Chloroform-d)
f1 (ppm)

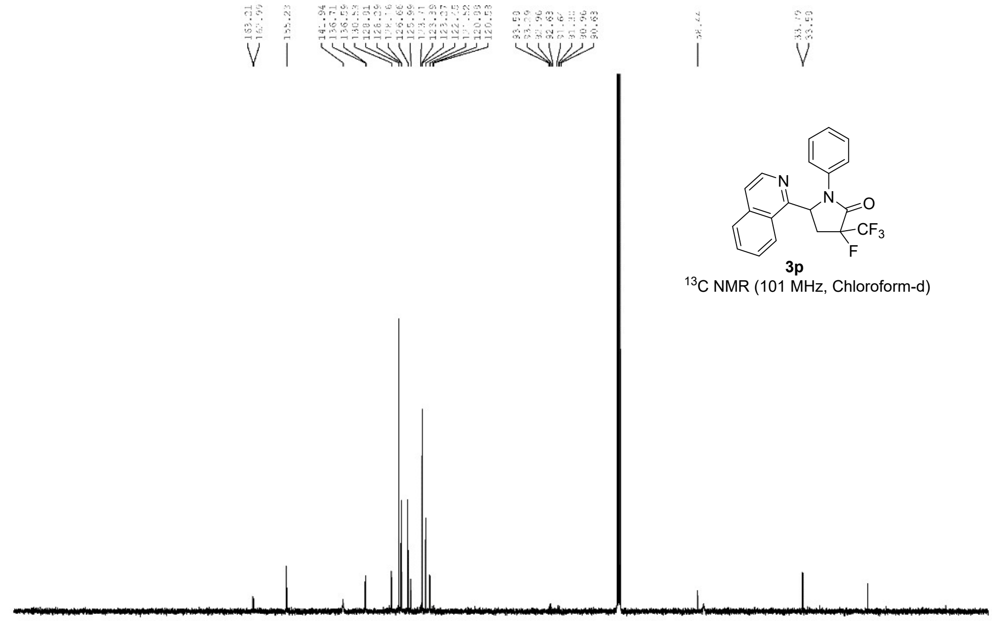
3p
$^{13}$C NMR (101 MHz, Chloroform-d)
ppm

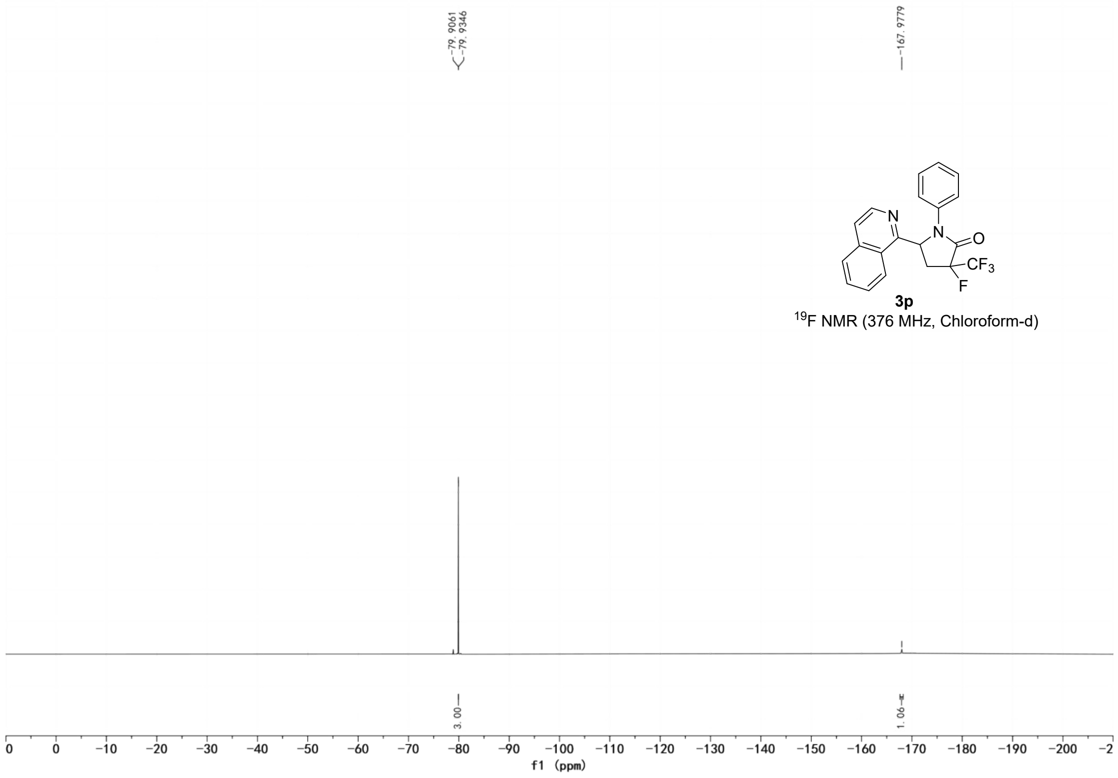


**3p**
$^{19}$F NMR (376 MHz, Chloroform-d)

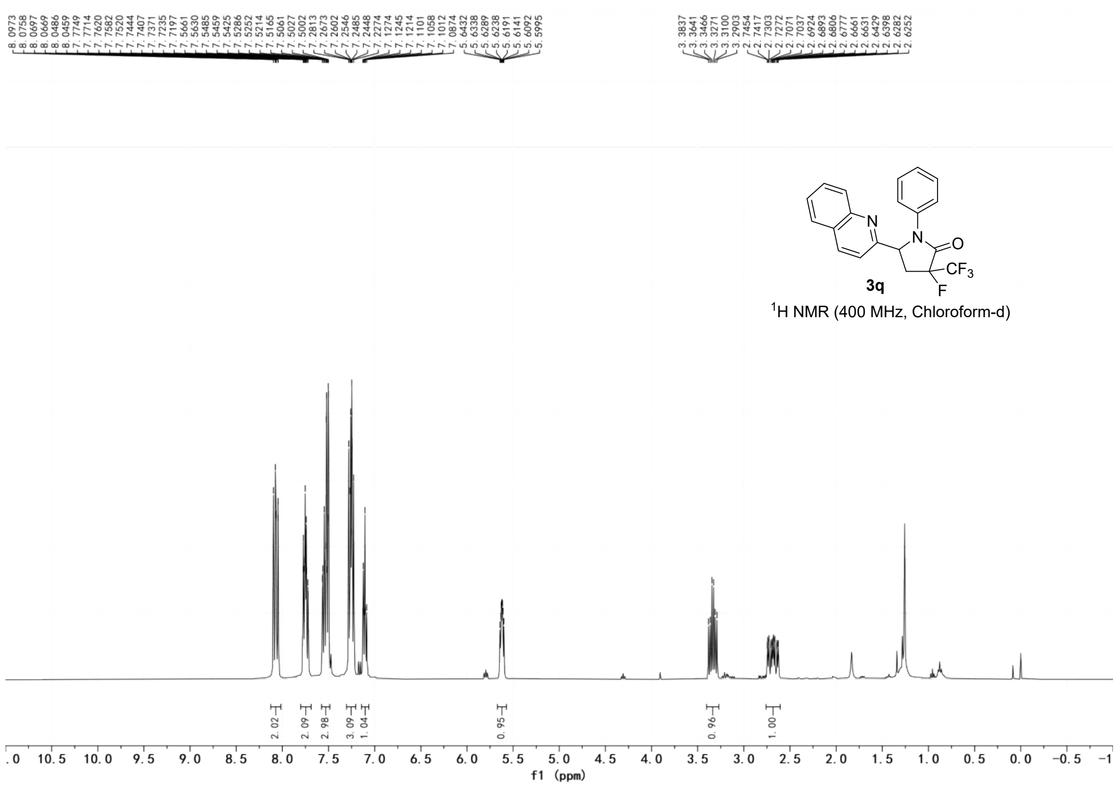

3q
$^{1}$H NMR (400 MHz, Chloroform-d)
f1 (ppm)


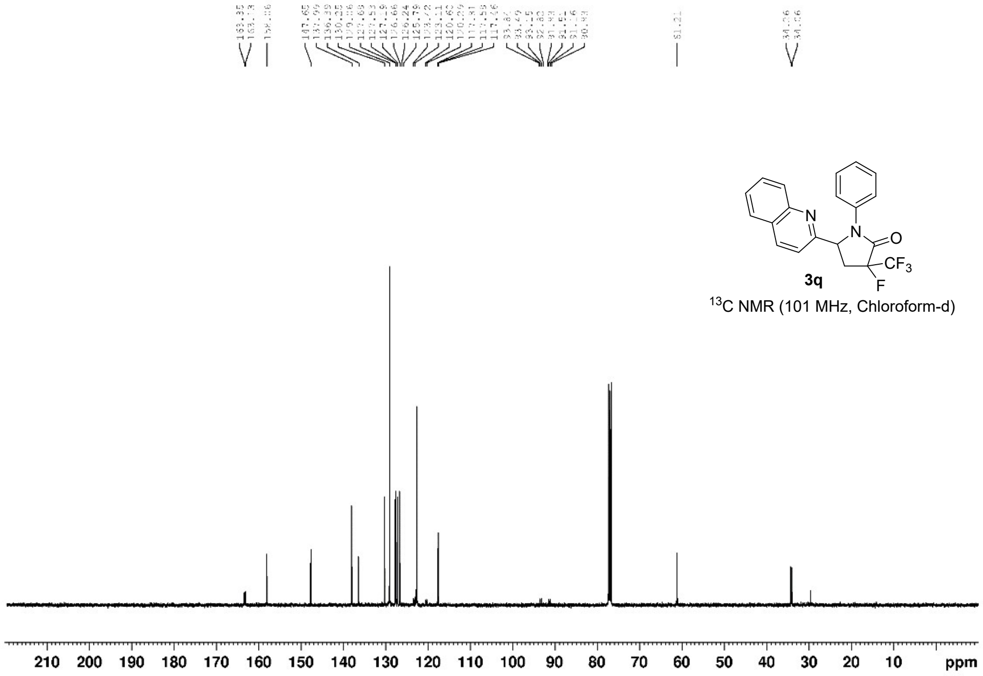

3q
$^{13}$C NMR (101 MHz, Chloroform-d)
ppm

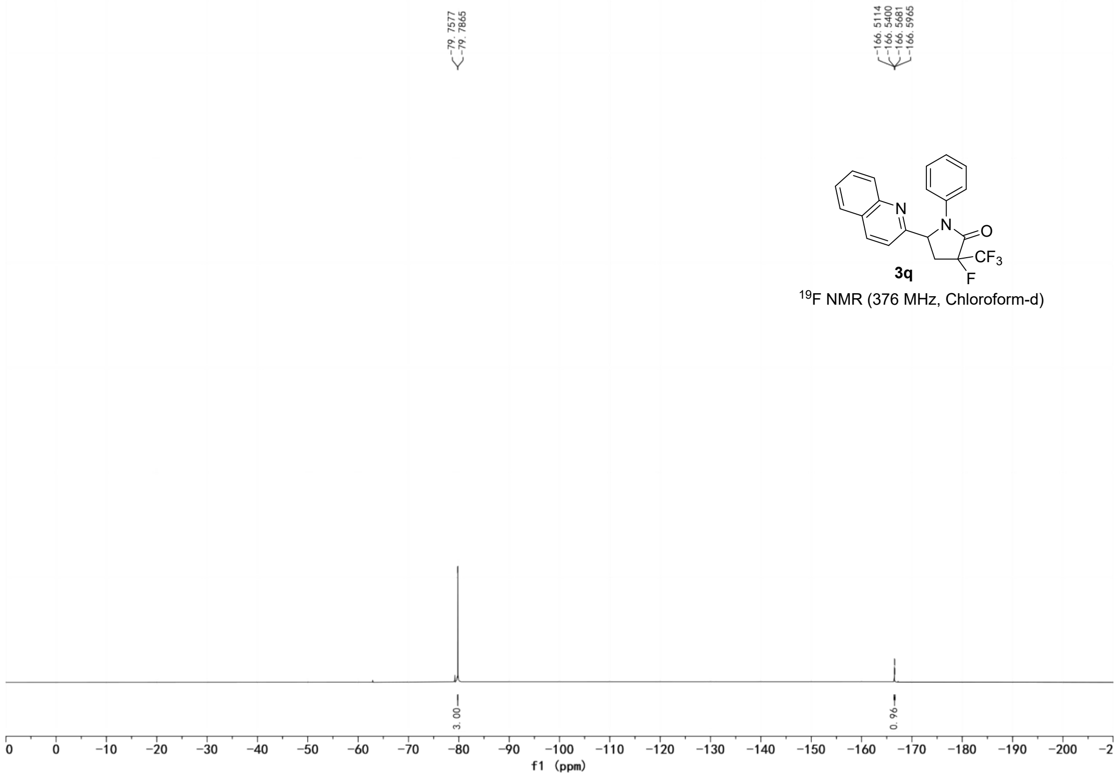
-79.7577
-79.7865
-166.5114
-166.5400
-166.5681
-166.5965
N
O
CF3
F
3q
19F NMR (376 MHz, Chloroform-d)
3.00
0.96
f1 (ppm)

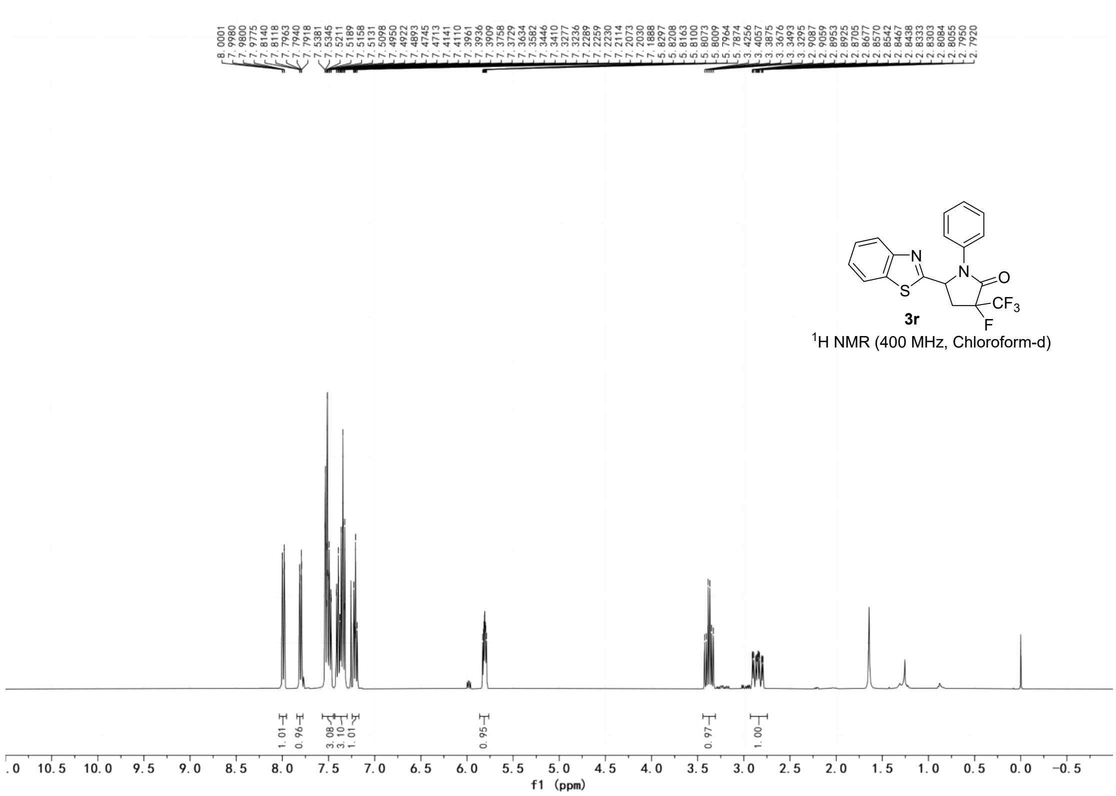
3r
¹H NMR (400 MHz, Chloroform-d)
f1 (ppm)

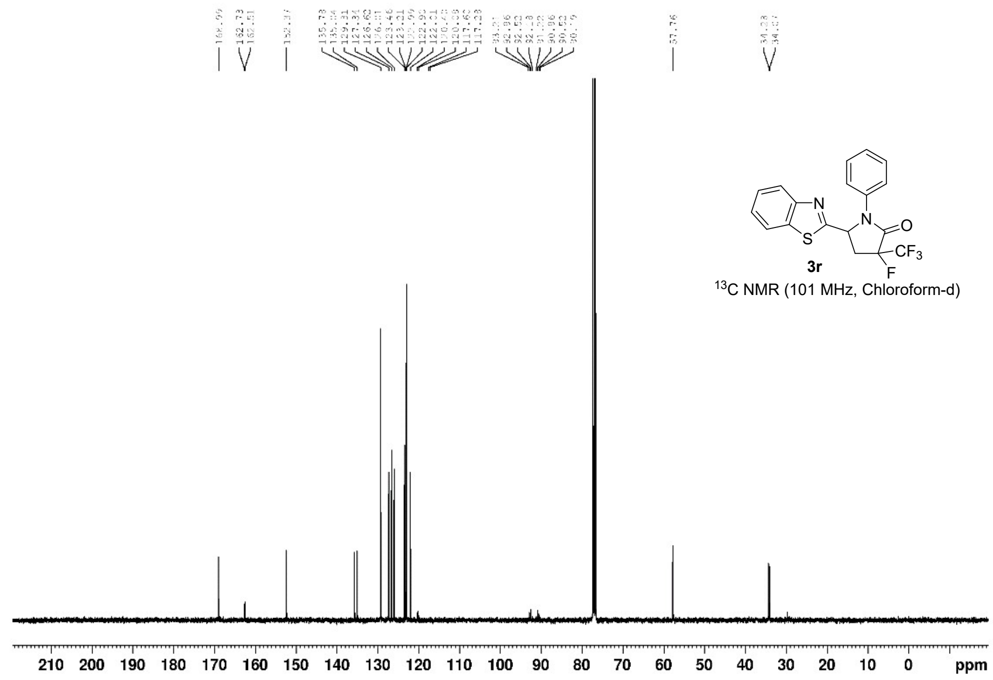
3r
¹³C NMR (101 MHz, Chloroform-d)
ppm

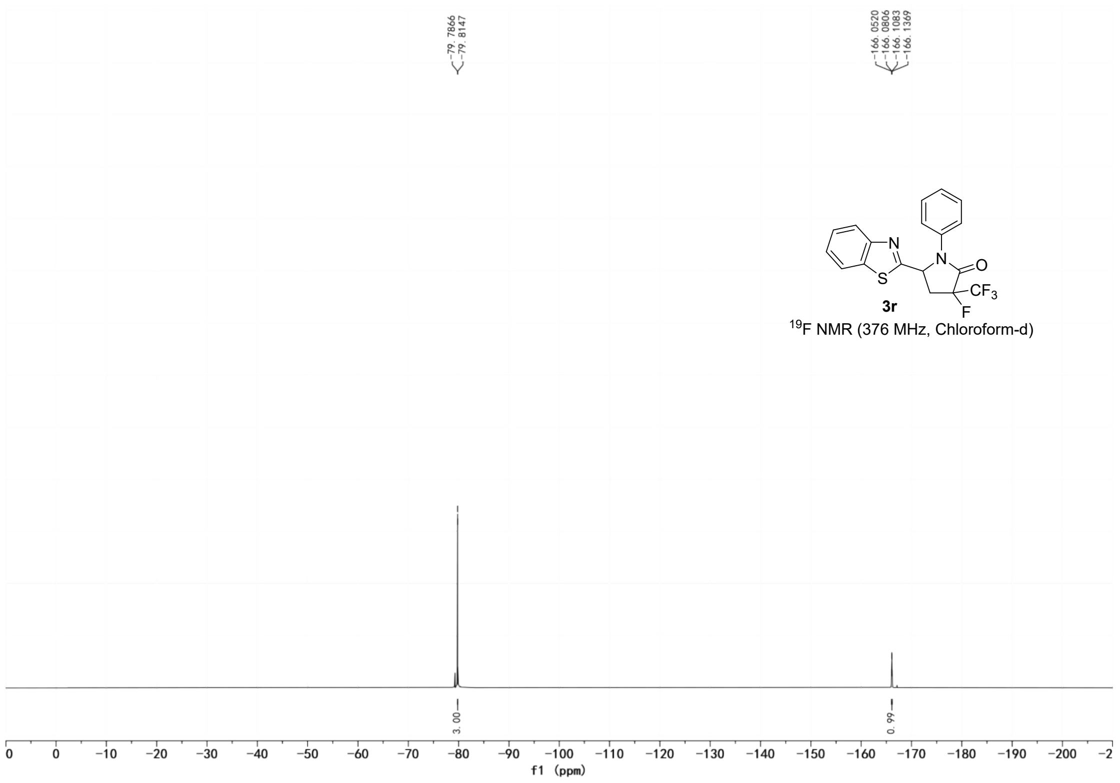
-79.7866
-79.8147
-166.0520
-166.0806
-166.1083
-166.1369
N
S
N
O
CF3
F
3r
19F NMR (376 MHz, Chloroform-d)
3.00
0.99
f1 (ppm)

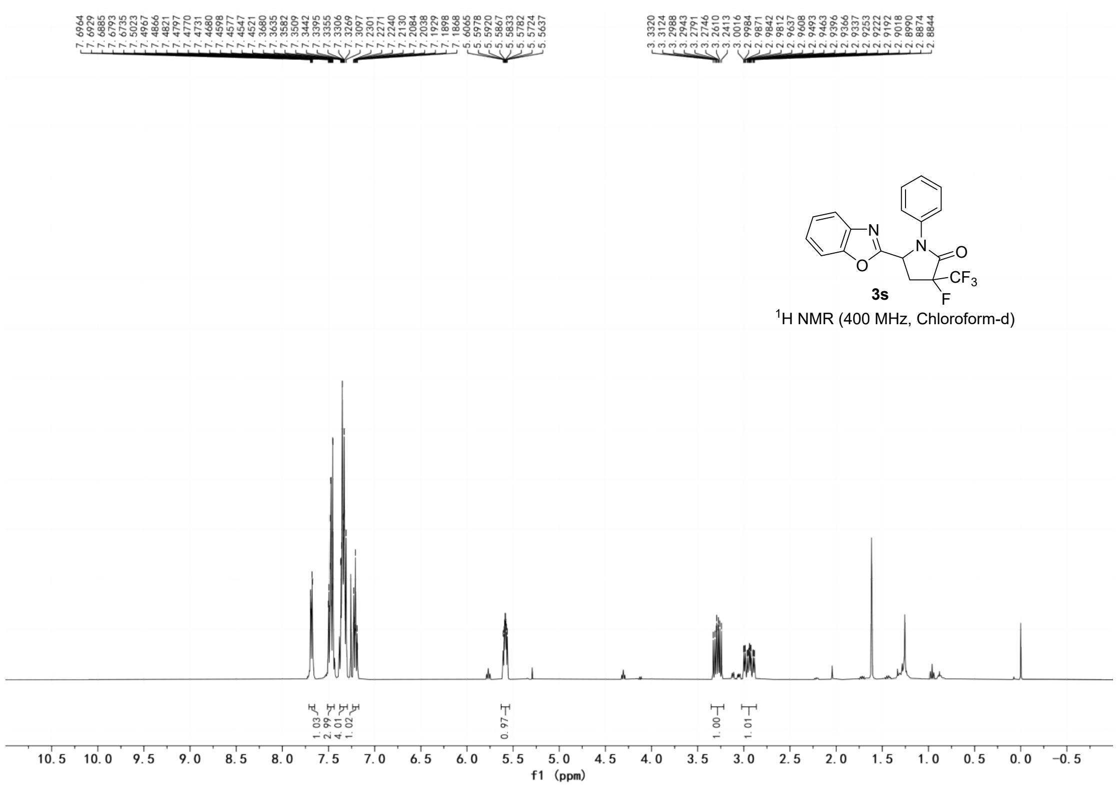
3s
1H NMR (400 MHz, Chloroform-d)
f1 (ppm)

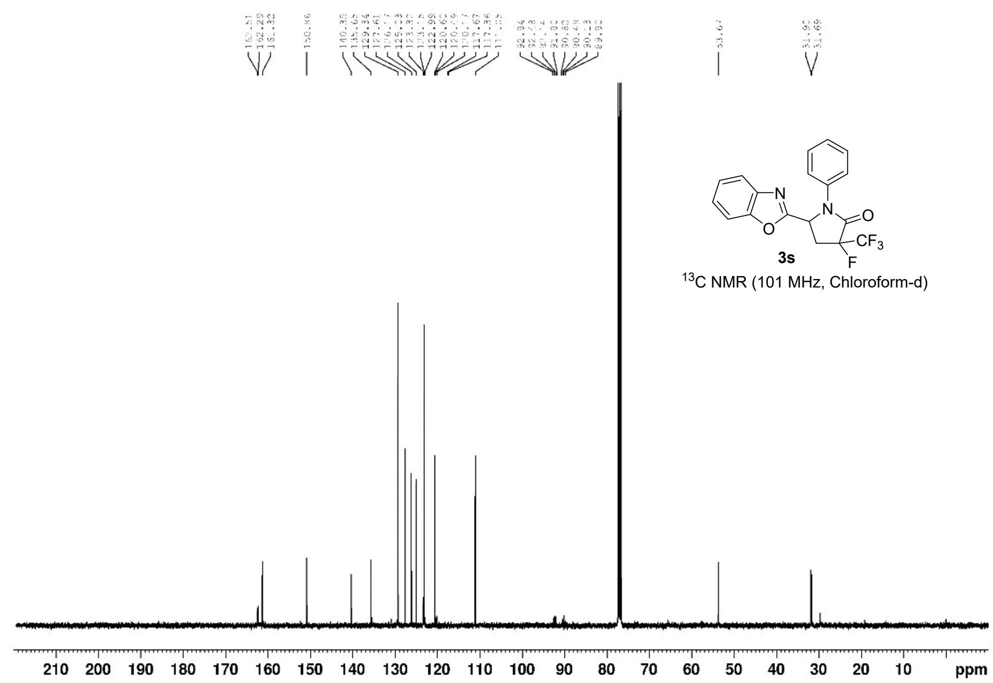
3s
13C NMR (101 MHz, Chloroform-d)
ppm

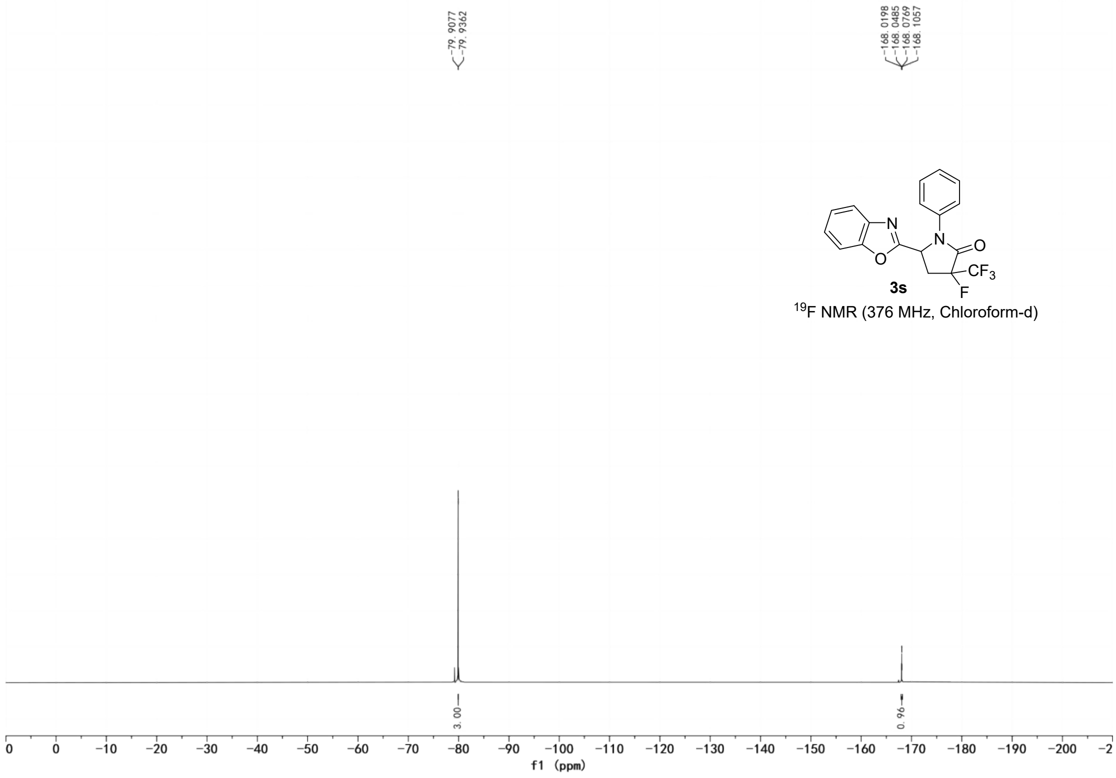

$^{19}$F NMR (376 MHz, Chloroform-d)

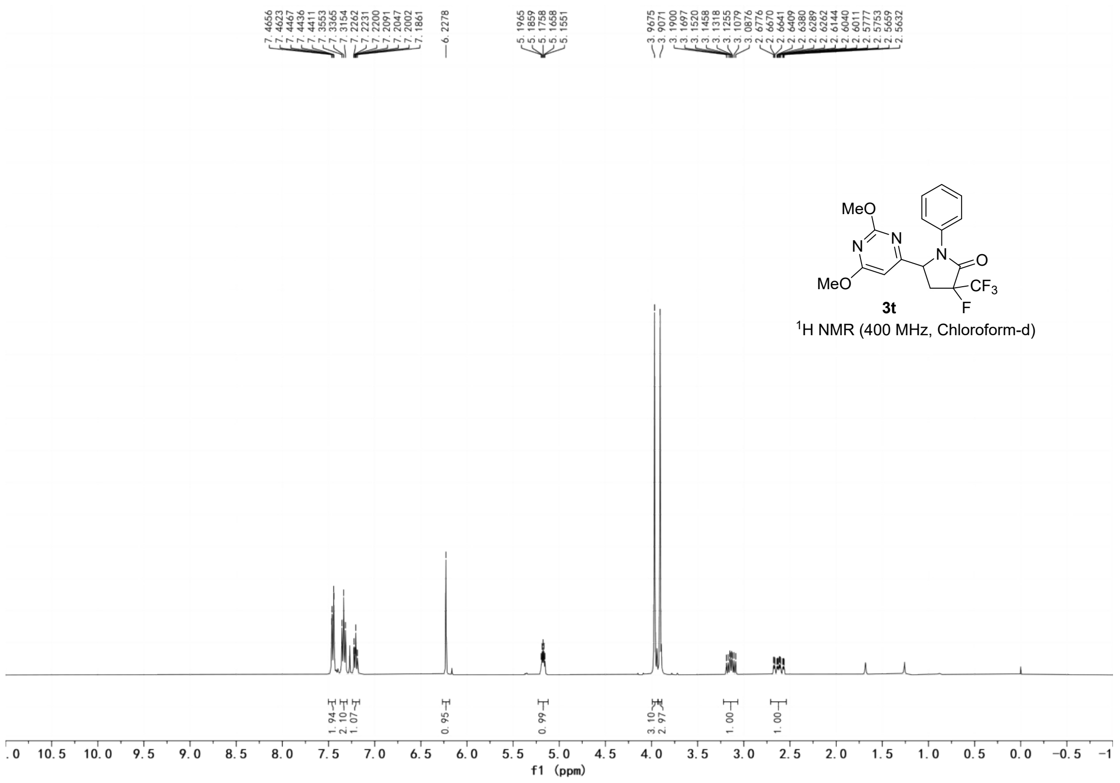
MeO
N
N
MeO
N
O
CF3
F
3t
1H NMR (400 MHz, Chloroform-d)
f1 (ppm)

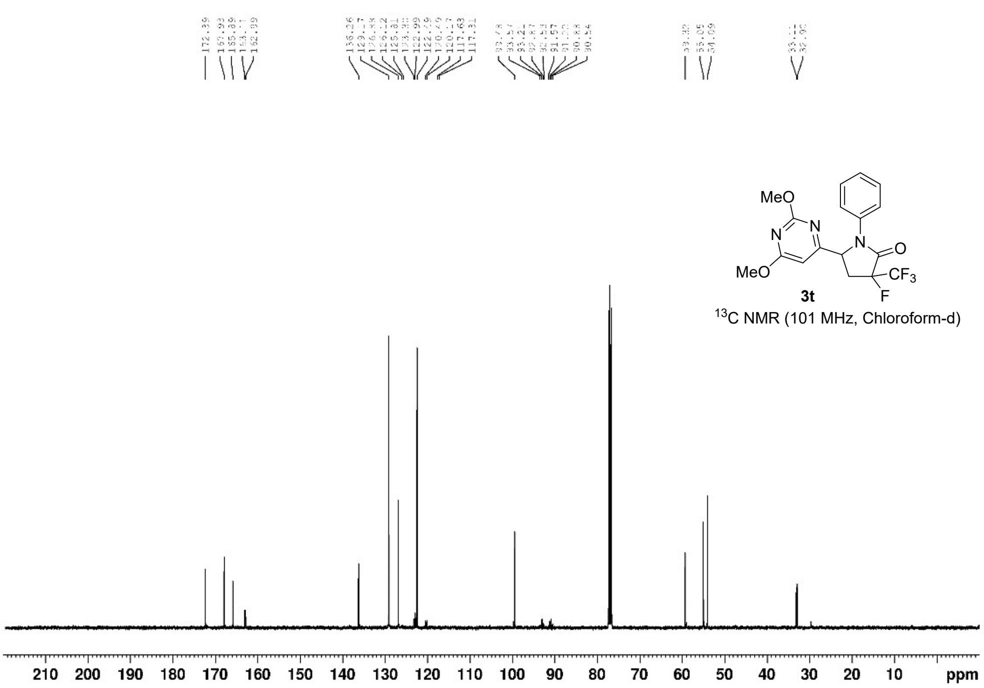
MeO
N
N
MeO
N
O
CF3
F
3t
13C NMR (101 MHz, Chloroform-d)
ppm

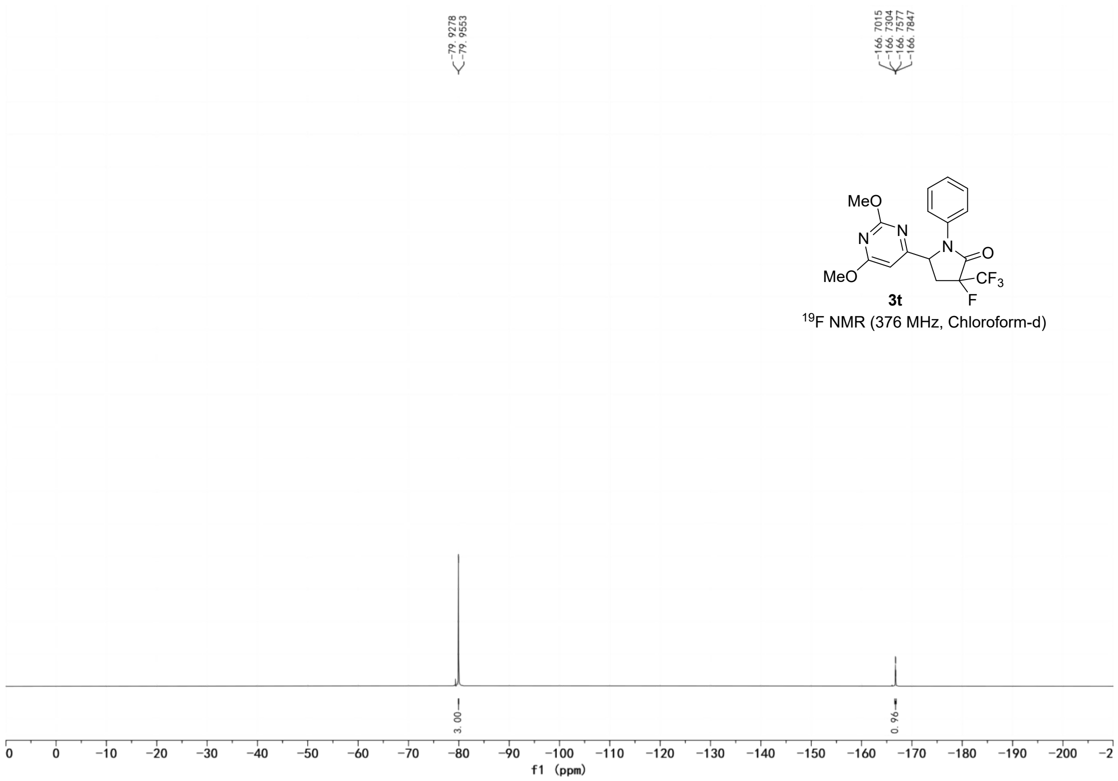


**3t**

$^{19}$F NMR (376 MHz, Chloroform-d)

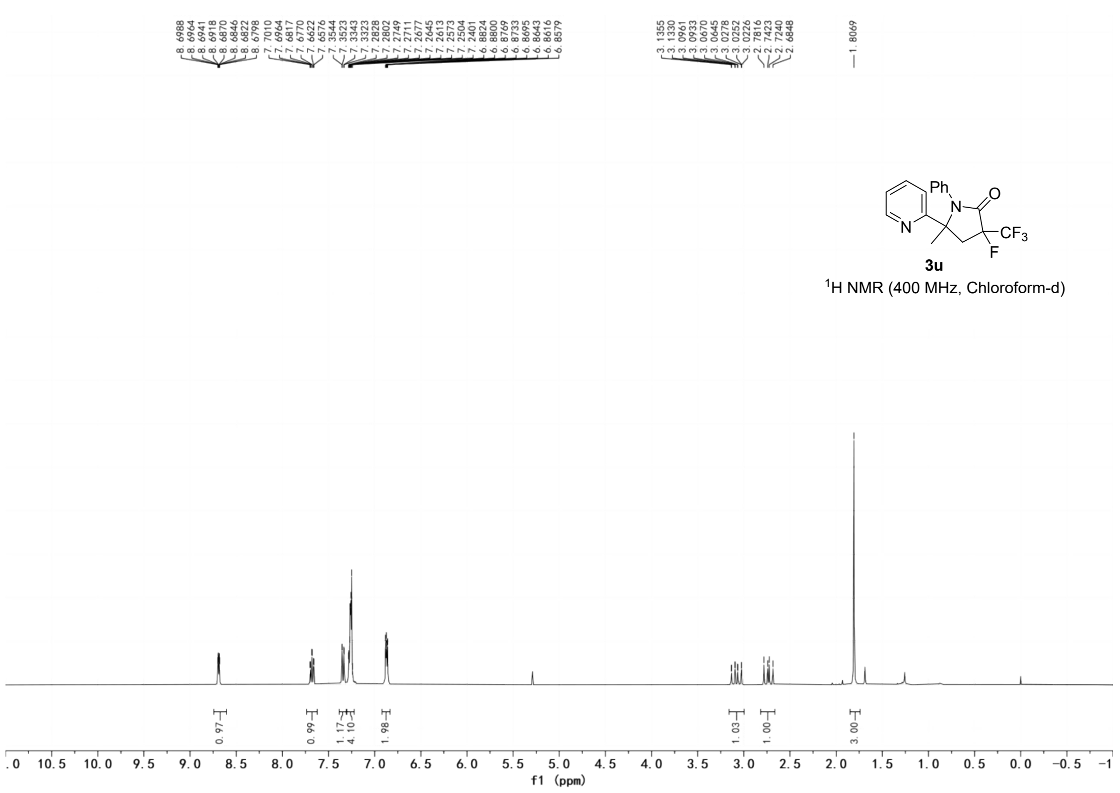

3u
$^1$H NMR (400 MHz, Chloroform-d)

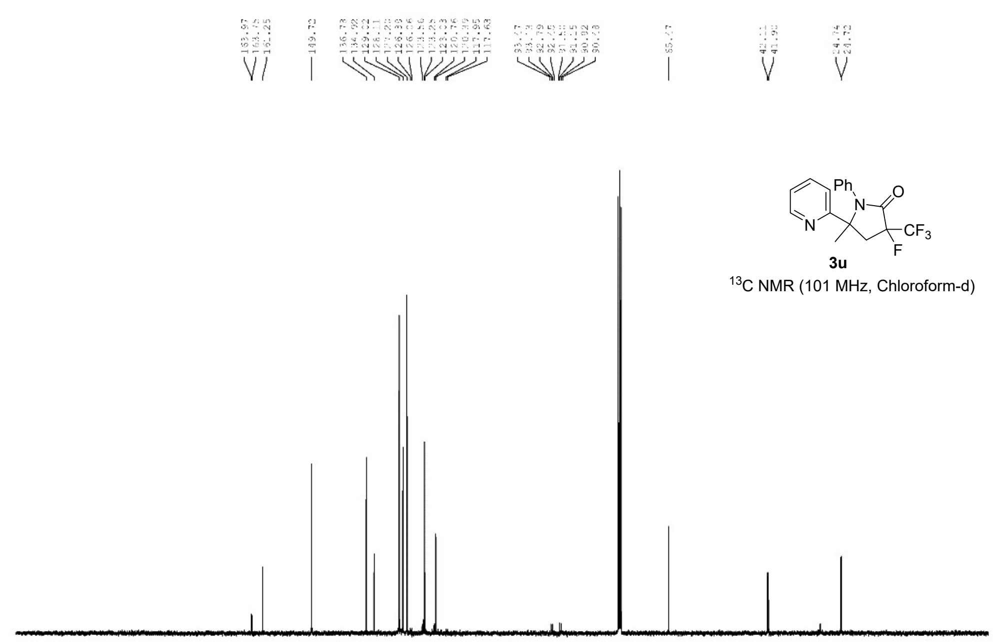

3u
$^{13}$C NMR (101 MHz, Chloroform-d)

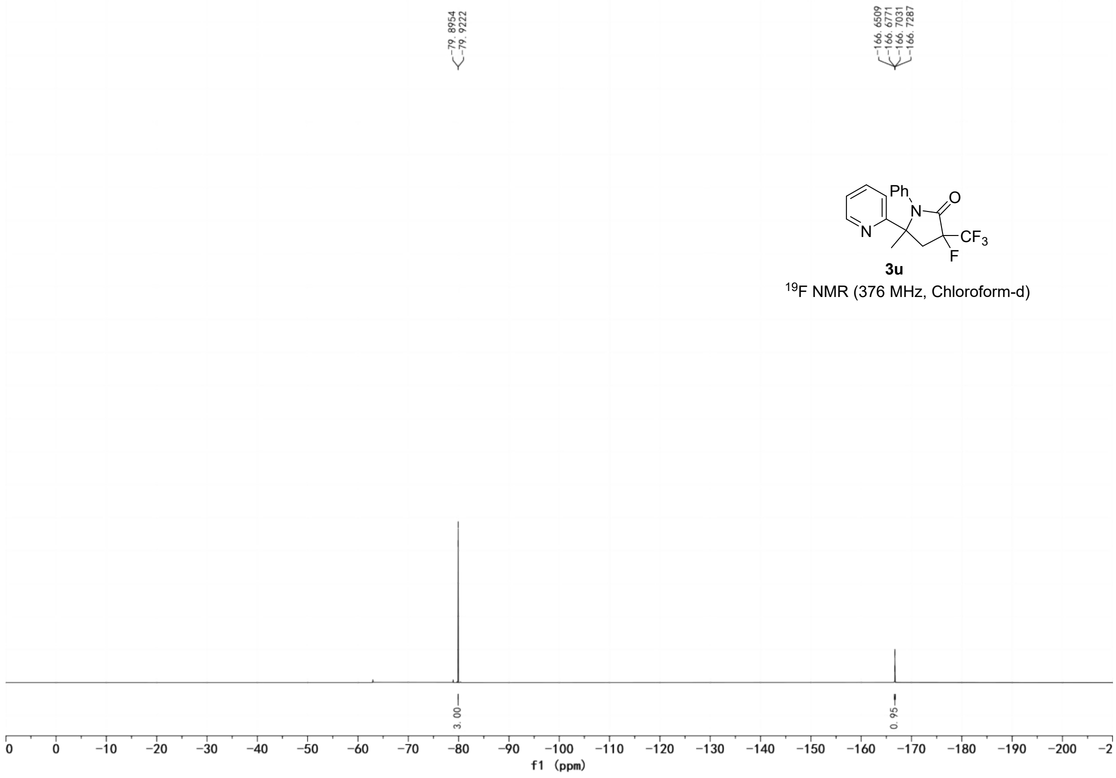


**3u**

$^{19}$F NMR (376 MHz, Chloroform-d)

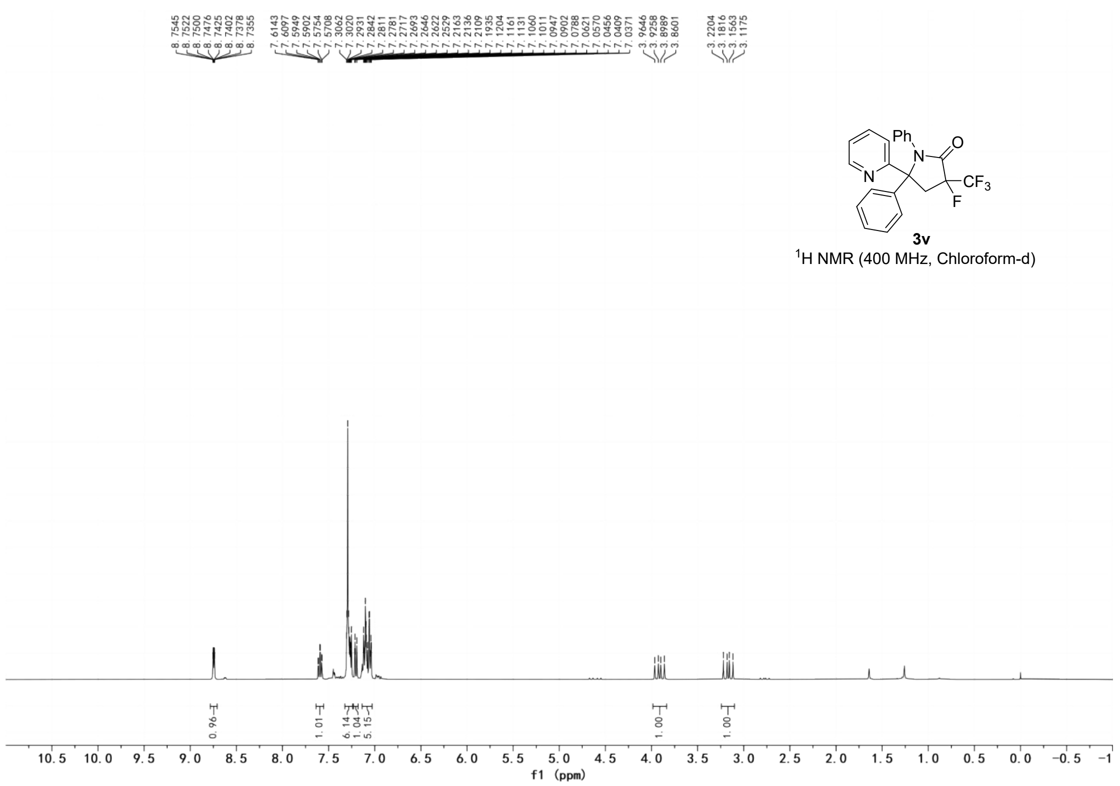

**3v**
$^{1}$H NMR (400 MHz, Chloroform-d)

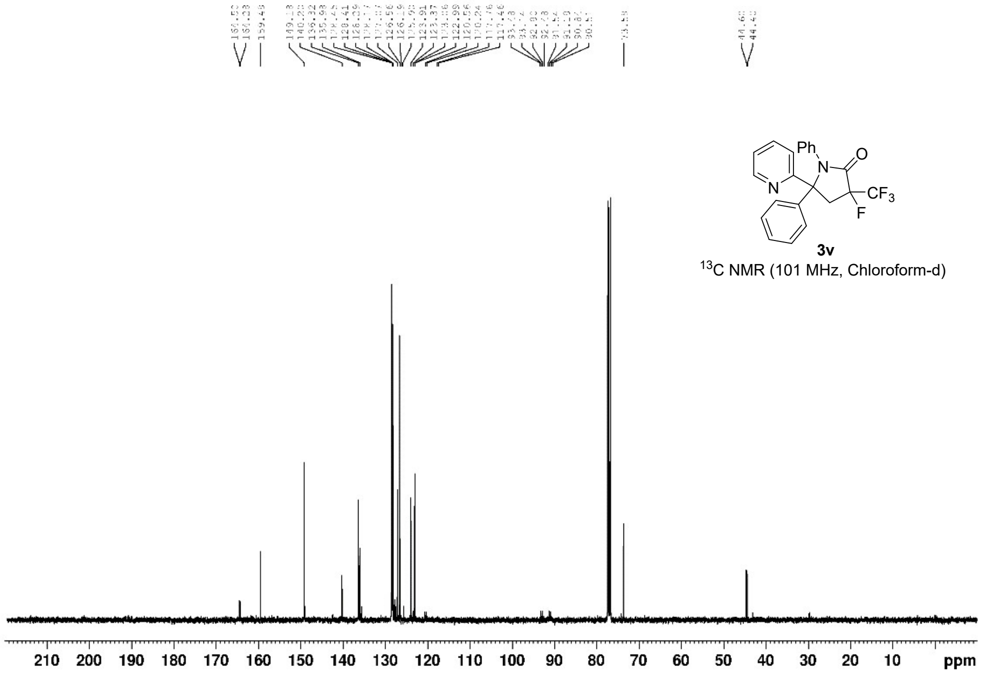

**3v**
$^{13}$C NMR (101 MHz, Chloroform-d)

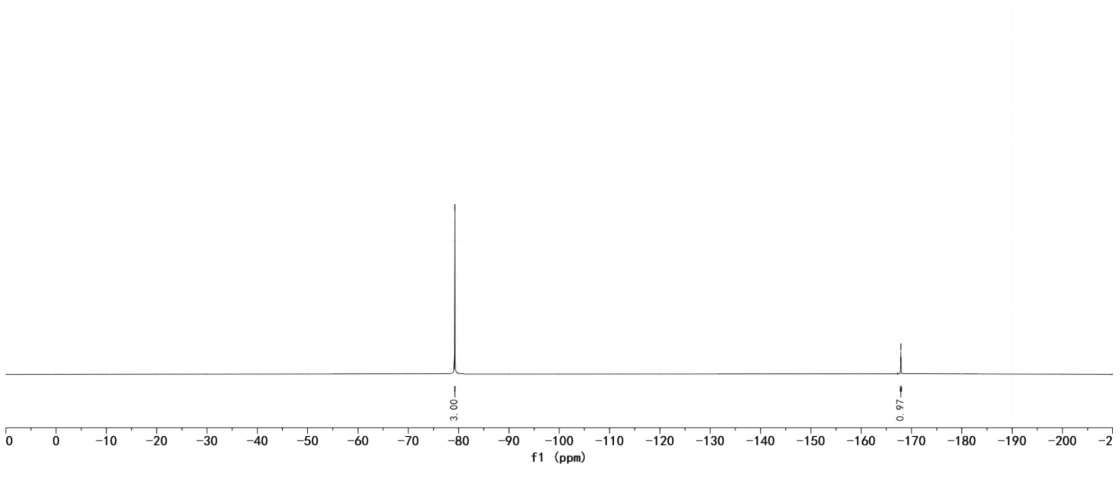


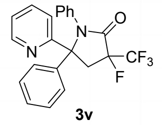


**3v**

$^{19}F$ NMR (376 MHz, Chloroform-d)

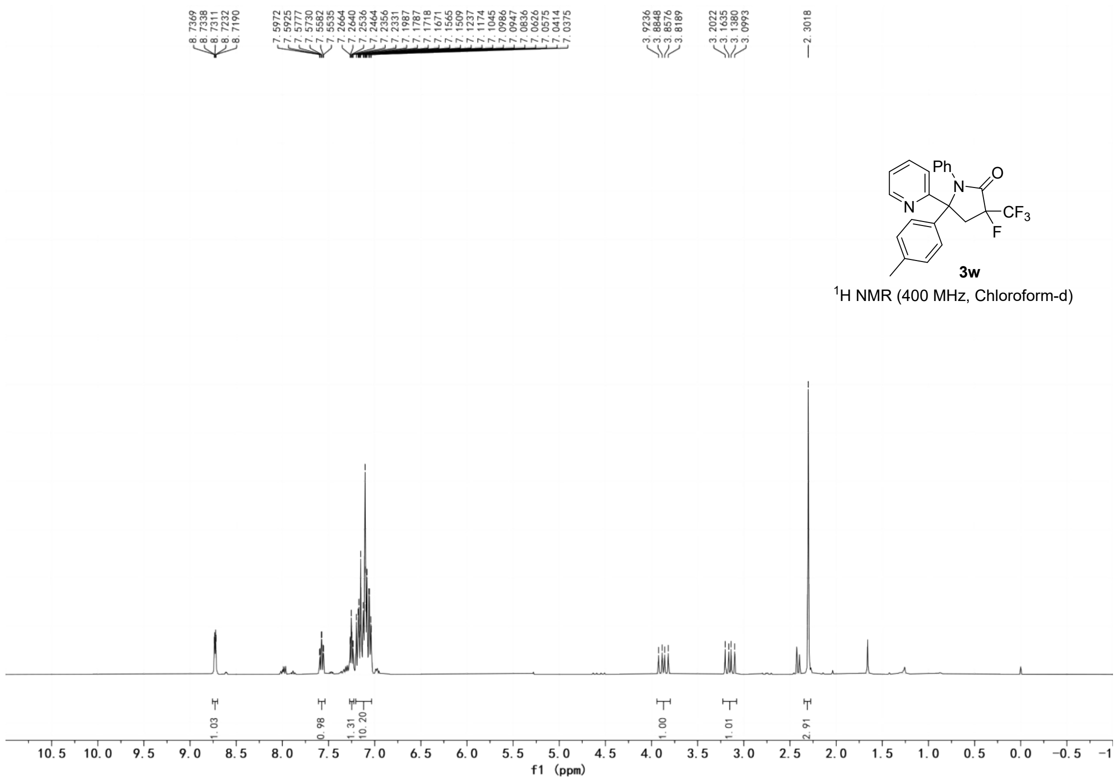
Ph
N
O
CF3
F
3w
1H NMR (400 MHz, Chloroform-d)
f1 (ppm)

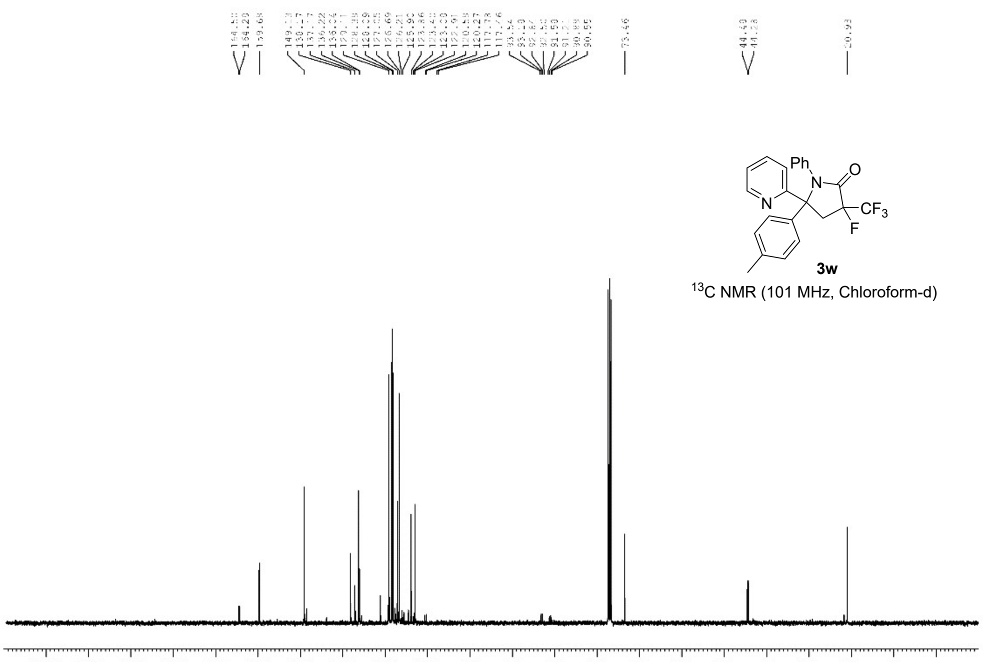
Ph
N
O
CF3
F
3w
13C NMR (101 MHz, Chloroform-d)
ppm

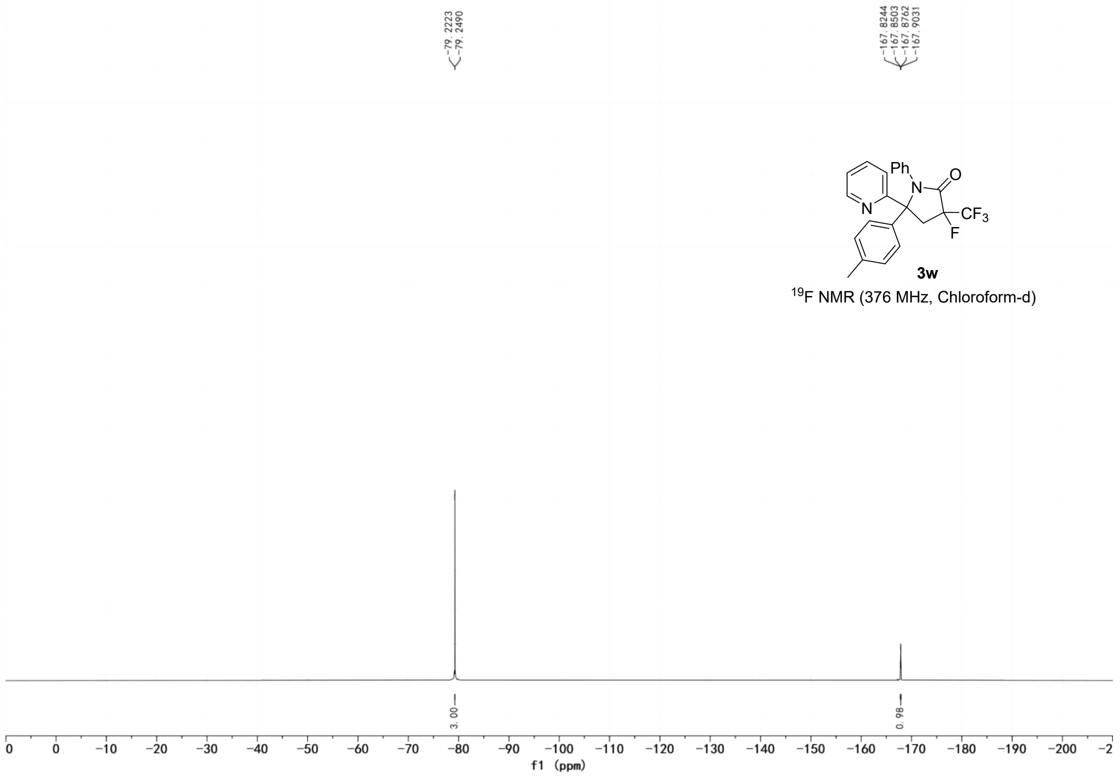

-79.2223
-79.2490
-167.8244
-167.8503
-167.8762
-167.9031
Ph
N
O
CF3
F
N
3w
19F NMR (376 MHz, Chloroform-d)
3.00
0.98
f1 (ppm)

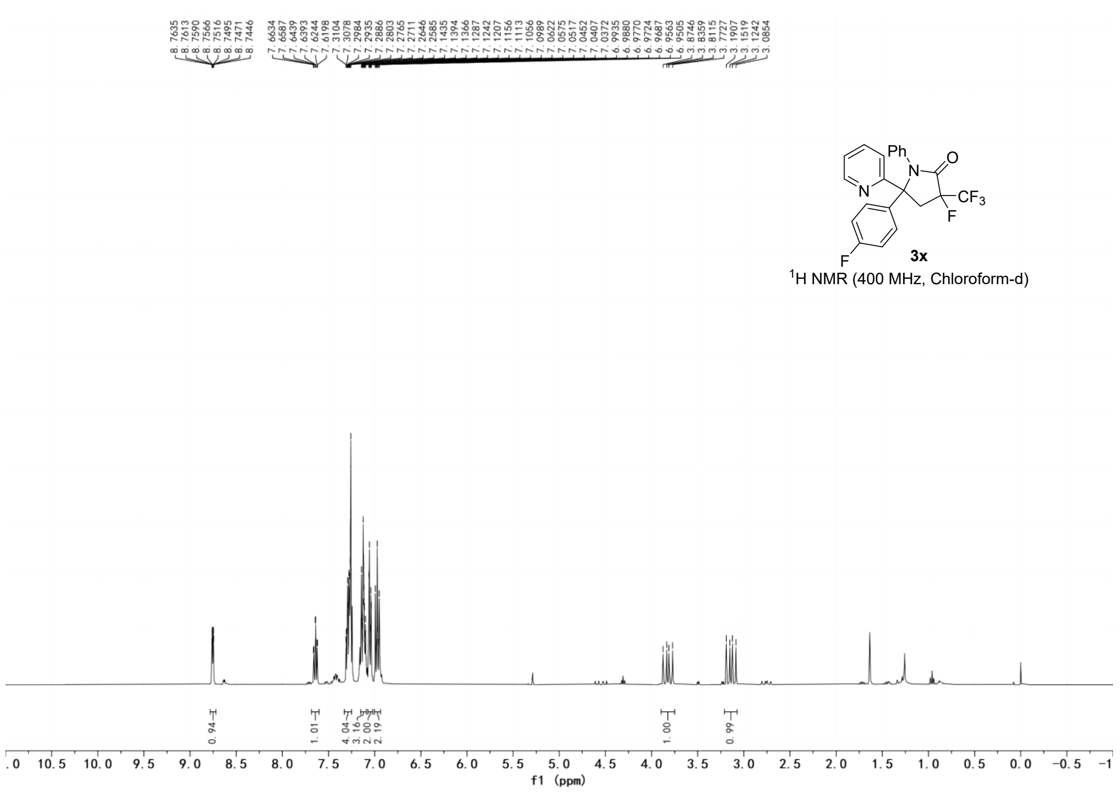
Ph
N
O
N
CF3
F
F
3x
1H NMR (400 MHz, Chloroform-d)
f1 (ppm)

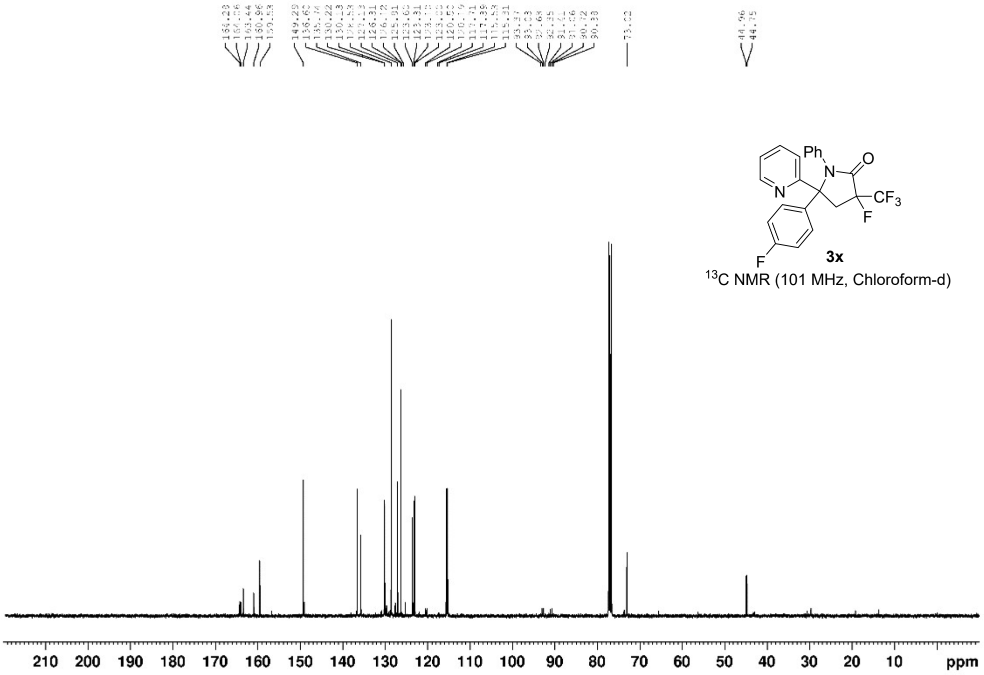
Ph
N
O
N
CF3
F
F
3x
13C NMR (101 MHz, Chloroform-d)
ppm

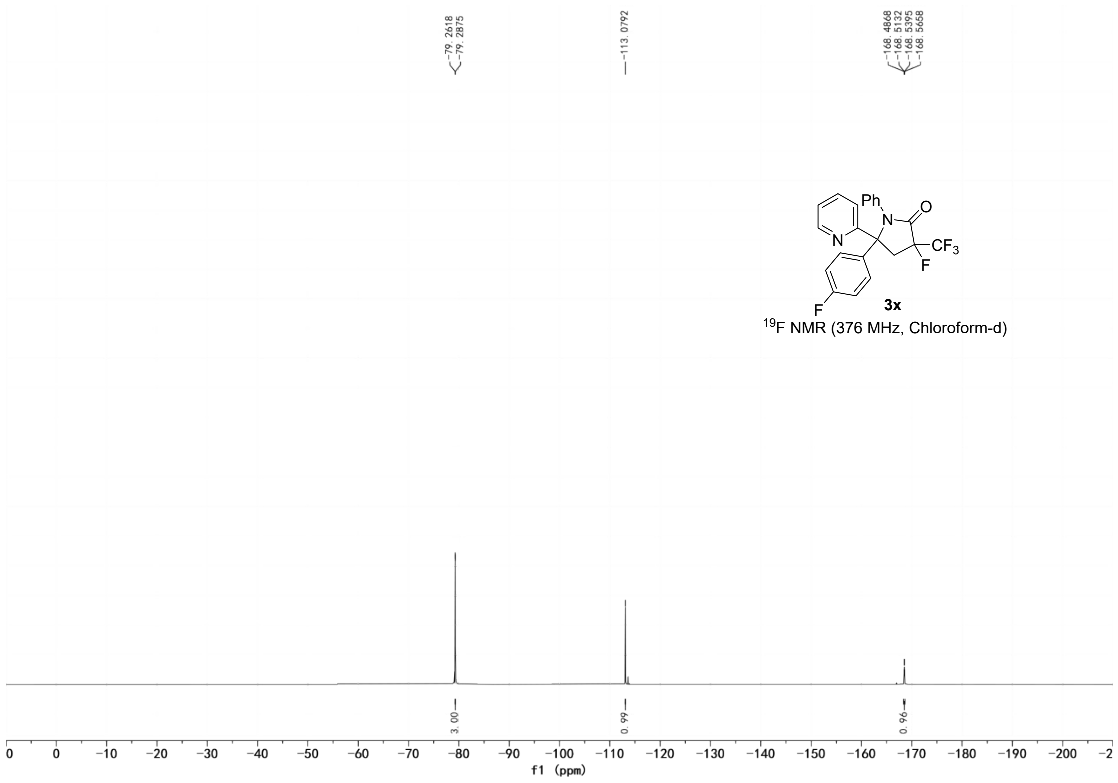
-79.2618
-79.2875
-113.0792
-168.4868
-168.5132
-168.5395
-168.5658
Ph
N
O
N
CF3
F
F
3x
19F NMR (376 MHz, Chloroform-d)
3.00
0.99
0.96
f1 (ppm)

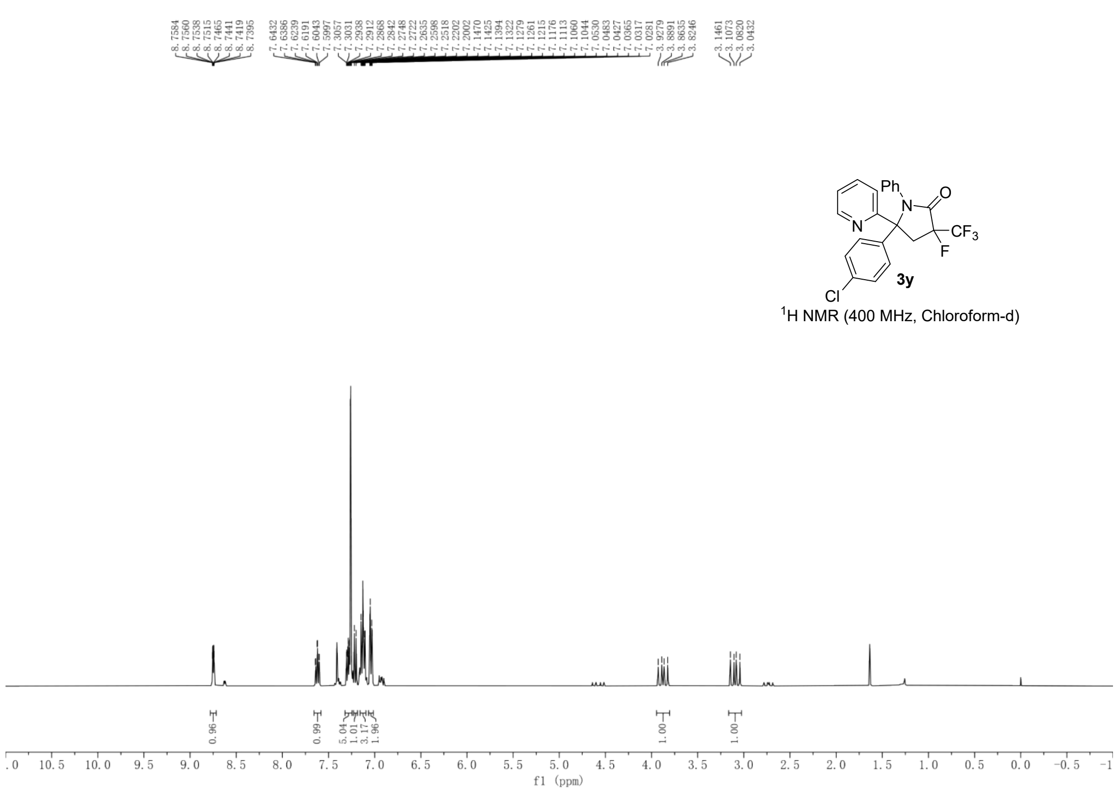

Ph
N
O
CF3
F
Cl
3y
1H NMR (400 MHz, Chloroform-d)
f1 (ppm)


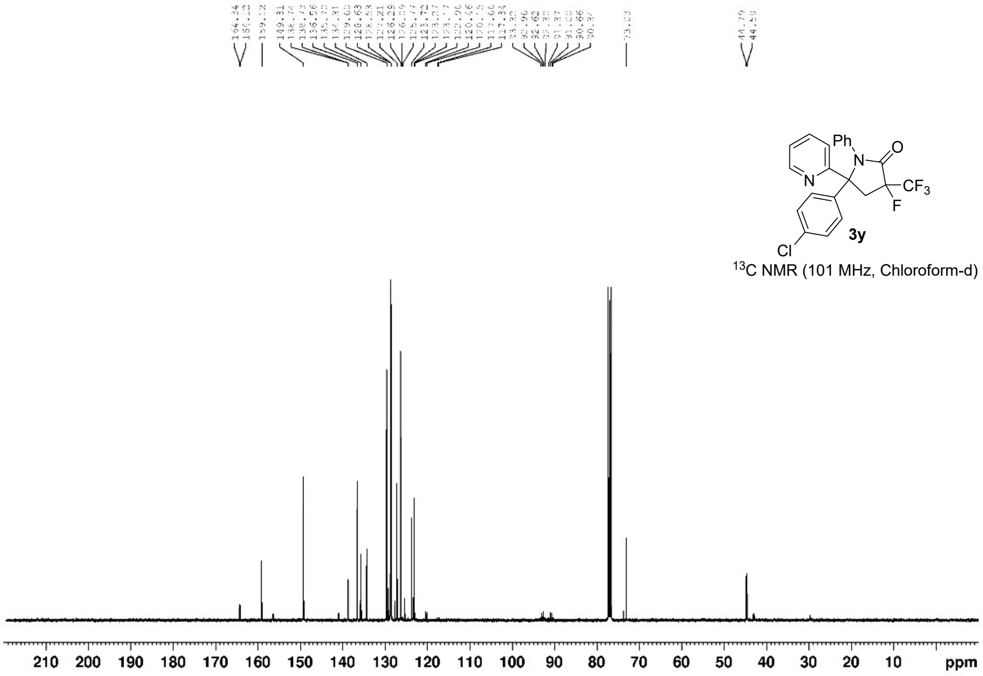

Ph
N
O
CF3
F
Cl
3y
13C NMR (101 MHz, Chloroform-d)
ppm

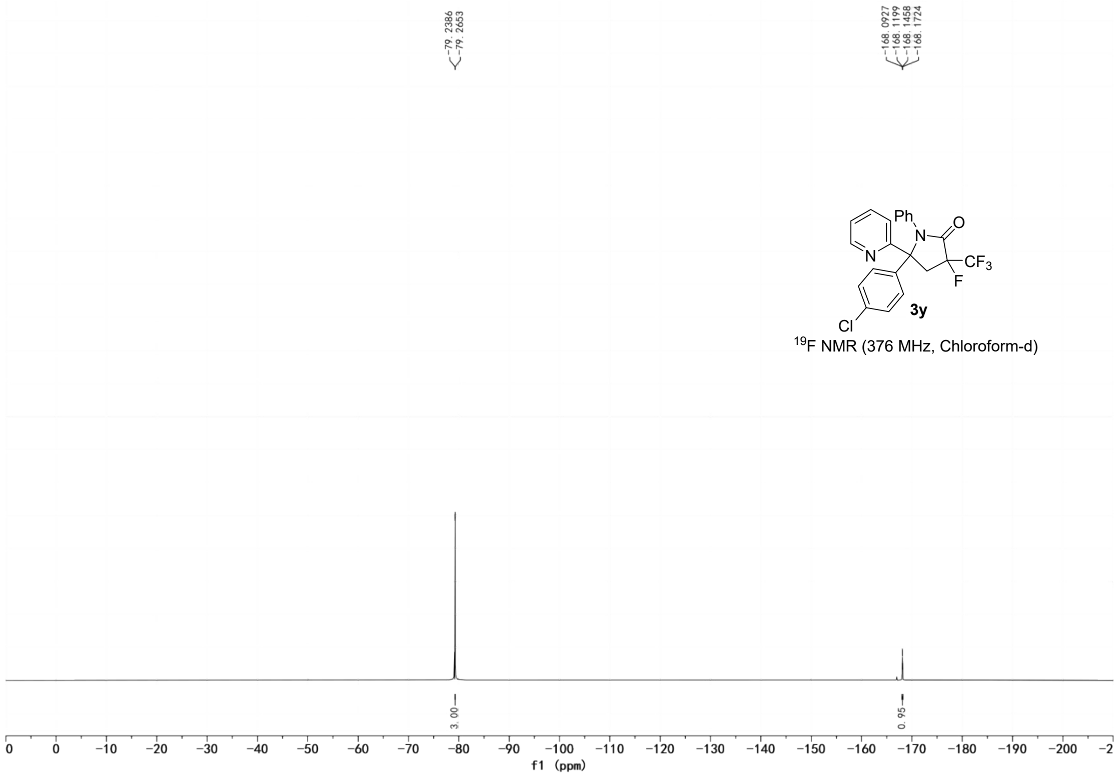

$^{19}$F NMR (376 MHz, Chloroform-d)

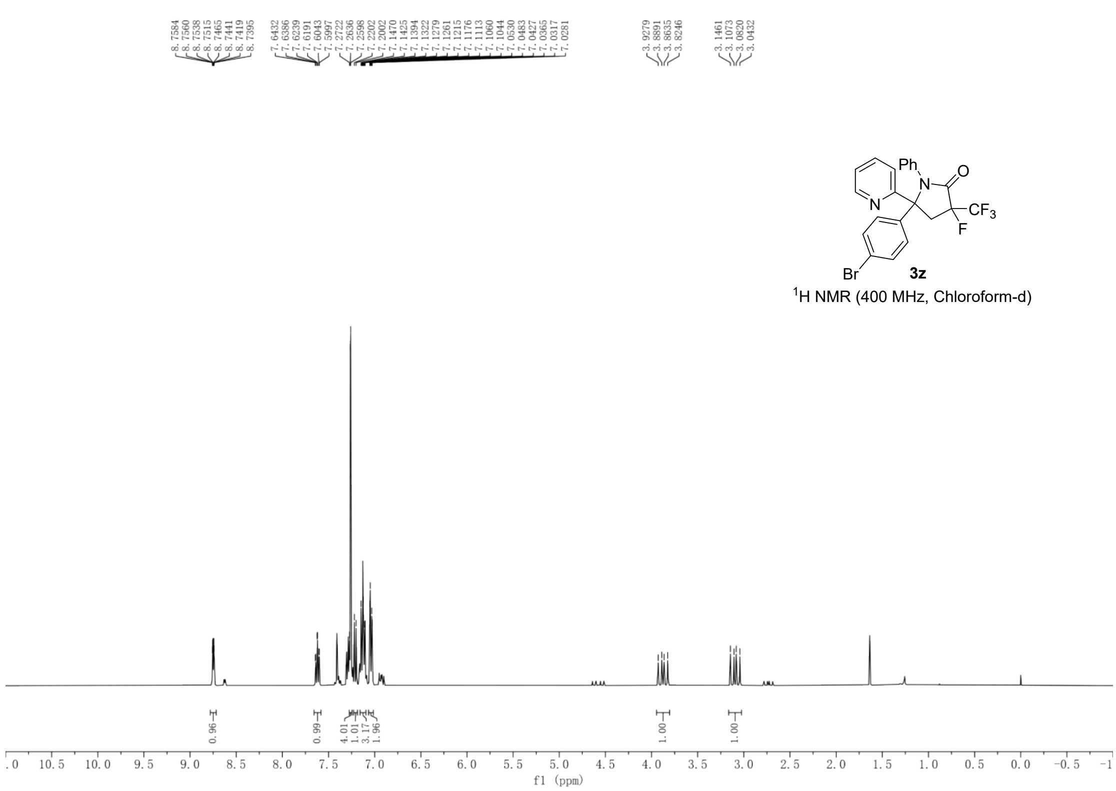
Ph
N
O
CF3
F
N
Br
3z
1H NMR (400 MHz, Chloroform-d)
f1 (ppm)

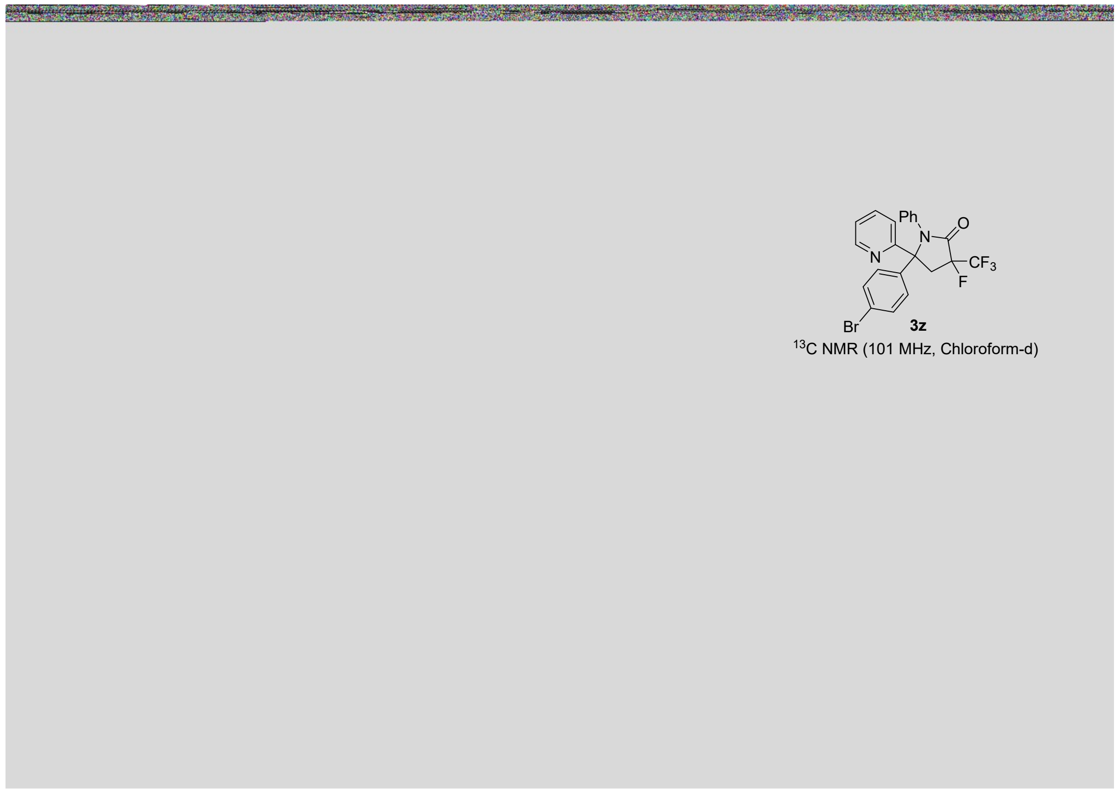
Ph
N
O
CF3
F
N
Br
3z
13C NMR (101 MHz, Chloroform-d)

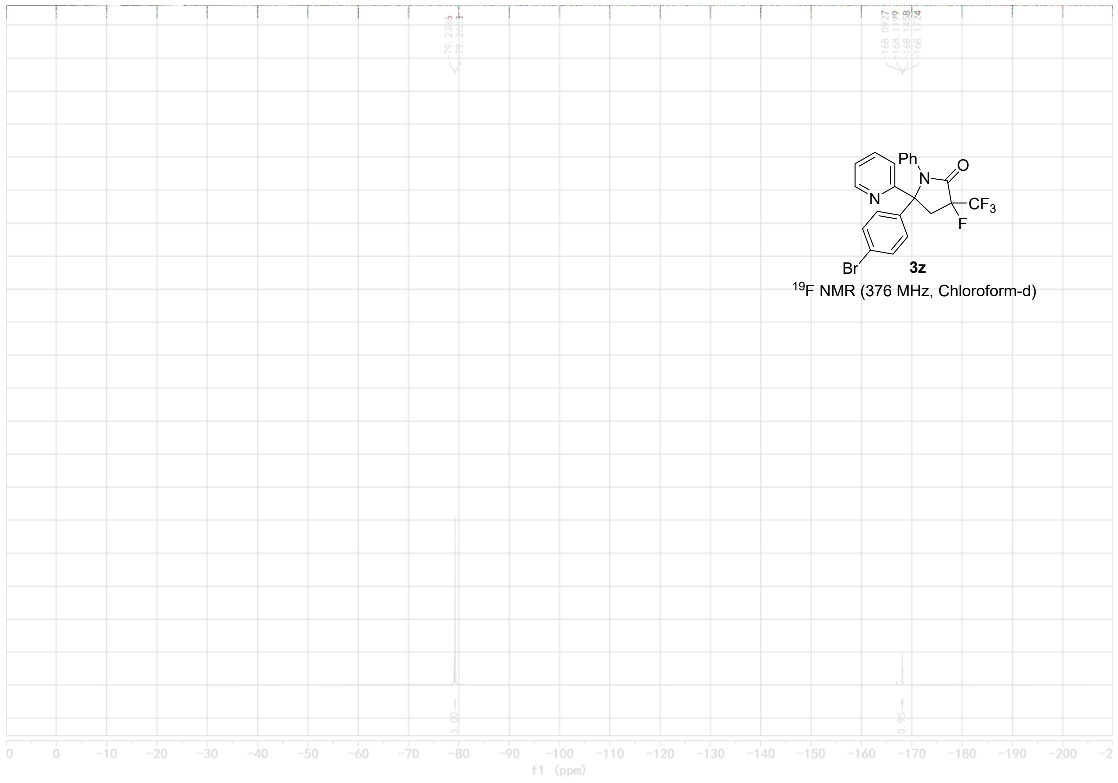

Ph
N
O
N
CF3
F
Br
3z
19F NMR (376 MHz, Chloroform-d)
f1 (ppm)

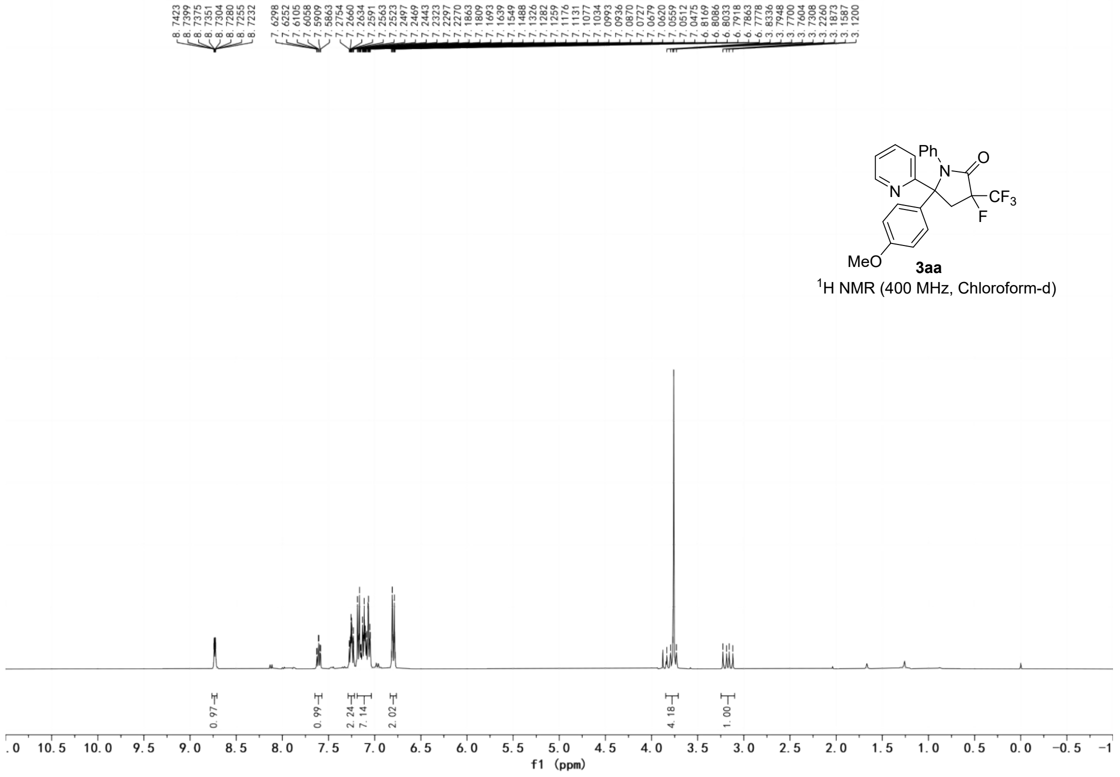
Ph
MeO
3aa
$^{1}$H NMR (400 MHz, Chloroform-d)
f1 (ppm)

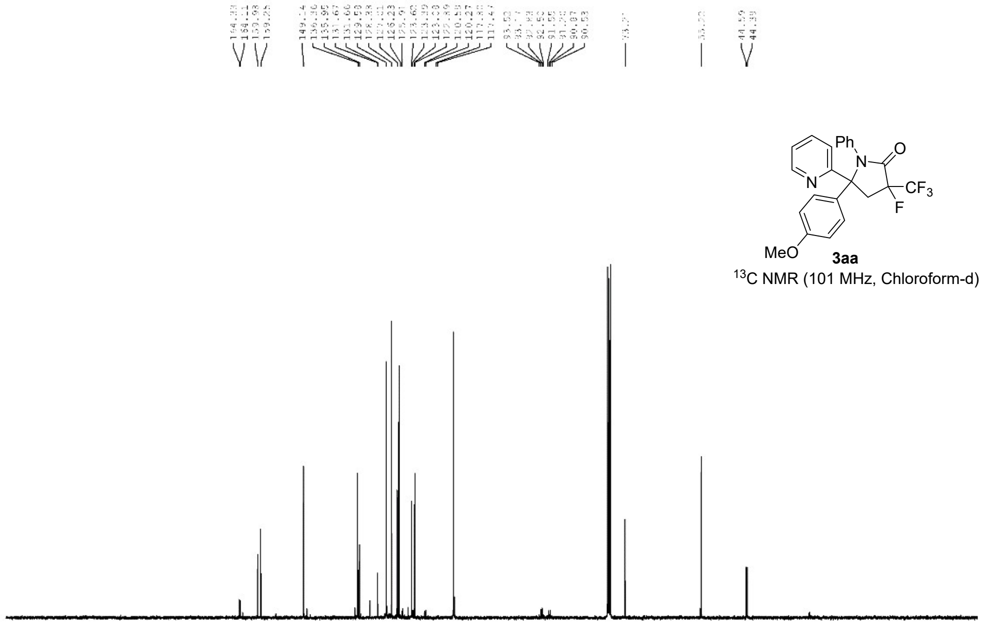
Ph
MeO
3aa
$^{13}$C NMR (101 MHz, Chloroform-d)
ppm

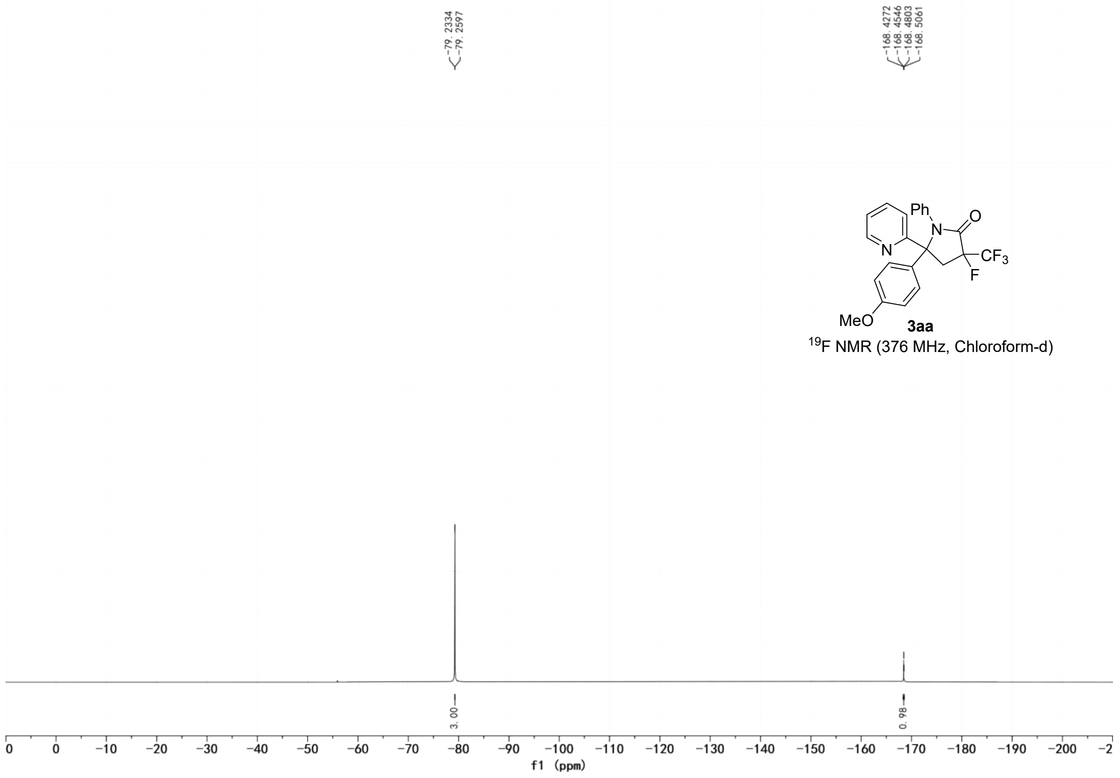

-79.2334
-79.2597
-168.4272
-168.4546
-168.4803
-168.5061
Ph
N
O
N
CF3
F
MeO
3aa
19F NMR (376 MHz, Chloroform-d)
3.00
0.98
f1 (ppm)

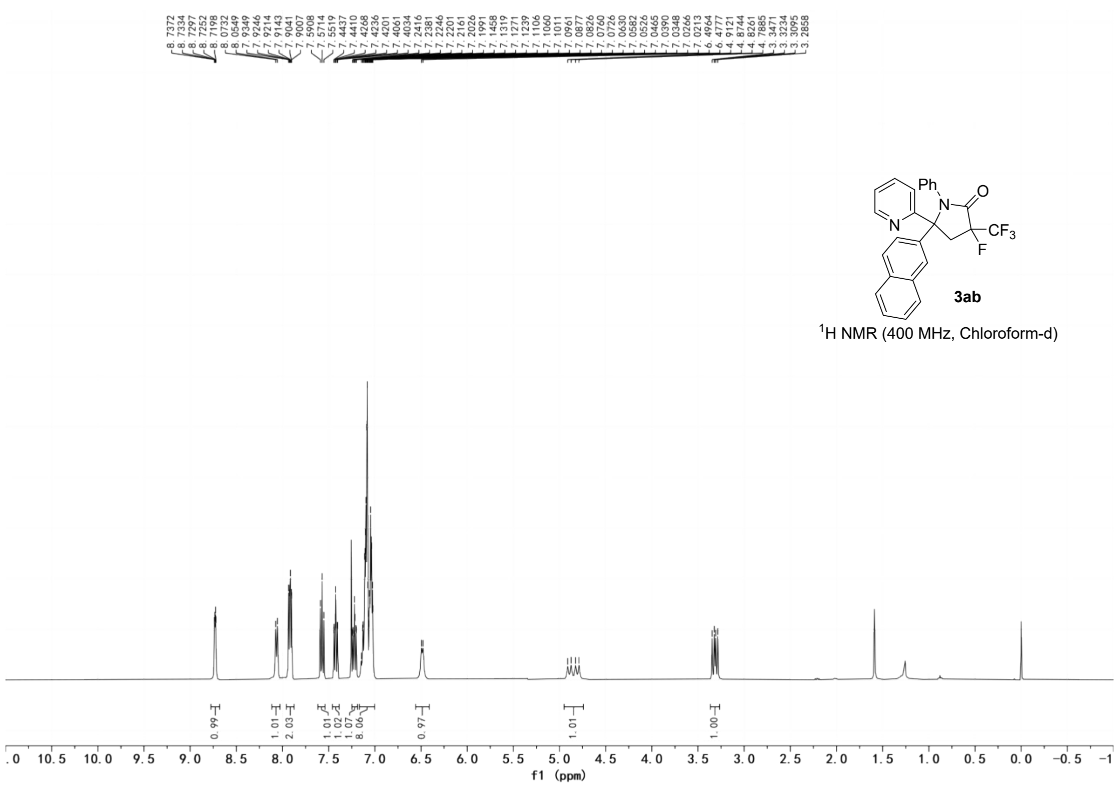


**3ab**

$^1$H NMR (400 MHz, Chloroform-d)

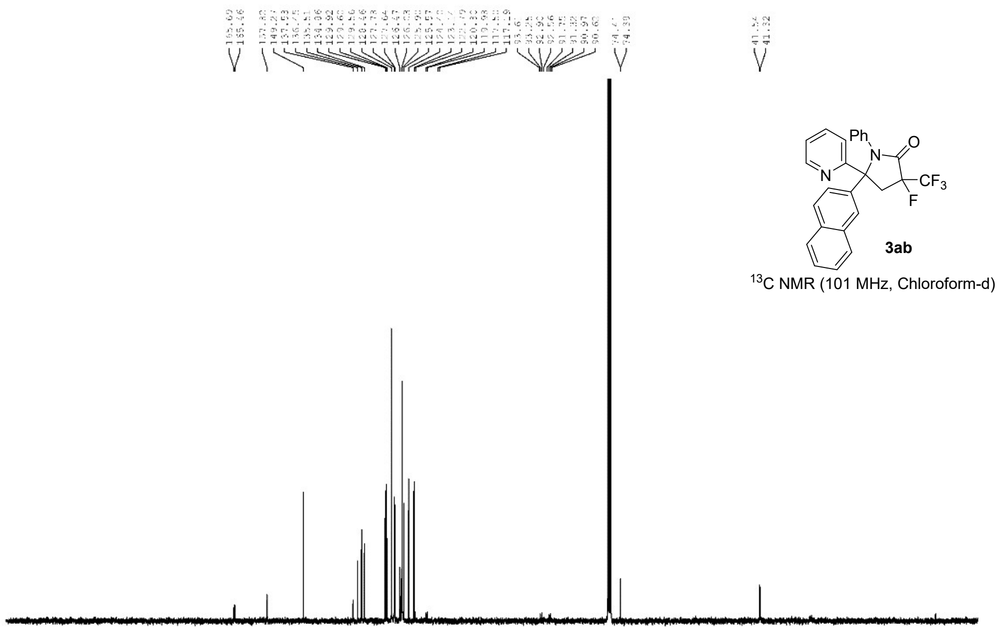


**3ab**

$^{13}$C NMR (101 MHz, Chloroform-d)

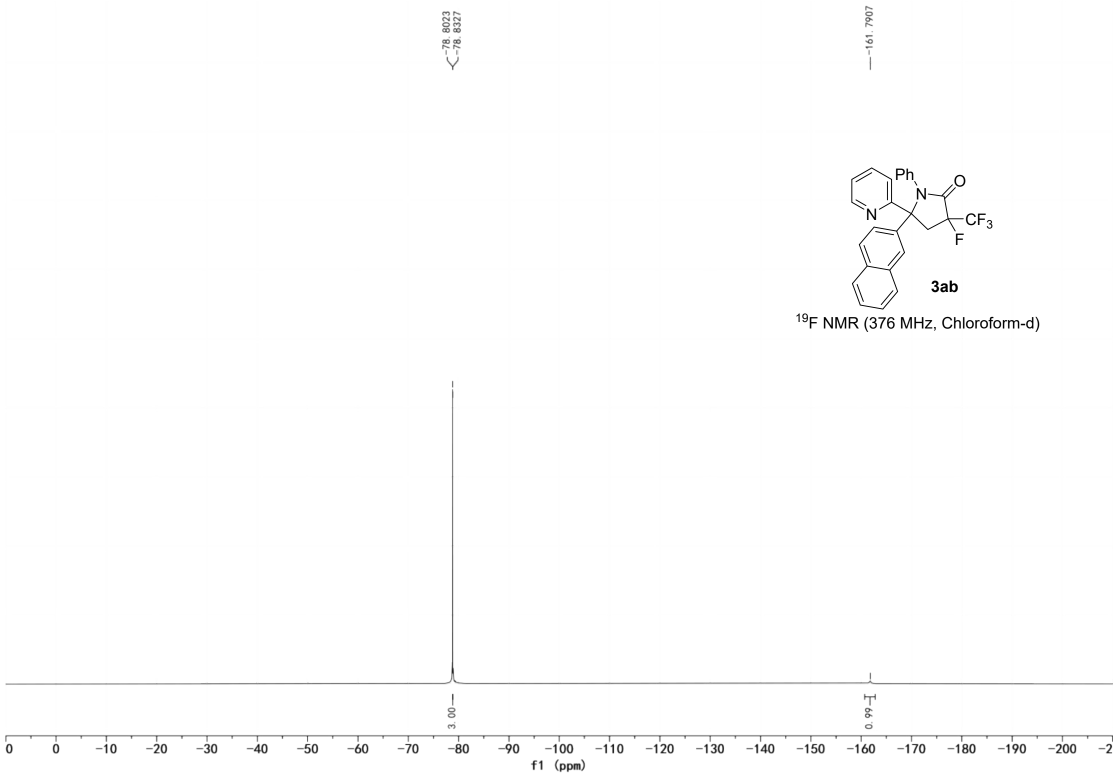
-78.8023
-78.8327
-161.7907
Ph
N
O
N
CF3
F
3ab
19F NMR (376 MHz, Chloroform-d)
3.00
0.99
f1 (ppm)

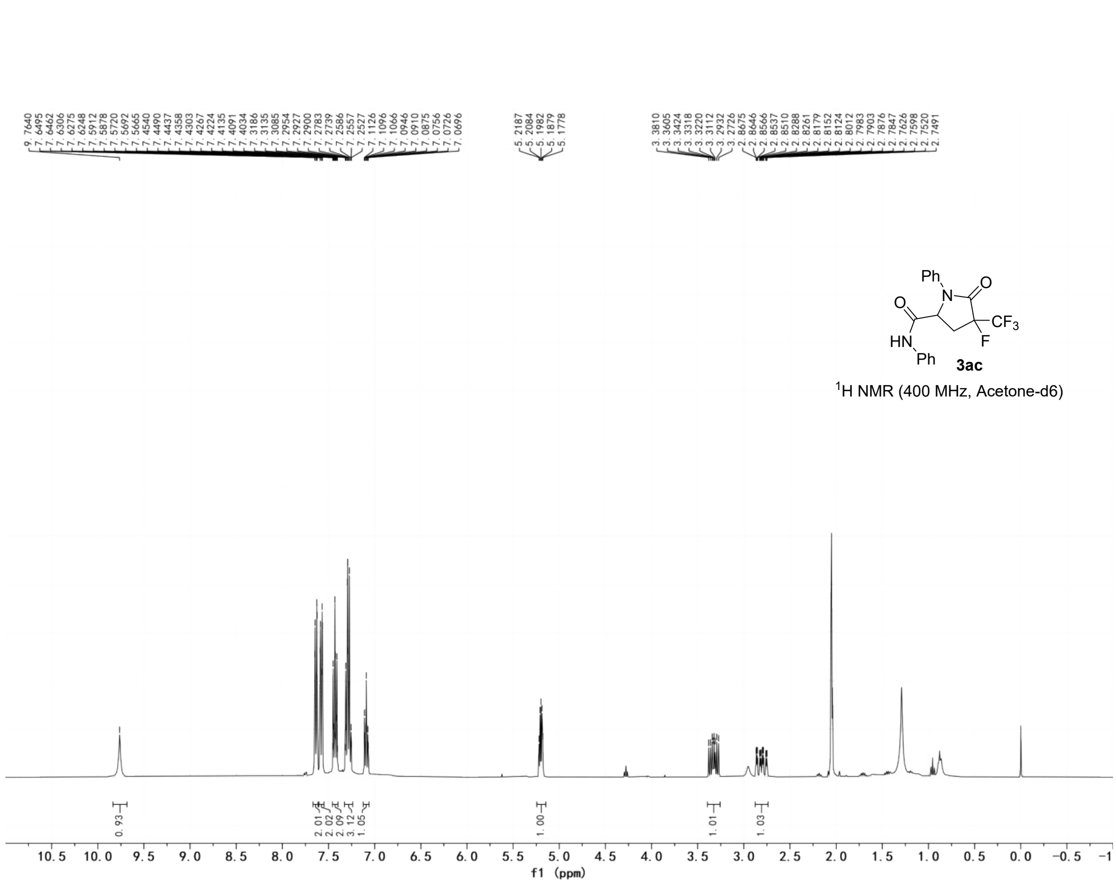
Ph
N
O
O
CF3
F
HN
Ph
3ac
1H NMR (400 MHz, Acetone-d6)
f1 (ppm)

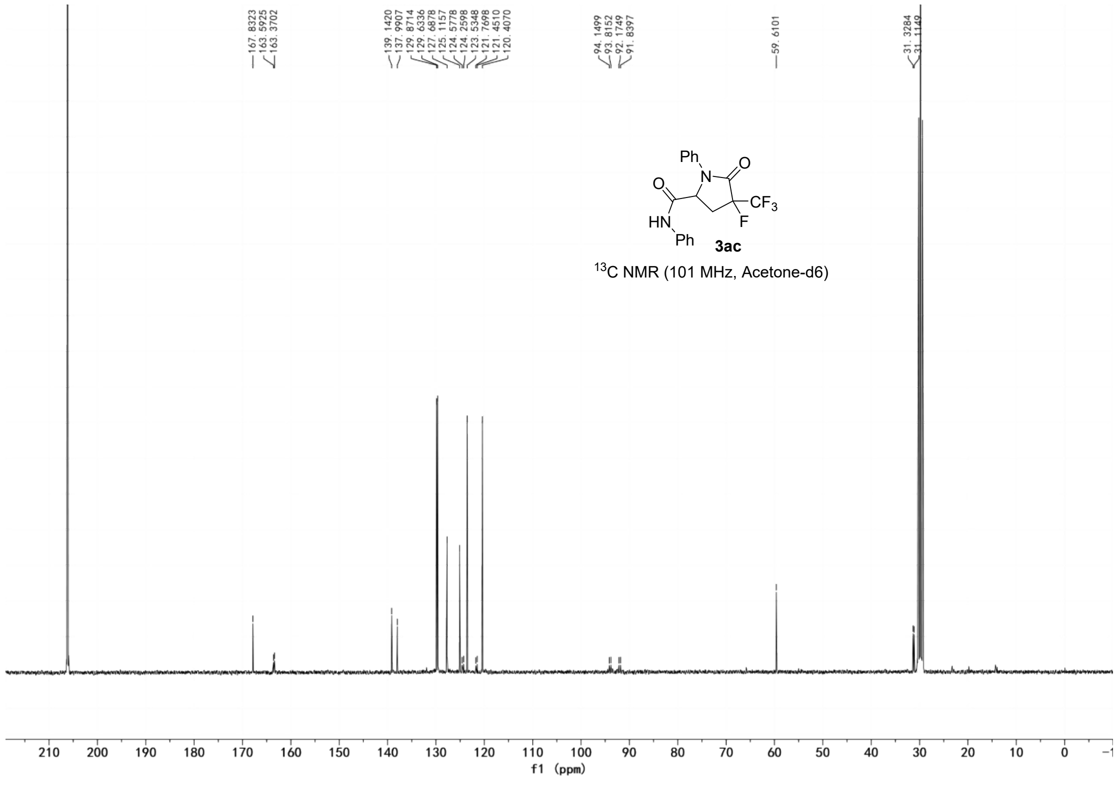
Ph
N
O
O
CF3
F
HN
Ph
3ac
13C NMR (101 MHz, Acetone-d6)
f1 (ppm)

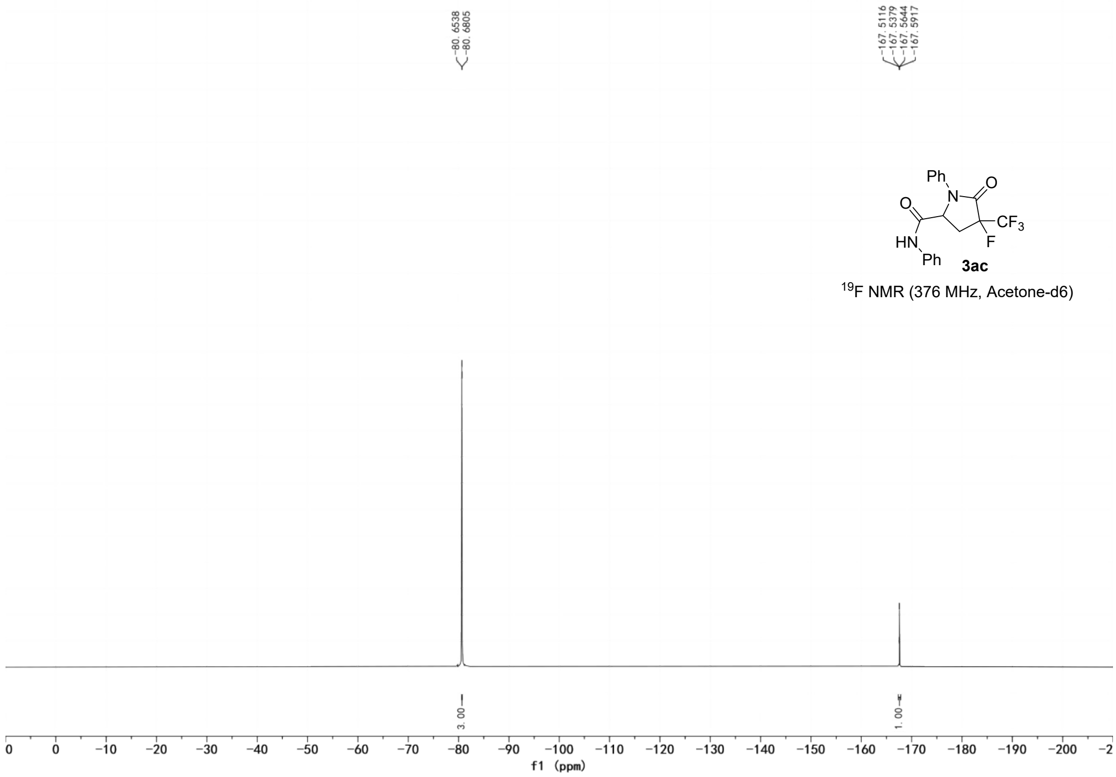


**3ac**

$^{19}$F NMR (376 MHz, Acetone-d6)

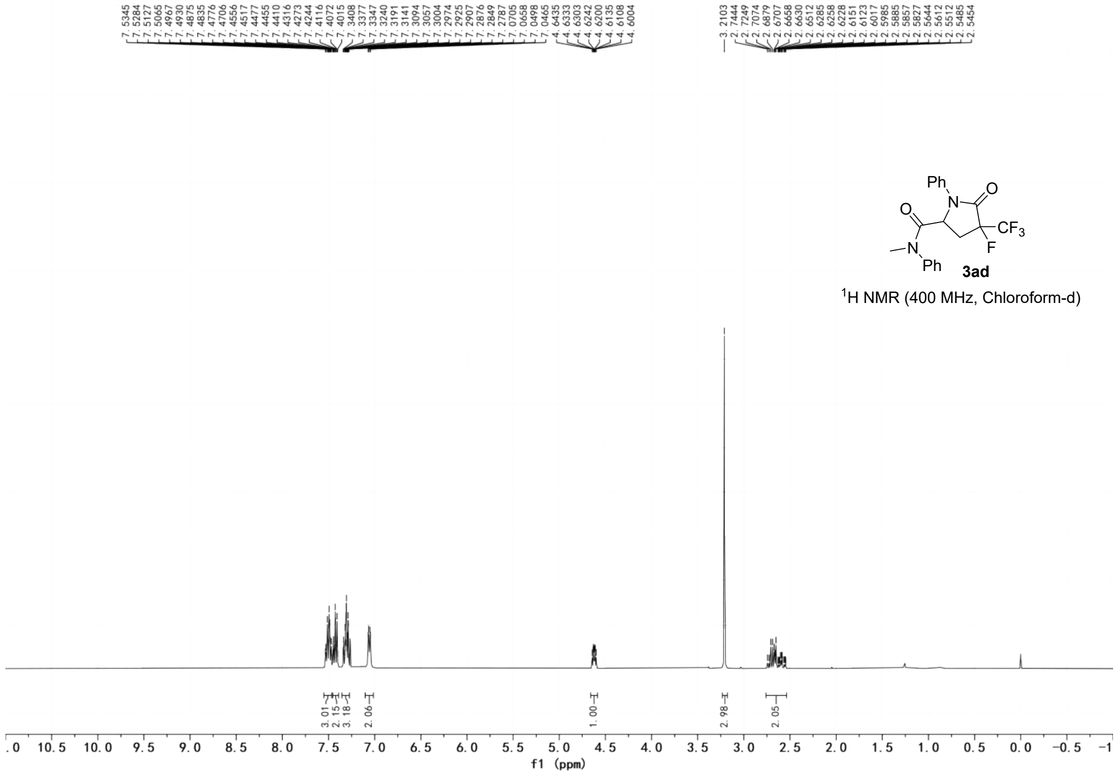


**3ad**

$^{1}$H NMR (400 MHz, Chloroform-d)

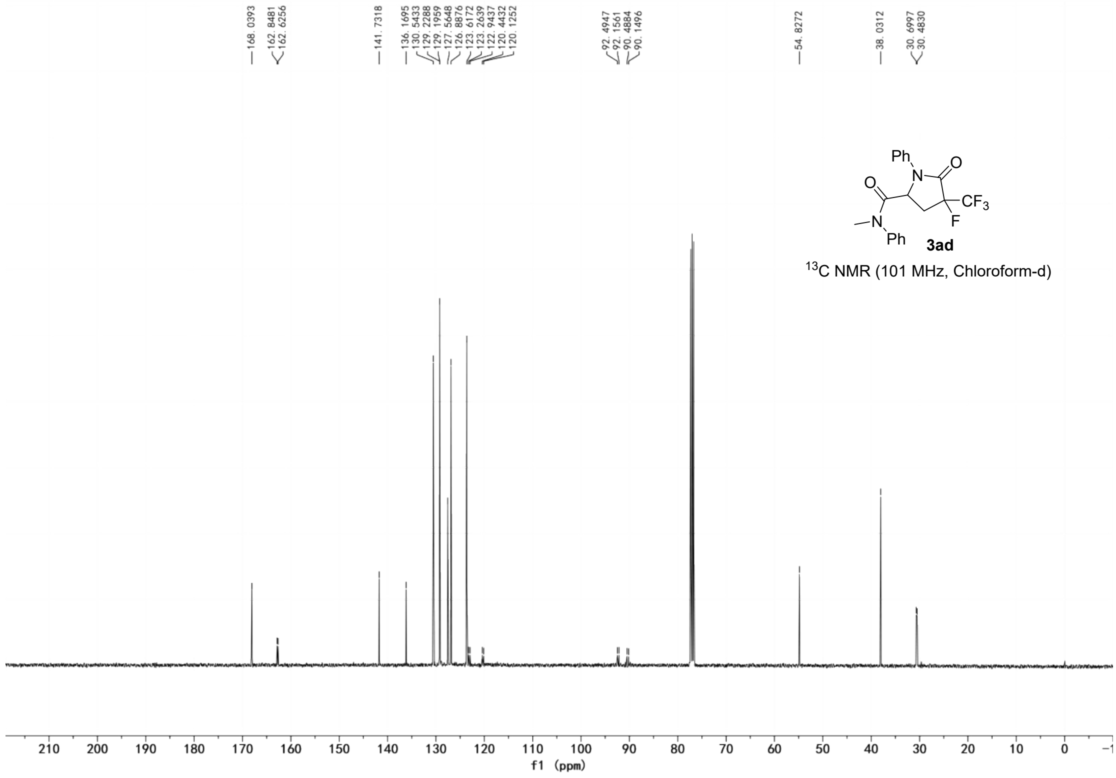
3ad
$^{13}$C NMR (101 MHz, Chloroform-d)
f1 (ppm)

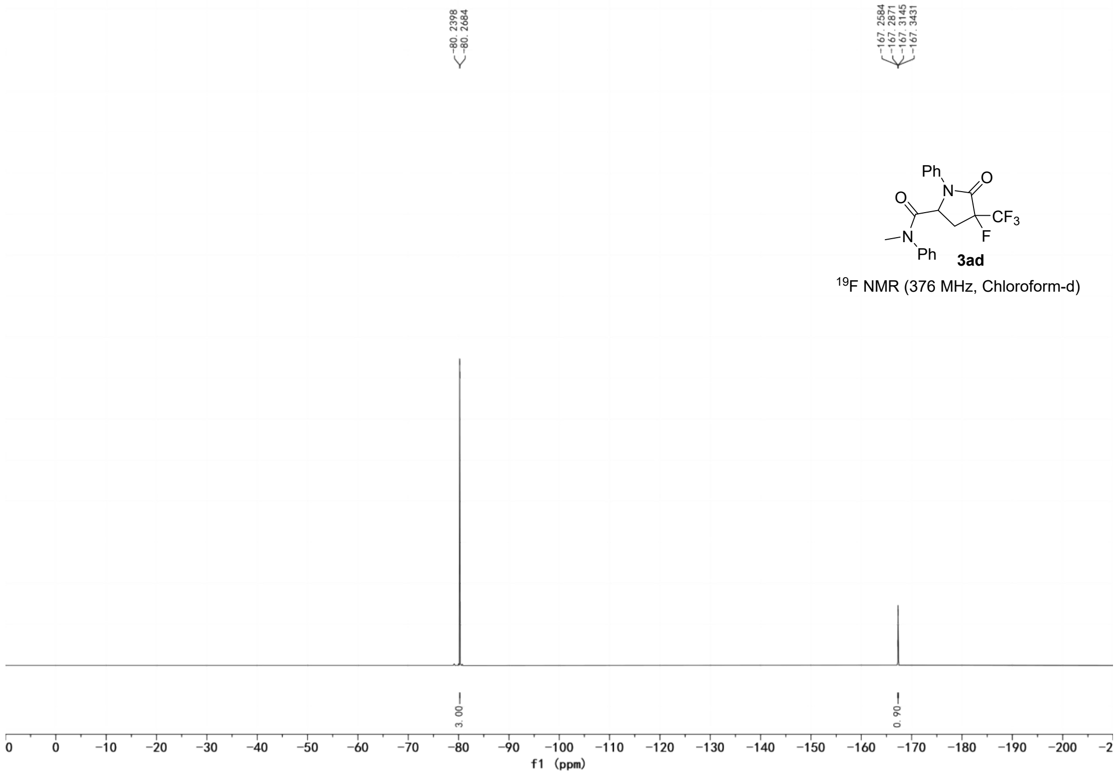
3ad
$^{19}$F NMR (376 MHz, Chloroform-d)
f1 (ppm)

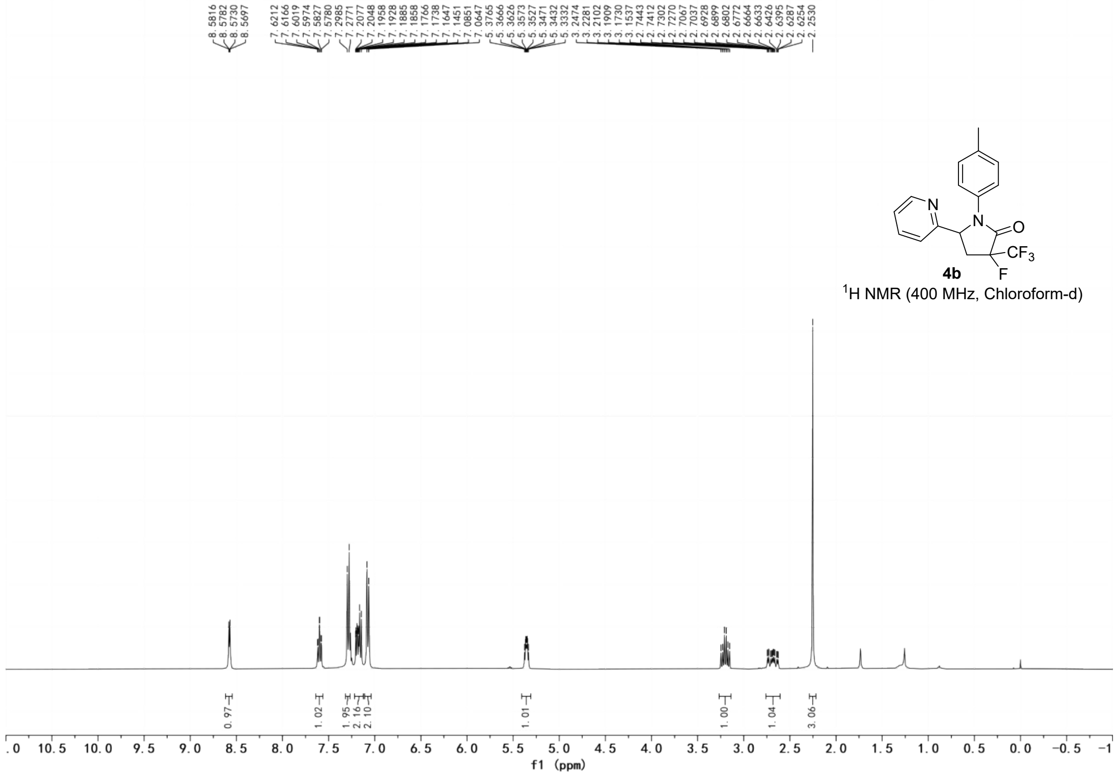
4b
$^{1}$H NMR (400 MHz, Chloroform-d)
f1 (ppm)

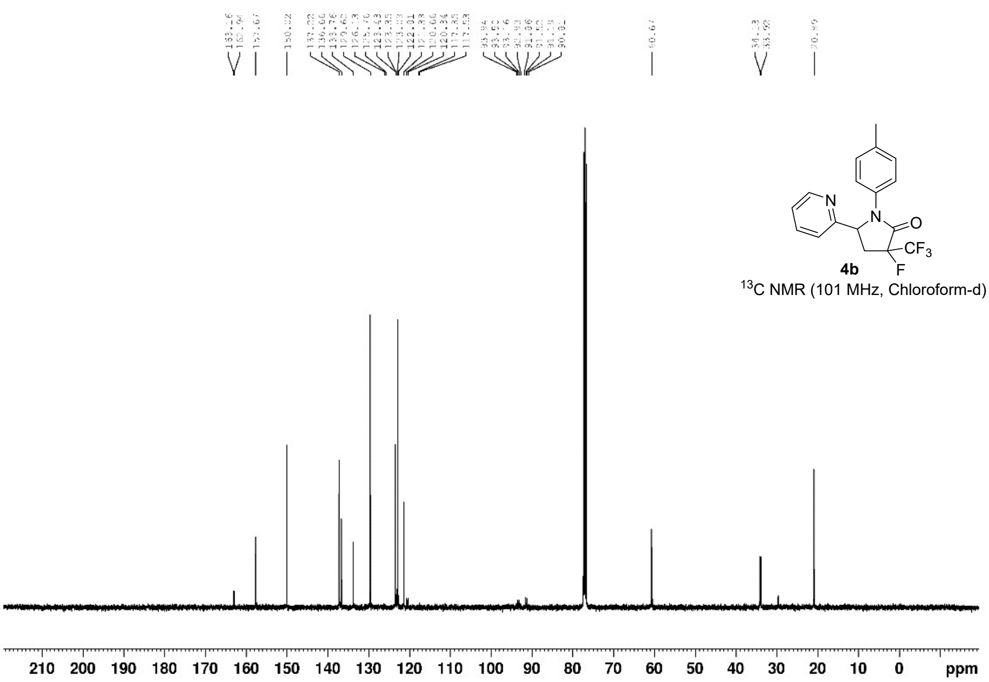
4b
$^{13}$C NMR (101 MHz, Chloroform-d)
ppm

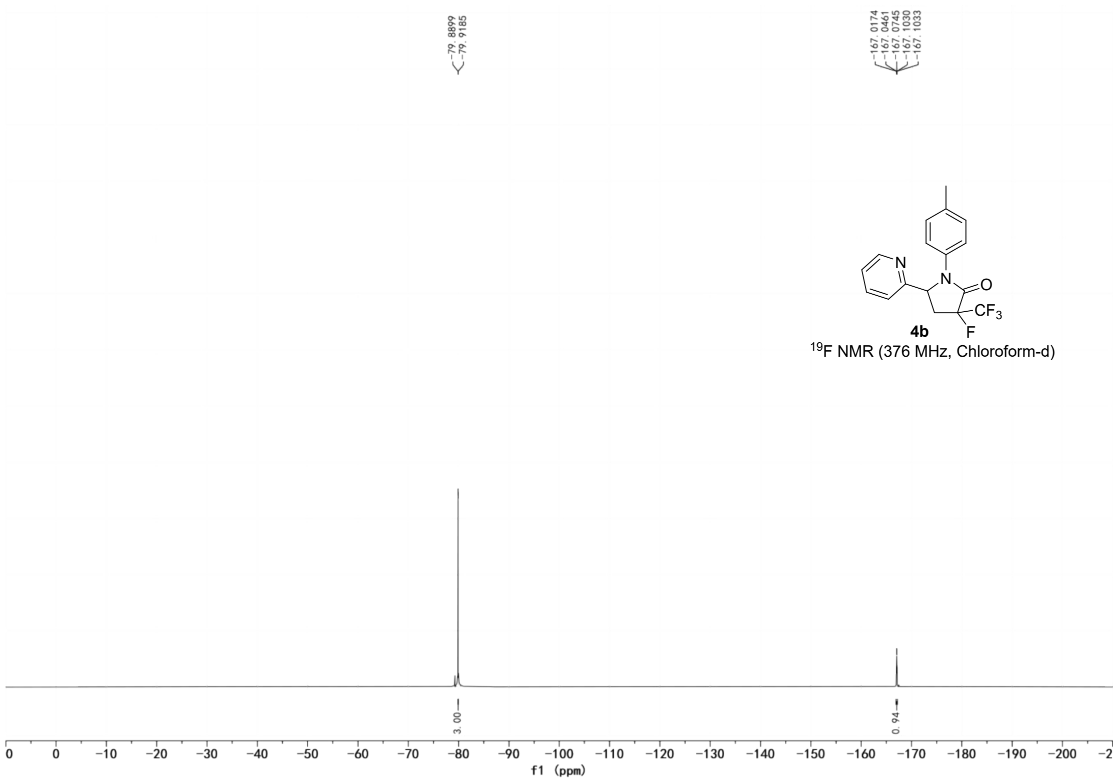
-79.8899
-79.9185
-167.0174
-167.0461
-167.0745
-167.1030
-167.1033
4b
CF3
F
N
O
19F NMR (376 MHz, Chloroform-d)
3.00
0.94
f1 (ppm)

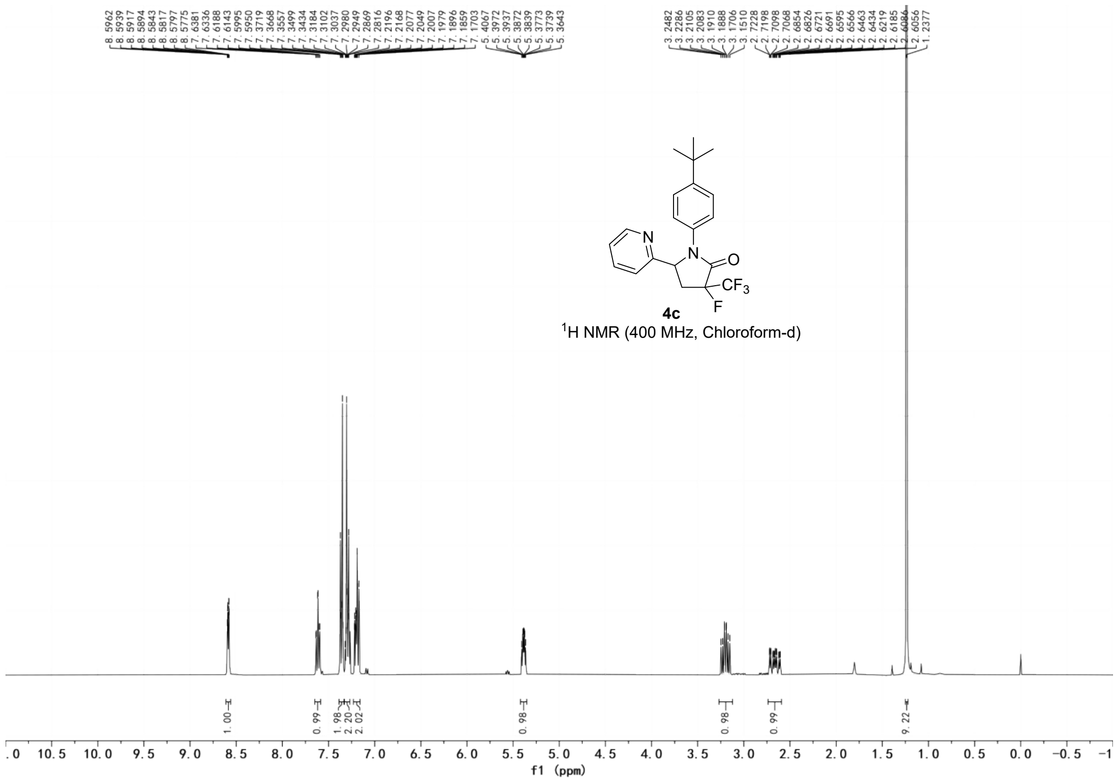

**4c**
$^{1}H$ NMR (400 MHz, Chloroform-d)

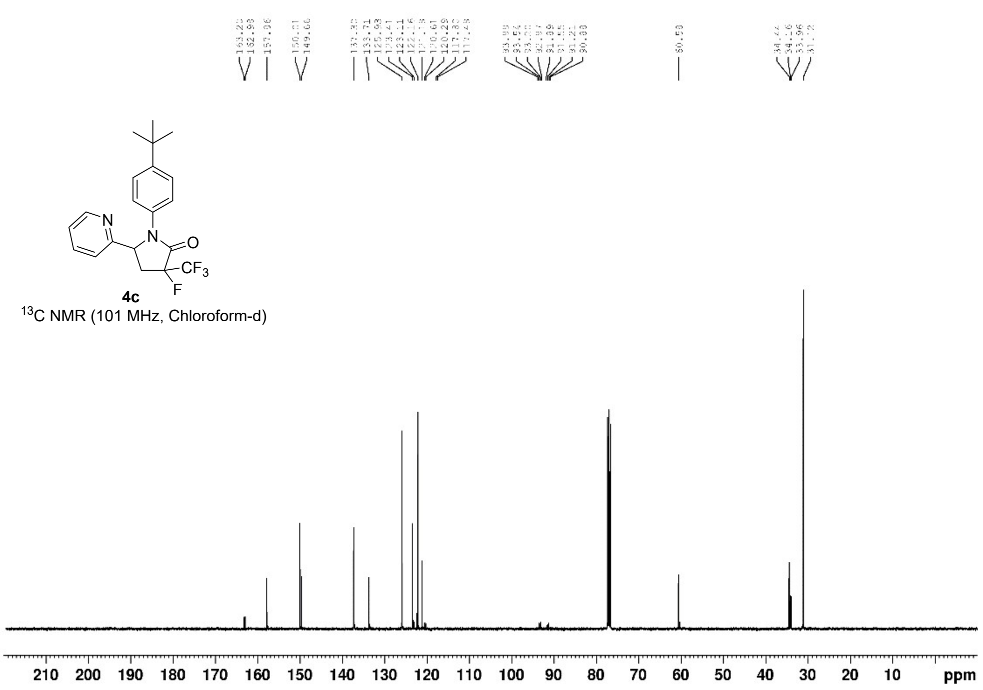

**4c**
$^{13}C$ NMR (101 MHz, Chloroform-d)

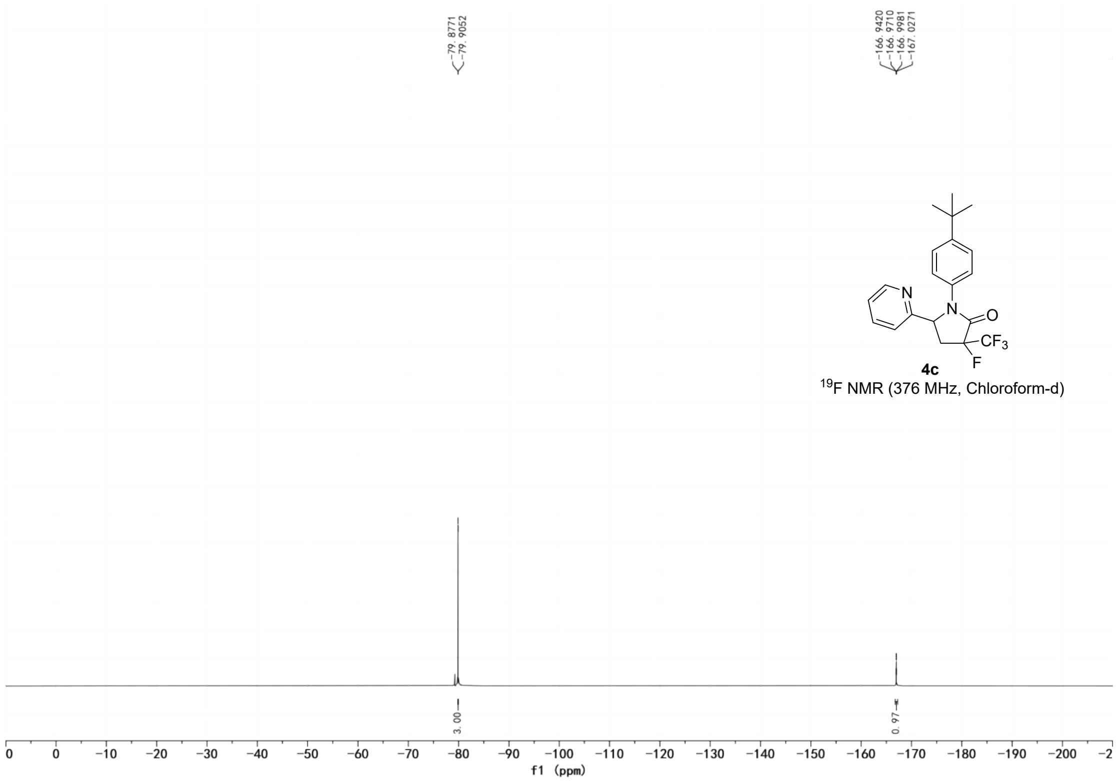

-79.8771
-79.9052
-166.9420
-166.9710
-166.9981
-167.0271
N
N
O
CF3
F
4c
19F NMR (376 MHz, Chloroform-d)
3.00
0.97
f1 (ppm)

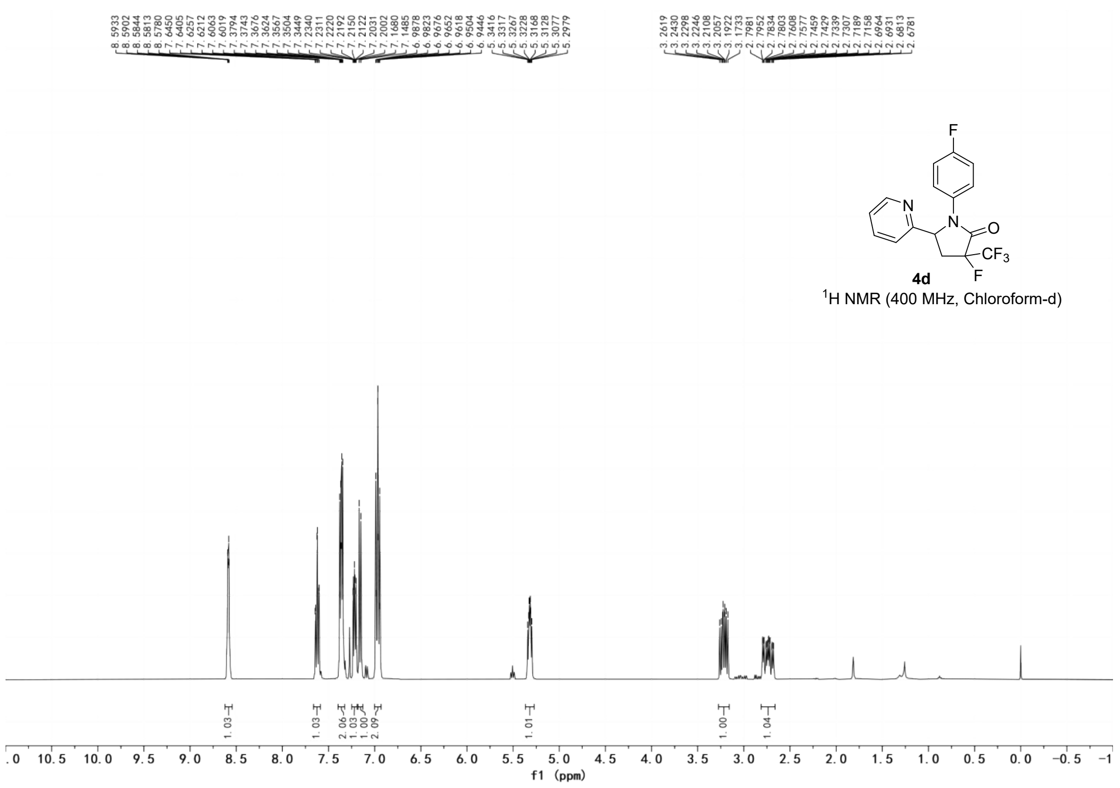
4d
$^{1}$H NMR (400 MHz, Chloroform-d)
f1 (ppm)

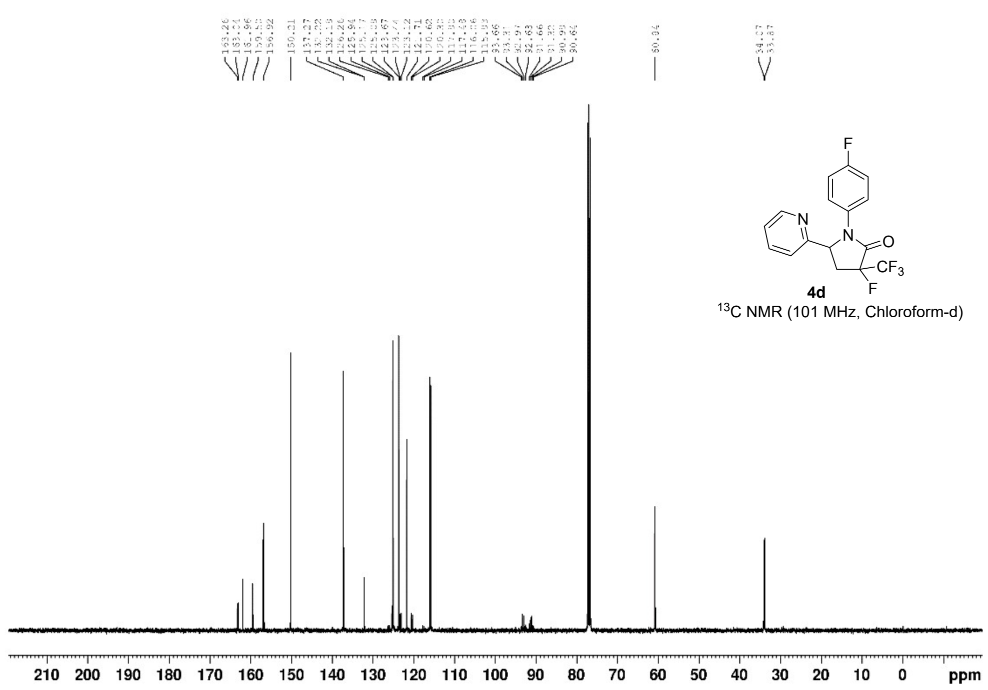
4d
$^{13}$C NMR (101 MHz, Chloroform-d)
ppm

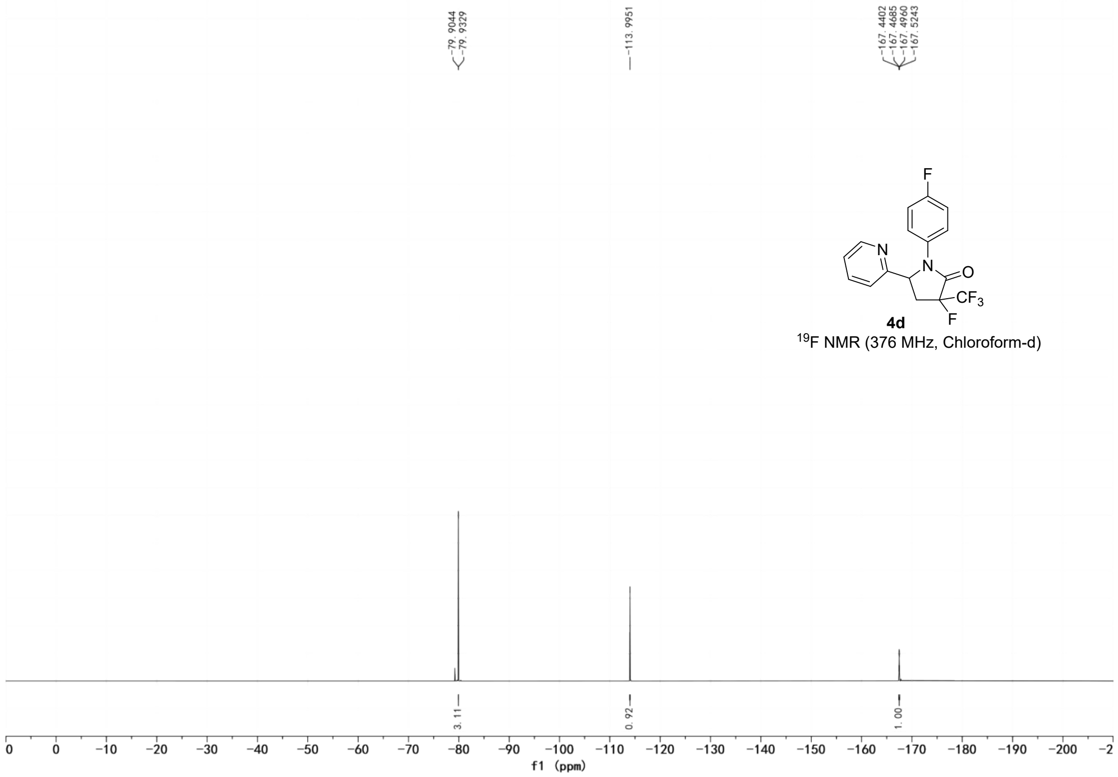
-79.9044
-79.9329
-113.9951
-167.4402
-167.4685
-167.4960
-167.5243
4d
19F NMR (376 MHz, Chloroform-d)
3.11
0.92
1.00
f1 (ppm)

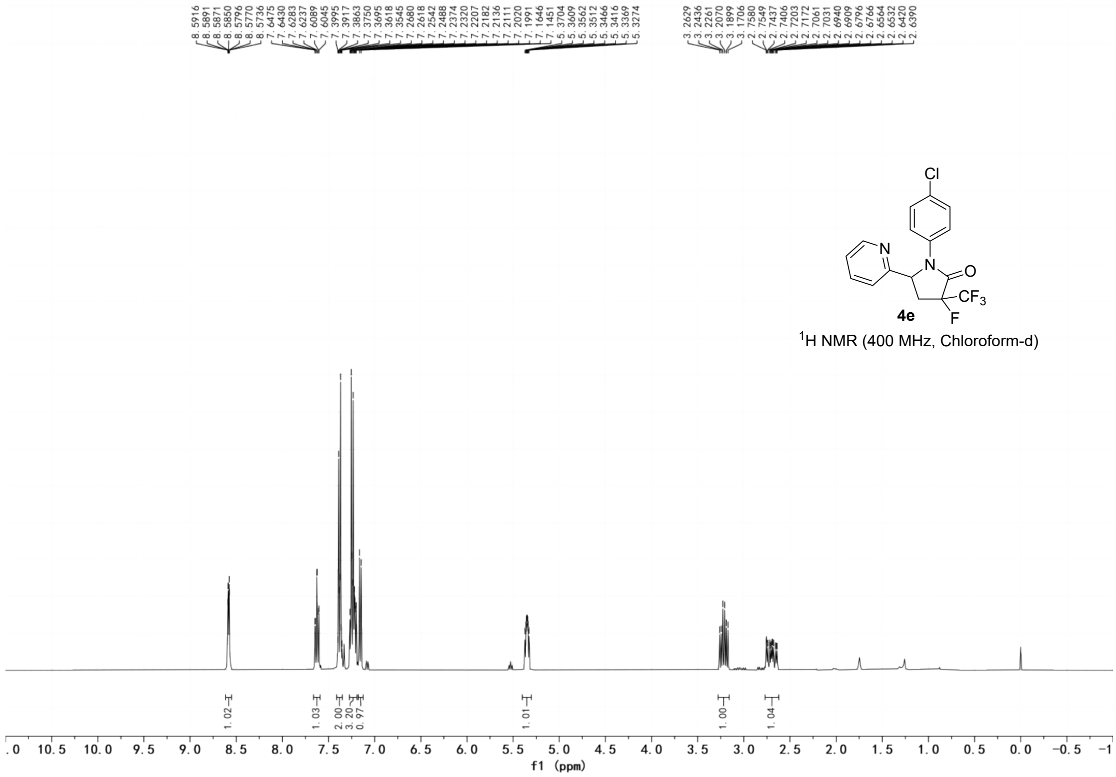
Cl
N
N
O
CF3
F
4e
1H NMR (400 MHz, Chloroform-d)
f1 (ppm)

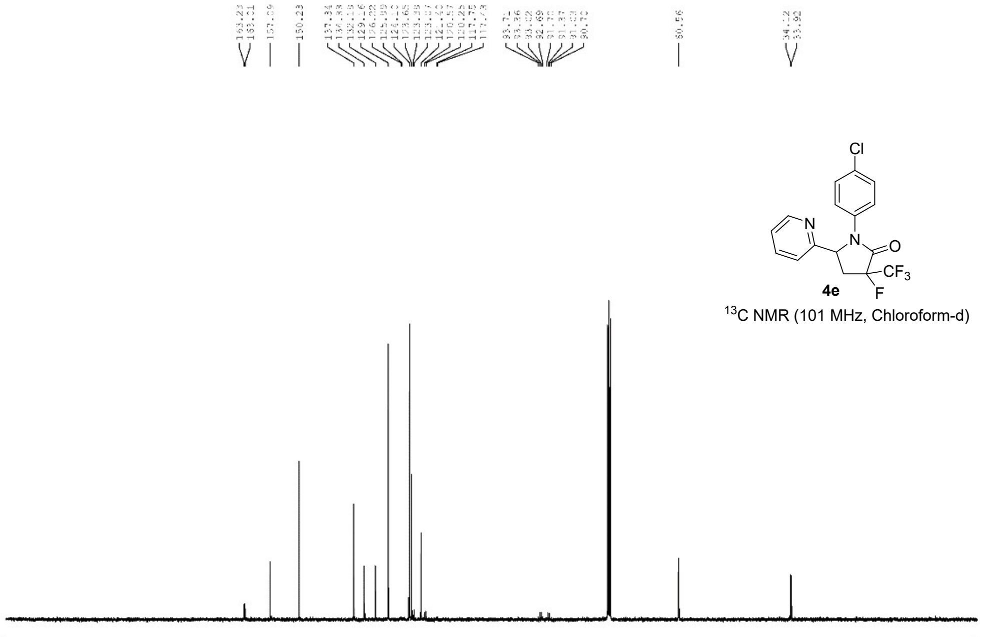
Cl
N
N
O
CF3
F
4e
13C NMR (101 MHz, Chloroform-d)
ppm

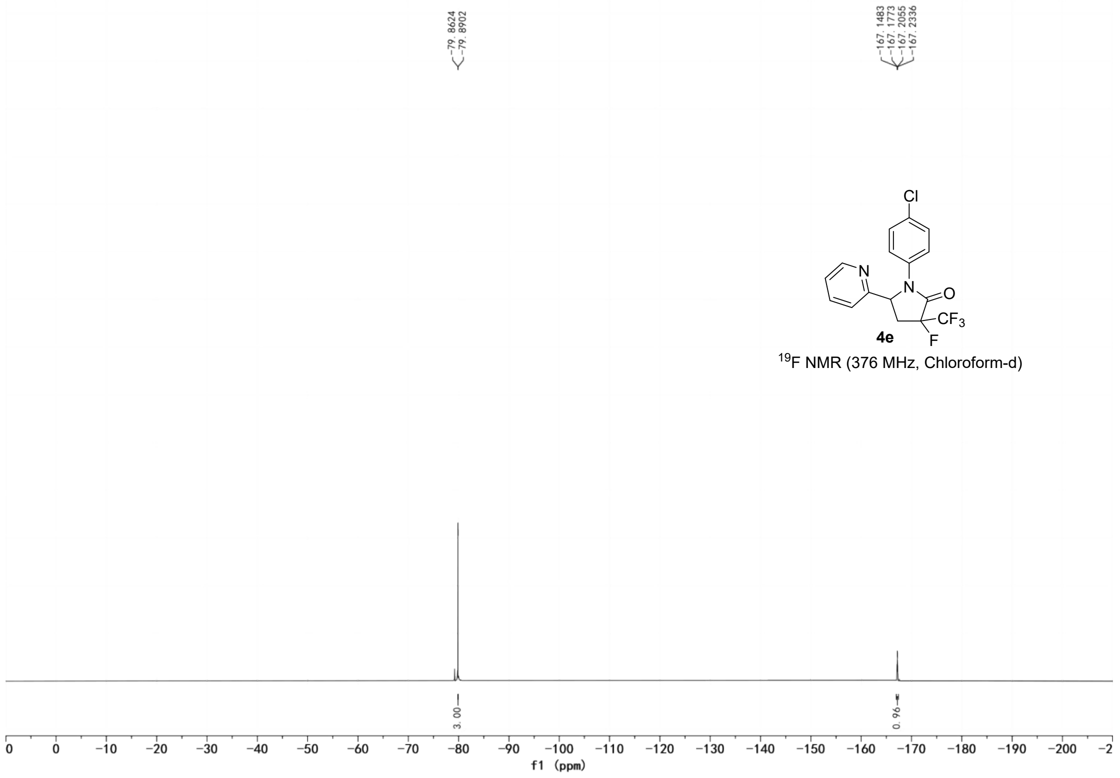

-79.8624
-79.8902
-167.1483
-167.1773
-167.2055
-167.2336
Cl
N
N
O
CF3
F
4e
19F NMR (376 MHz, Chloroform-d)
3.00
0.96
0
0
-10
-20
-30
-40
-50
-60
-70
-80
-90
-100
-110
-120
-130
-140
-150
-160
-170
-180
-190
-200
-2
f1 (ppm)

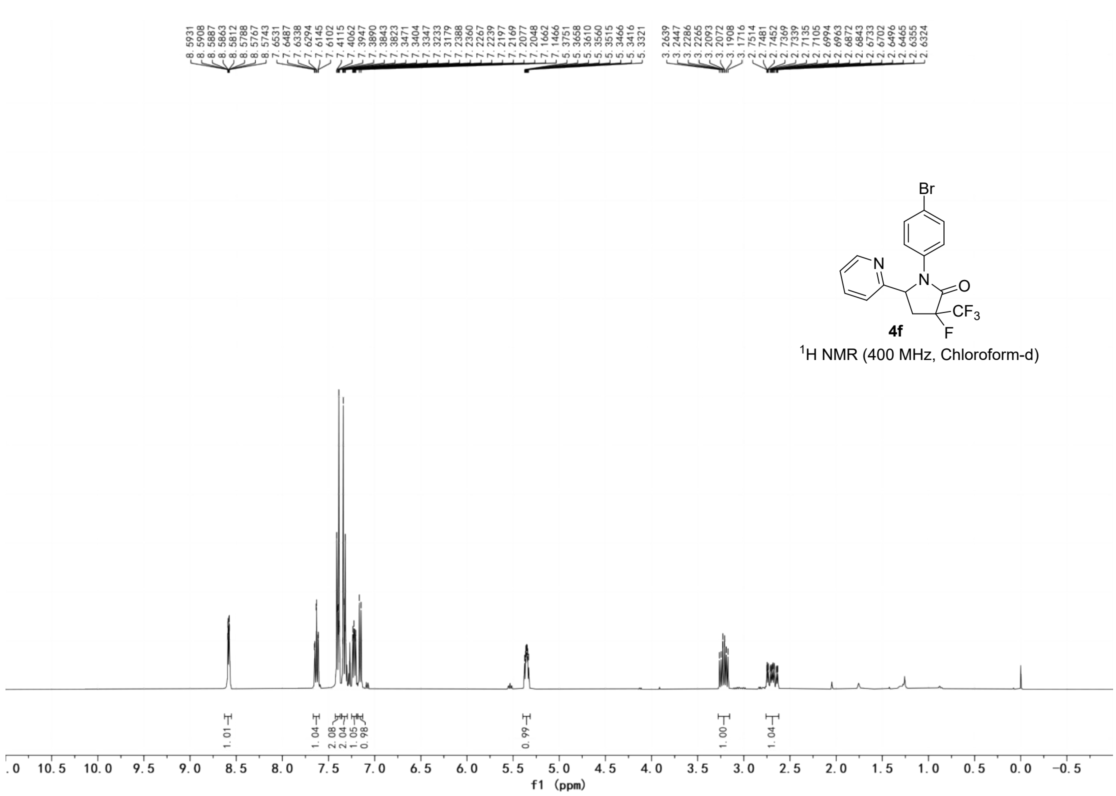

Br
N
O
CF3
F
4f
1H NMR (400 MHz, Chloroform-d)
f1 (ppm)


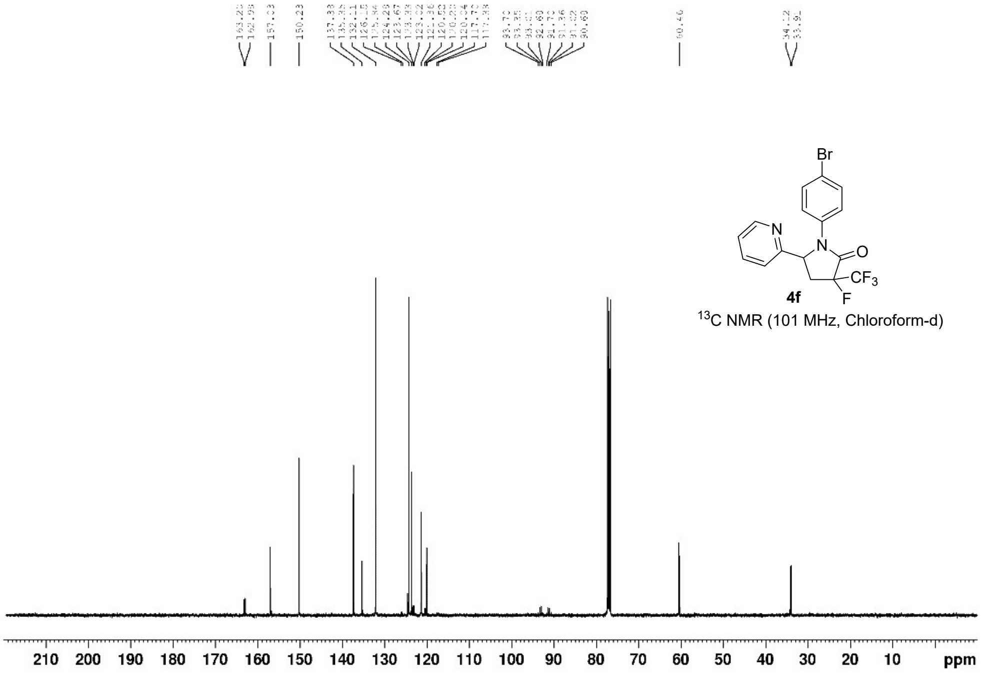

Br
N
O
CF3
F
4f
13C NMR (101 MHz, Chloroform-d)
ppm

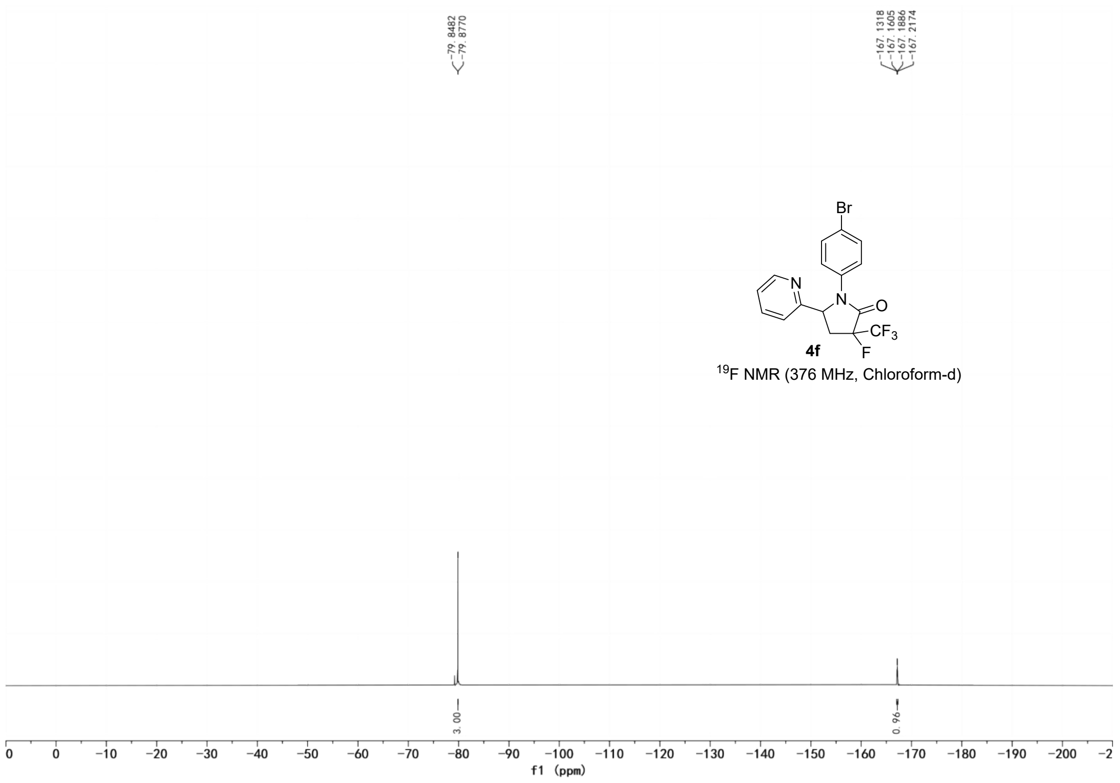
-79.8482
-79.8770
-167.1318
-167.1605
-167.1886
-167.2174
Br
N
N
O
CF3
F
4f
19F NMR (376 MHz, Chloroform-d)
3.00
0.96
f1 (ppm)

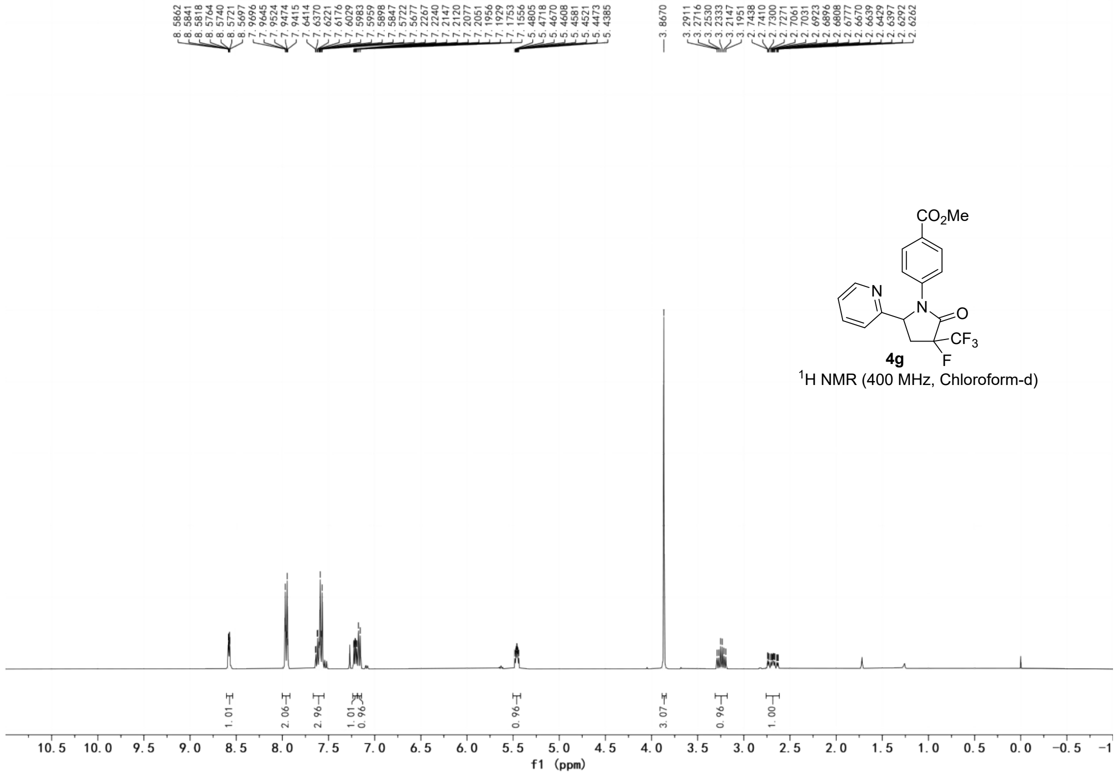
CO2Me
N
N
O
CF3
F
4g
1H NMR (400 MHz, Chloroform-d)
f1 (ppm)

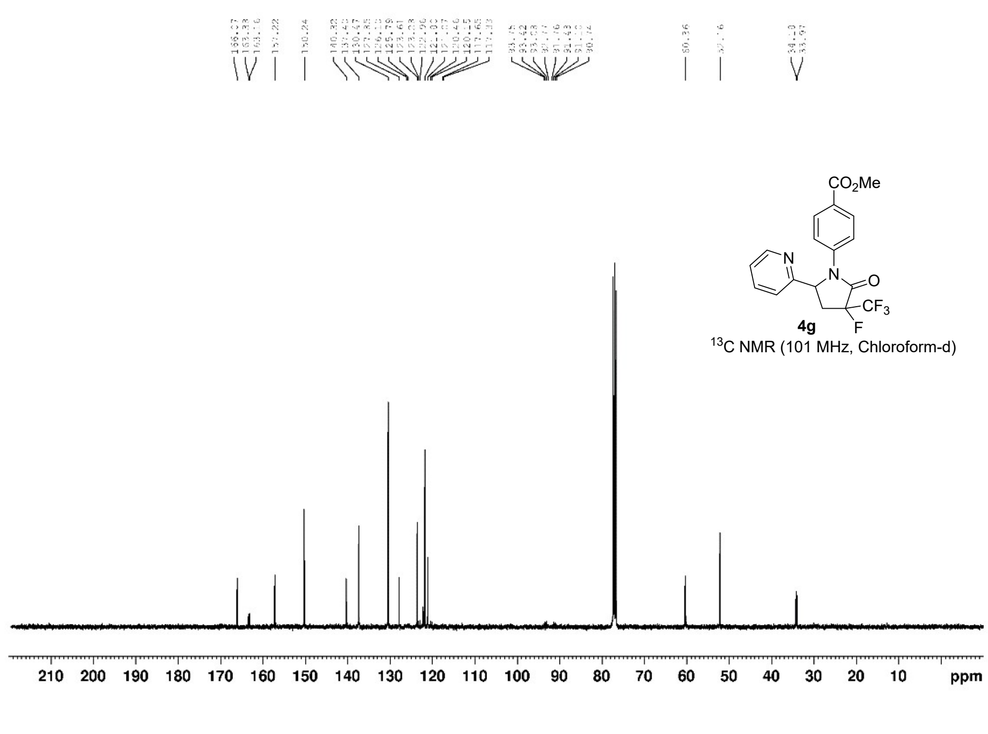
CO2Me
N
N
O
CF3
F
4g
13C NMR (101 MHz, Chloroform-d)
ppm

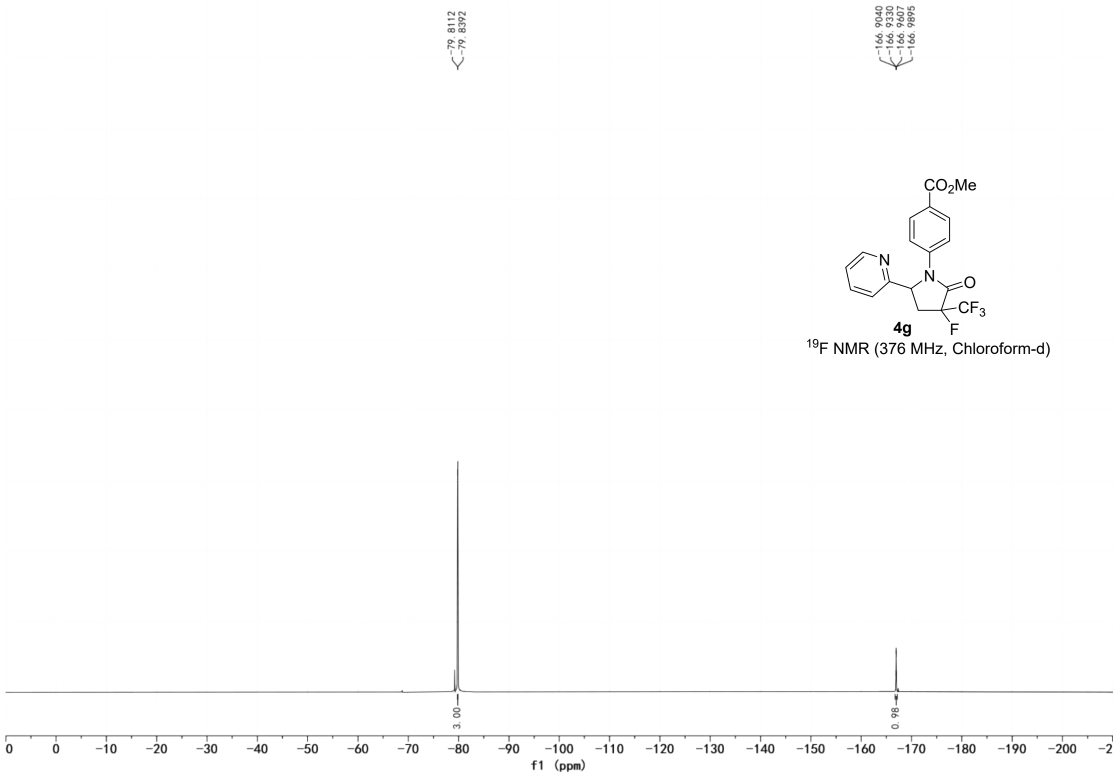
-79.8112
-79.8392
-166.9040
-166.9330
-166.9607
-166.9895
CO2Me
N
N
O
CF3
F
4g
19F NMR (376 MHz, Chloroform-d)
3.00
0.98
f1 (ppm)

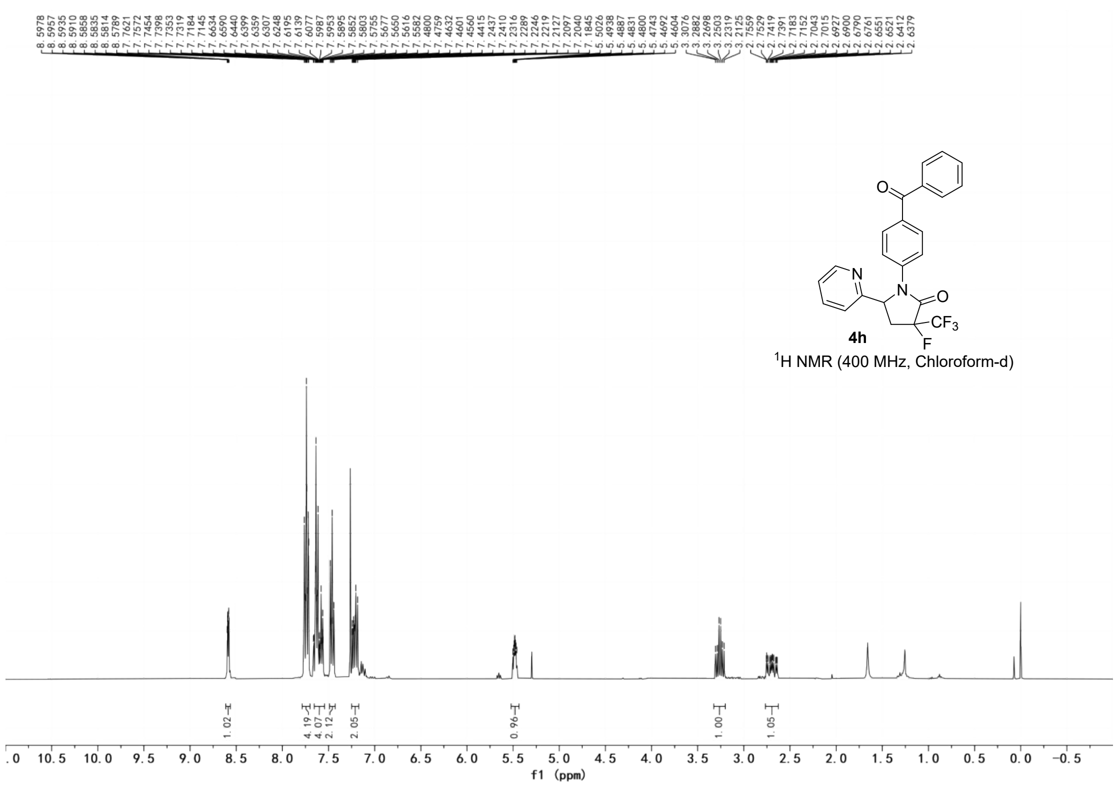
4h
$^{1}$H NMR (400 MHz, Chloroform-d)
f1 (ppm)

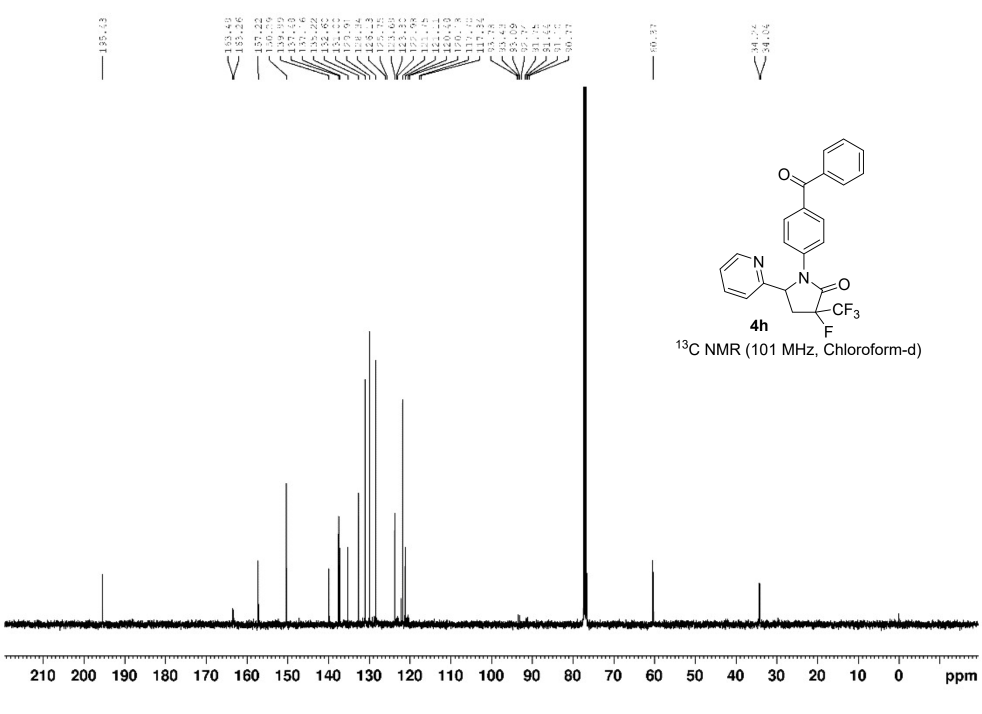
4h
$^{13}$C NMR (101 MHz, Chloroform-d)
ppm

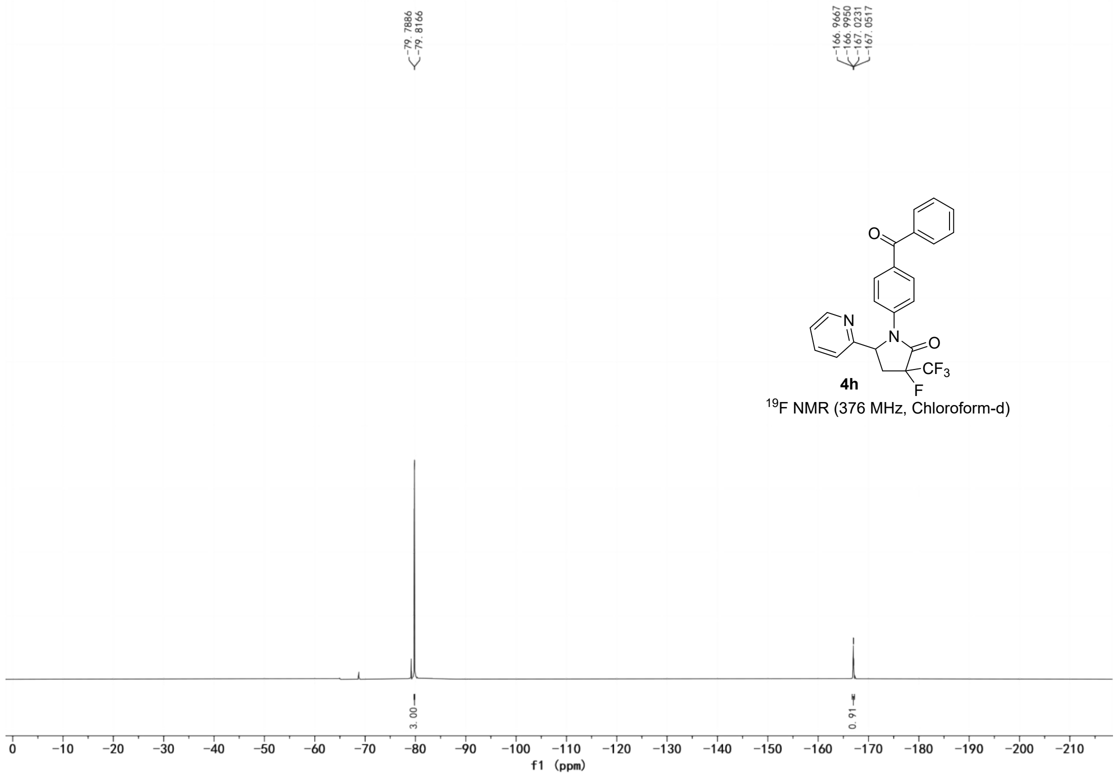


**4h**
$^{19}F$ NMR (376 MHz, Chloroform-d)

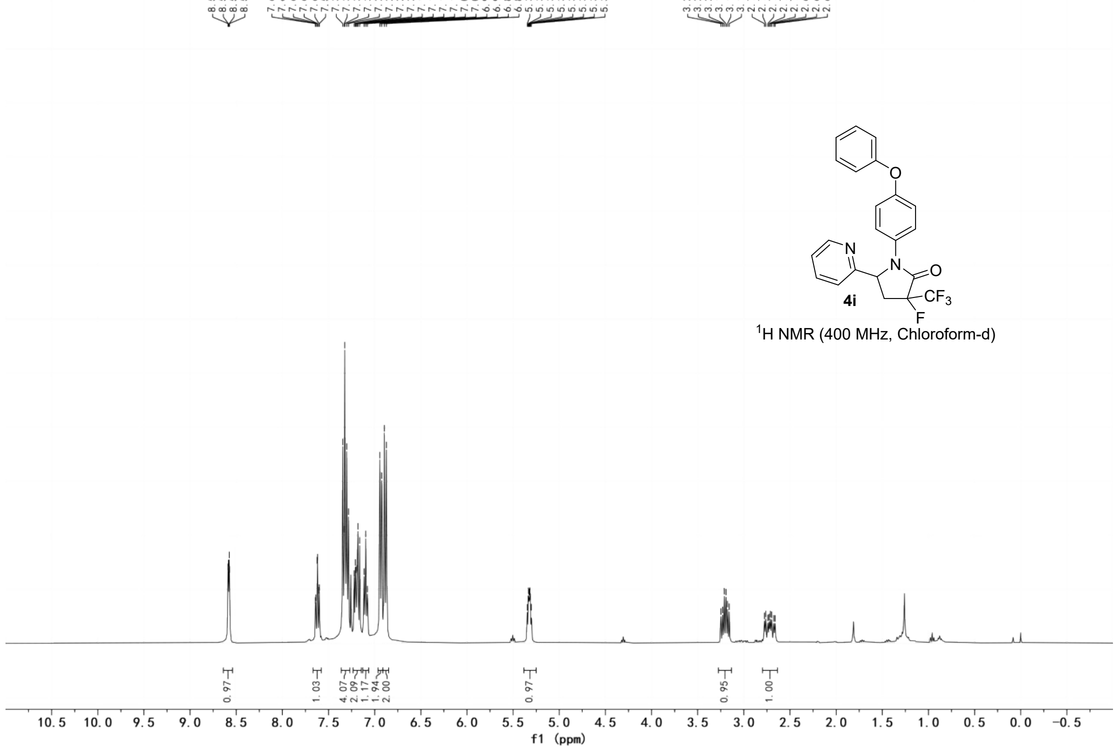

4i

$^{1}$H NMR (400 MHz, Chloroform-d)

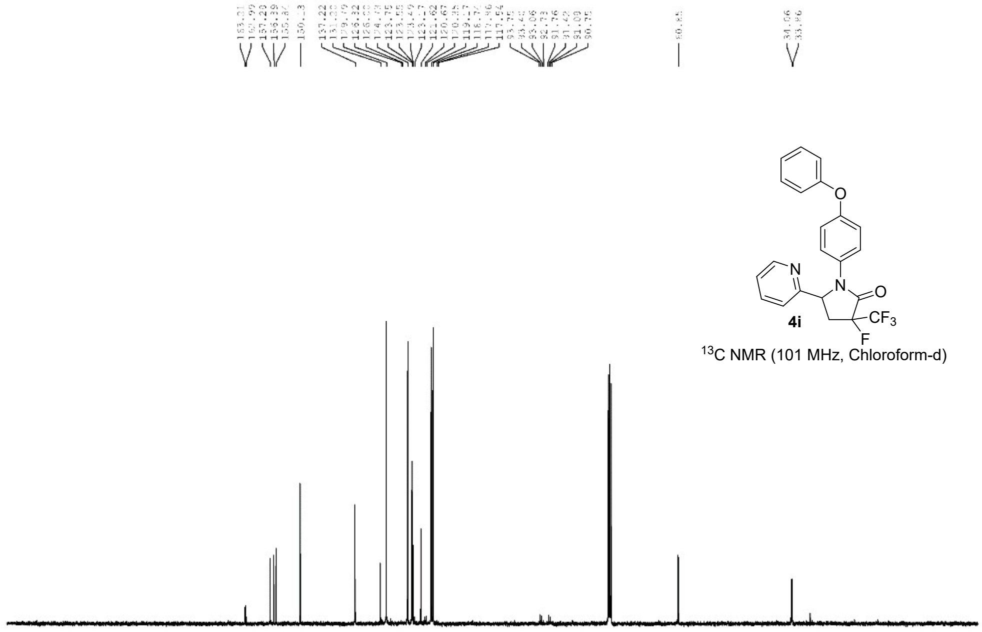

4i

$^{13}$C NMR (101 MHz, Chloroform-d)

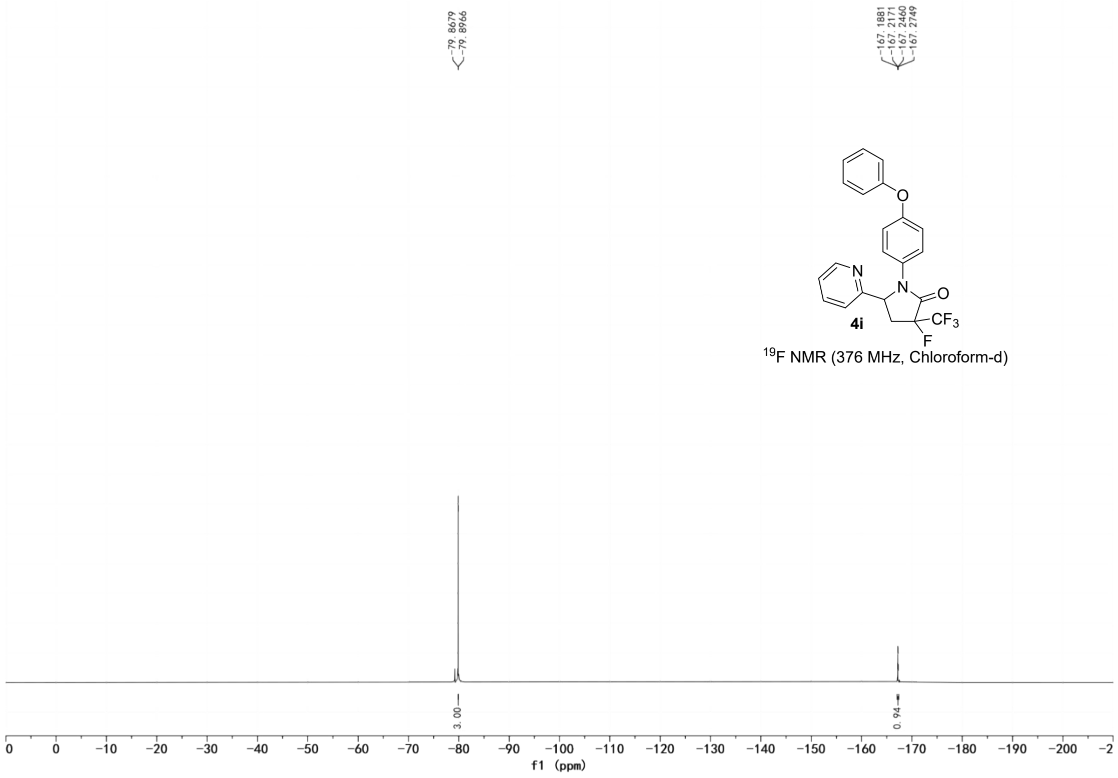
-79.8679
-79.8966
-167.1881
-167.2171
-167.2460
-167.2749
4i
¹⁹F NMR (376 MHz, Chloroform-d)
3.00
0.94
f1 (ppm)

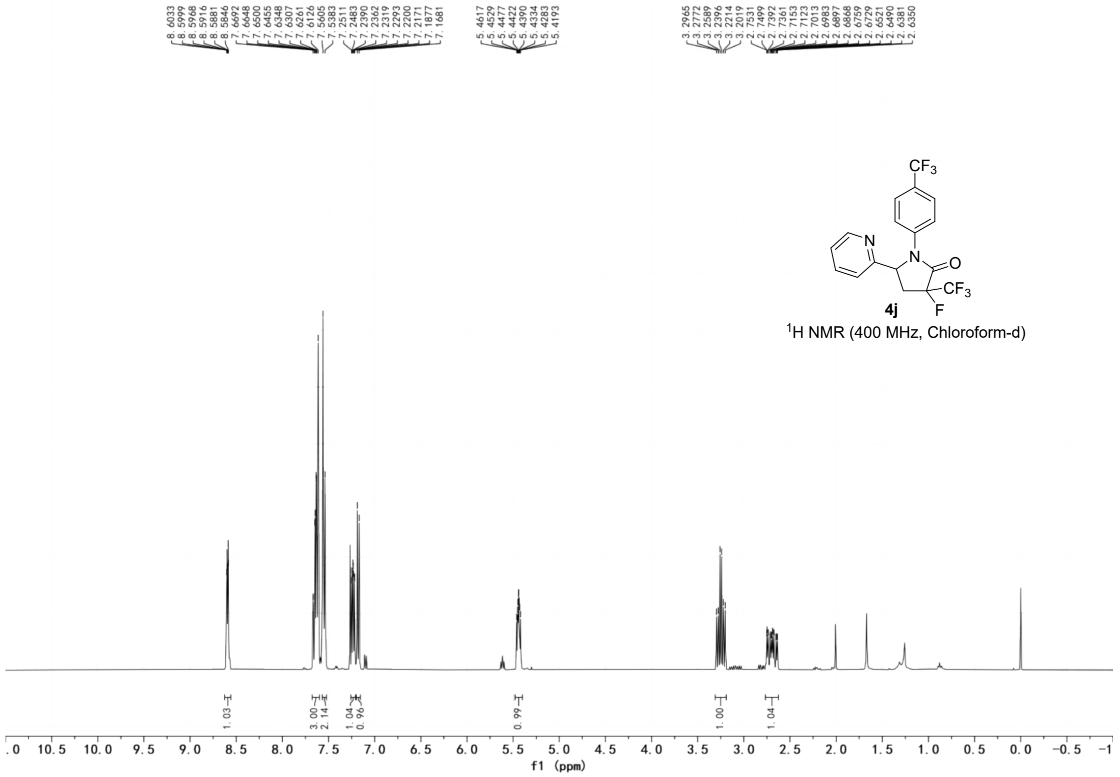
CF3
N
N
O
CF3
F
4j
1H NMR (400 MHz, Chloroform-d)
f1 (ppm)

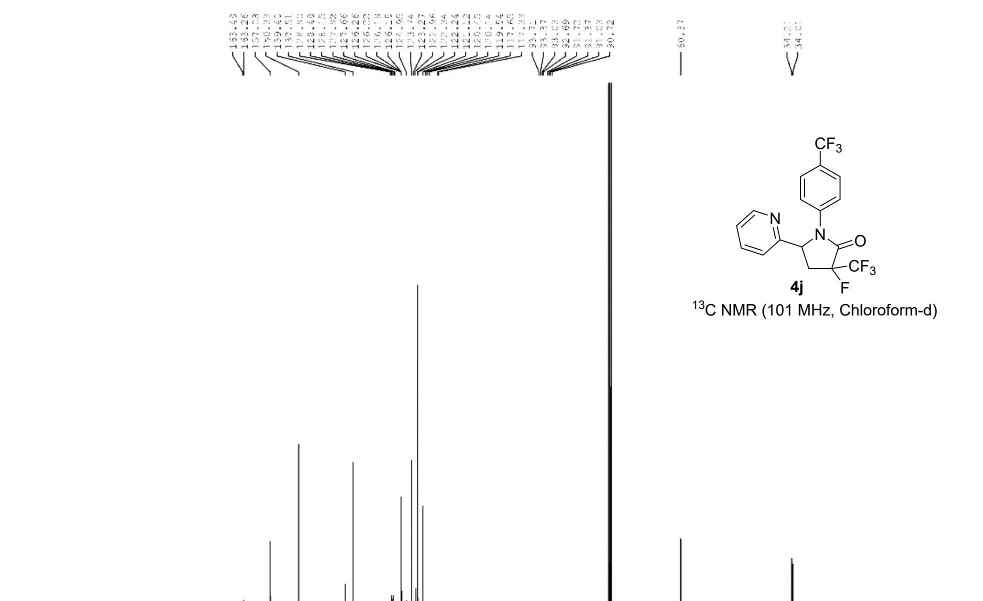
CF3
N
N
O
CF3
F
4j
13C NMR (101 MHz, Chloroform-d)
ppm

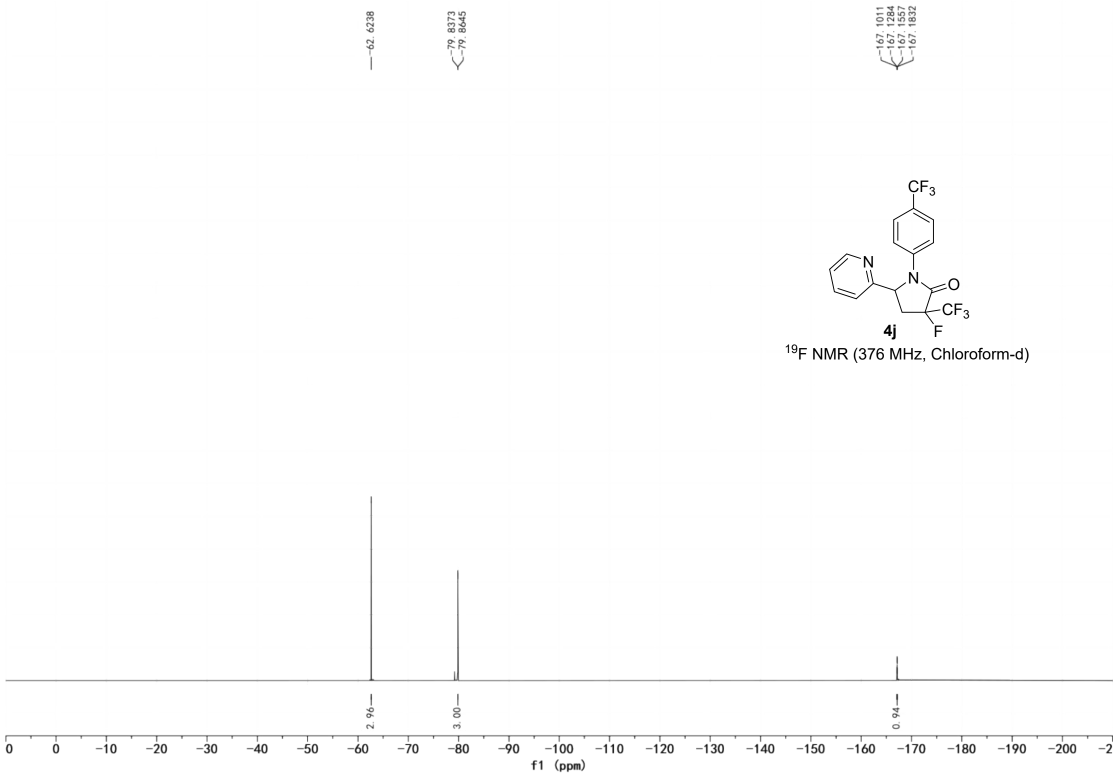
-62.6238
-79.8373
-79.8645
-167.1011
-167.1284
-167.1557
-167.1832
CF3
N
N
O
CF3
F
4j
19F NMR (376 MHz, Chloroform-d)
2.96
3.00
0.94
f1 (ppm)

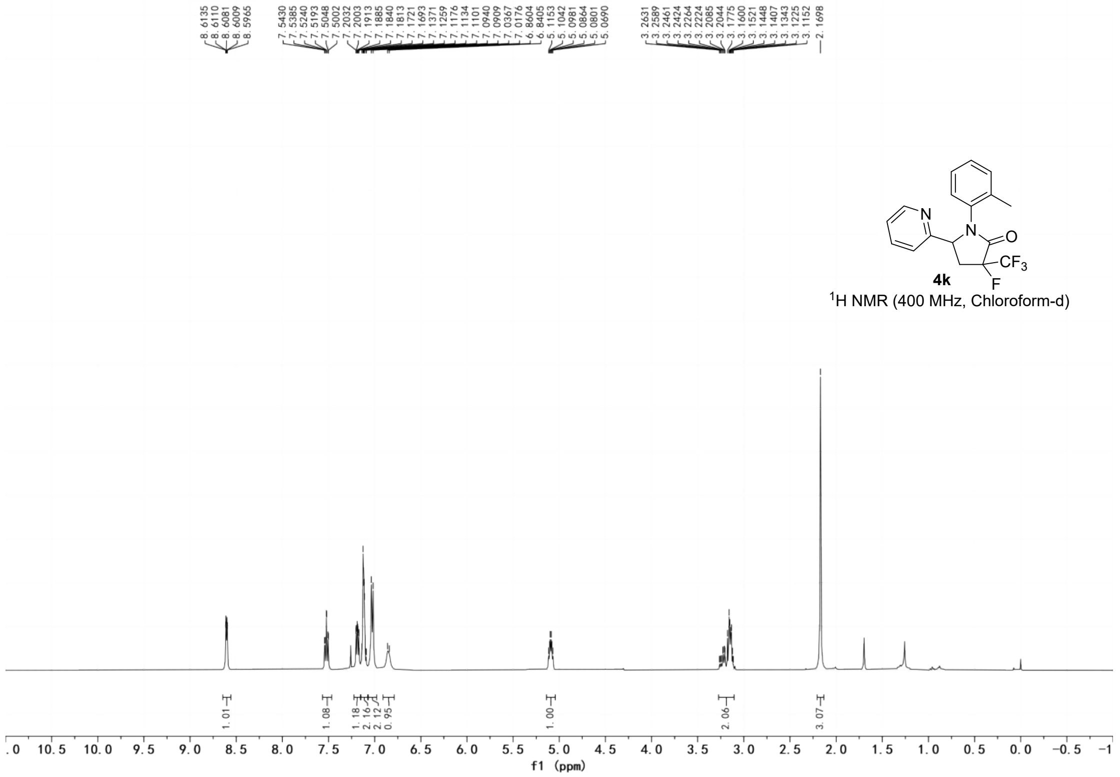
4k
1H NMR (400 MHz, Chloroform-d)
f1 (ppm)

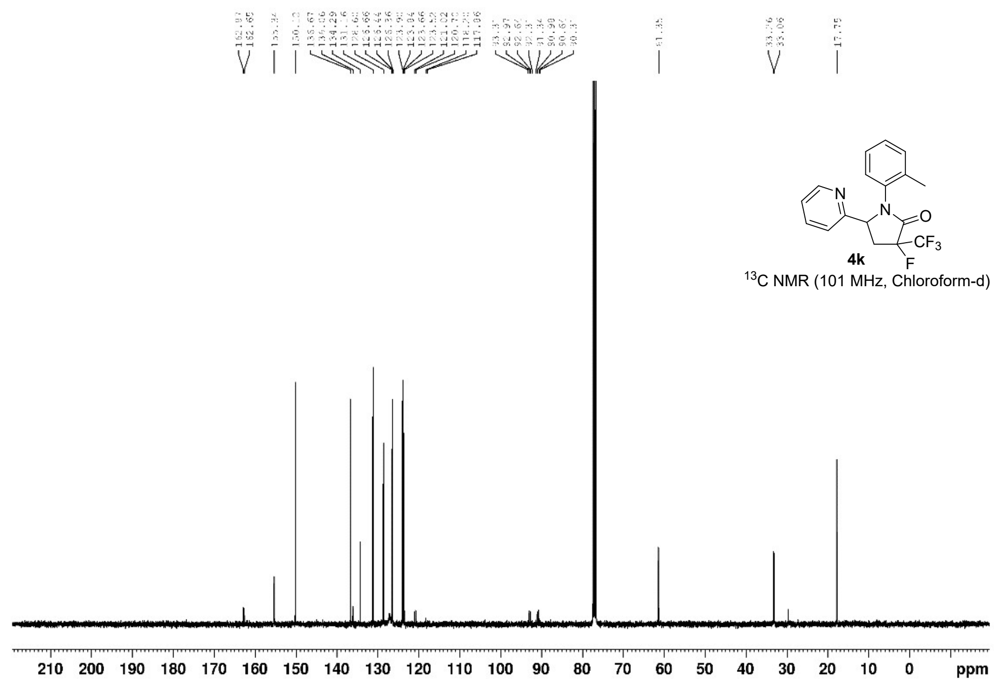
4k
13C NMR (101 MHz, Chloroform-d)
ppm

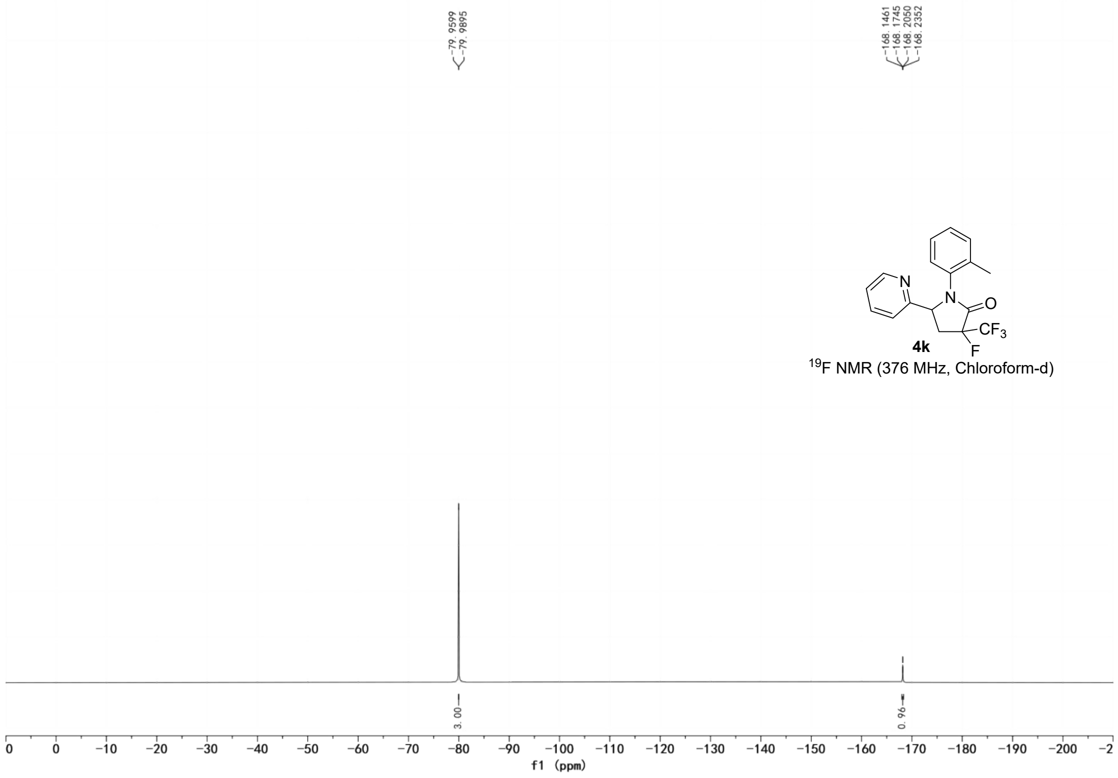
-79.9599
-79.9895
-168.1461
-168.1745
-168.2050
-168.2352
N
O
$CF_3$
F
4k
$^{19}$F NMR (376 MHz, Chloroform-d)
3.00
0.96
f1 (ppm)

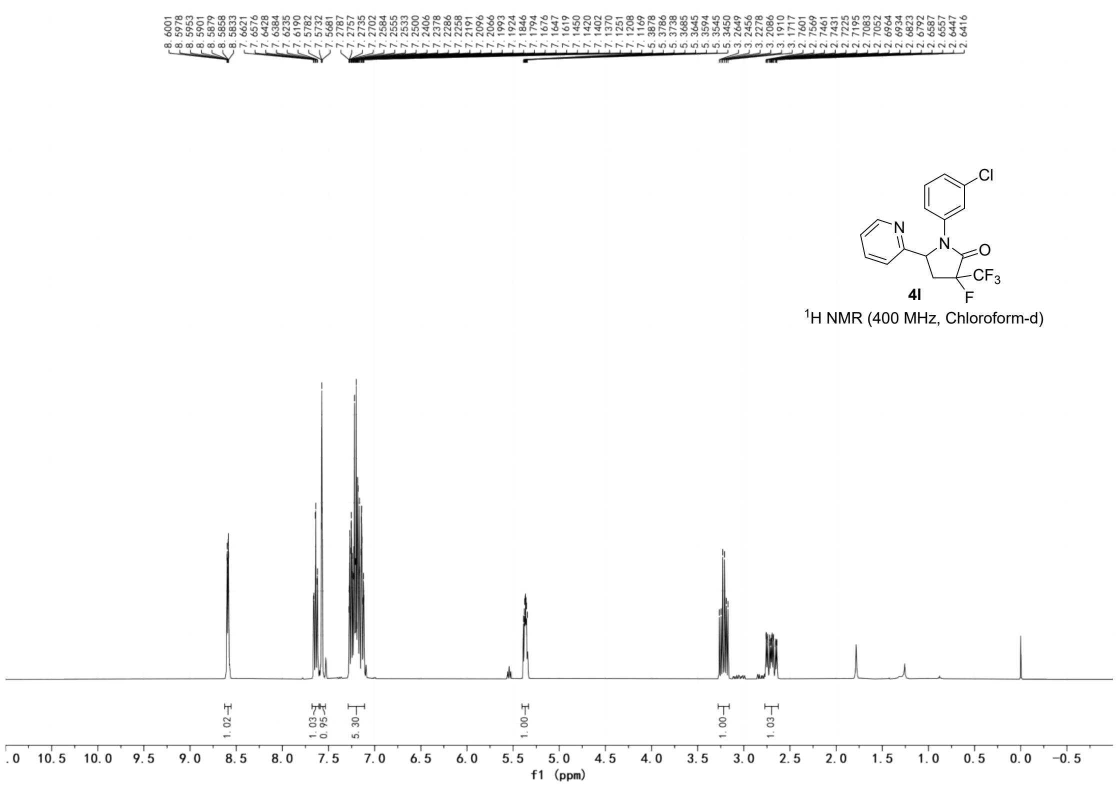
Cl
N
N
O
CF3
F
4l
1H NMR (400 MHz, Chloroform-d)
f1 (ppm)

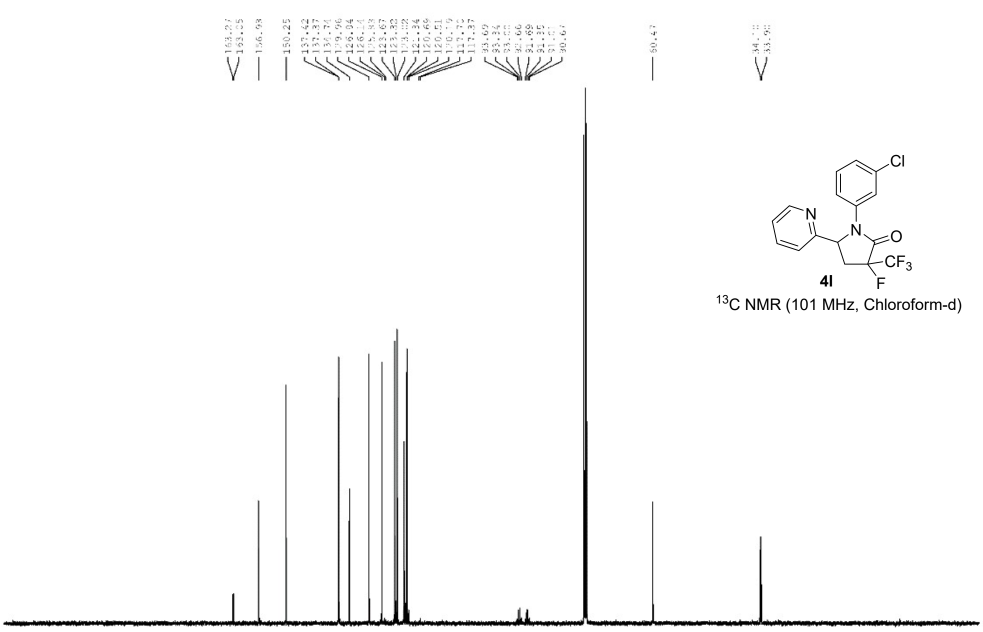
Cl
N
N
O
CF3
F
4l
13C NMR (101 MHz, Chloroform-d)
ppm

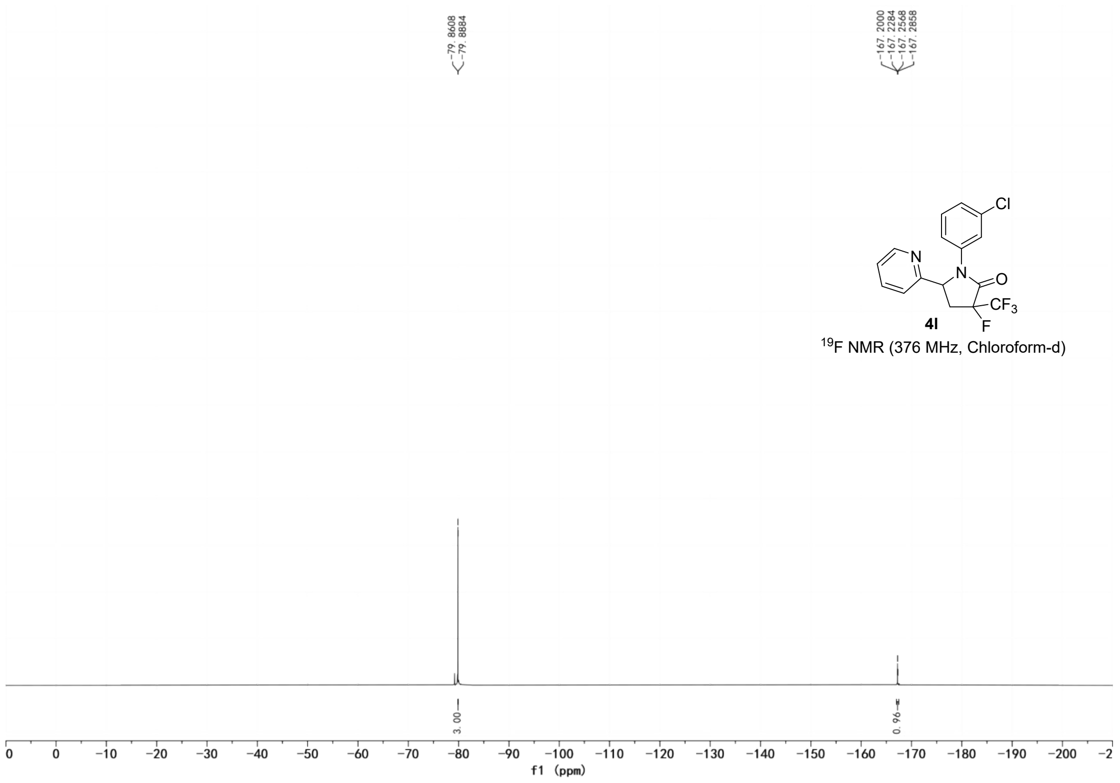

-79.8608
-79.8884
-167.2000
-167.2284
-167.2568
-167.2858
Cl
N
N
O
CF3
F
4l
19F NMR (376 MHz, Chloroform-d)
3.00
0.96
f1 (ppm)

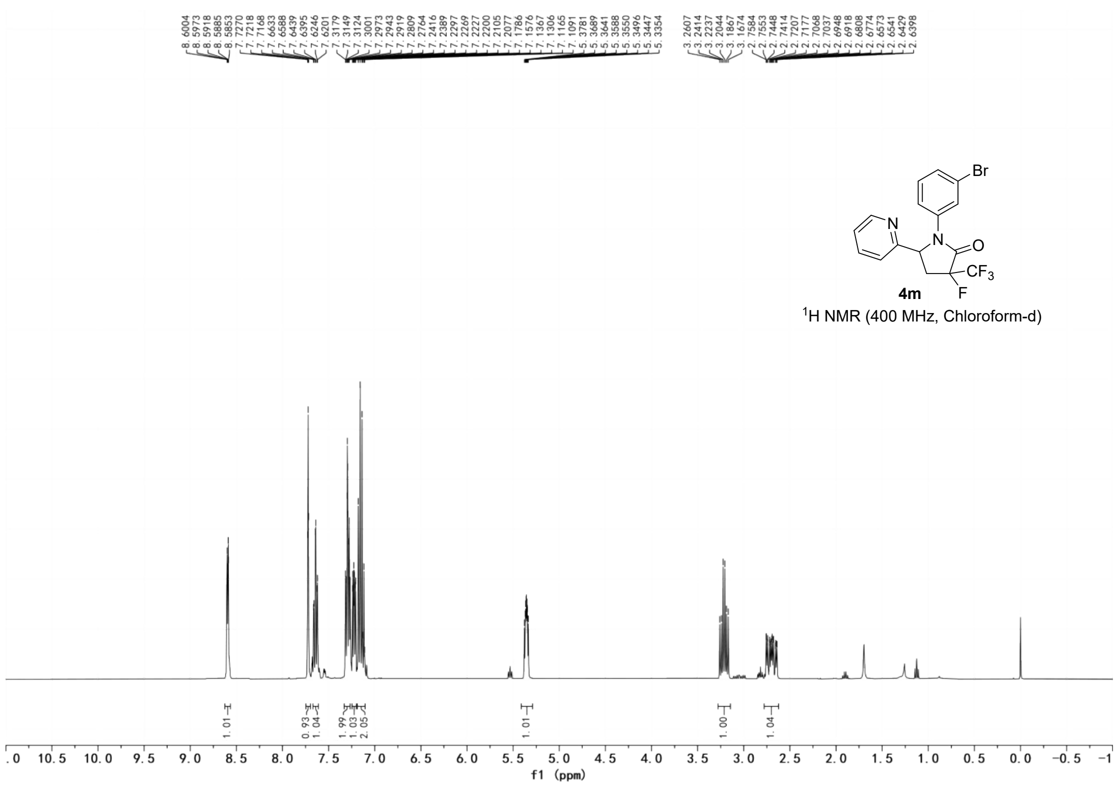
Br
N
O
CF3
F
4m
1H NMR (400 MHz, Chloroform-d)
f1 (ppm)

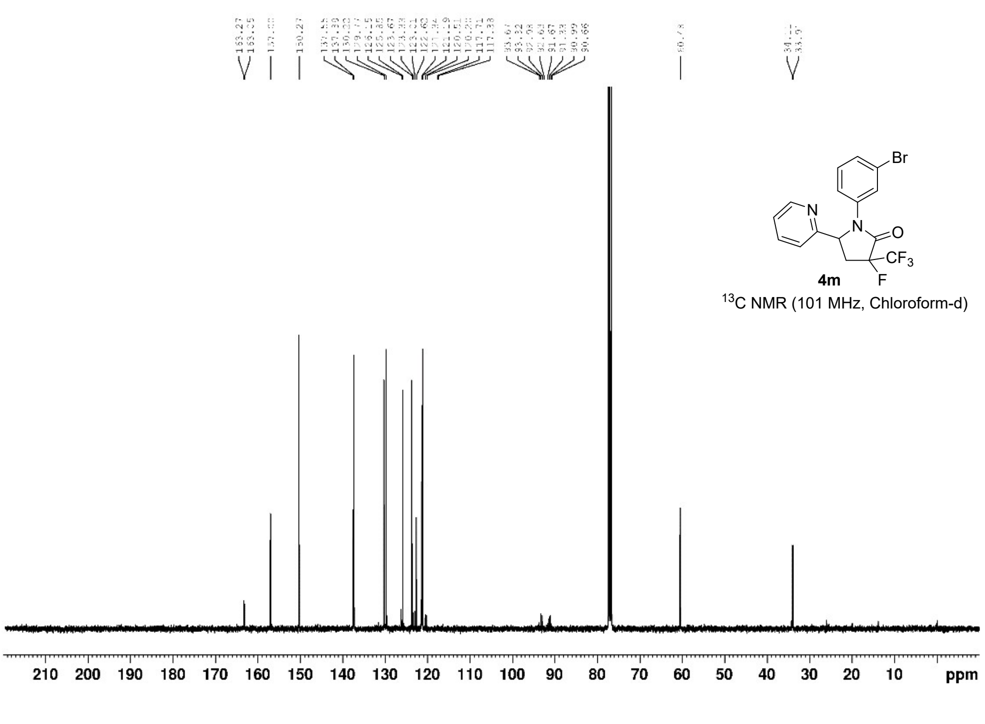
Br
N
O
CF3
F
4m
13C NMR (101 MHz, Chloroform-d)
ppm

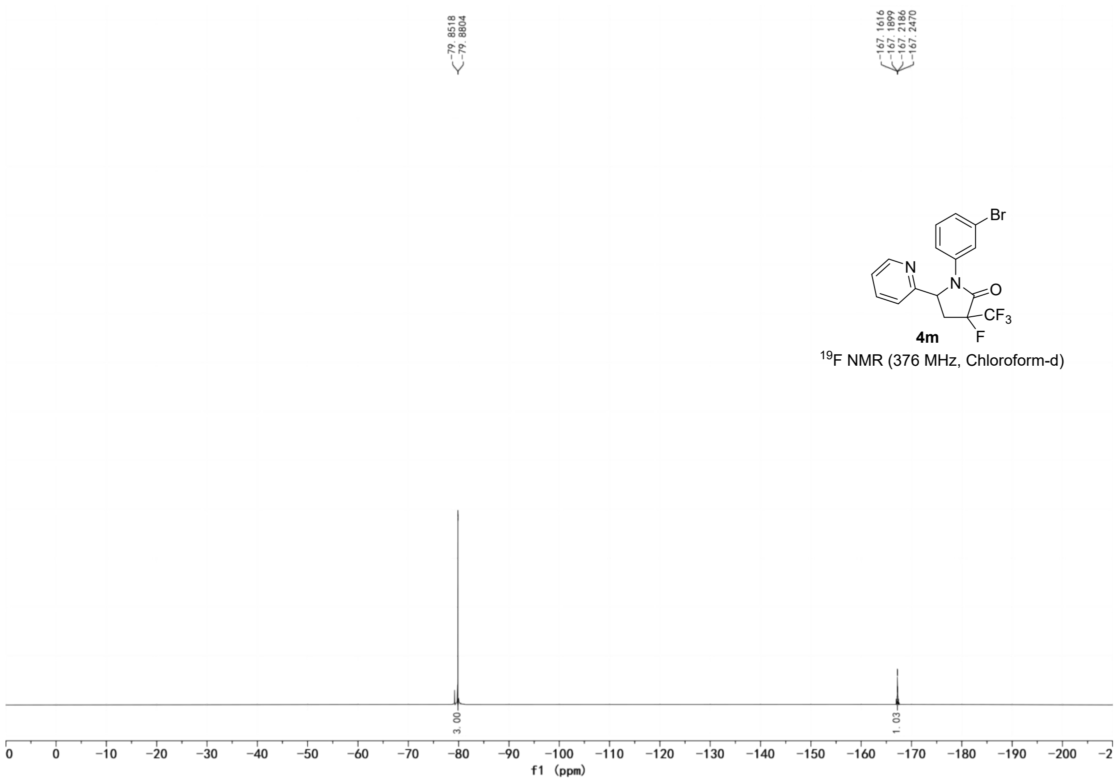

-79.8518
-79.8804
-167.1616
-167.1899
-167.2186
-167.2470
Br
N
N
O
CF3
F
4m
19F NMR (376 MHz, Chloroform-d)
3.00
1.03
f1 (ppm)

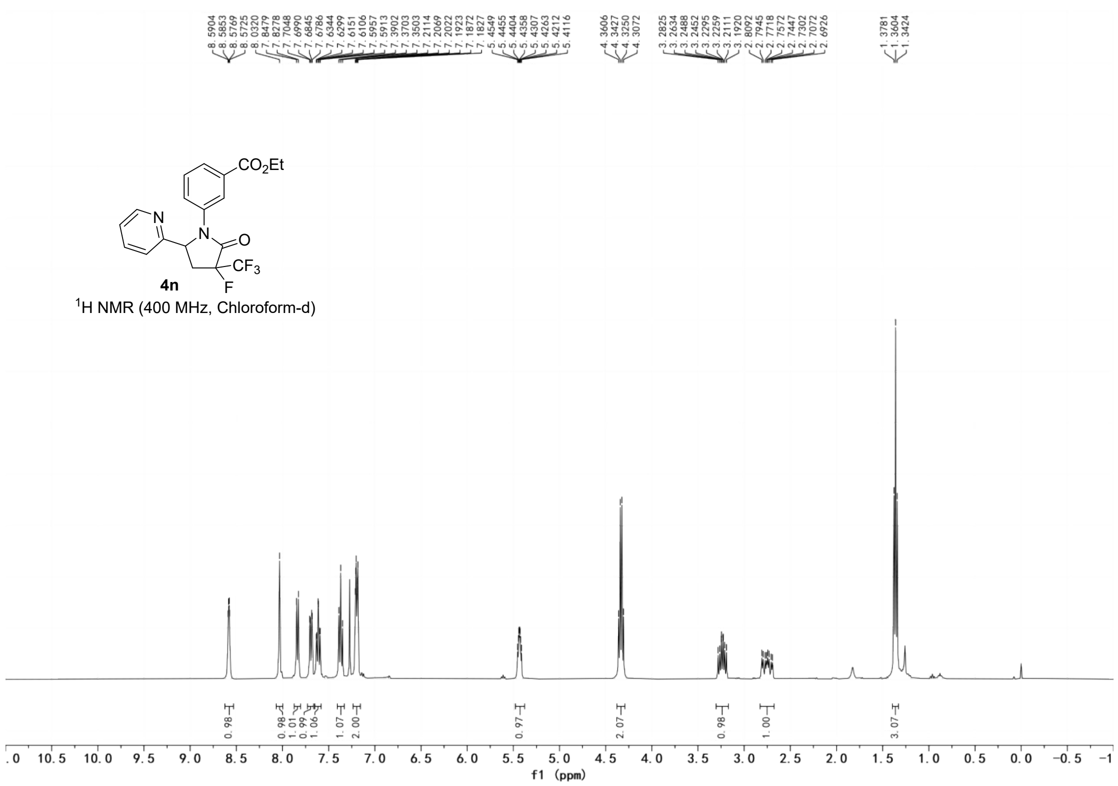
CO2Et
N
N
O
CF3
F
4n
1H NMR (400 MHz, Chloroform-d)
f1 (ppm)

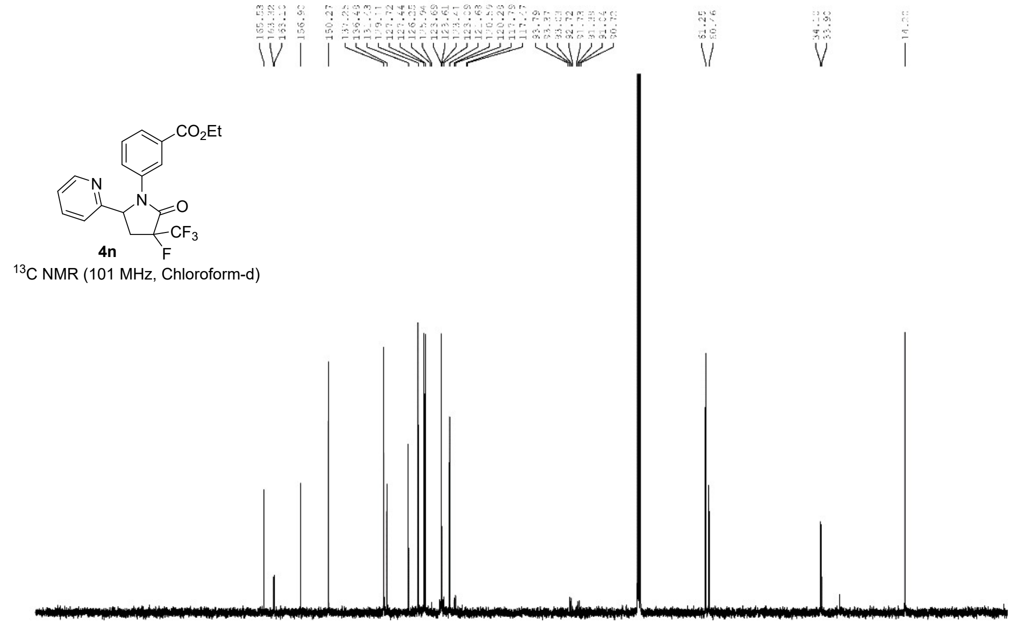
CO2Et
N
N
O
CF3
F
4n
13C NMR (101 MHz, Chloroform-d)
ppm

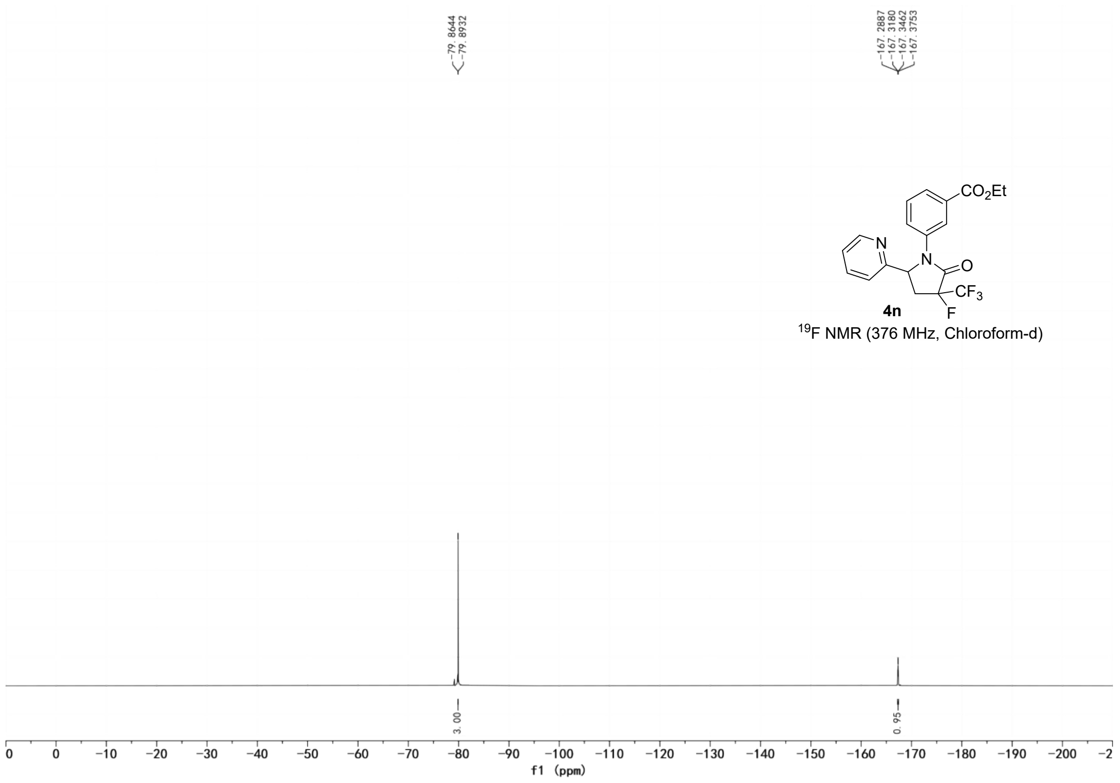

-79.8644
-79.8932
-167.2887
-167.3180
-167.3462
-167.3753
$CO_2Et$
N
N
O
$CF_3$
F
4n
$^{19}$F NMR (376 MHz, Chloroform-d)
3.00
0.95
0 0 −10 −20 −30 −40 −50 −60 −70 −80 −90 −100 −110 −120 −130 −140 −150 −160 −170 −180 −190 −200 −2
f1 (ppm)

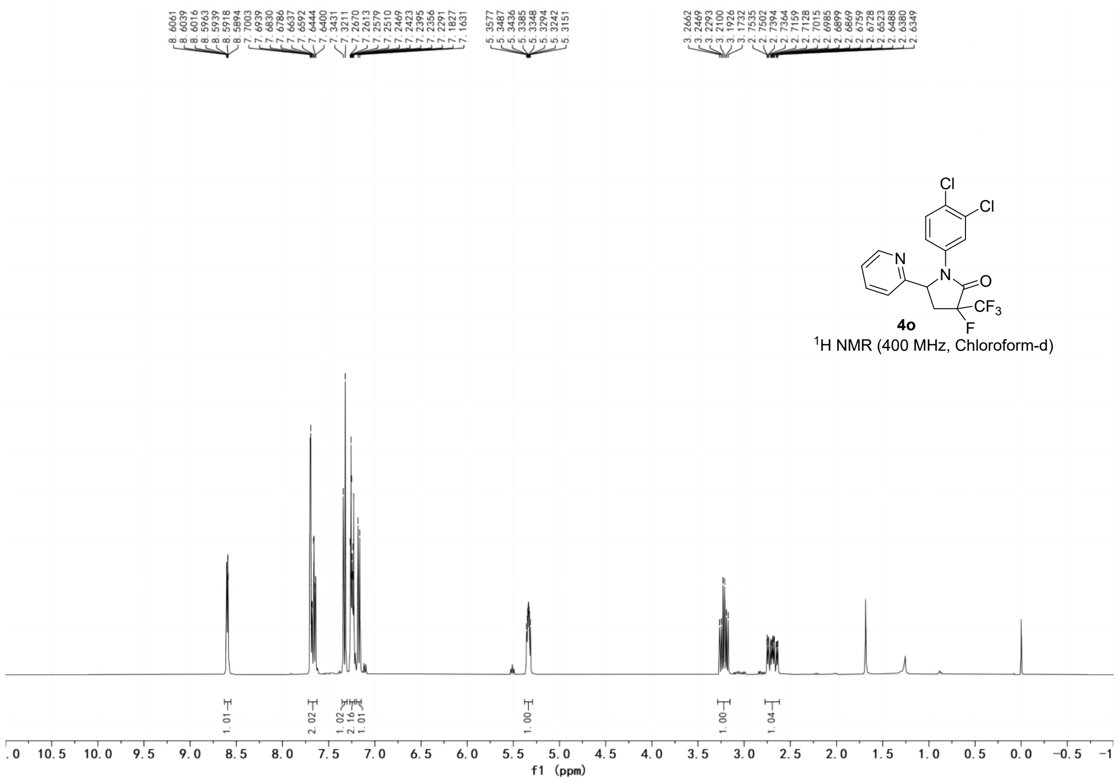
4o
$^{1}$H NMR (400 MHz, Chloroform-d)
f1 (ppm)

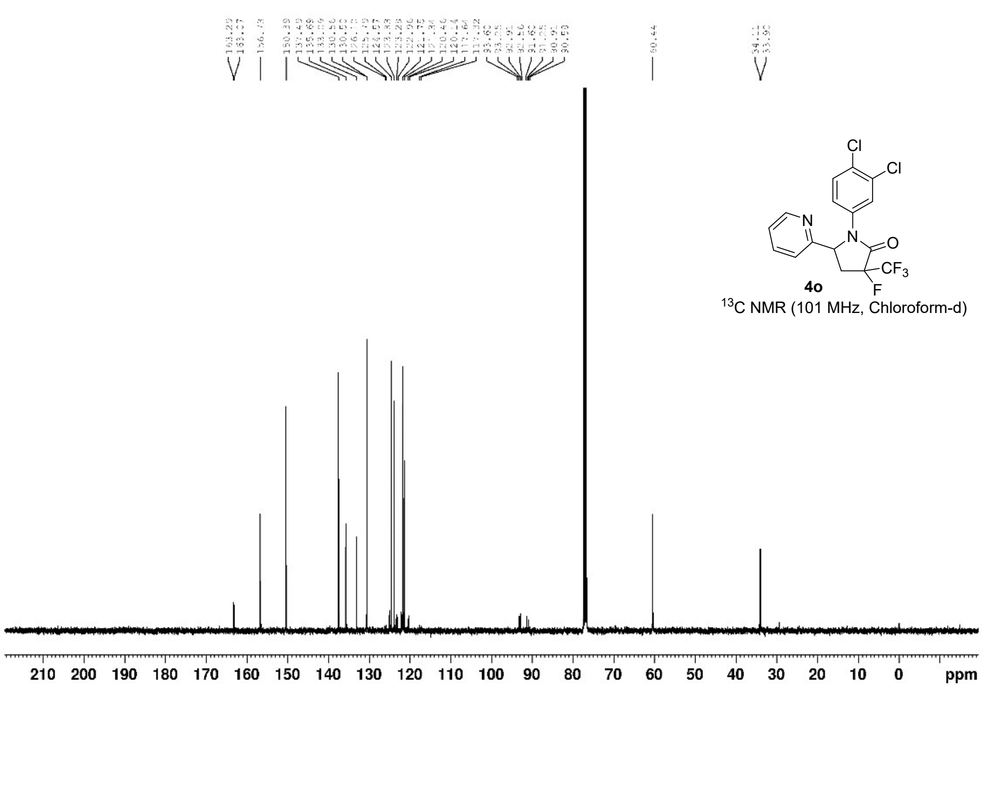
4o
$^{13}$C NMR (101 MHz, Chloroform-d)
ppm

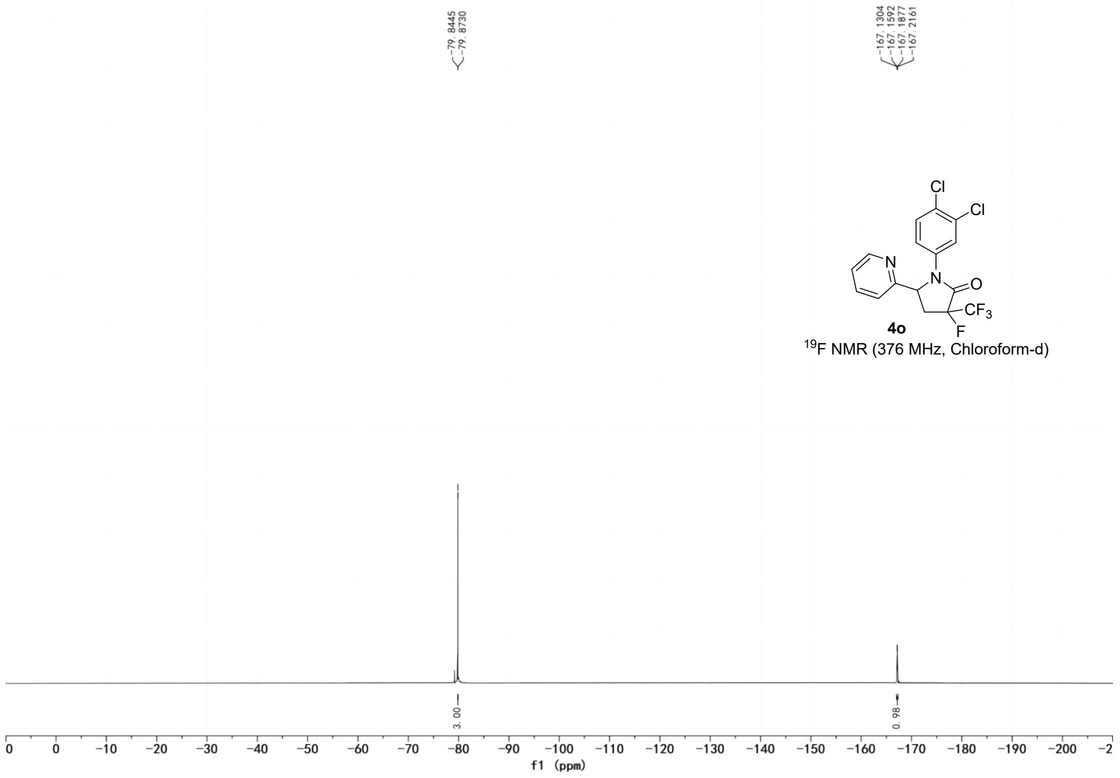

-79.8445
-79.8730
-167.1304
-167.1592
-167.1877
-167.2161
Cl
Cl
N
N
O
CF3
F
4o
19F NMR (376 MHz, Chloroform-d)
3.00
0.98
0 0 −10 −20 −30 −40 −50 −60 −70 −80 −90 −100 −110 −120 −130 −140 −150 −160 −170 −180 −190 −200 −2
f1 (ppm)

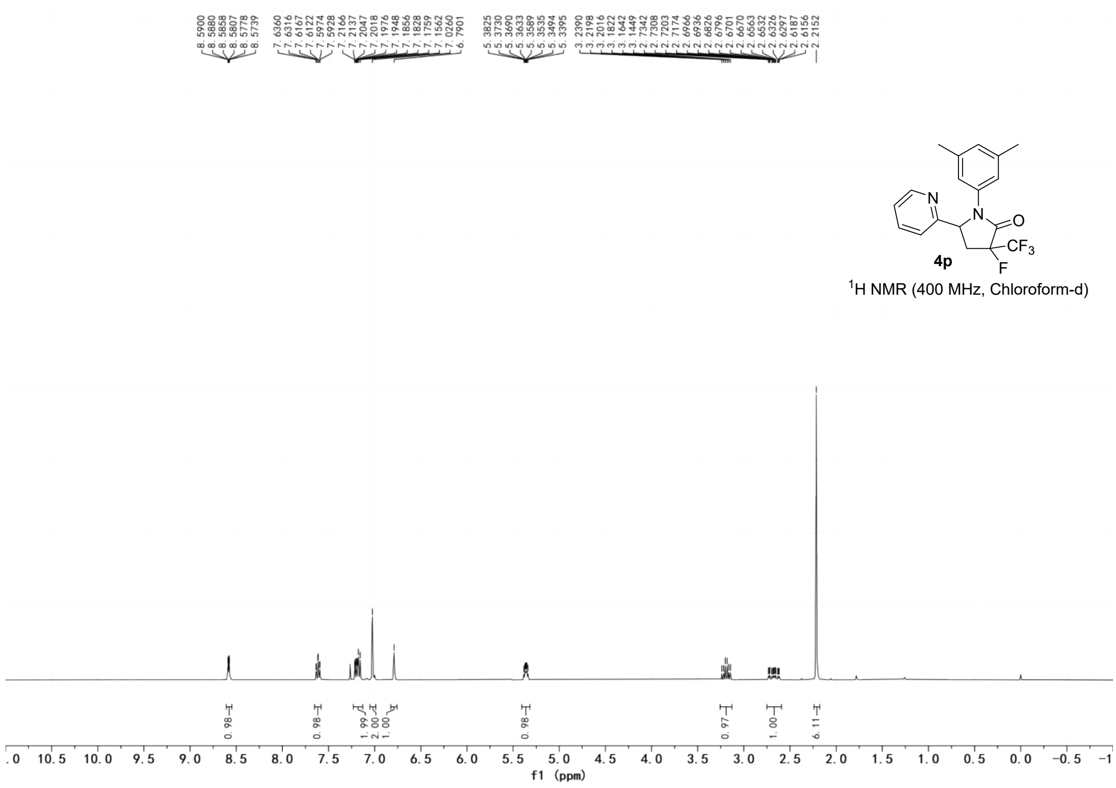
8.5900 8.5880 8.5858 8.5807 8.5778 8.5739
7.6360 7.6316 7.6167 7.6122 7.5974 7.5928 7.2166 7.2137 7.2047 7.2018 7.1976 7.1948 7.1856 7.1828 7.1759 7.1562 7.0260 6.7901
5.3825 5.3730 5.3690 5.3633 5.3589 5.3535 5.3494 5.3395
3.2390 3.2198 3.2016 3.1822 3.1642 3.1449 2.7342 2.7308 2.7203 2.7174 2.6966 2.6936 2.6826 2.6796 2.6701 2.6670 2.6563 2.6532 2.6326 2.6297 2.6187 2.6156 2.2152
N
O
N
CF3
F
4p
1H NMR (400 MHz, Chloroform-d)
0.98 0.98 1.99 2.00 1.00 0.98 0.97 1.00 6.11
f1 (ppm)

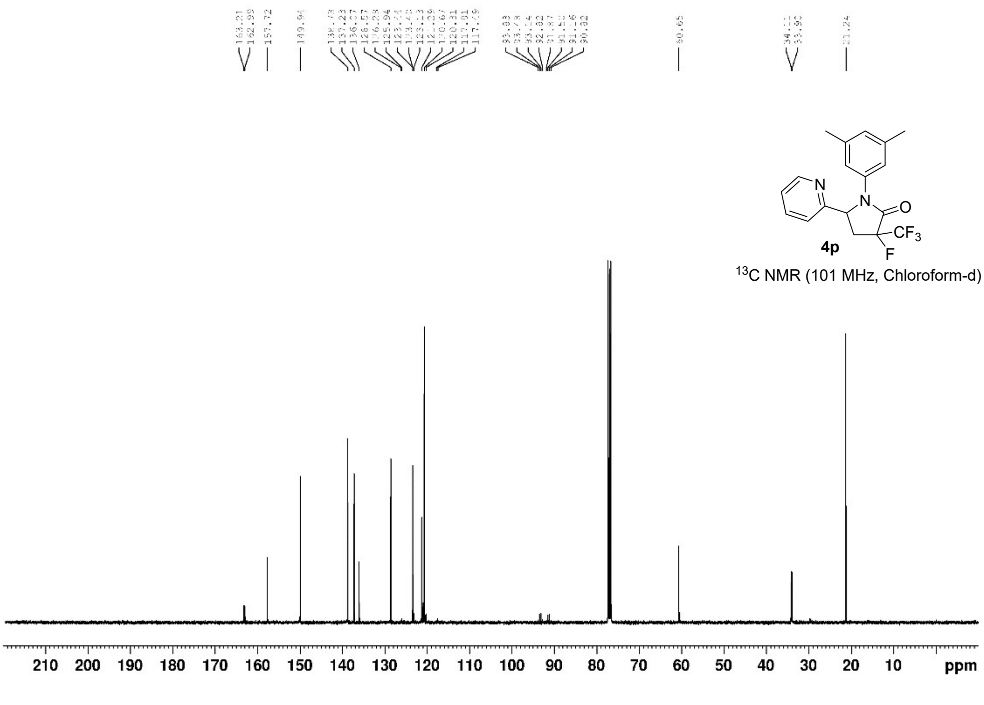
N
O
N
CF3
F
4p
13C NMR (101 MHz, Chloroform-d)
210 200 190 180 170 160 150 140 130 120 110 100 90 80 70 60 50 40 30 20 10 ppm

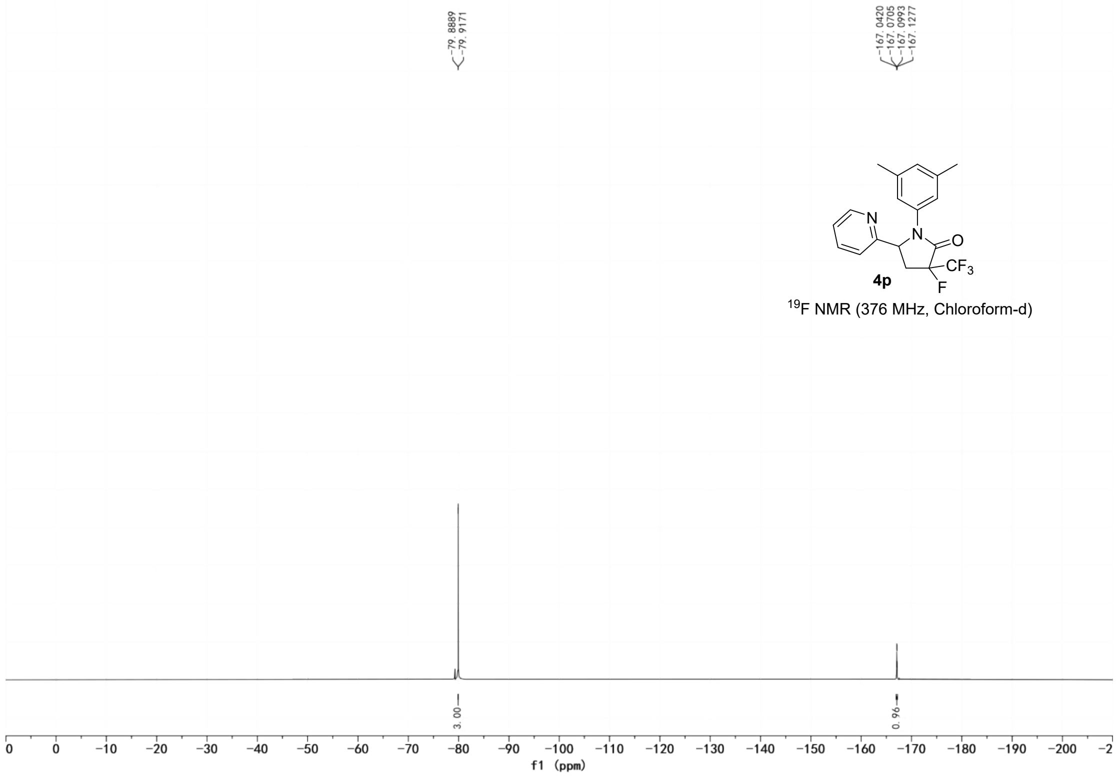

-79.8889
-79.9171
-167.0420
-167.0705
-167.0993
-167.1277
N
N
O
CF3
F
4p
19F NMR (376 MHz, Chloroform-d)
3.00
0.96
f1 (ppm)

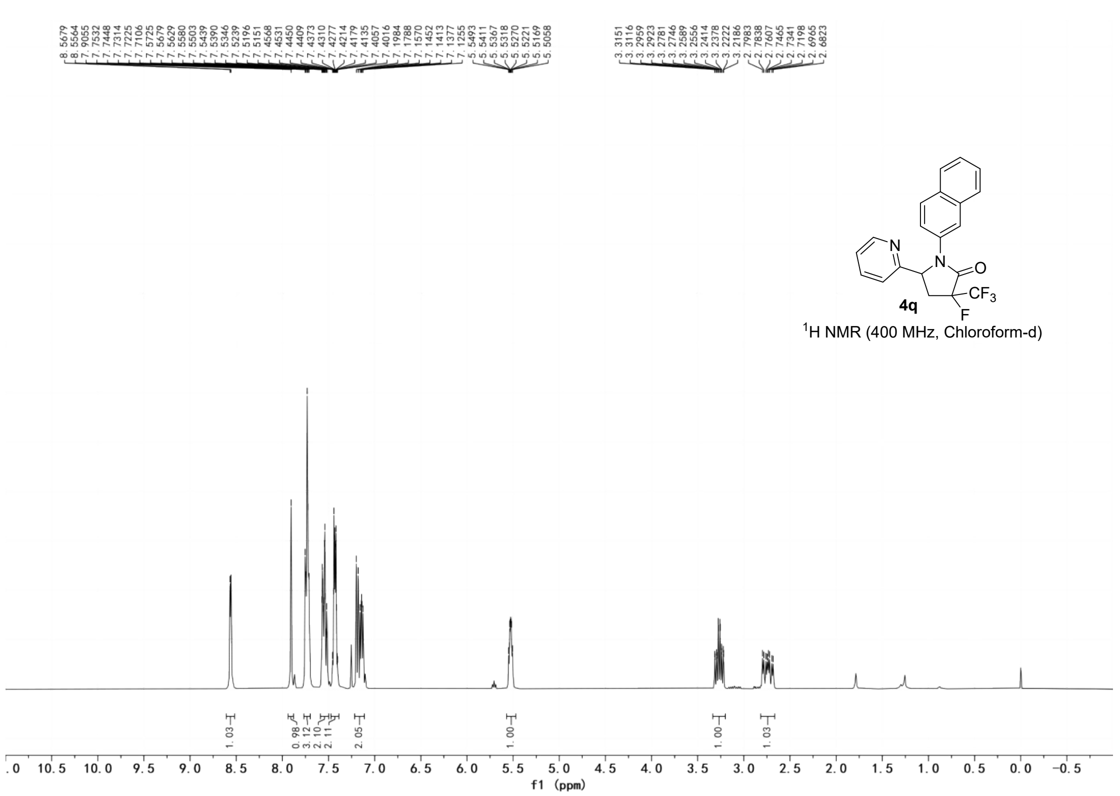

4q

$^{1}H$ NMR (400 MHz, Chloroform-d)

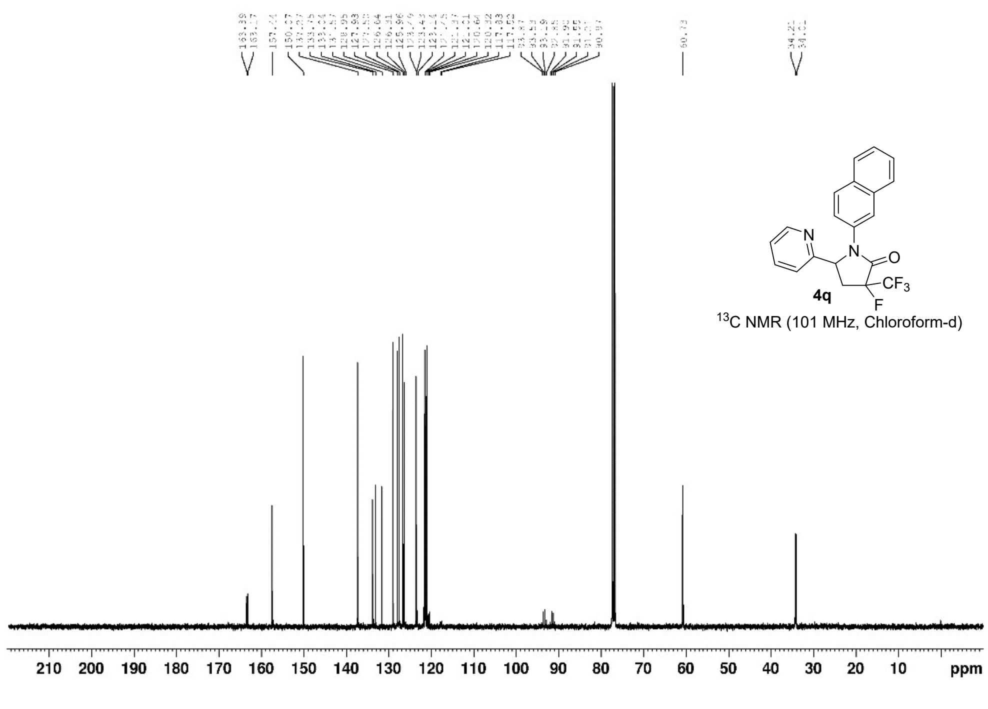

4q

$^{13}C$ NMR (101 MHz, Chloroform-d)

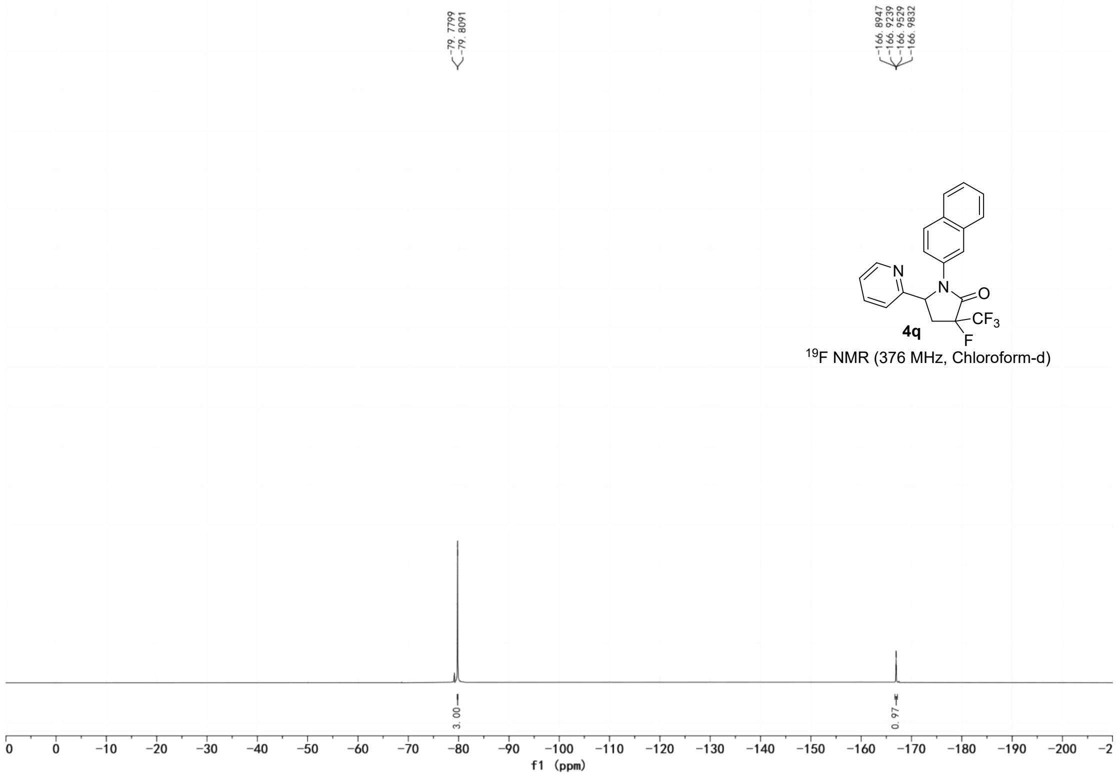

-79.7799
-79.8091
-166.8947
-166.9239
-166.9529
-166.9832
N
N
O
CF3
F
4q
19F NMR (376 MHz, Chloroform-d)
3.00
0.97
f1 (ppm)

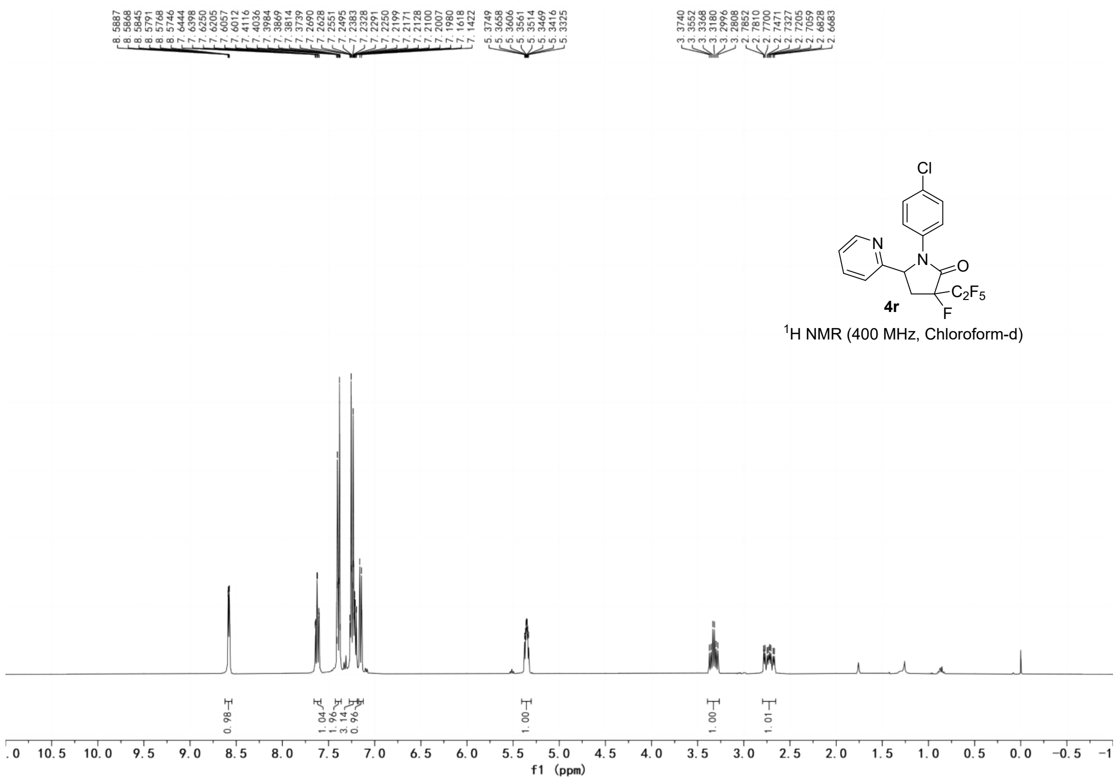
8.5887
8.5868
8.5845
8.5791
8.5768
8.5746
7.6444
7.6398
7.6250
7.6205
7.6057
7.6012
7.4116
7.4036
7.3984
7.3869
7.3814
7.3739
7.2690
7.2628
7.2551
7.2495
7.2383
7.2328
7.2291
7.2250
7.2199
7.2171
7.2128
7.2100
7.2007
7.1980
7.1618
7.1422
5.3749
5.3658
5.3606
5.3561
5.3514
5.3469
5.3416
5.3325
3.3740
3.3552
3.3368
3.3180
3.2996
3.2808
2.7852
2.7810
2.7700
2.7471
2.7327
2.7205
2.7059
2.6828
2.6683
Cl
N
N
O
C2F5
F
4r
1H NMR (400 MHz, Chloroform-d)
0.98
1.04
1.96
3.14
0.96
1.00
1.00
1.01
f1 (ppm)

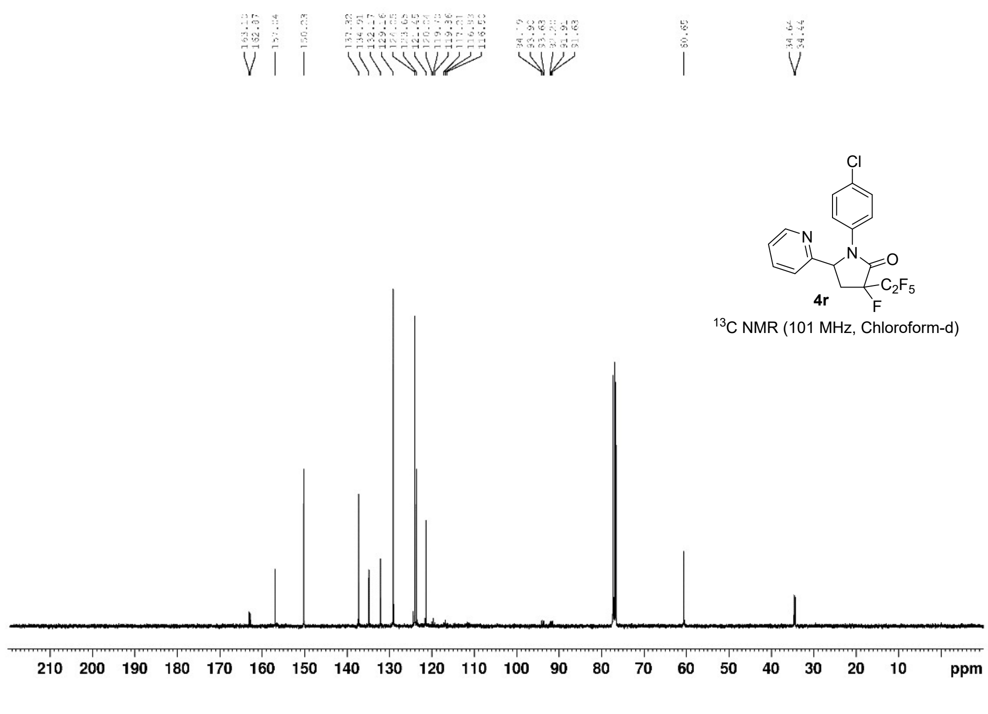
60.65
34.64
34.44
Cl
N
N
O
C2F5
F
4r
13C NMR (101 MHz, Chloroform-d)
ppm

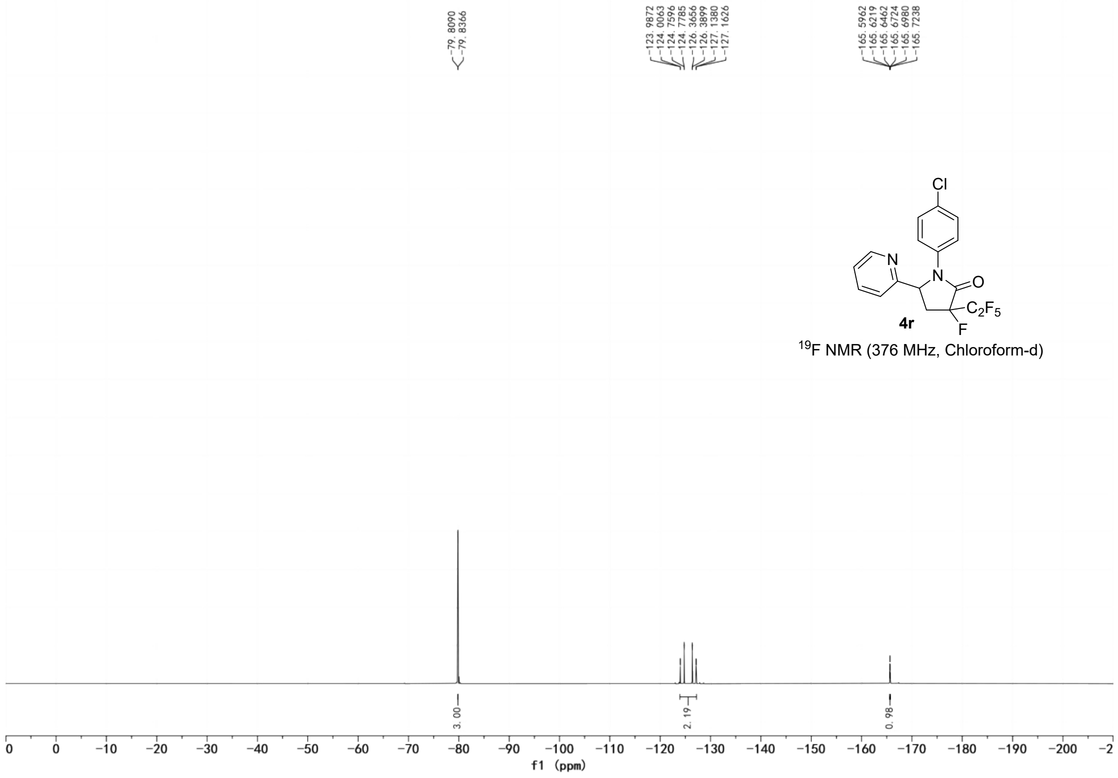
-79.8090
-79.8366
-123.9872
-124.0063
-124.7596
-124.7785
-126.3656
-126.3899
-127.1380
-127.1626
-165.5962
-165.6219
-165.6462
-165.6724
-165.6980
-165.7238
Cl
N
N
O
$C_2F_5$
F
4r
$^{19}$F NMR (376 MHz, Chloroform-d)
3.00
2.19
0.98
f1 (ppm)

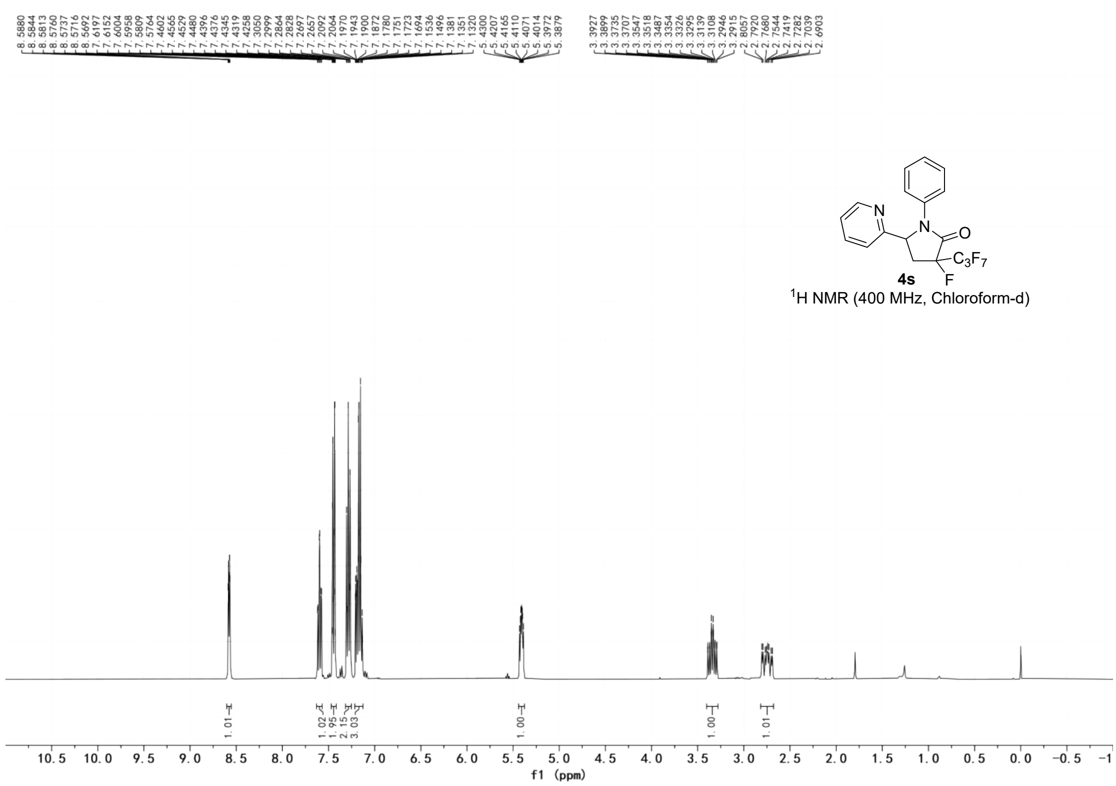
4s
$^{1}$H NMR (400 MHz, Chloroform-d)
f1 (ppm)

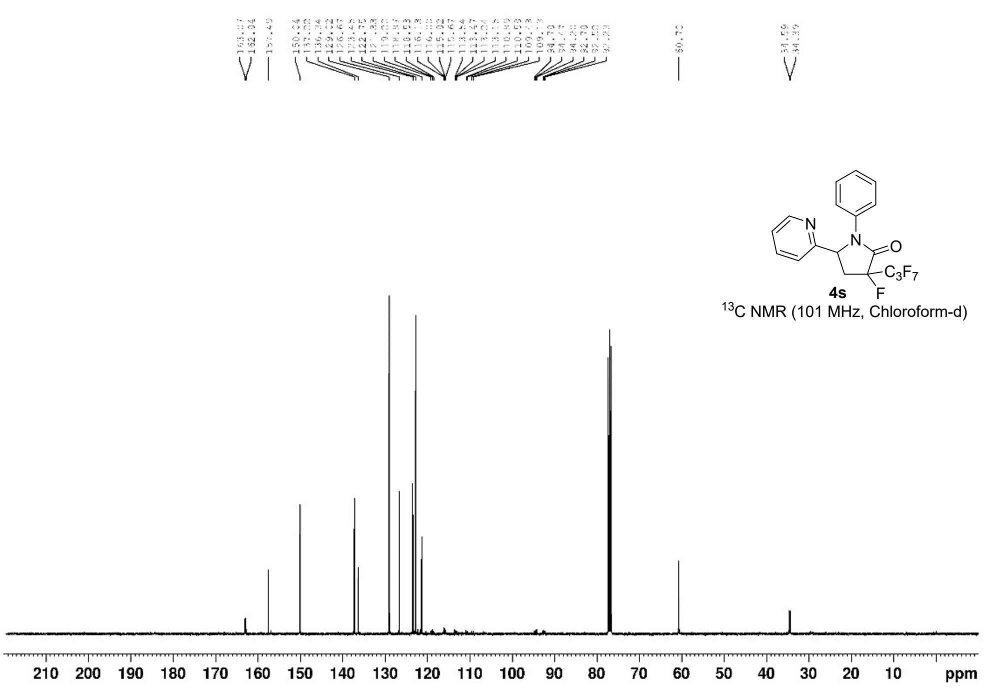
4s
$^{13}$C NMR (101 MHz, Chloroform-d)
ppm

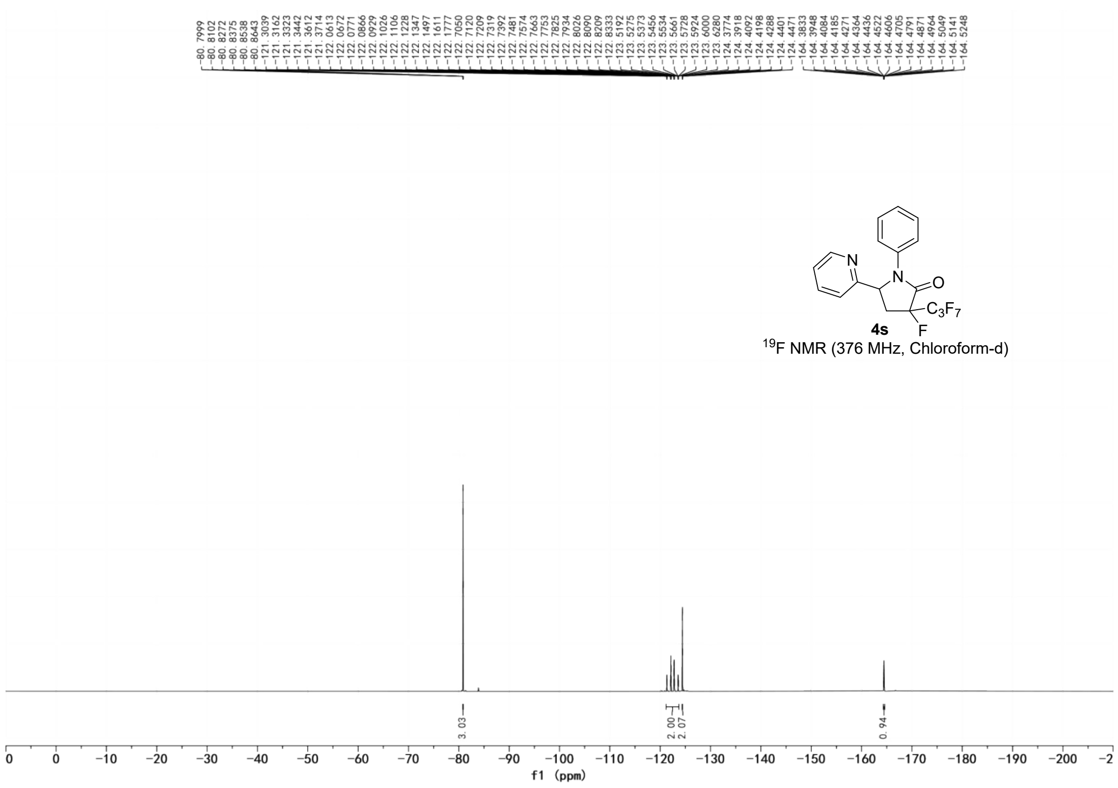

4s
19F NMR (376 MHz, Chloroform-d)
f1 (ppm)

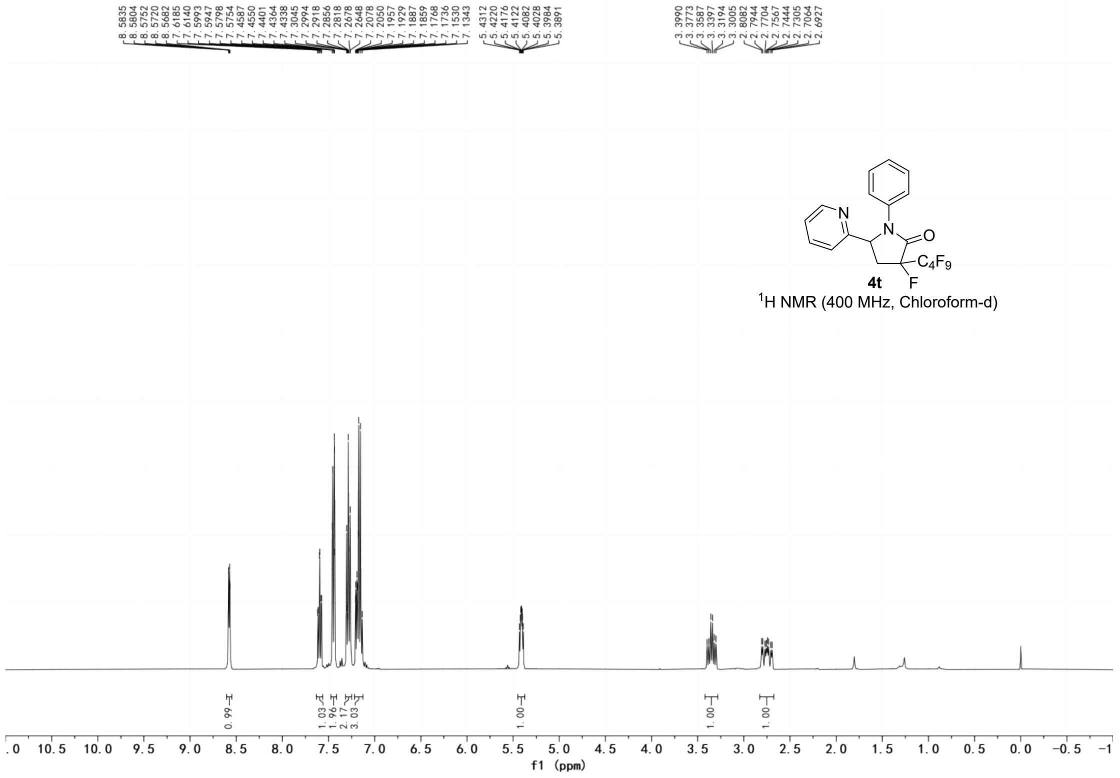

**4t**
$^1H$ NMR (400 MHz, Chloroform-d)

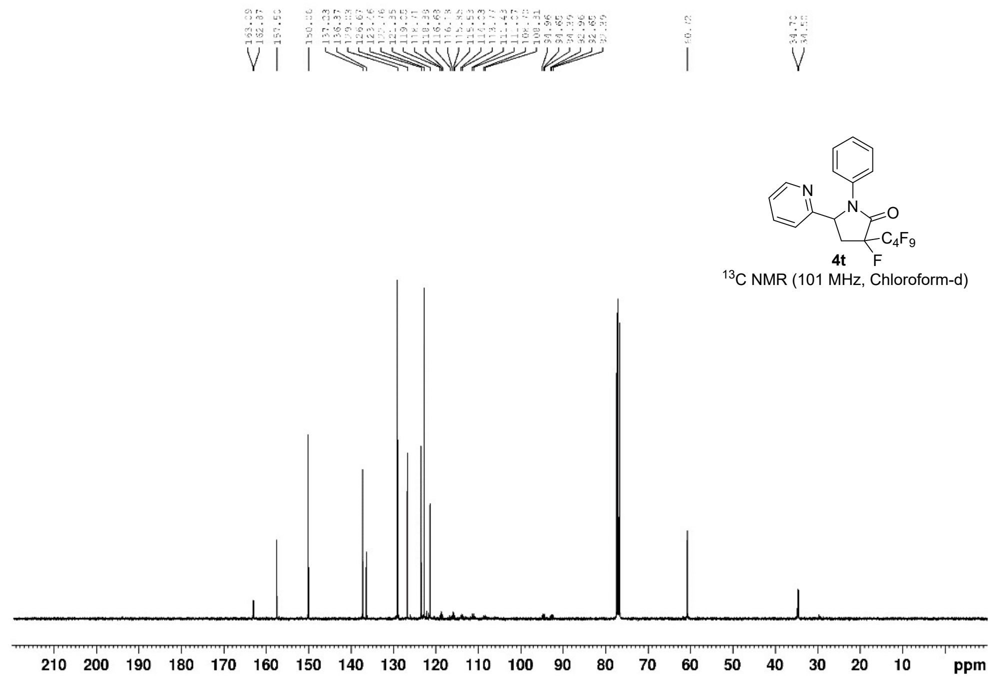

**4t**
$^{13}C$ NMR (101 MHz, Chloroform-d)

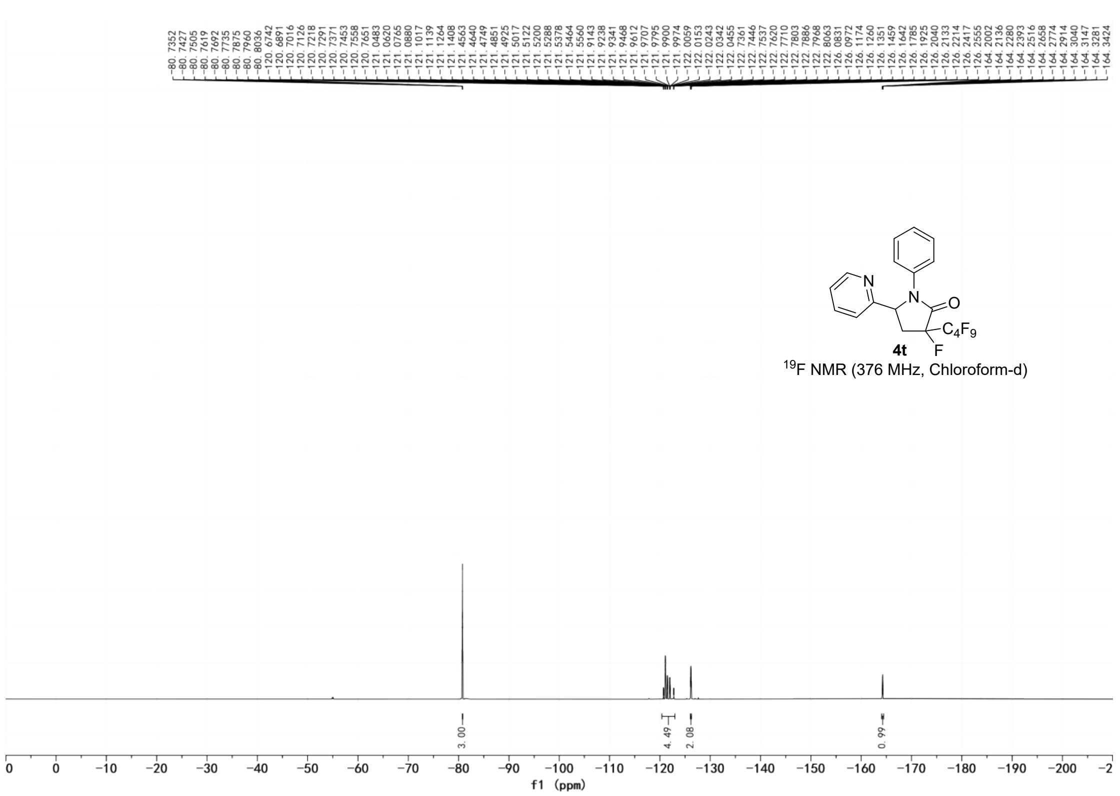

4t
$^{19}$F NMR (376 MHz, Chloroform-d)
f1 (ppm)


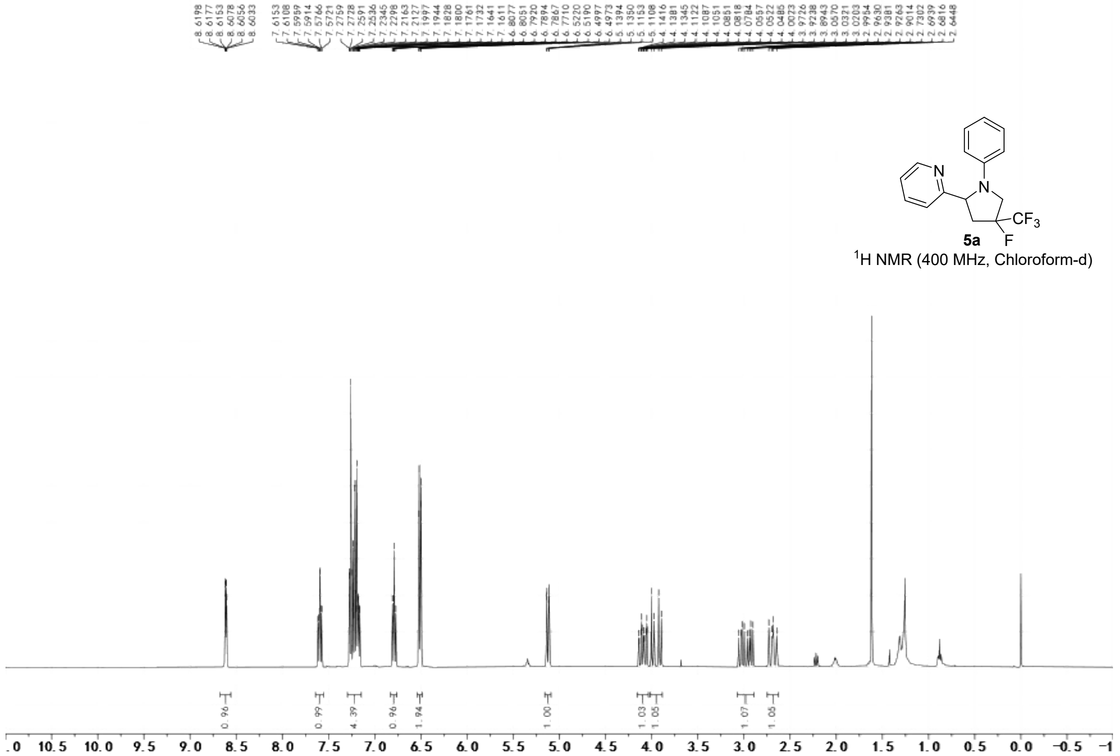

5a
$^{1}$H NMR (400 MHz, Chloroform-d)
f1 (ppm)

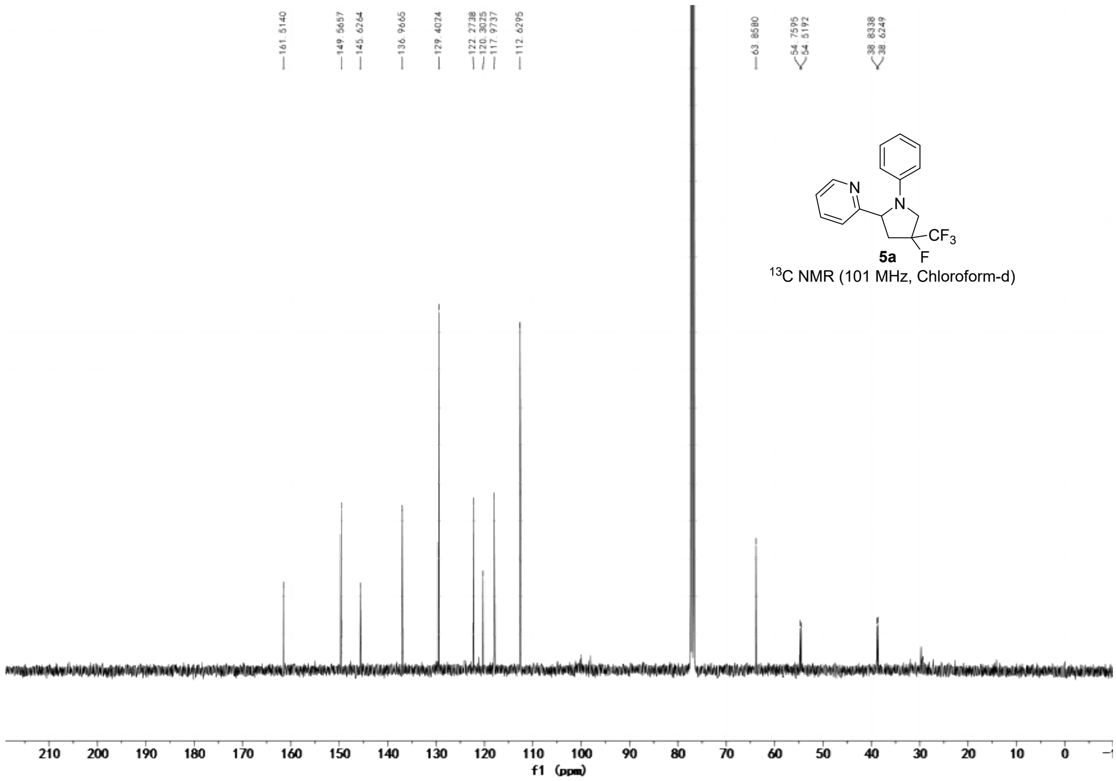
161.5140
149.5657
145.6264
136.9665
129.4024
122.2738
120.3025
117.9737
112.6295
63.8580
54.7595
54.5192
38.8338
38.6249
N
N
CF3
F
5a
13C NMR (101 MHz, Chloroform-d)
f1 (ppm)

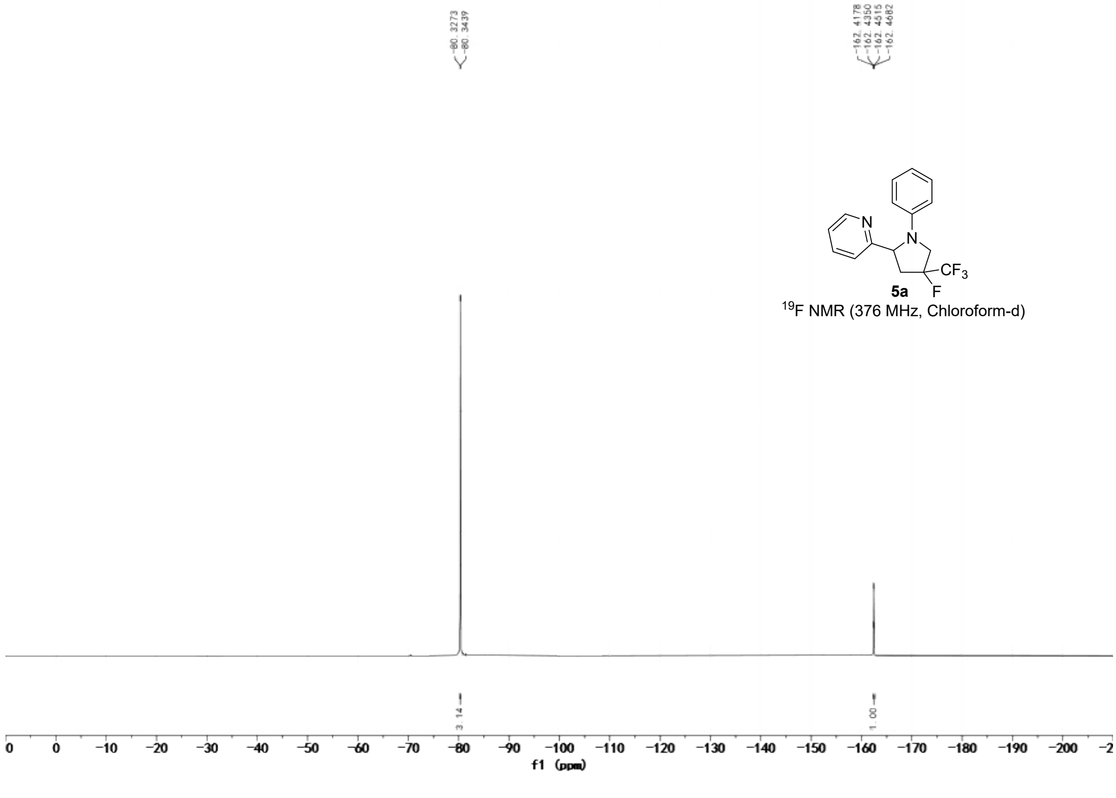
-80.3273
-80.3439
-162.4178
-162.4350
-162.4515
-162.4682
N
N
CF3
F
5a
19F NMR (376 MHz, Chloroform-d)
3.14
1.00
f1 (ppm)

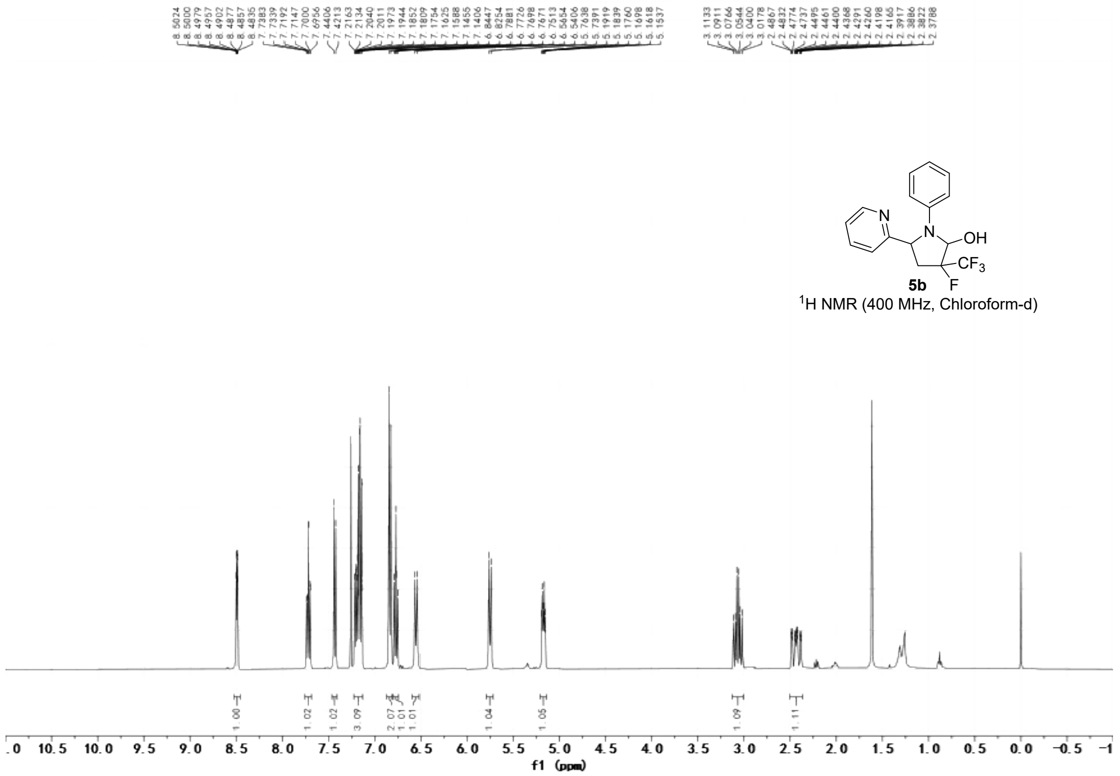
N
OH
CF3
5b
F
1H NMR (400 MHz, Chloroform-d)
f1 (ppm)

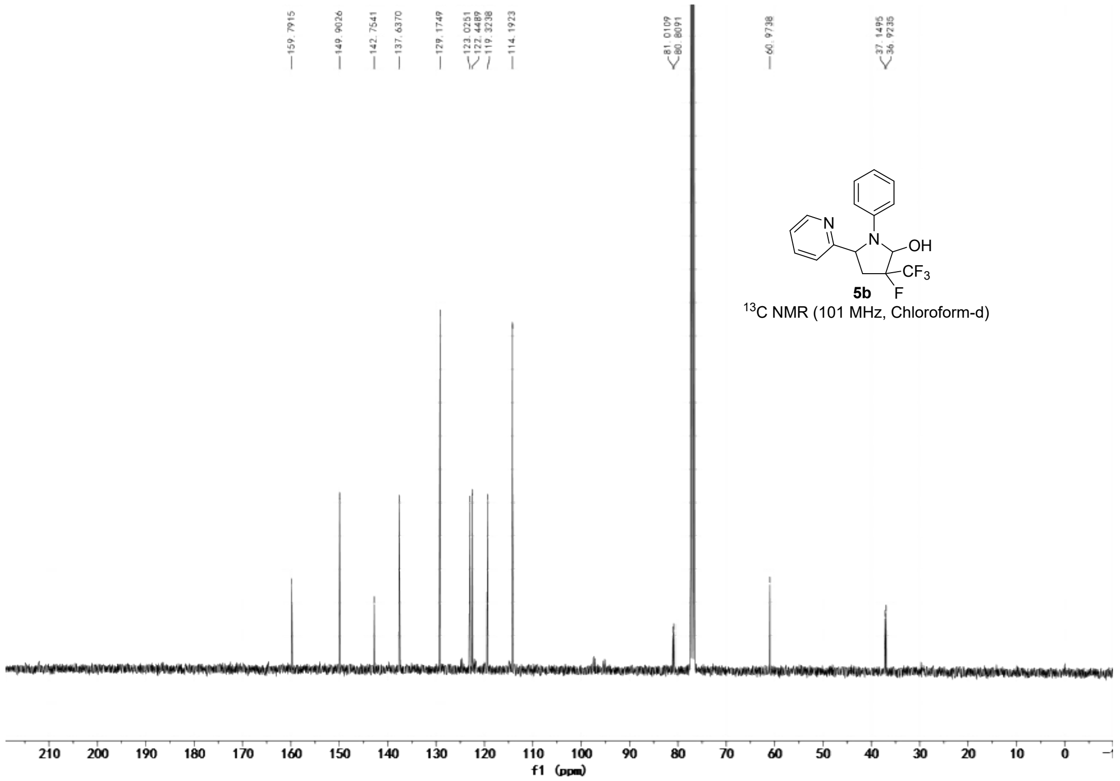
N
OH
CF3
5b
F
13C NMR (101 MHz, Chloroform-d)
f1 (ppm)

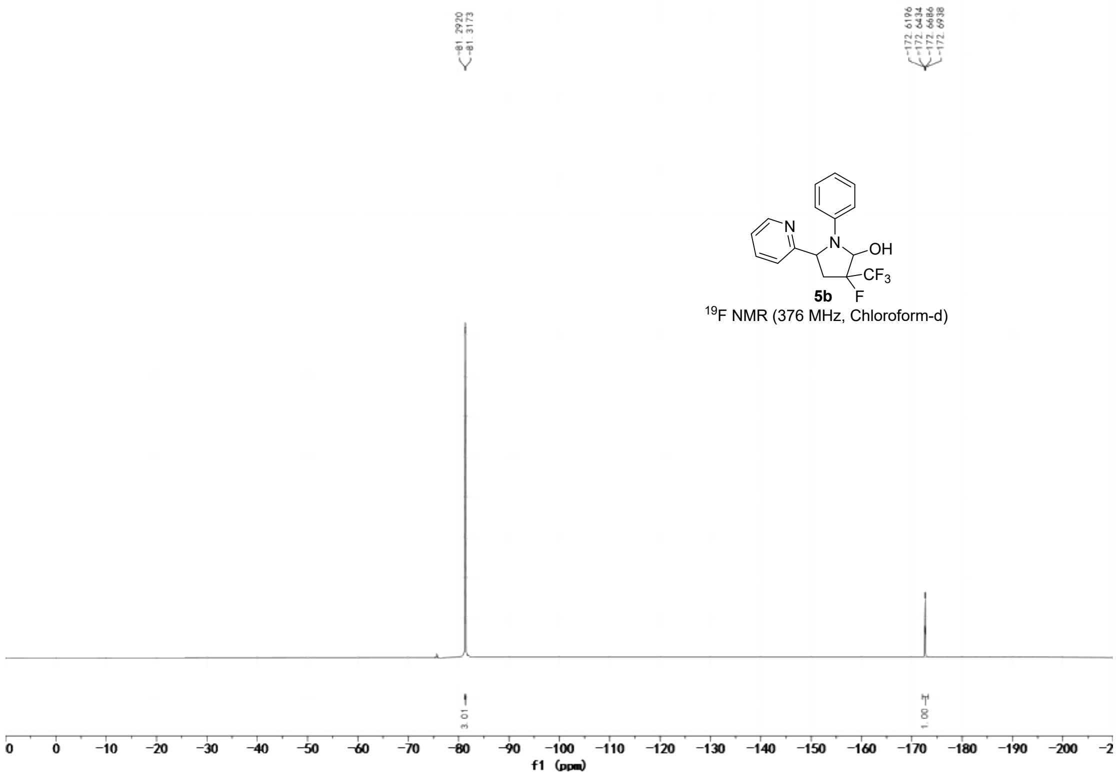

**5b**
$^{19}F$ NMR (376 MHz, Chloroform-d)
